# Synthesis of Benzo[*b*]thiophene 1,1-Dioxides via Pd-Catalyzed Sulfinylation of Aryl Triflates and Their Use as Large Stokes Shift Fluorophores for Multicolor Live-Cell Imaging with Self-Labeling Tags

**DOI:** 10.1021/jacsau.6c00024

**Published:** 2026-04-14

**Authors:** Alexey N. Butkevich, Mariano L. Bossi, Jasmine Hubrich, Stefan W. Hell

**Affiliations:** † Department of Optical Nanoscopy, 28296 Max Planck Institute for Medical Research, Jahnstraße 29, Heidelberg 69120, Germany; ‡ Department of NanoBiophotonics, Max Planck Institute for Multidisciplinary Sciences, Am Faßberg 11, Göttingen 37077, Germany

**Keywords:** Pd catalysis, sulfones, heterocycles, fluorescent dyes, super-resolution microscopy, self-labeling tags

## Abstract

Benzo­[*b*]­thiophene 1,1-dioxide is a relatively underrepresented sulfone-embedded heterocycle with niche uses in material science (OLED emitters), pharmaceutical research (an electrophilic warhead in targeted covalent inhibitors), and as a structural unit in reversibly switchable diarylethene fluorophores. Benzo­[*b*]­thiophene 1,1-dioxides are most commonly prepared by the oxidation of the corresponding benzothiophenes. Here, we propose an alternative approach based on Pd-catalyzed sulfinylation of ortho-carbonyl-substituted aryl triflates followed by *S*-alkylation and Knoevenagel condensation. This methodology allows for an expedient access to diversely substituted benzo­[*b*]­thiophene 1,1-dioxides, including 6-dialkylamino “push-pull” type fluorophores possessing large (>140 nm) Stokes shifts. Fluorescent labels derived from this core structure are compatible with live-cell imaging using self-labeling protein tags (HaloTag, SNAP-tag, and CLIP-tag). Together with the established live-cell fluorescent labels and orthogonal self-labeling tags, they allow for simultaneous observation of up to three intracellular targets below the diffraction limit using fluorescence microscopy with three excitation lasers (485, 561, and 640 nm) and stimulated emission depletion (STED) at 775 nm.

## Graphical abstract

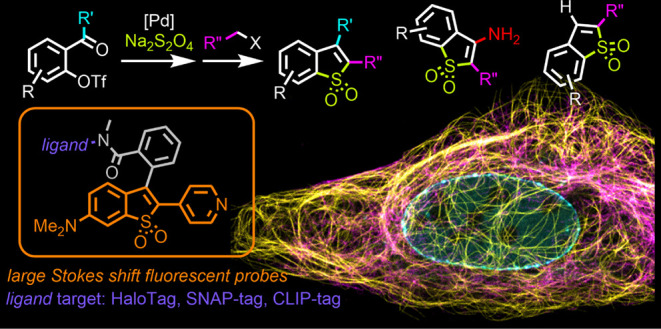

## Introduction

Unlike the parent benzo­[*b*]­thiophene heterocycle, long established in medicinal chemistry,^1^ benzo­[*b*]­thiophene 1,1-dioxides (BTO_2_s) have so far found limited practical applications. In material science, 2,3-diaryl-substituted BTO_2_s demonstrate aggregation-induced fluorescence emission^2^ and have been proposed as electroluminescent materials in organic light-emitting devices (OLEDs) due to their improved film-forming properties as compared to linear oligothiophene dioxides.^3^ More recently, it has been demonstrated that these compounds undergo intramolecular 6π-photocyclization, modulating their optical properties and biological activity.^4^ 2,3-Unsubstituted BTO_2_s are electrophilic species that have been identified as potent covalent inhibitors of phosphoglycerate dehydrogenase (PHGDH), with the regioselectivity of Michael addition of the C421 nucleophile at the allosteric site of PHGDH depending on the substitution pattern of a given BTO_2_.^5^ 2-Substituted BTO_2_s are among the most practically useful aryl substituents in reversibly photoswitchable diarylethene fluorophores (DAEs), especially in those used for fluorescence bioimaging.^6^ Incorporation of BTO_2_ unit allows to achieve the tight balance between emission brightness, photostability, and a high number of switching cycles in aqueous media required in advanced super-resolution fluorescence microscopy techniques such as reversible saturable optical fluorescence transitions (RESOLFT).^7^

By far, the most common method of preparation for arbitrarily substituted BTO_2_s is the oxidation of the corresponding benzo­[*b*]­thiophenes with a variety of suitable reagents (e.g., mCPBA,^8a^ Oxone,^8b^ H_2_O_2_ + acid^8c^). While versatile, this approach limits the scope of compatible substituents to oxidation-insensitive groups, prompting the use of direct oxidative Heck arylation^9a^ or alkenylation^9b^ for late-stage BTO_2_ modification. An arene-fused thiophene *S*,*S*-dioxide ring could be assembled via a cyclization of β-aryl vinyl ether sulfones,^10^ in an oxidative cyclization of arylsulfonyl hydrazides with alkynes,^11^ or in Cu­(I)-catalyzed sulfonylative cyclizations of benzotitanoles (generated *in situ* from aryl Grignard reagents) with SO_2_Cl_2_ ^12^ or of 2-alkynyl-substituted arylboronic acids^13a^ or aryldiazonium salts^13b^ with 1,4-diazabicyclo[2.2.2]­octane bis­(sulfur dioxide) adduct (DABSO). Other reported approaches include photoinduced isomerization of 1,2-benzothiazole-1,1-diones^14^ and Diels–Alder reaction, with 2,5-dibromothiophene-1,1-dioxide as the dienophile.^15^ However, given the substantial CH-acidity of alkyl (p*K*_a_ ∼ 30 in DMSO) and, in particular, of benzyl sulfones (p*K*_a_ ∼ 23),^16^ intramolecular aldol-type condensations of 2-(H, alkyl *or* alkoxy)­carbonyl-^17^ or 2-cyano-aryl alkyl sulfones^18^ remain the best established synthetic venue for BTO_2_ ring closure (see Figure S1).

We recently reported a facile and scalable synthetic approach toward 9*H*-thioxanthen-9-one 10,10-dioxides based on an intramolecular Pd-catalyzed sulfonylative homocoupling of 2,2′-dihydroxybenzophenone ditriflates with sodium dithionite.^19^ During the course of the study, it was noticed that a simple variation of the same reaction generated 2-(aroyl)­benzenesulfinic acids as stable intermediates that could be trapped with an electrophilic alkylating reagent and isolated in the form of 2-(aroyl)­aryl alkyl sulfones. These latter compounds are ideally positioned to serve as starting materials for a diverse variety of BTO_2_s (Scheme 1), and the synthesis of two sample series of BTO_2_s via Pd-catalyzed sulfinylation, investigation into their photophysical properties, and their application as live-cell compatible fluorescent labels with large Stokes shifts are described in the present work.

**Scheme 1:**
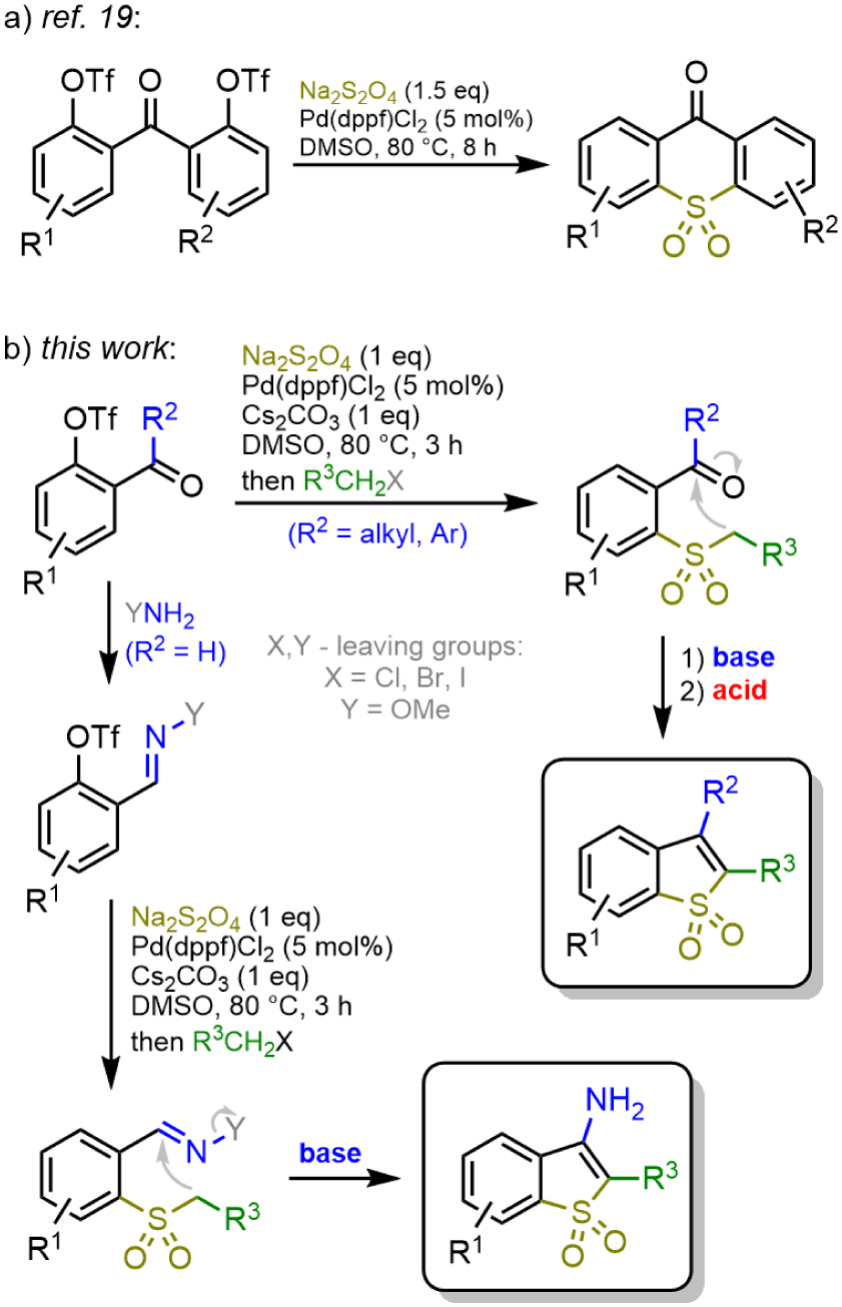
Synthetic Approaches to Sulfone-Embedded Heterocycles via (a) Pd-Catalyzed Sulfonylative Homocoupling and (b) Tandem Pd-Catalyzed Sulfinylation/Alkylation

## Results and Discussion

### Synthesis and Substrate Scope of 3-Substituted Benzothiophene 1,1-Dioxides (**3a**–**3ad**)

Initiating the studies from the conditions originally identified for Pd-catalyzed sulfinylation of 2-hydroxybenzophenone triflate,^19^ we first aimed at generalizing this method of arylsulfinic acid generation to diverse structural contexts. As we had found, a coordinating acyl group (−COR^2^) was required for the transformation to proceed. Free arylsulfinic acids are polar compounds and may be unstable against oxidation and disproportionation^20^ and thus are generally impractical to isolate. We therefore sought to stabilize them by the addition of an external base and performing subsequent alkylation in a one-pot, two-step procedure. Indeed, the addition of 1 equiv of Cs_2_CO_3_ increased the reaction rate, achieving maximal conversion of aryl triflate **1a** into the corresponding sulfinate salt within 3 h at 80 °C (in DMSO with 5 mol % Pd­(dppf)­Cl_2_ catalyst, monitored by *in situ* IR spectrometry and HPLC-MS, Figure S2). The sulfinate salts generated in this way were treated with 1.1–1.2 equiv of an alkylating reagent, generating (usually in high yield) the intermediate sulfones **2a**–**2ad** (see SI), which were isolated in the crude state or purified whenever feasible. Upon treatment with a sufficiently strong base (NaHMDS with p*K*_a_ = 26 for its conjugate acid in THF was satisfactory in most cases), the sulfones rapidly underwent an aldol condensation, converting into diastereomeric mixtures of cyclic β-hydroxy sulfones **2′** (a sample known compound **2′a** ^17a^ was isolated; see SI). These crude mixtures were subjected to acid treatment, producing the desired BTO_2_s in moderate to good isolated yields for most examples (Figure 1).

**Figure 1:**
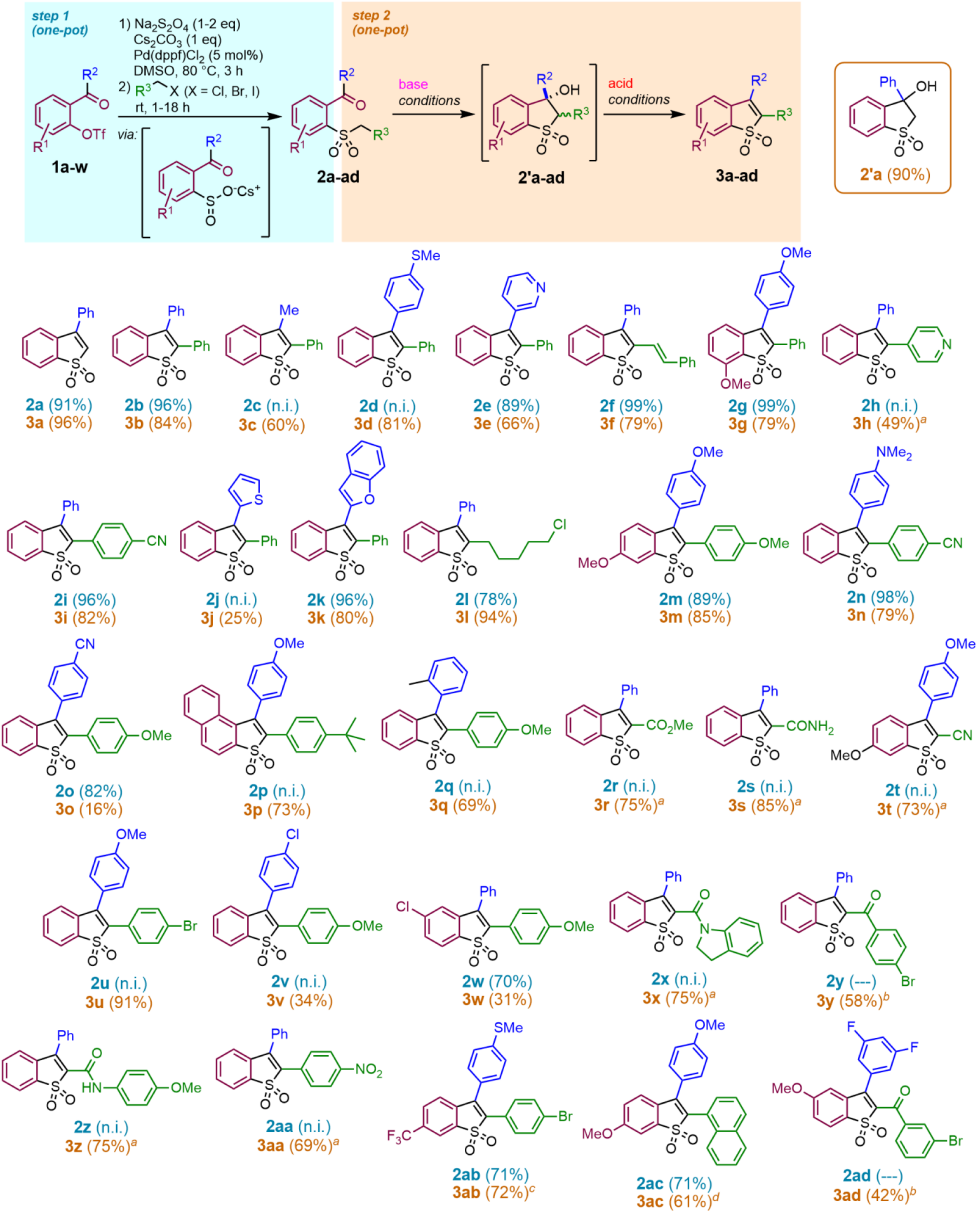
Synthesis of 3-substituted benzothiophene 1,1-dioxides (**3a–3ad**): substrate scope. Standard base treatment: NaHMDS (1.1 equiv), THF, 0 °C to rt, 30 min; standard acid treatment: MsOH, AcOH, 80 °C, 30 min. ^*a*^ DBU (2 equiv) used as a base. ^*b*^ No base treatment was necessary (**2y,ad** converted into **3y,ad** under step 1 conditions). ^*c*^ Acid: MsOH, AcOH, 80 °C, and 12 h. ^d^ Base: NaHMDS (1.6 equiv), rt, 24 h; n.i. = not isolated.

While the reaction sequence demonstrated reasonable tolerance of electron-donating (**3g,k,m,t**) and electron-withdrawing substituents (**3ab**) and moderate steric hindrance (**3p,q**,**ac**), its compatibility with aryl halides in the benzophenone fragment appears limited (**3v,3w**). The tolerated presence of a methylthio substituent in the substrates **1d,ab** leading to the products **3d** and **3ab** in overall 81% and 51% yield, respectively, highlighted the value of our approach versus the standard late-stage oxidation of benzo­[*b*]­thiophene; however, the 2-thienyl substituted substrate **1j** provided the product **3j** in only 25% isolated yield. Besides the (hetero)­aryl-substituted compounds, BTO_2_s with 3- and 2-alkyl (**3c,l**) and 2-alkenyl (**3f**) substituents could be assembled using the proposed strategy. With the stronger electron-withdrawing R^3^ groups, a weaker base (DBU, p*K*_a_ = 16.6 in THF^21^) was found sufficient to induce aldol condensation (**3r-t,x,z,aa**), and the most CH-acidic intermediate β-keto sulfones **2y,ad** converted into the target BTO_2_s **3y,ad** with dehydration directly under the conditions of step 1 and could not be identified in the reaction mixture.

### Synthetic Access to 3-Unsubstituted Benzothiophene 1,1-Dioxides

In stark contrast to 2-acylphenol triflates (Scheme 1b, R^2^ = alkyl or aryl), the corresponding salicylaldehyde derivatives (R^2^ = H) were unreactive in the Pd-catalyzed sulfinylation reaction. We hypothesized that installing a temporary coordinating group, such as an oxime,^22a^ an imine^22b^ or a hydrazone,^22c^ would ensure coordination of a catalytic Pd(0) species and thus promote an oxidative addition of the aryl triflate and generation of the intermediate arylsulfinic acid (Scheme 2a). Indeed, both the *N*,*N*-dimethyl hydrazone **7** and the *O-*methyl oxime **10a** underwent the desired transformation, affording, upon alkylation with benzyl bromide, the corresponding aryl benzyl sulfones (**8**, **11a**). Due to the limited hydrolytic stability of *N-*aryl imines, the corresponding sulfone product generated from the aryl imine-protected salicylaldehyde triflate **9** underwent partial hydrolysis under the reaction conditions and readily hydrolyzed to give free aldehyde **5a** on treatment of the crude mixture with dilute (0.5 N) hydrochloric acid in 67% yield (*method A*). Deprotection of *N,N*-dimethylhydrazone **8** proceeded quantitatively but required a superstoichiometric amount of a strong acid and was realized in a heterogeneous manner using a cation exchanger in hydrogen form (*method B*). On the contrary, the same product **5a** could be recovered from *O*-methyl oxime **11a** acid in 68% yield by milder treatment with excess glyoxylic acid (*method C*). Finally, **5a** was converted to 3-unsubstituted BTO_2_ **6a** employing the sequential base-acid treatment established above.

**Scheme 2:**
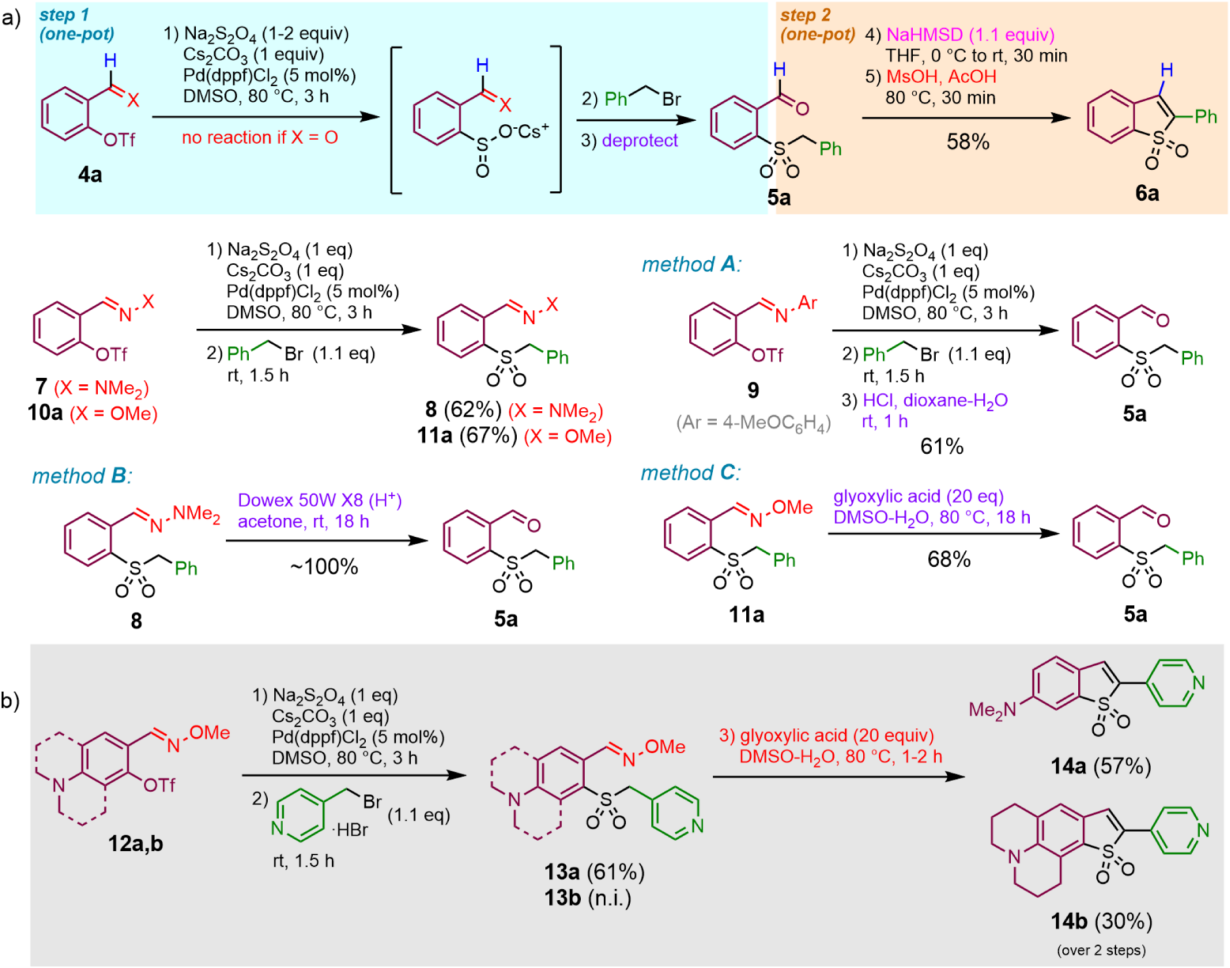
Synthetic Approach to 3-Unsubstituted Benzothiophene 1,1-Dioxide **6a** (A) and to Large Stokes Shift Fluorophores (**14a,b**) with BTO_2_ Core (B)

Relying on the latter synthetic sequence, and combining an electron-donating *N*,*N*-dialkylamino substituent in the 6-position of BTO_2_ with an electron-withdrawing pyridine substituent in position 2, the push–pull type large Stokes shift fluorophores **14a,b** could be constructed (Scheme 2b). These compounds represent BTO_2_ analogues of well-established large Stokes shift coumarin fluorescent dyes (e.g., Coumarins 6, 7, 30, 525, 545), which have been evaluated as lasing media in dye lasers^23^ and fluorescent reporters in bioimaging.^24^ Further modification of these lipophilic push–pull BTO_2_ cores via quaternization of pyridine nitrogen and introduction of anionic sulfonate or phosphate groups^25^ may provide large Stokes shift labels similar to commercial dyes (*abberior STAR 520L*, *Dyomics DY-485XL*, or *DY-511XL*) suitable for protein or DNA labeling and potentially applicable for fluorescence *in situ* hybridization (FisH) microscopy or in microarray technology.

### Synthesis of 3-Aminobenzothiophene 1,1-Dioxides (**15a–15o**)

Treatment of alkyl aryl sulfones bearing a carbaldehyde *O*-methyl oxime group in the 2-position of the aromatic ring with a base of sufficient strength resulted in a rapid ring closure with the formation of 3-aminobenzo­[*b*]­thiophene 1,1-dioxides (Scheme 1b). Similarly to the transformation shown in Figure 1, a stoichiometric amount of NaHMDS was sufficient in most cases, while more C–H acidic sulfones **11d,l** generated the BTO_2_s **15d,l** upon the addition of DBU (2 equiv) to the isolated (**11l**) or crude sulfone (**11d**). The scope of this transformation is illustrated in Figure 2.

**Figure 2:**
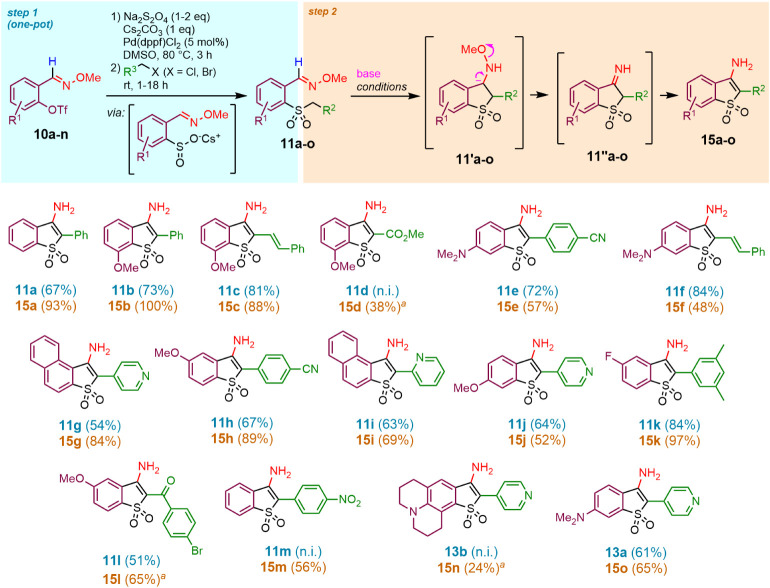
Synthesis of 3-aminobenzothiophene 1,1-dioxides (**15a**–**15o**): substrate scope. Standard base treatment: NaHMDS (1.1 equiv), THF, 0 °C to rt, 30 min. *^a^* DBU (2 equiv) used as a base. n.i. = not isolated.

### Synthesis of the Large Stokes Shift Fluorescent Dye 20 and Photophysical Characterization of Benzothiophene 1,1-Dioxide Fluorophores

Before evaluating the performance of the newly designed push–pull fluorophores in live-cell multicolor fluorescence imaging, we first studied the photophysical and photochemical properties of the best core candidates **14a,b**. In particular, to establish a three-color imaging scheme using a commercial fluorescence microscope without detection channel splitting or spectral unmixing, a selective excitation of the large Stokes shift label with a blue laser (e.g., 485 nm) and minimal cross-excitation with a green laser (e.g., 561 nm) under physiological conditions is a prerequisite (Figure S3). The main advantage of using a large Stokes shift dye instead of a small Stokes shift dye with an absorption maximum in the 488–520 nm range lies in avoiding the need for an additional STED laser (e.g., 595 nm), which would rapidly photobleach the other two fluorophores due to significant excitation cross-section at this wavelength. The presence of a basic pyridine nitrogen in the “pull” fragment required assessment of the pH-dependent behavior of the dye within the physiological range (5 < pH < 10 for different organelles). Unlike **14b**, the fluorophore **14a** did not demonstrate significant absorption at 561 nm in its nonprotonated state (at pH > 5, see Figure S4), and we selected this core for further modification into a large Stokes shift fluorescent dye **20** bearing a free carboxylic acid group suitable for coupling with a protein target-specific ligand. The compound **20** was synthesized according to the scheme in Figure 3,a, starting from the known precursor **16** (see SI) and relying on the Pd-catalyzed sulfinylation/alkylation sequence investigated above (Figure 1). BTO_2_ ring closure of intermediate compound **19** proceeded with concomitant formal hydrolysis of the methyl ester, which could be explained by an intramolecular attack of the intermediate tertiary alkoxide on the ester carbonyl group, followed by deprotonation of the resulting spirolactone and lactone ring opening in retro-Michael fashion. The resulting free carboxylic-acid-bearing dye **20** preserved the spectral characteristics of its parent fluorophore **14a** and, similar to rhodamine dyes, did not seem to undergo lactone ring closure in polar protic solvents such as methanol (see Figure S5). It however manifested significantly lower emission quantum yield and biexponential fluorescence lifetime, with the desired longer lifetime component (τ > 1 ns) being minor, likely due to free rotation of the 2-carboxyphenyl substituent, which is significantly less hindered compared to the pendant 2-carboxyphenyl substituent in rhodamine dyes. Photophysical properties of BTO_2_s **3** found to be fluorescent in solution, and the large Stokes shift fluorophores **14a**, **14b** and **20** are compiled in Table S1.

**Figure 3:**
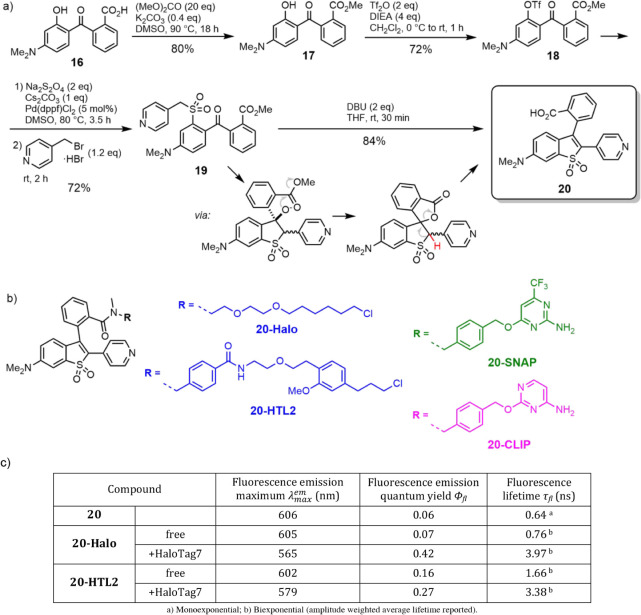
Synthesis of large Stokes shift fluorescent dye **20** (a), structures of fluorescent ligands for self-labeling tag proteins HaloTag, SNAP-tag, and CLIP-tag (b), and photophysical properties of the dye **20** and ligands **20-Halo** and **20-HTL2** measured in PBS (pH 7.4) free and upon covalent binding to HaloTag7 protein (c).

### Synthesis of Fluorescent Ligands for Self-Labeling Tags and Live-Cell Multicolor Confocal and STED Imaging

Having thus identified candidate dye **20** as the live-cell-compatible large Stokes shift fluorophore suitable for 485 nm excitation, our first concern was to exclude its possible adverse effects on mammalian cell viability. To negate the potential effects of the free carboxylate group of **20** on cell membrane permeability and intracellular distribution, we decided to perform cytotoxicity testing on the final targeted ligands applicable for imaging. As targets, we have chosen HaloTag,^26^ SNAP-tag,^27^ and CLIP-tag^28^ as mutually orthogonal self-labeling proteins, which could be coexpressed in living cells as fusions to the arbitrary proteins of interest, followed by selective staining via *in cellulo* conjugation with suitably designed fluorescent ligands.

For the HaloTag protein–ligand pair, we have selected the classic HaloTag­(O2) 6-chlorohexyl-PEG_2_ linker (with an added *N*-methyl group to prevent cyclization of the probe into a colorless spirolactam form – a known complication in the rhodamine amide series leading to blinking behavior, undesired in our case;^29^ see Figure S6) and the HaloTag7 version of the tag protein with improved labeling kinetics.^30^ Since all HaloTag protein versions were derived from an engineered ω-haloalkane dehalogenase originally optimized for its fastest reaction kinetics with rhodamine-type ligands, our concern was the possible loss of the rate of labeling with ligand **20-Halo**, well documented in a series of nontriarylmethane type HaloTag ligands.^31^ Fortunately, a recently designed second-generation HaloTag ligand, HTL.2,^32^ has been reported to achieve fast covalent binding of diverse ligands in a cargo-agnostic way and was demonstrated to work efficiently with fluorophores unsuitable for labeling with original HaloTag­(O2) derivatives.^33^ After some optimization aimed at reducing the number of synthetic steps compared to the original report (see SI), we prepared an *N*-methylated version of HTL.2 ligand and the corresponding large Stokes shift fluorescent label **20-HTL2**. Since the brightness of free dye **20** in solution was deemed unsatisfactory, we have compared the emission properties of **20-Halo** and **20-HTL2** in their free state and bound to the target protein (Figure 3,c). We observed a large signal enhancement upon binding, prompted by an increase of the fluorescence quantum yield and fluorescence lifetime, accompanied by a small hypsochromic shift of the emission (see Figure S7 for the results and for the confirmation of covalent binding), with the fluorescence quantum yield of the bound dye (0.27–0.42) now comparable to that of the previously validated live-cell large Stokes shift fluorophore *SiX* (0.28).^34^

For SNAP-tag and CLIP-tag proteins (both engineered versions of *O* ^6^-alkylguanine-DNA alkyltransferase, specific respectively for *O* ^6^-benzylguanine and *O* ^2^-benzylcytosine ligands), we selected their faster-acting versions SNAPf and CLIPf.^35^ While the fluorescent ligand **20-CLIP** was generated from *N*-methylated modification of the original CLIP-tag ligand *O* ^2^-(4-(aminomethyl)­benzyl)­cytosine, we relied on a recently reported refinement of the SNAP-tag ligand^36^ based on the replacement of the hydrophilic guanine fragment with a more lipophilic 2-amino-4-(trifluoromethyl)­pyrimidine residue. In our preliminary tests, the resulting **20-SNAP** label demonstrated markedly improved intracellular labeling efficiency at low label concentrations (30–100 nM).

The biocompatibility of all four fluorescent labels (Figure 3,b) was evaluated in a CellTiter-Glo 2.0 luminescent cell viability assay, quantifying the ATP content of metabolically active cells. The assay was performed on human bone osteosarcoma epithelial (U-2 OS) cells across a range of concentrations suitable for cellular imaging (0.37–10 μM) in complete cell culture medium over 24 h (see Figure S8). While no toxic response was noticed for any of the probes derived from **20** within this range, we limited the concentrations of fluorescent labels in the media to ≤2 μM.

To assess the applicability of large Stokes shift labels in live-cell multicolor fluorescence imaging, we have selected CRISPR-engineered U-2 OS-Vim-Halo cells^37^ with stable expression of fused HaloTag-Vimentin protein localized in intermediate filaments of the cytoskeleton and CRISPR-engineered U-2 OS HaloTag-Lamin A/C^38^ cells expressing a HaloTag fusion of lamin A/C, another intermediate filament protein with high longevity and predominant localization in the nuclear lamina.^39^ An orthogonal self-labeling tag (SNAP-tag, CLIP-tag, or dHaloTag7, a single point-mutated HaloTag7­(D106A) reversibly binding ω-hydroxyalkane analogues of HaloTag­(O2) ligand and retaining the fluorogenic response of the original HaloTag to Si-rhodamine derivatives^40^) was then introduced via transfection with a suitably designed plasmid (see SI). In three-color imaging, a third fluorescent ligand with a noncovalent selective targeting group (a taxoid derivative binding to polymerized tubulin^41^ or Hoechst 33342 conjugate binding to dsDNA and highlighting nuclear heterochromatin^42^) was employed. The small Stokes shift fluorophores complementary to **20** were selected based on their selective excitation with 561 nm (the derivatives of a carborhodamine dye 580CP,^43^ such as *abberior LIVE 590*) and 640 nm (Si-rhodamine^44^ derivatives), and clear color separation was achieved relying on the previously established three-color imaging scheme^34^ (see Figure S9). As a control, we first performed labeling in U-2 OS-Vim-Halo cells using the previously reported large Stokes shift *SiX* fluorophore,^34^ for which two HaloTag ligands (*SiX-Halo* and *SiX-HTL2*, see SI) were prepared. In accordance with our expectations, both labels failed to provide specific labeling of vimentin filaments and instead accumulated in endosomes (Figure S10).

On the contrary, with our selection of complementary fluorescent labels, we were able to validate the proposed large Stokes shift fluorescent markers based on dye **20** in living U-2 OS cells for a variety of target combinations. Thus, we observed selective staining and clear color separation in U-2 OS-Vim-Halo cells transfected with pSNAPf-GianCreg (introducing a self-labeling tag fusion of a Golgi membrane protein) labeled with **20-HTL2** and SNAP-tag and tubulin ligands conjugated orthogonally to *abberior LIVE 590* and Si-rhodamine (Figure S11). An alternative staining scheme, with **20-SNAP** used for labeling SNAPf-GianCreg fusion protein and *abberior LIVE 590 tubulin* and SiR-Halo^44^ labeling the tubulin and vimentin filaments, respectively, is shown in Figure 4A–D,H,I (nuclei are counterstained with Hoechst 33342). In the same cell line, clathrin-coated vesicles could be visualized together with vimentin cytoskeleton upon transfection with pSNAPf-Clathrin^45^ and double staining with **20-Halo** and SiR-SNAP^44^ ligands (counterstained with Hoechst 33342 for visualization of cell nuclei, Figure S12). Alternatively, cell mitochondria could be visualized along with HaloTag-fused vimentin and tubulin constituents of the cytoskeleton by transfecting these cells with pCLIP-Cox8A plasmid (targeting CLIP-tag ligand SiR-CLIP^44^ to mitochondrial cytochrome c oxidase 8A, which forms part of the mitochondrial transmembrane complex IV) in two (Figure S13) or three-color combinations (Figure 4E–G,J). Compatibility of **20** with CLIP-tag technology was verified by selective costaining of U-2 OS HaloTag-Lamin A/C cells transfected with pCLIPf-Cox8A with **20-CLIP** and SiR-Halo^44^ ligands (Figure S14).

**Figure 4:**
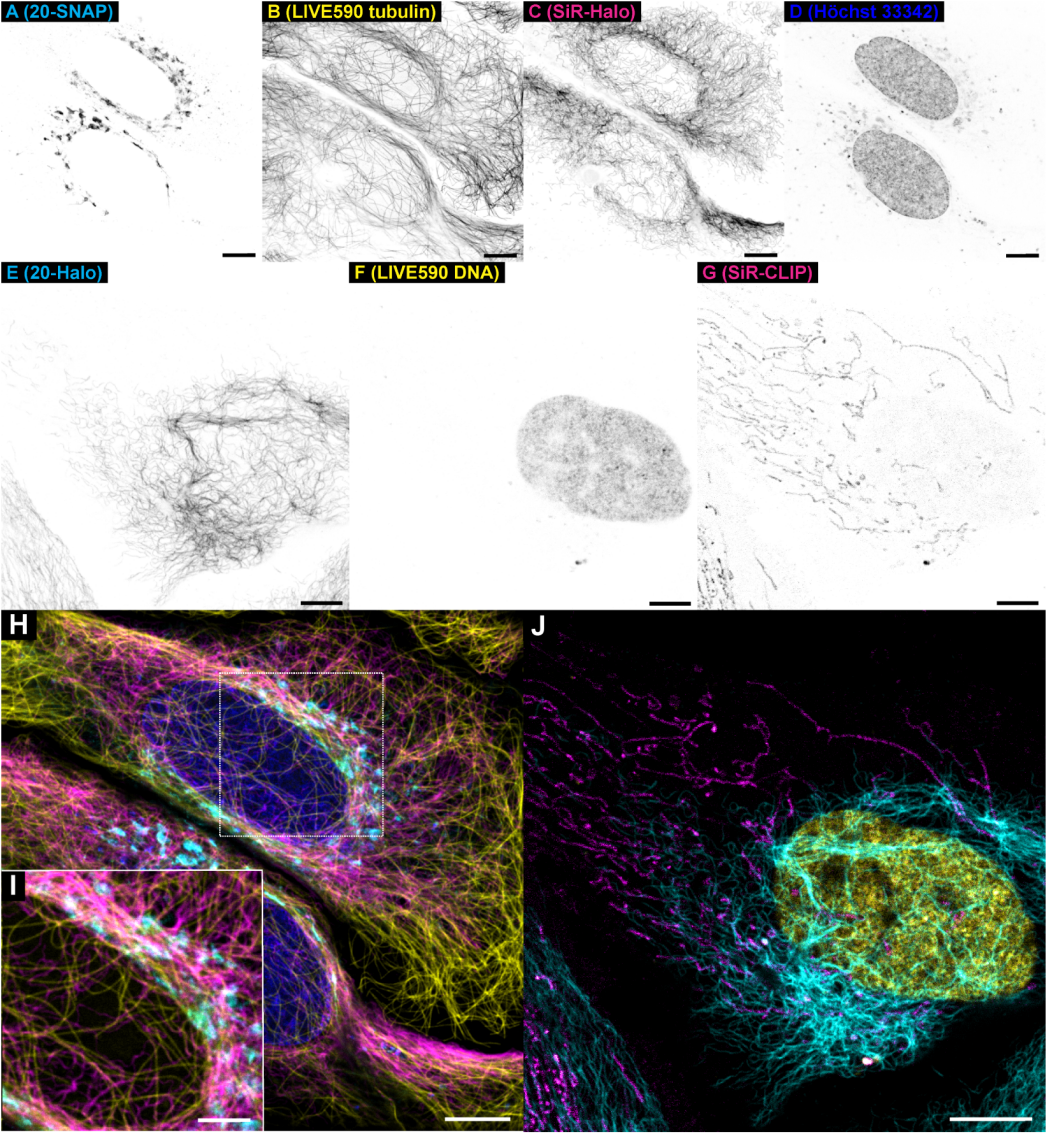
Live-cell multicolor confocal imaging with large Stokes shift labels **20-Halo** and **20-SNAP** and orthogonal self-labeling tags. (A–D) Four-color image of living U-2 OS-Vim-Halo transfected with pSNAPf-GianCreg and labeled with **20-SNAP** (1 μM over 2 h, Golgi apparatus), *abberior LIVE 590 tubulin* (500 nM over 1 h, β-tubulin), SiR-Halo (500 nM, 2 h, vimentin), and Hoechst 33342 (8 μM, DNA). (E–G) Three-color image of living U-2 OS-Vim-Halo transfected with pCLIPf-Cox8A and labeled with **20-Halo** (1 μM over 16 h, vimentin), *abberior LIVE 590 DNA* (500 nM over 1 h, DNA), and SiR-CLIP (200 nM over 16 h, mitochondria). (H, I) Four-color overlay image of individual channels shown in panels (A–D) with the three-color expansion of a perinuclear region in (I). (J) Three-color overlay image of individual channels shown in panels (E–G). Scale bars: 10 μm (A–H, J), 5 μM (I).

We also investigated the staining of self-labeling tag-fused lamins forming a distinct intracellular structure at the nuclear envelope, suitable for benchmarking of labeling selectivity using super-resolution microscopy techniques.^46^ To this end, in one experimental approach, a U-2 OS HaloTag-Lamin A/C cell line stably expressing the lamin A/C-HaloTag fusion protein was treated with **20-Halo** and an *abberior LIVE 590* or a Si-rhodamine tubulin ligand (Figure S15). Alternatively, U-2 OS-Vim-Halo cells were transfected with pSNAPf-LaminA-C, and the two expressed orthogonal tags (HaloTag and SNAP-tag) were labeled with a reversibly (SiR-T5 ^40^) or an irreversibly binding fluorescent marker (*abberior LIVE 590 SNAP*), with large Stokes shift labels **20-Halo** and **20-SNAP** employed for visualization of vimentin or lamin, respectively. To achieve three-color combinations, fluorescent staining of tubulin with *abberior LIVE 590 tubulin* or SiR-tubulin^47^ was additionally introduced (Figure S16).

Finally, we demonstrated the applicability of BTO_2_-based large Stokes shift fluorescent labels in multicolor fluorescence imaging with 775 nm stimulated emission depletion (STED), permitting to resolve structural features of multiple targets with resolution below the diffraction limit.^48^ U-2 OS-Vim-Halo cells, transfected with a plasmid expressing a SNAP-tag or a CLIP-tag fusion protein: clathrin (Figure 5A–E) or lamin A (Figure 5F–J, Figure S15D,H) for SNAP-tag and Cox8A for CLIP-tag (Figure S13,D) – were incubated overnight with 1 μM of large Stokes shift fluorescent ligand **20-Halo** or **20-SNAP** and 200 nM of the corresponding small Stokes shift fluorescent probe. After washing samples with dye-free medium, it was replaced with fresh medium containing a noncovalent fluorescent probe for the complementary third color channel (*abberior LIVE 590 tubulin* or SiR-tubulin), and the samples were imaged live after 1 h incubation. Alternatively, an exchangeable HaloTag ligand SiR-Hy4 ^40^ reversibly binding to dHaloTag7-lamin A fusion protein could be visualized orthogonally to vimentin, irreversibly labeled with **20-Halo** in U-2 OS-Vim-Halo cells (along with *abberior LIVE 590 DNA* for three-color imaging, Figure S17). Further improvement of the photostability and brightness of live cell-compatible large Stokes shift probes for fluorescence nanoscopy, as well as the development of their fluorogenic versions for reversibly binding exchangeable HaloTag ligands such as T5 and Hy4, will form the basis of future work.

**Figure 5:**
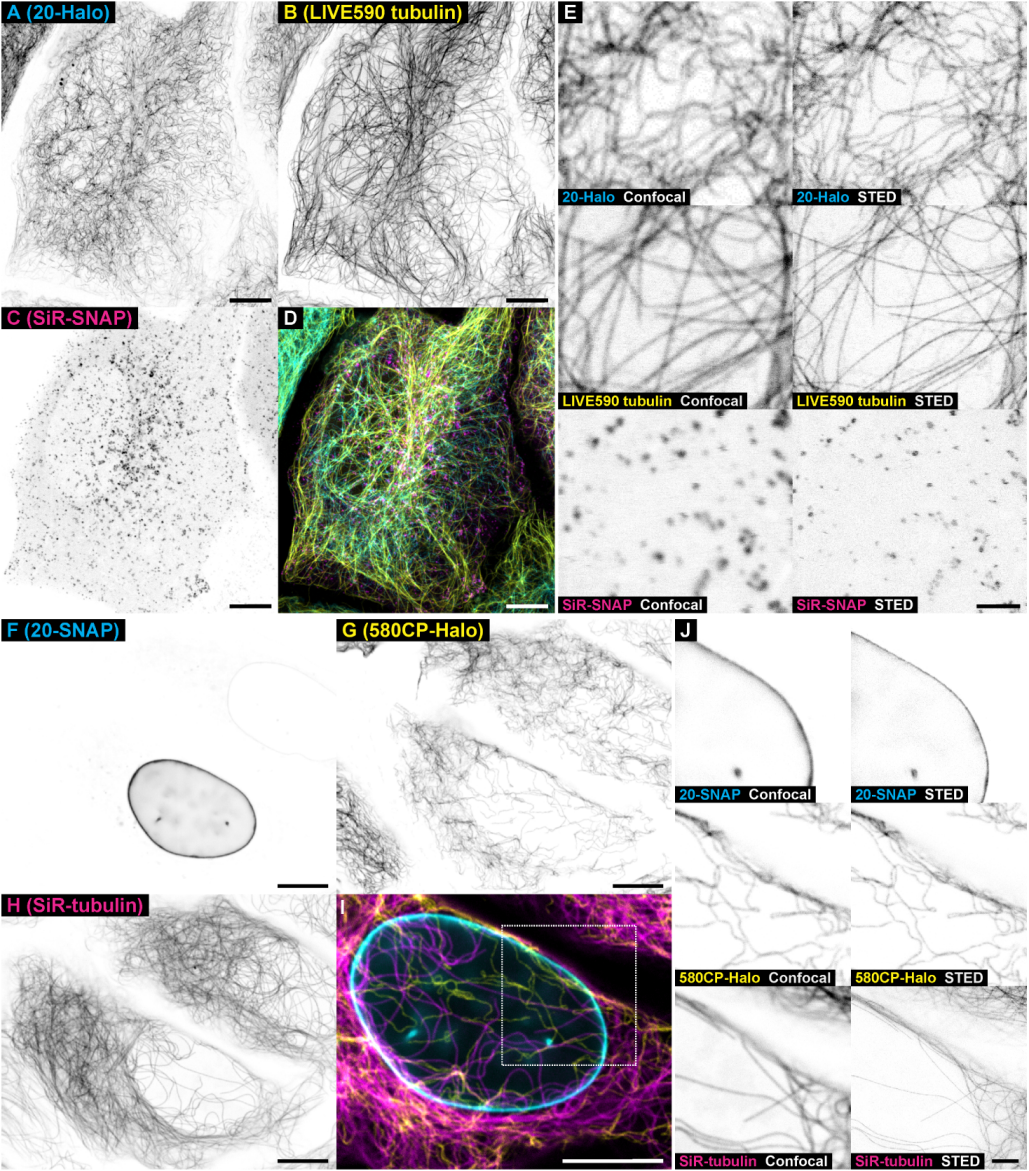
Live-cell multicolor super-resolution imaging with large Stokes shift labels **20-Halo** and **20-SNAP** and orthogonal self-labeling tags. (A–E) Three-color image of living U-2 OS-Vim-Halo transfected with pSNAPf-Clathrin and labeled with **20-Halo** (1 μM over 16 h, vimentin), *abberior LIVE 590 tubulin* (500 nM over 1 h, β-tubulin), and SiR-SNAP (200 nM, 16 h, clathrin-coated vesicles). (F–J) Three-color image of living U-2 OS-Vim-Halo transfected with pSNAPf-LaminA-C and labeled with **20SNAP** (1 μM over 16 h, lamin A), 580CP-Halo (200 nM over 16 h, vimentin), and SiR-tubulin (500 nM, 1 h, with the addition of 10 μM verapamil; β-tubulin). Scale bars: 10 μm (A–D and F–I), 2 μM (E, J).

## Conclusions

In our work, we propose a general synthetic approach toward benzo­[*b*]­thiophene 1,1-dioxides (BTO_2_s), increasing the accessibility of this relatively underrepresented heterocyclic unit. A diversity of substitution patterns may be achieved by choosing one of the established substrates (**1**, **7**, **9**, **10** – all of which are chemically stable), the conditions of the transformations are moderate, and a very common and relatively inexpensive Pd­(dppf)­Cl_2_ catalyst was employed in all cases. As opposed to the common synthetic strategy, the synthesis of BTO_2_s does not involve a (benzo)­thiophene oxidation step, thus increasing the number of compatible functional groups.

We applied the proposed synthetic method to the preparation of large Stokes shift fluorophores **14a,b** and introduced the fluorescent dye **20** as a possible solution, addressing the absence of large Stokes shift fluorescent markers compatible with self-labeling protein tags now routinely used in bioimaging. The derived labels **20-Halo**, **20-HTL2**, **20-CLIP**, and **20-SNAP** were found to be biocompatible and cell membrane-permeant and hence suitable for fluorescence imaging of subcellular structures of interest in living mammalian cells. Moreover, the labeling of target structures was sufficiently complete and stable to permit super-resolution STED imaging with 775 nm depletion wavelength. Good excitation peak separation of large Stokes shift probes derived from **20** from the common carbo- and Si-rhodamine fluorescent dyes allows simultaneous three-color imaging with commercial fluorescence microscopes using previously established imaging schemes. We therefore believe that newly proposed fluorescent labels demonstrate immediate potential for chemical biology and medicinal chemistry applications, in particular, for monitoring interactions between multiple labeled biomolecules in the complex context of living cells with subdiffraction resolution.

## Supplementary Material





## Notes

Additional experimental details, materials, methods, imaging parameters for confocal and STED images, characterization data for all new compounds and engineered plasmids Snapf-GianCreg and dHalo-LaminA, including Figures S1–S19 and Tables S1–S2­(PDF)
NMR spectra (PDF)

## #

Facility for Synthetic Chemistry, Max Planck Institute for Multidisciplinary Sciences, Am Faßberg 11, 37077 Göttingen, Germany. E-mail: alexey.butkevich@mpinat.mpg.d
