# Supplementary material for: Synthesis of Benzo[b]thiophene 1,1-Dioxides via Pd-Catalyzed Sulfinylation of Aryl Triflates and Their Use as Large Stokes Shift Fluorophores for Multicolor Live-Cell Imaging with Self-Labeling Tags
Source: JACS Au. 2026 Apr 14;6(4):2396–406. doi: 10.1021/jacsau.6c00024 (PMC13126164; doi:10.1021/jacsau.6c00024)
Supplement: Supplementary file 1 [file au6c00024_si_001.pdf]

Supplementary information

Synthesis of benzo[*b*]thiophene 1,1-dioxides via Pd-catalyzed sulfinylation of aryl triflates and their use as large Stokes shift fluorophores for multicolor live-cell imaging with self-labeling tags

Alexey N. Butkevich[a†*], Mariano L. Bossi[a], Jasmine Hubrich[a] and Stefan W. Hell[a,b*]

[a] Department of Optical Nanoscopy, Max Planck Institute for Medical Research, Jahnstraße 29, 69120 Heidelberg, Germany.

[b] Department of NanoBiophotonics, Max Planck Institute for Multidisciplinary Sciences, Am Faßberg 11, 37077 Göttingen, Germany.

[†] Present address: Facility for Synthetic Chemistry, Max Planck Institute for Multidisciplinary Sciences, Am Faßberg 11, 37077 Göttingen, Germany.

* Corresponding authors' e-mails: alexey.butkevich@mr.mpg.de, stefan.hell@mpinat.mpg.de.

Table of Contents

Supplementary Tables.. 8

Table S1. Photophysical properties of compounds **3** and **14**.. 8

Table S2. Imaging parameters for confocal and STED images. 10

Supplementary Figures 11

Figure S1. Synthetic approaches to benzo[*b*]thiophene 1,1-dioxides 11

Figure S2. *In situ* IR spectroscopy of Pd-catalyzed sulfinylation of **1a**.................................. 13

Figure S3. Absorption and fluorescence emission spectra of compounds **14a** and **14b** in chloroform and methanol. 14

Figure S4. Normalized absorption spectra of compounds **14a**, **14b**, **20** and **20-Halo** in 100 mM phosphate buffer at different pH values.. 15

Figure S5. Absorption and fluorescence emission spectra of compound **20**......................... 16

Figure S6. pH-dependent equilibria of the large Stokes dye **20** and its tertiary amide ligands (exemplified by **20-Halo**).. 17

Figure S7. Fluorescence enhancement of **20-Halo** and **20-HTL2** (500 nM) upon binding to excess HaloTag7 protein in PBS (pH 7.4)... 18

Figure S8. Influence of probes **20-Halo**, **20-HTL2**, **20-CLIP** and **20-SNAP** on viability of U-2 OS cells determined in a CellTiter-Glo 2.0 assay 19

Figure S9. General multichannel imaging scheme. 20

Figure S10. Confocal image of living U-2 OS-Vim-Halo cells labeled with *SiX-Halo* or with *SiX-HTL2* 21

Figure S11. Confocal image of living U-2 OS-Vim-Halo cells transfected with pSNAPf-GianCreg 22

Figure S12. Confocal image of living U-2 OS-Vim-Halo cells transfected with pSNAPf-Clathrin 23

Figure S13. Confocal and STED image of living U-2 OS-Vim-Halo cells transfected with pCLIP-Cox8a 24

Figure S14. Confocal image of living U-2 OS HaloTag-Lamin A/C cells transfected with pCLIP-Cox8a 25

Figure S15. Confocal and STED image of living U-2 OS HaloTag-Lamin A/C cells............... 26

Figure S16. Confocal image of living U-2 OS-Vim-Halo cells transfected with pSNAPf-LaminA-C labeled with **20-SNAP** 27

Figure S17. Confocal image of living U-2 OS-Vim-Halo cells transfected with pdHalo-LaminA-C labelled with **20-Halo**... 28

Figure S18. Annotated vector map of the engineered plasmid Snapf-GianCreg used for expression in mammalian cells. 29

Figure S19. Annotated vector map of the engineered plasmid dHalo-LaminA used for expression in mammalian cells. 30

Supplementary Methods 31
General experimental information and synthesis 31
Thin layer chromatography 31
Preparative flash column chromatography 31
High-Performance Liquid Chromatography (HPLC) and Mass Spectrometry (MS) 31
NMR spectra 32
Melting points 32
Optical spectroscopy 32
Plasmids 33
Transfection 34
Cell culture and labeling 34
Cell viability 34
Confocal and STED microscopy 35
Preparation of 2-hydroxybenzophenone triflates **1** 37
1d 37
1g 37
1j 38
S1 39
1k 40
1m 40
S2 41
1n 42
1p 42
1q 43
1ab 44
S4 45
1ad 45
Preparation of 3-substituted benzothiophene 1,1-dioxides **3** 46
2a 46

3a 47
2'a 48
2b 48
3b 49
3c 50
3d 51
2e 52
3e 53
2f 53
3f 54
2g 55
3g 56
3h 57
2i 58
3i 58
3j 59
2k 60
3k 61
2l 62
3l 63
2m 63
3m 64
2n 65
3n 66
2o 67
3o 68
3p 68
3q 70

3r 71
3s 72
3t 73
3u 74
3v 75
2w 76
3w 77
3x 77
3y 79
3z 80
3aa 81
2ab 82
3ab 83
2ac 84
3ac 85
3ad 86
Preparation of aryl triflates **7**, **9**, **10**, **12** 86
7 87
9 87
10a 88
10b 89
10g 90
10h 91
10j 92
10k 93
12a 94
12b 95
Preparation of 3-unsubstituted benzothiophene 1,1-dioxides **6a**, **14a,b** 95
Method A (from arylimine **9**) 96

5a 96
Method B (via *N*,*N*-dimethylhydrazone **8**) 97
8 97
5a 97
Method C (via *O*-methyloxime **11a**) 98
11a 98
5a 99
6a 99
13a 100
14a 101
13b 101
14b 102
Preparation of 3-aminobenzothiophene 1,1-dioxides **15** 103
15a 103
11b 103
15b 104
11c 105
15c 106
15d 106
11e 107
15e 108
11f 109
15f 109
11g 110
15g 111
11h 111
15h 112
11i 113

15i ... 114
11j ... 114
15j ... 115
11k ... 116
15k ... 117
11l ... 118
15l ... 119
11m ... 119
15m ... 120
15n ... 121
15o ... 121
Preparation of large Stokes shift fluorescent dye **20** ... 122
17 ... 122
18 ... 123
19 ... 123
20 ... 124
Preparation of fluorescent ligands **20-Halo**, **20-HTL2**, **20-SNAP**, **20-CLIP** ... 125
N-Me-HaloTag(O2) ligand **S7** ... 125
S6 ... 125
S7 ... 125
20-Halo ... 126
N-Me-HTL2 ligand **S14** ... 127
S8 ... 127
S9 ... 128
S10 ... 128
S11 ... 129
S12 ... 130
S13 ... 131
S14 ... 132

20-HTL2 133
N-Me-SNAP ligand ($CPCF_3$-NHMe) **S16** 134
S15 134
S16 135
20-SNAP 135
N-Me-CLIP ligand (BC-NHMe) **S18** 136
S17 136
S18 137
20-CLIP 138
Preparation of fluorescent ligands of large Stokes dye *SiX* 139
SiX-Halo 139
SiX-HTL2 140
Supplementary references 142

Supplementary Tables

Compound	λ_{max}^{abs} (nm) [$CHCl_3$/MeOH]	λ_{max}^{em} (nm) [$CHCl_3$/MeOH]	ε ($M^{-1}cm^{-1}$) [$CHCl_3$/MeOH]	Φ_{fl} [$CHCl_3$/MeOH]	τ (ns) [$CHCl_3$/MeOH]
**3f**	376/370	466/474	20800/24300	0.46/0.14	2.98/1.18
**3o**	363/357	486/497	8550/8270	0.10/0.015	0.95/0.29
**3p**	388/383	499/506	8990/7880	0.019/0.006	0.27/0.11
**3q**	356/351	463/472	11100/10900	0.88/0.77	6.09/6.78
**3v**	360/354	477/491	8980/9750	0.25/0.14	2.37/1.62
**3w**	363/357	479/496	9770/8350	0.49/0.25	4.55/2.90
**6a**	338/336	419/422	9280/10300	0.92/0.81	5.76/6.59
**14a**	438/441	535/568	13600/16900	0.28/0.34	2.42/3.30
**14b**	468/472	557/596	17100/18100	0.61/0.51	5.01/5.00
**20**	427[a]/430	575[a]/582	8400[a]/14500	0.02[a]/0.03	0.53 (96.6%), 2.75 (3.4%)[a] / / 0.40 (92.3%), 1.01 (7.7%)
**20-Halo**	430[a]/436	571[a]/577	13200[a]/13300	n.d.	0.34 (89.0%), 2.67 (11.0%)[a] / / 0.37 (88.4%), 1.94 (11.6%)

[a] in acetonitrile; b biexponential fluorescence decay, detected at λ_{max}^{em} and presented as τ_1 (I_1, %), τ_2 (I_2, %), where I_1, I_2 are fractional intensities of the individual lifetime components τ_1, τ_2.

Table S1. Photophysical properties of compounds **3** and **14** (demonstrating fluorescence in solution); see Supplementary Methods for the experimental information.

Figure	excitation (wavelength [nm]/ power [µW])[a]	STED[b] (wavelength [nm]/ power [mW])[a]	detection window [nm]	pixel size [nm]	dwell time [µs]	line accumulation
**4, A-D, H-I**	405/12<br>485/20<br>561/12<br>640/3	---	415-583<br>516-728<br>571-630<br>650/757	70	10<br>10<br>20<br>20	1<br>2<br>2<br>2
**4, E-G, J**	485/27<br>561/6.4<br>640/19	---	516-728<br>571-630<br>650/757	70	10<br>10<br>10	2<br>2<br>4
**5, A-D**	485/34<br>561/12<br>640/3	---	516-728<br>571-630<br>650/757	70	10<br>10<br>10	2<br>2<br>1
**5, E**	485/44<br>561/21<br>640/4	775/89<br>775/178<br>775/89	516-728<br>571-630<br>650-757	20	10<br>10<br>10	6<br>3<br>3
**5, F-I**	405/24<br>485/34<br>561/24<br>640/3	---	415-583<br>516-728<br>571-630<br>650-757	70	10<br>20<br>10<br>10	1<br>3<br>3<br>2
**5, J**	485/44<br>561/24<br>640/5	775/250<br>775/134<br>775/178	516-728<br>571-630<br>650-757	30	20<br>10<br>10	3<br>6<br>6
**S11, A-D**	405/12<br>485/44<br>561/12<br>640/6	---	415-583<br>516-728<br>571-630<br>650-757	70	10<br>10<br>10<br>10	1<br>2<br>2<br>2
**S11, E-H**	405/12<br>485/44<br>561/12<br>640/2	---	415-583<br>516-728<br>571-630<br>650-757	70	10<br>10<br>10<br>10	1<br>2<br>2<br>2
**S12**	405/24<br>485/34<br>640/6	---	415-583<br>516-728<br>650-757	70	10<br>10<br>10	1<br>2<br>2
**S13, A-C**	405/24<br>485/34<br>640/6	---	415-583<br>516-728<br>650-757	70	10<br>20<br>10	1<br>3<br>2

(continued)

Figure	excitation (wavelength [nm]/ power [µW])	STED (wavelength [nm]/ power [mW])	detection window [nm]	pixel size [nm]	dwell time [µs]	line accumulation
**S13, D**	485/44<br>640/7	775/178<br>775/89	516-728<br>650-757	30	20<br>10	3<br>6
**S14**	485/12<br>640/6	---	516-728<br>650-757	70	20<br>10	2<br>2
**S15, A-C**	405/24<br>485/32<br>561/12	---	415-583<br>516-728<br>571-630	70	10<br>20<br>10	1<br>3<br>2
**S15, D**	485/64<br>561/16	775/267<br>775/89	516-728<br>571-723	30	20<br>10	3<br>6
**S15, E-G**	405/24<br>485/32<br>640/3	---	415-583<br>516-728<br>650-757	70	20<br>20<br>10	1<br>3<br>2
**S15, H**	485/64<br>640/5	775/178<br>775/178	516-728<br>650-757	30	20<br>10	12<br>6
**S16, A-D**	485/32<br>561/6<br>640/19	---	516-728<br>571-630<br>650-757	70	10<br>10<br>10	2<br>2<br>4
**S16, E-H**	485/32<br>561/30<br>640/3	---	516/728<br>571-630<br>650-757	70	20<br>10<br>10	3<br>3<br>2
**S17, A-D**	485/32<br>561/6<br>640/3	---	516-728<br>571-630<br>650-757	70	20<br>10<br>10	4<br>2<br>2
**S17, E-G**	485/32<br>640/3	---	516-728<br>650-757	70	20<br>10	3<br>2
**S17, H**	485/55<br>640/5	775/178<br>775/178	516-728<br>650-757	30	20<br>10	3<br>6

Table S2. Imaging parameters for confocal and STED images. All powers were measured at the back focal plane of the objective lens. Channels were measured sequentially, adjusting the focus for each corresponding structure.

Supplementary Figures

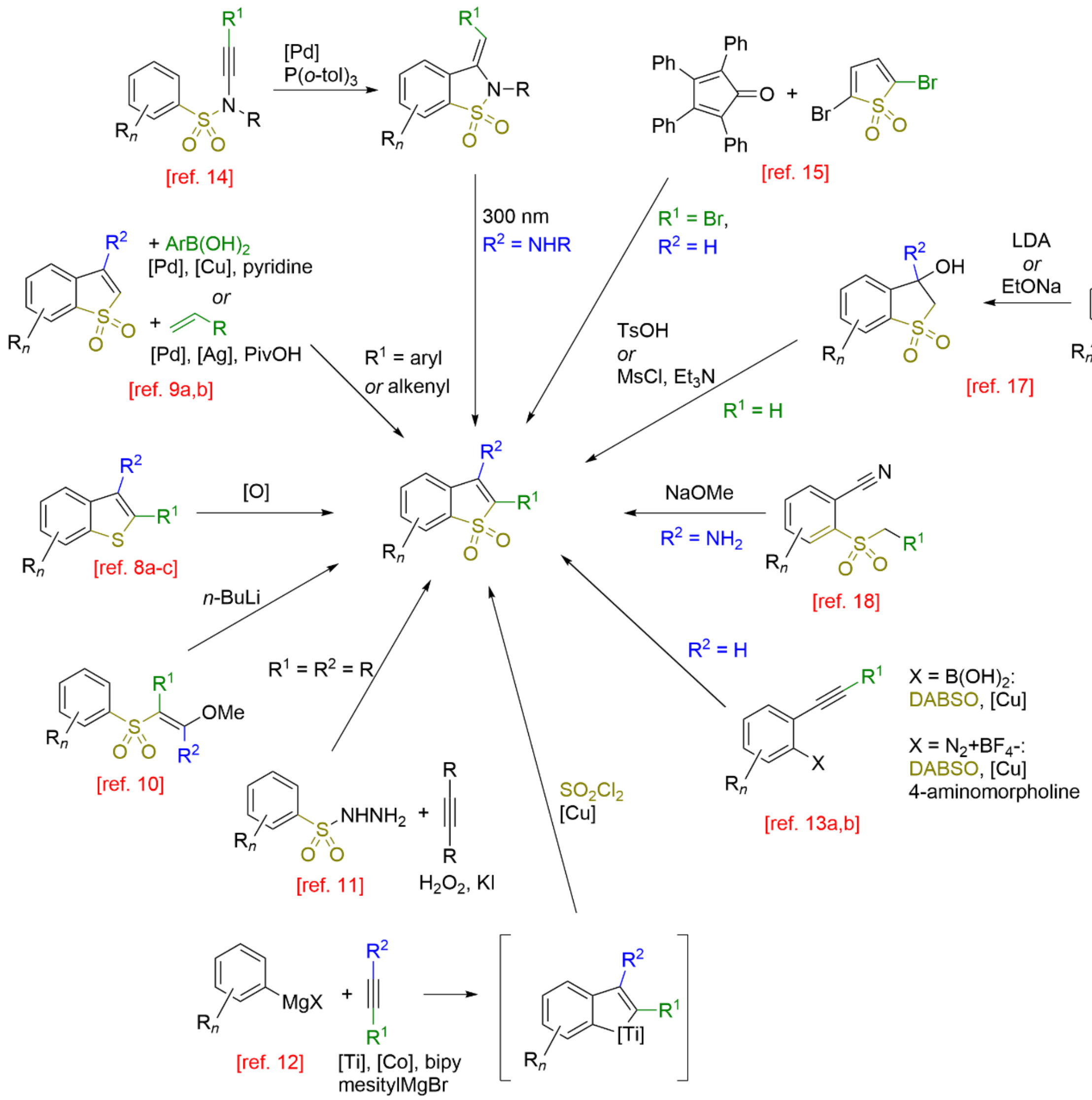

Figure S1. Synthetic approaches to benzo[*b*]thiophene 1,1-dioxides (see main text for the references).

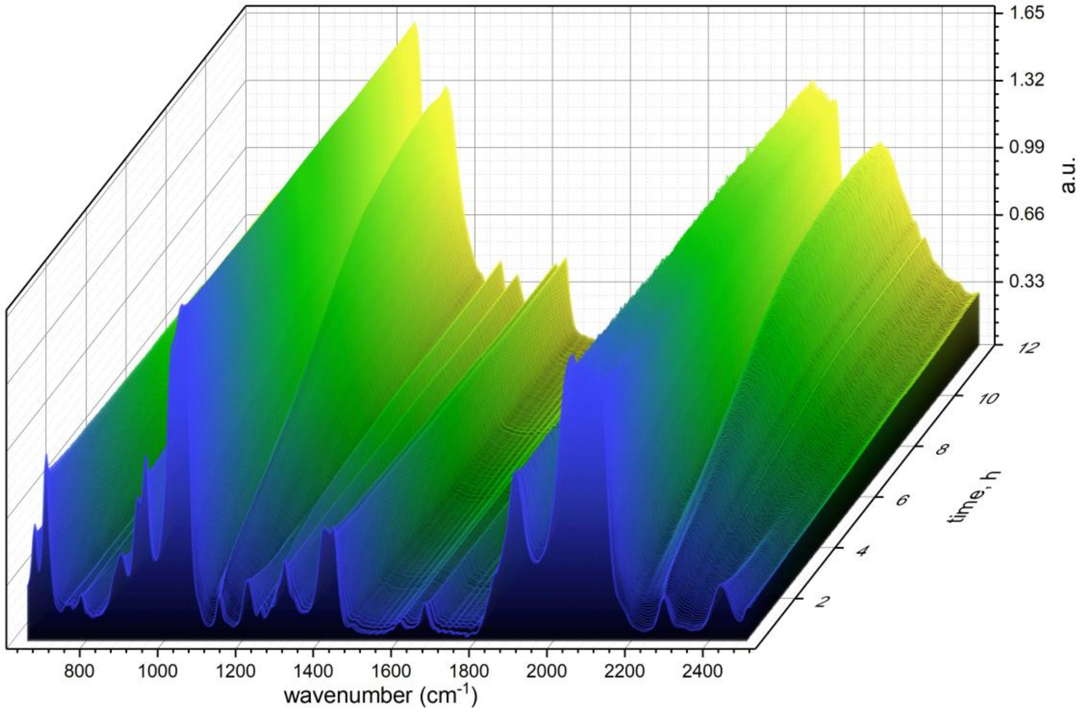

a

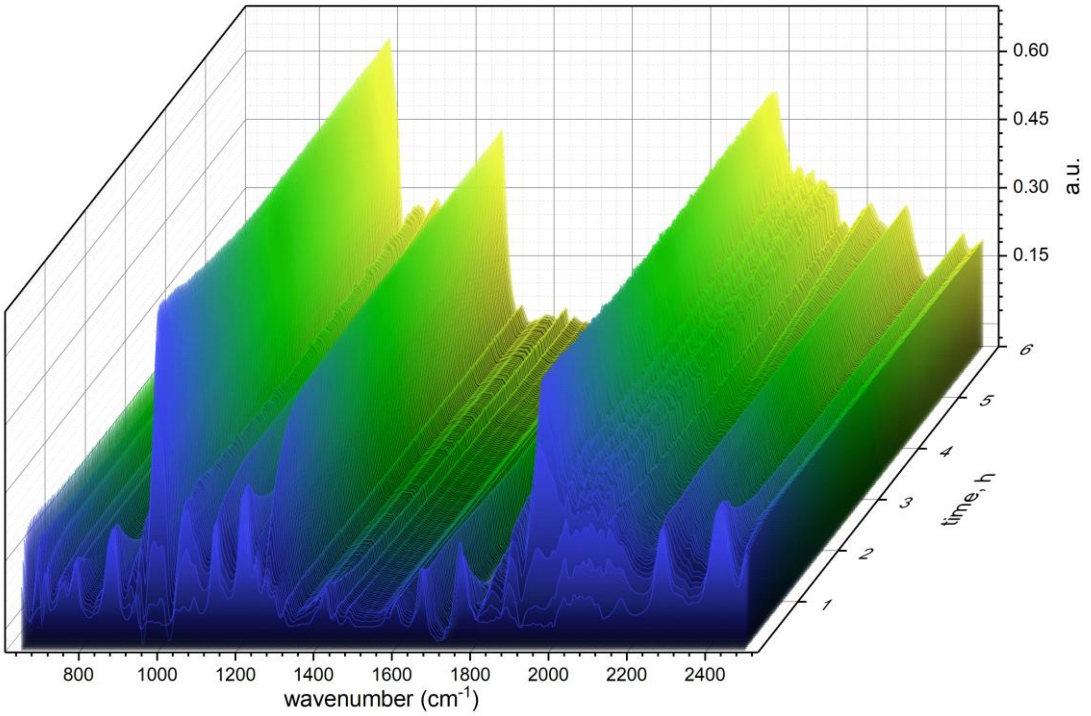

b

(continued)

c

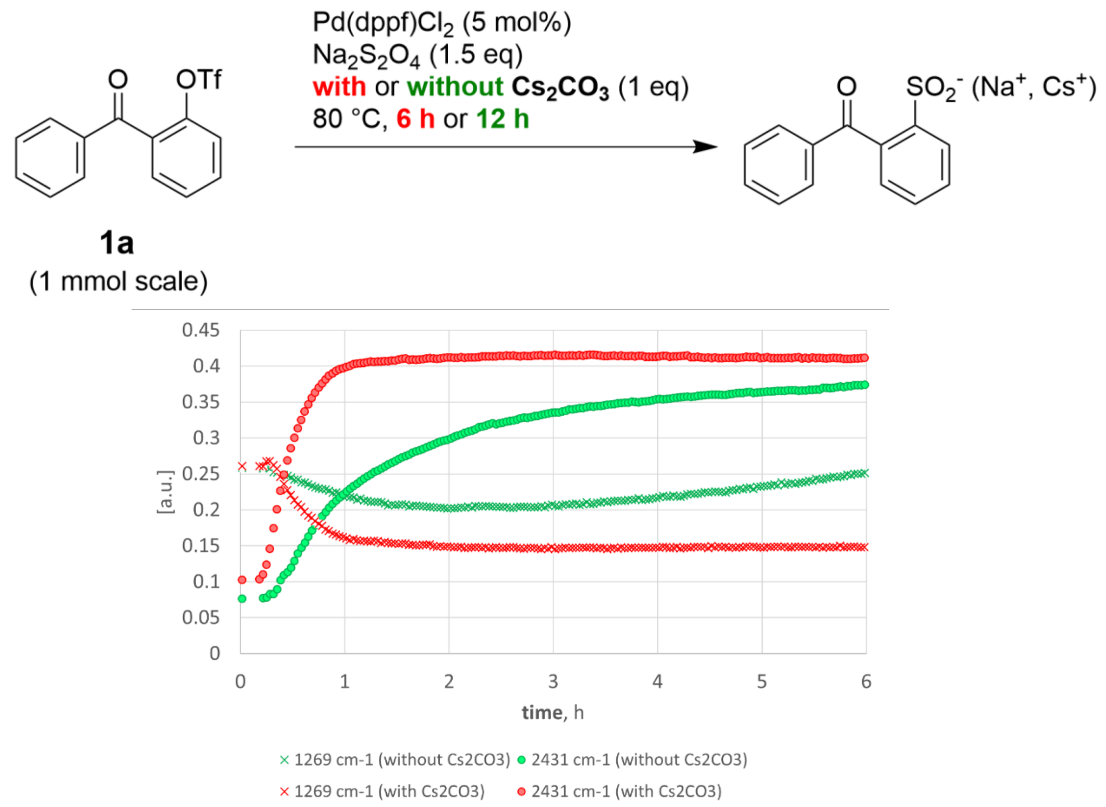

d

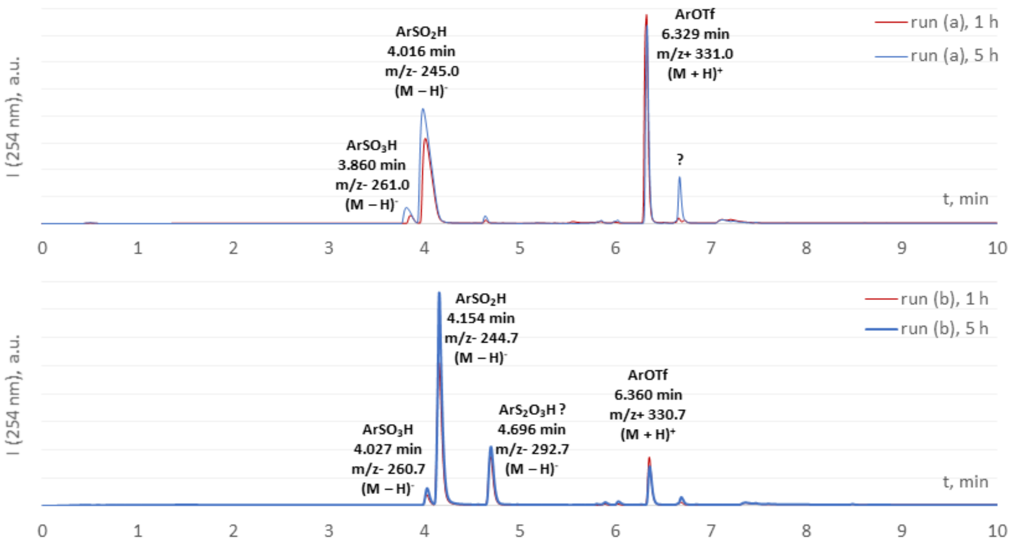

Figure S2. *In situ* IR spectroscopy of Pd-catalyzed sulfinylation of **1a** in the absence of (**a**) *vs* in the presence of Cs_2CO_3 base (1 eq) (**b**) at 80 °C. Isolated traces at 1269 cm^{-1} and 2431 cm^{-1} (corresponding to the product and the starting material, respectively) are shown in (**c**); time limited to 6 h due to background drift. (d) HPLC-MS traces of the reaction mixture from the runs (**a**) and (**b**) at 1 h and 5 h showing conversion of **1a** to aryl sulfinic acid and other identified and unidentified byproducts.

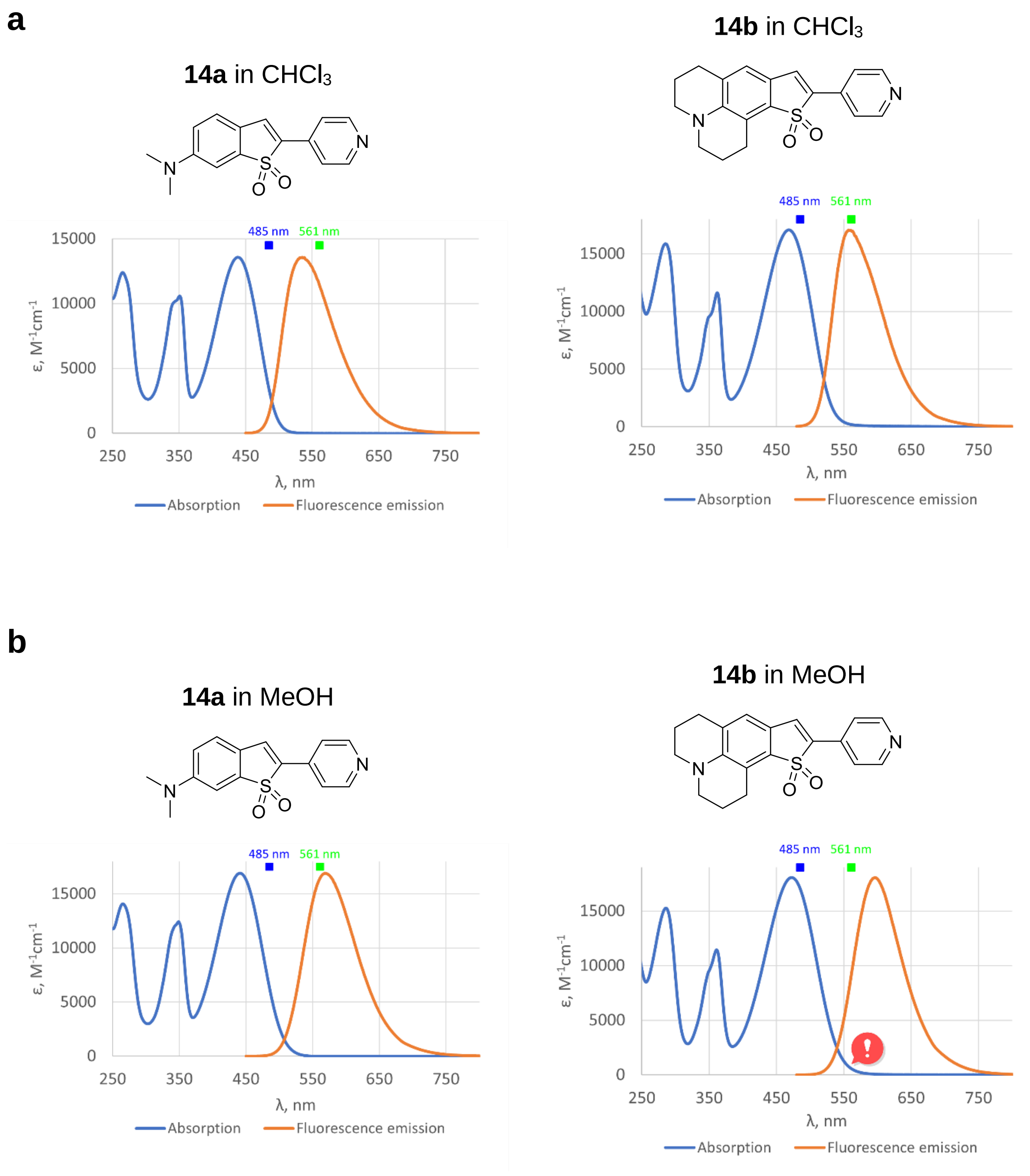

Figure S3. Absorption and fluorescence emission spectra of compounds **14a** and **14b** in chloroform (a) and methanol (b). Unlike **14a**, **14b** shows non-negligible absorption cross-section at 561 nm (common green excitation laser wavelength of a fluorescence microscope) in protic solvents.

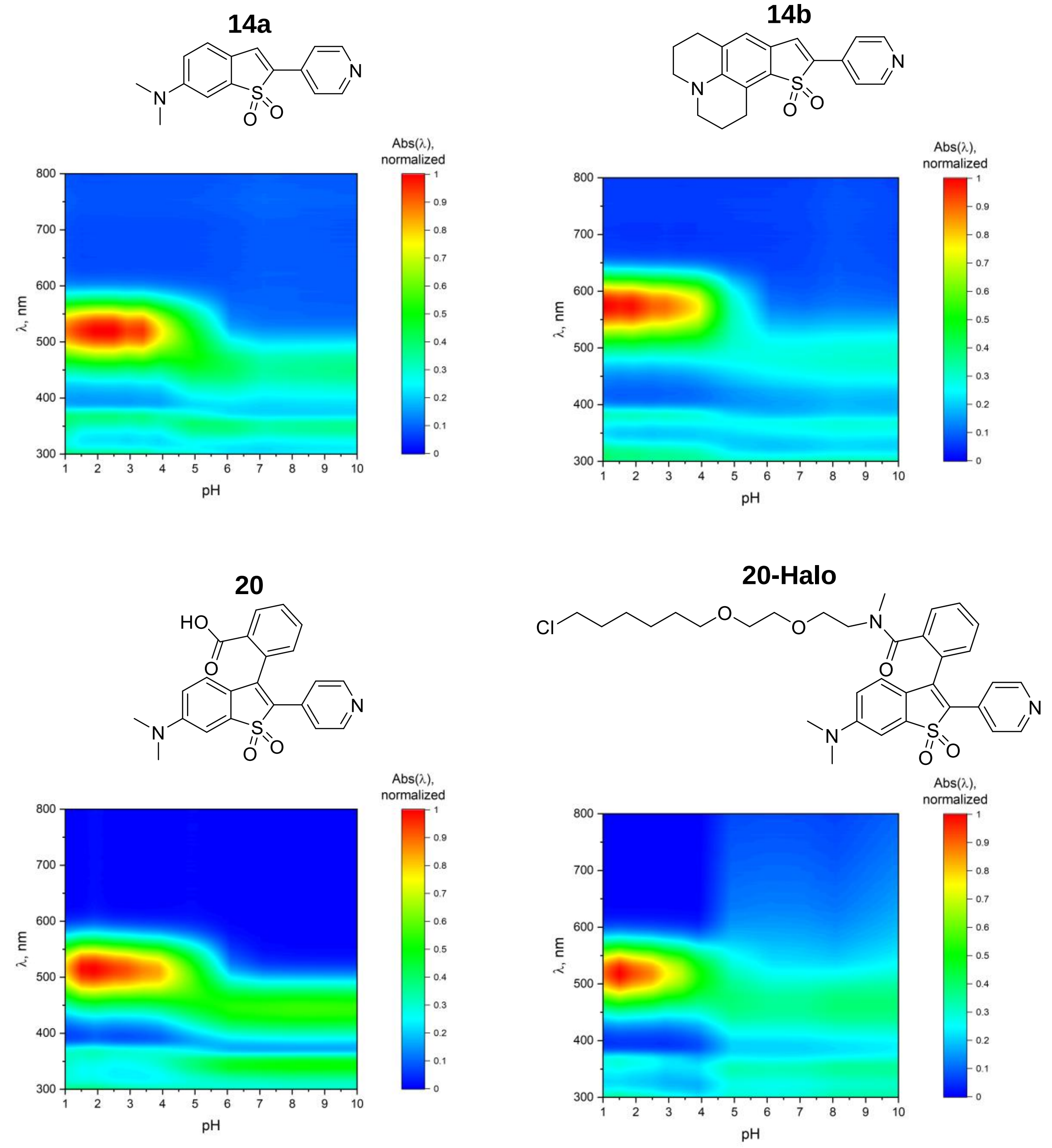

Figure S4. Normalized absorption spectra of compounds **14a**, **14b**, **20** and **20-Halo** in 100 mM phosphate buffer at different pH values (pH 1-10, +1% (v/v) DMSO).

a

20 in MeCN

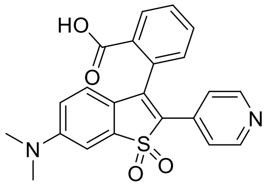

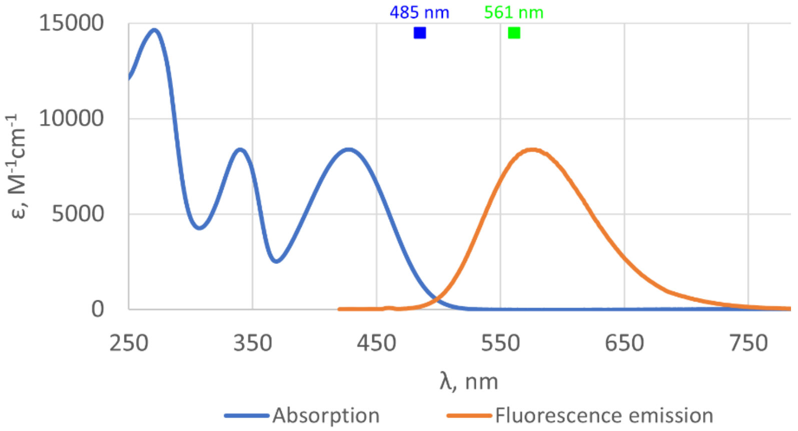

b

20 in MeOH

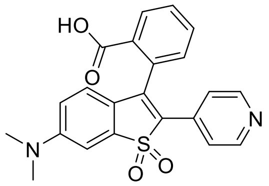

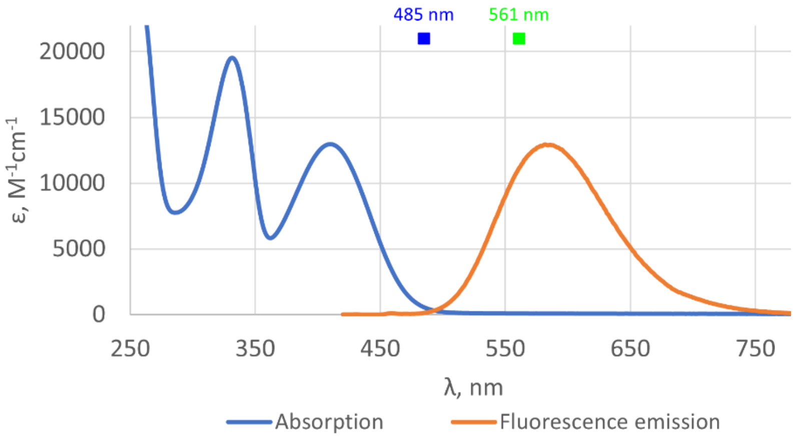

Figure S5. Absorption and fluorescence emission spectra of compound **20** in acetonitrile (a) and methanol (b).

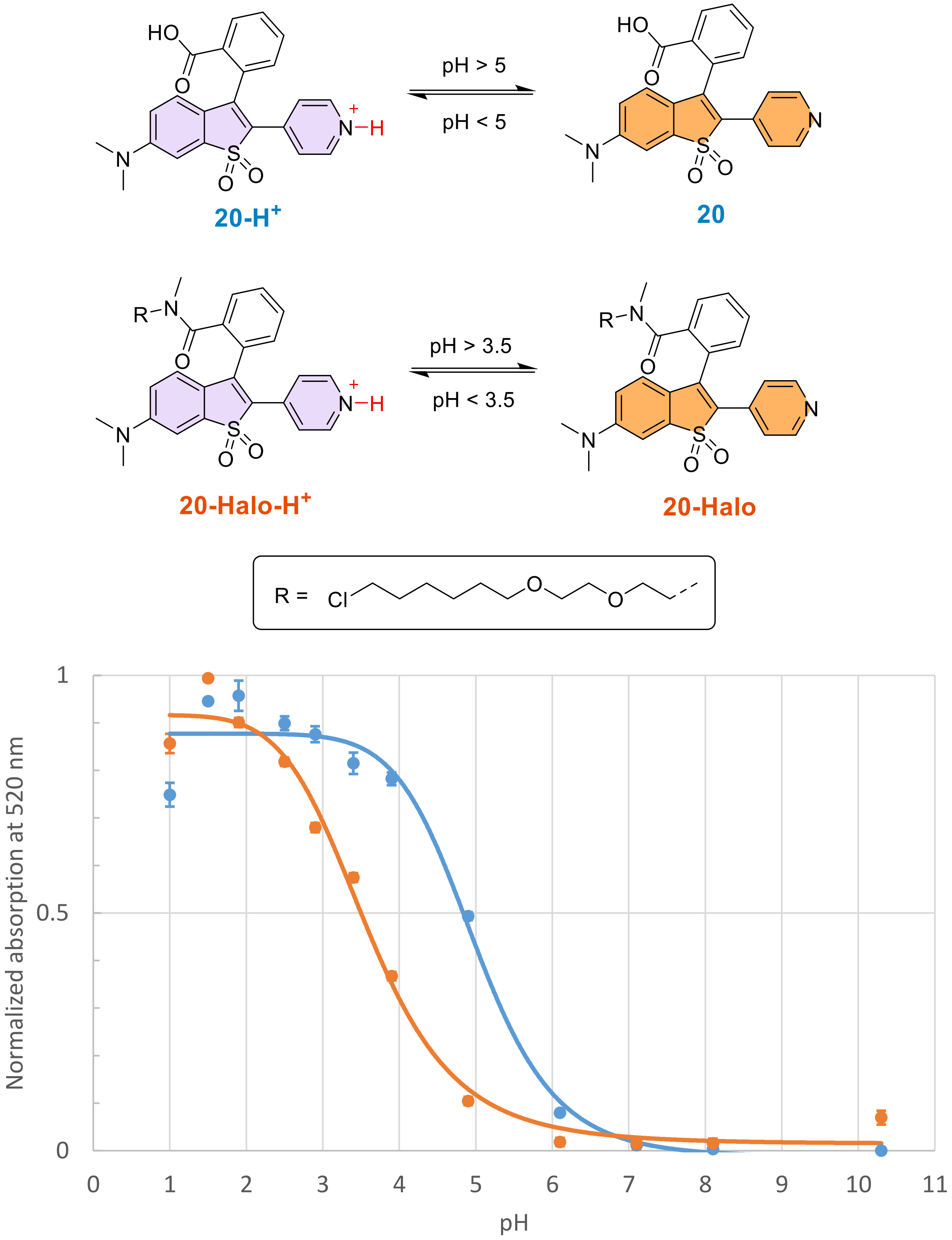

Figure S6. pH-dependent equilibria of the large Stokes dye **20** and its tertiary amide ligands (exemplified by **20-Halo**). Absorption in 100 mM phosphate buffer at 520 nm (averaged in triplicate) was plotted against pH, normalized to the maximum and minimum values between pH 1-10 and fitted to the Hill equation using Origin 2024.

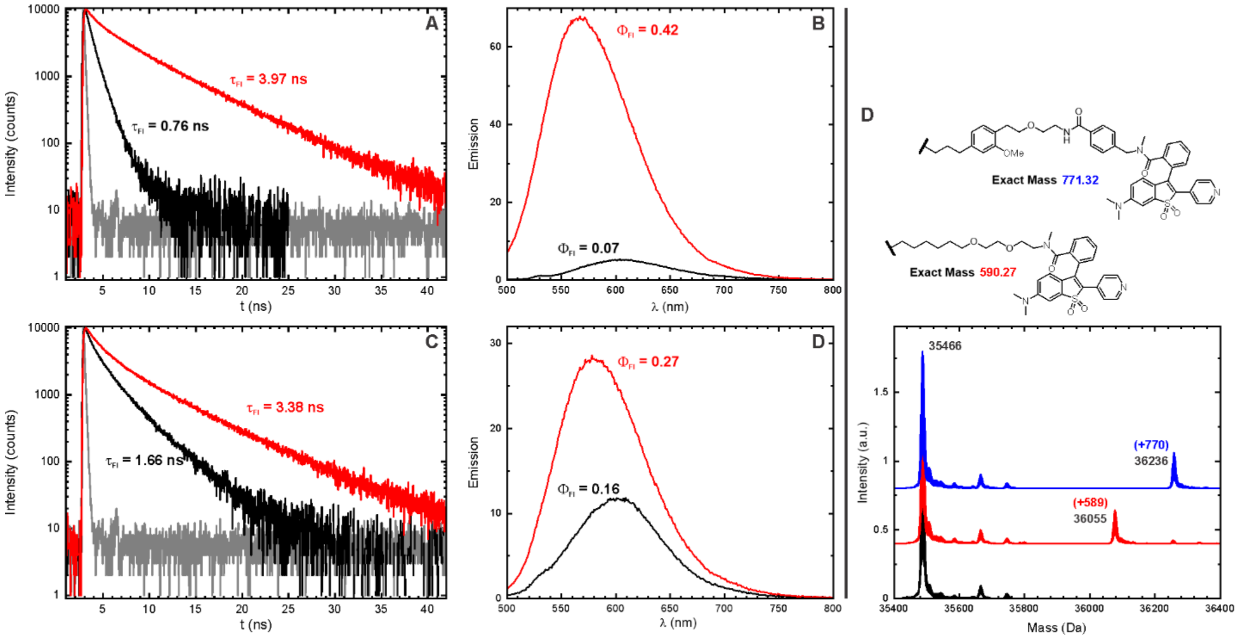

Figure S7. Fluorescence enhancement of **20-Halo** and **20-HTL2** (500 nM) upon binding to excess HaloTag7 protein in PBS (pH 7.4). Fluorescence lifetimes of **20-Halo** (A) and **20-HTL2** (C), and emission signal under 450 nm excitation of **20-Halo** (B) and **20-HTL2** (D) for the unbound label in PBS (black lines) and after the addition of excess Halotag7 protein (red lines). (D) ESI-MS spectra of solution after equilibration with HaloTag7 (red line: **20-Halo**; blue line: **20-HTL2**). The spectrum of the unlabeled protein is also shown (black line), along with the ligand structures covalently bound to the protein's active site.

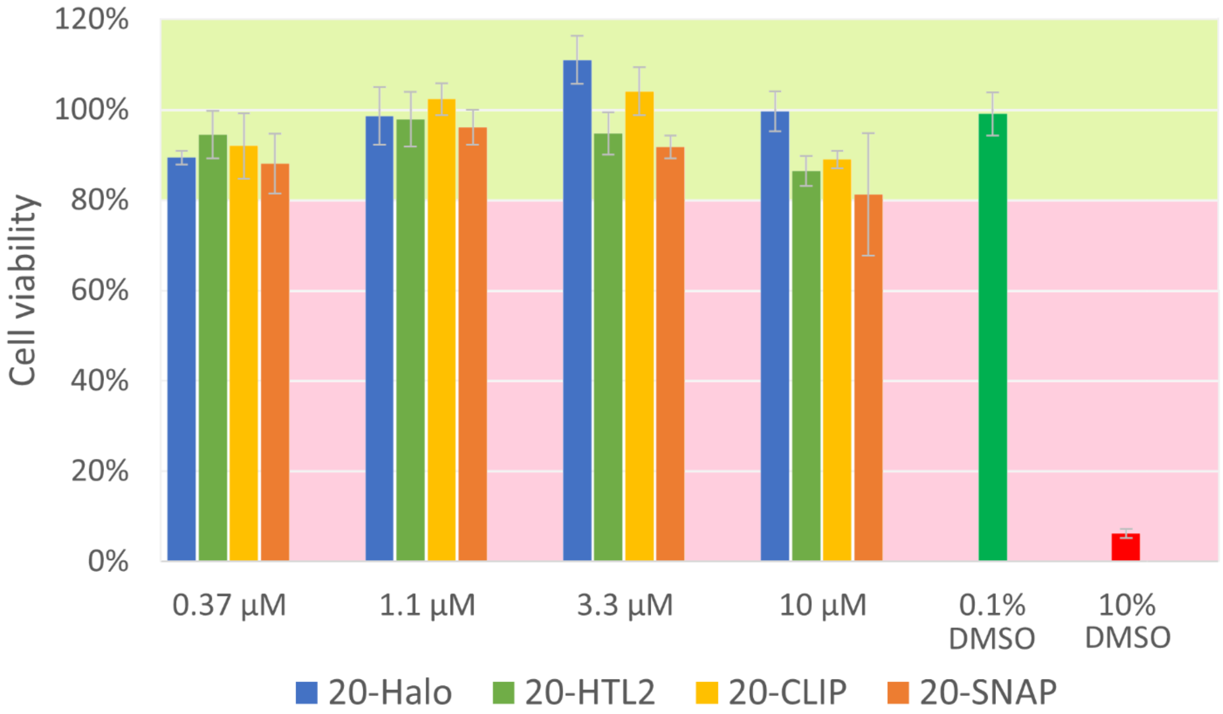

Figure S8. Influence of probes **20-Halo**, **20-HTL2**, **20-CLIP** and **20-SNAP** on viability of U-2 OS cells determined in a CellTiter-Glo 2.0 assay (see Cell viability section in Supplementary Methods for details). Error bars indicate standard deviation (*N* = 12).

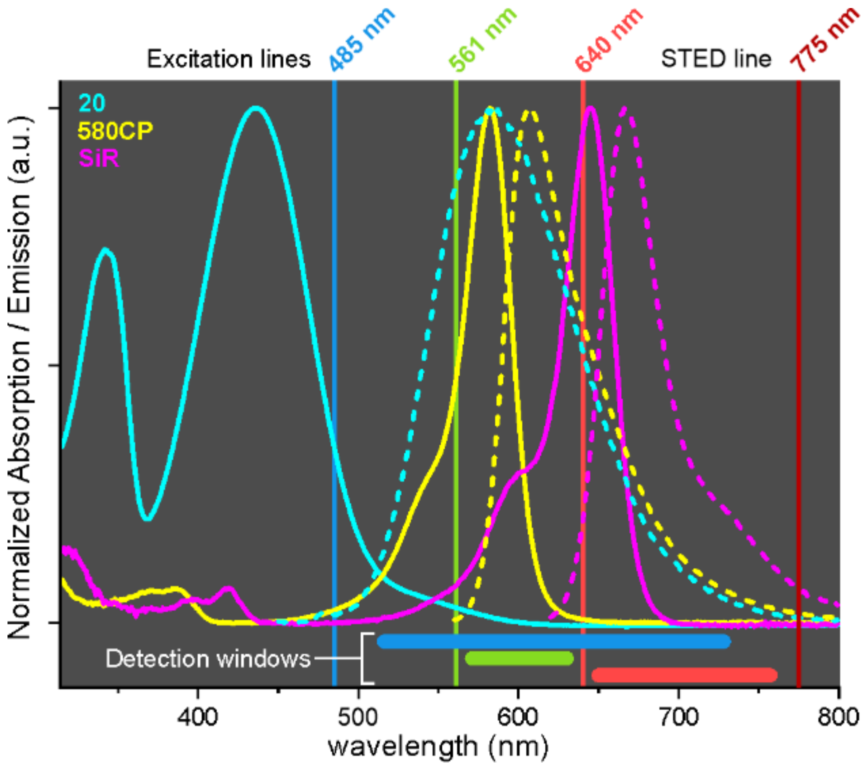

Figure S9. General multichannel imaging scheme. For images with one or two color channels, the additional channels were omitted (likewise, for confocal images the STED line was not used). Spectra for three representative dyes (**20**, *580CP* and *SiR*): absorption – solid line, fluorescence emission – dashed line. Laser lines (excitation and STED) and detection channels are color coded for blue, green and red channels. The color of the detection channel corresponds with the excitation laser line used, single STED line is used for all three channels. Further details for each channel (e.g. excitation intensities, integration times, pixel sizes) are specified in Table S2 for each recorded image.

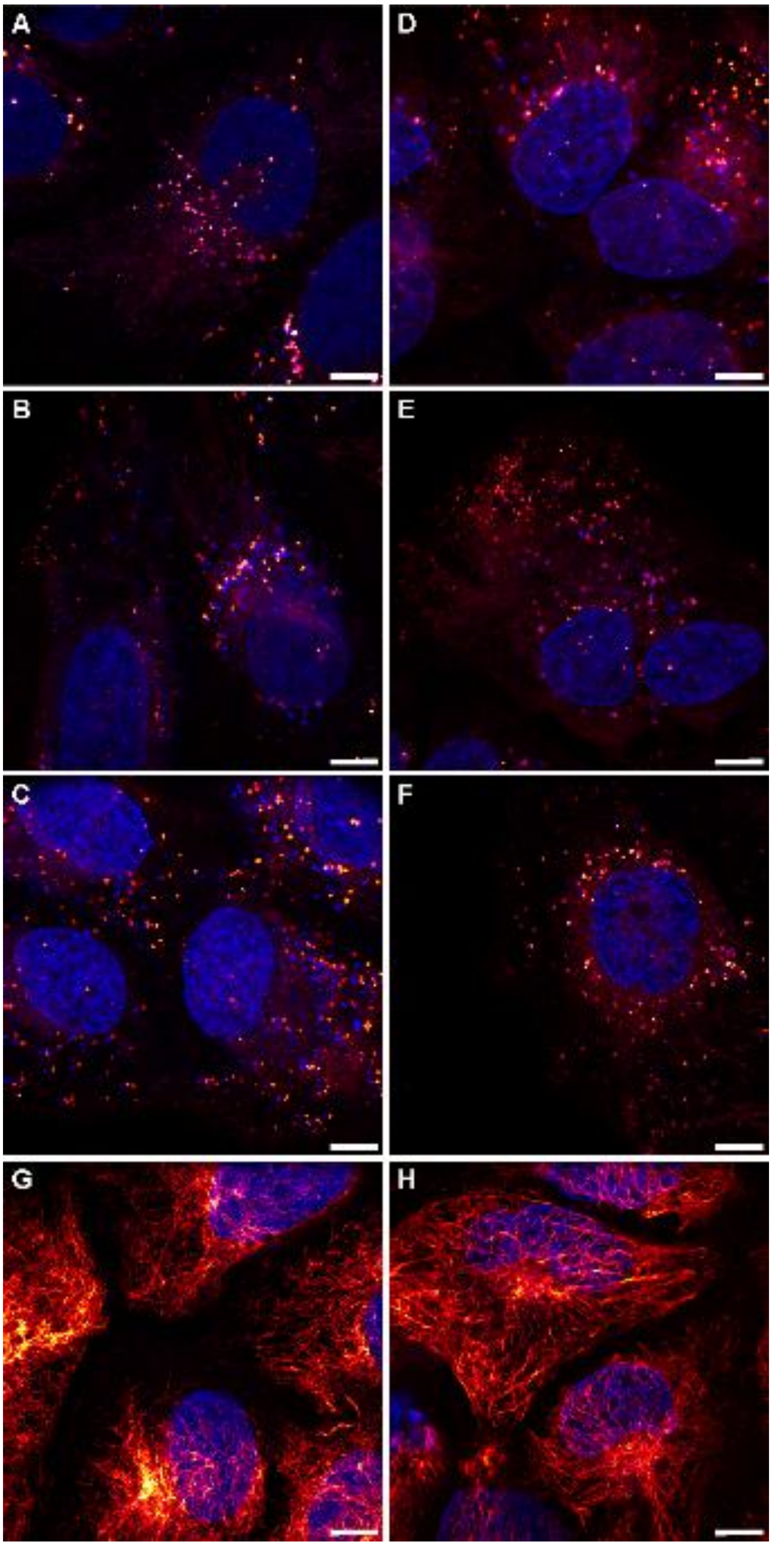

Figure S10. Confocal image of living U-2 OS-Vim-Halo cells labeled (A-C) with *SiX-Halo* (1 μM, 16 h) or (D-F) with *SiX-HTL2* (1 μM, 16 h), washed with dye-free media (20 min) and counterstained with Hoechst 33342 (8 μM, 10 min). For control, the cells were also treated with (G) **20-Halo** or (H) **20-HTL2** under the same conditions. Scale bars: 10 μm.

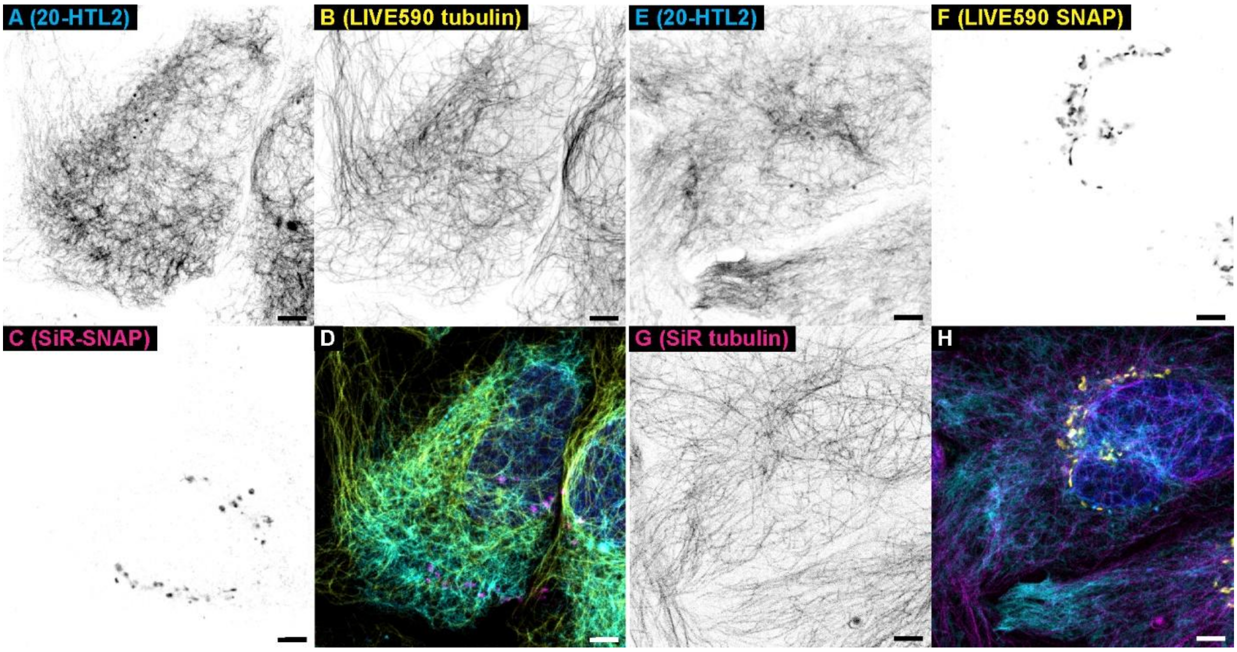

Figure S11. (A-D) Confocal image of living U-2 OS-Vim-Halo cells transfected with pSNAPf-GianCreg, labelled with **20-HTL2** (1 µM) and SiR-SNAP (500 nM) over 2 h, washed with dye-free media (20 min), counterstained with *abberior LIVE590 tubulin* (500 nM, 1 h) and Hoechst 33342 (8 µM, 10 min) and imaged without washing. (E-H) Confocal image of living U-2 OS-Vim-Halo cells transfected with pSNAPf-GianCreg, labelled with **20-HTL2** (1 µM) and *abberior LIVE590 SNAP* (500 nM) over 2 h, washed with dye-free media (20 min), counterstained with SiR-tubulin (500 nM, 1 h) and Hoechst 33342 (8 µM, 10 min) and imaged without washing. Scale bars: 5 µm.

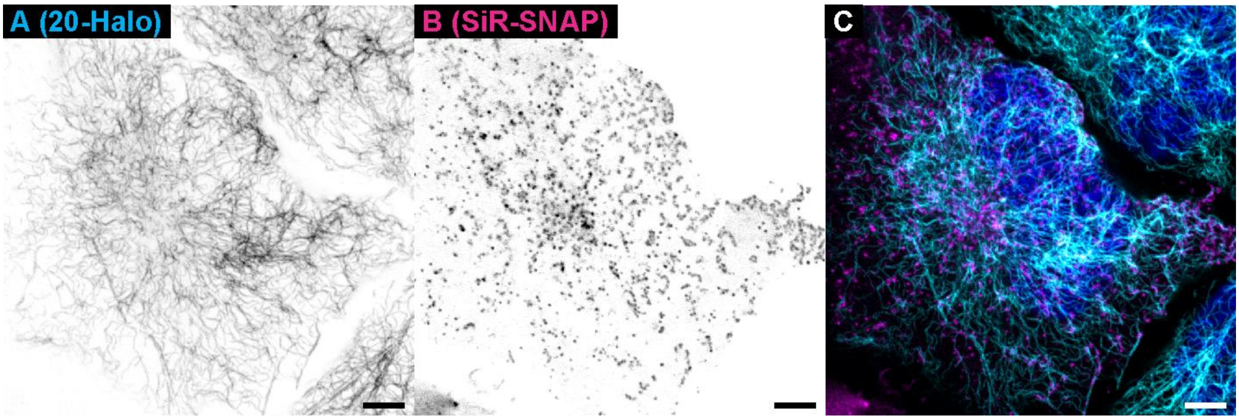

Figure S12. Confocal image of living U-2 OS-Vim-Halo cells transfected with pSNAPf-Clathrin, labelled with **20-Halo** (2 µM, 16 h) and SiR-SNAP (1 µM, 1 h), washed with dye-free media (20 min), counterstained with Hoechst 33342 (8 µM, 10 min) and imaged without washing. Scale bars: 5 µm.

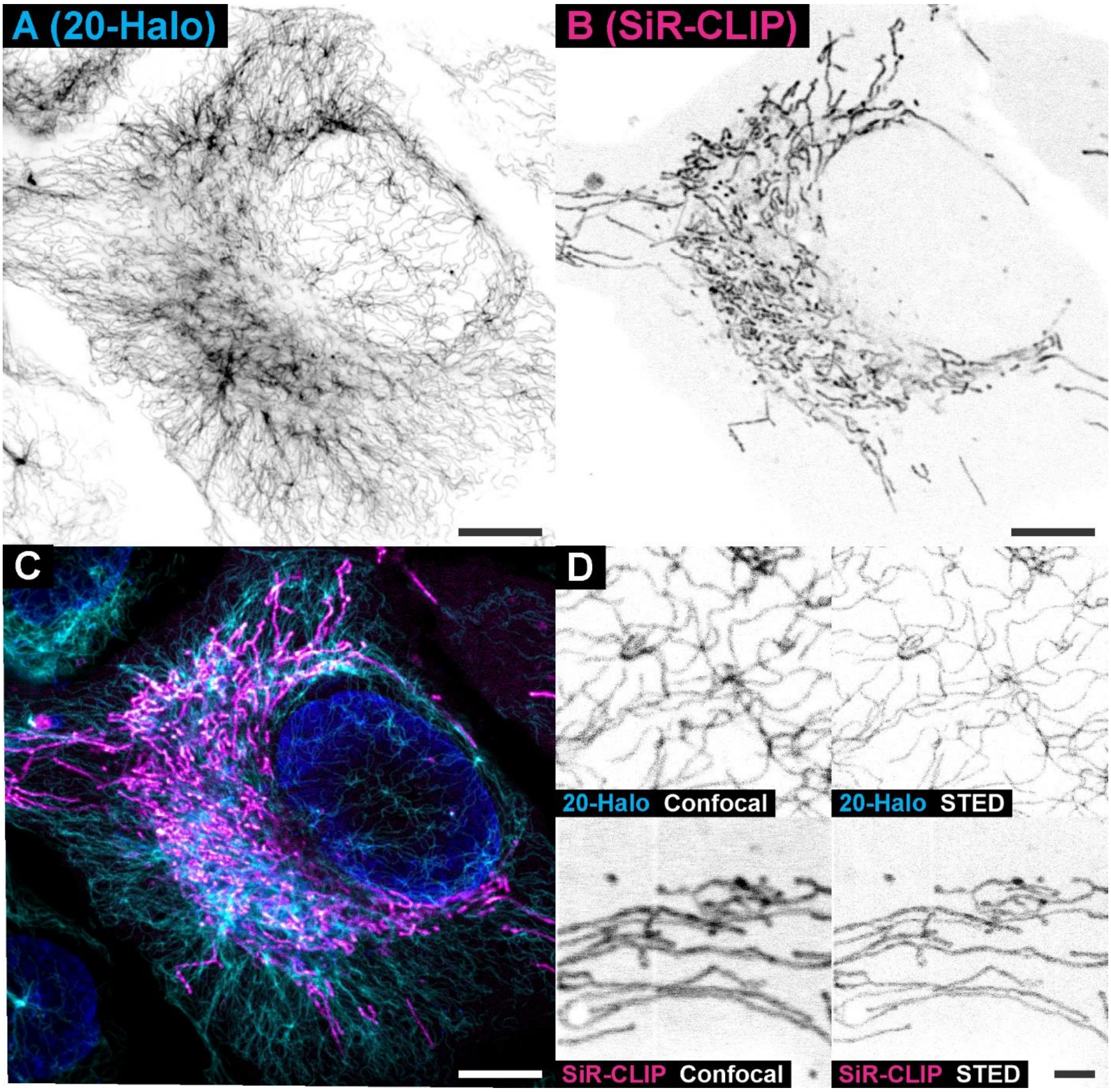

Figure S13. Confocal (A-C) and STED (D) image of living U-2 OS-Vim-Halo cells transfected with pCLIP-Cox8a, labelled with **20-Halo** (2 µM, 16 h) and SiR-CLIP (1 µM, 3 h), washed with dye-free media (20 min), counterstained with Hoechst 33342 (8 µM, 10 min) and imaged without washing. Scale bars: 10 µm (A-C), 2 µm (J).

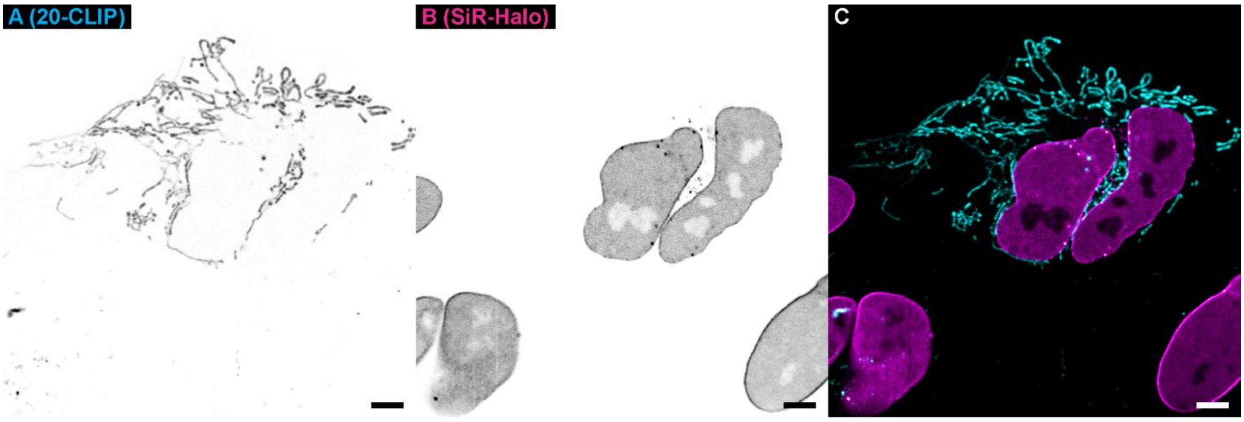

Figure S14. Confocal image of living U-2 OS HaloTag-Lamin A/C cells transfected with pCLIP-Cox8a, labelled with **20-CLIP** (1 µM, 3 h) and SiR-Halo (1 µM, 1 h) and washed with dye-free media (20 min). Scale bars: 5 µm.

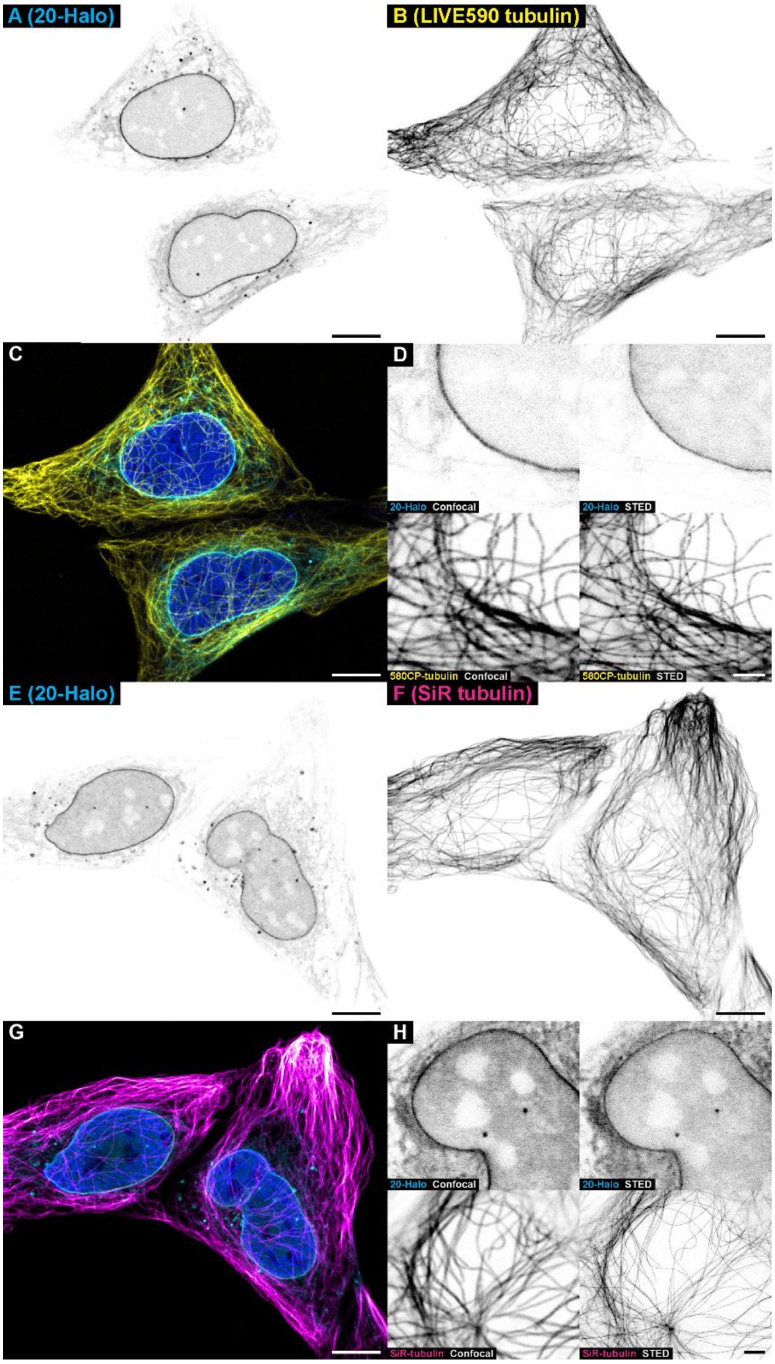

Figure S15. (A-D) Confocal (A-C) and STED (D) image of living U-2 OS HaloTag-Lamin A/C cells, labelled with **20-Halo** (1 µM, 16 h), washed with dye-free media (20 min), counterstained with *abberior LIVE590 tubulin* (500 nM, 1 h, together with 10 µM verapamil) and Hoechst 33342 (8 µM, 10 min) and imaged without washing. (E-H) Confocal (E-G) and STED (H) image of living U-2 OS HaloTag-Lamin A/C cells, labelled with **20-Halo** (1 µM, 16 h), washed with dye-free media (20 min), counterstained with SiR-tubulin (500 nM, 1 h, together with 10 µM verapamil) and Hoechst 33342 (8 µM, 10 min) and imaged without washing. Scale bars: 10 µm (A-C, E-G), 2 µm (D, H).

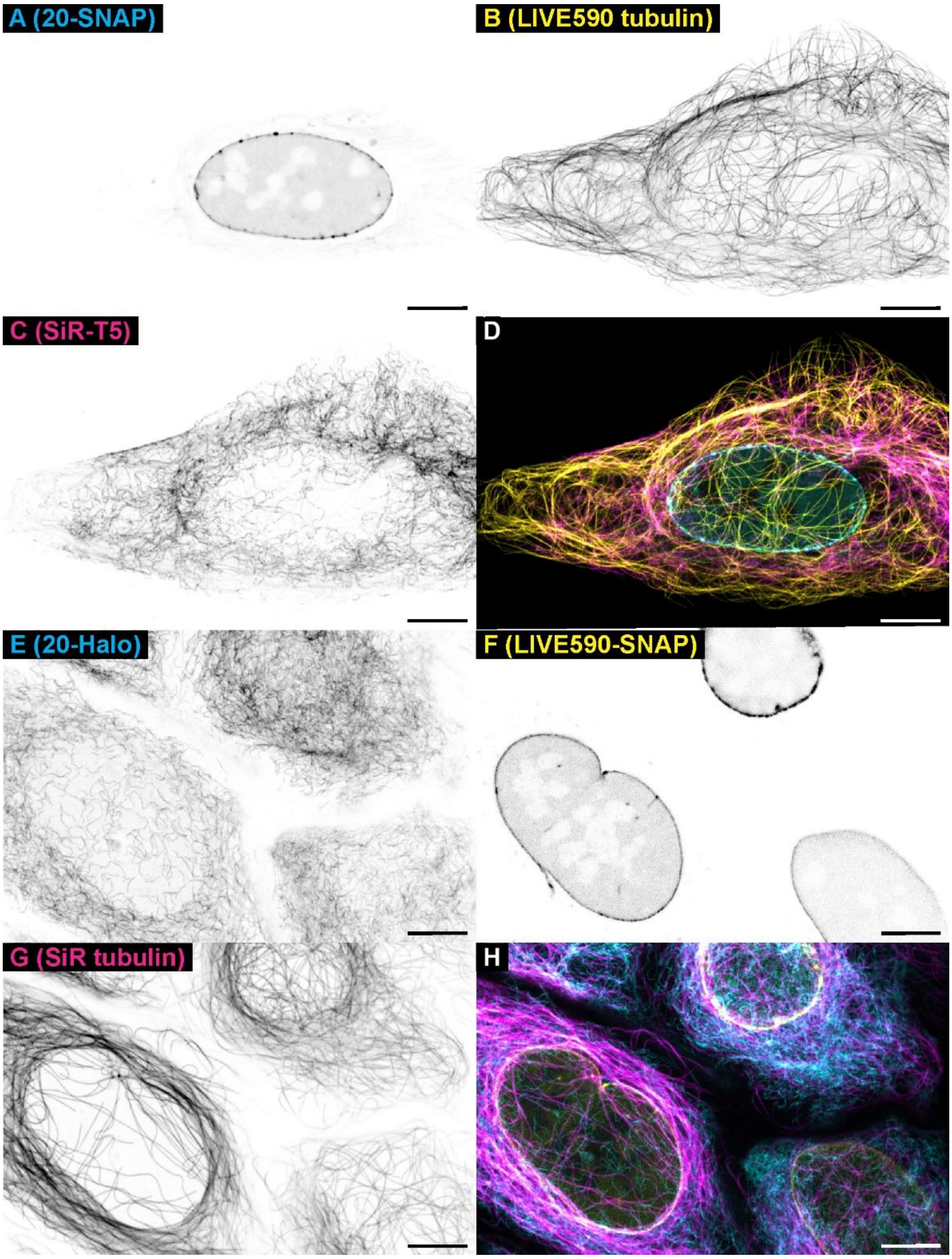

Figure S16. (A-C) Confocal image of living U-2 OS-Vim-Halo cells transfected with pSNAPf-LaminA-C, labelled with **20-SNAP** (1 µM, 16 h), washed with dye-free media (20 min), counterstained with *abberior LIVE590 tubulin* (500 nM, 1 h) and SiR-T5 (500 nM, 1 h) and imaged without washing. (E-H) Confocal image of living U-2 OS-Vim-Halo cells transfected with pSNAPf-LaminA-C, labelled with **20-Halo** (1 µM) and *abberior LIVE590 SNAP* (200 nM) over 16 h, washed with dye-free media (20 min), counterstained with SiR-tubulin (500 nM, 1 h, together with 10 µM verapamil) and imaged without washing. Scale bars: 10 µm.

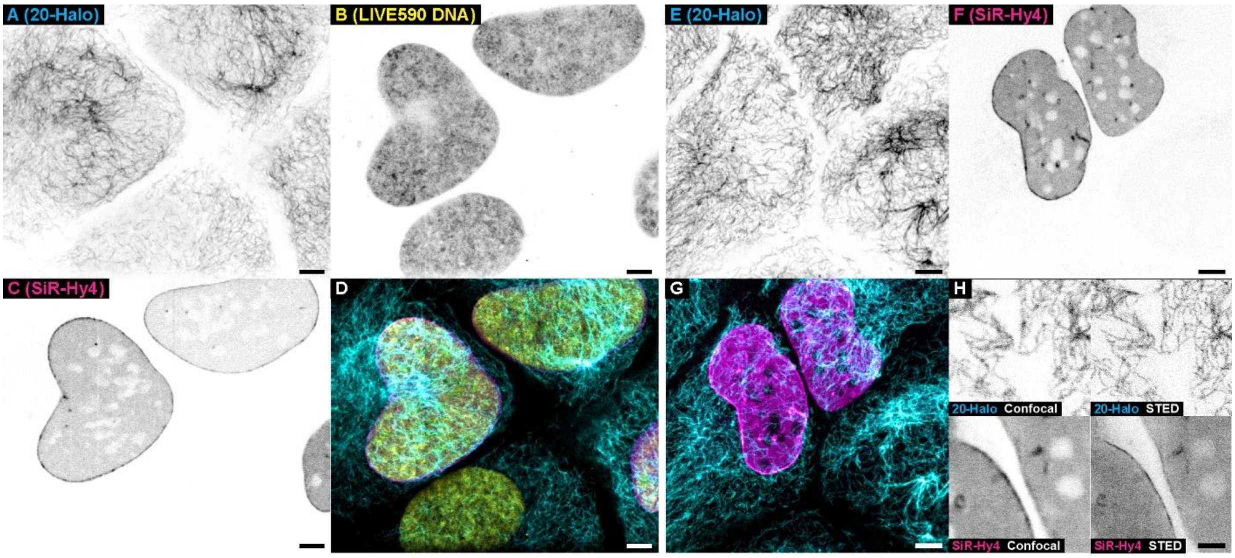

Figure S17. (A-D) Confocal image of living U-2 OS-Vim-Halo cells transfected with pdHalo-LaminA-C, labelled with **20-Halo** (1 µM, 3 h), washed with dye-free media (20 min), counterstained with *abberior LIVE590 DNA* (500 nM) and SiR-Hy4 (2 µM) over 1 h and imaged without washing. (E-H) Confocal (E-G) and STED (H) image of living U-2 OS-Vim-Halo cells transfected with pdHalo-LaminA-C, labelled with **20-Halo** (1 µM, 3 h), washed with dye-free media (20 min), counterstained with SiR-Hy4 (2 µM, 1 h) and imaged without washing. Scale bars: 5 µm (A-G), 2 µm (H).

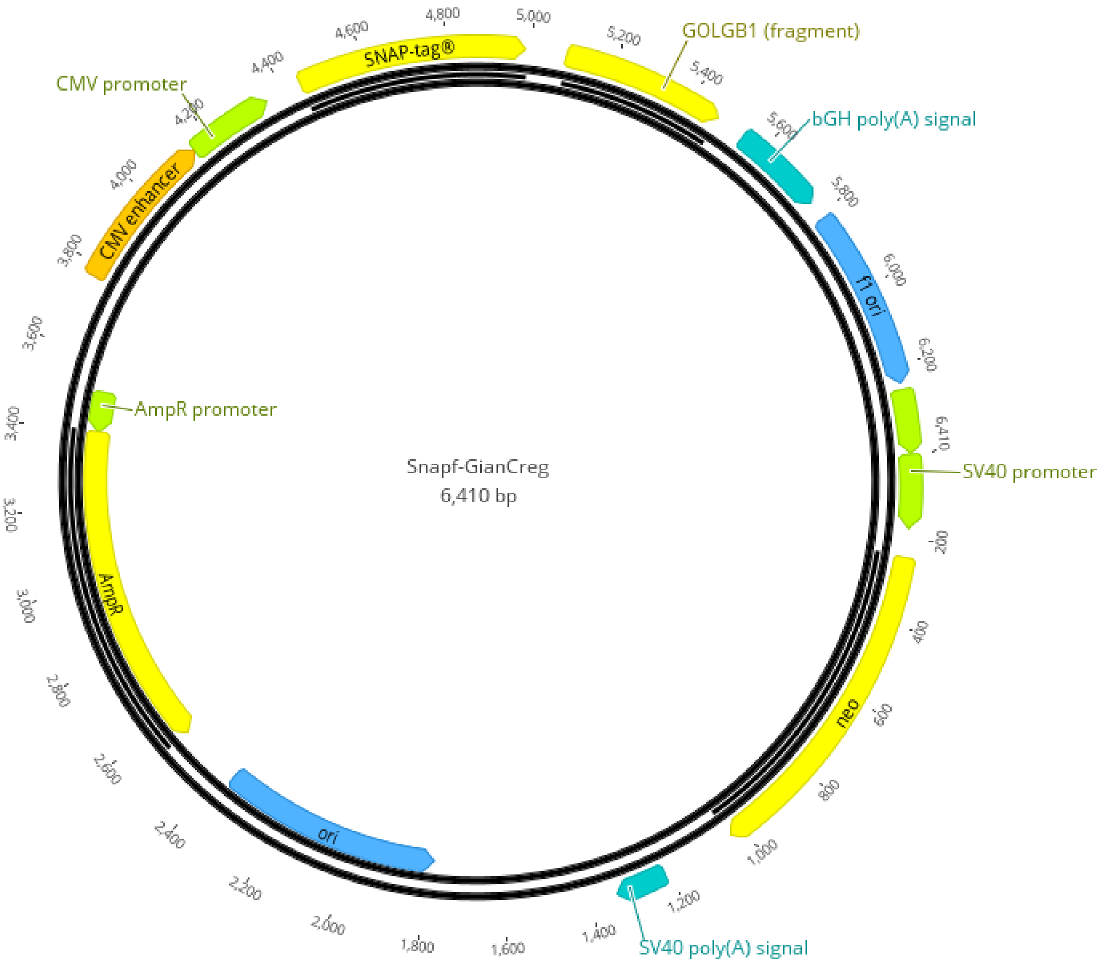

Figure S18. Annotated vector map of the engineered plasmid Snapf-GianCreg used for expression in mammalian cells. The plasmid is based on pcDNA3 backbone and was engineered to express N-terminal Snapf fused to the target protein Giantin C-terminal region (GOLGB1). Expression of the plasmid is driven by a CMV promoter. The construct contains an antibiotic resistance gene (AmpR) for bacterial selection and an origin of replication (ori). The map was generated by the Geneious Prime 2025.0.3 software.

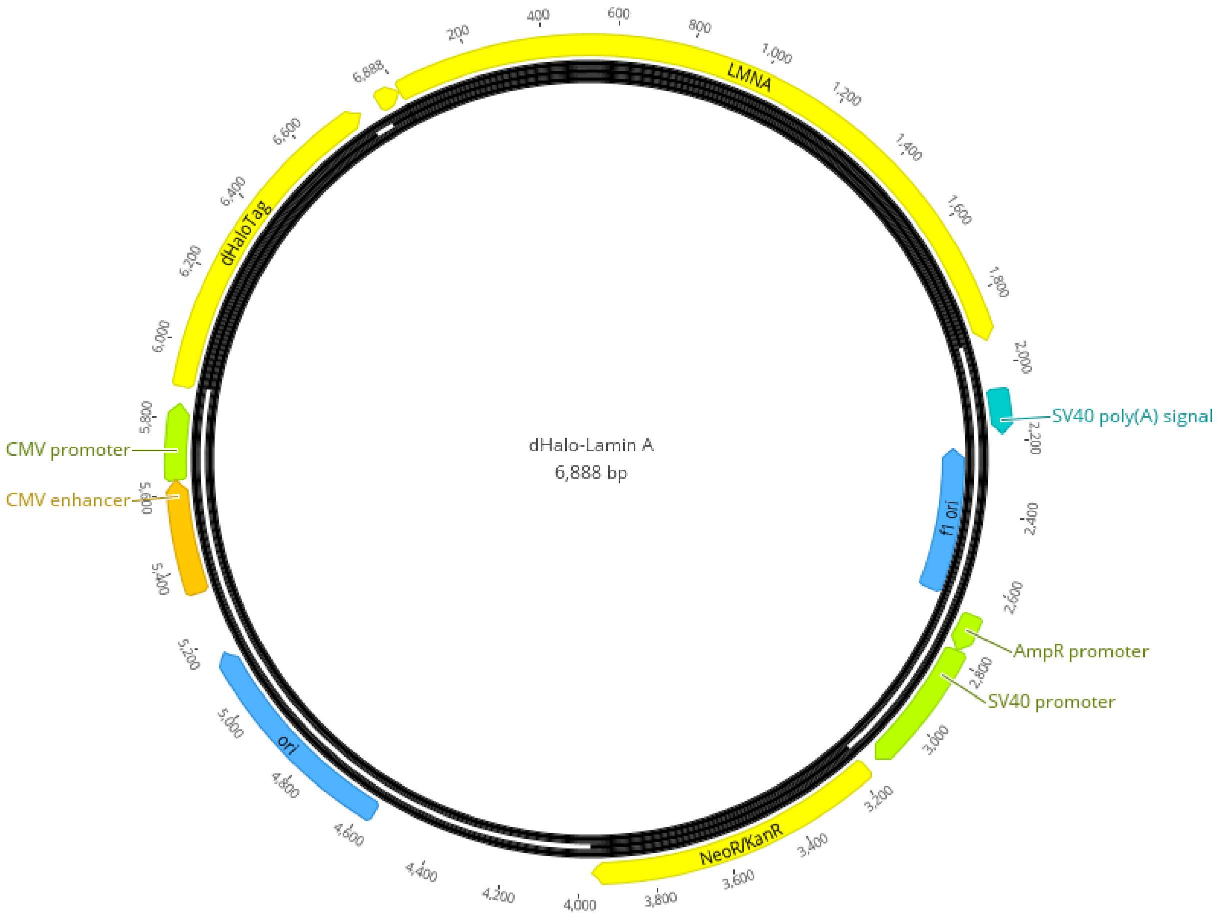

Figure S19. Annotated vector map of the engineered plasmid dHalo-LaminA used for expression in mammalian cells. The plasmid is based on pSnapf-C1 backbone and was engineered to express N-terminal dHalo fused to the target protein Lamin A (LMNA). Expression of the plasmid is driven by a CMV promoter. The construct contains an antibiotic resistance gene (KanR) for bacterial selection and an origin of replication (ori). The map was generated by the Geneious Prime 2025.0.3 software.

Supplementary Methods

General experimental information and synthesis

Thin layer chromatography

Analytical TLC (normal phase) was performed on Merck Millipore ready-to-use aluminum sheets coated with silica gel 60 (F_{254}) (Cat. No. 1.05554.0001). Analytical TLC on reversed phase (RP-C_{18}) was performed on Merck Millipore ready-to-use aluminum sheets coated with RP-18 60 (F_{254}s) (Cat. No. 1.05560.0001). Compounds were detected by exposing TLC plates to UV-light (254 or 366 nm) or by heating with vanillin stain (6 g vanillin and 1.5 mL conc. H_2SO_4 in 100 mL ethanol) or DNPH stain (0.5 g 2,4-dinitrophenylhydrazine hydrochloride in 100 mL 2 N HCl).

Preparative flash column chromatography

Automated separations on normal phase (silica gel) were performed with an Isolera Spektra One system (Biotage AG, Sweden) using commercially available cartridges of suitable size (BGB Scorpius 40-60 µm silica series from BGB Analytik AG, Puriflash Silica HP 30µm series from Interchim) and solvent gradient indicated.

High-Performance Liquid Chromatography (HPLC) and Mass Spectrometry (MS)

Analytical liquid chromatography-mass spectrometry was performed on an LC-MS system (Shimadzu): 2x LC-20AD HPLC pumps with DGU-20A3R solvent degassing unit, SIL-20ACHT autosampler, CTO-20AC column oven, SPD-M30A diode array detector and CBM-20A communication bus module, integrated with CAMAG TLC-MS interface 2, FCV-20AH_2 diverter valve and LCMS-2020 spectrometer with electrospray ionization (ESI, 100 – 1500 m/z). Analytical column: Hypersil GOLD 50×2.1 mm 1.9µm, standard conditions: sample volume 1-2 µL, solvent flow rate 0.5 mL/min, column temperature 30 °C. General method: isocratic 90:10 A:B over 2 min, then gradient 90:10 to 1:99 A:B over 5 min, then isocratic 1:99 A:B over 2 min; solvent A – water + 0.1% (v/v) HCO_2H, solvent B – acetonitrile + 0.1% (v/v) HCO_2H.

High resolution mass spectra (HRMS) were obtained on a maXis II ETD (Bruker) with electrospray ionization (ESI) at the Mass Spectrometry Core facility of the Max-Planck Institute for Medical Research (Heidelberg, Germany).

Preparative high-performance liquid chromatography was performed on an Interchim puriFlash 5.20P system using the suitable preparative columns and conditions as indicated for individual preparations. Method scouting was performed on a HPLC system (Shimadzu): 2x LC-20AD HPLC pumps with DGU-20A3R solvent degassing unit, CTO-20AC column oven equipped with a manual injector with a 20 µL sample loop, SPD-M20A diode array detector, RF-20A

fluorescence detector and CBM-20A communication bus module; or on a Dionex Ultimate 3000 UPLC system: LPG-3400SD pump, WPS-3000SL autosampler, TCC-3000SD column compartment with 2× 7-port 6-position valves and DAD-3000RS diode array detector. The test runs were performed on analytical columns with matching phases (HPLC: Interchim 250×4.6 mm 10 µm C18HQ, Interchim 250×4.6 mm 5 µm PhC4, solvent flow rate 1.2 mL/min; UPLC: Interchim C18HQ or PhC4 75×2.1 mm 2.2 µm, ThermoFisher Hypersil GOLD 100×2.1 mm 1.9 µm, solvent flow rate 0.5 mL/min).

NMR spectra

NMR spectra were recorded at 25 °C (unless indicated otherwise) with a Bruker Ascend 400 spectrometer at 400.15 MHz (^{1}H), 376.52 MHz (^{19}F) and 100.62 MHz (^{13}C) and are reported in ppm. All ^{1}H spectra are referenced to tetramethylsilane as an internal standard (δ = 0.00 ppm). ^{13}C spectra are referenced to tetramethylsilane (*δ* = 0 ppm) using the signals of the solvent: $CDCl_3$ (77.16 ppm), DMSO-d_6 (39.52 ppm), $\underline{C}D_3CN$ (1.32 ppm) or pyridine-d_5 (150.35 ppm, $\underline{C}$-2,6). Multiplicities of the signals are described as follows: s = singlet, d = doublet, t = triplet, q = quartet, m = multiplet or overlap of non-equivalent resonances; br = broad signal. Coupling constants $^{n}J_{X\text{-}Y}$ are given in Hz, where *n* is the number of bonds between the coupled nuclei X and Y ($J_{H\text{-}H}$ are always listed as *J* without indices).

Melting points

Melting points of crystalline samples (crystallization solvents indicated in parentheses) were determined whenever possible using Cole-Parmer® Stuart™ SMP50 Automatic Melting Point apparatus according to the manufacturer's instructions. All 3-aminobenzothiophene 1,1-dioxides **15** (with the sole exceptions of compounds **15b** and **15k**) were found to discolor and decompose without melting above 200 °C or 250 °C (visible decomposition range onset indicated for individual compounds).

Optical spectroscopy

Absorption spectra were recorded with a Varian Cary 4000 UV-Vis double-beam spectrophotometer (Agilent Technologies, USA). The emission spectra were recorded with a Varian Cary Eclipse fluorescence spectrophotometer (Agilent). The spectra were recorded in quartz cuvettes (light path 10 mm; Hellma Analytics, cat. # 119-10-40). For the data shown in Figure S4, absorption and fluorescence emission spectra were recorded in triplicate with a CLARIOstar Plus microplate reader (BMG LABTECH GmbH, Germany) in 96-well microplates

(200 µL/well): non-binding polystyrene F-bottom, µClear (Greiner Bio-One GmbH, Ref. 655906). All spectra were recorded at 25 °C in air-saturated solvents and are background corrected.

Fluorescence quantum yields (absolute method determinations) were obtained with a Quantaurus-QY absolute PL quantum yield spectrometer (model C11347-11, Hamamatsu) according to the manufacturer's instructions. Fluorescence lifetimes were measured with a FluoTime 300 fluorescence lifetime spectrometer (PicoQuant, controlled with the EasyTau1.4 software). All measurements were performed in air-saturated solvents at ambient temperature.

Plasmids

The plasmid for Snapf-GianCreg (see Figure S18) was engineered by PCR amplification of the coding sequence of SNAPf using the forward primer 5'-CAAGCTTGGTACCGCCACCATGGACAAAGACTGCGAAATG-3' (KpnI) and the reverse primer 3'-ACCATCGAATTCTACTGCACCCAGCCCAGGCTTG-5' (EcoRI). The fragment was ligated into the equally digested backbone of the pcDNA3/td5StayGold(c4)=GianCreg plasmid, which was a gift from Atsushi Miyawaki (Addgene plasmid #212022; http://n2t.net/addgene:212022; RRID:Addgene_212022) using KpnI and EcoRI restriction enzymes.

pCLIPf-Cox8A Control Plasmid was a gift from New England Biolabs & Ana Egana (Addgene plasmid #101130; http://n2t.net/addgene:101130; RRID:Addgene_101130).

pSNAPf-Clathrin was kindly provided by the laboratory of Francesca Bottanelli (Freie Universität Berlin; https://www.bcp.fu-berlin.de/en/chemie/biochemie/research-groups/bottanellli/index.html).

pSNAPf-LaminA-C (listed as LaminA-C-18) was a gift from Michael Davidson (Addgene plasmid #58193; http://n2t.net/addgene:58193; RRID:Addgene_58193).

pdHalo-LaminA-C (see Figure S19): the construct expressing dHalo-Lamin A-C-18 was generated by replacing SNAPf of pSNAPf-LaminA-C with corresponding HaloTag7. HaloTag7 was cut out of another plasmid Halo7-Sec61b-C1 (manuscript in preparation) using flanking NheI and BglII restriction enzymes (digested products were separated on a gel, correct bands were cut out, purified and ligated with T4 DNA ligase). Q5® Site-Directed Mutagenesis Kit (E0554S, New England Biolabs) with designed forward 5'-ATTCACGCCTGGGGCTCC-3' and reverse primer 5'-GACCAGGACGACCTCTTCCA-3' were used to mutate the residue Asp106 in HaloTag7 to Ala (D106A), resulting in dHaloTag7.[S1]

The coding sequences of all recombinant plasmids were confirmed by whole plasmid sequencing.

Transfection

Cells were transfected with 0.5 µg plasmid per well using 1 µL Lipofectamine 2000 (ThermoFisher Scientific, cat. # 11668019) according to the manufacturer's instructions and imaged 24 h after transfection.

Cell culture and labeling

U2OS-Vim-Halo[S2] or U2OS-HaloTag-Lamin A/C[S3] cells were cultured in DMEM (Dulbecco's modified Eagle medium), high glucose, GlutaMAX Supplement, pyruvate (ThermoFisher, cat. # 10569010), supplemented with 10% (v/v) fetal bovine serum (FBS, ThermoFisher 10500064) and 1% penicillin-streptomycin (ThermoFisher, cat. # 15140122) in a humidified 5% CO_2 incubator at 37 °C. Cells were split at 80-90% confluency and regularly tested for mycoplasma contamination. Prior to labelling, cells were grown for 48 h on glass coverslips. For labeling with covalent ligands (Halo, SNAP, CLIP, HTL.2), a stock solution of the fluorescent probe (500 µM - 2 mM in DMSO) was diluted in the cell medium to the desired final concentration of the probe, ensuring that the final DMSO content of the medium does not exceed 0.1%. Cells were incubated (1 h to overnight, see Figures 4,5 and Figures S11-S17 for the individual labelling conditions) with this solution and then the medium was exchanged with dye-free medium and incubated for 30 minutes (this washing process was repeated one more time).

Exchangeable fluorescent ligands (T5 and Hy4 for HaloTag, fluorescent taxoid derivatives for tubulin and Hoechst 33342 derivatives for dsDNA) were added 1 h prior to imaging in complete medium, incubated for 1 h (15 min for Hoechst 33342) and imaged without washing. In some experiments (see Figure 5,F-J, Figure S15 and Figure S16,E-H), verapamil was added (10 µM final concentration) to enhance tubulin staining. For imaging, the coverslips with labeled cells and the media were transferred to live-cell magnetic chambers (Live Cell Instrument Co. Ltd., cat. # CM-B18-1) and imaged within 1 h.

Cell viability

The viability of non-transfected U2 O-S in the presence of fluorescent ligands (**20-Halo**, **20-HTL2**, **20-CLIP** and **20-SNAP**) was evaluated by relative quantification of amount of ATP present (which correlates with the number of metabolically active cells) using CellTiter-Glo® 2.0 chemiluminescence-based assay (Promega, cat. # G9242) in 96-well chimney well high-binding

white polystyrene, sterile, ATP-free microplates (Promega, E5650). The assay was performed according to the supplier's protocol (https://www.promega.de/en/products/cell-health-assays/cell-viability-and-cytotoxicity-assays/celltiter_glo-2_0-assay/) using a CLARIOstar Plus microplate reader (BMG LABTECH GmbH, Germany) as luminometer. More specifically, U2 O-S cells were seeded in 96-well flat-bottom white microplates (100 µL/well, ~ 5×10^3 cells/well) and kept at 37 °C in humidified air with 5% CO_2. The next day, the cells were treated with 0.37/1.1/3.3/10 µM dilutions of a fluorescent probe (**20-Halo**, **20-HTL2**, **20-CLIP** and **20-SNAP**, in ×12 replicates per condition) **3-Halo** or **5-Halo** in Fluorobrite media over 24 h (with 0.1% DMSO in the media; Fluorobrite media (Cat. No. A1896701, Gibco) was supplemented with 10% (v/v) fetal bovine serum (Cat. No. 10500064, Thermo Fisher Scientific), 2% GlutaMAX (Cat. No. 35050061, Gibco) and 1% penicillin/streptomycin). For negative control, and 0.1% DMSO in Fluorobrite were used; 10% DMSO in Fluorobrite was employed as positive control. The plates were then removed from the incubator, the media was replaced with 100 µL/well of fresh probe-free Fluorobrite and the plates were equilibrated to rt (22 °C) over 30 min. An equal volume (100 µL/well) of CellTiter-Glo® 2.0 reagent was added, the well contents were mixed by shaking the plates on orbital shaker (300 rpm, 2 min), and the plates were incubated for further 10 min at rt (protected from light) before reading the luminescent signal. The readout was performed with the following settings of CLARIOstar Plus reader: luminescence endpoint assay, optic: top, well scan: matrix scan 4×4 (diameter 4 mm), no emission filter, using enhanced dynamic range and 96/384 aperture holder.

Confocal and STED microscopy

Imaging was performed with an Abberior expert line microscope (Abberior Instruments GmbH) built on a motorized inverted microscope IX83 (Olympus). The microscope is equipped with 405 nm excitation CW laser, a 640 nm excitation pulsed (40 MHz) laser, and a 775 nm pulsed (40 MHz) STED laser shaped by a Spatial Light Modulator. Spectral detection was performed with avalanche photodiodes (APD) in the indicated spectral windows. Images were acquired with a 100x/1.40 UPlanSApo Oil immersion objective lens (Olympus), and the pinhole set to 100 µm (1 Airy unit). A *z*-focus drift compensation unit was used to minimized axial drift during measurement. The imaging parameters for live-cell confocal and STED images presented in this work are summarized in Table S2.

The general 3-channel imaging scheme is schematized in Figure S7, showing the spectra of three representative dyes, laser lines and detection channels. Channels were imaged

sequentially for the following reasons: (1) to minimize cross-talk between channels, (2) because the detection ranges of the channels (Spectral detection unit of the microscope) cannot be adjusted during the image acquisition, and (3) to allow for focus optimization for each of the corresponding structures, which are in general located at different planes within the cell.

Preparation of 2-hydroxybenzophenone triflates 1

1d

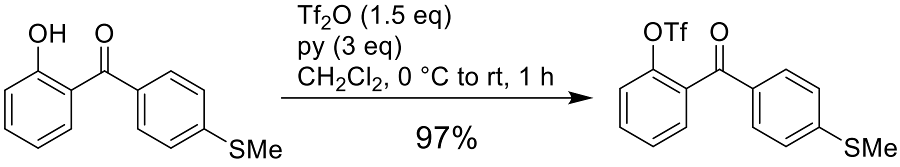

1d

Trifluoromethanesulfonic anhydride (0.66 mL, 3.95 mmol, 1.5 eq) was added quickly dropwise to a stirred solution of 2-hydroxy-4'-(methylthio)benzophenone (2.64 mmol; known compound[S4]) and pyridine (0.64 mL, 7.90 mmol, 3 equiv) in dry CH_2Cl_2 (10 mL), cooled in ice-water bath. The reaction mixture was allowed to warm up and left stirring at rt for 1 h. The mixture was then diluted with 0.1 N HCl (50 mL), extracted with CH_2Cl_2 (3×25 mL), the combined extracts were washed with brine (50 mL) and dried over Na_2SO_4. The product was isolated by flash column chromatography (12 g Interchim SiHP 30 µm cartridge, gradient 5% to 50% EtOAc/hexane) and freeze-dried from 1,4-dioxane to give 961 mg (97%) of **1d** as white solid.

mp (EtOAc/hexane): 50-51 °C.

^{1}H NMR (400 MHz, $CDCl_3$): δ 7.75 – 7.70 (m, 2H), 7.62 (ddd, *J* = 8.3, 7.3, 1.9 Hz, 1H), 7.55 (dd, *J* = 7.5, 1.9 Hz, 1H), 7.48 (td, *J* = 7.5, 1.1 Hz, 1H), 7.42 (d, *J* = 8.3 Hz, 1H), 7.31 – 7.25 (m, 2H), 2.53 (s, 3H).

^{19}F NMR (376 MHz, $CDCl_3$): δ -73.43.

^{13}C NMR (101 MHz, $CDCl_3$): δ 191.7, 147.5, 146.8, 132.8, 132.7, 132.6, 131.1, 130.7, 128.2, 125.0, 122.6, 118.6 (q, *J* = 320.5 Hz), 14.8.

HRMS ($C_{15}H_{11}F_3O_4S_2$): *m/z* (positive mode) = 377.0123 (found $[M+H]^+$), 377.0124 (calc.).

1g

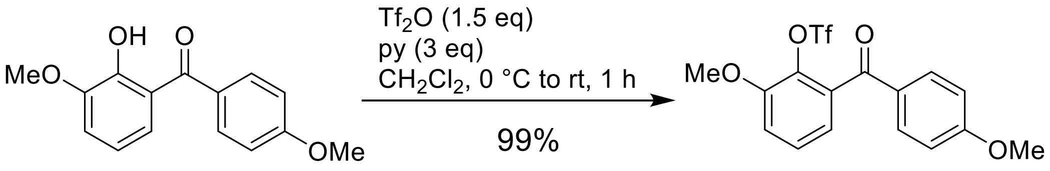

1g

Trifluoromethanesulfonic anhydride (0.36 mL, 2.14 mmol, 1.5 eq) was added quickly dropwise to a stirred solution of 2-hydroxy-3,4'-dimethoxybenzophenone (1.43 mmol; known compound[S4]) and pyridine (0.35 mL, 4.29 mmol, 3 equiv) in dry CH_2Cl_2 (6 mL), cooled in ice-

water bath. The reaction mixture was allowed to warm up and left stirring at rt for 1 h. The mixture was then diluted with 0.1 N HCl (70 mL), extracted with CH_2Cl_2 (3×20 mL), the combined extracts were washed with brine (50 mL) and dried over Na_2SO_4. The product was isolated by flash column chromatography (12 g Interchim SiHP 30 µm cartridge, gradient 10% to 80% EtOAc/hexane) and freeze-dried from 1,4-dioxane to give 557 mg (99%) of **1g** as light tan solid.

mp (EtOAc/hexane): 65-66 °C.
^{1}H NMR (400 MHz, $CDCl_3$): δ 7.84 – 7.77 (m, 2H), 7.43 – 7.36 (m, 1H), 7.19 (dd, *J* = 8.4, 1.5 Hz, 1H), 7.06 (dd, *J* = 7.7, 1.5 Hz, 1H), 6.97 – 6.90 (m, 2H), 3.97 (s, 3H), 3.88 (s, 3H).
^{19}F NMR (376 MHz, $CDCl_3$): δ -73.70.
^{13}C NMR (101 MHz, $CDCl_3$): δ 191.1, 164.3, 151.8, 136.0, 134.3, 132.8, 129.5, 128.7, 121.7, 118.5 (q, *J* = 320.7 Hz), 115.0, 114.0, 56.6, 55.7.
HRMS ($C_{16}H_{13}F_3O_6S$): *m/z* (positive mode) = 391.0460 (found $[M+H]^+$), 391.0458 (calc.).

1j

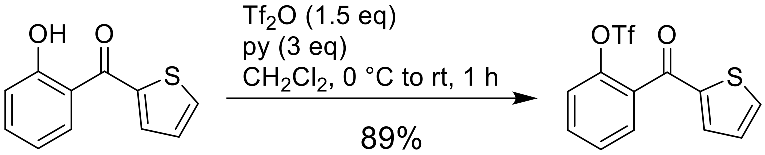

1j

Trifluoromethanesulfonic anhydride (0.76 mL, 4.50 mmol, 1.5 equiv) was added quickly dropwise to a stirred solution of (2-hydroxyphenyl)(thiophen-2-yl)methanone (3 mmol; known compound[S4]) and pyridine (0.73 mL, 9 mmol, 3 equiv) in dry CH_2Cl_2 (12 mL), cooled in ice-water bath. The reaction mixture was allowed to warm up and left stirring at rt for 1 h. The mixture was then diluted with 0.2 N HCl (50 mL), extracted with CH_2Cl_2 (3×20 mL), the combined extracts were washed with brine (50 mL) and dried over Na_2SO_4. The product was isolated by flash column chromatography (25 g Interchim SiHP 30 µm cartridge, gradient 0% to 50% CH_2Cl_2/hexane) and dried *in vacuo* to give 898 mg (89%) of **1j** as colorless oil.

^{1}H NMR (400 MHz, $CDCl_3$): δ 7.79 (dd, *J* = 4.9, 1.2 Hz, 1H), 7.69 (dd, *J* = 7.6, 1.8 Hz, 1H), 7.63 (ddd, *J* = 8.3, 7.6, 1.8 Hz, 1H), 7.54 – 7.48 (m, 2H), 7.44 (dd, *J* = 8.3, 1.1 Hz, 1H), 7.15 (dd, *J* = 4.9, 3.8 Hz, 1H).
^{19}F NMR (376 MHz, $CDCl_3$): δ -73.47.
^{13}C NMR (101 MHz, $CDCl_3$): δ 184.3, 146.5, 143.5, 136.1, 136.0, 132.7, 132.6, 130.8, 128.4, 128.2, 122.7, 118.6 (q, *J* = 320.5 Hz).
HRMS ($C_{12}H_7F_3O_4S_2$): *m/z* (positive mode) = 336.9812 (found $[M+H]^+$), 336.9811 (calc.).

S1

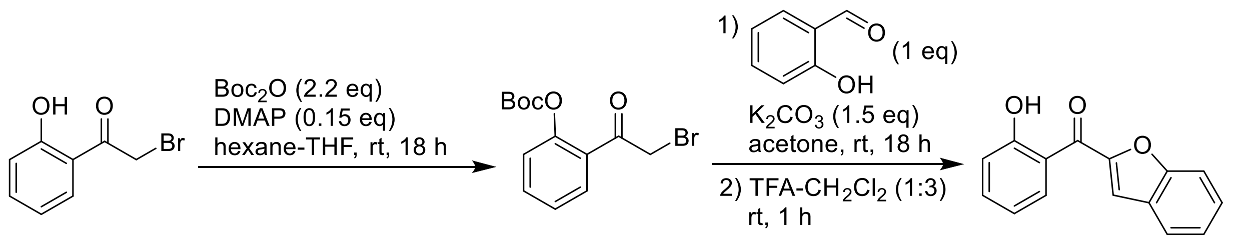

4-(Dimethylamino)pyridine (DMAP; 43 mg, 0.35 mmol, 0.15 equiv) was added to the stirred solution of 2-bromo-1-(2-hydroxyphenyl)ethanone (500 mg, 2.33 mmol) and di-*tert*-butyl dicarbonate (Boc_2O; 1.12 g, 5.13 mmol, 2.2 equiv) in hexane (6 mL) and THF (2 mL), and the resulting suspension was stirred at rt overnight (18 h). It was then diluted with EtOAc (50 mL), washed with 1 N HCl + brine (1:1, 50 mL), sat. aq. $NaHCO_3$ (50 mL), brine (30 mL) and dried over Na_2SO_4. The filtrate was evaporated to orange oil (1.20 g), which was used directly in the next step.

Following the procedure from [S5], this crude material was dissolved in dry acetone (5 mL), salicylaldehyde (0.25 mL, 2.33 mmol, 1 equiv) and K_2CO_3 (483 mg, 3.5 mmol, 1.5 equiv) were added, and the mixture as stirred vigorously overnight (18 h) at rt. It was then diluted with sat. aq. $NaHCO_3$ (50 mL), extracted with EtOAc (3×25 mL), the combined extracts were washed with brine and evaporated to viscous brown oil. It was dissolved in CH_2Cl_2 (10 mL), trifluoroacetic acid (3 mL) was added, and the mixture was stirred at rt for 1 h. The mixture was diluted with equal volume of toluene, evaporated and chased with toluene (2×25 mL), the residue was dissolved in CH_2Cl_2 and evaporated on silica. The product was isolated by flash column chromatography (25 g Interchim SiHP 30 µm cartridge, gradient 10% to 60% CH_2Cl_2/hexane) and freeze-dried from 1,4-dioxane to give 274 mg (49%) of **S1** as yellow solid.

^{1}H NMR (400 MHz, $CDCl_3$): δ 12.03 (s, 1H), 8.40 (dd, *J* = 8.1, 1.7 Hz, 1H), 7.76 (dt, *J* = 8.0, 1.0 Hz, 1H), 7.69 (d, *J* = 1.0 Hz, 1H), 7.66 (dq, *J* = 8.4, 0.9 Hz, 1H), 7.60 – 7.48 (m, 2H), 7.36 (ddd, *J* = 8.0, 7.2, 0.9 Hz, 1H), 7.08 (dd, *J* = 8.4, 1.2 Hz, 1H), 7.01 (ddd, *J* = 8.3, 7.2, 1.2 Hz, 1H).
^{13}C NMR (101 MHz, $CDCl_3$): δ 186.9, 163.6, 156.2, 152.3, 136.7, 131.9, 128.6, 126.9, 124.3, 123.4, 119.3, 119.2, 118.7, 116.8, 112.6.
HRMS ($C_{15}H_{13}O_3$): *m/z* (positive mode) = 239.0706 (found $[M+H]^+$), 239.0703 (calc.).

1k

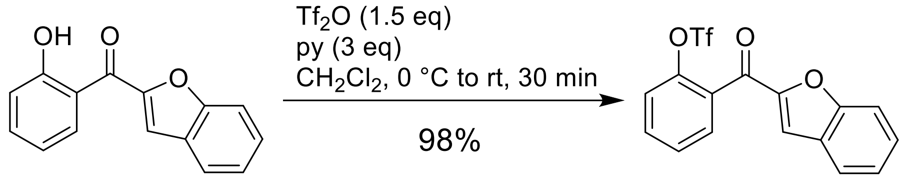

S1 **1k**

Trifluoromethanesulfonic anhydride (0.29 mL, 1.73 mmol, 1.5 equiv) was added quickly dropwise to a stirred solution of **S1** (274 mg, 1.15 mmol) and pyridine (0.28 mL, 3.45 mmol, 3 equiv) in dry CH_2Cl_2 (5 mL), cooled in ice-water bath. The reaction mixture was allowed to warm up and left stirring at rt for 30 min. The mixture was then diluted with 0.2 N HCl (50 mL), extracted with CH_2Cl_2 (3×25 mL), the combined extracts were washed with brine (50 mL) and dried over Na_2SO_4. The product was isolated by flash column chromatography (12 g Interchim SiHP 30 µm cartridge, gradient 10% to 60% EtOAc/hexane) and freeze-dried from 1,4-dioxane to give 417 mg (98%) of **1k** as yellow solid.

mp (EtOAc/hexane): 77-78 °C.

^{1}H NMR (400 MHz, $CDCl_3$): δ 7.81 (dd, *J* = 7.6, 1.8 Hz, 1H), 7.72 (dt, *J* = 7.9, 1.1 Hz, 1H), 7.68 (ddd, *J* = 8.3, 7.6, 1.8 Hz, 1H), 7.64 – 7.59 (m, 1H), 7.58 – 7.50 (m, 2H), 7.49 (d, *J* = 1.0 Hz, 1H), 7.47 (dd, *J* = 8.3, 1.1 Hz, 1H), 7.34 (ddd, *J* = 8.0, 7.1, 1.0 Hz, 1H).

^{19}F NMR (376 MHz, $CDCl_3$): δ -73.37.

^{13}C NMR (101 MHz, $CDCl_3$): δ 181.1, 156.6, 151.8, 146.9, 133.3, 131.7, 131.1, 129.3, 128.3, 127.0, 124.4, 123.8, 122.8, 118.6 (q, *J* = 320.5 Hz), 117.7, 112.9.

HRMS ($C_{16}H_9F_3O_5S$): *m/z* (positive mode) = 371.0195 (found $[M+H]^+$), 371.0196 (calc.).

1m

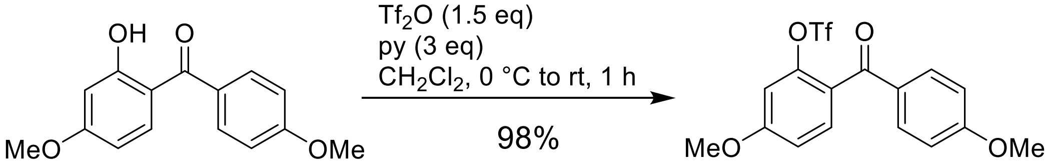

1m

Trifluoromethanesulfonic anhydride (0.66 mL, 3.95 mmol, 1.5 eq) was added quickly dropwise to a stirred solution of 2-hydroxy-4,4'-dimethoxybenzophenone (680 mg, 2.64 mmol; known compound[S4]) and pyridine (0.64 mL, 7.92 mmol, 3 equiv) in dry CH_2Cl_2 (10 mL), cooled in ice-water bath. The reaction mixture was allowed to warm up and left stirring at rt for 1 h. The mixture

was then diluted with 0.2 N HCl (50 mL), extracted with CH_2Cl_2 (3×20 mL), the combined extracts were washed with brine (50 mL) and dried over Na_2SO_4. The product was isolated by flash column chromatography (12 g Interchim SiHP 30 µm cartridge, gradient 10% to 60% EtOAc/hexane) and freeze-dried from 1,4-dioxane to give 1.00 g (98%) of **1m** as white solid.

mp (EtOAc/hexane): 102-103 °C.
^{1}H NMR (400 MHz, $CDCl_3$): δ 7.81 – 7.75 (m, 2H), 7.52 (d, *J* = 8.6 Hz, 1H), 6.98 – 6.92 (m, 3H), 6.90 (d, *J* = 2.4 Hz, 1H), 3.90 (s, 3H), 3.88 (s, 3H).
^{19}F NMR (376 MHz, $CDCl_3$): δ -73.47.
^{13}C NMR (101 MHz, $CDCl_3$): δ 191.0, 164.0, 162.7, 148.2, 132.8, 132.6, 130.1, 125.1, 118.6 (q, *J* = 320.7 Hz), 113.9, 113.2, 108.8, 56.1, 55.7.
HRMS ($C_{16}H_{13}F_3O_6S$): *m/z* (positive mode) = 391.0458 (found $[M+H]^+$), 391.0458 (calc.).

S2

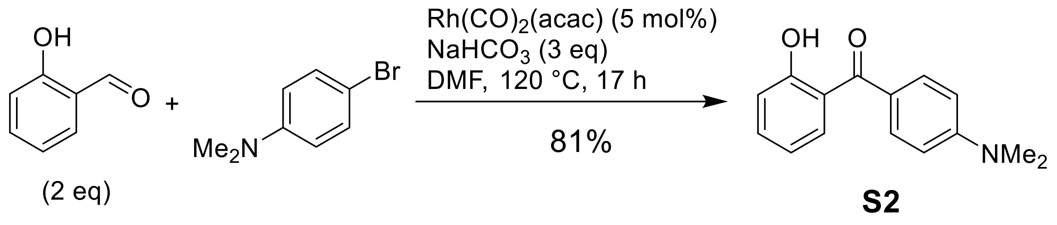

Following the procedure from [S4], a degassed mixture of salicylaldehyde (610 mg, 5 mmol, 2 equiv), 4-bromo-*N*,*N*-dimethylaniline (500 mg, 2.5 mmol), sodium bicarbonate (630 mg, 7.5 mmol, 3 equiv) and dicarbonyl(acetylacetonato)rhodium(I) (32 mg, 0.125 mmol, 5 mol%) in dry DMF (10 mL) was stirred at 120 °C overnight (17 h). On cooling, the mixture was extracted with CH_2Cl_2 (3×40 mL) from water (100 mL, adjusted to pH 3-4 with 1 N HCl), the combined extracts were washed with water (2×150 mL), brine and dried over Na_2SO_4. The product was isolated by flash column chromatography (40 g BGB Scorpius silica 50 µm cartridge, gradient 5% to 50% EtOAc/hexane) and freeze-dried from 1,4-dioxane to give 490 mg (81%) of **S2** as yellow solid.

^{1}H NMR (400 MHz, $CDCl_3$): δ 12.01 (s, 1H), 7.75 – 7.70 (m, 2H), 7.68 (dd, *J* = 8.0, 1.7 Hz, 1H), 7.45 (ddd, *J* = 8.7, 7.2, 1.7 Hz, 1H), 7.04 (dd, *J* = 8.4, 1.2 Hz, 1H), 6.87 (ddd, *J* = 8.3, 7.2, 1.2 Hz, 1H), 6.74 – 6.68 (m, 2H), 3.08 (s, 6H).
^{13}C NMR (101 MHz, $CDCl_3$): δ 199.0, 162.7, 153.3, 135.1, 133.2, 132.5, 125.0, 120.1, 118.4, 118.2, 110.8, 40.2.
HRMS ($C_{15}H_{15}NO_2$): *m/z* (positive mode) = 242.1174 (found $[M+H]^+$), 242.1176 (calc.).

1n

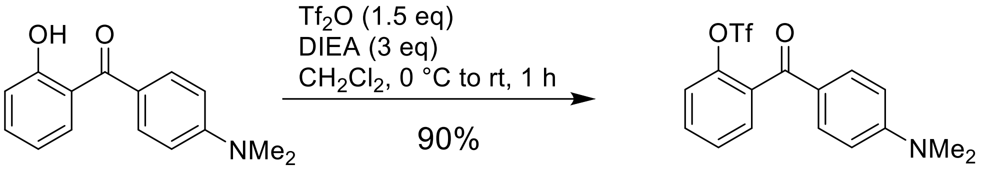

S2 **1n**

Trifluoromethanesulfonic anhydride (0.50 mL, 2.97 mmol, 1.5 equiv) was added quickly dropwise to a stirred solution of **S2** (478 mg, 1.98 mmol) and *N*,*N*-diisopropylethylamine (DIEA; 1.0 mL, 5.94 mmol, 3 equiv) in dry CH_2Cl_2 (6 mL), cooled in ice-water bath. The resulting red-brown solution was allowed to warm up and left stirring at rt for 1 h. The mixture was then diluted with water (100 mL), extracted with CH_2Cl_2 (3×25 mL), the combined extracts were washed with brine (50 mL) and dried over Na_2SO_4. The product was isolated by flash column chromatography (25 g Interchim SiHP 30 µm cartridge, gradient 10% to 70% EtOAc/hexane) and freeze-dried from 1,4-dioxane to give 668 mg (90%) of **1n** as yellow solid.

mp (EtOAc/hexane): 101-111 °C.

^{1}H NMR (400 MHz, $CDCl_3$): δ 7.76 – 7.69 (m, 2H), 7.58 – 7.51 (m, 2H), 7.48 – 7.42 (m, 1H), 7.39 (dd, *J* = 8.2, 1.1 Hz, 1H), 6.68 – 6.62 (m, 2H), 3.08 (s, 6H).

^{19}F NMR (376 MHz, $CDCl_3$): δ -73.61.

^{13}C NMR (101 MHz, $CDCl_3$): δ 190.3, 154.1, 146.7, 134.0, 132.8, 131.5, 130.9, 128.0, 124.2, 122.3, 118.6 (q, *J* = 320.6 Hz), 110.7, 40.2.

HRMS ($C_{16}H_{14}F_3NO_4S$): *m/z* (positive mode) = 374.0668 (found $[M+H]^+$), 374.0668 (calc.).

1p

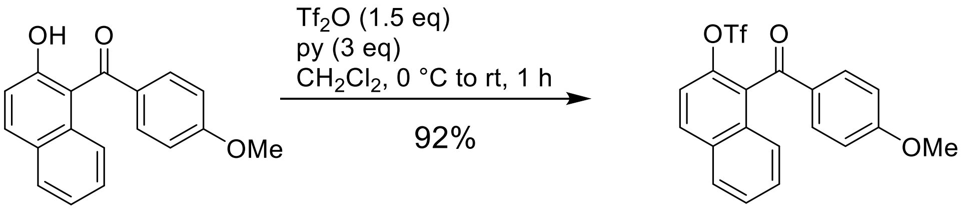

1p

Trifluoromethanesulfonic anhydride (0.76 mL, 4.50 mmol, 1.5 equiv) was added quickly dropwise to a stirred solution of (2-hydroxynaphthalen-1-yl)(4-methoxyphenyl)methanone (834 mg, 3 mmol; known compound[S4]) and pyridine (0.73 mL, 9.00 mmol, 3 equiv) in dry CH_2Cl_2 (12 mL), cooled in ice-water bath. The resulting solution was allowed to warm up and left stirring at rt for 1 h. The mixture was then diluted with 0.2 N HCl (50 mL), extracted with CH_2Cl_2 (3×20 mL), the combined extracts were washed with brine (50 mL) and dried over Na_2SO_4. The product

was isolated by flash column chromatography (12 g Interchim SiHP 30 µm cartridge, gradient 10% to 70% EtOAc/hexane) and freeze-dried from 1,4-dioxane to give 1.13 g (92%) of **1p** as light grey solid.

mp (EtOAc/hexane): 84-86 °C.
^{1}H NMR (400 MHz, $CDCl_3$): δ 8.03 (d, *J* = 9.2 Hz, 1H), 7.95 (d, *J* = 8.2 Hz, 1H), 7.80 (d, *J* = 8.3 Hz, 2H), 7.72 – 7.64 (m, 1H), 7.63 – 7.55 (m, 1H), 7.52 (dd, *J* = 8.7, 2.0 Hz, 2H), 6.99 – 6.86 (m, 2H), 3.87 (s, 3H).
^{19}F NMR (376 MHz, $CDCl_3$): δ -73.73.
^{13}C NMR (101 MHz, $CDCl_3$): δ 191.8, 164.8, 143.2, 132.6, 132.4, 131.8, 131.5, 130.3, 130.2, 128.5, 128.4, 127.6, 126.0, 119.3, 118.5 (q, *J* = 320.2 Hz), 114.3, 55.7.
HRMS ($C_{19}H_{13}F_3O_5S$): *m/z* (positive mode) = 411.0509 (found $[M+H]^+$), 411.0509 (calc.).

1q

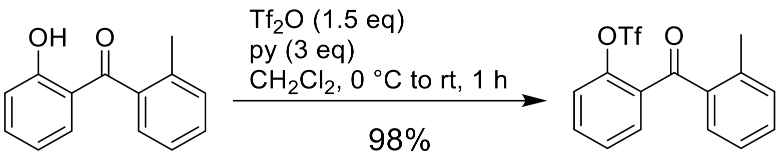

1q

Trifluoromethanesulfonic anhydride (0.36 mL, 2.17 mmol, 1.5 equiv) was added quickly dropwise to a stirred solution of 2-hydroxy-2'-methylbenzophenone (306 mg, 1.44 mmol; known compound[S4]) and pyridine (0.35 mL, 4.32 mmol, 3 equiv) in dry CH_2Cl_2 (6 mL), cooled in ice-water bath. The resulting solution was allowed to warm up and left stirring at rt for 1 h. The mixture was then diluted with 0.1 N HCl (50 mL), extracted with CH_2Cl_2 (3×20 mL), the combined extracts were washed with brine (50 mL) and dried over Na_2SO_4. The product was isolated by flash column chromatography (12 g Interchim SiHP 30 µm cartridge, gradient 10% to 80% EtOAc/hexane) and dried *in vacuo* to give 484 mg (98%) of **1q** as light pink oil.

^{1}H NMR (400 MHz, $CDCl_3$): δ 7.62 (ddd, *J* = 8.2, 7.4, 1.9 Hz, 1H), 7.57 (dd, *J* = 7.7, 1.8 Hz, 1H), 7.49 – 7.41 (m, 2H), 7.38 (dd, *J* = 8.3, 1.1 Hz, 1H), 7.36 – 7.30 (m, 2H), 7.24 – 7.19 (m, 1H), 2.53 (s, 3H).
^{19}F NMR (376 MHz, $CDCl_3$): δ -73.42.
^{13}C NMR (101 MHz, $CDCl_3$): δ 194.6, 147.2, 139.7, 136.7, 133.5, 133.3, 132.4, 132.2, 132.0, 131.1, 128.4, 125.6, 122.6, 118.7 (q, *J* = 320.7 Hz), 20.9.
HRMS ($C_{15}H_{11}F_3O_4S$): *m/z* (positive mode) = 345.0404 (found $[M+H]^+$), 345.0403 (calc.).

1ab

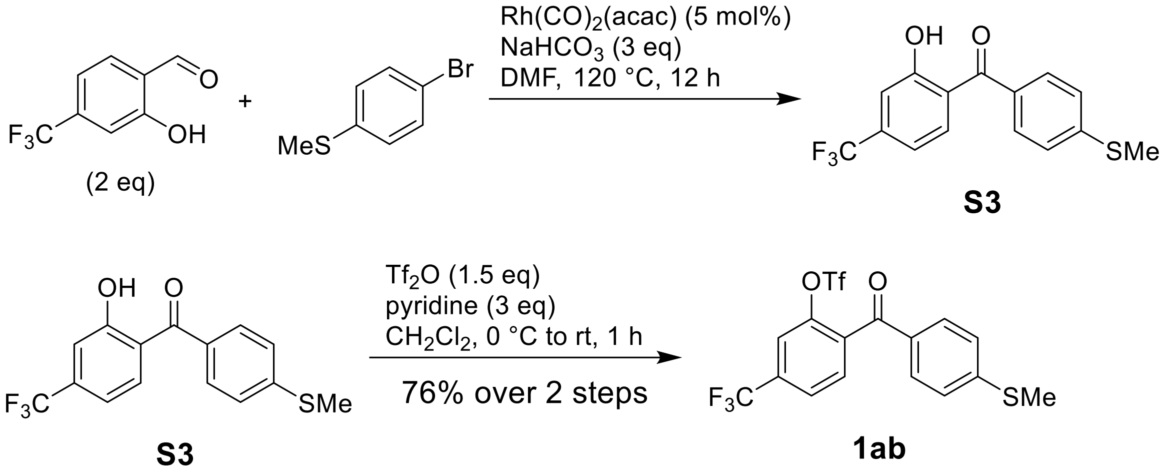

Following the procedure from [S4], a degassed mixture of 2-hydroxy-4-(trifluoromethyl) benzaldehyde (570 mg, 3 mmol, 2 equiv), 4-bromothioanisole (305 mg, 1.5 mmol), sodium bicarbonate (378 mg, 4.5 mmol, 3 equiv) and dicarbonyl(acetylacetonato)rhodium(I) (19.4 mg, 0.075 mmol, 5 mol%) in dry DMF (6 mL) was stirred at 120 °C for 12 h in a 50 mL round-bottom flask. On cooling, the mixture was extracted with CH_2Cl_2 (3×25 mL) from 0.5 N HCl (50 mL), the combined extracts were washed with water (2×60 mL), brine and dried over Na_2SO_4. The intermediate product **S3** was isolated by flash column chromatography (25 g Interchim SiHP 30 µm cartridge, gradient 0% to 20% EtOAc/hexane) and evaporated to give 432 mg of crude material as viscous yellow oil which was used directly in the following step.

Trifluoromethanesulfonic anhydride (0.35 mL, 2.08 mmol, ~1.5 equiv) was added quickly dropwise to a stirred solution of crude **S3** (430 mg, <1.38 mmol) and pyridine (0.35 mL, 4.20 mmol, 3 equiv) in dry CH_2Cl_2 (5 mL), cooled in ice-water bath. The reaction mixture was allowed to warm up and left stirring at rt for 1 h. It was then diluted with 0.1 N HCl (50 mL), extracted with CH_2Cl_2 (3×20 mL), the combined extracts were washed with brine (50 mL) and dried over Na_2SO_4. The product was isolated by flash column chromatography (12 g Interchim SiHP 30 µm cartridge, gradient 5% to 50% EtOAc/hexane) and freeze-dried from 1,4-dioxane to give 506 mg (76% over two steps) of **1ab** as white solid.

mp (EtOAc/hexane): 104-105 °C.
^{1}H NMR (400 MHz, $CDCl_3$): δ 7.77 (ddd, *J* = 8.0, 1.6, 0.8 Hz, 1H), 7.73 – 7.66 (m, 4H), 7.32 – 7.27 (m, 2H), 2.54 (s, 3H).
^{19}F NMR (376 MHz, $CDCl_3$): -63.01, -73.19.

^{13}C NMR (101 MHz, $CDCl_3$): δ 190.3, 148.5, 146.6, 136.3, 134.5 (q, J = 34.1 Hz), 131.9, 131.6, 130.63, 125.2 (q, J = 3.7 Hz), 125.1, 122.7 (q, J = 273.1 Hz), 120.0 (q, J = 4.1 Hz), 118.5 (q, J = 320.9 Hz), 14.8.
HRMS ($C_{16}H_{10}F_6O_4S_2$): m/z (positive mode) = 445.0003 (found $[M+H]^+$), 444.9997 (calc.).

S4

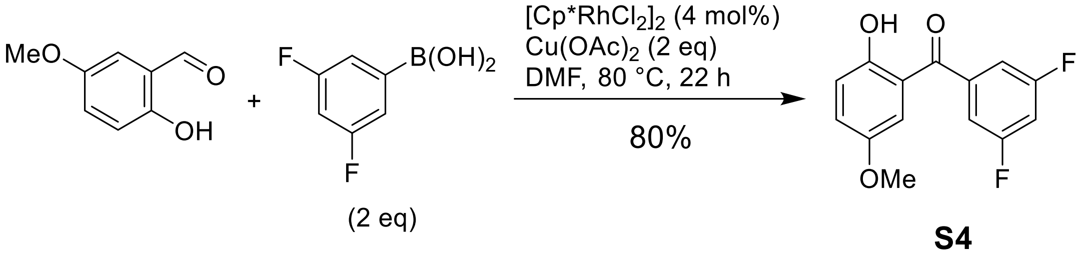

S4

Following the procedure from [S6], a degassed mixture of 5-methoxy-2-hydroxybenzaldehyde (374 µL, 3 mmol), 3,5-difluorophenylboronic acid (948 mg, 6 mmol), anhydrous copper(II) acetate (1.09 g, 6 mmol, 2 equiv) dichloro(pentamethylcyclopentadienyl)rhodium(III) dimer (74 mg, 0.12 mmol, 4 mol%) in dry DMF (22 mL) was stirred at 80 °C overnight (22 h). On cooling, the mixture was extracted with CH_2Cl_2 (3×40 mL) from sat. aq. NH_4Cl (100 mL), the combined extracts were washed with water (2×150 mL), brine and dried over Na_2SO_4. The product was isolated by flash column chromatography (25 g Interchim SiHP 30 µm cartridge, gradient 2% to 50% EtOAc/hexane) and dried *in vacuo* to give 630 mg (80%) of **S4** as bright yellow solid.

^1H NMR (400 MHz, $CDCl_3$): δ 11.28 (s, 1H), 7.25 – 7.16 (m, 3H), 7.08 – 7.01 (m, 2H), 6.97 (d, J = 3.1 Hz, 1H), 3.73 (s, 3H).
^{19}F NMR (376 MHz, $CDCl_3$): δ -107.55.
^{13}C NMR (101 MHz, $CDCl_3$): δ 198.3 (t, J = 2.4 Hz), 164.1 (d, J = 11.9 Hz), 161.6 (d, J = 12.0 Hz), 157.8, 151.8, 140.8 (t, J = 8.2 Hz), 125.0, 119.8, 118.1, 115.7, 112.3 (d, J = 11.3 Hz), 112.3 (d, J = 26.8 Hz), 107.4 (t, J = 25.1 Hz), 56.1.
HRMS ($C_{14}H_{10}F_2O_3$): m/z (positive mode) = 265.0672 (found $[M+H]^+$), 265.0671 (calc.).

1ad

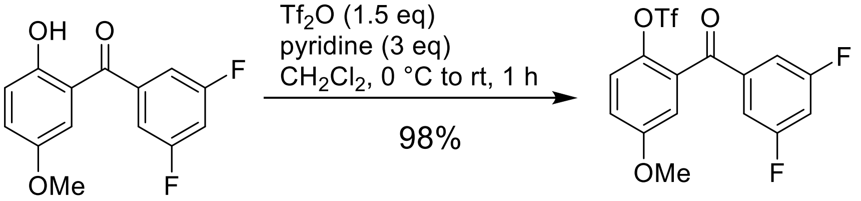

S4 **1ad**

Trifluoromethanesulfonic anhydride (0.56 mL, 3.49 mmol, 1.5 equiv) was added quickly dropwise to a stirred solution of **S4** (614 mg, 2.33 mmol) and pyridine (1.0 mL, 6.99 mmol, 3 equiv) in dry CH_2Cl_2 (10 mL), cooled in ice-water bath. The resulting yellowish solution was allowed to warm up and left stirring at rt for 1 h. The mixture was then diluted with 0.5 N HCl (100 mL), extracted with CH_2Cl_2 (3×25 mL), the combined extracts were washed with brine (50 mL) and dried over Na_2SO_4. The product was isolated by flash column chromatography (12 g Interchim SiHP 30 µm cartridge, gradient 5% to 50% EtOAc/hexane) and freeze-dried from 1,4-dioxane to give 904 mg (98%) of **1ad** as white solid.

mp (EtOAc/hexane): 60-60.5 °C.
^{1}H NMR (400 MHz, $CDCl_3$): δ 7.37 – 7.30 (m, 3H), 7.13 (dd, *J* = 9.1, 3.1 Hz, 1H), 7.08 (tt, *J* = 8.4, 2.4 Hz, 1H), 7.02 (d, *J* = 3.1 Hz, 1H), 3.86 (s, 3H).
^{19}F NMR (376 MHz, $CDCl_3$): δ -73.22, -107.54.
^{13}C NMR (101 MHz, $CDCl_3$): δ 190.2 (t, *J* = 2.7 Hz), 164.3 (d, *J* = 11.7 Hz), 161.8 (d, *J* = 11.7 Hz), 158.9, 139.9, 139.4 (t, *J* = 7.9 Hz), 132.4, 124.0, 118.6 (q, *J* = 320.9 Hz), 118.2, 116.1, 113.1 (d, *J* = 11.5 Hz), 113.1 (d, *J* = 26.5 Hz), 109.3 (t, *J* = 25.4 Hz), 56.2.
HRMS ($C_{15}H_9F_5O_5S$): *m/z* (positive mode) = 397.0170 (found $[M+H]^+$), 397.0164 (calc.).

Preparation of 3-substituted benzothiophene 1,1-dioxides 3

2a

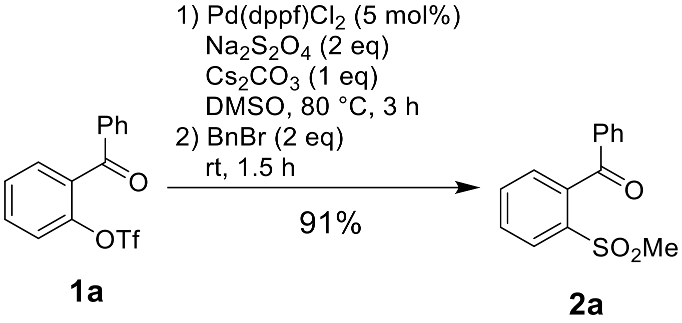

A 25 mL round-bottom flask was loaded with 2'-hydroxybenzophenone triflate **1a** (407 mg, 1.23 mmol; known compound[S7]), $Pd(dppf)Cl_2{\cdot}CH_2Cl_2$ catalyst (50 mg, 0.062 mmol, 5 mol%), sodium dithionite (428 mg, 2.46 mmol, 2 equiv.) and cesium carbonate (401 mg, 1.23 mmol, 1 equiv.). Dry DMSO solvent (5 mL) was added, the reaction mixture was degassed on a Schlenk line, placed in a preheated 80 °C silicon oil bath and stirred for 3 h. Upon cooling down to rt, iodomethane (150 µL, 2.46 mmol, 2 equiv.) was injected and the mixture was stirred at rt for further 1.5 h. The reaction mixture was diluted with brine (50 mL), extracted with CH_2Cl_2 (3×25 mL) and the combined extracts were dried over Na_2SO_4. The product was isolated by flash

column chromatography (12 g Interchim SiHP 30 µm cartridge, gradient 10% to 70% EtOAc/hexane) and freeze-dried from 1,4-dioxane to give 291 mg (91%) of **2a** as white solid. Known compound, the analytical data match those reported in [S8].

mp (EtOAc/hexane): 125-126 °C.

3a

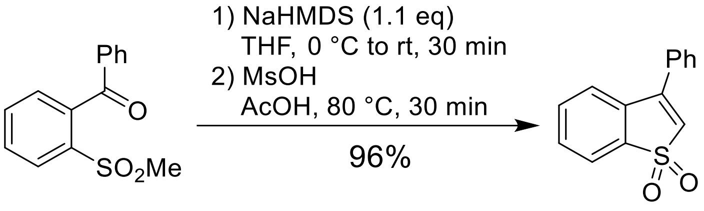

A solution of NaHMDS (0.28 mL of 2 M in THF, 0.6 mmol, 1.1 equiv.) was added dropwise to a stirred solution of **2a** (130 mg, 0.5 mmol; known compound[S8]) in dry THF (2.5 mL), cooled in ice-water bath. The reaction mixture was warmed up to rt and stirred for 30 min, then quenched by addition of acetic acid (0.5 mL) and evaporated. The residue was dissolved in acetic acid (2 mL), methanesulfonic acid (0.5 mL) was added and the mixture was stirred at 80 °C for 30 min. Upon cooling to rt, the mixture was diluted with CH_2Cl_2 and poured carefully into 50 mL of sat. aq. $NaHCO_3$. The aqueous layer was extracted with CH_2Cl_2 (3×20 mL), washed with brine and dried over Na_2SO_4. The product was isolated by flash column chromatography (12 g Interchim SiHP 30 µm cartridge, gradient 10% to 70% EtOAc/hexane) and dried *in vacuo* to give 116 mg (96%) of **3a** as white solid. Known compound, see [S9].

mp (EtOAc/hexane): 153-154 °C; lit.: 159-160 °C (EtOH[S9]).

^{1}H NMR (400 MHz, $CDCl_3$): δ 7.83 – 7.78 (m, 1H), 7.60 – 7.56 (m, 2H), 7.56 – 7.47 (m, 6H), 6.65 (s, 1H).
^{13}C NMR (101 MHz, $CDCl_3$): δ 146.1, 138.3, 133.4, 132.1, 131.2, 130.7, 130.6, 129.3, 128.1, 125.8, 124.4, 121.8.

2'a

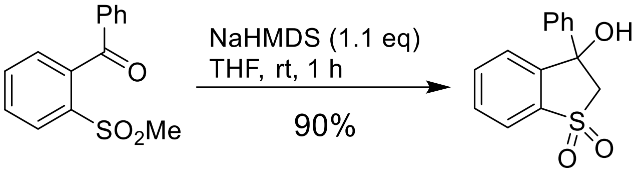

2a **2'a**

A solution of NaHMDS (0.21 mL of 2 M in THF, 0.424 mmol, 1.1 equiv.) was added dropwise to a stirred solution of **2a** (100 mg, 0.386 mmol; known compound[S8]) in dry THF (3 mL) at rt. The resulting light orange solution was stirred at rt for 1 h and then quenched by addition of acetic acid (0.1 mL). The mixture was diluted with brine (50 mL), the product was extracted with EtOAc (3×25 mL) and the combined extracts were dried over Na_2SO_4. The product was isolated by flash column chromatography (12 g Interchim SiHP 30 µm cartridge, gradient 0% to 20% EtOAc/CH_2Cl_2) and dried *in vacuo* to give 90 mg (90%) of **2'a** as white solid. Known compound, see [S9].

^{1}H NMR (400 MHz, $CDCl_3$): δ 7.82 – 7.76 (m, 1H), 7.65 – 7.58 (m, 2H), 7.49 – 7.44 (m, 2H), 7.43 – 7.33 (m, 3H), 7.28 – 7.21 (m, 1H), 3.90 – 3.80 (m, 2H), 3.30 (s, 1H).
^{13}C NMR (101 MHz, $CDCl_3$): δ 143.4, 142.0, 139.0, 134.6, 131.0, 128.9, 128.5, 126.6, 125.8, 121.2, 66.6.

2b

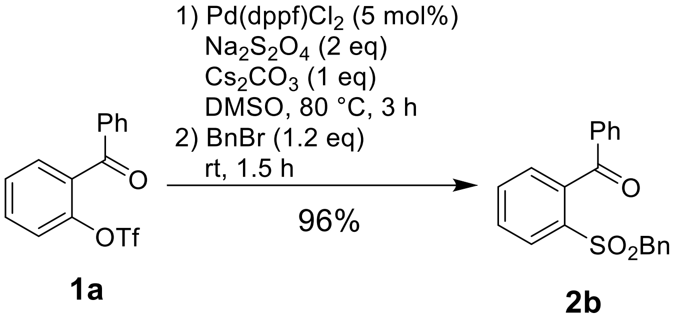

1a **2b**

A 25 mL round-bottom flask was loaded with 2'-hydroxybenzophenone triflate **1a** (415 mg, 1.26 mmol; known compound[S7]), Pd(dppf)Cl_2·CH_2Cl_2 catalyst (51 mg, 0.063 mmol, 5 mol%), sodium dithionite (438 mg, 2.52 mmol, 2 equiv.) and cesium carbonate (411 mg, 1.26 mmol, 1 equiv.). Dry DMSO solvent (5 mL) was added, the reaction mixture was degassed on a Schlenk line, placed in a preheated 80 °C silicon oil bath and stirred for 3 h. Upon cooling down to rt, benzyl bromide (180 µL, 1.51 mmol, 1.1 equiv.) was injected and the mixture was stirred at rt for further 1.5 h. The reaction mixture was diluted with brine (50 mL), extracted with CH_2Cl_2 (3×25 mL) and

the combined extracts were dried over Na_2SO_4. The product was isolated by flash column chromatography (12 g Interchim SiHP 30 µm cartridge, gradient 10% to 70% EtOAc/hexane) and dried *in vacuo* to give 408 mg (96%) of **2b** as viscous colorless oil.

^{1}H NMR (400 MHz, $CDCl_3$): δ 7.87 – 7.82 (m, 2H), 7.61 (td, *J* = 7.4, 1.3 Hz, 2H), 7.53 (ddd, *J* = 8.0, 1.3, 0.5 Hz, 1H), 7.50 – 7.45 (m, 2H), 7.43 – 7.36 (m, 2H), 7.32 – 7.21 (m, 5H), 4.65 (s, 2H).
^{13}C NMR (101 MHz, $CDCl_3$): δ 196.7, 140.6, 137.0, 136.6, 134.1, 132.9, 131.6, 131.2, 130.7, 129.5, 128.8, 128.70, 128.65, 128.4, 128.3, 64.2.
HRMS ($C_{20}H_{16}O_3S$): *m/z* (positive mode) = 337.0896 (found $[M+H]^+$), 337.0893 (calc.).

3b

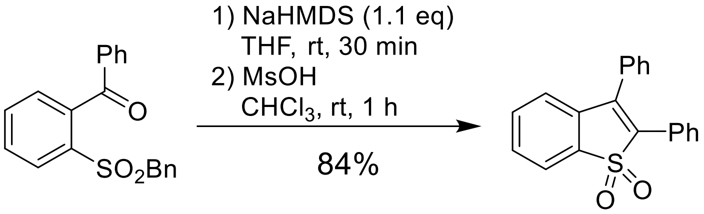

2b **3b**

A solution of NaHMDS (0.32 mL of 2 M in THF, 0.64 mmol, 1.1 equiv.) was added dropwise to a stirred solution of **2b** (196 mg, 0.59 mmol) in dry THF (3 mL) at rt. The reaction mixture was stirred for 30 min, then quenched by addition of acetic acid (0.5 mL) and evaporated. The residue was dissolved in chloroform (2 mL), methanesulfonic acid (0.59 mL) was added and the mixture was stirred at rt for 1 h. It was then carefully poured into 50 mL of sat. aq. $NaHCO_3$, extracted with CH_2Cl_2 (3×25 mL), washed with brine and dried over Na_2SO_4. The product was isolated by flash column chromatography (12 g Interchim SiHP 30 µm cartridge, gradient 5% to 60% EtOAc/hexane) and freeze-dried from 1,4-dioxane to give 157 mg (84%) of **3b** as white solid. Known compound, see [S10].

mp (EtOAc/hexane): 165-166 °C (lit. 158-159 °C [S10]).
^{1}H NMR (400 MHz, $CDCl_3$): δ 7.88 – 7.82 (m, 1H), 7.57 – 7.51 (m, 2H), 7.50 – 7.42 (m, 5H), 7.36 – 7.26 (m, 6H).
^{13}C NMR (101 MHz, $CDCl_3$): δ 138.2, 136.5, 133.6, 133.3, 131.0, 130.2, 129.8, 129.6, 129.4, 129.3, 129.2, 128.9, 127.2, 124.3, 121.7.

3c

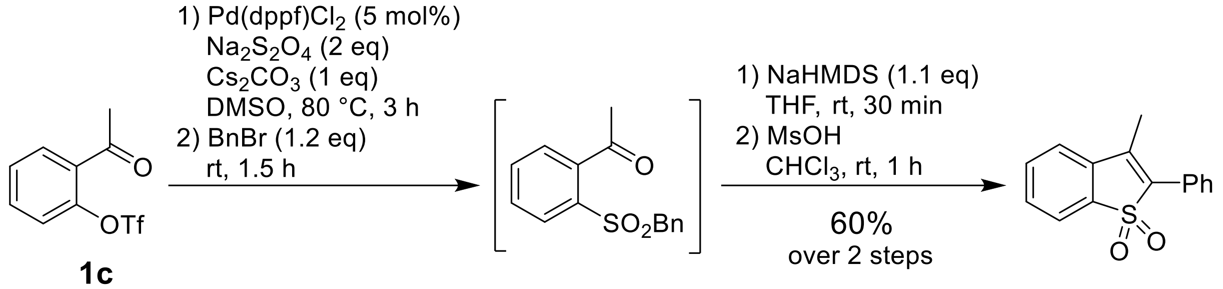

1c

3c

A 25 mL round-bottom flask was loaded with compound 2'-hydroxyacetophenone triflate **1c** (268 mg, 1 mmol; known compound [S11]), Pd(dppf)$Cl_2 \cdot CH_2Cl_2$ catalyst (40 mg, 0.05 mmol, 5 mol%), sodium dithionite (348 mg, 2 mmol, 2 equiv.) and cesium carbonate (326 mg, 1 mmol, 1 equiv.). Dry DMSO solvent (4 mL) was added, the reaction mixture was degassed on a Schlenk line, placed in a preheated 80 °C silicon oil bath and stirred for 3 h. Upon cooling down to rt, benzyl bromide (143 µL, 1.2 mmol, 1.2 equiv.) was injected and the mixture was stirred at rt for further 1.5 h. The reaction mixture was diluted with brine (50 mL), extracted with CH_2Cl_2 (3×20 mL) and the combined extracts were dried over Na_2SO_4. The crude intermediate (mixture of expected ketone and aldol condensation products) was isolated by flash column chromatography (12 g Interchim SiHP 30 µm cartridge, gradient 10% to 80% EtOAc/hexane) and used directly in the next step.

A solution of NaHMDS (0.55 mL of 2 M in THF, 1.1 mmol, ~1.1 equiv.) was added dropwise to a stirred solution of the crude sulfone from the previous step in dry THF (5 mL) at rt. The reaction mixture was stirred for 30 min at rt, then quenched by addition of acetic acid (0.5 mL) and evaporated. The residue was dissolved in chloroform (4 mL), methanesulfonic acid (1 mL) was added and the mixture was stirred at rt for 1 h. It was then carefully poured into 50 mL of sat. aq. $NaHCO_3$, extracted with CH_2Cl_2 (3×25 mL), washed with brine and dried over Na_2SO_4. The product was isolated by flash column chromatography (12 g Interchim SiHP 30 µm cartridge, gradient 10% to 60% EtOAc/hexane) and dried *in vacuo* to give 154 mg (60% over 2 steps) of **3c** as light cream-colored solid. Known compound, see [S12].

mp (EtOAc/hexane): 147-148 °C.
^{1}H NMR (400 MHz, $CDCl_3$): δ 7.81 – 7.74 (m, 1H), 7.67 – 7.60 (m, 3H), 7.56 – 7.45 (m, 5H), 2.28 (s, 3H).
^{13}C NMR (101 MHz, $CDCl_3$): δ 137.7, 136.3, 135.5, 133.7, 133.6, 130.1, 129.9, 129.6, 129.2, 127.3, 122.4, 121.2, 12.2.
HRMS ($C_{15}H_{15}O_2S$): *m/z* (positive mode) = 257.0630 (found $[M+H]^+$), 257.0631 (calc.).

3d

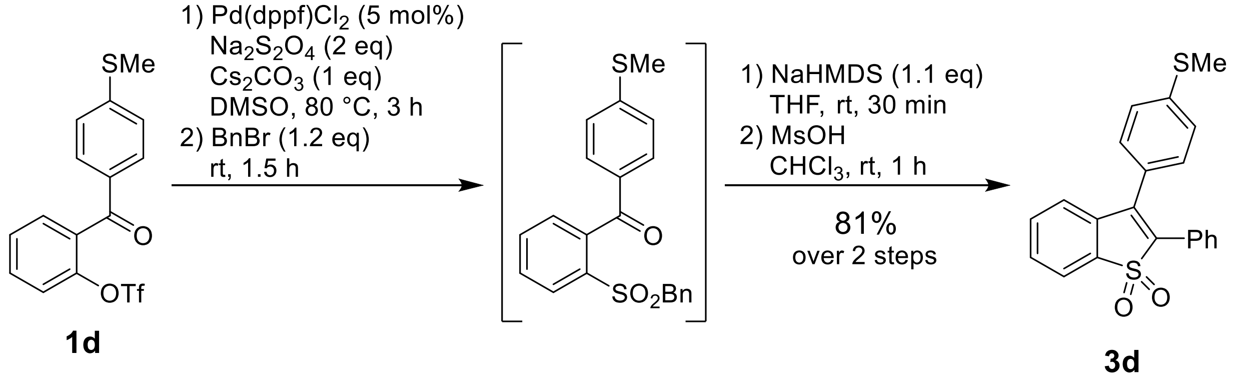

1d **3d**

A 10 mL microwave vial (Biotage) was loaded with compound **1d** (168 mg, 0.5 mmol), $Pd(dppf)Cl_2 \cdot CH_2Cl_2$ catalyst (20 mg, 0.025 mmol, 5 mol%), sodium dithionite (174 mg, 1 mmol, 2 equiv.) and cesium carbonate (163 mg, 0.5 mmol, 1 equiv.). Dry DMSO solvent (2 mL) was added, the reaction mixture was degassed on a Schlenk line, placed in a preheated 80 °C silicon oil bath and stirred for 3 h. Upon cooling down to rt, benzyl bromide (71 µL, 0.6 mmol, 1.2 equiv.) was injected and the mixture was stirred at rt for further 1.5 h. The reaction mixture was diluted with brine (50 mL), extracted with CH_2Cl_2 (3×20 mL) and the combined extracts were dried over Na_2SO_4. The crude intermediate was isolated by flash column chromatography (12 g Interchim SiHP 30 µm cartridge, gradient 10% to 80% EtOAc/hexane) and used directly in the next step.

A solution of NaHMDS (0.28 mL of 2 M in THF, 0.56 mmol, ~1.1 equiv.) was added dropwise to a stirred solution of the crude sulfone from the previous step in dry THF (2.5 mL) at rt. The reaction mixture was stirred for 30 min at rt, then quenched by addition of methanol (0.5 mL) and evaporated. The residue was dissolved in chloroform (2 mL), methanesulfonic acid (0.5 mL) was added and the mixture was stirred at rt for 1 h. It was then carefully poured into 50 mL of sat. aq. $NaHCO_3$, extracted with CH_2Cl_2 (3×25 mL), washed with brine and dried over Na_2SO_4. The product was isolated by flash column chromatography (12 g Interchim SiHP 30 µm cartridge, gradient 5% to 60% EtOAc/hexane) and freeze-dried from 1,4-dioxane to give 148 mg (81% over 2 steps) of **3d** as yellowish solid.

mp (EtOAc/hexane): 198-199 °C.
^1H NMR (400 MHz, $CDCl_3$): δ 7.87 – 7.82 (m, 1H), 7.57 – 7.51 (m, 2H), 7.51 – 7.46 (m, 2H), 7.35 – 7.22 (m, 8H), 2.51 (s, 3H).
^{13}C NMR (101 MHz, $CDCl_3$): δ 141.1, 137.7, 137.3, 136.5, 133.6, 133.2, 130.2, 129.8, 129.6, 129.5, 129.0, 127.2, 127.0, 126.4, 124.2, 121.7, 15.2.
HRMS ($C_{21}H_{16}O_2S_2$): *m/z* (positive mode) = 387.0487 (found $[M+Na]^+$), 387.0484 (calc.).

2e

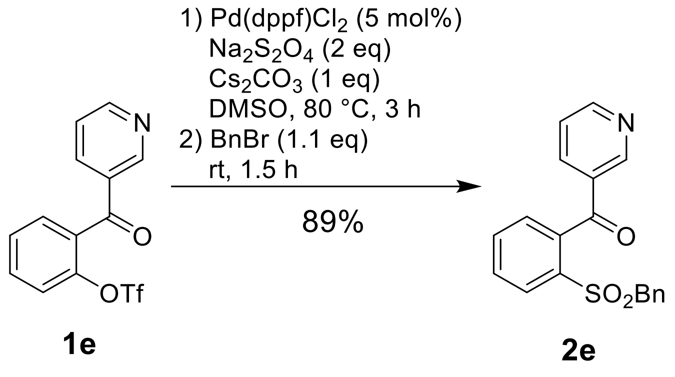

A 10 mL microwave vial (Biotage) was loaded with aryl triflate **1e** (167 mg, 0.5 mmol; known compound[S11]), Pd(dppf)Cl_2·CH_2Cl_2 catalyst (20 mg, 0.025 mmol, 5 mol%), sodium dithionite (174 mg, 1 mmol, 2 equiv.) and cesium carbonate (163 mg, 0.5 mmol, 1 equiv.). Dry DMSO solvent (2 mL) was added, the reaction mixture was degassed on a Schlenk line, placed in a preheated 80 °C silicon oil bath and stirred for 3 h. Upon cooling down to rt, benzyl bromide (65 µL, 0.55 mmol, 1.1 equiv.) was injected and the mixture was stirred at rt for further 1.5 h. The reaction mixture was diluted with brine (50 mL), extracted with CH_2Cl_2 (3×20 mL) and the combined extracts were dried over Na_2SO_4. The product was isolated by flash column chromatography (12 g Interchim SiHP 30 µm cartridge, gradient 30% to 100% EtOAc/hexane) and dried *in vacuo* to give 150 mg (89%) of **2e** as viscous brownish oil.

^{1}H NMR (400 MHz, $CDCl_3$): δ 9.00 (dd, *J* = 2.2, 0.9 Hz, 1H), 8.82 (dd, *J* = 4.9, 1.7 Hz, 1H), 8.15 (dt, *J* = 8.0, 2.0 Hz, 1H), 7.66 (td, *J* = 7.5, 1.3 Hz, 1H), 7.56 (dd, *J* = 8.0, 1.3 Hz, 1H), 7.48 – 7.42 (m, 2H), 7.39 (dd, *J* = 7.6, 1.2 Hz, 1H), 7.33 – 7.27 (m, 1H), 7.27 – 7.23 (m, 4H), 4.63 (s, 2H).
^{13}C NMR (101 MHz, $CDCl_3$): δ 195.6, 154.2, 151.8, 139.4, 137.5, 137.2, 133.2, 132.1, 131.8, 131.1, 130.0, 129.0, 128.7, 128.1, 127.9, 123.7, 64.3.
HRMS ($C_{19}H_{15}NO_3S$): *m/z* (positive mode) = 338.0844 (found $[M+H]^+$), 338.0845 (calc.).

3e

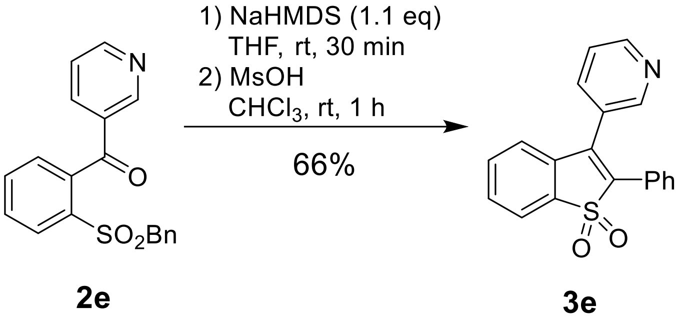

A solution of NaHMDS (0.24 mL of 2 M in THF, 0.48 mmol, ~1.1 equiv.) was added dropwise to a stirred solution of **2e** (150 mg, 0.44 mmol) in dry THF (2.5 mL) at rt. The reaction mixture was stirred for 30 min at rt, then quenched by addition of methanol (0.5 mL) and evaporated. The residue was dissolved in chloroform (2 mL), methanesulfonic acid (0.45 mL) was added and the mixture was stirred at rt for 1 h. It was then carefully poured into 50 mL of sat. aq. $NaHCO_3$, extracted with CH_2Cl_2 (3×25 mL), washed with brine and dried over Na_2SO_4. The product was isolated by flash column chromatography (12 g Interchim SiHP 30 µm cartridge, gradient 50% to 100% EtOAc/hexane) and freeze-dried from 1,4-dioxane to give 93 mg (66%) of **3e** as light tan solid.

mp (EtOAc/hexane): 173-174 °C.
^{1}H NMR (400 MHz, $CDCl_3$): δ 8.70 (dd, *J* = 4.9, 1.7 Hz, 1H), 8.62 (dd, *J* = 2.3, 0.9 Hz, 1H), 7.91 – 7.85 (m, 1H), 7.67 (ddd, *J* = 7.8, 2.3, 1.7 Hz, 1H), 7.61 – 7.55 (m, 2H), 7.49 – 7.44 (m, 2H), 7.39 (ddd, *J* = 8.0, 4.9, 1.0 Hz, 1H), 7.37 – 7.30 (m, 3H), 7.29 – 7.24 (m, 1H).
^{13}C NMR (101 MHz, $CDCl_3$): δ 150.7, 149.9, 139.4, 136.9, 136.3, 134.9, 133.8, 132.5, 130.6, 130.3, 129.5, 129.2, 127.3, 126.5, 123.9, 123.8, 122.1.
HRMS ($C_{19}H_{13}NO_2S$): *m/z* (positive mode) = 320.0738 (found $[M+H]^+$), 320.0740 (calc.).

2f

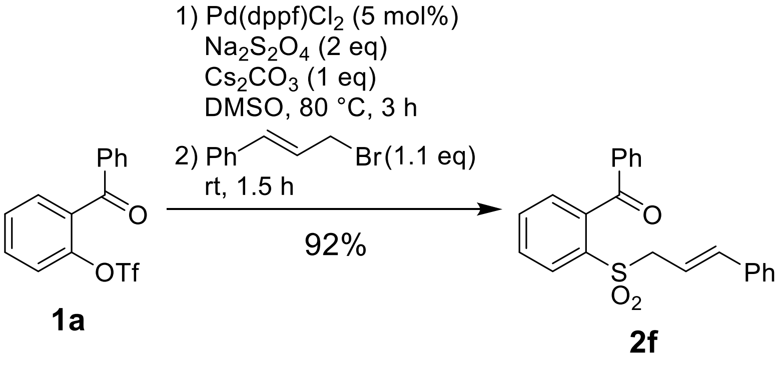

A 10 mL microwave vial (Biotage) was loaded with compound 2'-hydroxybenzophenone triflate **1a** (165 mg, 0.5 mmol; known compound[S7]), Pd(dppf)Cl_2·CH_2Cl_2 catalyst (20 mg, 0.025 mmol, 5 mol%), sodium dithionite (174 mg, 1 mmol, 2 equiv.) and cesium carbonate (163 mg, 0.5 mmol, 1 equiv.). Dry DMSO solvent (2 mL) was added, the reaction mixture was degassed on a Schlenk line, placed in a preheated 80 °C silicon oil bath and stirred for 3 h. Upon cooling down to rt, a solution of cinnamyl bromide (108 µL, 0.55 mmol, 1.1 equiv.) in DMSO (0.5 mL) was injected and the mixture was stirred at rt for further 1.5 h. The reaction mixture was diluted with brine (50 mL), extracted with CH_2Cl_2 (3×20 mL) and the combined extracts were dried over Na_2SO_4. The product was isolated by flash column chromatography (12 g Interchim SiHP 30 µm cartridge, gradient 10% to 60% EtOAc/hexane) and freeze-dried from dioxane to give 180 mg (92%, considering ~8% of an impurity content) of **2f** as light-yellow solid, which was used directly in the following step.

^{1}H NMR (400 MHz, $CDCl_3$): δ 8.00 (dd, J = 7.9, 1.3 Hz, 1H), 7.87 – 7.80 (m, 2H), 7.66 (td, J = 7.5, 1.3 Hz, 1H), 7.63 – 7.55 (m, 2H), 7.47 (t, J = 7.8 Hz, 2H), 7.39 (dd, J = 7.5, 1.4 Hz, 1H), 7.33 – 7.23 (m, 5H), 6.55 (d, J = 15.8 Hz, 1H), 6.14 (dt, J = 15.8, 7.7 Hz, 1H), 4.28 (dd, J = 7.7, 1.2 Hz, 2H).
^{13}C NMR (101 MHz, $CDCl_3$): δ 196.6, 140.6, 139.6, 137.5, 136.5, 136.1, 134.1, 133.1, 131.6, 130.6, 129.8, 128.72, 128.69, 128.5, 128.4, 126.8, 115.2, 62.0.
HRMS ($C_{22}H_{18}O_3S$): *m/z* (positive mode) = 385.0871 (found [M+Na]$^+$), 385.0869 (calc.).

3f

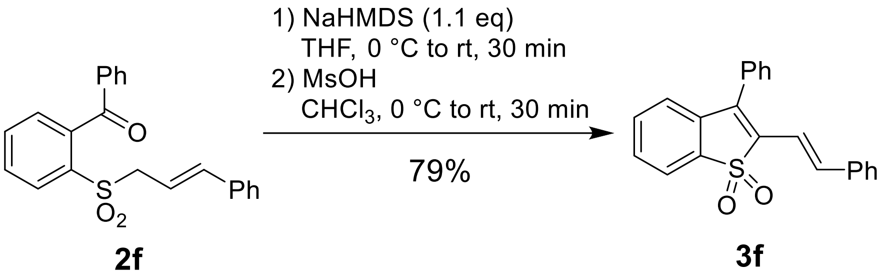

A solution of NaHMDS (0.28 mL of 2 M in THF, 0.56 mmol, ~1.1 equiv.) was added dropwise to a stirred solution of **2f** (180 mg, ~0.5 mmol) in dry THF (2.5 mL), cooled in ice-water bath. The reaction mixture was warmed up to rt and stirred for 30 min, then quenched by addition of methanol (0.5 mL) and evaporated. The residue was dissolved in chloroform (2 mL), cooled in ice-water, methanesulfonic acid (0.5 mL) was added and the mixture was stirred for 30 min at rt. It was then carefully poured into 50 mL of sat. aq. $NaHCO_3$, extracted with CH_2Cl_2 (3×20 mL), washed with brine and dried over Na_2SO_4. The product was isolated by flash column

chromatography (12 g Interchim SiHP 30 µm cartridge, gradient 5% to 60% EtOAc/hexane) and freeze-dried from 1,4-dioxane to give 136 mg (79%) of **3f** as yellow solid.

mp (EtOAc/hexane): 171-172 °C.

^{1}H NMR (400 MHz, $CDCl_3$): δ 7.88 – 7.81 (m, 1H), 7.62 – 7.49 (m, 6H), 7.48 – 7.41 (m, 4H), 7.35 – 7.27 (m, 4H), 6.79 (d, *J* = 16.7 Hz, 1H).

^{13}C NMR (101 MHz, $CDCl_3$): δ 137.0, 136.8, 136.5, 136.3, 136.1, 133.7, 133.5, 130.8, 129.9, 129.4, 129.3, 129.2, 128.9, 127.4, 124.1, 121.6, 114.4.

HRMS ($C_{22}H_{16}O_2S$): *m/z* (positive mode) = 367.0765 (found $[M+Na]^+$), 367.0763 (calc.).

2g

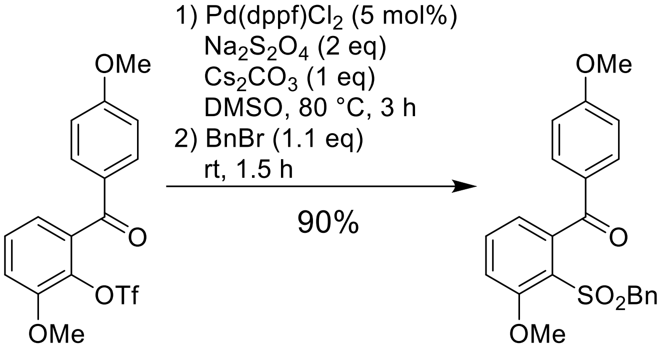

1g **2g**

A 10 mL microwave vial (Biotage) was loaded with compound **1g** (195 mg, 0.5 mmol), $Pd(dppf)Cl_2{\cdot}CH_2Cl_2$ catalyst (20 mg, 0.025 mmol, 5 mol%), sodium dithionite (174 mg, 1 mmol, 2 equiv.) and cesium carbonate (163 mg, 0.5 mmol, 1 equiv.). Dry DMSO solvent (2 mL) was added, the reaction mixture was degassed on a Schlenk line, placed in a preheated 80 °C silicon oil bath and stirred for 3 h. Upon cooling down to rt, benzyl bromide (65 µL, 0.55 mmol, 1.1 equiv.) was injected and the mixture was stirred at rt for further 1.5 h. The reaction mixture was diluted with brine (50 mL), extracted with CH_2Cl_2 (3×20 mL) and the combined extracts were dried over Na_2SO_4. The product was isolated by flash column chromatography (12 g Interchim SiHP 30 µm cartridge, gradient 20% to 100% EtOAc/hexane) and freeze-dried from 1,4-dioxane to give 178 mg (90%) of **2g** as white solid.

^{1}H NMR (400 MHz, $CDCl_3$): δ 7.59 – 7.50 (m, 3H), 7.34 – 7.28 (m, 1H), 7.28 – 7.22 (m, 2H), 7.22 – 7.18 (m, 2H), 7.10 (dd, *J* = 8.4, 1.0 Hz, 1H), 6.83 – 6.78 (m, 3H), 4.82 – 4.39 (m, 2H), 4.01 (s, 3H), 3.83 (s, 3H).

^{13}C NMR (101 MHz, $CDCl_3$): δ 193.5, 163.5, 158.2, 144.2, 135.0, 132.0, 131.2, 130.4, 128.8, 128.7, 127.8, 125.0, 120.4, 113.8, 112.9, 62.2, 56.9, 55.6.

HRMS ($C_{22}H_{20}O_5S$): *m/z* (positive mode) = 397.1104 (found $[M+H]^+$), 397.1104 (calc.).

3g

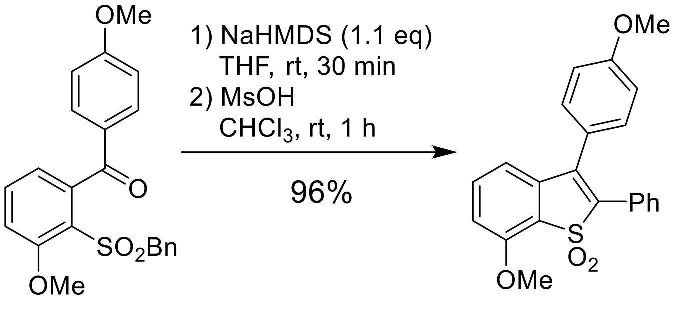

2g **3g**

A solution of NaHMDS (0.22 mL of 2 M in THF, 0.44 mmol, ~1.1 equiv.) was added dropwise to a stirred solution of **2g** (178 mg, 0.45 mmol) in dry THF (2.5 mL) at rt. The reaction mixture was stirred for 30 min, then quenched by addition of methanol (0.5 mL) and evaporated. The residue was dissolved in chloroform (2 mL), methanesulfonic acid (0.45 mL) was added and the mixture was stirred for 1 h at rt. It was then carefully poured into 50 mL of sat. aq. $NaHCO_3$, extracted with CH_2Cl_2 (3×20 mL), washed with brine and dried over Na_2SO_4. The product was isolated by flash column chromatography (12 g Interchim SiHP 30 µm cartridge, gradient 30% to 100% EtOAc/hexane) and dried *in vacuo* to give 164 mg (96%) of **3g** as white solid.

mp (EtOAc/hexane): 221-222 °C.
^{1}H NMR (400 MHz, $CDCl_3$): δ 7.51 – 7.44 (m, 3H), 7.33 – 7.27 (m, 3H), 7.26 – 7.21 (m, 2H), 7.02 (d, *J* = 8.4 Hz, 1H), 6.95 – 6.91 (m, 2H), 6.89 (d, *J* = 7.6 Hz, 1H), 4.04 (s, 3H), 3.84 (s, 3H).
^{13}C NMR (101 MHz, $CDCl_3$): δ 160.5, 155.7, 137.7, 137.3, 135.8, 135.3, 130.8, 129.6, 129.5, 128.8, 127.6, 123.2, 122.2, 116.4, 114.6, 113.5, 56.6, 55.5.
HRMS ($C_{22}H_{18}O_4S$): *m/z* (positive mode) = 401.0816 (found $[M+Na]^+$), 401.0818 (calc.).

3h

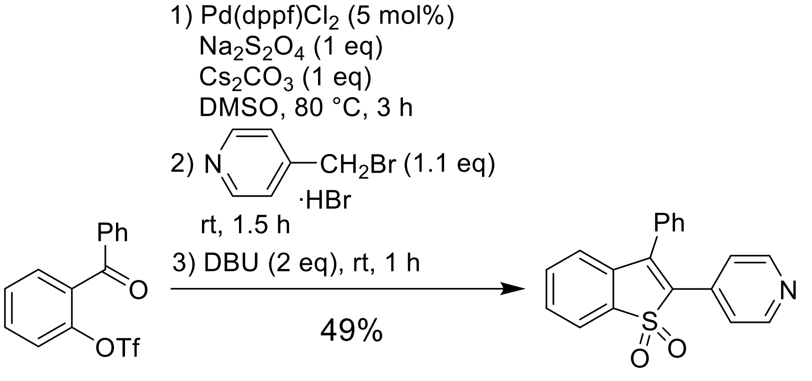

1a **3h**

A 10 mL microwave vial (Biotage) was loaded with 2'-hydroxybenzophenone triflate **1a** (165 mg, 0.5 mmol; known compound[S7]), Pd(dppf)Cl_2·CH_2Cl_2 catalyst (20 mg, 0.025 mmol, 5 mol%), sodium dithionite (87 mg, 0.5 mmol, 1 equiv.) and cesium carbonate (163 mg, 0.5 mmol, 1 equiv.). Dry DMSO solvent (2 mL) was added, the reaction mixture was degassed on a Schlenk line, placed in a preheated 80 °C silicon oil bath and stirred for 3 h. Upon cooling down to rt, a solution of 4-(bromomethyl)pyridine hydrobromide (139 mg, 0.55 mmol, 1.1 equiv.) in DMSO (0.5 mL) was injected and the mixture was stirred at rt for further 1.5 h. DBU (150 µL, 1 mmol, 2 equiv.) was then injected, and the brown-orange color of the reaction mixture quickly changed to dark brown. After stirring at rt for 1 h, the reaction mixture was evaporated on silica and the product was isolated by flash column chromatography (12 g Interchim SiHP 30 µm cartridge, gradient 30% to 100% EtOAc/hexane) and freeze-dried from 1,4-dioxane to give 78 mg (49%) of **3h** as light tan solid.

mp (EtOAc/hexane): 158-159 °C.
^{1}H NMR (400 MHz, $CDCl_3$): δ 8.59 – 8.54 (m, 2H), 7.91 – 7.85 (m, 1H), 7.64 – 7.56 (m, 2H), 7.55 – 7.46 (m, 3H), 7.37 – 7.30 (m, 5H).
^{13}C NMR (101 MHz, $CDCl_3$): δ 150.4, 141.3, 136.6, 135.2, 134.9, 133.9, 132.7, 131.0, 130.3, 130.2, 129.7, 128.9, 124.9, 123.1, 121.8.
HRMS ($C_{19}H_{13}NO_2S$): *m/z* (positive mode) = 320.0739 (found $[M+H]^+$), 320.0740 (calc.).

2i

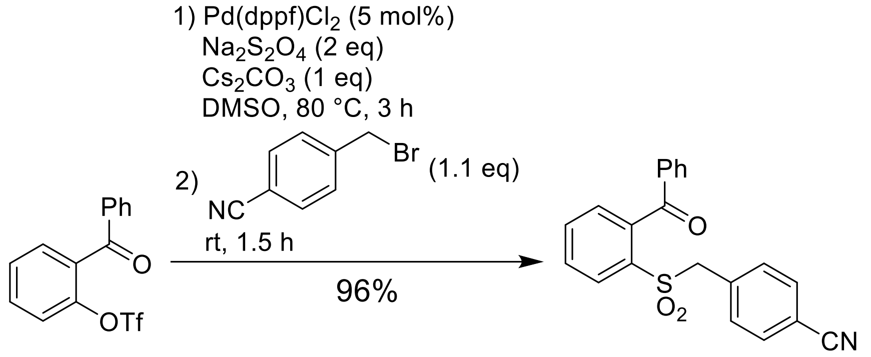

1a **2i**

A 10 mL microwave vial (Biotage) was loaded with 2'-hydroxybenzophenone triflate **1a** (165 mg, 0.5 mmol; known compound[S7]), Pd(dppf)Cl_2·CH_2Cl_2 catalyst (20 mg, 0.025 mmol, 5 mol%), sodium dithionite (174 mg, 1 mmol, 2 equiv.) and cesium carbonate (163 mg, 0.5 mmol, 1 equiv.). Dry DMSO solvent (2 mL) was added, the reaction mixture was degassed on a Schlenk line, placed in a preheated 80 °C silicon oil bath and stirred for 3 h. Upon cooling down to rt, a solution of 4-cyanobenzyl bromide (108 mg, 0.55 mmol, 1.1 equiv.) in DMSO (0.5 mL) was injected and the mixture was stirred at rt for further 1.5 h. The reaction mixture was diluted with brine (50 mL), extracted with CH_2Cl_2 (3×20 mL) and the combined extracts were dried over Na_2SO_4. The product was isolated by flash column chromatography (12 g Interchim SiHP 30 µm cartridge, gradient 20% to 80% EtOAc/hexane) and dried *in vacuo* to give 174 mg (96%) of **2i** as viscous yellowish material, which crystallizes very slowly.

^{1}H NMR (400 MHz, $CDCl_3$): δ 7.86 – 7.82 (m, 2H), 7.69 – 7.61 (m, 2H), 7.59 (dd, *J* = 8.0, 1.3 Hz, 1H), 7.57 – 7.54 (m, 2H), 7.52 – 7.45 (m, 3H), 7.44 – 7.40 (m, 3H), 4.74 (s, 2H).
^{13}C NMR (101 MHz, $CDCl_3$): δ 196.7, 140.6, 136.7, 136.3, 134.4, 133.7, 133.4, 132.4, 131.9, 131.4, 130.7, 129.8, 128.8, 128.6, 118.5, 112.9, 63.9.
HRMS ($C_{21}H_{15}NO_3S$): *m/z* (positive mode) = 362.0848 (found $[M+H]^+$), 362.0845 (calc.).

3i

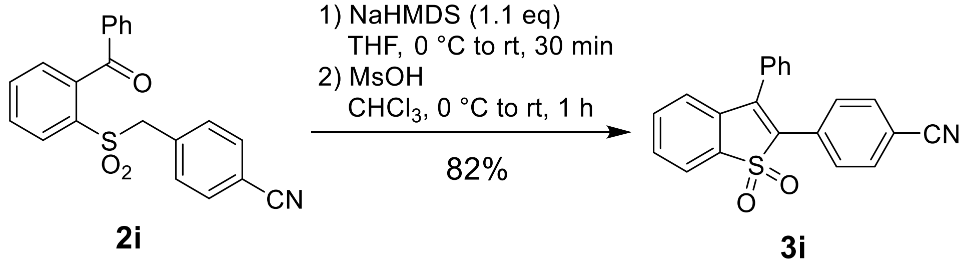

2i **3i**

A solution of NaHMDS (0.26 mL of 2 M in THF, 0.52 mmol, ~1.1 equiv.) was added dropwise to a stirred solution of **2i** (174 mg, 0.48 mmol) in dry THF (2.5 mL), cooled in ice-water bath. The reaction mixture was warmed up to rt and stirred for 30 min, then quenched by addition of methanol (0.5 mL) and evaporated. The residue was dissolved in chloroform (2 mL), cooled in ice-water, methanesulfonic acid (0.45 mL) was added and the mixture was stirred for 1 h at rt. It was then carefully poured into 50 mL of sat. aq. $NaHCO_3$, extracted with CH_2Cl_2 (3×20 mL), washed with brine and dried over Na_2SO_4. The product was isolated by flash column chromatography (12 g Interchim SiHP 30 µm cartridge, gradient 10% to 50% EtOAc/hexane) and freeze-dried from 1,4-dioxane to give 136 mg (82%) of **3i** as white solid.

mp (EtOAc/hexane): 212-213 °C.

^{1}H NMR (400 MHz, $CDCl_3$): δ 7.92 – 7.84 (m, 1H), 7.64 – 7.55 (m, 6H), 7.54 – 7.44 (m, 3H), 7.37 – 7.29 (m, 3H).

^{13}C NMR (101 MHz, $CDCl_3$): δ 140.6, 136.4, 135.7, 133.9, 132.7, 132.5, 132.1, 130.9, 130.3, 130.2, 129.9, 129.7, 129.0, 124.8, 121.9, 118.3, 113.2.

HRMS ($C_{21}H_{13}NO_2S$): *m/z* (positive mode) = 344.0741 (found $[M+H]^+$), 344.0740 (calc.).

3j

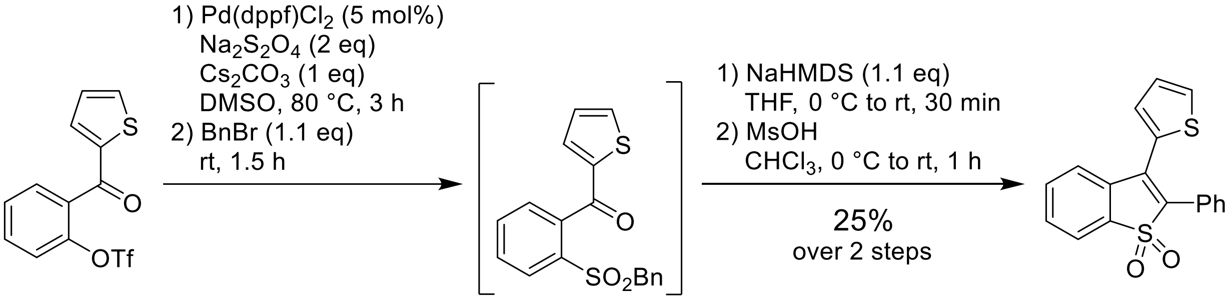

A 10 mL microwave vial (Biotage) was loaded with compound **1j** (168 mg, 0.5 mmol), $Pd(dppf)Cl_2{\cdot}CH_2Cl_2$ catalyst (20 mg, 0.025 mmol, 5 mol%), sodium dithionite (174 mg, 1 mmol, 2 equiv.) and cesium carbonate (163 mg, 0.5 mmol, 1 equiv.). Dry DMSO solvent (2 mL) was added, the reaction mixture was degassed on a Schlenk line, placed in a preheated 80 °C silicon oil bath and stirred for 3 h. Upon cooling down to rt, benzyl bromide (65 µL, 0.55 mmol, 1.1 equiv.) was injected and the mixture was stirred at rt for further 1.5 h. The reaction mixture was diluted with brine (50 mL), extracted with CH_2Cl_2 (3×20 mL) and the combined extracts were dried over Na_2SO_4. The crude intermediate (119 mg) was isolated by flash column chromatography (12 g Interchim SiHP 30 µm cartridge, gradient 10% to 60% EtOAc/hexane) and used directly in the next step.

A solution of NaHMDS (0.17 mL of 2 M in THF, 0.34 mmol, ~1.1 equiv.) was added dropwise to a stirred solution of the crude sulfone from the previous step in dry THF (2 mL), cooled in ice-water bath. The reaction mixture was stirred for 30 min at rt, then quenched by addition of methanol (0.5 mL) and evaporated. The residue was dissolved in chloroform (2 mL), cooled in ice-water bath, methanesulfonic acid (0.5 mL) was added and the mixture was stirred at rt for 1 h. It was then carefully poured into 50 mL of sat. aq. $NaHCO_3$, extracted with CH_2Cl_2 (3×20 mL), washed with brine and dried over Na_2SO_4. The product was isolated by flash column chromatography (12 g Interchim SiHP 30 µm cartridge, gradient 5% to 60% EtOAc/hexane) and freeze-dried from 1,4-dioxane to give 40 mg (25% over 2 steps) of **3j** as off-white solid.

mp (EtOAc/hexane): 181-182 °C.

^{1}H NMR (400 MHz, $CDCl_3$): δ 7.88 – 7.84 (m, 1H), 7.66 – 7.56 (m, 3H), 7.55 – 7.51 (m, 2H), 7.48 (dd, J = 5.1, 1.2 Hz, 1H), 7.43 – 7.34 (m, 3H), 7.21 (dd, J = 3.7, 1.2 Hz, 1H), 7.12 (dd, J = 5.1, 3.6 Hz, 1H).

^{13}C NMR (101 MHz, $CDCl_3$): δ 138.2, 136.3, 133.7, 132.8, 132.0, 131.1, 130.3, 130.14, 130.12, 129.9, 129.0, 128.9, 127.9, 127.0, 124.3, 121.8.

HRMS ($C_{18}H_{12}O_2S_2$): m/z (positive mode) = 325.0353 (found $[M+H]^+$), 325.0351 (calc.).

2k

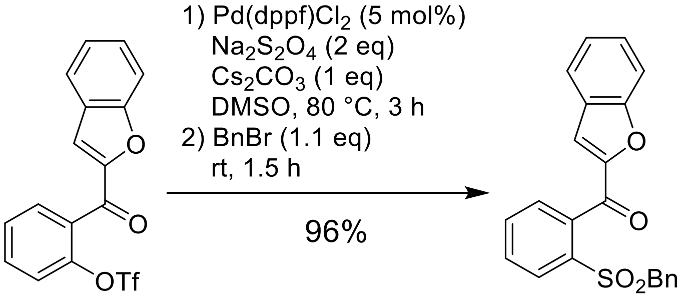

1k **2k**

A 10 mL microwave vial (Biotage) was loaded with compound **1k** (185 mg, 0.5 mmol), $Pd(dppf)Cl_2 \cdot CH_2Cl_2$ catalyst (20 mg, 0.025 mmol, 5 mol%), sodium dithionite (174 mg, 1 mmol, 2 equiv.) and cesium carbonate (163 mg, 0.5 mmol, 1 equiv.). Dry DMSO solvent (2 mL) was added, the reaction mixture was degassed on a Schlenk line, placed in a preheated 80 °C silicon oil bath and stirred for 3 h. Upon cooling down to rt, benzyl bromide (65 µL, 0.55 mmol, 1.1 equiv.) was injected and the mixture was stirred at rt for further 1.5 h. The reaction mixture was diluted with brine (50 mL), extracted with CH_2Cl_2 (3×20 mL) and the combined extracts were dried over Na_2SO_4. The product was isolated by flash column chromatography (12 g Interchim

SiHP 30 µm cartridge, gradient 10% to 60% EtOAc/hexane) and freeze-dried from 1,4-dioxane to give 180 mg (96%) of **2k** as light-yellow solid.

mp (EtOAc/hexane): 85-87 °C.

^{1}H NMR (400 MHz, $CDCl_3$): δ 7.71 – 7.66 (m, 2H), 7.65 – 7.57 (m, 3H), 7.55 – 7.45 (m, 2H), 7.35 – 7.23 (m, 7H), 4.71 (s, 2H).

^{13}C NMR (101 MHz, $CDCl_3$): δ 185.2, 156.7, 152.1, 138.8, 137.4, 133.1, 131.9, 131.2, 130.3, 129.3, 128.9, 128.7, 128.2, 127.1, 124.3, 123.8, 118.8, 112.9, 64.4.

HRMS ($C_{22}H_{16}O_4S$): *m/z* (positive mode) = 377.0840 (found $[M+H]^+$), 377.0842 (calc.).

3k

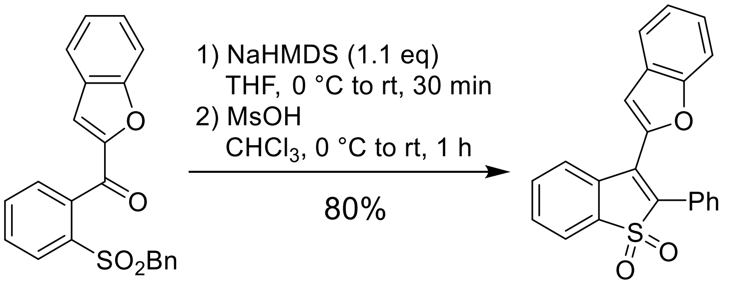

2k **3k**

A solution of NaHMDS (0.22 mL of 2 M in THF, 0.44 mmol, ~1.1 equiv.) was added dropwise to a stirred solution of **2k** (150 mg, 0.40 mmol) in dry THF (2.5 mL), cooled in ice-water bath. The reaction mixture was warmed up to rt and stirred for 30 min, then quenched by addition of acetic acid (0.5 mL) and evaporated. The residue was dissolved in chloroform (2 mL), cooled in ice-water, methanesulfonic acid (0.4 mL) was added and the mixture was stirred for 1 h at rt. It was then carefully poured into 50 mL of sat. aq. $NaHCO_3$, extracted with CH_2Cl_2 (3×20 mL), washed with brine and dried over Na_2SO_4. The product was isolated by flash column chromatography (12 g Interchim SiHP 30 µm cartridge, gradient 5% to 50% EtOAc/hexane) and freeze-dried from 1,4-dioxane to give 115 mg (80%) of **3k** as light-yellow solid.

mp (CH_2Cl_2/hexane): 194-195 °C.

^{1}H NMR (400 MHz, $CDCl_3$): δ 8.14 (dt, J = 7.8, 0.8 Hz, 1H), 7.88 (ddd, J = 7.4, 1.3, 0.6 Hz, 1H), 7.68 (td, J = 7.6, 1.3 Hz, 1H), 7.63 – 7.57 (m, 3H), 7.57 – 7.43 (m, 5H), 7.38 (ddd, J = 8.4, 7.2, 1.3 Hz, 1H), 7.30 – 7.25 (m, 1H), 6.79 (d, J = 1.0 Hz, 1H).

^{13}C NMR (101 MHz, $CDCl_3$): δ 155.1, 147.6, 138.8, 136.4, 133.9, 130.9, 130.5, 130.4, 129.7, 129.4, 127.6, 127.5, 127.2, 126.4, 125.7, 123.8, 122.01, 121.95, 111.7, 111.3.

HRMS ($C_{22}H_{14}O_3S$): *m/z* (positive mode) = 359.0729 (found $[M+H]^+$), 359.0736 (calc.).

2l

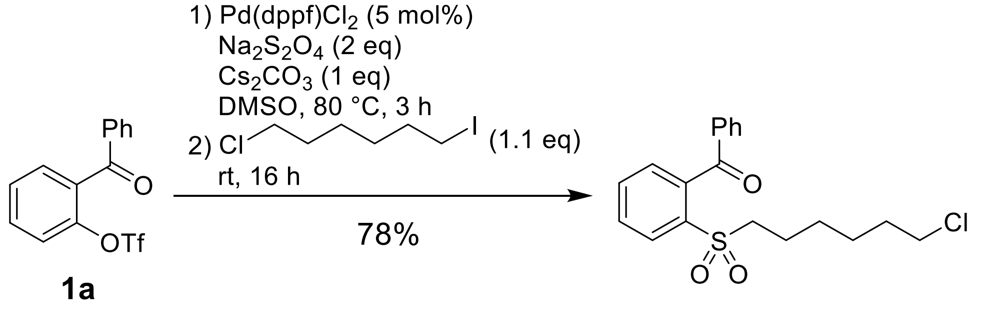

2l

A 25 mL round-bottom flask was loaded with 2'-hydroxybenzophenone triflate **1a** (330 mg, 1 mmol; known compound[S7]), Pd(dppf)Cl_2·CH_2Cl_2 catalyst (40 mg, 0.05 mmol, 5 mol%), sodium dithionite (348 mg, 1 mmol, 2 equiv.) and cesium carbonate (326 mg, 1 mmol, 1 equiv.). Dry DMSO solvent (4 mL) was added, the reaction mixture was degassed on a Schlenk line, placed in a preheated 80 °C silicon oil bath and stirred for 3 h. Upon cooling down to rt, 1-chloro-6-iodohexane (167 µL, 1.1 mmol, 1.1 equiv.) was injected and the mixture was stirred at rt overnight (16 h). The reaction mixture was diluted with brine (50 mL), extracted with CH_2Cl_2 (3×20 mL) and the combined extracts were dried over Na_2SO_4. The product was isolated by flash column chromatography (25 g Interchim SiHP 30 µm cartridge, gradient 10% to 50% EtOAc/hexane) and freeze-dried from 1,4-dioxane to give 285 mg (78%) of **2l** as white solid.

mp (EtOAc/hexane): 94-95 °C.

^{1}H NMR (400 MHz, $CDCl_3$): δ 8.13 – 8.07 (m, 1H), 7.83 – 7.77 (m, 2H), 7.73 – 7.65 (m, 2H), 7.63 – 7.57 (m, 1H), 7.50 – 7.43 (m, 2H), 7.42 – 7.35 (m, 1H), 3.51 (t, *J* = 6.6 Hz, 2H), 3.42 – 3.35 (m, 2H), 1.84 – 1.71 (m, 4H), 1.50 – 1.41 (m, 4H).

^{13}C NMR (101 MHz, $CDCl_3$): δ 196.5, 140.6, 138.3, 136.5, 134.1, 133.0, 130.8, 130.6, 130.1, 128.7, 128.5, 57.8, 44.9, 32.3, 27.7, 26.4, 22.4.

HRMS ($C_{19}H_{21}ClO_3S$): *m/z* (positive mode) = 387.0796 (found $[M+Na]^+$), 387.0792 (calc.).

3l

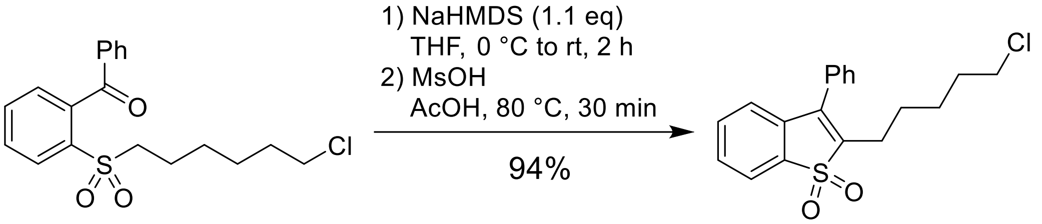

2l **3l**

A solution of NaHMDS (0.15 mL of 2 M in THF, 0.30 mmol, ~1.1 equiv.) was added dropwise to a stirred solution of **2l** (100 mg, 0.27 mmol) in dry THF (2 mL), cooled in ice-water bath. The reaction mixture was warmed up to rt and stirred for 2 h, then quenched by addition of acetic acid (0.5 mL) and evaporated. The residue was dissolved in acetic acid (2 mL), methanesulfonic acid (0.5 mL) was added and the mixture was stirred for 30 min at 80 °C. On cooling, it was diluted with CH_2Cl_2 and carefully poured into 50 mL of sat. aq. $NaHCO_3$, extracted with CH_2Cl_2 (3×20 mL), washed with brine and dried over Na_2SO_4. The product was isolated by flash column chromatography (12 g Interchim SiHP 30 µm cartridge, gradient 5% to 50% EtOAc/hexane) and freeze-dried from 1,4-dioxane to give 89 mg (94%) of **3l** as white solid.

mp (EtOAc/hexane): 64-65 °C.
^{1}H NMR (400 MHz, $CDCl_3$): δ 7.81 – 7.75 (m, 1H), 7.57 – 7.45 (m, 5H), 7.36 – 7.31 (m, 2H), 7.15 – 7.09 (m, 1H), 3.47 (t, J = 6.6 Hz, 2H), 2.62 – 2.54 (m, 2H), 1.82 – 1.67 (m, 4H), 1.49 – 1.40 (m, 2H).
^{13}C NMR (101 MHz, $CDCl_3$): δ 139.1, 138.9, 136.4, 133.62, 133.56, 130.8, 129.7, 129.6, 129.3, 128.6, 123.7, 121.4, 44.9, 32.1, 27.3, 26.8, 23.7.
HRMS ($C_{19}H_{19}ClO_2S$): *m/z* (positive mode) = 347.0868 (found $[M+H]^+$), 347.0867 (calc.).

2m

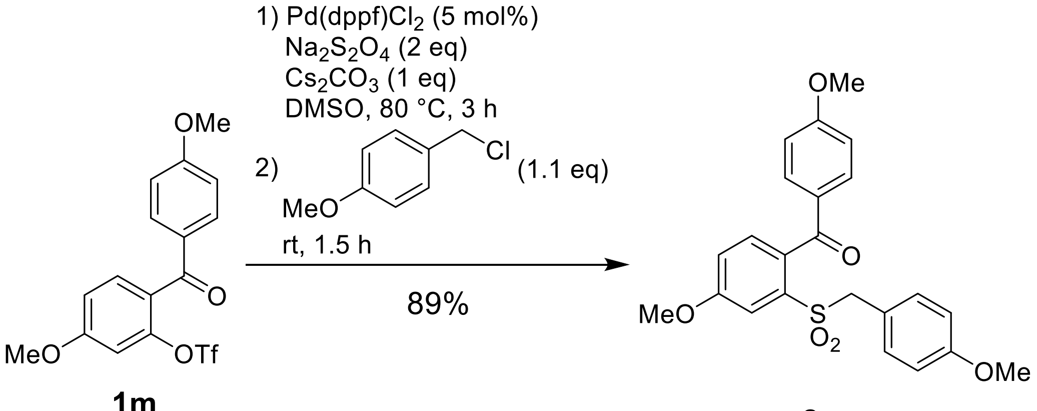

1m **2m**

A 10 mL microwave vial (Biotage) was loaded with compound **1m** (195 mg, 0.5 mmol), $Pd(dppf)Cl_2 \cdot CH_2Cl_2$ catalyst (20 mg, 0.025 mmol, 5 mol%), sodium dithionite (174 mg, 1 mmol, 2 equiv.) and cesium carbonate (163 mg, 0.5 mmol, 1 equiv.). Dry DMSO solvent (2 mL) was added, the reaction mixture was degassed on a Schlenk line, placed in a preheated 80 °C silicon oil bath and stirred for 3 h. Upon cooling down to rt, 4-methoxybenzyl chloride (74 µL, 0.55 mmol, 1.1 equiv.) was injected and the mixture was stirred at rt for further 1.5 h. The reaction mixture was diluted with brine (50 mL), extracted with CH_2Cl_2 (3×20 mL) and the combined extracts were dried over Na_2SO_4. The product was isolated by flash column chromatography (12 g Interchim SiHP 30 µm cartridge, gradient 20% to 80% EtOAc/hexane) and freeze-dried from 1,4-dioxane to give 189 mg (89%) of **2m** as white solid (purity ~90%).

^{1}H NMR (400 MHz, $CDCl_3$): δ 7.84 – 7.78 (m, 2H), 7.29 (d, J = 8.3 Hz, 1H), 7.23 – 7.18 (m, 2H), 7.07 (dd, J = 8.3, 2.6 Hz, 1H), 7.03 (d, J = 2.6 Hz, 1H), 6.97 – 6.91 (m, 2H), 6.82 – 6.76 (m, 2H), 3.87 (s, 3H), 3.77 (s, 3H), 3.70 (s, 3H).
^{13}C NMR (101 MHz, $CDCl_3$): δ 195.1, 164.3, 160.1, 159.9, 138.8, 133.0, 132.9, 132.5, 130.18, 130.06, 120.5, 119.0, 116.0, 114.1, 113.9, 63.5, 55.9, 55.7, 55.4.
HRMS ($C_{23}H_{22}O_6S$): m/z (positive mode) = 427.1209 (found $[M+H]^+$), 427.1210 (calc.).

3m

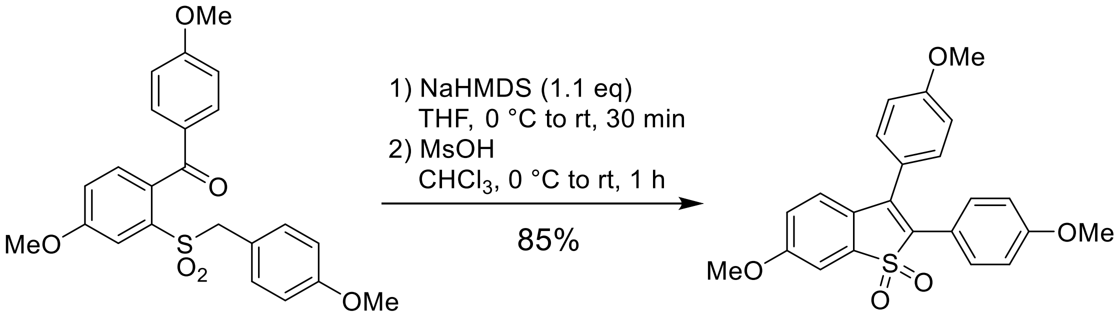

2m **3m**

A solution of NaHMDS (0.22 mL of 2 M in THF, 0.44 mmol, ~1.1 equiv.) was added dropwise to a stirred solution of **2m** (170 mg, 0.40 mmol) in dry THF (2 mL), cooled in ice-water bath. The reaction mixture was warmed up to rt and stirred for 30 min, then quenched by addition of acetic acid (0.5 mL) and evaporated. The residue was dissolved in chloroform (2 mL), cooled in ice-water, methanesulfonic acid (0.4 mL) was added and the mixture was stirred for 1 h at rt. It was then carefully poured into 50 mL of sat. aq. $NaHCO_3$, extracted with CH_2Cl_2 (3×20 mL), washed with brine and dried over Na_2SO_4. The product was isolated by flash column chromatography (12 g Interchim SiHP 30 µm cartridge, gradient 0% to 40% EtOAc/hexane + 20% CH_2Cl_2

constant additive) and freeze-dried from 1,4-dioxane to give 139 mg (85%) of **3m** as yellow solid.

mp (EtOAc/hexane): 189-190 °C.
^{1}H NMR (400 MHz, $CDCl_3$): δ 7.42 – 7.38 (m, 2H), 7.37 (d, *J* = 2.4 Hz, 1H), 7.29 – 7.24 (m, 2H), 7.22 (d, *J* = 8.5 Hz, 1H), 7.00 (dd, *J* = 8.5, 2.4 Hz, 1H), 6.98 – 6.93 (m, 2H), 6.84 – 6.80 (m, 2H), 3.90 (s, 3H), 3.85 (s, 3H), 3.79 (s, 3H).
^{13}C NMR (101 MHz, $CDCl_3$): δ 161.4, 160.44, 160.36, 138.1, 136.7, 135.1, 130.7, 125.9, 125.2, 123.4, 119.9, 119.1, 114.7, 114.4, 107.2, 56.2, 55.5, 55.4.
HRMS ($C_{23}H_{20}O_5S$): *m/z* (positive mode) = 431.0929 (found $[M+Na]^+$), 431.0924 (calc.).

2n

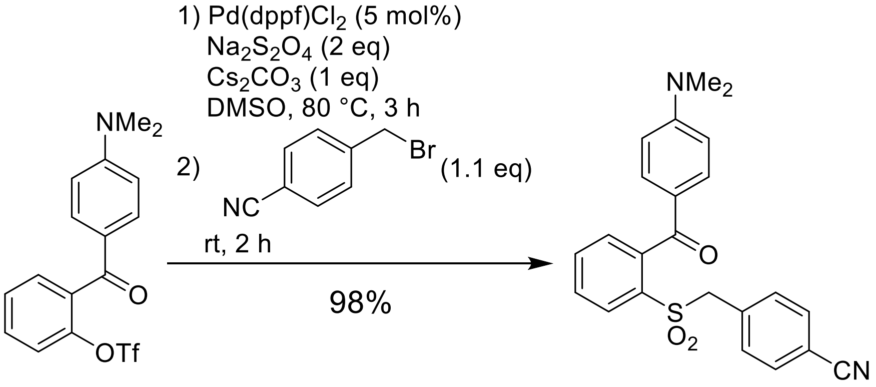

1n **2n**

A 10 mL microwave vial (Biotage) was loaded with compound **1n** (187 mg, 0.5 mmol), $Pd(dppf)Cl_2 \cdot CH_2Cl_2$ catalyst (20 mg, 0.025 mmol, 5 mol%), sodium dithionite (174 mg, 1 mmol, 2 equiv.) and cesium carbonate (163 mg, 0.5 mmol, 1 equiv.). Dry DMSO solvent (2 mL) was added, the reaction mixture was degassed on a Schlenk line, placed in a preheated 80 °C silicon oil bath and stirred for 3 h. Upon cooling down to rt, a solution of 4-cyanobenzyl bromide (108 mg, 0.55 mmol, 1.1 equiv.) in DMSO (0.5 mL) was injected and the mixture was stirred at rt for further 2 h. The reaction mixture was diluted with brine (50 mL), extracted with CH_2Cl_2 (3×20 mL) and the combined extracts were dried over Na_2SO_4. The product was isolated by flash column chromatography (12 g Interchim SiHP 30 µm cartridge, gradient 20% to 80% EtOAc/hexane) and freeze-dried from 1,4-dioxane to give 197 mg (98%) of **2n** as light-yellow solid.

^{1}H NMR (400 MHz, $CDCl_3$): δ 7.74 – 7.69 (m, 2H), 7.62 (td, *J* = 7.5, 1.3 Hz, 1H), 7.56 – 7.51 (m, 3H), 7.45 – 7.37 (m, 4H), 6.68 – 6.62 (m, 2H), 4.77 (s, 2H), 3.08 (s, 6H).

^{13}C NMR (101 MHz, $CDCl_3$): δ 194.3, 154.3, 141.6, 136.2, 134.1, 133.2, 133.1, 132.3, 131.9, 131.2, 129.1, 128.9, 124.2, 118.6, 112.6, 110.8, 63.6, 40.2.

HRMS ($C_{23}H_{20}N_2O_3S$): *m/z* (positive mode) = 405.1264 (found $[M+H]^+$), 405.1267 (calc.).

3n

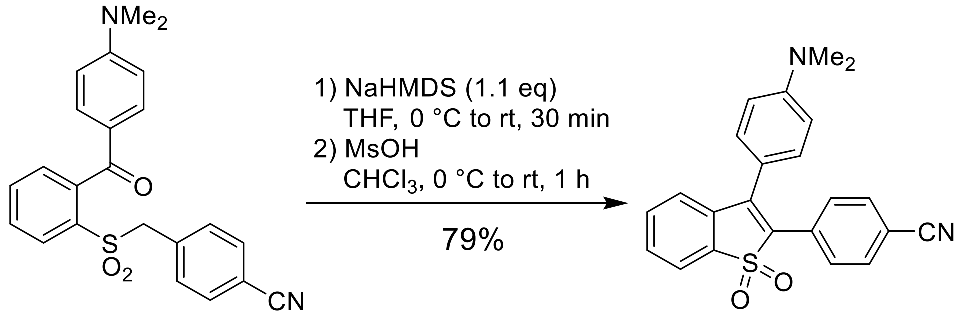

2n **3n**

A solution of NaHMDS (0.26 mL of 2 M in THF, 0.52 mmol, ~1.1 equiv.) was added dropwise to a stirred solution of **2n** (197 mg, 0.49 mmol) in dry THF (2.5 mL), cooled in ice-water bath. The reaction mixture was warmed up to rt and stirred for 30 min, then quenched by addition of acetic acid (0.5 mL) and evaporated. The residue was dissolved in chloroform (2 mL), cooled in ice-water, methanesulfonic acid (0.49 mL) was added and the mixture was stirred for 1 h at rt. It was then carefully poured into 50 mL of sat. aq. $NaHCO_3$, extracted with CH_2Cl_2 (3×20 mL), washed with brine and dried over Na_2SO_4. The product was isolated by flash column chromatography (12 g Interchim SiHP 30 µm cartridge, gradient 10% to 60% EtOAc/hexane) and freeze-dried from 1,4-dioxane to give 149 mg (79%) of **3n** as orange solid.

mp (CH_2Cl_2/hexane): 232-233 °C.

^{1}H NMR (400 MHz, $CDCl_3$): δ 7.88 – 7.82 (m, 1H), 7.65 – 7.56 (m, 6H), 7.54 – 7.50 (m, 1H), 7.20 – 7.14 (m, 2H), 6.74 – 6.68 (m, 2H), 3.04 (s, 6H).

^{13}C NMR (101 MHz, $CDCl_3$): δ 151.4, 141.2, 136.8, 133.6, 133.22, 133.16, 132.9, 132.5, 130.6, 130.5, 130.0, 125.0, 121.6, 118.6, 116.3, 112.6, 112.2, 40.2.

HRMS ($C_{23}H_{18}N_2O_2S$): *m/z* (positive mode) = 387.1160 (found $[M+H]^+$), 387.1162 (calc.).

2o

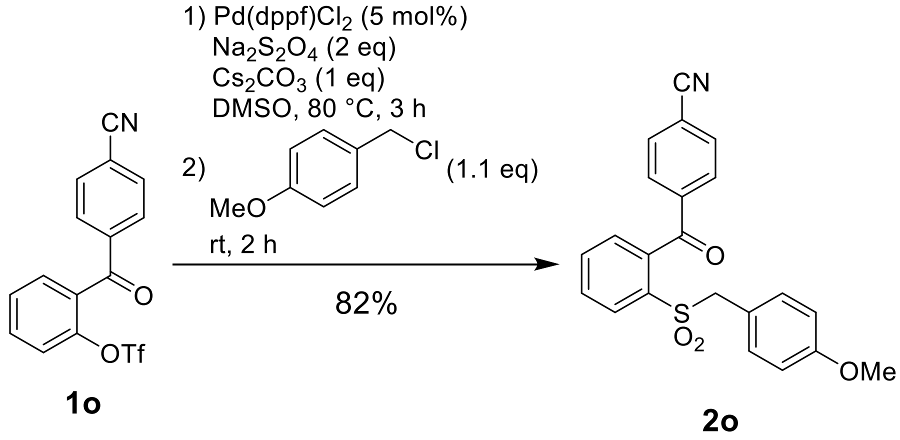

A 10 mL microwave vial (Biotage) was loaded with compound **1o** (178 mg, 0.5 mmol; known compound[S11]), Pd(dppf)Cl_2·CH_2Cl_2 catalyst (20 mg, 0.025 mmol, 5 mol%), sodium dithionite (174 mg, 1 mmol, 2 equiv.) and cesium carbonate (163 mg, 0.5 mmol, 1 equiv.). Dry DMSO solvent (2 mL) was added, the reaction mixture was degassed on a Schlenk line, placed in a preheated 80 °C silicon oil bath and stirred for 3 h. Upon cooling down to rt, 4-methoxybenzyl chloride (74 µL, 0.55 mmol, 1.1 equiv.) was injected and the mixture was stirred at rt for further 2 h. The reaction mixture was diluted with brine (50 mL), extracted with CH_2Cl_2 (3×20 mL) and the combined extracts were dried over Na_2SO_4. The product was isolated by flash column chromatography (12 g Interchim SiHP 30 µm cartridge, gradient 20% to 80% EtOAc/hexane) and freeze-dried from 1,4-dioxane to give 197 mg (98%) of **2o** as off-white solid (purity ~90%).

^{1}H NMR (400 MHz, $CDCl_3$): δ 7.94 – 7.90 (m, 2H), 7.79 – 7.75 (m, 2H), 7.66 (td, *J* = 7.5, 1.3 Hz, 1H), 7.57 (ddd, *J* = 8.0, 1.3, 0.5 Hz, 1H), 7.52 – 7.46 (m, 1H), 7.34 (ddd, *J* = 7.6, 1.3, 0.5 Hz, 1H), 7.17 – 7.12 (m, 2H), 6.81 – 6.76 (m, 2H), 4.53 (s, 2H), 3.78 (s, 3H).

^{13}C NMR (101 MHz, $CDCl_3$): δ 195.4, 160.2, 139.6, 139.4, 137.3, 133.2, 132.5, 132.3, 131.8, 130.8, 130.1, 127.7, 119.8, 118.0, 117.2, 114.2, 63.6, 55.4.

HRMS ($C_{22}H_{17}NO_4S$): *m/z* (positive mode) = 409.1213 (found $[M+NH_4]^+$), 409.1217 (calc.).

3o

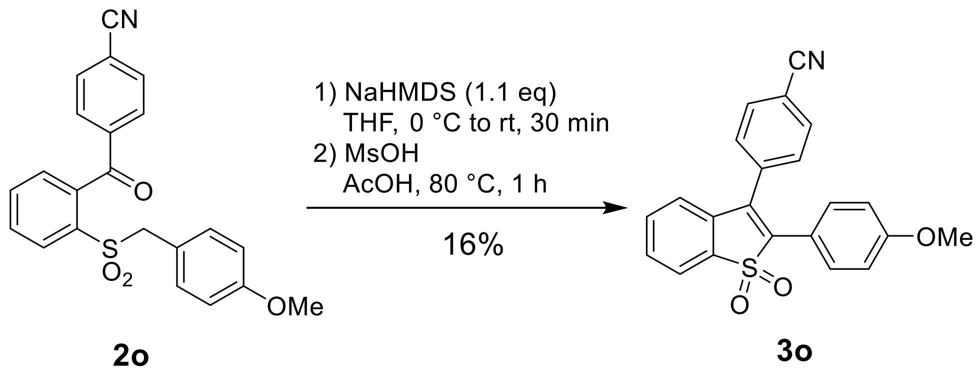

A solution of NaHMDS (0.23 mL of 2 M in THF, 0.46 mmol, ~1.1 equiv.) was added dropwise to a stirred solution of **2o** (160 mg, 0.41 mmol) in dry THF (2.5 mL), cooled in ice-water bath. The reaction mixture was warmed up to rt and stirred for 30 min, then quenched by addition of acetic acid (0.5 mL) and evaporated. The residue was dissolved in acetic acid (2 mL), methanesulfonic acid (0.5 mL) was added and the mixture was stirred at 80 °C for 1 h. It was then carefully poured into 70 mL of sat. aq. $NaHCO_3$, extracted with CH_2Cl_2 (3×20 mL), washed with brine and dried over Na_2SO_4. The product was isolated by flash column chromatography (12 g Interchim SiHP 30 µm cartridge, gradient 5% to 50% EtOAc/hexane + 20% CH_2Cl_2 constant additive) and freeze-dried from 1,4-dioxane to give 25 mg (16%) of **3o** as light yellow solid.

mp (CH_2Cl_2/hexane): 224-225 °C.
^{1}H NMR (400 MHz, $CDCl_3$): δ 7.89 – 7.83 (m, 1H), 7.79 – 7.74 (m, 2H), 7.59 – 7.53 (m, 2H), 7.51 – 7.46 (m, 2H), 7.40 – 7.34 (m, 2H), 7.20 – 7.15 (m, 1H), 6.87 – 6.81 (m, 2H), 3.80 (s, 3H).
^{13}C NMR (101 MHz, $CDCl_3$): δ 161.2, 139.0, 136.5, 136.1, 134.3, 133.8, 133.1, 132.5, 130.8, 130.3, 130.2, 123.5, 122.1, 118.4, 118.2, 114.8, 113.5, 55.5.
HRMS ($C_{22}H_{15}NO_3S$): *m/z* (positive mode) = 374.0846 (found $[M+H]^+$), 374.0845 (calc.).

3p

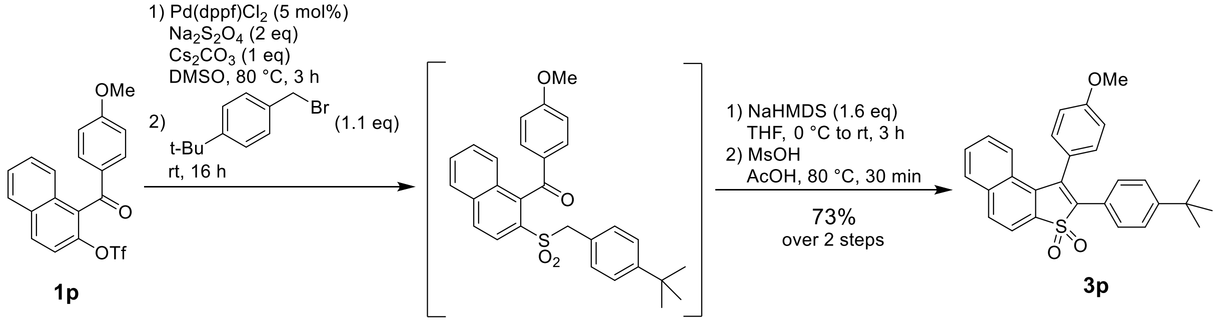

A 10 mL microwave vial (Biotage) was loaded with compound **1p** (205 mg, 0.5 mmol), $Pd(dppf)Cl_2 \cdot CH_2Cl_2$ catalyst (20 mg, 0.025 mmol, 5 mol%), sodium dithionite (174 mg, 1 mmol, 2 equiv.) and cesium carbonate (163 mg, 0.5 mmol, 1 equiv.). Dry DMSO solvent (2 mL) was added, the reaction mixture was degassed on a Schlenk line, placed in a preheated 80 °C silicon oil bath and stirred for 3 h. Upon cooling down to rt, 4-*tert*-butylbenzyl bromide (101 µL, 0.55 mmol, 1.1 equiv.) was injected and the mixture was stirred at rt overnight (16 h). The reaction mixture was diluted with brine (50 mL), extracted with CH_2Cl_2 (3×20 mL) and the combined extracts were dried over Na_2SO_4. The crude intermediate (239 mg) was isolated by flash column chromatography (12 g Interchim SiHP 30 µm cartridge, gradient 10% to 60% EtOAc/hexane) and used directly in the next step.

A solution of NaHMDS (0.38 mL of 2 M in THF, 0.76 mmol, ~1.6 equiv.) was added dropwise to a stirred solution of the crude sulfone from the previous step in dry THF (3 mL), cooled in ice-water bath. The reaction mixture was stirred for 3 h at rt, then quenched by addition of acetic acid (0.5 mL) and evaporated. The residue was dissolved in acetic acid (2 mL), methanesulfonic acid (0.5 mL) was added and the mixture was stirred at 80 °C for 30 min. It was then carefully poured into 50 mL of sat. aq. $NaHCO_3$, extracted with CH_2Cl_2 (3×20 mL), washed with brine and dried over Na_2SO_4. The product was isolated by flash column chromatography (12 g Interchim SiHP 30 µm cartridge, gradient 0% to 30% EtOAc/hexane + 20% CH_2Cl_2 constant additive) and freeze-dried from 1,4-dioxane to give 167 mg (73% over 2 steps) of **3p** as yellow solid.

mp (EtOAc/hexane): 210-210.5 °C.
^1H NMR (400 MHz, $CDCl_3$): δ 8.04 (d, J = 8.2 Hz, 1H), 7.92 – 7.87 (m, 2H), 7.50 (ddd, J = 8.1, 6.3, 1.7 Hz, 1H), 7.41 – 7.37 (m, 2H), 7.34 – 7.28 (m, 4H), 7.27 – 7.21 (m, 2H), 7.05 – 7.00 (m, 2H), 3.92 (s, 3H), 1.28 (s, 9H).
^{13}C NMR (101 MHz, $CDCl_3$): δ 160.4, 152.7, 138.6, 138.4, 137.3, 134.4, 131.9, 130.6, 129.6, 129.5, 129.0, 128.7, 127.9, 127.7, 126.3, 125.8, 125.6, 124.3, 116.8, 114.9, 55.5, 34.9, 31.2.
HRMS ($C_{29}H_{26}O_3S$): *m/z* (positive mode) = 472.1946 (found $[M+NH_4]^+$), 472.1941 (calc.).

3q

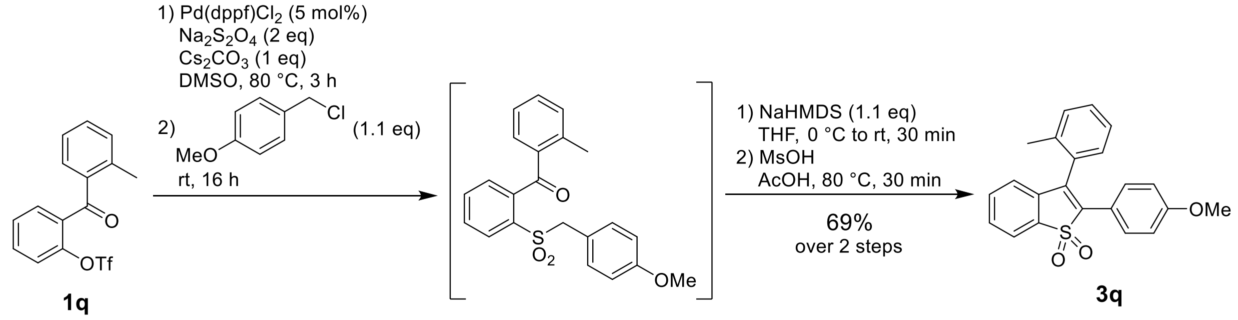

A 10 mL microwave vial (Biotage) was loaded with compound **1q** (172 mg, 0.5 mmol), $Pd(dppf)Cl_2{\cdot}CH_2Cl_2$ catalyst (20 mg, 0.025 mmol, 5 mol%), sodium dithionite (174 mg, 1 mmol, 2 equiv.) and cesium carbonate (163 mg, 0.5 mmol, 1 equiv.). Dry DMSO solvent (2 mL) was added, the reaction mixture was degassed on a Schlenk line, placed in a preheated 80 °C silicon oil bath and stirred for 3 h. Upon cooling down to rt, 4-methoxybenzyl chloride (74 µL, 0.55 mmol, 1.1 equiv.) was injected and the mixture was stirred at rt overnight (16 h). The reaction mixture was diluted with brine (50 mL), extracted with CH_2Cl_2 (3×20 mL) and the combined extracts were dried over Na_2SO_4. The crude intermediate (192 mg) was isolated by flash column chromatography (12 g Interchim SiHP 30 µm cartridge, gradient 10% to 60% EtOAc/hexane) and used directly in the next step.

A solution of NaHMDS (0.28 mL of 2 M in THF, 0.55 mmol, ~1.1 equiv.) was added dropwise to a stirred solution of the crude sulfone from the previous step in dry THF (2.5 mL), cooled in ice-water bath. The reaction mixture was stirred for 30 min at rt, then quenched by addition of acetic acid (0.5 mL) and evaporated. The residue was dissolved in acetic acid (2 mL), methanesulfonic acid (0.5 mL) was added and the mixture was stirred at 80 °C for 30 min. It was then carefully poured into 50 mL of sat. aq. $NaHCO_3$, extracted with CH_2Cl_2 (3×20 mL), washed with brine and dried over Na_2SO_4. The product was isolated by flash column chromatography (12 g Interchim SiHP 30 µm cartridge, gradient 5% to 60% EtOAc/hexane) and freeze-dried from 1,4-dioxane to give 125 mg (69% over 2 steps) of **3q** as light yellow solid.

mp (EtOAc/hexane): 127-128 °C.
^{1}H NMR (400 MHz, $CDCl_3$): δ 8.04 (d, *J* = 8.2 Hz, 1H), 7.92 – 7.87 (m, 2H), 7.50 (ddd, *J* = 8.1, 6.3, 1.7 Hz, 1H), 7.41 – 7.37 (m, 2H), 7.34 – 7.28 (m, 4H), 7.27 – 7.21 (m, 2H), 7.05 – 7.00 (m, 2H), 3.92 (s, 3H), 1.28 (s, 9H).
^{13}C NMR (101 MHz, $CDCl_3$): δ 160.4, 152.7, 138.6, 138.4, 137.3, 134.4, 131.9, 130.6, 129.6, 129.5, 129.0, 128.7, 127.9, 127.7, 126.3, 125.8, 125.6, 124.3, 116.8, 114.9, 55.5, 34.9, 31.2.
HRMS ($C_{22}H_{18}O_3S$): *m/z* (positive mode) = 385.0870 (found $[M+Na]^+$), 385.0869 (calc.).

3r

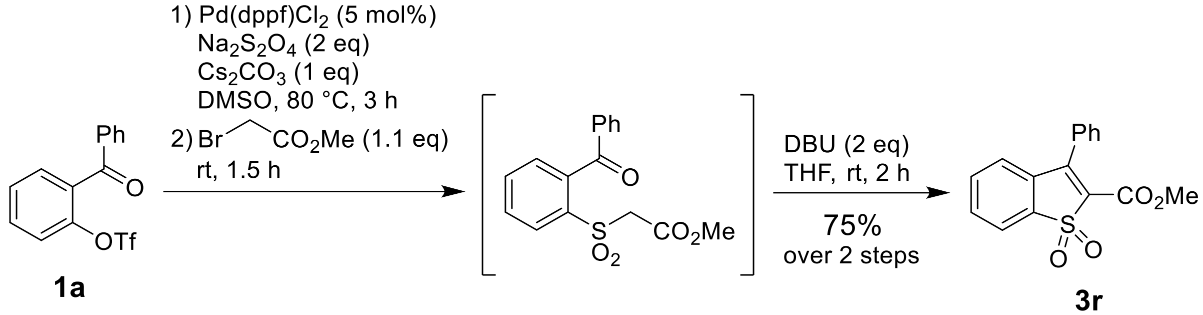

1a **3r**

A 10 mL microwave vial (Biotage) was loaded with 2'-hydroxybenzophenone triflate **1a** (198 mg, 0.6 mmol; known compound[S7]), Pd(dppf)Cl_2·CH_2Cl_2 catalyst (25 mg, 0.03 mmol, 5 mol%), sodium dithionite (209 mg, 1.2 mmol, 2 equiv.) and cesium carbonate (196 mg, 0.6 mmol, 1 equiv.). Dry DMSO solvent (2.4 mL) was added, the reaction mixture was degassed on a Schlenk line, placed in a preheated 80 °C silicon oil bath and stirred for 3 h. Upon cooling down to rt, methyl bromoacetate (63 µL, 0.66 mmol, 1.1 equiv.) was injected and the mixture was stirred at rt for 1.5 h. The reaction mixture was diluted with brine (50 mL), extracted with CH_2Cl_2 (3×20 mL) and the combined extracts were dried over Na_2SO_4. The solution was filtered through a short (3 cm) plug of silica, washing with additional EtOAc – CH_2Cl_2 (1:1), and the filtrate was evaporated. The crude intermediate was dissolved in dry THF (3 mL), DBU (0.18 mL, 1.2 mmol, 2 equiv.) was added, and the reaction mixture was stirred at rt for 2 h. It was then diluted with 0.2 N hydrochloric acid (50 mL), extracted with CH_2Cl_2 (3×25 mL), the combined extracts were washed with brine and dried over Na_2SO_4. The product was isolated by flash column chromatography (12 g Interchim SiHP 30 µm cartridge, gradient 10% to 70% EtOAc/hexane) and freeze-dried from 1,4-dioxane to give 135 mg (75% over 2 steps) of **3r** as off-white solid.

mp (EtOAc/hexane): 192-193 °C.

^{1}H NMR (400 MHz, $CDCl_3$): δ 7.86 (ddd, *J* = 7.6, 1.2, 0.6 Hz, 1H), 7.69 (td, *J* = 7.6, 1.0 Hz, 1H), 7.59 (td, *J* = 7.6, 1.1 Hz, 1H), 7.56 – 7.53 (m, 3H), 7.43 – 7.38 (m, 2H), 7.28 (dt, *J* = 7.7, 0.8 Hz, 1H), 3.87 (s, 3H).

^{13}C NMR (101 MHz, $CDCl_3$): δ 159.2, 153.0, 137.1, 133.8, 133.1, 131.4, 130.5, 129.7, 128.8, 128.7, 128.4, 126.6, 121.9, 52.9.

HRMS ($C_{16}H_{12}O_4S$): *m/z* (positive mode) = 324.0385 (found [M+Na]$^+$), 324.0381 (calc.).

3s

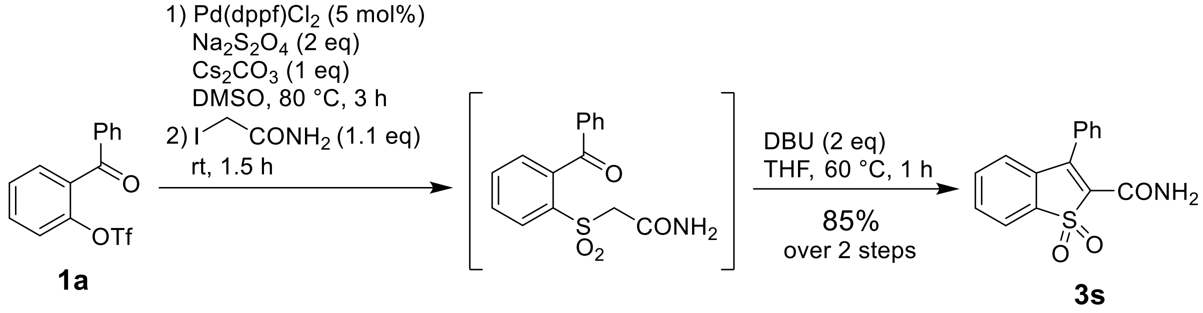

A 10 mL microwave vial (Biotage) was loaded with 2'-hydroxybenzophenone triflate **1a** (198 mg, 0.6 mmol; known compound[S7]), Pd(dppf)Cl_2·CH_2Cl_2 catalyst (25 mg, 0.03 mmol, 5 mol%), sodium dithionite (209 mg, 1.2 mmol, 2 equiv.) and cesium carbonate (196 mg, 0.6 mmol, 1 equiv.). Dry DMSO solvent (2.4 mL) was added, the reaction mixture was degassed on a Schlenk line, placed in a preheated 80 °C silicon oil bath and stirred for 3 h. Upon cooling down to rt, a solution of iodoacetamide (122 mg, 0.66 mmol, 1.1 equiv.) in DMSO (0.5 mL) was injected and the mixture was stirred at rt for 1.5 h. The reaction mixture was diluted with brine (50 mL), extracted with CH_2Cl_2 (3×20 mL) and the combined extracts were dried over Na_2SO_4. The solution was filtered through a short (3 cm) plug of silica, washing with additional EtOAc – CH_2Cl_2 (1:1), and the filtrate was evaporated. The crude intermediate was dissolved in dry THF (3 mL), DBU (0.18 mL, 1.2 mmol, 2 equiv.) was added, and the reaction mixture was stirred at 60 °C for 1 h. It was then diluted with 0.2 N hydrochloric acid (50 mL), extracted with CH_2Cl_2 (3×25 mL), the combined extracts were washed with brine and dried over Na_2SO_4. The product was isolated by flash column chromatography (12 g Interchim SiHP 30 µm cartridge, gradient 5% to 60% EtOAc/ CH_2Cl_2) and freeze-dried from 1,4-dioxane to give 146 mg (85% over 2 steps) of **3s** as off-white solid.

mp (*i*-PrOH/CH_2Cl_2): 275-276 °C (dec.).

^{1}H NMR (400 MHz, DMSO-d_6): δ 8.05 – 7.98 (m, 1H), 7.89 (br.s, 1H), 7.78 – 7.70 (m, 2H), 7.61 – 7.53 (m, 5H), 7.51 (s, 1H), 7.42 – 7.36 (m, 1H).

^{13}C NMR (101 MHz, DMSO-d_6): δ 159.5, 141.6, 135.8, 134.4, 133.6, 131.8, 130.6, 130.3, 129.4, 129.0, 128.6, 125.2, 121.7.

HRMS ($C_{15}H_{11}NO_3S$): *m/z* (positive mode) = 308.0355 (found [M+Na]$^+$), 308.0352 (calc.).

3t

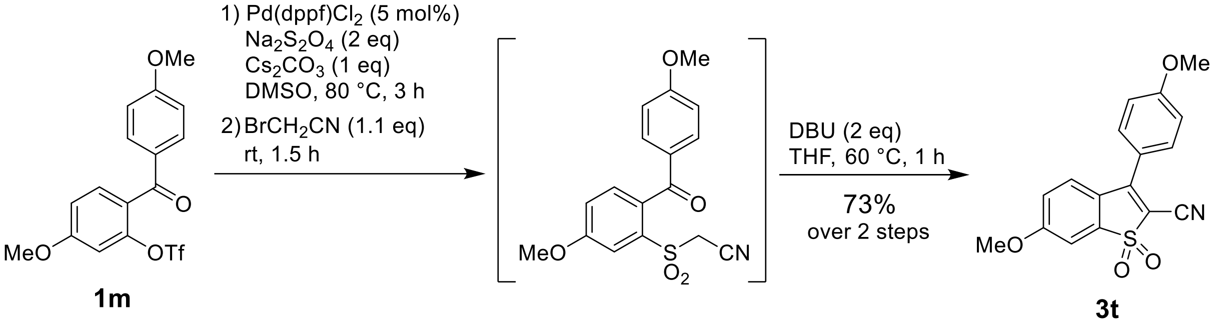

A 10 mL microwave vial (Biotage) was loaded with compound **1m** (195 mg, 0.5 mmol), Pd(dppf)Cl_2·CH_2Cl_2 catalyst (20 mg, 0.025 mmol, 5 mol%), sodium dithionite (174 mg, 1 mmol, 2 equiv.) and cesium carbonate (163 mg, 0.5 mmol, 1 equiv.). Dry DMSO solvent (2 mL) was added, the reaction mixture was degassed on a Schlenk line, placed in a preheated 80 °C silicon oil bath and stirred for 3 h. Upon cooling down to rt, bromoacetonitrile (38 µL, 0.55 mmol, 1.1 equiv.) was injected and the mixture was stirred at rt for 1.5 h. The reaction mixture was diluted with brine (50 mL), extracted with CH_2Cl_2 (3×20 mL) and the combined extracts were dried over Na_2SO_4. The solution was filtered through a short (3 cm) plug of silica, washing with additional EtOAc – CH_2Cl_2 (1:1), and the filtrate was evaporated. The crude intermediate was dissolved in dry THF (2.5 mL), DBU (0.15 mL, 1 mmol, 2 equiv.) was added, and the reaction mixture was stirred at 60 °C for 1 h. It was then diluted with 0.2 N hydrochloric acid (50 mL), extracted with CH_2Cl_2 (3×25 mL), the combined extracts were washed with brine and dried over Na_2SO_4. The product was isolated by flash column chromatography (12 g Interchim SiHP 30 µm cartridge, gradient 0% to 50% EtOAc + 20% CH_2Cl_2 constant additive) and freeze-dried from 1,4-dioxane to give 119 mg (73% over 2 steps) of **3t** as bright yellow solid.

mp (CH_2Cl_2/hexane): 229-230 °C.
^{1}H NMR (400 MHz, $CDCl_3$): δ 7.65 – 7.60 (m, 2H), 7.55 (d, *J* = 8.6 Hz, 1H), 7.37 (d, *J* = 2.4 Hz, 1H), 7.15 – 7.08 (m, 3H), 3.95 (s, 3H), 3.92 (s, 3H).
^{13}C NMR (101 MHz, $CDCl_3$): δ 164.4, 163.1, 155.4, 139.4, 130.8, 128.1, 121.5, 120.7, 119.5, 115.2, 110.6, 109.1, 108.4, 56.6, 55.8.
HRMS ($C_{17}H_{13}NO_4S$): *m/z* (positive mode) = 328.0638 (found $[M+H]^+$), 328.0638 (calc.).

3u

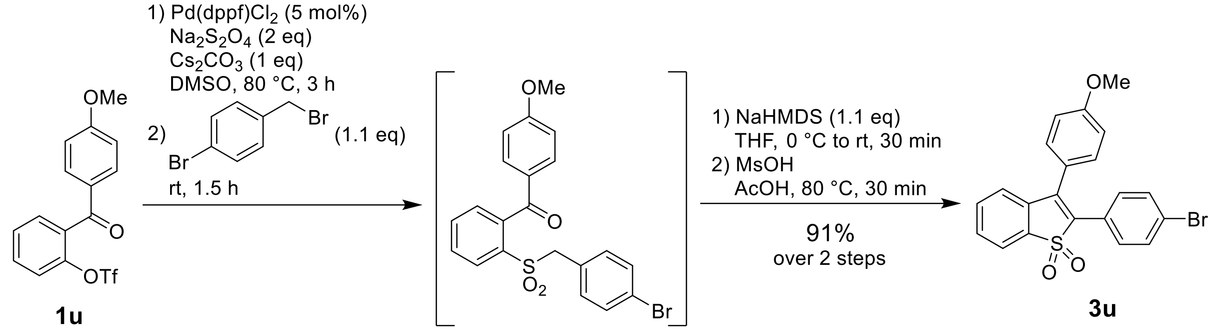

A 10 mL microwave vial (Biotage) was loaded with compound **1u** (180 mg, 0.5 mmol; known compound[S13]), Pd(dppf)Cl_2·CH_2Cl_2 catalyst (20 mg, 0.025 mmol, 5 mol%), sodium dithionite (174 mg, 1 mmol, 2 equiv.) and cesium carbonate (163 mg, 0.5 mmol, 1 equiv.). Dry DMSO solvent (2 mL) was added, the reaction mixture was degassed on a Schlenk line, placed in a preheated 80 °C silicon oil bath and stirred for 3 h. Upon cooling down to rt, a solution of 4-bromobenzyl bromide (138 mg, 0.55 mmol, 1.1 equiv.) in DMSO (0.5 mL) was injected and the mixture was stirred at rt for 1.5 h. The reaction mixture was diluted with brine (50 mL), extracted with CH_2Cl_2 (3×20 mL) and the combined extracts were dried over Na_2SO_4. The crude intermediate (223 mg) was isolated by flash column chromatography (12 g Interchim SiHP 30 µm cartridge, gradient 20% to 80% EtOAc/hexane) and used directly in the next step.

A solution of NaHMDS (0.28 mL of 2 M in THF, 0.55 mmol, ~1.1 equiv.) was added dropwise to a stirred solution of the crude sulfone from the previous step in dry THF (2.5 mL), cooled in ice-water bath. The reaction mixture was stirred for 30 min at rt, then quenched by addition of acetic acid (0.5 mL) and evaporated. The residue was dissolved in acetic acid (2 mL), methanesulfonic acid (0.5 mL) was added and the mixture was stirred at 80 °C for 30 min. It was then carefully poured into 50 mL of sat. aq. $NaHCO_3$, extracted with CH_2Cl_2 (3×20 mL), washed with brine and dried over Na_2SO_4. The product was isolated by flash column chromatography (12 g Interchim SiHP 30 µm cartridge, gradient 20% to 80% EtOAc/hexane) and freeze-dried from 1,4-dioxane to give 194 mg (91% over 2 steps) of **3u** as yellowish solid.

mp (EtOAc/hexane): 162-164 °C.

^{1}H NMR (400 MHz, $CDCl_3$): δ 7.88 – 7.81 (m, 1H), 7.59 – 7.52 (m, 2H), 7.48 – 7.42 (m, 2H), 7.39 – 7.33 (m, 3H), 7.28 – 7.21 (m, 2H), 7.00 – 6.94 (m, 2H), 3.86 (s, 3H).

^{13}C NMR (101 MHz, $CDCl_3$): δ 160.8, 138.6, 136.5, 135.7, 133.7, 133.3, 132.2, 130.9, 130.6, 130.3, 126.5, 124.4, 124.2, 122.4, 121.7, 114.9, 55.5.

HRMS ($C_{21}H_{15}BrO_3S$): *m/z* (positive mode) = 427.0000 (found $[M+H]^+$), 426.9998 (calc.).

3v

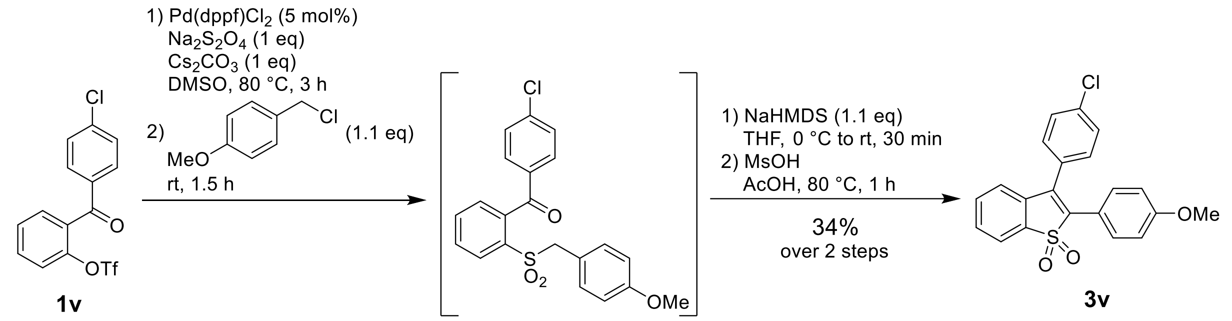

A 10 mL microwave vial (Biotage) was loaded with compound **1v** (183 mg, 0.5 mmol; known compound[S11]), Pd(dppf)Cl_2·CH_2Cl_2 catalyst (20 mg, 0.025 mmol, 5 mol%), sodium dithionite (87 mg, 0.5 mmol, 1 equiv.) and cesium carbonate (163 mg, 0.5 mmol, 1 equiv.). Dry DMSO solvent (2 mL) was added, the reaction mixture was degassed on a Schlenk line, placed in a preheated 80 °C silicon oil bath and stirred for 3 h. Upon cooling down to rt, 4-methoxybenzyl chloride (74 µL, 0.55 mmol, 1.1 equiv.) was injected and the mixture was stirred at rt for 1.5 h. The reaction mixture was diluted with brine (50 mL), extracted with CH_2Cl_2 (3×20 mL) and the combined extracts were dried over Na_2SO_4. The crude intermediate (149 mg) was isolated by flash column chromatography (12 g Interchim SiHP 30 µm cartridge, gradient 10% to 80% EtOAc/hexane) and used directly in the next step.

A solution of NaHMDS (0.2 mL of 2 M in THF, 0.4 mmol, ~1.1 equiv.) was added dropwise to a stirred solution of the crude sulfone from the previous step in dry THF (2 mL), cooled in ice-water bath. The reaction mixture was stirred for 30 min at rt, then quenched by addition of acetic acid (0.5 mL) and evaporated. The residue was dissolved in acetic acid (1.5 mL), methanesulfonic acid (0.38 mL) was added and the mixture was stirred at 80 °C for 1 h. It was then carefully poured into 50 mL of sat. aq. $NaHCO_3$, extracted with CH_2Cl_2 (3×20 mL), washed with brine and dried over Na_2SO_4. The product was isolated by flash column chromatography (12 g Interchim SiHP 30 µm cartridge, gradient 0% to 50% EtOAc/hexane + 20% CH_2Cl_2 constant additive) and freeze-dried from 1,4-dioxane to give 65 mg (34% over 2 steps) of **3v** as light-yellow solid.

mp (EtOAc/hexane): 162-163 °C.

^{1}H NMR (400 MHz, $CDCl_3$): δ 7.87 – 7.81 (m, 1H), 7.56 – 7.50 (m, 2H), 7.46 – 7.43 (m, 2H), 7.43 – 7.39 (m, 2H), 7.32 – 7.27 (m, 2H), 7.25 – 7.22 (m, 1H), 6.87 – 6.82 (m, 2H), 3.80 (s, 3H).

^{13}C NMR (101 MHz, $CDCl_3$): δ 160.9, 137.9, 136.2, 135.6, 135.2, 133.7, 133.2, 130.8, 130.7, 123.0, 129.81, 129.77, 123.7, 121.8, 119.0, 114.6, 55.4.

HRMS ($C_{21}H_{15}ClO_3S$): *m/z* (positive mode) = 383.0511 (found $[M+H]^+$), 383.0503 (calc.).

2w

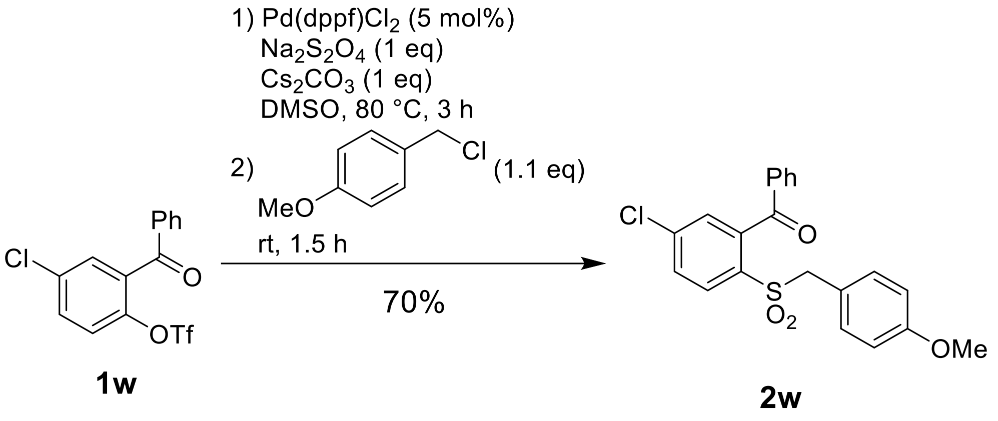

A 10 mL microwave vial (Biotage) was loaded with compound **1w** (146 mg, 0.4 mmol; known compound[S14]), Pd(dppf)$Cl_2 \cdot CH_2Cl_2$ catalyst (16 mg, 0.02 mmol, 5 mol%), sodium dithionite (70 mg, 0.4 mmol, 1 equiv.) and cesium carbonate (130 mg, 0.4 mmol, 1 equiv.). Dry DMSO solvent (1.6 mL) was added, the reaction mixture was degassed on a Schlenk line, placed in a preheated 80 °C silicon oil bath and stirred for 3 h. Upon cooling down to rt, 4-methoxybenzyl chloride (60 µL, 0.44 mmol, 1.1 equiv.) was injected and the mixture was stirred at rt for further 1.5 h. The reaction mixture was diluted 0.2 N HCl (50 mL), extracted with CH_2Cl_2 (3×20 mL), the combined extracts were washed with brine and dried over Na_2SO_4. The product was isolated by flash column chromatography (12 g Interchim SiHP 30 µm cartridge, gradient 5% to 70% EtOAc/hexane) and freeze-dried from 1,4-dioxane to give 113 mg (70%) of **2w** as white solid.

^{1}H NMR (400 MHz, $CDCl_3$): δ 7.86 – 7.81 (m, 2H), 7.67 – 7.61 (m, 1H), 7.54 – 7.45 (m, 1H), 7.44 (d, J = 8.4 Hz, 1H), 7.40 – 7.34 (m, 2H), 7.20 – 7.15 (m, 2H), 6.82 – 6.76 (m, 2H), 4.56 (s, 2H), 3.78 (s, 3H).

^{13}C NMR (101 MHz, $CDCl_3$): δ 195.2, 160.2, 142.1, 139.8, 136.0, 135.6, 134.5, 133.2, 132.4, 130.7, 129.6, 128.9, 128.2, 120.0, 114.2, 63.5, 55.4.

HRMS ($C_{21}H_{17}ClO_4S$): *m/z* (positive mode) = 423.0425 (found $[M+Na]^+$), 423.0428 (calc.).

3w

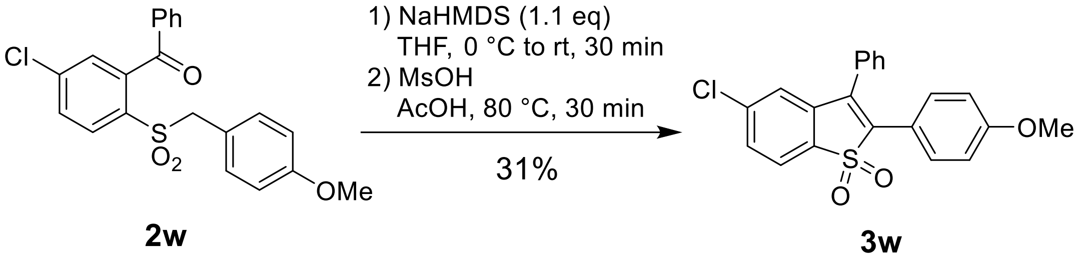

A solution of NaHMDS (0.15 mL of 2 M in THF, 0.3 mmol, ~1.1 equiv.) was added dropwise to a stirred solution of **2w** (113 mg, 0.282 mmol) in dry THF (1.5 mL), cooled in ice-water bath. The reaction mixture was warmed up to rt and stirred for 30 min, then quenched by addition of acetic acid (0.4 mL) and evaporated. The residue was resuspended in acetic acid (1.5 mL), methanesulfonic acid (0.3 mL) was added and the mixture was stirred for at 80 °C for 30 min. It was then carefully poured into 50 mL of sat. aq. $NaHCO_3$, extracted with CH_2Cl_2 (3×20 mL), washed with brine and dried over Na_2SO_4. The product was isolated by flash column chromatography (12 g Interchim SiHP 30 µm cartridge, gradient 0% to 50% EtOAc/hexane + 20% CH_2Cl_2 constant additive) and freeze-dried from 1,4-dioxane to give 33 mg (31%) of **3w** as light-yellow solid.

mp (CH_2Cl_2/hexane): 219-220 °C.
^{1}H NMR (400 MHz, $CDCl_3$): δ 7.76 (d, J = 8.0 Hz, 1H), 7.51 – 7.46 (m, 4H), 7.43 – 7.39 (m, 2H), 7.36 – 7.31 (m, 2H), 7.21 (d, J = 1.7 Hz, 1H), 6.84 – 6.79 (m, 2H), 3.79 (s, 3H).
^{13}C NMR (101 MHz, $CDCl_3$): δ 161.0, 140.1, 138.8, 135.8, 135.1, 134.4, 130.8, 129.8, 129.62, 129.61, 129.1, 124.3, 122.7, 119.0, 114.6, 55.4.
HRMS ($C_{21}H_{25}ClO_3S$): m/z (positive mode) = 405.0308 (found [M+Na]$^+$), 405.0323 (calc.).

3x

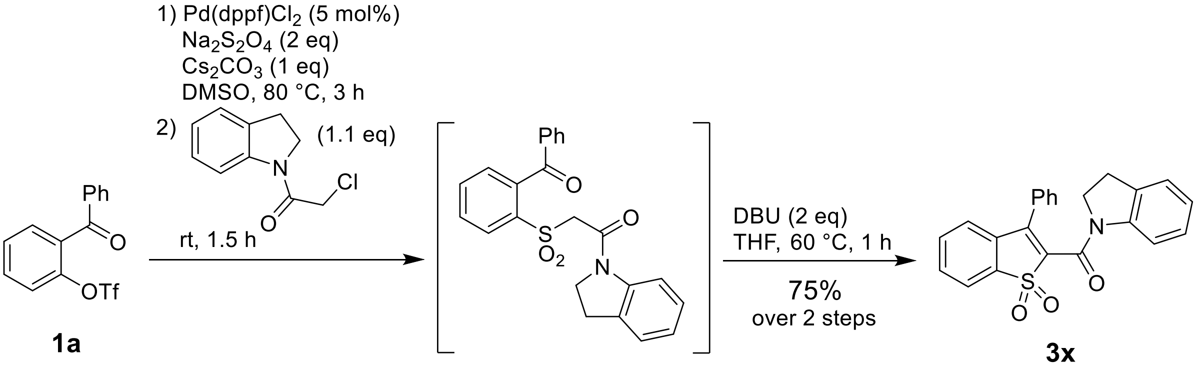

A 10 mL microwave vial (Biotage) was loaded with 2'-hydroxybenzophenone triflate **1a** (165 mg, 0.5 mmol; known compound[S7]), Pd(dppf)Cl_2·CH_2Cl_2 catalyst (20 mg, 0.025 mmol, 5 mol%), sodium dithionite (174 mg, 1 mmol, 2 equiv.) and cesium carbonate (163 mg, 0.5 mmol, 1 equiv.). Dry DMSO solvent (2 mL) was added, the reaction mixture was degassed on a Schlenk line, placed in a preheated 80 °C silicone oil bath and stirred for 3 h. Upon cooling down to rt, a solution of *N*-(chloroacetyl)indoline (108 mg, 0.55 mmol, 1.1 equiv.; known compound[S15]) in DMSO (0.5 mL) was injected and the mixture was stirred at rt for 1.5 h. The reaction mixture was diluted with brine (50 mL), extracted with CH_2Cl_2 (3×20 mL) and the combined extracts were dried over Na_2SO_4. The intermediate sulfone (214 mg) was isolated by flash column chromatography (12 g Interchim SiHP 30 µm cartridge, gradient 10% to 80% EtOAc/hexane) and was used directly in the next step.

The crude intermediate was dissolved in dry THF (2.5 mL), DBU (0.15 mL, 1 mmol, 2 equiv.) was added, and the reaction mixture was stirred at 60 °C for 1 h. It was then diluted with 0.2 N hydrochloric acid (50 mL), extracted with CH_2Cl_2 (3×25 mL), the combined extracts were washed with brine and dried over Na_2SO_4. The product was isolated by flash column chromatography (12 g Interchim SiHP 30 µm cartridge, gradient 20% to 80% EtOAc/hexane) and freeze-dried from 1,4-dioxane to give 145 mg (75% over 2 steps) of **3x** as light-yellow solid.

mp (CH_2Cl_2/hexane): 233-234 °C.
^{1}H NMR (400 MHz, pyridine-d_5, 60 °C): δ 8.48 (br.s, 1H), 8.12 – 8.03 (m, 1H), 7.78 – 7.61 (m, 4H), 7.56 – 7.42 (m, 4H), 7.20 (br.s, 1H), 7.10 – 6.99 (m, 2H), 4.04 (br.t, J = 8.3 Hz, 2H), 2.79 (br.t, J = 8.3 Hz, 2H) – broad signals in common solvents ($CDCl_3$, DMSO-d_6) due to hindered rotations.
^{13}C NMR (101 MHz, DMSO-d_6): δ 156.7, 141.3, 140.8, 135.9, 134.6, 132.8, 131.9, 131.0, 130.0, 129.5, 129.1, 128.3, 127.2, 125.5, 125.2, 122.0, 116.6, 48.5, 27.2.
HRMS ($C_{23}H_{17}NO_3S$): *m/z* (positive mode) = 388.1001 (found $[M+H]^+$), 388.1002 (calc.).

3y

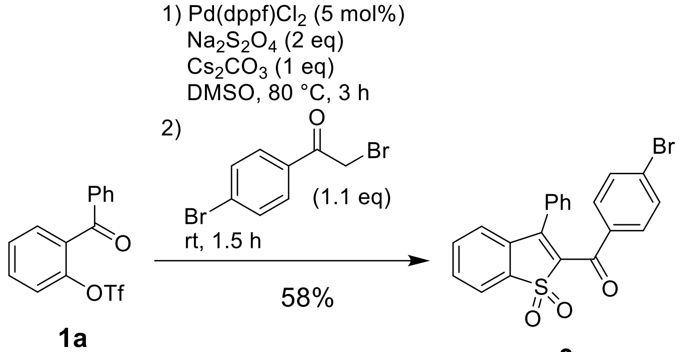

1a **3y**

A 10 mL microwave vial (Biotage) was loaded with 2'-hydroxybenzophenone triflate **1a** (165 mg, 0.5 mmol; known compound[S7]), Pd(dppf)Cl_2·CH_2Cl_2 catalyst (20 mg, 0.025 mmol, 5 mol%), sodium dithionite (174 mg, 1 mmol, 2 equiv.) and cesium carbonate (163 mg, 0.5 mmol, 1 equiv.). Dry DMSO solvent (2 mL) was added, the reaction mixture was degassed on a Schlenk line, placed in a preheated 80 °C silicone oil bath and stirred for 3 h. Upon cooling down to rt, a solution of 2,4'-dibromoacetophenone (153 mg, 0.55 mmol, 1.1 equiv.) in DMSO (0.5 mL) was injected and the mixture was stirred at rt for 1.5 h. The reaction mixture was diluted with brine (50 mL), extracted with CH_2Cl_2 (3×20 mL) and the combined extracts were dried over Na_2SO_4. The product was isolated by flash column chromatography (12 g Interchim SiHP 30 µm cartridge, gradient 0% to 50% EtOAc/hexane + 20% CH_2Cl_2 constant additive) and freeze-dried from 1,4-dioxane to give 124 mg (58%) of **3y** as light-yellow solid.

mp (CH_2Cl_2/hexane): 208-209 °C.
^{1}H NMR (400 MHz, $CDCl_3$): δ 7.90 – 7.86 (m, 1H), 7.68 (td, J = 7.5, 1.2 Hz, 1H), 7.66 – 7.59 (m, 3H), 7.52 – 7.47 (m, 1H), 7.43 – 7.30 (m, 7H).
^{13}C NMR (101 MHz, $CDCl_3$): δ 187.0, 145.9, 137.5, 135.8, 135.2, 133.9, 132.2, 131.9, 131.1, 130.9, 129.6, 129.4, 129.2, 128.9, 125.8, 122.1.
HRMS ($C_{21}H_{13}BrO_3S$): *m/z* (positive mode) = 446.9663 (found $[M+Na]^+$), 446.9661 (calc.).

3z

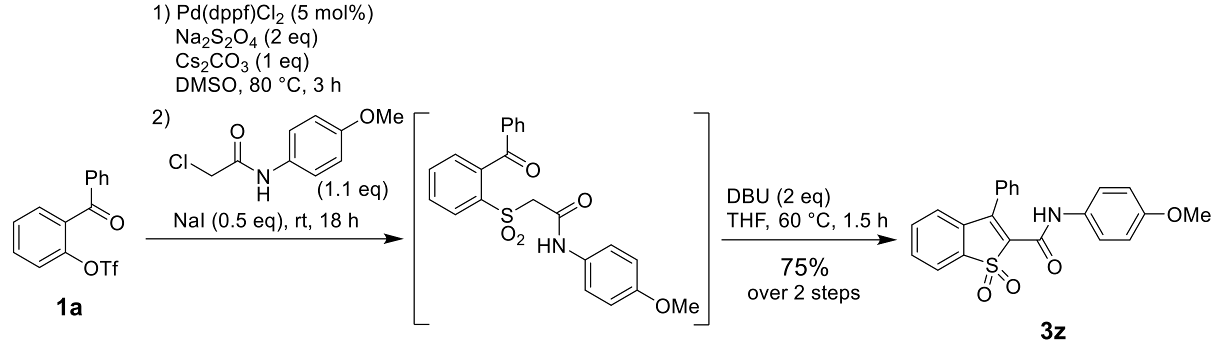

A 10 mL microwave vial (Biotage) was loaded with 2'-hydroxybenzophenone triflate **1a** (165 mg, 0.5 mmol; known compound[S7]), Pd(dppf)Cl_2·CH_2Cl_2 catalyst (20 mg, 0.025 mmol, 5 mol%), sodium dithionite (174 mg, 1 mmol, 2 equiv.) and cesium carbonate (163 mg, 0.5 mmol, 1 equiv.). Dry DMSO solvent (2 mL) was added, the reaction mixture was degassed on a Schlenk line, placed in a preheated 80 °C silicone oil bath and stirred for 3 h. Upon cooling down to rt, a solution of 2-chloro-4'-methoxyacetanilide (110 mg, 0.55 mmol, 1.1 equiv.; known compound[S16]) and sodium iodide (40 mg, 0.25 mmol, 0.5 equiv.) in DMSO (0.5 mL) was injected and the mixture was stirred at rt overnight (18 h). The reaction mixture was diluted with brine (50 mL), extracted with CH_2Cl_2 (3×20 mL) and the combined extracts were dried over Na_2SO_4. The crude intermediate (167 mg) was isolated by flash column chromatography (12 g Interchim SiHP 30 µm cartridge, gradient 0% to 100% EtOAc/hexane + 50% CH_2Cl_2 constant additive) and was used directly in the next step.

The crude intermediate was dissolved in dry THF (2.5 mL), DBU (0.15 mL, 1 mmol, 2 equiv.) was added, and the reaction mixture was stirred at 60 °C for 1.5 h. It was then diluted with 0.2 N hydrochloric acid (50 mL), extracted with CH_2Cl_2 (3×25 mL), the combined extracts were washed with brine and dried over Na_2SO_4. The product was isolated by flash column chromatography (12 g Interchim SiHP 30 µm cartridge, gradient 20% to 80% EtOAc/hexane) and freeze-dried from 1,4-dioxane to give 147 mg (75% over 2 steps) of **3z** as yellow solid.

mp (CH_2Cl_2/hexane): 95-96 °C.
^{1}H NMR (400 MHz, $CDCl_3$): δ 7.97 (br.s, 1H), 7.87 (ddd, J = 7.4, 1.3, 0.6 Hz, 1H), 7.67 (td, J = 7.5, 1.1 Hz, 1H), 7.60 (td, J = 7.6, 1.2 Hz, 1H), 7.58 – 7.54 (m, 3H), 7.54 – 7.48 (m, 2H), 7.40 – 7.34 (m, 2H), 7.32 – 7.28 (m, 1H), 6.85 – 6.79 (m, 2H), 3.77 (s, 3H).
^{13}C NMR (101 MHz, $CDCl_3$): δ 157.1, 155.3, 147.4, 136.1, 134.1, 132.2, 132.1, 131.7, 130.7, 130.1, 129.6, 129.1, 128.7, 126.2, 122.1, 122.0, 114.3, 55.6.
HRMS ($C_{22}H_{17}NO_4S$): m/z (positive mode) = 392.0954 (found $[M+H]^+$), 392.0951 (calc.).

3aa

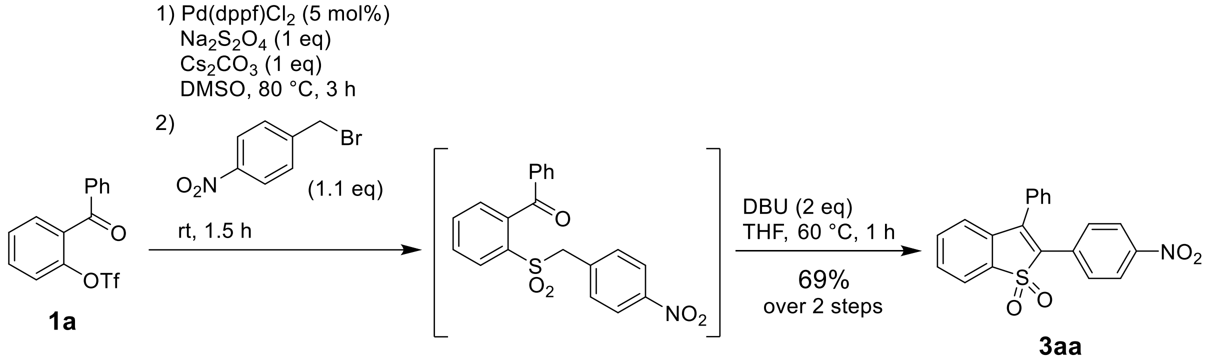

A 10 mL microwave vial (Biotage) was loaded with 2'-hydroxybenzophenone triflate **1a** (165 mg, 0.5 mmol; known compound[S7]), Pd(dppf)Cl_2·CH_2Cl_2 catalyst (20 mg, 0.025 mmol, 5 mol%), sodium dithionite (87 mg, 0.5 mmol, 2 equiv.) and cesium carbonate (163 mg, 0.5 mmol, 1 equiv.). Dry DMSO solvent (2 mL) was added, the reaction mixture was degassed on a Schlenk line, placed in a preheated 80 °C silicone oil bath and stirred for 3 h. Upon cooling down to rt, a solution of 4-nitrobenzyl bromide (119 mg, 0.55 mmol, 1.1 equiv.) in DMSO (0.5 mL) was injected and the mixture was stirred at rt for 1.5 h. The reaction mixture was diluted with brine (50 mL), extracted with CH_2Cl_2 (3×20 mL) and the combined extracts were dried over Na_2SO_4. The crude intermediate (143 mg) was isolated by flash column chromatography (12 g Interchim SiHP 30 µm cartridge, gradient 20% to 80% EtOAc/hexane) and was used directly in the next step.

The crude intermediate was dissolved in dry THF (3 mL), DBU (112 µL, 0.75 mmol, 2 equiv.) was added, and the reaction mixture was stirred at 60 °C for 1 h. It was then diluted with brine (50 mL, with 1 mL acetic acid added), extracted with EtOAc (3×25 mL), the combined extracts were washed with brine and dried over Na_2SO_4. The product was isolated by flash column chromatography (12 g Interchim SiHP 30 µm cartridge, gradient 10% to 80% EtOAc/hexane) and freeze-dried from 1,4-dioxane to give 125 mg (69% over 2 steps) of **3aa** as light-yellow solid.

mp (CH_2Cl_2/hexane): 180-180.5 °C.
^{1}H NMR (400 MHz, $CDCl_3$): δ 8.17 – 8.12 (m, 2H), 7.91 – 7.86 (m, 1H), 7.67 – 7.63 (m, 2H), 7.63 – 7.58 (m, 2H), 7.55 – 7.46 (m, 3H), 7.39 – 7.30 (m, 3H).
^{13}C NMR (101 MHz, $CDCl_3$): δ 148.1, 141.1, 136.5, 135.4, 134.03, 133.96, 132.6, 131.1, 130.4, 130.3, 130.1, 129.7, 129.0, 124.9, 124.0, 121.9.
HRMS ($C_{20}H_{13}NO_4S$): *m/z* (positive mode) = 364.0639 (found $[M+H]^+$), 364.0638 (calc.).

2ab

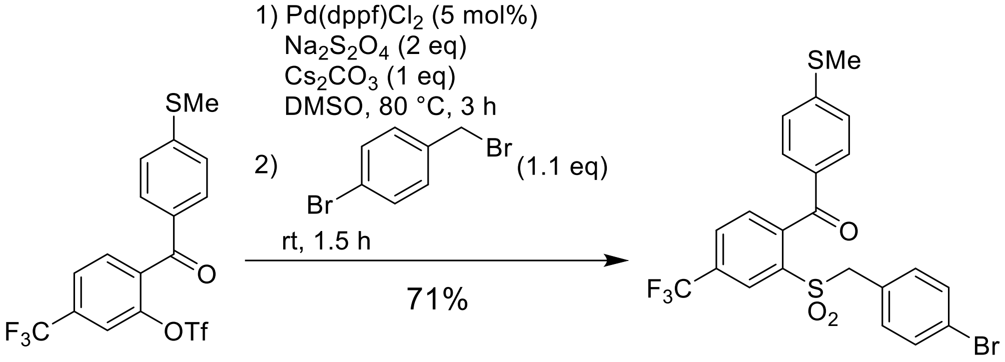

1ab **2ab**

A 10 mL microwave vial (Biotage) was loaded with compound **1ab** (222 mg, 0.5 mmol), $Pd(dppf)Cl_2 \cdot CH_2Cl_2$ catalyst (20 mg, 0.025 mmol, 5 mol%), sodium dithionite (174 mg, 1 mmol, 2 equiv.) and cesium carbonate (163 mg, 0.5 mmol, 1 equiv.). Dry DMSO solvent (2 mL) was added, the reaction mixture was degassed on a Schlenk line, placed in a preheated 80 °C silicon oil bath and stirred for 3 h. Upon cooling down to rt, 4-bromobenzyl bromide (138 mg, 0.55 mmol, 1.1 equiv.; freshly dissolved in 0.5 mL dry DMSO) was injected and the mixture was stirred at rt for further 1.5 h. The reaction mixture was diluted with brine (50 mL), extracted with CH_2Cl_2 (3×20 mL), the combined extracts were dried over Na_2SO_4. The product was isolated by flash column chromatography (12 g Interchim SiHP 30 µm cartridge, gradient 5% to 60% EtOAc/hexane) and freeze-dried from 1,4-dioxane to give 188 mg (71%) of **2ab** as yellowish solid.

^{1}H NMR (400 MHz, $CDCl_3$): δ 7.89 (ddd, *J* = 8.0, 1.8, 0.8 Hz, 1H), 7.78 (dt, *J* = 1.8, 0.6 Hz, 1H), 7.74 – 7.68 (m, 2H), 7.52 (dt, *J* = 7.9, 0.7 Hz, 1H), 7.43 – 7.37 (m, 2H), 7.31 – 7.25 (m, 2H), 7.15 – 7.10 (m, 2H), 4.60 (s, 2H), 2.52 (s, 3H).
^{19}F NMR (376 MHz, $CDCl_3$): δ -63.11.
^{13}C NMR (101 MHz, $CDCl_3$): δ 194.4, 148.7, 143.9, 138.0, 132.6, 132.1, 132.0 (q, *J* = 34.4 Hz), 132.0, 130.9, 129.9 (q, *J* = 3.7 Hz), 128.9, 128.8 (q, *J* = 3.8 Hz), 126.7, 125.1, 123.8, 122.7 (q, *J* = 273.2 Hz), 63.5, 14.8.
HRMS ($C_{22}H_{16}BrF_3O_3S_2$): *m/z* (positive mode) = 528.9752 (found $[M+H]^+$), 528.9749 (calc.).

3ab

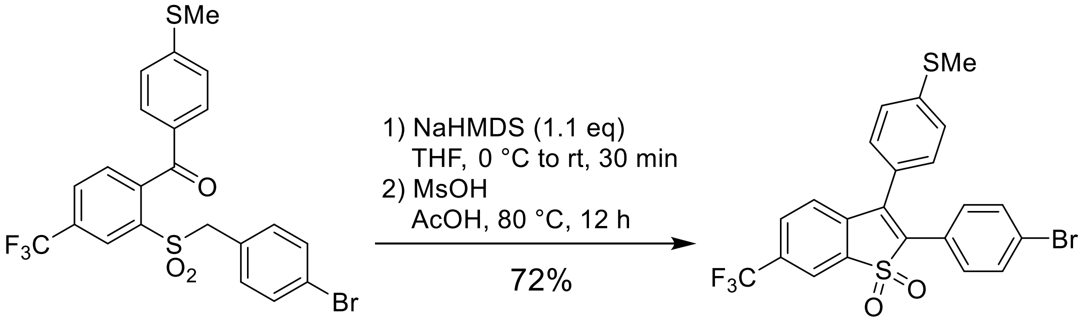

2ab **3ab**

A solution of NaHMDS (0.18 mL of 2 M in THF, 0.36 mmol, ~1.1 equiv.) was added dropwise to a stirred solution of **2ab** (173 mg, 0.33 mmol) in dry THF (2.5 mL), cooled in ice-water bath. The reaction mixture was warmed up to rt and stirred for 30 min, then quenched by addition of acetic acid (0.5 mL) and evaporated. The residue was resuspended in acetic acid (2 mL), methanesulfonic acid (0.4 mL) was added and the mixture was stirred for at 80 °C for 12 h. It was then carefully poured into 50 mL of sat. aq. $NaHCO_3$, extracted with CH_2Cl_2 (3×30 mL), washed with brine and dried over Na_2SO_4. The product was isolated by flash column chromatography (12 g Interchim SiHP 30 µm cartridge, gradient 0% to 25% EtOAc/hexane + 20% CH_2Cl_2 constant additive) and freeze-dried from 1,4-dioxane to give 120 mg (72%) of **3ab** as yellowish solid.

mp (CH_2Cl_2/hexane): 219-220 °C.

^{1}H NMR (400 MHz, $CDCl_3$): δ 8.09 (dt, *J* = 1.4, 0.7 Hz, 1H), 7.82 (ddd, *J* = 8.1, 1.7, 0.8 Hz, 1H), 7.51 – 7.45 (m, 3H), 7.39 – 7.34 (m, 2H), 7.33 – 7.29 (m, 2H), 7.25 – 7.20 (m, 2H), 2.53 (s, 3H).

^{19}F NMR (376 MHz, $CDCl_3$): δ -62.71.

^{13}C NMR (101 MHz, $CDCl_3$): δ 142.3, 138.4, 137.1, 136.9, 136.3, 132.6 (q, *J* = 33.9 Hz), 132.5, 130.9 (q, *J* = 4.4 Hz), 130.8, 129.4, 126.6, 125.8, 125.6, 125.0, 124.6, 123.2 (q, *J* = 272.9 Hz), 119.1 (q, *J* = 3.9 Hz), 15.1.

HRMS ($C_{22}H_{14}BrF_3O_2S_2$): *m/z* (positive mode) = 532.9461 (found $[M+Na]^+$), 532.9463 (calc.).

2ac

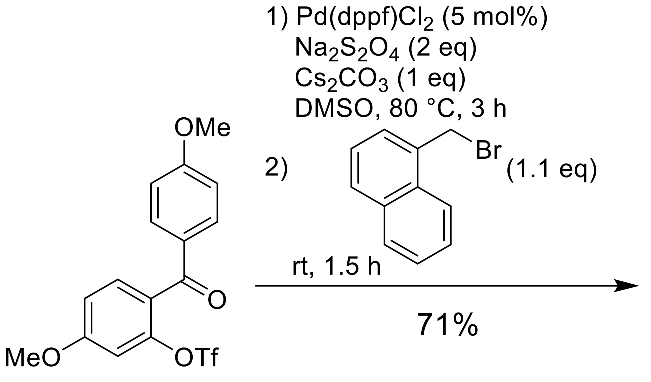

1m **2ac**

A 10 mL microwave vial (Biotage) was loaded with compound **1m** (195 mg, 0.5 mmol), Pd(dppf)Cl_2·CH_2Cl_2 catalyst (20 mg, 0.025 mmol, 5 mol%), sodium dithionite (174 mg, 1 mmol, 2 equiv.) and cesium carbonate (163 mg, 0.5 mmol, 1 equiv.). Dry DMSO solvent (2 mL) was added, the reaction mixture was degassed on a Schlenk line, placed in a preheated 80 °C silicon oil bath and stirred for 3 h. Upon cooling down to rt, 1-(bromomethyl)naphthalene (122 mg, 0.55 mmol, 1.1 equiv.; freshly dissolved in 0.5 mL dry DMSO) was injected and the mixture was stirred at rt for further 1.5 h. The reaction mixture was diluted with brine (50 mL), extracted with CH_2Cl_2 (3×20 mL), the combined extracts were dried over Na_2SO_4. The product was isolated by flash column chromatography (12 g Interchim SiHP 30 µm cartridge, gradient 5% to 60% EtOAc/hexane + 20% CH_2Cl_2 constant additive) and freeze-dried from 1,4-dioxane to give 181 mg (81%) of **2ac** as white solid (purity 85-90%, used without additional purification).

^{1}H NMR (400 MHz, $CDCl_3$): δ 8.15 – 8.10 (m, 1H), 7.89 – 7.84 (m, 2H), 7.84 – 7.78 (m, 2H), 7.52 (dd, *J* = 7.0, 1.2 Hz, 1H), 7.44 – 7.35 (m, 3H), 7.29 (d, *J* = 8.4 Hz, 1H), 6.99 (dd, *J* = 8.4, 2.6 Hz, 1H), 6.97 – 6.92 (m, 2H), 6.77 (d, *J* = 2.6 Hz, 1H), 5.23 (s, 2H), 3.87 (s, 3H), 3.45 (s, 3H).

^{13}C NMR (101 MHz, $CDCl_3$): δ 195.3, 164.3, 159.9, 138.8, 133.8, 133.1, 132.8, 132.6, 131.2, 130.1, 130.0, 129.6, 128.4, 126.7, 126.0, 125.3, 125.2, 124.3, 119.3, 115.6, 114.0, 61.1, 55.8, 55.7.

HRMS ($C_{26}H_{22}O_5S$): *m/z* (positive mode) = 447.1262 (found $[M+H]^+$), 447.1261 (calc.).

3ac

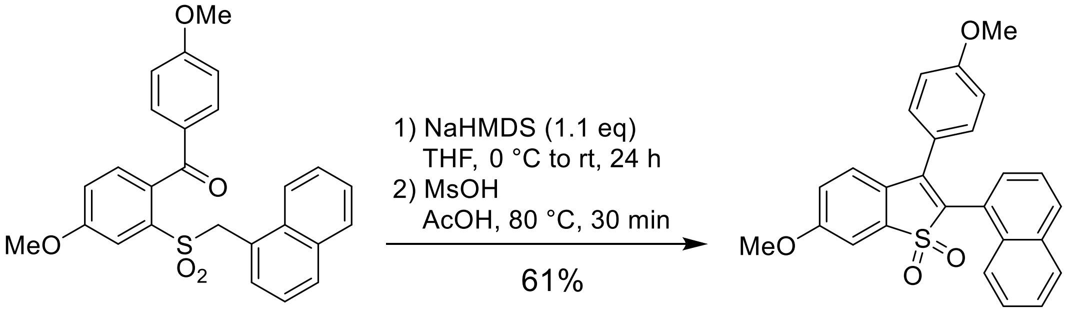

2ac **3ac**

A solution of NaHMDS (0.3 mL of 2 M in THF, 0.60 mmol, ~1.6 equiv.) was added dropwise to a stirred solution of **2ac** (166 mg, ≤0.37 mmol, 85-90% purity) in dry THF (3 mL), cooled in ice-water bath. The reaction mixture was warmed up to rt and stirred for 24 h; it was then quenched by addition of acetic acid (0.5 mL) and evaporated. The residue was resuspended in acetic acid (2 mL), methanesulfonic acid (0.5 mL) was added and the mixture was stirred for at 80 °C for 30 min. On cooling to rt, the mixture was carefully poured into 50 mL of sat. aq. $NaHCO_3$, extracted with CH_2Cl_2 (3×30 mL), washed with brine and dried over Na_2SO_4. The product was isolated by flash column chromatography (12 g Interchim SiHP 30 µm cartridge, gradient 0% to 20% EtOAc/hexane + 50% CH_2Cl_2 constant additive) and freeze-dried from 1,4-dioxane to give 97 mg (61%) of **3ac** as yellowish solid.

mp (CH_2Cl_2/hexane): 124-125 °C.
^{1}H NMR (400 MHz, $CDCl_3$): δ 7.96 (ddd, *J* = 7.6, 1.9, 0.8 Hz, 1H), 7.88 (dt, *J* = 8.4, 1.1 Hz, 1H), 7.83 – 7.79 (m, 1H), 7.68 (dd, *J* = 7.1, 1.3 Hz, 1H), 7.49 (dd, *J* = 8.3, 7.1 Hz, 1H), 7.45 (d, *J* = 2.5 Hz, 1H), 7.44 – 7.36 (m, 3H), 7.21 – 7.16 (m, 2H), 7.07 (dd, *J* = 8.5, 2.5 Hz, 1H), 6.74 – 6.67 (m, 2H), 3.92 (s, 3H), 3.68 (s, 3H).
^{13}C NMR (101 MHz, $CDCl_3$): δ 161.7, 160.4, 141.0, 139.1, 134.6, 133.9, 132.6, 130.3, 130.1, 128.4, 126.9, 126.3, 125.9, 125.6, 125.5, 125.1, 124.6, 123.0, 119.0, 114.2, 107.6, 56.2, 55.3.
HRMS ($C_{26}H_{20}O_4S$): *m/z* (positive mode) = 429.1157 (found $[M+H]^+$), 429.1155 (calc.).

3ad

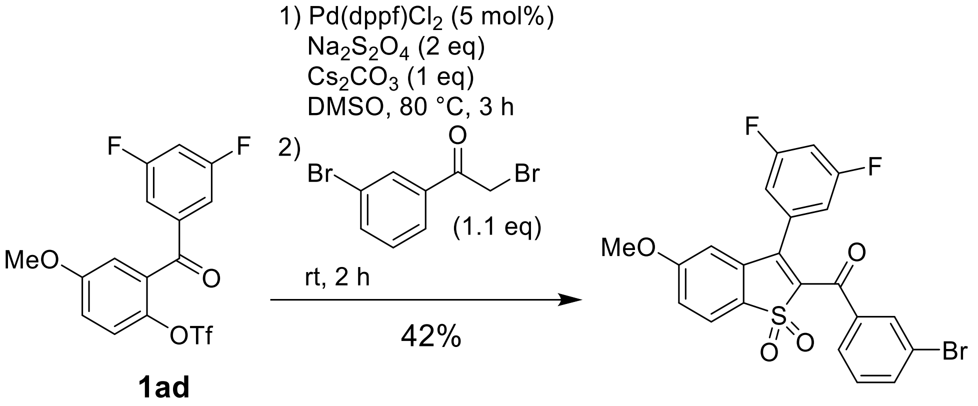

A 10 mL microwave vial (Biotage) was loaded with compound **1ad** (198 mg, 0.5 mmol), Pd(dppf)Cl_2·CH_2Cl_2 catalyst (20 mg, 0.025 mmol, 5 mol%), sodium dithionite (174 mg, 1 mmol, 2 equiv.) and cesium carbonate (163 mg, 0.5 mmol, 1 equiv.). Dry DMSO solvent (2 mL) was added, the reaction mixture was degassed on a Schlenk line, placed in a preheated 80 °C silicone oil bath and stirred for 3 h. Upon cooling down to rt, a solution of 2,3'-dibromoacetophenone (153 mg, 0.55 mmol, 1.1 equiv.) in DMSO (0.5 mL) was injected and the mixture was stirred at rt for 2 h. The reaction mixture was diluted with brine (50 mL), extracted with CH_2Cl_2 (3×20 mL) and the combined extracts were dried over Na_2SO_4. The product was isolated by flash column chromatography (12 g Interchim SiHP 30 µm cartridge, gradient 10% to 60% EtOAc/hexane) and freeze-dried from 1,4-dioxane to give 102 mg (42%) of **3ad** as light-yellow solid.

^{1}H NMR (400 MHz, $CDCl_3$): δ 7.85 (t, *J* = 1.7 Hz, 1H), 7.81 (d, *J* = 8.4 Hz, 1H), 7.74 (ddd, *J* = 7.9, 1.7, 1.1 Hz, 1H), 7.62 (ddd, *J* = 7.9, 2.0, 1.1 Hz, 1H), 7.26 (t, *J* = 7.9 Hz, 1H), 7.13 (dd, *J* = 8.5, 2.3 Hz, 1H), 6.91 – 6.80 (m, 4H), 3.88 (s, 3H).
^{19}F NMR (376 MHz, $CDCl_3$): δ -106.24.
^{13}C NMR (101 MHz, $CDCl_3$): δ 185.9, 164.5 (d, *J* = 12.8 Hz), 164.3, 162.0 (d, *J* = 12.8 Hz), 143.3 (t, *J* = 2.6 Hz), 138.3, 137.8, 137.2, 132.5 (t, *J* = 9.8 Hz), 132.4, 130.4, 128.6, 128.3, 124.1, 122.9, 115.8, 112.5, 112.1 (d, *J* = 11.1 Hz), 112.1 (d, *J* = 27.2 Hz), 106.3 (t, *J* = 24.9 Hz), 56.3.
HRMS ($C_{22}H_{13}BrF_2O_4S$): *m/z* (positive mode) = 512.9573 (found $[M+Na]^+$), 512.9578 (calc.).

Preparation of aryl triflates 7, 9, 10, 12

7

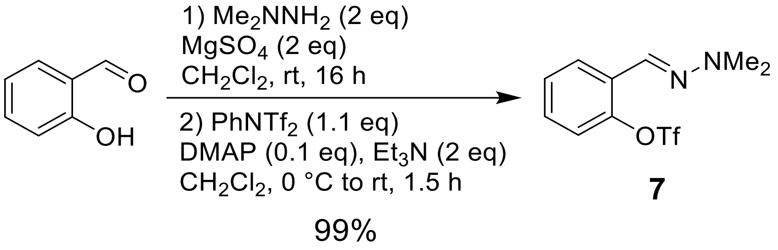

Following a procedure from [S17], *N*,*N*-dimethylhydrazine (0.76 mL, 10 mmol, 2 equiv) was added to a mixture of salicylaldehyde (521 µL, 610 mg, 5 mmol) and anhydrous $MgSO_4$ (1.2 g, 10 mmol, 2 equiv) in CH_2Cl_2 (30 mL), and the reaction mixture was stirred at rt for overnight (16 h). The mixture was then filtered through a short (1 cm) plug of Celite, the filtrate was evaporated, chased with toluene and the remaining crude product was used directly in the following step.

The entire amount from the previous step was dissolved in CH_2Cl_2 (10 mL), 4-(dimethylamino)pyridine (DMAP; 61 mg, 0.5 mmol, 0.1 equiv) and *N*-phenyl-bis(trifluoromethanesulfonimide) (1.96 g, 5.5 mmol, 1.1 equiv) were added, the reaction mixture was cooled in ice-water bath followed by addition of triethylamine (1.4 mL, 10 mmol, 2 equiv). The reaction mixture was allowed to warm up to rt and left stirring for 1.5 h. It was then evaporated on silica and the product was isolated by flash column chromatography (40 g BGB Scorpius silica 50 µm cartridge, gradient 10% to 60% CH_2Cl_2/hexane) and dried *in vacuo* to give 1.48 g (99% over two steps) of **7** as colorless oil.

^{1}H NMR (400 MHz, $CDCl_3$): δ 7.97 – 7.91 (m, 1H), 7.34 – 7.28 (m, 1H), 7.28 – 7.19 (m, 3H), 3.05 (s, 6H).
^{19}F NMR (376 MHz, $CDCl_3$): δ -73.72.
^{13}C NMR (101 MHz, $CDCl_3$): δ 146.6, 130.4, 128.4, 127.9, 126.6, 123.2, 121.7, 118.8 (q, *J* = 320.3 Hz), 42.6.
HRMS ($C_{10}H_{11}F_3N_2O_3S$): *m/z* (positive mode) = 297.0513 (found $[M+H]^+$), 297.0515 (calc.).

9

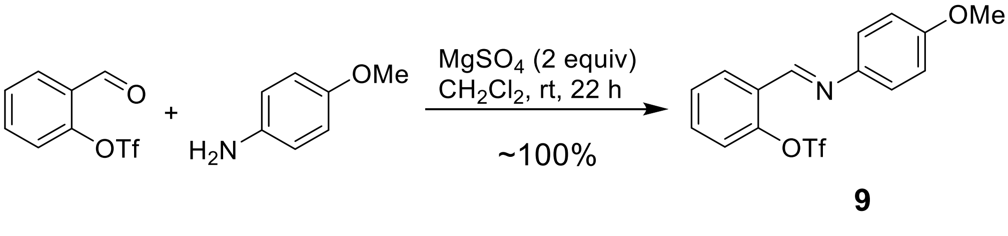

Anhydrous $MgSO_4$ (240 mg, 2 mmol, 2 equiv.) was added to the solution of 2-formylphenyl triflate[S18] (254 mg, 1 mmol) and *p*-anisidine (123 mg, 1 mmol) in CH_2Cl_2 (4 mL), and the reaction mixture was stirred at rt overnight (22 h). The mixture was then filtered through a short (1 cm) plug of Celite, the filtrate was evaporated and the remaining viscous yellow oil was freeze-dried from 1,4-dioxane to give 362 mg (~quant.) of the product **9** as yellowish solid.

mp (EtOAc/hexane): 39-40 °C.
^{1}H NMR (400 MHz, $CDCl_3$): δ 8.75 (s, 1H), 8.29 (dd, *J* = 7.6, 2.1 Hz, 1H), 7.56 – 7.51 (m, 1H), 7.48 (tdd, *J* = 7.4, 1.5, 0.7 Hz, 1H), 7.36 (dd, *J* = 8.0, 1.4 Hz, 1H), 7.32 – 7.27 (m, 2H), 6.99 – 6.92 (m, 2H), 3.84 (s, 3H).
^{19}F NMR (376 MHz, $CDCl_3$): δ -73.20.
^{13}C NMR (101 MHz, $CDCl_3$): δ 159.2, 150.4, 149.0, 144.1, 132.3, 129.5, 129.3, 128.8, 122.7, 122.0, 118.8 (q, *J* = 320.4 Hz), 114.7, 55.7.
LC-HRMS data were impossible to obtain due to hydrolytic instability of the compound.

10a

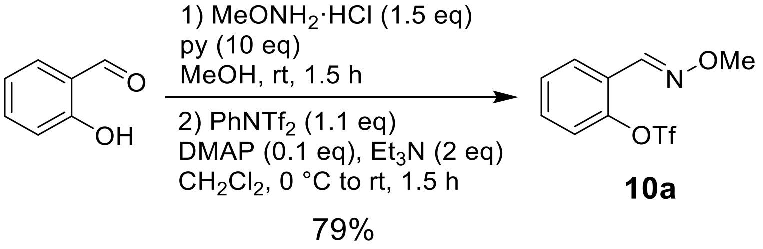

Pyridine (4.0 mL, 50 mmol, 10 equiv) was added to a mixture of salicylaldehyde (521 µL, 610 mg, 5 mmol) and *O*-methylhydroxylamine hydrochloride (630 mg, 7.5 mmol, 1.5 equiv) in methanol (10 mL), and the resulting solution was stirred at rt for 1.5 h. The mixture was then diluted with 0.2 N HCl (150 mL), extracted with EtOAc (3×50 mL), the combined extracts were washed with 0.5 N HCl (100 mL), brine (50 mL) and dried over Na_2SO_4. The filtrate was evaporated, chased with toluene, and the crude oxime (682 mg, 4.52 mmol, ~90%) was used in the following step without additional drying or purification.

The entire amount from the previous step was dissolved in CH_2Cl_2 (10 mL), 4-(dimethylamino)pyridine (DMAP; 55 mg, 0.452 mmol, 0.1 equiv) and *N*-phenyl-bis(trifluoromethanesulfonimide) (1.78 g, 4.97 mmol, 1.1 equiv) were added, the reaction mixture was cooled in ice-water bath followed by addition of triethylamine (1.3 mL, 9.44 mmol, ~2 equiv). The reaction mixture was allowed to warm up to rt and left stirring for 1.5 h. It was then evaporated on silica and the product was isolated by flash column chromatography (40 g BGB

Scorpius silica 50 µm cartridge, gradient 5% to 60% CH_2Cl_2/hexane) and dried *in vacuo* to give 1.11 g (79% over two steps) of **10a** as colorless oil.

^{1}H NMR (400 MHz, $CDCl_3$): δ 8.26 (s, 1H), 7.92 (app.dd, *J* = 7.7, 1.9 Hz, 1H), 7.45 (ddd, *J* = 8.2, 7.4, 1.9 Hz, 1H), 7.39 (tdd, *J* = 7.4, 1.3, 0.6 Hz, 1H), 7.32 (dd, *J* = 8.1, 1.3 Hz, 1H), 4.02 (s, 3H).
^{19}F NMR (376 MHz, $CDCl_3$): δ -73.28.
^{13}C NMR (101 MHz, $CDCl_3$): δ 147.3, 142.2, 131.3, 128.6, 128.3, 125.8, 122.1, 118.7 (d, *J* = 320.4 Hz), 62.7.
HRMS ($C_9H_8F_3NO_4S$): *m/z* (positive mode) = 284.0201 (found $[M+H]^+$), 284.0199 (calc.).

10b

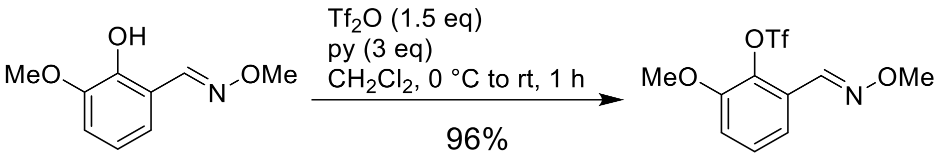

10b

Trifluoromethanesulfonic anhydride (1 mL, 5.94 mmol, 1.5 equiv) was added quickly dropwise to a stirred solution of *o*-vanillin *O*-methyloxime (717 mg, 3.96 mmol; known compound[S19]) and pyridine (1 mL, 12.4 mmol, ~3 equiv) in dry CH_2Cl_2 (15 mL), cooled in ice-water bath. The resulting light orange-yellow solution was allowed to warm up and left stirring at rt for 1 h. The mixture was then diluted with 0.2 N HCl (100 mL), extracted with CH_2Cl_2 (3×25 mL), the combined extracts were washed with brine (50 mL) and dried over Na_2SO_4. The product was isolated by flash column chromatography (25 g Interchim SiHP 30 µm cartridge, gradient 10% to 60% CH_2Cl_2/hexane) and dried *in vacuo* to give 1.20 g (96%) of **10b** as colorless oil.

^{1}H NMR (400 MHz, $CDCl_3$): δ 8.23 (s, 1H), 7.46 (dd, *J* = 8.2, 1.5 Hz, 1H), 7.30 (t, *J* = 8.2 Hz, 1H), 7.03 (dd, *J* = 8.2, 1.5 Hz, 1H), 4.01 (s, 3H), 3.92 (s, 3H).
^{19}F NMR (376 MHz, $CDCl_3$): δ -73.02.
^{13}C NMR (101 MHz, $CDCl_3$): δ 151.8, 142.3, 137.0, 128.8, 126.8, 119.0, 118.8 (q, *J* = 320.9 Hz), 113.9, 62.6, 56.4.
HRMS ($C_{10}H_{10}F_3NO_5S$): *m/z* (positive mode) = 314.0305 (found $[M+H]^+$), 314.0305 (calc.).

10g

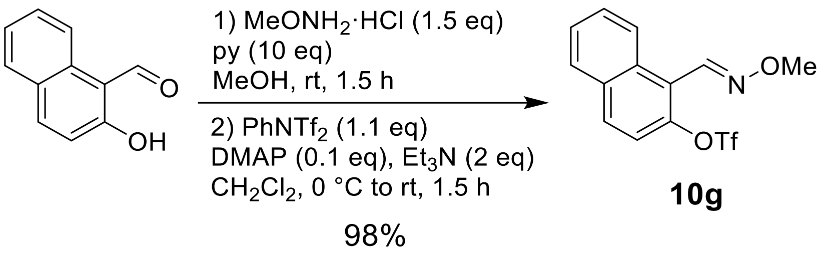

10g

Pyridine (3.2 mL, 40 mmol, 10 equiv) was added to a mixture of 2-hydroxy-1-naphthaldenyde (688 mg, 4 mmol) and *O*-methylhydroxylamine hydrochloride (504 mg, 6 mmol, 1.5 equiv) in methanol (10 mL), and the resulting solution was stirred at rt for 1.5 h. The mixture was then diluted with 0.2 N HCl (150 mL), extracted with EtOAc (3×50 mL), the combined extracts were washed with 0.5 N HCl (100 mL), brine (50 mL) and dried over Na_2SO_4. The filtrate was evaporated, chased with toluene and the residue (viscous yellowish oil) was dried *in vacuo* to give 819 mg (~quant.) of the oxime, which was used in the following step without additional purification.

The entire amount from the previous step was dissolved in CH_2Cl_2 (8 mL), 4-(dimethylamino)pyridine (DMAP; 49 mg, 0.4 mmol, 0.1 equiv) and *N*-phenyl-bis(trifluoromethanesulfonimide) (1.57 g, 4.4 mmol, 1.1 equiv) were added, the reaction mixture was cooled in ice-water bath followed by addition of triethylamine (1.11 mL, 8 mmol, 2 equiv). The reaction mixture was allowed to warm up to rt and left stirring for 1.5 h. It was then evaporated on silica and the product was isolated by flash column chromatography (40 g BGB Scorpius silica 50 µm cartridge, gradient 0% to 30% EtOAc/hexane) and dried *in vacuo* to give 1.30 g (98% over two steps) of **10g** as colorless oil.

^{1}H NMR (400 MHz, $CDCl_3$): δ 8.68 – 8.63 (m, 2H), 7.94 (d, *J* = 9.0 Hz, 1H), 7.94 – 7.86 (m, 1H), 7.66 (ddd, *J* = 8.5, 6.9, 1.5 Hz, 1H), 7.60 (ddd, *J* = 8.1, 6.9, 1.3 Hz, 1H), 7.43 (d, *J* = 9.0 Hz, 1H), 4.13 (s, 3H).
^{19}F NMR (376 MHz, $CDCl_3$): δ -73.43.
^{13}C NMR (101 MHz, $CDCl_3$): δ 146.0, 142.8, 132.8, 132.1, 131.5, 128.7, 127.5, 126.4, 121.2, 119.7, 118.8 (q, *J* = 320.6 Hz), 62.8.
HRMS ($C_{13}H_{10}F_3NO_4S$): *m/z* (positive mode) = 334.0356 (found $[M+H]^+$), 334.0355 (calc.).

10h

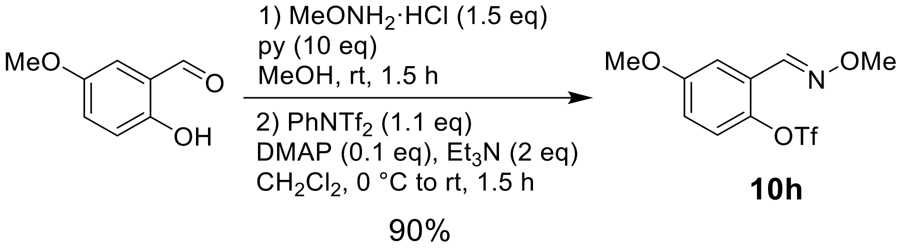

Pyridine (3.2 mL, 40 mmol, 10 equiv) was added to a mixture of 2-hydroxy-5-methoxybenzaldenyde (608 mg, 4 mmol) and *O*-methylhydroxylamine hydrochloride (504 mg, 6 mmol, 1.5 equiv) in methanol (10 mL), and the resulting solution was stirred at rt for 1.5 h. The mixture was then diluted with 0.2 N HCl (150 mL), extracted with EtOAc (3×50 mL), the combined extracts were washed with 0.5 N HCl (100 mL), brine (50 mL) and dried over Na_2SO_4. The filtrate was evaporated, chased with toluene, and the residue was dried *in vacuo* to give 713 mg (~quant.) of the oxime, which was used in the following step without additional purification.

The entire amount from the previous step was dissolved in CH_2Cl_2 (8 mL), 4-(dimethylamino)pyridine (DMAP; 49 mg, 0.4 mmol, 0.1 equiv) and *N*-phenyl-bis(trifluoromethanesulfonimide) (1.57 g, 4.4 mmol, 1.1 equiv) were added, the reaction mixture was cooled in ice-water bath followed by addition of triethylamine (1.11 mL, 8 mmol, 2 equiv). The reaction mixture was allowed to warm up to rt and left stirring for 1.5 h. It was then evaporated on silica and the product was isolated by flash column chromatography (40 g BGB Scorpius silica 50 µm cartridge, gradient 0% to 40% EtOAc/hexane) and dried *in vacuo* to give 1.12 g (90% over two steps) of **10h** as colorless oil.

^{1}H NMR (400 MHz, $CDCl_3$): δ 8.21 (s, 1H), 7.38 (d, *J* = 3.2 Hz, 1H), 7.22 (d, *J* = 9.1 Hz, 1H), 6.95 (dd, *J* = 9.1, 3.2 Hz, 1H), 4.02 (s, 3H), 3.85 (s, 3H).
^{19}F NMR (376 MHz, $CDCl_3$): δ -73.22.
^{13}C NMR (101 MHz, $CDCl_3$): δ 159.0, 142.2, 140.9, 126.7, 123.2, 118.7 (q, *J* = 320.6 Hz), 117.7, 111.2, 62.7, 56.0.
HRMS ($C_{10}H_{10}F_3NO_5S$): *m/z* (positive mode) = 314.0305 (found $[M+H]^+$), 314.0305 (calc.).

10j

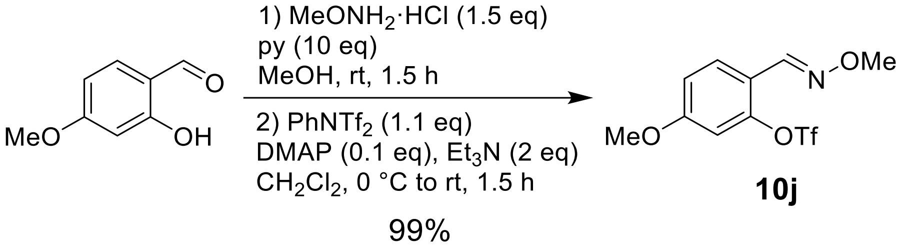

10j

Pyridine (3.2 mL, 40 mmol, 10 equiv) was added to a mixture of 2-hydroxy-4-methoxybenzaldenyde (608 mg, 4 mmol) and *O*-methylhydroxylamine hydrochloride (504 mg, 6 mmol, 1.5 equiv) in methanol (10 mL), and the resulting solution was stirred at rt for 1.5 h. The mixture was then diluted with 0.2 N HCl (150 mL), extracted with EtOAc (3×50 mL), the combined extracts were washed with 0.5 N HCl (100 mL), brine (50 mL) and dried over Na_2SO_4. The filtrate was evaporated, chased with toluene, and the residue was dried *in vacuo* to give the crude oxime, which was used in the following step without additional purification.

The entire amount from the previous step was dissolved in CH_2Cl_2 (8 mL), 4-(dimethylamino)pyridine (DMAP; 49 mg, 0.4 mmol, 0.1 equiv) and *N*-phenyl-bis(trifluoromethanesulfonimide) (1.57 g, 4.4 mmol, 1.1 equiv) were added, the reaction mixture was cooled in ice-water bath followed by addition of triethylamine (1.11 mL, 8 mmol, 2 equiv). The reaction mixture was allowed to warm up to rt and left stirring for 1.5 h. It was then evaporated on silica and the product was isolated by flash column chromatography (40 g BGB Scorpius silica 50 µm cartridge, gradient 0% to 40% EtOAc/hexane) and dried *in vacuo* to give 1.24 g (99% over two steps) of **10j** as colorless oil.

^{1}H NMR (400 MHz, $CDCl_3$): δ 8.18 (s, 1H), 7.83 (d, *J* = 8.9 Hz, 1H), 6.93 (dd, *J* = 8.9, 2.5 Hz, 1H), 6.82 (d, *J* = 2.5 Hz, 1H), 3.99 (s, 3H), 3.85 (s, 3H).
^{19}F NMR (376 MHz, $CDCl_3$): δ -73.22.
^{13}C NMR (101 MHz, $CDCl_3$): δ 161.8, 147.9, 142.0, 128.9, 118.7 (q, *J* = 320.6 Hz), 118.0, 114.9, 107.7, 62.4, 56.0.
HRMS ($C_{10}H_{10}F_3NO_5S$): *m/z* (positive mode) = 314.0310 (found $[M+H]^+$), 314.0305 (calc.).

10k

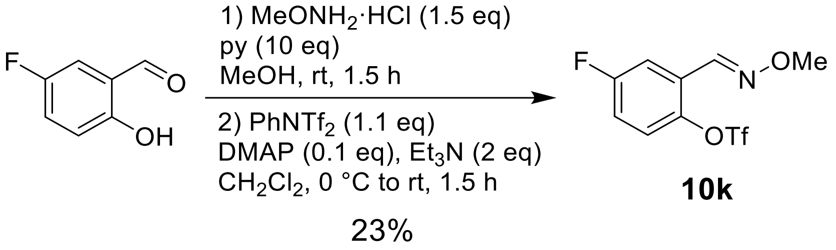

10k

Pyridine (3.2 mL, 40 mmol, 10 equiv) was added to a mixture of 5-fluorosalicylaldehyde (560 mg, 4 mmol) and *O*-methylhydroxylamine hydrochloride (504 mg, 6 mmol, 1.5 equiv) in methanol (10 mL), and the resulting solution was stirred at rt for 1.5 h. The mixture was then diluted with 0.2 N HCl (150 mL), extracted with EtOAc (3×50 mL), the combined extracts were washed with 0.5 N HCl (100 mL), brine (50 mL) and dried over Na_2SO_4. The filtrate was evaporated, chased with toluene, and the residue was dried *in vacuo* to give the crude oxime, which was used in the following step without additional purification.

The entire amount from the previous step was dissolved in CH_2Cl_2 (8 mL), 4-(dimethylamino)pyridine (DMAP; 49 mg, 0.4 mmol, 0.1 equiv) and *N*-phenyl-bis(trifluoromethanesulfonimide) (1.57 g, 4.4 mmol, 1.1 equiv) were added, the reaction mixture was cooled in ice-water bath followed by addition of triethylamine (1.11 mL, 8 mmol, 2 equiv). The reaction mixture was allowed to warm up to rt and left stirring for 1.5 h. It was then evaporated on silica and the mixture was separated by flash column chromatography (40 g BGB Scorpius silica 50 µm cartridge, gradient 0% to 20% EtOAc/hexane); fractions containing the product were pooled, evaporated and the crude material was repurified (25 g Interchim SiHP 30 µm cartridge, gradient 0% to 20% CH_2Cl_2/hexane) to give 277 mg (23% over two steps) of **10k** as colorless oil.

^{1}H NMR (400 MHz, $CDCl_3$): δ 8.20 (d, *J* = 1.6 Hz, 1H), 7.64 (dd, *J* = 8.8, 3.2 Hz, 1H), 7.30 (dd, *J* = 9.1, 4.4 Hz, 1H), 7.13 (ddd, *J* = 9.1, 7.2, 3.2 Hz, 1H), 4.03 (s, 3H).
^{19}F NMR (376 MHz, $CDCl_3$): δ -73.12, -111.47.
^{13}C NMR (101 MHz, $CDCl_3$): δ 161.5 (d, *J* = 249.3 Hz), 142.9 (d, *J* = 3.0 Hz), 141.1 (d, *J* = 2.5 Hz), 127.9 (d, *J* = 8.8 Hz), 123.9 (d, *J* = 8.9 Hz), 118.7 (q, *J* = 320.6 Hz), 118.2 (d, *J* = 24.6 Hz), 114.3 (d, *J* = 25.6 Hz), 62.9.
HRMS ($C_9H_7F_4NO_4S$): *m/z* (positive mode) = 302.0104 (found $[M+H]^+$), 302.0105 (calc.).

12a

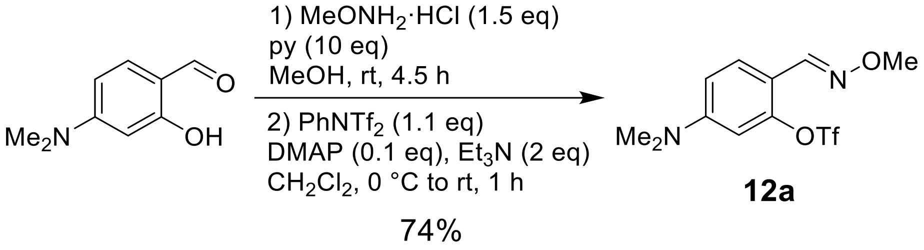

Pyridine (3.2 mL, 40 mmol, 10 equiv) was added to a mixture of 4-(dimethylamino)-2-hydroxybenzaldenyde (495 mg, 4 mmol) and *O*-methylhydroxylamine hydrochloride (504 mg, 6 mmol, 1.5 equiv) in methanol (7 mL), and the resulting solution was stirred at rt for 4.5 h. The mixture was then diluted with 0.2 N HCl (60 mL), extracted with EtOAc (3×25 mL), the combined extracts were washed with brine (50 mL) and dried over Na_2SO_4. The solution was filtered through a 3 cm plug of silica, eluting all product with EtOAc, the filtrate was evaporated, chased with toluene and the residue (orange oil) was dried *in vacuo* to give 590 mg (~3 mmol) of the oxime, which was used directly.

The entire amount from the previous step was dissolved in CH_2Cl_2 (6 mL), 4-(dimethylamino)pyridine (DMAP; 37 mg, 0.3 mmol, 0.1 equiv) and *N*-phenyl-bis(trifluoromethanesulfonimide) (1.19 g, 3.35 mmol, 1.1 equiv) were added, the reaction mixture was cooled in ice-water bath followed by addition of triethylamine (0.85 mL, 6.08 mmol, 2 equiv). The reaction mixture was allowed to warm up to rt and left stirring for 1 h. It was then evaporated on silica and the product was isolated by flash column chromatography (25 g Interchim SiHP 30 µm cartridge, gradient 5% to 50% EtOAc/hexane) and freeze-dried from 1,4-dioxane to give 967 mg (74% over two steps) of **12a** as white solid.

mp (EtOAc/hexane): 61-62 °C.

^{1}H NMR (400 MHz, $CDCl_3$): δ 8.14 (s, 1H), 7.71 (d, *J* = 9.0 Hz, 1H), 6.64 (dd, *J* = 9.0, 2.5 Hz, 1H), 6.47 (d, *J* = 2.5 Hz, 1H), 3.96 (s, 3H), 3.02 (s, 6H).

^{19}F NMR (376 MHz, $CDCl_3$): δ -73.31.

^{13}C NMR (101 MHz, $CDCl_3$): δ 152.2, 148.7, 142.8, 128.6, 118.8 (q, *J* = 320.5 Hz), 112.1, 111.9, 104.1, 62.2, 40.2.

HRMS ($C_{11}H_{13}F_3N_2O_4S$): *m/z* (positive mode) = 327.0622 (found $[M+H]^+$), 327.0621 (calc.).

12b

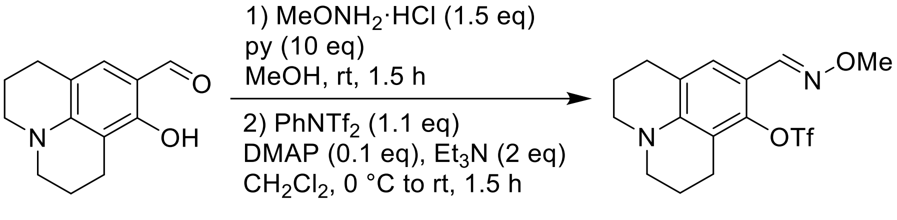

12b

Pyridine (3.2 mL, 40 mmol, 10 equiv) was added to a mixture of 8-hydroxyjulolidine-9-carboxaldehyde (868 mg, 4 mmol) and *O*-methylhydroxylamine hydrochloride (504 mg, 6 mmol, 1.5 equiv) in methanol (10 mL), and the resulting solution was stirred at rt for 1.5 h. The mixture was then diluted with 0.2 N HCl (150 mL), extracted with EtOAc (3×30 mL), the combined extracts were washed with 0.2 N HCl (100 mL), brine (50 mL) and dried over Na_2SO_4. The filtrate was evaporated, chased with toluene and the residue (viscous light purple oil) was dried *in vacuo* to give 1.05 g of crude oxime, which was used directly in the following step.

The entire amount was dissolved in CH_2Cl_2 (8 mL), 4-(dimethylamino)pyridine (DMAP; 49 mg, 0.4 mmol, 0.1 equiv) and *N*-phenyl-bis(trifluoromethanesulfonimide) (1.57 g, 4.4 mmol, 1.1 equiv) were added, the reaction mixture was cooled in ice-water bath followed by addition of triethylamine (1.11 mL, 8 mmol, 2 equiv). The reaction mixture was allowed to warm up to rt and left stirring for 1.5 h. It was then evaporated on silica and the product was isolated by flash column chromatography (40 g BGB Scorpius 50 µm cartridge, gradient 0% to 50% EtOAc/hexane) and freeze-dried from 1,4-dioxane to give 1.03 g (68% over two steps) of **12b** as white solid.

mp (EtOAc/hexane): 69-70 °C.
^{1}H NMR (400 MHz, $CDCl_3$): δ 8.13 (s, 1H), 7.34 (s, 1H), 3.93 (s, 3H), 3.23 (td, *J* = 5.7, 1.7 Hz, 4H), 2.82 – 2.69 (m, 4H), 1.99 – 1.88 (m, 4H).
^{19}F NMR (376 MHz, $CDCl_3$): δ -73.23.
^{13}C NMR (101 MHz, $CDCl_3$): δ 145.4, 144.0, 143.9, 124.9, 121.5, 118.7 (q, *J* = 320.2 Hz), 113.7, 111.5, 62.0, 49.9, 49.4, 27.7, 22.5, 21.3, 20.8.
HRMS ($C_{15}H_{17}F_3N_2O_4S$): *m/z* (positive mode) = 379.0931 (found $[M+H]^+$), 379.0934 (calc.).

Preparation of 3-unsubstituted benzothiophene 1,1-dioxides 6a, 14a,b

Method A (from arylimine 9)

5a

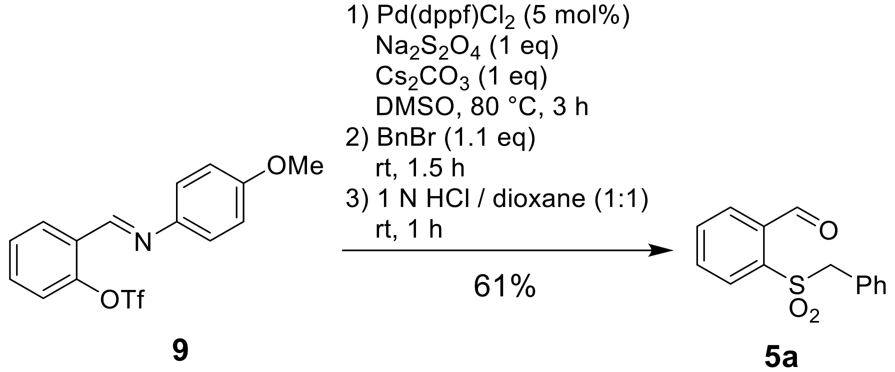

A 10 mL microwave vial (Biotage) was loaded with compound **9** (180 mg, 0.5 mmol), Pd(dppf)Cl_2·CH_2Cl_2 catalyst (20 mg, 0.05 mmol, 5 mol%), sodium dithionite (87 mg, 0.5 mmol, 1 equiv.) and cesium carbonate (163 mg, 0.5 mmol, 1 equiv.). Dry DMSO solvent (2 mL) was added, the reaction mixture was degassed on a Schlenk line, placed in a preheated 80 °C silicon oil bath and stirred for 3 h. Upon cooling down to rt, benzyl bromide (66 µL, 0.55 mmol, 1.1 equiv.) was injected and the mixture was stirred at rt for further 1.5 h. The reaction mixture was diluted with 1 N HCl (50 mL), extracted with CH_2Cl_2 (3×20 mL) and the combined extracts were dried over Na_2SO_4. The product was isolated by flash column chromatography (12 g Interchim SiHP 30 µm cartridge, gradient 10% to 60% EtOAc/hexane) as viscous yellow oil (105 mg, ~2:1 mixture of **5a** and its imine with *p*-anisidine). This material was dissolved in dioxane (2 mL) and 1 N HCl (2 mL), the mixture was stirred at rt for 1 h, then diluted with 1 N HCl (50 mL) and extracted with CH_2Cl_2 (3×20 mL). The combined extracts were washed with 1 N HCl (50 mL) and brine, dried over Na_2SO_4, filtered through a 2 cm plug of silica, washing with 20% EtOAc – CH_2Cl_2 (50 mL). The filtrate was evaporated and the residue was freeze-dried from 1,4-dioxane to give 79 mg (61%) of **5a** as white solid.

mp (EtOAc/hexane): 89-90 °C.
^{1}H NMR (400 MHz, DMSO-*d*$_6$): δ 8.55 – 8.48 (m, 2H), 7.95 (d, *J* = 8.8 Hz, 1H), 7.56 (br.s, 2H), 7.54 – 7.48 (m, 2H), 7.06 (d, *J* = 2.4 Hz, 1H), 6.95 (dd, *J* = 8.8, 2.5 Hz, 1H), 3.06 (s, 6H).
^{13}C NMR (101 MHz, DMSO-*d*$_6$): δ 152.4, 149.7, 146.2, 140.7, 137.8, 122.9, 119.2, 114.5, 114.4, 102.3, 98.7, 66.4, 40.0.
HRMS ($C_{15}H_{15}N_3O_2S$): *m/z* (positive mode) = 302.0955 (found $[M+H]^+$), 302.0958 (calc.).

Method B (via N,N-dimethylhydrazone 8)

8

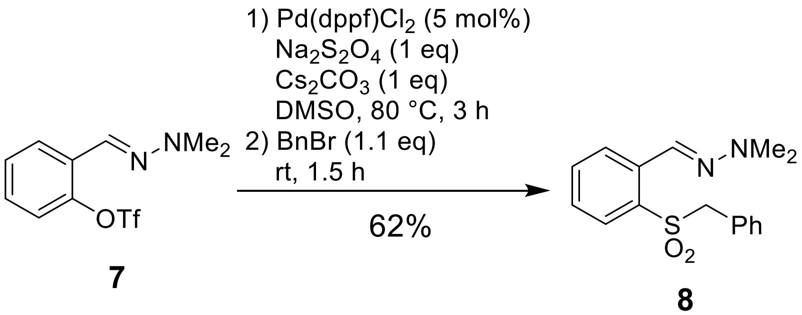

7 **8**

A 10 mL microwave vial (Biotage) was loaded with compound **7** (148 mg, 0.5 mmol), Pd(dppf)Cl_2·CH_2Cl_2 catalyst (20 mg, 0.025 mmol, 5 mol%), sodium dithionite (87 mg, 0.5 mmol, 1 equiv.) and cesium carbonate (163 mg, 0.5 mmol, 1 equiv.). Dry DMSO solvent (2 mL) was added, the reaction mixture was degassed on a Schlenk line, placed in a preheated 80 °C silicon oil bath and stirred for 3 h. Upon cooling down to rt, benzyl bromide (65 µL, 1.1 mmol, 1.1 equiv) was injected and the mixture was stirred at rt for further 1.5 h. The reaction mixture was diluted with brine (50 mL), extracted with CH_2Cl_2 (3×25 mL) and the combined extracts were dried over Na_2SO_4. The product was isolated by flash column chromatography (12 g Interchim SiHP 30 µm cartridge, gradient 10% to 80% EtOAc/hexane) and dried *in vacuo* to give 93 mg (62%) of **8** as white solid.

mp (EtOAc/hexane): 101-102 °C.

^{1}H NMR (400 MHz, $CDCl_3$): δ 8.06 (dd, *J* = 8.0, 1.3 Hz, 1H), 7.82 (s, 1H), 7.70 – 7.63 (m, 1H), 7.50 (dddd, *J* = 8.0, 7.2, 1.3, 0.7 Hz, 1H), 7.31 – 7.16 (m, 4H), 7.10 – 7.03 (m, 2H), 4.33 (s, 2H), 3.03 (s, 6H).

^{13}C NMR (101 MHz, $CDCl_3$): δ 137.3, 133.7, 133.2, 131.0, 130.6, 128.8, 128.7, 128.1, 126.8, 126.6, 126.3, 62.7, 42.8.

HRMS ($C_{16}H_{18}N_2O_2S$): *m/z* (positive mode) = 303.1168 (found $[M+H]^+$), 303.1162 (calc.).

5a

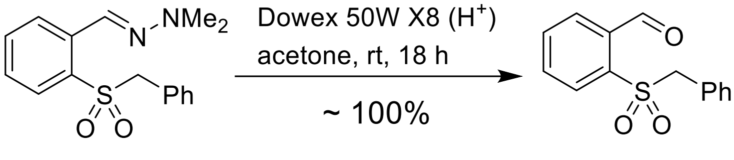

8 **5a**

Following the procedure from [S20], Dowex 50W X8 ion exchange resin (H^+ form, 200-400 mesh) was added to a solution of **AB1-932** (66 mg, 0.22 mmol) in acetone (15 mL), and the reaction mixture was vigorously stirred at rt overnight (18 h). The suspension was then filtered through a 1.5 cm plug of silica, washing with EtOAc (50 mL). The filtrate was evaporated, redissolved in 1,4-dioxane, microfiltered through a syringe filter with 0.22 µm PTFE-hydrophilic membrane and freeze-dried to give 64 mg (~quant. yield, remainder dioxane) of **AB1-934** as white solid; the analytical data match with those of the above prepared sample.

Method C (via O-methyloxime 11a)

11a

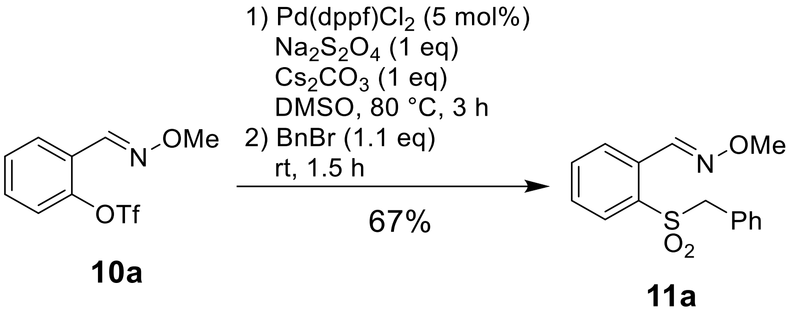

A 25 mL round-bottom flask was loaded with compound **10a** (283 mg, 1 mmol), $Pd(dppf)Cl_2 \cdot CH_2Cl_2$ catalyst (40 mg, 0.05 mmol, 5 mol%), sodium dithionite (174 mg, 1 mmol, 1 equiv.) and cesium carbonate (326 mg, 1 mmol, 1 equiv.). Dry DMSO solvent (4 mL) was added, the reaction mixture was degassed on a Schlenk line, placed in a preheated 80 °C silicon oil bath and stirred for 3 h. Upon cooling down to rt, benzyl bromide (131 µL, 1.1 mmol, 1.1 equiv.) was injected and the mixture was stirred at rt for further 1.5 h. The reaction mixture was diluted with brine (50 mL), extracted with CH_2Cl_2 (3×25 mL) and the combined extracts were dried over Na_2SO_4. The product was isolated by flash column chromatography (25 g Interchim SiHP 30 µm cartridge, gradient 5% to 60% EtOAc/hexane) and dried *in vacuo* to give 193 mg (67%) of **11a** as white solid.

mp (EtOAc/hexane): 71-72 °C.

^1H NMR (400 MHz, $CDCl_3$): δ 8.70 (s, 1H), 7.95 (dd, J = 7.9, 1.3 Hz, 1H), 7.74 (dd, J = 7.9, 1.3 Hz, 1H), 7.57 (tdd, J = 7.9, 1.4, 0.7 Hz, 1H), 7.41 (td, J = 7.7, 1.3 Hz, 1H), 7.33 – 7.27 (m, 1H), 7.27 – 7.21 (m, 2H), 7.07 – 7.02 (m, 2H), 4.32 (s, 2H), 3.98 (s, 3H).

^{13}C NMR (101 MHz, $CDCl_3$): δ 145.6, 135.6, 133.9, 132.5, 131.0, 130.9, 129.7, 129.0, 128.8, 128.0, 127.6, 63.6, 62.5.

HRMS ($C_{15}H_{15}NO_3S$): *m/z* (positive mode) = 290.0848 (found $[M+H]^+$), 290.0845 (calc.).

5a

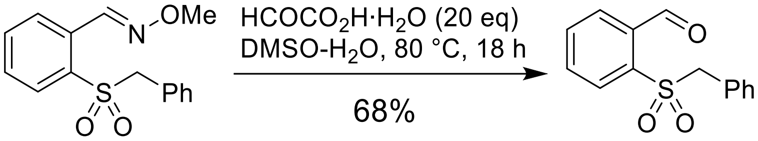

11a **5a**

A solution of glyoxylic acid monohydrate (1.17 g, 12.7 mmol, 20 equiv.) in water (1.2 mL) was added to the stirred solution of **11a** (184 mg, 0.64 mmol) in DMSO (3 mL), and the reaction mixture was stirred at 80 °C overnight (18 h). On cooling, it was diluted with 0.1 N NaOH (50 mL), extracted with EtOAc (3×25 mL), the combined extracts were washed with brine and dried over Na_2SO_4. The filtrate was evaporated on silica, and the product was isolated by flash column chromatography (12 g Interchim SiHP 30 µm cartridge, gradient 10% to 80% EtOAc/hexane) to give 113 mg (68%) of **5a** as white solid; the analytical data match with those of the above prepared sample.

6a

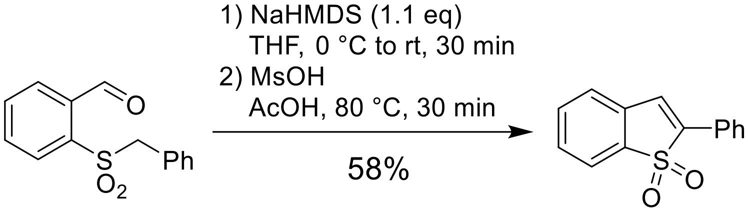

5a **6a**

A solution of NaHMDS (0.21 mL of 2 M in THF, 0.42 mmol, ~1.1 equiv.) was added dropwise to a stirred solution of **5a** (100 mg, 0.385 mmol) in dry THF (2.5 mL), cooled in ice-water bath. The reaction mixture was warmed up to rt and stirred for 30 min, then quenched by addition of methanol (0.5 mL) and evaporated. The residue was resuspended in acetic acid (2 mL), methanesulfonic acid (0.4 mL) was added and the mixture was stirred for 30 min at 80 °C. It was then carefully poured into 50 mL of sat. aq. $NaHCO_3$, extracted with CH_2Cl_2 (3×20 mL), washed with brine and dried over Na_2SO_4. The product was isolated by flash column chromatography (12 g Interchim SiHP 30 µm cartridge, gradient 0% to 50% EtOAc/hexane + 20% CH_2Cl_2 constant additive) and freeze-dried from 1,4-dioxane to give 54 mg (58%) of **6a** as yellowish solid.

mp (EtOAc/hexane): 174-175 °C.

^{1}H NMR (400 MHz, $CDCl_3$): δ 7.86 – 7.80 (m, 2H), 7.76 (dq, J = 7.4, 0.9 Hz, 1H), 7.56 (td, J = 7.5, 1.2 Hz, 1H), 7.52 – 7.42 (m, 4H), 7.40 (dt, J = 7.6, 0.9 Hz, 1H), 7.28 (d, J = 0.9 Hz, 1H).

^{13}C NMR (101 MHz, $CDCl_3$): δ 142.8, 137.2, 133.9, 131.3, 130.5, 130.1, 129.4, 127.3, 126.7, 125.2, 123.8, 121.6.

HRMS ($C_{14}H_{10}O_2S$): *m/z* (positive mode) = 243.0486 (found $[M+H]^+$), 243.0474 (calc.).

13a

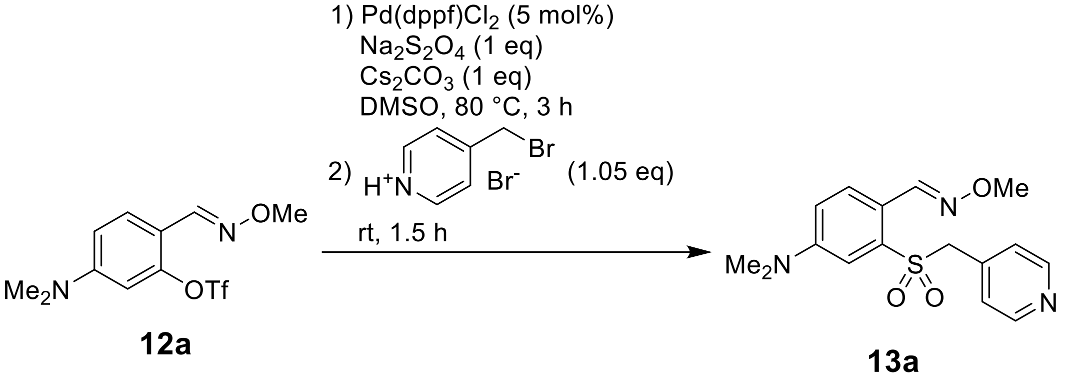

A 25 mL round-bottom flask was loaded with compound **12a** (326 mg, 1 mmol), $Pd(dppf)Cl_2{\cdot}CH_2Cl_2$ catalyst (40 mg, 0.05 mmol, 5 mol%), sodium dithionite (174 mg, 1 mmol, 1 equiv.) and cesium carbonate (326 mg, 1 mmol, 1 equiv.). Dry DMSO solvent (4 mL) was added, the reaction mixture was degassed on a Schlenk line, placed in a preheated 80 °C silicon oil bath and stirred for 3 h. Upon cooling down to rt, 4-(bromomethyl)pyridine hydrobromide (266 mg, 1.05 mmol, 1.05 equiv.; dissolved in 1 mL DMSO) was injected and the mixture was stirred at rt for 1.5 h. The reaction mixture was diluted with brine (50 mL), extracted with CH_2Cl_2 (3×25 mL) and the combined extracts were dried over Na_2SO_4. The product was isolated by flash column chromatography (12 g Interchim SiHP 30 µm cartridge, gradient 20% to 100% EtOAc/CH_2Cl_2) to give 202 mg (61%) of **13a** as light tan solid (purity ~90%).

^{1}H NMR (400 MHz, $CDCl_3$): δ 8.76 (s, 1H), 8.54 – 8.48 (m, 2H), 7.85 (d, J = 8.8 Hz, 1H), 7.07 – 7.02 (m, 2H), 6.86 (d, J = 2.8 Hz, 1H), 6.81 (dd, J = 8.8, 2.8 Hz, 1H), 4.29 (s, 2H), 3.96 (s, 3H), 2.93 (s, 6H).

^{13}C NMR (101 MHz, $CDCl_3$): δ 150.6, 150.1, 145.5, 137.0, 135.8, 129.4, 125.7, 118.2, 116.5, 112.9, 62.4, 62.2, 40.1.

HRMS ($C_{16}H_{19}N_3O_3S$): *m/z* (positive mode) = 334.1220 (found $[M+H]^+$), 334.1220 (calc.).

14a

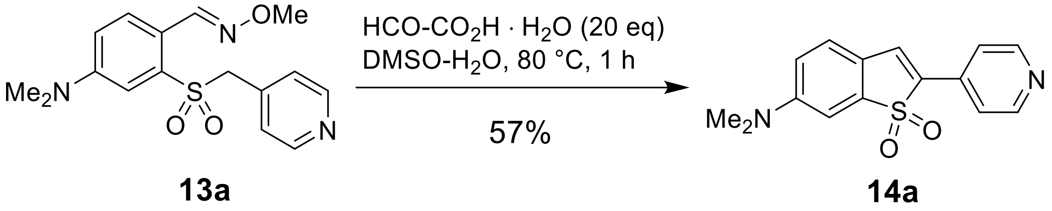

Excess glyoxylic acid monohydrate (552 mg, 6 mmol, 20 equiv) was added to a stirred solution of **13a** (100 mg, 0.3 mmol) in the mixture of DMSO (1.5 mL) and water (0.6 mL), and the reaction mixture was stirred at 80 °C for 1 h (quickly turned intense red). On cooling, the aqueous phase made basic by addition of 0.1 N NaOH and saturated with NaCl, the compound was extracted with CH_2Cl_2 – 2-propanol mixture (the product is poorly soluble in EtOAc and CH_2Cl_2). The combined extracts were dried over Na_2SO_4, filtered and evaporated on neutral alumina; the product was isolated by flash column chromatography (12 g Interchim SiHP 30 µm cartridge, gradient 0% to 100% A/B, A = 10% 2-propanol – CH_2Cl_2, B = CH_2Cl_2) and freeze-dried from 1,4-dioxane to give 49 mg (57%) of **14a** as orange solid.

mp (*i*-PrOH/CH_2Cl_2): > 200 °C (dec.).
^{1}H NMR (400 MHz, DMSO-d_6): δ 8.71 – 8.64 (m, 2H), 8.17 (d, *J* = 0.9 Hz, 1H), 7.70 – 7.65 (m, 2H), 7.46 (d, *J* = 8.6 Hz, 1H), 7.24 (dd, *J* = 2.5, 0.9 Hz, 1H), 6.91 (dd, *J* = 8.6, 2.5 Hz, 1H), 3.07 (s, 6H).
^{13}C NMR (101 MHz, DMSO-d_6): δ 152.3, 150.5, 139.4, 135.0, 133.3, 130.6, 127.5, 118.9, 115.9, 115.2, 104.7, 40.1.
HRMS ($C_{15}H_{14}N_2O_2S$): *m/z* (positive mode) = 287.0850 (found [M+H]$^+$), 287.0849 (calc.).

13b

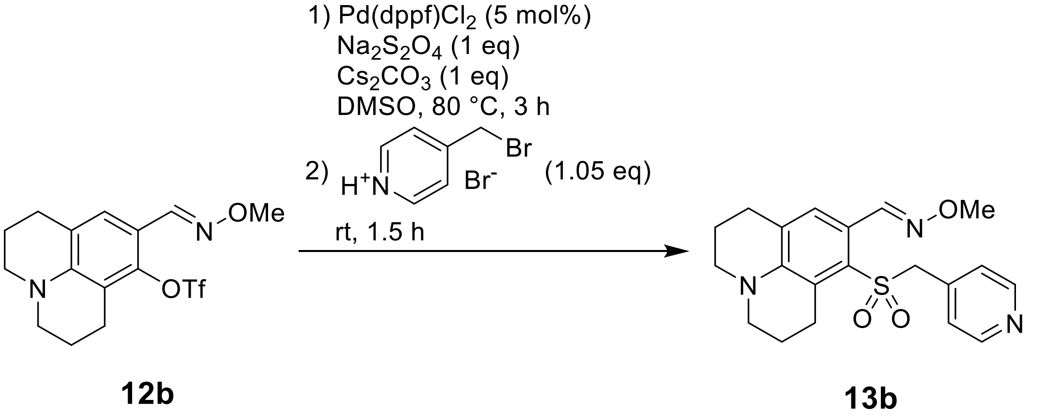

A 10 mL round-bottom flask was loaded with compound **12b** (189 mg, 0.5 mmol), Pd(dppf)Cl_2·CH_2Cl_2 catalyst (20 mg, 0.05 mmol, 5 mol%), sodium dithionite (87 mg, 0.5 mmol, 1 equiv.) and cesium carbonate (163 mg, 0.5 mmol, 1 equiv.). Dry DMSO solvent (2 mL) was added, the reaction mixture was degassed on a Schlenk line, placed in a preheated 80 °C silicon oil bath and stirred for 3 h. Upon cooling down to rt, 4-(bromomethyl)pyridine hydrobromide (133 mg, 0.525 mmol, 1.05 equiv.; dissolved in 0.5 mL DMSO) was injected and the mixture was stirred at rt for 1.5 h. The reaction mixture was diluted with brine (50 mL), extracted with CH_2Cl_2 (3×25 mL) and the combined extracts were dried over Na_2SO_4. The product was isolated by flash column chromatography (12 g Interchim SiHP 30 µm cartridge, gradient 20% to 100% EtOAc/hexane) to give 88 mg (~45%) of **13b** as light orange solid, which was in the following step without further characterization.

14b

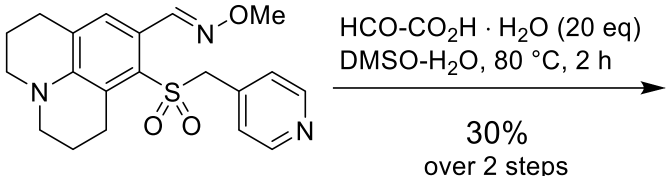

13b **14b**

Excess glyoxylic acid monohydrate (920 mg, 10 mmol, 20 equiv) was added to a stirred solution of crude **13b** (prepared from 0.5 mmol **12b** as described above) in the mixture of DMSO (2.5 mL) and water (1 mL), and the reaction mixture was stirred at 80 °C for 2 h (quickly turned violet). On cooling, the aqueous phase made basic by addition of 0.1 N NaOH, the product was extracted with EtOAc (3×25 mL), washed with brine and dried over Na_2SO_4. The filtrate was evaporated on neutral alumina, the product was isolated by flash column chromatography (12 g Interchim SiHP 30 µm cartridge, gradient 0% to 100% A/B, A = 10% 2-propanol – CH_2Cl_2, B = CH_2Cl_2) and freeze-dried from 1,4-dioxane to give 51 mg (30% over 2 steps) of **14b** as orange solid.

mp (*i*-PrOH/CH_2Cl_2): > 200 °C (dec.).
^{1}H NMR (400 MHz, DMSO-d_6): δ 8.69 – 8.60 (m, 2H), 8.03 (s, 1H), 7.68 – 7.58 (m, 2H), 6.98 (s, 1H), 3.32 – 3.23 (m, 4H), 3.03 (t, J = 6.4 Hz, 2H), 2.72 (t, J = 6.3 Hz, 2H), 1.98 – 1.80 (m, 4H).
^{13}C NMR (101 MHz, DMSO-d_6): δ 150.4, 145.2, 135.1, 133.6, 132.2, 130.8, 124.9, 124.3, 118.7, 117.9, 115.2, 49.6, 48.8, 27.4, 21.8, 20.3, 19.7.
HRMS ($C_{19}H_{18}N_2O_2S$): *m/z* (positive mode) = 339.1162 (found $[M+H]^+$), 339.1162 (calc.).

Preparation of 3-aminobenzothiophene 1,1-dioxides 15

15a

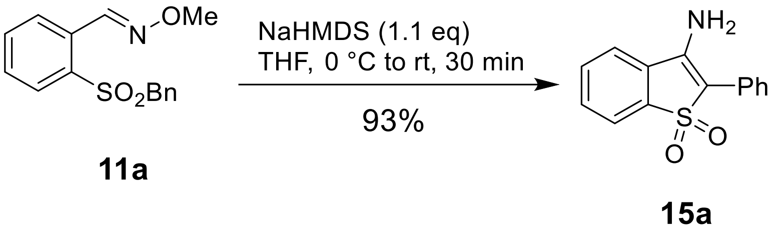

A solution of NaHMDS (0.25 mL of 2 M in THF, 0.25 mmol, ~1.1 equiv.) was added dropwise to a stirred solution of **11a** (131 mg, 0.45 mmol) in dry THF (3 mL), cooled in ice-water bath. The reaction mixture was warmed up to rt and stirred for 30 min, then quenched by addition of brine (50 mL) and acetic acid (0.5 mL). The product was extracted with CH_2Cl_2 (3×25 mL), the combined organic layers were washed with brine, pyridine (0.5 mL) was added and the solution was dried over Na_2SO_4. The filtrate was evaporated on silica, the product was isolated by flash column chromatography (12 g Interchim SiHP 30 µm cartridge, gradient 0% to 20% EtOAc/CH_2Cl_2) and freeze-dried from 1,4-dioxane to give 108 mg (93%) of **15a** as light-yellow solid.

^{1}H NMR (400 MHz, DMSO-d_6): δ 8.09 (d, J = 7.8 Hz, 1H), 7.83 (d, J = 7.6 Hz, 1H), 7.75 (td, J = 7.6, 1.2 Hz, 1H), 7.69 – 7.60 (m, 3H), 7.53 – 7.45 (m, 2H), 7.37 – 7.30 (m, 1H), 7.17 (br.s, 2H).
^{13}C NMR (101 MHz, DMSO-d_6): δ 141.9, 138.2, 133.0, 130.8, 129.4, 129.0, 128.9, 127.5, 127.0, 121.4, 119.8, 103.5.
HRMS ($C_{14}H_{11}NO_2S$): m/z (positive mode) = 258.0584 (found $[M+H]^+$), 258.0583 (calc.).

11b

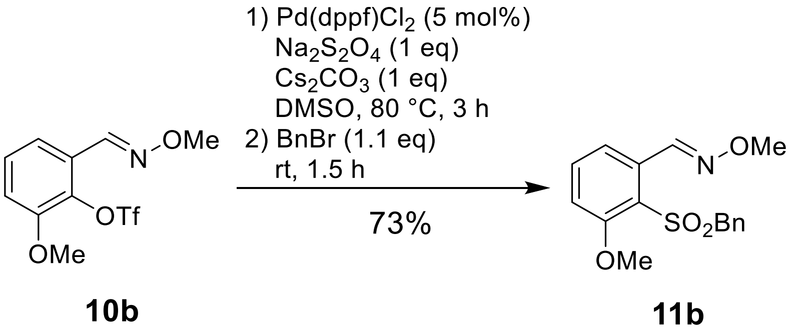

A 10 mL microwave vial (Biotage) was loaded with compound **10b** (157 mg, 0.5 mmol), Pd(dppf)Cl_2·CH_2Cl_2 catalyst (20 mg, 0.025 mmol, 5 mol%), sodium dithionite (87 mg, 0.5 mmol,

1 equiv.) and cesium carbonate (163 mg, 0.5 mmol, 1 equiv.). Dry DMSO solvent (2 mL) was added, the reaction mixture was degassed on a Schlenk line, placed in a preheated 80 °C silicon oil bath and stirred for 3 h. Upon cooling down to rt, benzyl bromide (65 µL, 0.55 mmol, 1.1 equiv.) was injected and the mixture was stirred at rt for further 1.5 h. The reaction mixture was diluted with brine (50 mL), extracted with CH_2Cl_2 (3×20 mL) and the combined extracts were dried over Na_2SO_4. The product was isolated by flash column chromatography (12 g Interchim SiHP 30 µm cartridge, gradient 20% to 80% EtOAc/hexane) and dried *in vacuo* to give 117 mg (73%) of **11b** as white solid.

^{1}H NMR (400 MHz, $CDCl_3$): δ 8.34 (s, 1H), 7.49 (ddd, *J* = 8.3, 7.7, 0.6 Hz, 1H), 7.27 – 7.20 (m, 4H), 7.16 (dq, *J* = 6.6, 2.3 Hz, 2H), 7.11 (dd, *J* = 8.3, 1.1 Hz, 1H), 4.59 (s, 2H), 4.07 (s, 3H), 3.88 (s, 3H).
^{13}C NMR (101 MHz, $CDCl_3$): δ 158.6, 147.8, 135.5, 134.7, 130.9, 129.0, 128.7, 128.2, 124.9, 122.2, 114.0, 62.11, 62.05, 57.3.
HRMS ($C_{16}H_{17}NO_4S$): *m/z* (positive mode) = 320.0955 (found $[M+H]^+$), 320.0951 (calc.).

15b

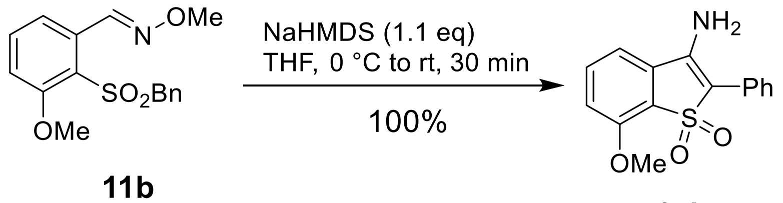

11b **15b**

A solution of NaHMDS (0.19 mL of 2 M in THF, 0.38 mmol, ~1.1 equiv.) was added dropwise to a stirred solution of **11b** (113 mg, 0.35 mmol) in dry THF (2 mL), cooled in ice-water bath. The reaction mixture was warmed up to rt and stirred for 30 min, then quenched by addition of brine (50 mL) and acetic acid (2 mL). The product was extracted with EtOAc (3×25 mL), the combined organic layers were washed with brine, dried over Na_2SO_4, and the product was isolated by flash column chromatography (12 g Interchim SiHP 30 µm cartridge, gradient 50% to 100% EtOAc/hexane) and freeze-dried from 1,4-dioxane to give 104 mg (quant., remainder dioxane) of **15b** as yellow solid.

mp (EtOAc/CH_2Cl_2): 213-214 °C.
^{1}H NMR (400 MHz, DMSO-d_6): δ 7.72 – 7.62 (m, 2H), 7.62 – 7.58 (m, 2H), 7.50 – 7.44 (m, 2H), 7.35 – 7.28 (m, 2H), 6.99 (br.s, 2H), 3.95 (s, 3H).

^{13}C NMR (101 MHz, DMSO-d_6): δ 153.5, 141.1, 134.9, 131.5, 129.0, 128.9, 127.6, 126.9, 123.4, 114.6, 113.2, 104.6, 56.3.
HRMS ($C_{15}H_{13}NO_3S$): *m/z* (positive mode) = 288.0688 (found $[M+H]^+$), 288.0689 (calc.).

11c

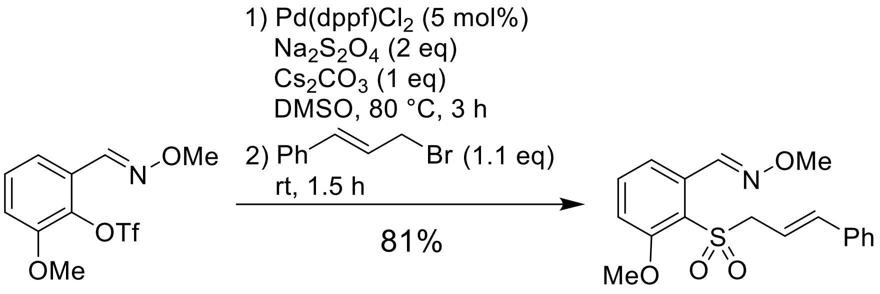

10b **11c**

A 10 mL microwave vial (Biotage) was loaded with compound **10b** (157 mg, 0.5 mmol), $Pd(dppf)Cl_2 \cdot CH_2Cl_2$ catalyst (20 mg, 0.025 mmol, 5 mol%), sodium dithionite (174 mg, 1 mmol, 2 equiv.) and cesium carbonate (163 mg, 0.5 mmol, 1 equiv.). Dry DMSO solvent (2 mL) was added, the reaction mixture was degassed on a Schlenk line, placed in a preheated 80 °C silicon oil bath and stirred for 3 h. Upon cooling down to rt, cinnamyl bromide (108 mg, 0.55 mmol, 1.1 equiv., dissolved in 0.5 mL DMSO) was injected and the mixture was stirred at rt for further 1.5 h. The reaction mixture was diluted with brine (50 mL), extracted with CH_2Cl_2 (3×20 mL) and the combined extracts were dried over Na_2SO_4. The product was isolated by flash column chromatography (12 g Interchim SiHP 30 µm cartridge, gradient 20% to 80% EtOAc/hexane) and dried *in vacuo* to give 140 mg (81%) of **11c** as white solid.

^1H NMR (400 MHz, $CDCl_3$): δ 8.92 (s, 1H), 7.52 (ddd, *J* = 8.3, 7.8, 0.6 Hz, 1H), 7.38 (ddd, *J* = 7.8, 1.3, 0.6 Hz, 1H), 7.31 – 7.20 (m, 5H), 7.11 (dd, *J* = 8.3, 1.2 Hz, 1H), 6.50 (dt, *J* = 15.7, 1.2 Hz, 1H), 6.09 (dt, *J* = 15.7, 7.7 Hz, 1H), 4.25 (dd, *J* = 7.7, 1.2 Hz, 2H), 4.03 (s, 3H), 3.92 (s, 3H).
^{13}C NMR (101 MHz, $CDCl_3$): δ 158.4, 147.9, 139.3, 136.0, 135.3, 134.7, 128.7, 128.6, 126.8, 125.5, 122.4, 115.3, 114.0, 62.2, 60.2, 57.2.
HRMS ($C_{18}H_{19}NO_4S$): *m/z* (positive mode) = 346.1111 (found $[M+H]^+$), 346.1108 (calc.).

15c

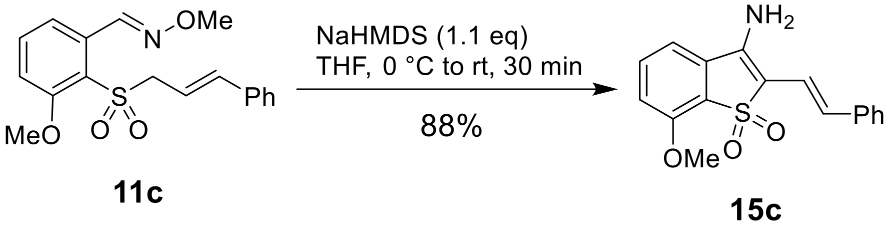

A solution of NaHMDS (0.22 mL of 2 M in THF, 0.44 mmol, ~1.1 equiv.) was added dropwise to a stirred solution of **11c** (140 mg, 0.41 mmol) in dry THF (2 mL), cooled in ice-water bath. The reaction mixture was warmed up to rt and stirred for 30 min, then quenched by addition of brine (50 mL) and acetic acid (2 mL). The product was extracted with EtOAc (3×30 mL), the combined organic layers were washed with brine, dried over Na_2SO_4, and the product was isolated by flash column chromatography (12 g Interchim SiHP 30 µm cartridge, gradient 50% to 100% EtOAc/hexane) and freeze-dried from 1,4-dioxane to give 144 mg of **15c** as bright yellow solid, containing 1 mol dioxane/mol compound (88% yield).

mp (EtOAc/hexane): > 250 °C (dec.).

^{1}H NMR (400 MHz, DMSO-d_6): δ 7.69 (dd, J = 8.5, 7.5 Hz, 1H), 7.55 – 7.48 (m, 3H), 7.39 – 7.27 (m, 6H), 7.24 – 7.18 (m, 1H), 6.67 (d, J = 16.4 Hz, 1H), 3.95 (s, 3H).

^{13}C NMR (101 MHz, DMSO-d_6): δ 153.6, 141.6, 137.8, 135.1, 131.2, 128.7, 126.9, 125.6, 124.4, 122.8, 115.2, 114.7, 113.1, 105.6, 56.3.

HRMS ($C_{17}H_{15}NO_3S$): m/z (positive mode) = 314.0848 (found [M+H]$^+$), 314.0845 (calc.).

15d

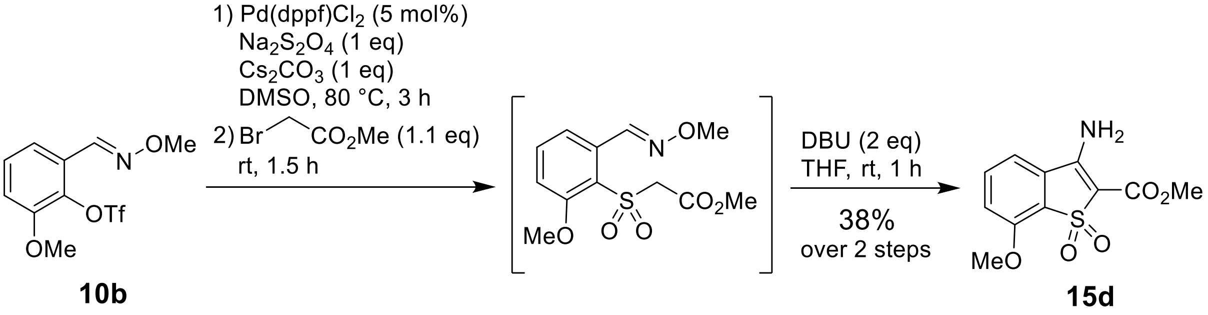

A 10 mL microwave vial (Biotage) was loaded with compound **10b** (189 mg, 0.6 mmol), Pd(dppf)Cl_2·CH_2Cl_2 catalyst (25 mg, 0.03 mmol, 5 mol%), sodium dithionite (104 mg, 0.6 mmol, 1 equiv.) and cesium carbonate (196 mg, 0.6 mmol, 1 equiv.). Dry DMSO solvent (2.4 mL) was added, the reaction mixture was degassed on a Schlenk line, placed in a preheated 80 °C silicon oil bath and stirred for 3 h. Upon cooling down to rt, methyl bromoacetate (63 µL, 0.66 mmol,

1.1 equiv.) was injected and the mixture was stirred at rt for 1.5 h. The reaction mixture was diluted with brine (50 mL), extracted with CH_2Cl_2 (3×20 mL) and the combined extracts were dried over Na_2SO_4. The intermediate was isolated by flash column chromatography (12 g Interchim SiHP 30 µm cartridge, gradient 20% to 100% EtOAc/hexane) to give an impure material (160 mg of viscous light-yellow oil) which was used directly in the following step.

The crude intermediate was dissolved in dry THF (3 mL), DBU (0.18 mL, 1.2 mmol, 2 equiv.) was added, and the reaction mixture was stirred at rt for 1 h. It was then quenched by addition of acetic acid (120 µL, ~2 equiv.), the reaction mixture was evaporated on silica, the product was isolated by flash column chromatography (12 g Interchim SiHP 30 µm cartridge, gradient 50% to 100% EtOAc/hexane) and freeze-dried from 1,4-dioxane to give 62 mg (38% over 2 steps) of **15d** as off-white solid.

mp (EtOAc/hexane): > 250 °C (dec.).
^{1}H NMR (400 MHz, DMSO-d_6): δ 9.04 (br.s, 1H), 8.30 (br.s, 1H), 7.82 (dd, J = 7.8, 0.8 Hz, 1H), 7.76 (t, J = 7.8 Hz, 1H), 7.46 (dd, J = 7.8, 0.8 Hz, 1H), 3.95 (s, 3H), 3.76 (s, 3H).
^{13}C NMR (101 MHz, DMSO-d_6): δ 162.6, 154.3, 154.1, 135.2, 128.1, 125.0, 117.4, 114.6, 96.3, 56.4, 51.0.
HRMS ($C_{11}H_{11}NO_5S$): m/z (positive mode) = 270.0435 (found $[M+H]^+$), 270.0431 (calc.).

11e

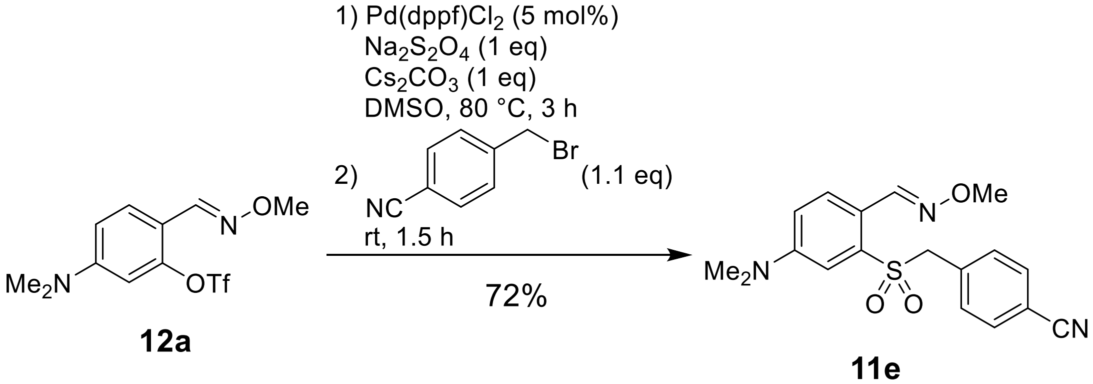

12a **11e**

A 10 mL microwave vial (Biotage) was loaded with compound **12a** (163 mg, 0.5 mmol), $Pd(dppf)Cl_2{\cdot}CH_2Cl_2$ catalyst (20 mg, 0.025 mmol, 5 mol%), sodium dithionite (87 mg, 0.5 mmol, 1 equiv.) and cesium carbonate (163 mg, 0.5 mmol, 1 equiv.). Dry DMSO solvent (2 mL) was added, the reaction mixture was degassed on a Schlenk line, placed in a preheated 80 °C silicon oil bath and stirred for 3 h. Upon cooling down to rt, 4-cyanobenzyl bromide (108 mg, 0.55 mmol, 1.1 equiv., dissolved in 0.5 mL DMSO) was injected and the mixture was stirred at rt for 1.5 h.

The reaction mixture was diluted with brine (50 mL), extracted with CH_2Cl_2 (3×20 mL) and the combined extracts were dried over Na_2SO_4. The product was isolated by flash column chromatography (12 g Interchim SiHP 30 µm cartridge, gradient 20% to 80% EtOAc/hexane) and freeze-dried from 1,4-dioxane to give 129 mg (72%) of **11e** as yellowish solid (containing ~7% of an impurity by ^{1}H NMR analysis).

^{1}H NMR (400 MHz, $CDCl_3$): δ 8.92 (s, 1H), 7.52 (ddd, *J* = 8.3, 7.8, 0.6 Hz, 1H), 7.38 (ddd, *J* = 7.8, 1.3, 0.6 Hz, 1H), 7.31 – 7.20 (m, 5H), 7.11 (dd, *J* = 8.3, 1.2 Hz, 1H), 6.50 (dt, *J* = 15.7, 1.2 Hz, 1H), 6.09 (dt, *J* = 15.7, 7.7 Hz, 1H), 4.25 (dd, *J* = 7.7, 1.2 Hz, 2H), 4.03 (s, 3H), 3.92 (s, 3H).
^{13}C NMR (101 MHz, $CDCl_3$): δ 158.4, 147.9, 139.3, 136.0, 135.3, 134.7, 128.7, 128.6, 126.8, 125.5, 122.4, 115.3, 114.0, 62.2, 60.2, 57.2.
HRMS ($C_{18}H_{19}N_3O_3S$): *m/z* (positive mode) = 358.1217 (found $[M+H]^+$), 358.1220 (calc.).

15e

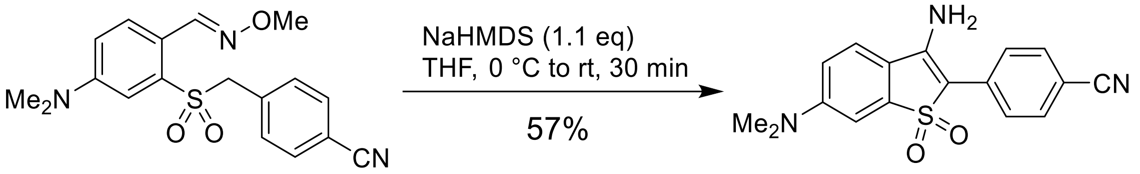

11e **15e**

A solution of NaHMDS (0.2 mL of 2 M in THF, 0.40 mmol, ~1.1 equiv.) was added dropwise to a stirred solution of **11e** (129 mg, 0.36 mmol) in dry THF (2 mL), cooled in ice-water bath. The reaction mixture was warmed up to rt and stirred for 30 min, then quenched by addition of brine (50 mL) and acetic acid (2 mL). The product was extracted with EtOAc (3×20 mL), the combined organic layers were washed with brine, dried over Na_2SO_4, and the product was isolated by flash column chromatography (12 g Interchim SiHP 30 µm cartridge, gradient 0% to 100% EtOAc/hexane + 50% CH_2Cl_2 constant additive) and freeze-dried from 1,4-dioxane to give 67 mg (57%) of **15e** as bright yellow solid.

mp (EtOAc/CH_2Cl_2): > 200 °C (dec.).
^{1}H NMR (400 MHz, DMSO-*d*$_6$): δ 7.92 (d, *J* = 8.7 Hz, 1H), 7.87 – 7.81 (m, 2H), 7.76 – 7.70 (m, 2H), 7.49 (br.s, 2H), 7.05 (d, *J* = 2.5 Hz, 1H), 6.95 (dd, *J* = 8.7, 2.5 Hz, 1H), 3.06 (s, 6H).
^{13}C NMR (101 MHz, DMSO-*d*$_6$): δ 152.3, 145.5, 140.5, 135.5, 132.6, 125.9, 122.9, 119.3, 114.52, 114.47, 106.6, 102.4, 99.9, 40.0.
HRMS ($C_{17}H_{15}N_3O_2S$): *m/z* (positive mode) = 326.0960 (found $[M+H]^+$), 326.0958 (calc.).

11f

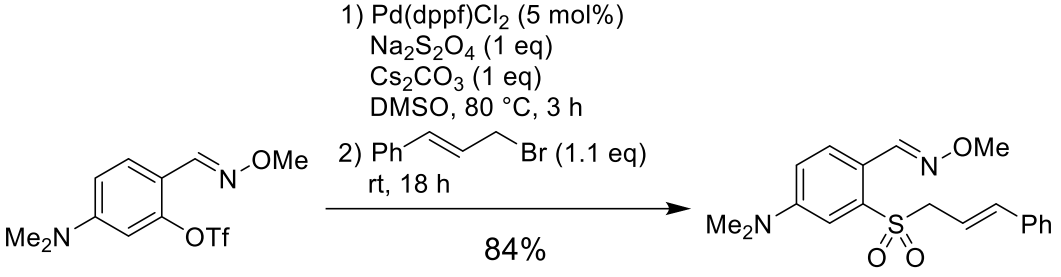

12a **11f**

A 10 mL microwave vial (Biotage) was loaded with compound **12a** (163 mg, 0.5 mmol), Pd(dppf)Cl_2·CH_2Cl_2 catalyst (20 mg, 0.025 mmol, 5 mol%), sodium dithionite (87 mg, 0.5 mmol, 1 equiv.) and cesium carbonate (163 mg, 0.5 mmol, 1 equiv.). Dry DMSO solvent (2 mL) was added, the reaction mixture was degassed on a Schlenk line, placed in a preheated 80 °C silicon oil bath and stirred for 3 h. Upon cooling down to rt, cinnamyl bromide (108 mg, 0.55 mmol, 1.1 equiv., dissolved in 0.5 mL DMSO) was injected and the mixture was stirred at rt overnight (18 h). The reaction mixture was diluted with brine (50 mL), extracted with CH_2Cl_2 (3×25 mL) and the combined extracts were dried over Na_2SO_4. The product was isolated by flash column chromatography (12 g Interchim SiHP 30 µm cartridge, gradient 10% to 80% EtOAc/hexane) and freeze-dried from 1,4-dioxane to give 151 mg (84%) of **11f** as light tan solid.

^{1}H NMR (400 MHz, $CDCl_3$): δ 8.92 (s, 1H), 7.90 (d, J = 8.8 Hz, 1H), 7.34 – 7.22 (m, 6H), 7.17 (d, J = 2.8 Hz, 1H), 6.83 (ddd, J = 8.9, 2.9, 0.7 Hz, 1H), 6.41 (dt, J = 15.8, 1.2 Hz, 1H), 6.06 (dt, J = 15.8, 7.6 Hz, 1H), 3.95 (dd, J = 7.6, 1.2 Hz, 2H), 3.89 (s, 3H), 2.98 (s, 6H).
^{13}C NMR (101 MHz, $CDCl_3$): δ 150.8, 146.2, 139.6, 137.0, 136.0, 129.2, 128.72, 128.68, 128.6, 126.9, 118.2, 116.3, 115.0, 112.9, 62.0, 61.2, 40.2.
HRMS ($C_{19}H_{22}N_2O_3S$): m/z (positive mode) = 359.1423 (found $[M+H]^+$), 359.1424 (calc.).

15f

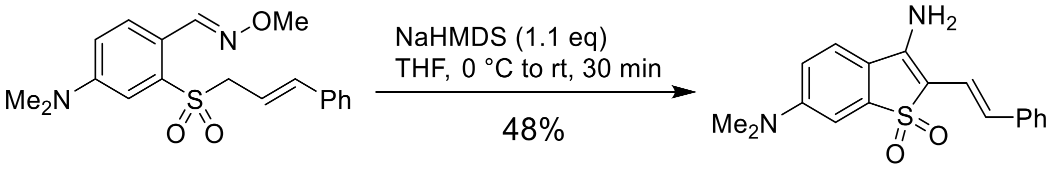

11f **15f**

A solution of NaHMDS (0.23 mL of 2 M in THF, 0.46 mmol, ~1.1 equiv.) was added dropwise to a stirred solution of **11f** (150 mg, 0.42 mmol) in dry THF (2 mL), cooled in ice-water bath. The reaction mixture was warmed up to rt and stirred for 30 min, then quenched by addition of brine

(50 mL) and acetic acid (2 mL). The product was extracted with EtOAc (2×20 mL) and CH_2Cl_2 (2×20 mL), the combined organic layers were washed with brine, dried over Na_2SO_4, and the product was isolated by flash column chromatography (12 g Interchim SiHP 30 µm cartridge, gradient 0% to 100% EtOAc/hexane + 50% CH_2Cl_2 constant additive) and freeze-dried from 1,4-dioxane to give 66 mg (48%) of **15f** as orange solid.

mp (EtOAc/CH_2Cl_2): > 250 °C (dec.).
^{1}H NMR (400 MHz, DMSO-d_6): δ 7.74 (d, J = 8.7 Hz, 1H), 7.51 – 7.45 (m, 2H), 7.36 – 7.28 (m, 4H), 7.20 – 7.13 (m, 1H), 7.00 (d, J = 2.4 Hz, 1H), 6.91 (dd, J = 8.7, 2.4 Hz, 1H), 6.55 (d, J = 16.3 Hz, 1H), 3.04 (s, 6H).
^{13}C NMR (101 MHz, DMSO-d_6): δ 152.0, 144.1, 141.5, 138.3, 128.7, 126.2, 125.2, 122.3, 120.0, 115.8, 115.1, 114.4, 102.5, 102.2, 40.1.
HRMS ($C_{18}H_{18}N_2O_2S$): m/z (positive mode) = 327.1164 (found [M+H]$^+$), 327.1162 (calc.).

11g

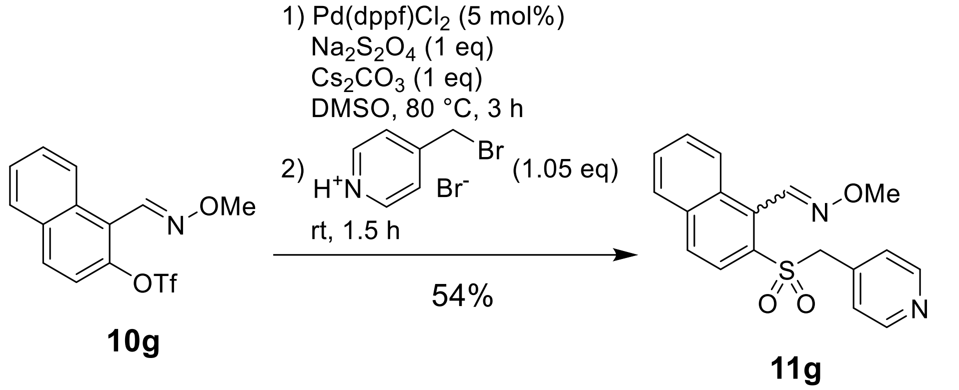

A 10 mL microwave vial (Biotage) was loaded with compound **10g** (167 mg, 0.5 mmol), Pd(dppf)Cl_2·CH_2Cl_2 catalyst (20 mg, 0.025 mmol, 5 mol%), sodium dithionite (87 mg, 0.5 mmol, 1 equiv.) and cesium carbonate (163 mg, 0.5 mmol, 1 equiv.). Dry DMSO solvent (2 mL) was added, the reaction mixture was degassed on a Schlenk line, placed in a preheated 80 °C silicon oil bath and stirred for 3 h. Upon cooling down to rt, 4-(bromomethyl)pyridine hydrobromide (133 mg, 0.53 mmol, 1.05 equiv., dissolved in 0.5 mL DMSO) was injected and the mixture was stirred at rt for 1.5 h. The reaction mixture was diluted with brine (50 mL), extracted with CH_2Cl_2 (3×20 mL) and the combined extracts were dried over Na_2SO_4. The product was isolated by flash column chromatography (12 g Interchim SiHP 30 µm cartridge, gradient 20% to 100% EtOAc/hexane + 20% CH_2Cl_2 constant additive) and freeze-dried from 1,4-dioxane to give 92 mg (54%) of **11g** as a mixture of (E/Z)-isomers of the oxime in ~70:30 ratio, which was used in the following step without further characterization.

15g

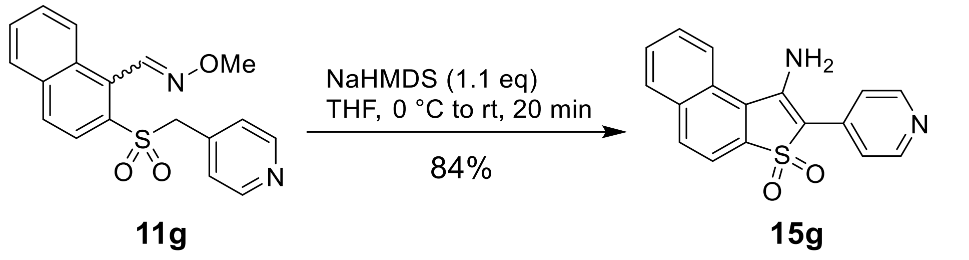

A solution of NaHMDS (0.15 mL of 2 M in THF, 0.30 mmol, ~1.1 equiv.) was added dropwise to a stirred solution of **11g** (92 mg, 0.27 mmol) in dry THF (2 mL), cooled in ice-water bath. The reaction mixture was warmed up to rt and stirred for 20 min, then quenched by addition of acetic acid (50 µL). The resulting brown-yellow suspension was diluted with CH_2Cl_2 and 2-propanol and evaporated on silica, and the product was isolated by flash column chromatography (12 g Interchim SiHP 30 µm cartridge, gradient 3% to 30% 2-propanol/CH_2Cl_2) and freeze-dried from 1,4-dioxane to give 70 mg (84%, or 45% over 2 steps) of **15g** as yellow solid.

mp (*i*-PrOH/CH_2Cl_2): > 200 °C (dec.).
^{1}H NMR (400 MHz, DMSO-d_6): δ 8.77 – 8.71 (m, 1H), 8.66 – 8.61 (m, 2H), 8.36 (d, J = 8.3 Hz, 1H), 8.21 – 8.16 (m, 1H), 7.95 (d, J = 8.3 Hz, 1H), 7.83 – 7.73 (m, 2H), 7.66 – 7.60 (m, 2H), 7.50 (br.s, 2H).
^{13}C NMR (101 MHz, DMSO-d_6): δ 150.1, 147.8, 137.6, 136.5, 136.1, 133.8, 129.9, 129.1, 128.3, 126.9, 125.2, 124.5, 120.9, 115.7, 102.5.
HRMS ($C_{17}H_{12}N_2O_2S$): m/z (positive mode) = 309.0697 (found [M+H]$^+$), 309.0692 (calc.).

11h

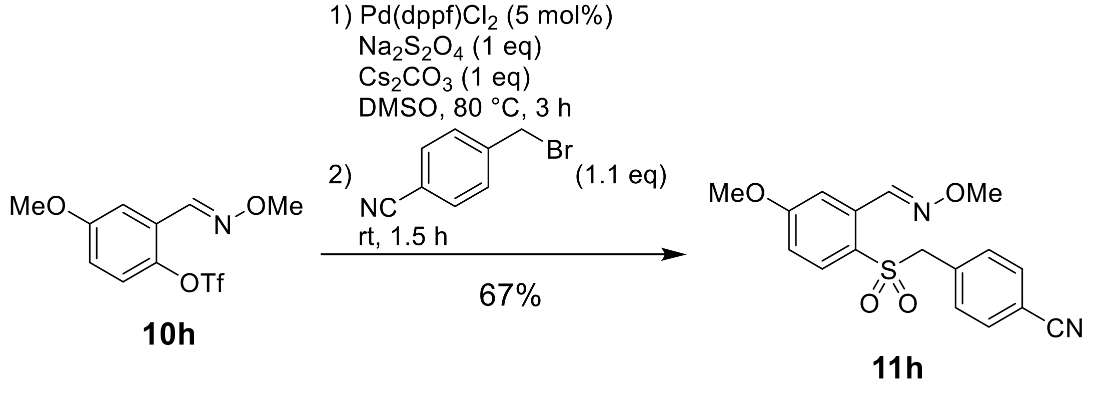

A 10 mL microwave vial (Biotage) was loaded with compound **10h** (157 mg, 0.5 mmol), Pd(dppf)Cl_2·CH_2Cl_2 catalyst (20 mg, 0.025 mmol, 5 mol%), sodium dithionite (87 mg, 0.5 mmol,

1 equiv.) and cesium carbonate (163 mg, 0.5 mmol, 1 equiv.). Dry DMSO solvent (2 mL) was added, the reaction mixture was degassed on a Schlenk line, placed in a preheated 80 °C silicon oil bath and stirred for 3 h. Upon cooling down to rt, 4-cyanobenzyl bromide (108 mg, 0.55 mmol, 1.1 equiv., dissolved in 0.5 mL DMSO) was injected and the mixture was stirred at rt for 1.5 h. The reaction mixture was diluted with brine (50 mL), extracted with CH_2Cl_2 (3×25 mL) and the combined extracts were dried over Na_2SO_4. The product was isolated by flash column chromatography (12 g Interchim SiHP 30 µm cartridge, gradient 20% to 80% EtOAc/hexane) and freeze-dried from 1,4-dioxane to give 116 mg (67%) of **11h** as white solid.

^{1}H NMR (400 MHz, $CDCl_3$): δ 8.59 (s, 1H), 7.67 (d, *J* = 8.9 Hz, 1H), 7.60 – 7.54 (m, 2H), 7.41 (d, *J* = 2.7 Hz, 1H), 7.21 – 7.16 (m, 2H), 6.91 (dd, *J* = 8.9, 2.7 Hz, 1H), 4.33 (s, 2H), 3.99 (s, 3H), 3.90 (s, 3H).
^{13}C NMR (101 MHz, $CDCl_3$): δ 163.8, 145.0, 134.6, 133.3, 133.2, 132.5, 131.7, 127.0, 118.4, 115.6, 113.0, 112.4, 63.5, 62.7, 56.0.
HRMS ($C_{17}H_{16}N_2O_4S$): *m/z* (positive mode) = 345.0908 (found $[M+H]^+$), 345.0904 (calc.).

15h

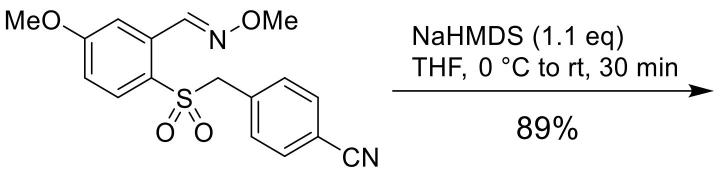

11h **15h**

A solution of NaHMDS (0.17 mL of 2 M in THF, 0.34 mmol, ~1.1 equiv.) was added dropwise to a stirred solution of **11h** (106 mg, 0.31 mmol) in dry THF (2 mL), cooled in ice-water bath. The reaction mixture was warmed up to rt and stirred for 30 min, then quenched by addition of brine (50 mL) and acetic acid (2 mL). The product precipitated as yellow solid, very poorly soluble in common low-to-moderate boiling organic solvents except pyridine. It was extracted in suspension with EtOAc (3×20 mL) and CH_2Cl_2 (3×20 mL), the undissolved product was filtered off, and the filtrate was dried over Na_2SO_4, filtered and evaporated. The combined solid product was dissolved in minimal volume of pyridine, filtered and evaporated; the solid residue was triturated with 20% CH_2Cl_2/hexane, dried, resuspended in 1,4-dioxane and freeze-dried to give 86 mg (89%) of **15h** as yellow solid.

mp (EtOAc/CH_2Cl_2, poorly soluble): > 250 °C (dec.).

^{1}H NMR (400 MHz, pyridine-d_5): δ 8.28 (br.s, 2H), 8.27 – 8.22 (m, 2H), 8.00 (d, J = 8.4 Hz, 1H), 7.75 (d, J = 2.2 Hz, 1H), 7.72 – 7.67 (m, 2H), 7.13 (dd, J = 8.4, 2.2 Hz, 1H), 3.61 (s, 3H).

^{13}C NMR (101 MHz, pyridine-d_5): δ 164.4, 144.9, 133.4, 133.1, 132.0, 128.6, 122.5, 119.8, 116.5, 110.3, 108.7, 106.4, 56.3.

HRMS ($C_{16}H_{12}N_2O_3S$): *m/z* (positive mode) = 313.0642 (found $[M+H]^+$), 313.0641 (calc.).

11i

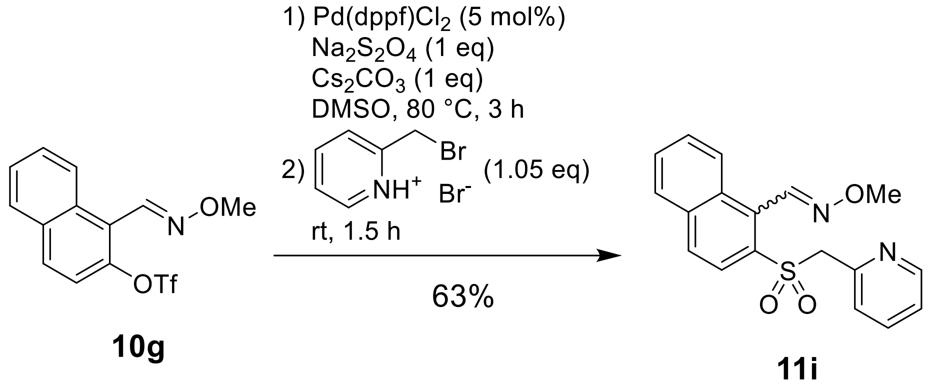

A 10 mL microwave vial (Biotage) was loaded with compound **10g** (167 mg, 0.5 mmol), $Pd(dppf)Cl_2 \cdot CH_2Cl_2$ catalyst (20 mg, 0.025 mmol, 5 mol%), sodium dithionite (87 mg, 0.5 mmol, 1 equiv.) and cesium carbonate (163 mg, 0.5 mmol, 1 equiv.). Dry DMSO solvent (2 mL) was added, the reaction mixture was degassed on a Schlenk line, placed in a preheated 80 °C silicon oil bath and stirred for 3 h. Upon cooling down to rt, 2-(bromomethyl)pyridine hydrobromide (133 mg, 0.53 mmol, 1.05 equiv.; dissolved in 0.5 mL DMSO) was injected and the mixture was stirred at rt for 1.5 h. The reaction mixture was diluted with brine (50 mL), extracted with CH_2Cl_2 (3×20 mL) and the combined extracts were dried over Na_2SO_4. The product was isolated by flash column chromatography (12 g Interchim SiHP 30 µm cartridge, gradient 20% to 100% EtOAc/hexane) and freeze-dried from 1,4-dioxane to give 107 mg (63%) of **11i** as a mixture of (E/Z)-isomers of the oxime in ~75:25 ratio, which was used in the following step without further characterization.

15i

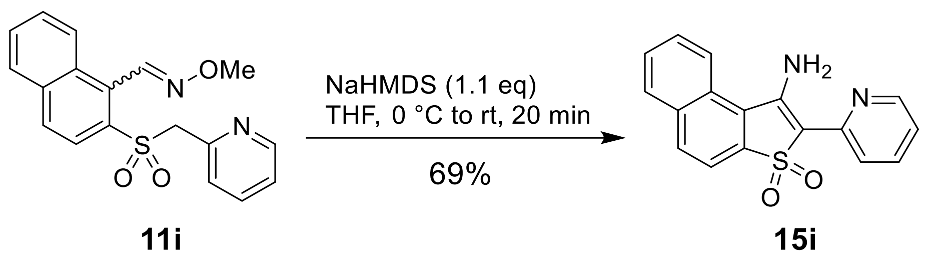

A solution of NaHMDS (0.17 mL of 2 M in THF, 0.34 mmol, ~1.1 equiv.) was added dropwise to a stirred solution of **11i** (107 mg, 0.32 mmol) in dry THF (2 mL), cooled in ice-water bath. The reaction mixture was warmed up to rt and stirred for 20 min, then quenched by addition of acetic acid (70 µL). The resulting orange-yellow suspension was diluted with CH_2Cl_2, 2-propanol and a little pyridine and evaporated on silica, and the product was isolated by flash column chromatography (12 g Interchim SiHP 30 µm cartridge, gradient 50% to 100% CH_2Cl_2/hexane followed by 0% to 50% EtOAc/CH_2Cl_2) and freeze-dried from 1,4-dioxane to give 67 mg (69%, or 43% over 2 steps) of **15i** as bright yellow solid.

mp (EtOAc/CH_2Cl_2): > 250 °C (dec.).
^{1}H NMR (400 MHz, DMSO-d_6): δ 9.20 (br.s, 2H), 8.79 – 8.73 (m, 1H), 8.66 (ddd, J = 5.0, 1.8, 1.0 Hz, 1H), 8.43 – 8.37 (m, 1H), 8.25 – 8.19 (m, 1H), 7.99 (d, J = 8.3 Hz, 1H), 7.92 (ddd, J = 8.2, 7.5, 1.9 Hz, 1H), 7.84 (ddd, J = 8.6, 6.9, 1.6 Hz, 1H), 7.79 (ddd, J = 8.0, 6.9, 1.2 Hz, 1H), 7.60 (dt, J = 8.2, 1.0 Hz, 1H), 7.24 (ddd, J = 7.5, 5.0, 1.1 Hz, 1H).
^{13}C NMR (101 MHz, DMSO-d_6): δ 150.9, 150.2, 148.4, 138.0, 137.1, 136.0, 134.2, 130.1, 129.4, 128.4, 127.0, 124.7, 123.8, 119.7, 118.6, 115.8, 102.0.
HRMS ($C_{17}H_{12}N_2O_2S$): m/z (positive mode) = 309.0695 (found $[M+H]^+$), 309.0692 (calc.).

11j

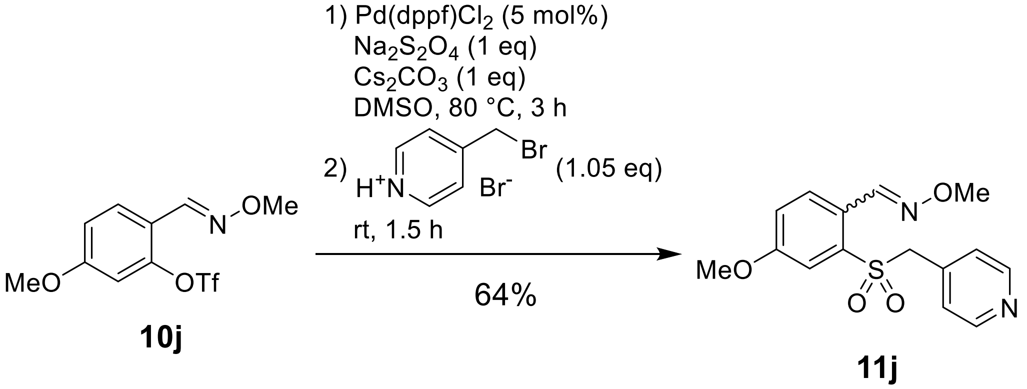

A 10 mL microwave vial (Biotage) was loaded with compound **10j** (157 mg, 0.5 mmol), Pd(dppf)$Cl_2 \cdot CH_2Cl_2$ catalyst (20 mg, 0.025 mmol, 5 mol%), sodium dithionite (87 mg, 0.5 mmol, 1 equiv.) and cesium carbonate (163 mg, 0.5 mmol, 1 equiv.). Dry DMSO solvent (2 mL) was added, the reaction mixture was degassed on a Schlenk line, placed in a preheated 80 °C silicon oil bath and stirred for 3 h. Upon cooling down to rt, 4-(bromomethyl)pyridine hydrobromide (133 mg, 0.53 mmol, 1.05 equiv., dissolved in 0.5 mL DMSO) was injected and the mixture was stirred at rt for 1.5 h. The reaction mixture was diluted with brine (50 mL), extracted with CH_2Cl_2 (3×20 mL) and the combined extracts were dried over Na_2SO_4. The product was isolated by flash column chromatography (12 g Interchim SiHP 30 µm cartridge, gradient 5% to 80% EtOAc/CH_2Cl_2) and freeze-dried from 1,4-dioxane to give 102 mg (64%) of **11j**, purity 93% (NMR).

^{1}H NMR (400 MHz, $CDCl_3$): δ 8.74 (s, 1H), 8.54 – 8.50 (m, 2H), 7.92 (d, *J* = 8.7 Hz, 1H), 7.19 (d, *J* = 2.7 Hz, 1H), 7.12 (ddd, *J* = 8.7, 2.7, 0.6 Hz, 1H), 7.05 – 7.00 (m, 2H), 4.31 (s, 2H), 3.98 (s, 3H), 3.75 (s, 3H).
^{13}C NMR (101 MHz, $CDCl_3$): δ 160.5, 150.3, 144.9, 136.5, 136.3, 129.9, 125.6, 124.6, 120.9, 114.9, 62.5, 62.4, 56.0.
HRMS ($C_{15}H_{16}N_2O_4S$): *m/z* (positive mode) = 321.0902 (found $[M+H]^+$), 321.0904 (calc.).

15j

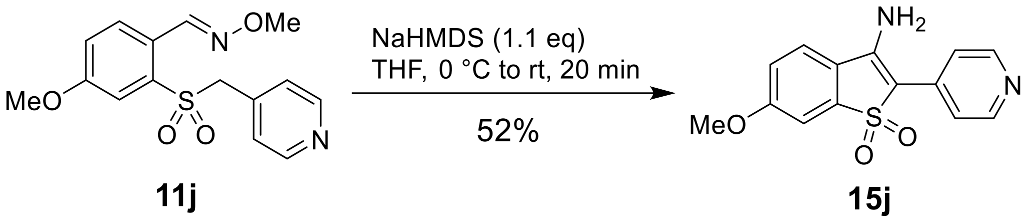

11j **15j**

A solution of NaHMDS (0.17 mL of 2 M in THF, 0.34 mmol, ~1.1 equiv.) was added dropwise to a stirred solution of **11j** (101 mg, 0.32 mmol) in dry THF (2 mL), cooled in ice-water bath. The reaction mixture was warmed up to rt and stirred for 20 min, then quenched by addition of acetic acid (70 µL). The resulting brown-yellow suspension was diluted with CH_2Cl_2 and 2-propanol and evaporated on silica, and the product was isolated by flash column chromatography (12 g Interchim SiHP 30 µm cartridge, gradient 3% to 30% 2-propanol/CH_2Cl_2) and freeze-dried from 1,4-dioxane to give 47 mg (52%) of **15j** as light tan solid.

mp (*i*-PrOH/CH_2Cl_2): > 250 °C (dec.).

^{1}H NMR (400 MHz, DMSO-d_6): δ 8.58 – 8.54 (m, 2H), 8.10 (d, J = 8.6 Hz, 1H), 7.70 (s, 2H), 7.57 – 7.53 (m, 2H), 7.48 (d, J = 2.4 Hz, 1H), 7.32 (dd, J = 8.6, 2.4 Hz, 1H), 3.91 (s, 3H).
^{13}C NMR (101 MHz, DMSO-d_6): δ 162.3, 149.9, 144.9, 140.4, 137.2, 123.5, 120.6, 119.8, 118.8, 105.7, 100.2, 56.3.
HRMS ($C_{14}H_{12}N_2O_3S$): *m/z* (positive mode) = 309.0697 (found $[M+H]^+$), 309.0692 (calc.).

11k

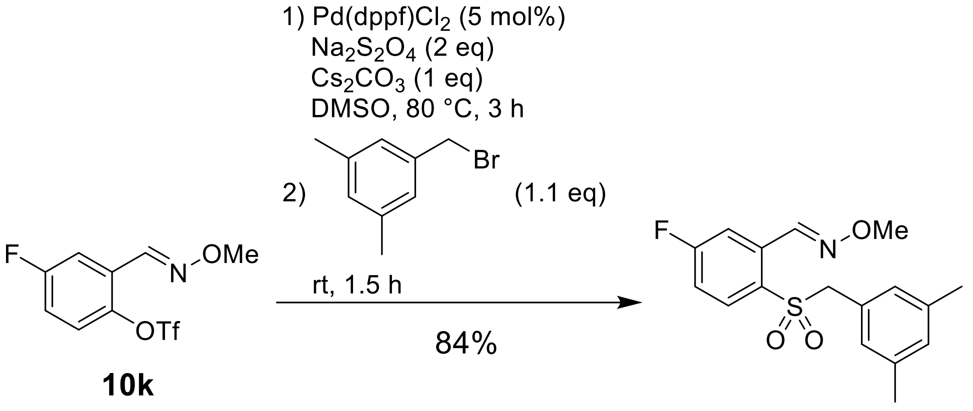

A 10 mL microwave vial (Biotage) was loaded with compound **10k** (151 mg, 0.5 mmol), Pd(dppf)Cl_2·CH_2Cl_2 catalyst (20 mg, 0.025 mmol, 5 mol%), sodium dithionite (174 mg, 1 mmol, 2 equiv.) and cesium carbonate (163 mg, 0.5 mmol, 1 equiv.). Dry DMSO solvent (2 mL) was added, the reaction mixture was degassed on a Schlenk line, placed in a preheated 80 °C silicon oil bath and stirred for 3 h. Upon cooling down to rt, 3,5-dimethylbenzyl bromide (109 mg, 0.55 mmol, 1.1 equiv., dissolved in 0.5 mL DMSO) was injected and the mixture was stirred at rt for 1.5 h. The reaction mixture was diluted with brine (50 mL), extracted with CH_2Cl_2 (3×20 mL) and the combined extracts were dried over Na_2SO_4. The product was isolated by flash column chromatography (12 g Interchim SiHP 30 µm cartridge, gradient 3% to 60% EtOAc/hexane) and freeze-dried from 1,4-dioxane to give 140 mg (84%) of **11k** as white solid.

^{1}H NMR (400 MHz, $CDCl_3$): δ 8.55 (d, J = 1.7 Hz, 1H), 7.81 (dd, J = 8.8, 5.5 Hz, 1H), 7.67 (dd, J = 9.7, 2.7 Hz, 1H), 7.10 (ddd, J = 8.9, 7.4, 2.7 Hz, 1H), 6.93 (dq, J = 1.6, 0.8 Hz, 1H), 6.62 (dq, J = 1.6, 0.8 Hz, 2H), 4.22 (s, 2H), 3.96 (s, 3H), 2.21 (d, J = 0.8 Hz, 6H).
^{19}F NMR (376 MHz, $CDCl_3$): δ -103.4.
^{13}C NMR (101 MHz, $CDCl_3$): δ 165.4 (d, J = 256.1 Hz), 144.4 (d, J = 2.2 Hz), 138.6, 136.0 (d, J = 9.4 Hz), 134.0 (d, J = 9.6 Hz), 131.7 (d, J = 3.2 Hz), 130.8, 128.7, 127.1, 116.7 (d, J = 22.4 Hz), 114.2 (d, J = 24.5 Hz), 63.9, 62.6, 21.2.

HRMS ($C_{17}H_{18}FNO_3S$): *m/z* (positive mode) = 336.1065 (found $[M+H]^+$), 336.1064 (calc.).

15k

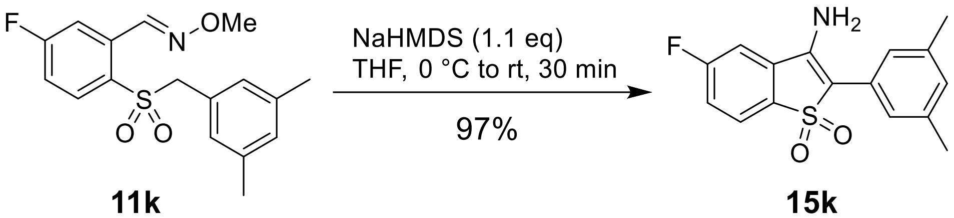

11k **15k**

A solution of NaHMDS (0.23 mL of 2 M in THF, 0.46 mmol, ~1.1 equiv.) was added dropwise to a stirred solution of **11k** (139 mg, 0.42 mmol) in dry THF (3 mL), cooled in ice-water bath. The reaction mixture was warmed up to rt and stirred for 20 min, then quenched by addition of brine. The product was extracted with EtOAc (3×25 mL) from the mixture of brine (80 mL) and acetic acid (2 mL), the combined extracts were dried over Na_2SO_4 and the product was isolated by flash column chromatography (12 g Interchim SiHP 30 µm cartridge, gradient 0% to 60% EtOAc/hexane with 20% CH_2Cl_2 constant additive) and freeze-dried from 1,4-dioxane to give 122 mg (97%) of **15k** as light-yellow solid.

mp (CH_2Cl_2/hexane): 227-228 °C.
^{1}H NMR (400 MHz, DMSO-d_6): δ 8.00 (dd, *J* = 9.4, 2.3 Hz, 1H), 7.90 (dd, *J* = 8.3, 4.9 Hz, 1H), 7.47 (ddd, *J* = 9.0, 8.3, 2.3 Hz, 1H), 7.21 (dq, *J* = 1.5, 0.7 Hz, 2H), 7.08 (br.s, 2H), 7.00 – 6.95 (m, 1H), 2.32 (d, *J* = 0.7 Hz, 6H).
^{19}F NMR (376 MHz, DMSO-d_6): δ -105.73.
^{13}C NMR (101 MHz, DMSO-d_6): δ 164.9 (d, *J* = 249.6 Hz), 140.4 (d, *J* = 1.8 Hz), 138.0, 134.2 (d, *J* = 3.0 Hz), 132.8 (d, *J* = 9.9 Hz), 128.9, 128.2, 125.3, 122.3 (d, *J* = 9.9 Hz), 117.2 (d, *J* = 23.5 Hz), 109.4 (d, *J* = 26.3 Hz), 105.3, 21.1.
HRMS ($C_{16}H_{14}FNO_2S$): *m/z* (positive mode) = 304.0802 (found $[M+H]^+$), 304.0802 (calc.).

11l

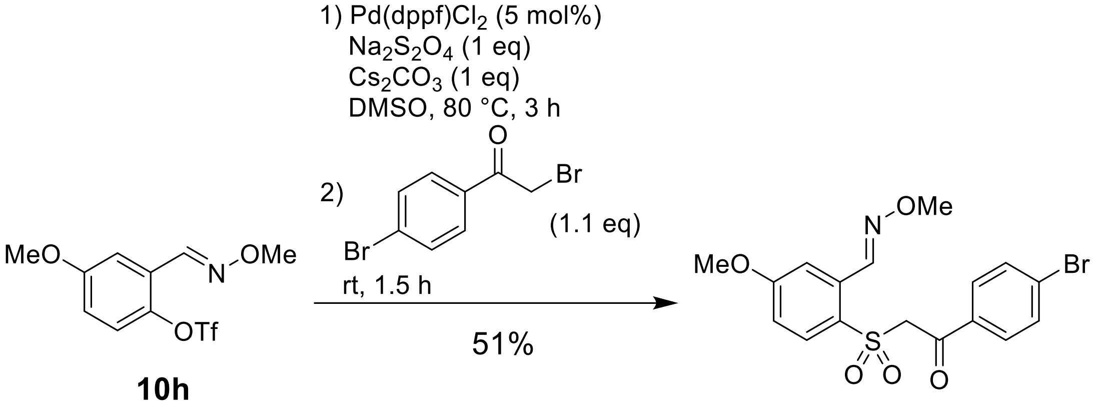

10h **11l**

A 10 mL microwave vial (Biotage) was loaded with compound **10h** (157 mg, 0.5 mmol), $Pd(dppf)Cl_2 \cdot CH_2Cl_2$ catalyst (20 mg, 0.025 mmol, 5 mol%), sodium dithionite (87 mg, 0.5 mmol, 1 equiv.) and cesium carbonate (163 mg, 0.5 mmol, 1 equiv.). Dry DMSO solvent (2 mL) was added, the reaction mixture was degassed on a Schlenk line, placed in a preheated 80 °C silicon oil bath and stirred for 3 h. Upon cooling down to rt, 2,4'-dibromoacetophenone (153 mg, 0.55 mmol, 1.1 equiv., dissolved in 0.5 mL DMSO) was injected and the mixture was stirred at rt for 1.5 h. The resulting orange reaction mixture was diluted with brine (50 mL), extracted with CH_2Cl_2 (3×20 mL) and the combined extracts were dried over Na_2SO_4. The product was isolated by flash column chromatography (12 g Interchim SiHP 30 µm cartridge, gradient 0% to 50% EtOAc/hexane with 20% CH_2Cl_2 constant additive) and freeze-dried from 1,4-dioxane to give 108 mg (51%) of **11l** as off-white solid with purity ~92% (^{1}H NMR), used in the following step without additional purification.

^{1}H NMR (400 MHz, $CDCl_3$): δ 8.87 (s, 1H), 7.82 (d, J = 8.9 Hz, 1H), 7.82 – 7.73 (m, 2H), 7.64 – 7.59 (m, 2H), 7.47 (d, J = 2.7 Hz, 1H), 6.95 (dd, J = 8.9, 2.7 Hz, 1H), 4.67 (s, 2H), 4.02 (s, 3H), 3.91 (s, 3H).
^{13}C NMR (101 MHz, $CDCl_3$): δ 187.1, 164.0, 145.3, 134.6, 134.2, 133.2, 132.4, 130.9, 130.1, 128.3, 115.5, 112.9, 64.4, 62.7, 56.0.
HRMS ($C_{17}H_{16}BrNO_5S$): m/z (positive mode) = 426.0007 (found $[M+H]^+$), 426.0005 (calc.).

15l

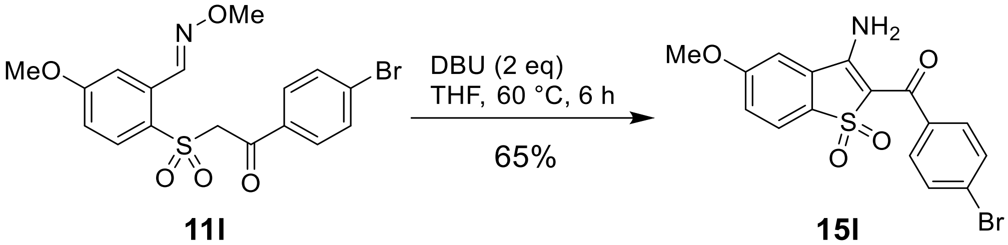

DBU (76 µL, 0.51 mmol, 2 equiv.) was added at rt to the stirred light-yellow solution of **11l** (108 mg, 0.25 mmol) in dry THF (2 mL), which immediately turned orange. The reaction mixture was placed in a preheated (60 °C) silicone oil bath and stirred at 60 °C for 6 h. On cooling down to rt, the product was extracted with EtOAc (3×25 mL) from the mixture of brine (50 mL) and acetic acid (1 mL), the combined extracts were dried over Na_2SO_4 and the product was isolated by flash column chromatography (12 g Interchim SiHP 30 µm cartridge, gradient 0% to 50% EtOAc/CH_2Cl_2) and freeze-dried from 1,4-dioxane to give 65 mg (65%) of **15l** as off-white solid.

mp (EtOAc/CH_2Cl_2): > 250 °C (dec.).
^{1}H NMR (400 MHz, DMSO-d_6): δ 10.00 (br.s, 1H), 9.76 (br.s, 1H), 8.04 (d, J = 2.3 Hz, 1H), 7.83 – 7.76 (m, 3H), 7.74 – 7.69 (m, 2H), 7.38 (dd, J = 8.5, 2.3 Hz, 1H), 3.91 (s, 3H).
^{13}C NMR (101 MHz, DMSO-d_6): δ 185.9, 163.3, 157.5, 139.0, 131.2, 130.6, 129.2, 128.3, 124.9, 122.4, 120.7, 108.5, 107.6, 56.3.
HRMS ($C_{16}H_{12}BrNO_4S$): m/z (positive mode) = 393.9744 (found $[M+H]^+$), 393.9743 (calc.).

11m

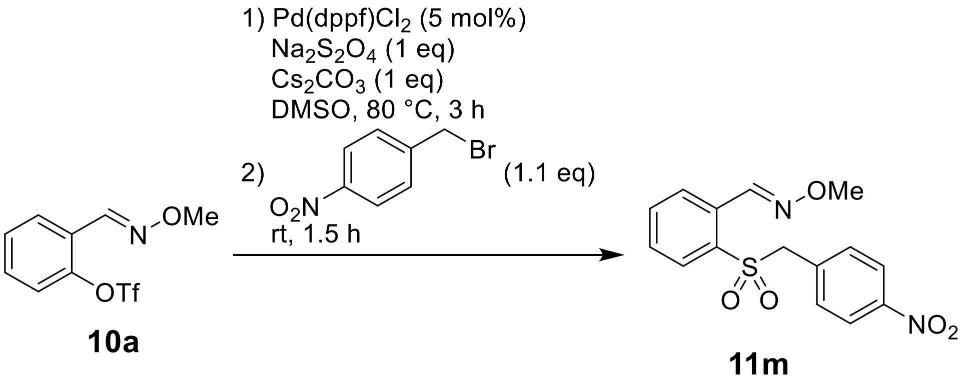

A 10 mL microwave vial (Biotage) was loaded with compound **10a** (142 mg, 0.5 mmol), $Pd(dppf)Cl_2 \cdot CH_2Cl_2$ catalyst (20 mg, 0.025 mmol, 5 mol%), sodium dithionite (87 mg, 0.5 mmol, 1 equiv.) and cesium carbonate (163 mg, 0.5 mmol, 1 equiv.). Dry DMSO solvent (2 mL) was added, the reaction mixture was degassed on a Schlenk line, placed in a preheated 80 °C silicon

oil bath and stirred for 3 h. Upon cooling down to rt, 4-nitrobenzyl bromide (119 mg, 0.55 mmol, 1.05 equiv.; dissolved in 0.5 mL DMSO) was injected and the mixture was stirred at rt for 1.5 h. The reaction mixture was diluted with brine (50 mL), extracted with CH_2Cl_2 (3×20 mL) and the combined extracts were dried over Na_2SO_4. The product was isolated by flash column chromatography (12 g Interchim SiHP 30 µm cartridge, gradient 5% to 60% EtOAc/hexane with 20% CH_2Cl_2 constant additive) to give 129 mg (63%) of impure **11m**, which was used in the following step without further characterization.

15m

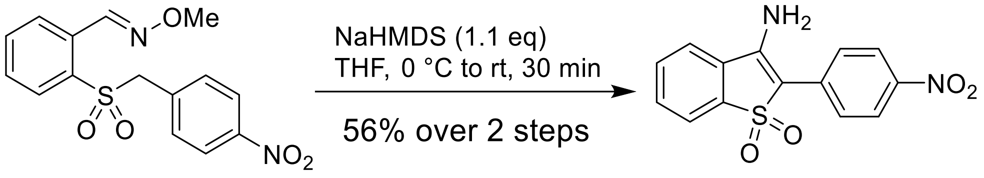

11m **15m**

A solution of NaHMDS (0.21 mL of 2 M in THF, 0.42 mmol, ~1.1 equiv.) was added dropwise to a stirred solution of **11m** (129 mg, from the previous step) in dry THF (2.5 mL), cooled in ice-water bath. Deep purple reaction mixture was warmed up to rt and stirred for 20 min, then diluted with the mixture of brine (50 mL) and acetic acid (1 mL). The product was extracted with CH_2Cl_2 (3×25 mL), pyridine (1 mL) was added to the combined extracts, which were then dried over Na_2SO_4. The product was isolated by flash column chromatography (12 g Interchim SiHP 30 µm cartridge, gradient 0% to 50% CH_2Cl_2/EtOAc/) and freeze-dried from 1,4-dioxane to give 85 mg (56% over 2 steps) of **15m** as bright yellow solid.

mp (EtOAc/CH_2Cl_2): > 250 °C (dec.).
^{1}H NMR (400 MHz, DMSO-d_6): δ 8.35 – 8.29 (m, 2H), 8.20 (d, J = 7.7 Hz, 1H), 7.93 – 7.90 (m, 1H), 7.90 – 7.85 (m, 2H), 7.84 – 7.78 (m, 3H), 7.74 (td, J = 7.5, 1.0 Hz, 1H).
^{13}C NMR (101 MHz, DMSO-d_6): δ 144.7, 144.5, 138.1, 136.7, 133.4, 131.8, 128.7, 127.0, 124.2, 121.9, 120.1, 102.0.
HRMS ($C_{14}H_{10}N_2O_4S$): m/z (positive mode) = 303.0437 (found $[M+H]^+$), 303.0434 (calc.).

15n

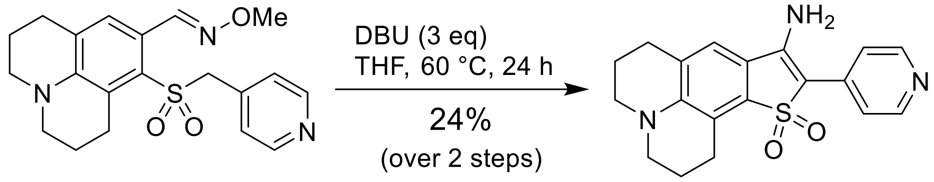

13b **15n**

1,8-Diazabicyclo[5.4.0]undec-7-ene (0.22 mL, 1.5 mmol, ~3 equiv.) was added to the solution of **13b** (88 mg crude; prepared from 0.5 mmol **12b** as described above)) in dry THF (4 mL). The resulting solution was placed in a 60 °C oil bath and left stirring overnight (24 h). It was then diluted with CH_2Cl_2 and evaporated on neutral alumina, the product was isolated by flash column chromatography (12 g Interchim SiHP 30 µm cartridge, gradient 5% to 50% 2-propanol/CH_2Cl_2) and freeze-dried from 1,4-dioxane to give 42 mg (24% over two steps) of **15n** as yellow solid.

mp (*i*-PrOH/CH_2Cl_2): > 200 °C (dec.).
^{1}H NMR (400 MHz, DMSO-*d*$_6$): δ 8.52 – 8.44 (m, 2H), 7.53 (s, 1H), 7.51 – 7.47 (m, 2H), 7.39 (br.s, 2H), 3.30 – 3.22 (m, 4H), 3.00 (t, *J* = 6.4 Hz, 2H), 2.74 (t, *J* = 6.3 Hz, 2H), 1.97 – 1.82 (m, 4H).
^{13}C NMR (101 MHz, DMSO-*d*$_6$): δ 149.6, 146.4, 145.1, 137.9, 134.5, 123.4, 120.1, 119.0, 115.5, 113.6, 98.7, 49.4, 48.7, 27.8, 21.7, 20.5, 19.9.
HRMS ($C_{19}H_{19}N_3O_2S$): *m/z* (positive mode) = 354.1275 (found $[M+H]^+$), 354.1271 (calc.).

15o

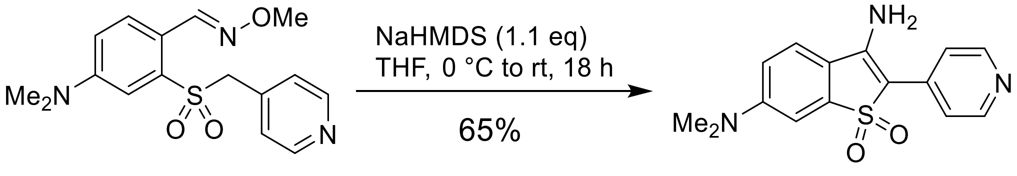

13a **15o**

A solution of NaHMDS (0.3 mL of 2 M in THF, 0.6 mmol, ~1.1 equiv.) was added dropwise to a stirred solution of **13a** (180 mg, 0.54 mmol) in dry THF (4 mL), cooled in ice-water bath. The resulting brown suspension was warmed up to rt and left stirring overnight (18 h); it was then diluted with CH_2Cl_2 and 2-propanol and evaporated on silica, and the product was isolated by flash column chromatography (12 g Interchim SiHP 30 µm cartridge, gradient 0% to 100% A/B, A = CH_2Cl_2 – ethanol - 25% aq. NH_3 80:20:2, B = CH_2Cl_2) and freeze-dried from 1,4-dioxane to give 105 mg (65%) of **15o** as yellow solid.

mp ($EtOH/CH_2Cl_2$): > 250 °C (dec.).

^{1}H NMR (400 MHz, DMSO-d_6): δ 8.55 – 8.48 (m, 2H), 7.95 (d, J = 8.8 Hz, 1H), 7.56 (br.s, 2H), 7.54 – 7.48 (m, 2H), 7.06 (d, J = 2.4 Hz, 1H), 6.95 (dd, J = 8.8, 2.5 Hz, 1H), 3.06 (s, 6H).

^{13}C NMR (101 MHz, DMSO-d_6): δ 152.4, 149.7, 146.2, 140.7, 137.8, 122.9, 119.2, 114.5, 114.4, 102.3, 98.7, 66.4, 40.0.

HRMS ($C_{15}H_{15}N_3O_2S$): *m/z* (positive mode) = 302.0955 (found $[M+H]^+$), 302.0958 (calc.).

Preparation of large Stokes shift fluorescent dye 20

17

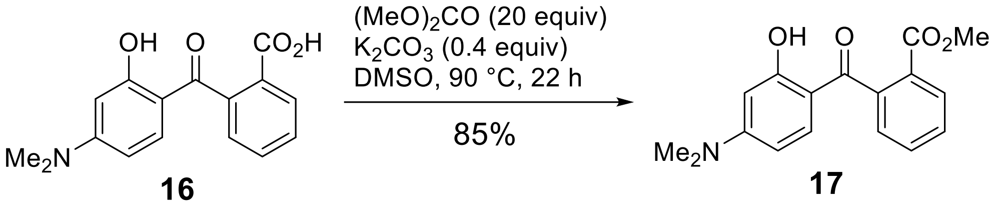

Following the procedure from [S21], a mixture of compound **16** (2.89 g, 10 mmol; known compound[S22]) and K_2CO_3 (552 mg, 4 mmol, 0.4 equiv.) in dimethyl carbonate (17 mL, 200 mmol, 20 equiv.) and DMSO (50 mL) was stirred at 90 °C for 22 h. On cooling, the reaction mixture was poured into 400 mL water, acidified with 1 N HCl to pH 3-4 and extracted with EtOAc (4×50 mL, with some CH_2Cl_2 added to enhance the solubility). The combined extracts were washed with brine, dried over Na_2SO_4, and the product was isolated by flash column chromatography (80 g RediSep Rf silica 40-63 µm cartridge, gradient 0% to 50% EtOAc/hexane + 20% CH_2Cl_2 constant additive). Fractions containing the product were evaporated, the residue was triturated with hexane and cooled to -20 °C; the crystals were filtered off, washed with hexane and dried *in vacuo*. Yield 2.55 g (85%) of **17** as yellowish solid.

mp (EtOAc/hexane): 141-142 °C.

^{1}H NMR (400 MHz, DMSO-d_6): δ 12.49 (s, 1H), 8.00 – 7.95 (m, 1H), 7.73 (td, J = 7.5, 1.3 Hz, 1H), 7.65 (td, J = 7.5, 1.3 Hz, 1H), 7.44 (ddd, J = 7.5, 1.4, 0.5 Hz, 1H), 6.82 (d, J = 9.1 Hz, 1H), 6.21 (dd, J = 9.1, 2.5 Hz, 1H), 6.12 (d, J = 2.5 Hz, 1H), 3.66 (s, 3H), 3.01 (s, 6H).

^{13}C NMR (101 MHz, DMSO-d_6): δ 198.0, 165.9, 164.4, 155.8, 140.0, 133.8, 132.5, 129.8, 129.7, 128.4, 127.9, 109.4, 104.4, 97.0, 52.3, 39.6.

HRMS ($C_{17}H_{17}NO_4$): *m/z* (positive mode) = 300.1229 (found $[M+H]^+$), 300.1230 (calc.).

18

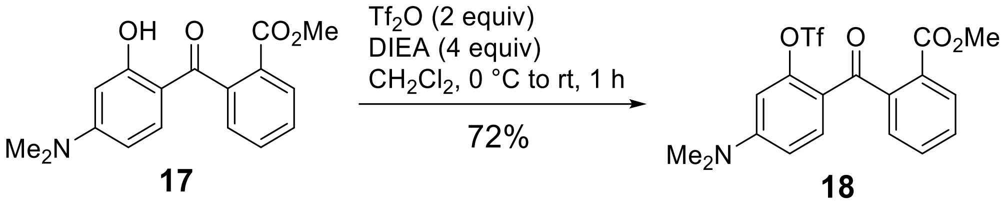

17 **18**

Trifluoromethanesulfonic anhydride (3.6 mL of 1 M solution in CH_2Cl_2, ~3.6 mmol, 2 equiv.) was added dropwise to a solution of compound **17** (532 mg, 1.78 mmol) and *N*,*N*-diisopropylethylamine (DIEA; 1.24 mL, 7.12 mmol, 4 equiv.) in dry CH_2Cl_2, cooled in ice-water bath. The reaction mixture was then allowed to warm up to rt and stirred for 1 h. The mixture was then diluted with water (100 mL), extracted with CH_2Cl_2 (3×30 mL), the combined extracts were washed with brine and dried over Na_2SO_4. The filtrate was evaporated on silica, the product was isolated by flash column chromatography (40 g RediSep Rf silica 40-63 µm cartridge, gradient 10% to 80% EtOAc/hexane) and freeze-dried from 1,4-dioxane to give 549 mg (72%) of **18** as light-brown solid.

mp (EtOAc/hexane): 120-121 °C.

^{1}H NMR (400 MHz, $CDCl_3$): δ 8.00 (d, *J* = 6.5 Hz, 1H), 7.58 (t, *J* = 7.7 Hz, 1H), 7.52 (t, *J* = 8.3 Hz, 1H), 7.38 (d, *J* = 6.2 Hz, 1H), 7.25 (d, *J* = 8.9 Hz, 1H), 6.56 – 6.41 (m, 2H), 3.69 (s, 3H), 3.06 (s, 6H).

^{13}C NMR (101 MHz, $CDCl_3$): δ 192.6, 166.8, 154.0, 150.2, 142.2, 135.3, 132.3, 130.3, 129.6, 129.4, 128.0, 118.9 (q, *J* = 320.7 Hz), 117.4, 109.6, 105.2, 105.1, 52.5, 40.1.

HRMS ($C_{18}H_{16}F_3NO_6S$): *m/z* (positive mode) = 432.0719 (found $[M+H]^+$), 432.0723 (calc.).

19

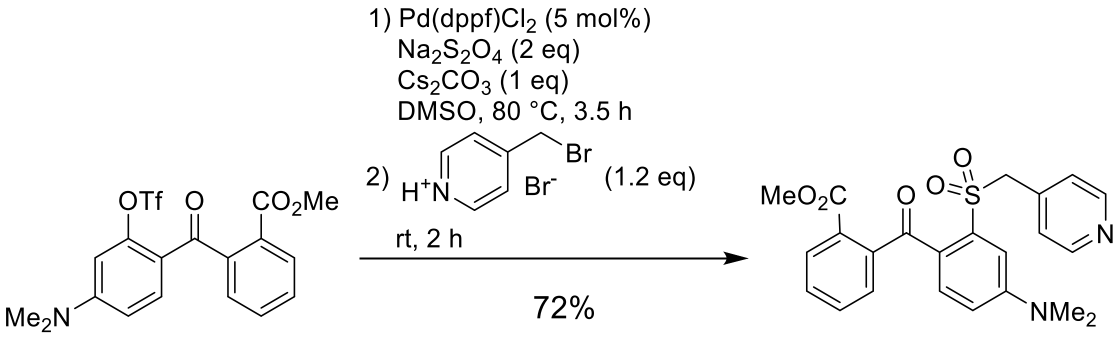

18 **19**

A 10 mL microwave vial (Biotage) was loaded with compound **18** (172 mg, 0.4 mmol), $Pd(dppf)Cl_2 \cdot CH_2Cl_2$ catalyst (16.3 mg, 0.02 mmol, 5 mol%), sodium dithionite (139 mg, 0.8

mmol, 2 equiv.) and cesium carbonate (130 mg, 1 mmol, 1 equiv.). Dry DMSO solvent (2 mL) was added, the reaction mixture was degassed on a Schlenk line, placed in a preheated 80 °C silicon oil bath and stirred for 3.5 h. Upon cooling down to rt, 4-(bromomethyl)pyridine hydrobromide (121 mg, 0.48 mmol, 1.2 equiv.; dissolved in 0.4 mL DMSO) was injected and the mixture was stirred at rt for 2 h. The reaction mixture was diluted with brine (50 mL), extracted with CH_2Cl_2 (3×25 mL) and the combined extracts were dried over Na_2SO_4. The product was isolated by flash column chromatography (12 g Interchim SiHP 30 µm cartridge, gradient 0% to 100% A/B, A = 20% *i*-PrOH/CH_2Cl_2, B = CH_2Cl_2) to give 127 mg (72%) of **19** as light red solid.

^{1}H NMR (400 MHz, $CDCl_3$): δ 8.57 – 8.50 (m, 2H), 7.97 – 7.91 (m, 1H), 7.61 – 7.49 (m, 3H), 7.40 – 7.35 (m, 2H), 7.29 (d, *J* = 8.7 Hz, 1H), 7.14 (d, *J* = 2.7 Hz, 1H), 6.58 (dd, *J* = 8.7, 2.7 Hz, 1H), 5.11 (s, 2H), 3.78 (s, 3H), 2.96 (s, 6H).
^{13}C NMR (101 MHz, $CDCl_3$): δ 195.3, 167.6, 151.8, 150.0, 141.7, 139.6, 138.0, 135.0, 131.9, 130.8, 130.4, 130.0, 129.2, 126.3, 124.4, 115.6, 113.0, 62.2, 52.7, 40.1.
HRMS ($C_{23}H_{22}N_2O_5S$): *m/z* (positive mode) = 439.1320 (found $[M+H]^+$), 439.1322 (calc.).

20

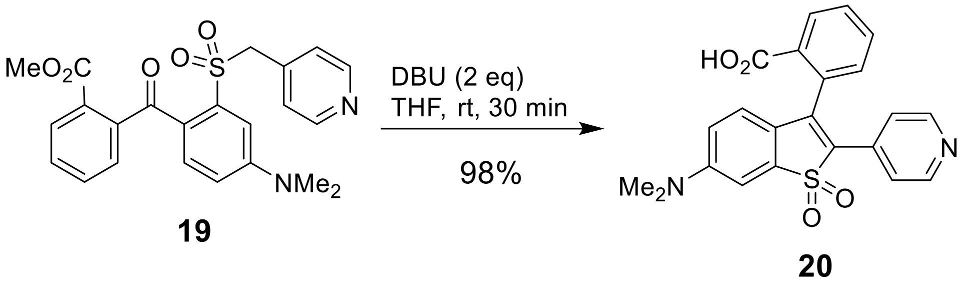

19 **20**

1,8-Diazabicyclo[5.4.0]undec-7-ene (DBU; 180 µL, 1.21 mmol, 2 equiv) was added to a stirred solution of **19** (265 mg, 0.61 mmol) in dry THF (6 mL), and the resulting brown-yellow solution was stirred at rt for 30 min. Acetic acid (0.45 mL) was then added, the solvents were evaporated and the product was isolated by flash column chromatography (25 g Interchim SiHP 30 µm cartridge, gradient 0% to 60% A/B, A = 50% *i*-PrOH/CH_2Cl_2, B = CH_2Cl_2) to give 240 mg (98%) of **20** as orange solid.

^{1}H NMR (400 MHz, DMSO-d_6): δ 13.0 (br.s, 1H), 8.52 – 8.46 (m, 2H), 8.15 – 8.08 (m, 1H), 7.70 – 7.62 (m, 2H), 7.31 – 7.24 (m, 2H), 7.22 – 7.17 (m, 2H), 6.82 (dd, *J* = 8.7, 2.5 Hz, 1H), 6.68 (d, *J* = 8.7 Hz, 1H), 3.05 (s, 6H).
^{13}C NMR (101 MHz, DMSO-d_6): δ 152.0, 150.1, 137.7, 135.7, 133.0, 132.0, 130.9, 129.9, 129.3, 127.8, 124.8, 122.3, 121.4, 119.1, 119.0, 115.3, 104.3, 40.1.

HRMS ($C_{22}H_{18}N_2O_4S$): *m/z* (positive mode) = 407.1058 (found $[M+H]^+$), 407.1060 (calc.).

Preparation of fluorescent ligands 20-Halo, 20-HTL2, 20-SNAP, 20-CLIP

N-Me-HaloTag(O2) ligand S7

S6

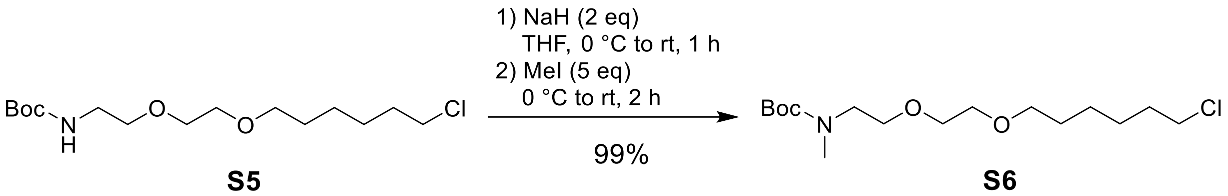

A solution of **S5** (532 mg, 1.64 mmol; known compound[S23]) in dry THF (3 mL) was added dropwise to a stirred suspension of sodium hydride (131 mg of 60 wt% in mineral oil; 3.28 mmol, 2 equiv) in dry THF (10 mL), cooled in ice-water bath (under argon). The mixture was allowed to warm up to rt, stirred for 1 h and cooled again in ice-water bath. Iodomethane (0.5 mL, 8 mmol, ~5 equiv) was then added dropwise, the mixture was warmed up to rt and stirred for 2 h. It was then cooled in ice-water bath and quenched by dropwise addition of sat. aq. NH_4Cl + sat. aq. $Na_2S_2O_3$ (1:1, 10 mL), the product was extracted with diethyl ether (3×20 mL), the combined extracts were washed with brine and dried over $MgSO_4$. The product was isolated by flash column chromatography (25 g Interchim SiHP 30 µm cartridge, gradient 10% to 60% EtOAc/hexane) to give 552 mg (99%) of **S6** as yellowish oil.

^1H NMR (400 MHz, $CDCl_3$): δ 3.63 – 3.50 (m, 8H), 3.46 (t, *J* = 6.6 Hz, 2H), 3.39 (br.s, 2H), 2.91 (s, 3H), 1.78 (dq, *J* = 7.9, 6.7 Hz, 2H), 1.60 (dq, *J* = 7.9, 6.7 Hz, 2H), 1.51 – 1.33 (m, 13H).
^{13}C NMR (101 MHz, $CDCl_3$): δ 155.8, 79.3, 71.3, 70.5, 70.3, 70.1, 69.6, 48.6, 48.2, 45.0, 35.6, 35.3, 32.6, 29.5, 28.5, 26.7, 25.4.
HRMS ($C_{16}H_{32}ClNO_4$): *m/z* (positive mode) = 338.2090 (found $[M+H]^+$), 338.2093 (calc.).

S7

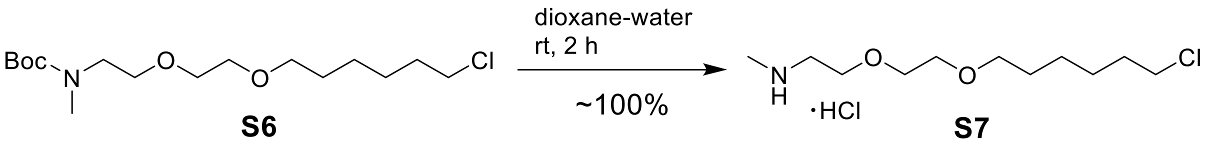

Conc. aq. HCl (5 mL) was added to the stirred solution of **S6** (537 mg, 1.59 mmol) in 1,4-dioxane (5 mL), and the resulting solution was stirred at rt for 2 h. It was then diluted with 1,4-dioxane-water mixture (1:1, 10 mL), evaporated to dryness and chased once with aqueous 1,4-dioxane (1:1). The residue was dried in vacuo to obtain **S7** as yellowish hygroscopic solid (452 mg, ~quantitative yield, remainder 1,4-dioxane).

^{1}H NMR (400 MHz, $CDCl_3$): δ 9.52 (br.s, 2H), 3.95 – 3.87 (m, 2H), 3.72 – 3.66 (m, 2H), 3.65 – 3.59 (m, 2H), 3.54 (t, *J* = 6.7 Hz, 2H), 3.47 (t, *J* = 6.7 Hz, 2H), 3.19 (p, *J* = 5.4 Hz, 2H), 2.84 – 2.73 (m, 3H), 1.78 (dq, *J* = 8.0, 6.7 Hz, 2H), 1.59 (dt, *J* = 14.2, 6.9 Hz, 2H), 1.51 – 1.41 (m, 2H), 1.40 – 1.32 (m, 2H).
^{13}C NMR (101 MHz, $CDCl_3$): δ 71.4, 70.7, 70.1, 66.2, 48.5, 45.2, 33.4, 32.6, 29.5, 26.8, 25.5.
HRMS ($C_{11}H_{24}ClNO_2$): *m/z* (positive mode) = 238.1568 (found $[M+H]^+$), 238.1568 (calc.).

20-Halo

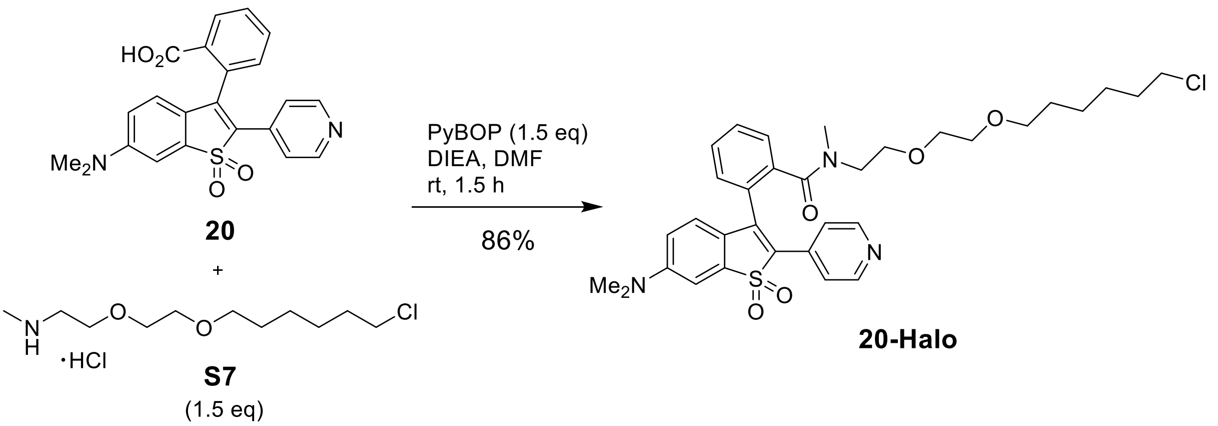

Benzotriazole-1-yl-oxy-tris-pyrrolidino-phosphonium hexafluorophosphate (PyBOP; 29 mg, 55 µmol, 1.5 equiv) was added to a stirred solution of **20** (15 mg, 37 µmol), **S7** (15 mg, 55 µmol, 1.5 equiv) and *N*,*N*-diisopropylethylamine (DIEA; 80 µL) in anhydrous DMF (0.3 mL), and the resulting solution was stirred at rt for 1.5 h. The solvents were removed *in vacuo*, and the product was isolated by preparative HPLC (ThermoFisher Hypersil Gold C18 250×21.2 mm 5 µm, solvent flow rate 18 mL/min, gradient 25% to 80% A:B, A – acetonitrile + 0.1% (v/v) HCO_2H, B – water + 0.1% (v/v) HCO_2H) to give 20 mg (86%) of **20-Halo** as viscous orange oil.

^{1}H NMR (400 MHz, $CDCl_3$): δ 8.51 – 8.45 (m, 2H), 7.66 – 7.33 (m, 6H), 7.11 (d, *J* = 2.4 Hz, 1H), 6.93 (d, *J* = 8.7 Hz, 1H), 6.67 (dd, *J* = 8.7, 2.4 Hz, 1H), 3.58 – 3.39 (m, 10H), 3.09 (s, 6H), 2.79 (s, 0.8H, N–C*H*$_3$, minor rotamer), 2.51 (s, 2.2H, N–C*H*$_3$, major rotamer), 1.82 – 1.71 (m, 2H),

1.58 (p, J = 6.8 Hz, 2H), 1.50 – 1.29 (m, 4H). Mixture of 2 rotameric forms in ~85:15 ratio (only signals of the major rotamer are listed).
^{13}C NMR (101 MHz, $CDCl_3$): δ 169.1, 168.4, 152.3, 149.4, 142.5, 138.4, 136.5, 130.6, 130.2, 129.8, 129.6, 129.5, 129.33, 129.28, 128.1, 126.1, 122.4, 122.2, 119.8, 114.9, 104.7, 71.5, 71.3, 70.8, 70.4, 70.3, 70.1, 69.2, 68.2, 47.4, 45.2, 40.6, 38.2, 32.67, 32.65, 32.5, 29.64, 29.59, 26.8, 25.6 (mixture of major and minor rotameric forms).
HRMS ($C_{33}H_{40}ClN_3O_5S$): *m/z* (positive mode) = 626.2459 (found $[M+H]^+$), 626.2450 (calc.).

N-Me-HTL2 ligand S14

S8

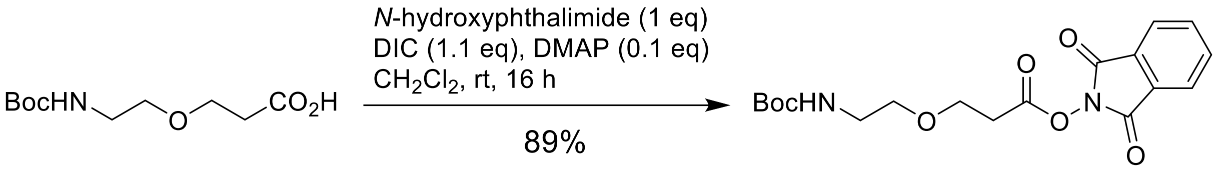

S8

N,N'-Diisopropylcarbodiimide (DIC; 1.71 mL, 11 mmol, 1.1 equiv) was added quickly dropwise to a stirred suspension of 3-(2-*N*-Boc-aminoethoxy)propanoic acid (2.33 g, 10 mmol), *N*-hydroxyphthalimide (1.63 g, 10 mmol, 1 equiv) and 4-(dimethylamino)pyridine (DMAP; 122 mg, 1 mmol, 0.1 equiv) in dry CH_2Cl_2 (50 mL), placed in a rt water bath. Most of the carboxylic acid dissolved by the end of addition, and the urea began to precipitate. After stirring at rt overnight (16 h), the suspension was cooled in ice-water and filtered through a plug of Celite, washing the filter cake with CH_2Cl_2. The filtrate was evaporated on silica, the product was isolated by flash column chromatography (40 g BGB Scorpius 50 µm cartridge, gradient 10% to 60% EtOAc/hexane) and dried *in vacuo* to give 3.38 g (89%) of **S8** as viscous colorless oil.

^{1}H NMR (400 MHz, $CDCl_3$): δ 7.93 – 7.86 (m, 2H), 7.84 – 7.77 (m, 2H), 5.30 (br.s, 1H), 3.85 (t, J = 6.1 Hz, 2H), 3.57 (t, J = 5.0 Hz, 2H), 3.35 (q, J = 5.3 Hz, 2H), 2.93 (t, J = 6.1 Hz, 2H), 1.43 (s, 9H).
^{13}C NMR (101 MHz, $CDCl_3$): δ 167.9, 161.9, 156.3, 134.9, 129.0, 124.1, 79.1, 70.5, 65.8, 40.5, 32.5, 28.5.
HRMS ($C_{18}H_{22}N_2O_7$): *m/z* (positive mode) = 401.1302 (found $[M+Na]^+$), 401.1319 (calc.).

S9

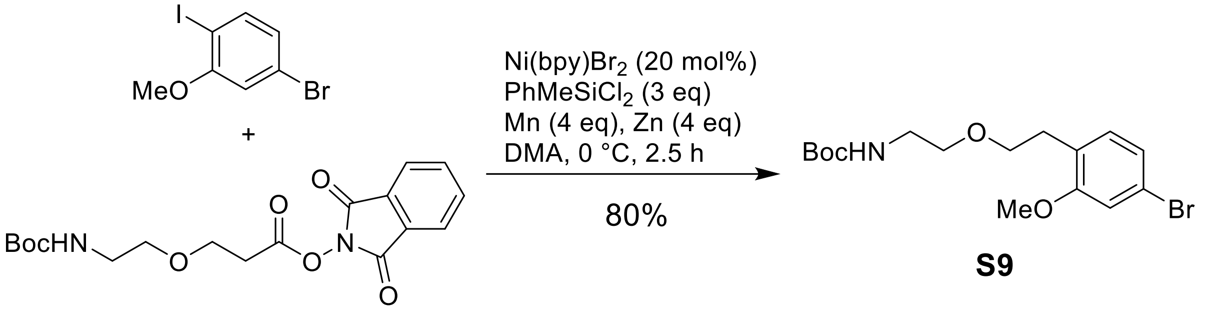

S9

S8
(2 eq)

Following the procedure from [S24], a freshly prepared solution of dichloro(methyl)phenylsilane (1.5 mL, 9 mmol, 3 equiv) in dry DMA (15 mL) was added to the mixture of 4-bromo-1-iodo-2-methoxybenzene (939 mg, 3 mmol), **S8** (2.27 g, 6 mmol, 2 equiv), Ni(bpy)Br_2 catalyst (225 mg, 0.6 mmol, 20 mol%; prepared as described in[S25]), manganese powder (660 mg, 12 mmol, 4 equiv) and zinc dust (780 mg, 12 mmol, 4 equiv) in a 50 mL round-bottom flask, cooled in ice-water bath. The reaction mixture was vigorously stirred at 0 °C for 2.5 h, quickly turning red-orange. It was then diluted with EtOAc and sat. aq. NH_4Cl (100 mL), extracted with EtOAc (3×40 mL), and the combined extracts were dried over Na_2SO_4. The product was isolated by flash column chromatography (40 g BGB Scorpius 50 µm cartridge, gradient 5% to 50% EtOAc/hexane) and dried *in vacuo* to give 903 mg (80%) of **S9** as viscous yellow oil.

^{1}H NMR (400 MHz, $CDCl_3$): δ 7.02 (d, *J* = 1.1 Hz, 2H), 6.97 (d, *J* = 1.1 Hz, 1H), 4.79 (br.s, 1H), 3.81 (s, 3H), 3.59 (t, *J* = 7.0 Hz, 2H), 3.48 (dd, *J* = 5.6, 4.7 Hz, 2H), 3.27 (q, *J* = 4.7 Hz, 2H), 2.83 (t, *J* = 7.0 Hz, 2H), 1.45 (s, 9H).
^{13}C NMR (101 MHz, $CDCl_3$): δ 158.3, 156.1, 131.8, 126.3, 123.5, 120.7, 114.1, 79.4, 70.2, 69.7, 55.7, 40.6, 30.4, 28.6.
HRMS ($C_{16}H_{24}BrNO_4$): *m/z* (positive mode) = 396.0791 (found $[M+Na]^+$), 396.0781 (calc.).

S10

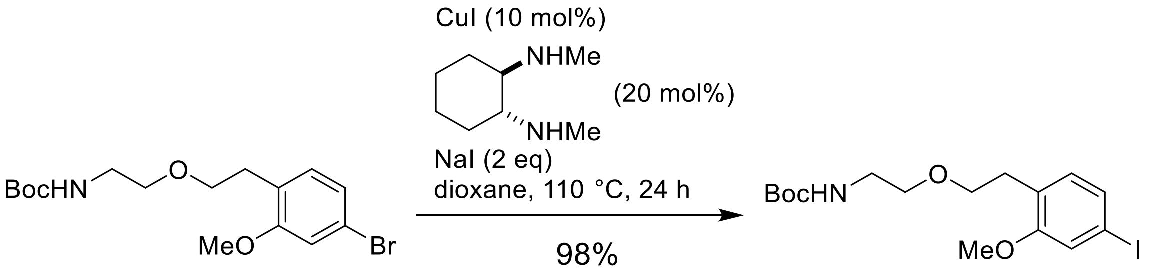

S9 **S10**

Following the procedure from [S26], *trans-N,N'*-dimethylcyclohexane-1,2-diamine (76 µL, 0.482 mmol, 20 mol%) was injected into the stirred, degassed suspension of copper(I) iodide (46 mg, 0.241 mmol, 10 mol%), sodium iodide (724 mg, 4.82 mmol, 2 equiv) and **S9** (903 mg, 2.41 mmol) in 1,4-dioxane (6 mL) in a 25 mL round-bottom flask. The mixture was placed in a preheated 110 °C oil bath and stirred at this temperature for 24 h. On cooling to rt, the mixture was diluted with sat. aq. NH_4Cl (100 mL), extracted with EtOAc (3×25 mL), the combined extracts were washed with brine and dried over Na_2SO_4. The product was isolated by flash column chromatography (25 g Interchim SiHP 30 µm cartridge, gradient 5% to 60% EtOAc/hexane) and dried *in vacuo* to give 996 mg (98%) of **S10** as viscous brownish oil.

^{1}H NMR (400 MHz, $CDCl_3$): δ 7.22 (dd, *J* = 7.8, 1.7 Hz, 1H), 7.13 (d, *J* = 1.7 Hz, 1H), 6.88 (d, *J* = 7.8 Hz, 1H), 4.81 (br.s, 1H), 3.80 (s, 3H), 3.59 (t, *J* = 7.0 Hz, 2H), 3.48 (t, *J* = 5.2 Hz, 2H), 3.27 (q, *J* = 5.2 Hz, 2H), 2.83 (t, *J* = 7.0 Hz, 2H), 1.45 (s, 9H).
^{13}C NMR (101 MHz, $CDCl_3$): δ 158.3, 156.1, 132.2, 129.7, 127.1, 119.8, 91.7, 79.4, 70.2, 69.7, 55.7, 40.5, 30.5, 28.6.
HRMS ($C_{16}H_{24}INO_4$): *m/z* (positive mode) = 444.0651 (found $[M+Na]^+$), 444.0642 (calc.).

S11

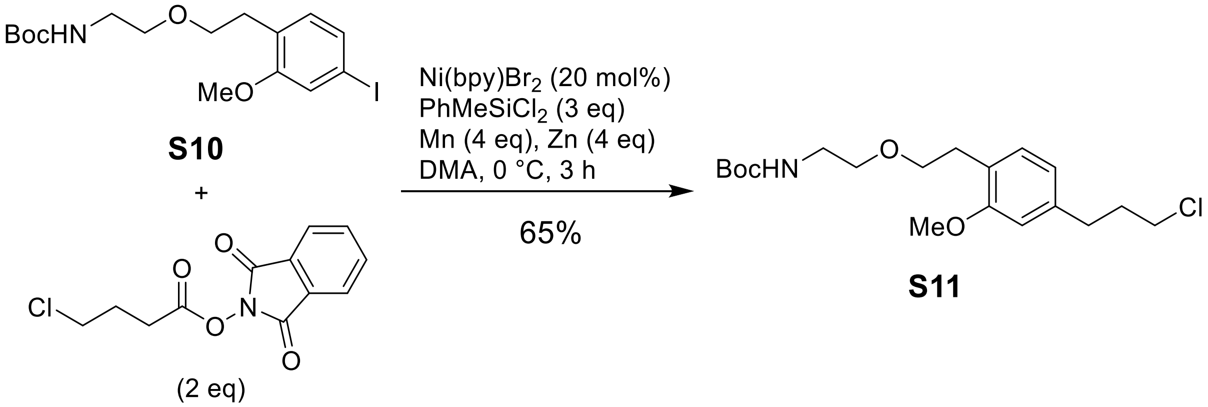

Following the procedure from [S24], a freshly prepared solution of dichloro(methyl)phenylsilane (1.1 mL, 6.78 mmol, 3 equiv) in dry DMA (11 mL) was added to the mixture of **S10** (953 mg, 2.26 mmol), 4-chlorobutyric acid *N*-hydroxyphthalimide ester (1.21 g, 4.52 mmol, 2 equiv; known compound [S27]), Ni(bpy)Br_2 catalyst (170 mg, 0.452 mmol, 20 mol%; prepared as described in [S25]), manganese powder (497 mg, 9.04 mmol, 4 equiv) and zinc dust (588 mg, 9.04 mmol, 4 equiv) in a 25 mL round-bottom flask, cooled in ice-water bath. The reaction mixture was vigorously stirred at 0 °C for 3 h, quickly turning red-orange. It was then diluted with EtOAc and sat. aq. NH_4Cl (100 mL), extracted with EtOAc (4×30 mL), and the combined extracts were washed with brine (50 mL) and dried over Na_2SO_4. The product was isolated by flash column

chromatography (40 g BGB Scorpius 50 μm cartridge, gradient 20% to 80% diethyl ether/hexane) and dried *in vacuo* to give 550 mg (65%) of **S11** as viscous light-yellow oil. Known compound, see [S28].

^{1}H NMR (400 MHz, $CDCl_3$): δ 7.07 (d, *J* = 7.6 Hz, 1H), 6.72 (dd, *J* = 7.6, 1.6 Hz, 1H), 6.68 (d, *J* = 1.6 Hz, 1H), 4.86 (s, 1H), 3.82 (s, 3H), 3.61 (t, *J* = 7.2 Hz, 2H), 3.53 (t, *J* = 6.5 Hz, 2H), 3.50 (t, *J* = 5.3 Hz, 2H), 3.28 (q, *J* = 5.3 Hz, 2H), 2.86 (t, *J* = 7.2 Hz, 2H), 2.75 (dd, *J* = 8.1, 6.7 Hz, 2H), 2.13 – 2.04 (m, 2H), 1.45 (s, 9H).
^{13}C NMR (101 MHz, $CDCl_3$): δ 157.8, 156.1, 140.5, 130.7, 124.8, 120.5, 110.9, 79.3, 70.7, 69.7, 55.4, 44.4, 40.6, 34.2, 33.0, 30.5, 28.6.

S12

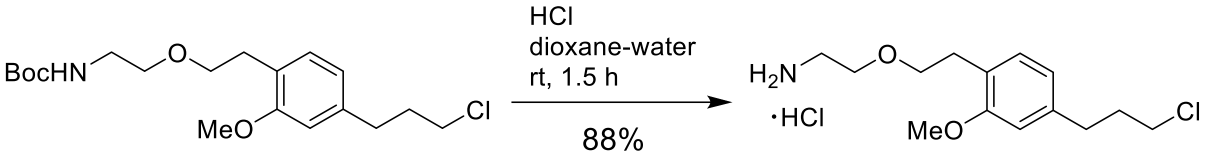

S11 **S12**

Conc. aq. HCl (3 mL) was added to the stirred solution of **S11** (550 mg, 1.25 mmol) in 1,4-dioxane (3 mL), and the resulting solution was stirred at rt for 30 min. It was then diluted with 1,4-dioxane-water mixture (1:1, 10 mL), evaporated to dryness, chased with ethanol and freeze-dried from 1,4-dioxane, yielding 400 mg (88%) of **S12** as white solid (yield estimated for mono-hydrochloride salt). Known compound, see [S28].

^{1}H NMR (400 MHz, $CDCl_3$): δ 8.28 (br.s, 3H), 7.07 (d, *J* = 7.5 Hz, 1H), 6.70 (dd, *J* = 7.5, 1.6 Hz, 1H), 6.66 (d, *J* = 1.6 Hz, 1H), 3.79 (s, 3H), 3.73 (br.t, *J* = 4.8 Hz, 2H), 3.66 (t, *J* = 7.2 Hz, 2H), 3.52 (t, *J* = 6.5 Hz, 2H), 3.20 (br.t, *J* = 4.8 Hz, 2H), 2.88 (t, *J* = 7.2 Hz, 2H), 2.73 (dd, *J* = 8.1, 6.7 Hz, 2H), 2.06 (dq, *J* = 8.1, 6.5 Hz, 2H).
^{13}C NMR (101 MHz, $CDCl_3$): δ 157.7, 140.6, 130.9, 124.5, 120.6, 110.9, 71.1, 66.0, 55.4, 44.5, 39.8, 34.2, 32.9, 30.4.

S13

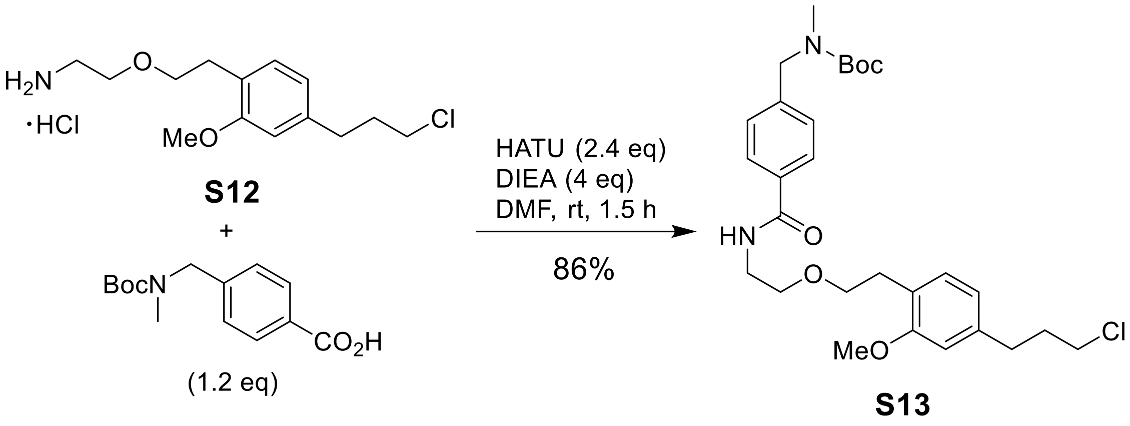

HATU (1-[bis(dimethylamino)methylene]-1*H*-1,2,3-triazolo[4,5-*b*]pyridinium 3-oxid hexafluorophosphate; 547 mg, 1.44 mmol, 2.4 equiv) was added to a stirred solution of 4-(*N*-Boc-*N*-methylamino)methylbenzoic acid (190 mg, 0.72 mmol, 1.2 equiv), compound **S12** (186 mg, 0.6 mmol) and *N*,*N*-diisopropylethylamine (DIEA; 0.42 mL, 2.4 mmol, 4 equiv) in DMF (1.5 mL), and the resulting yellow solution was stirred at rt for 1.5 h. It was then diluted with water (70 mL), extracted with EtOAc (3×25 mL), the combined extracts were washed with brine and dried over Na_2SO_4. The product was isolated by flash column chromatography (25 g Interchim 30 µm SiHP cartridge, gradient 20% to 80% EtOAc/hexane) and dried *in vacuo* to give 323 mg (86%) of **S13** as viscous colorless oil (purity 93-94%, HPLC).

^{1}H NMR (400 MHz, $CDCl_3$): δ 7.69 (br.d, *J* = 7.9 Hz, 2H), 7.29 (br.d, *J* = 7.9 Hz, 2H), 7.08 (d, *J* = 7.9 Hz, 1H), 6.70 – 6.65 (m, 2H), 6.46 (br.s, 1H), 4.47 (br.s, 2H), 3.80 (s, 3H), 3.67 (t, *J* = 7.0 Hz, 2H), 3.63 (br.s, 2H), 3.62 (br.s, 2H), 3.52 (t, *J* = 6.5 Hz, 2H), 2.88 (t, *J* = 7.0 Hz, 2H), 2.81 (s, 3H), 2.73 (t, *J* = 7.1 Hz, 2H), 2.10 – 2.00 (m, 2H), 1.48 (br.s, 9H).
^{13}C NMR (101 MHz, $CDCl_3$): δ 167.2, 157.8, 141.9, 140.5, 133.7, 130.6, 127.8, 127.4, 124.8, 120.5, 110.9, 80.0, 70.6, 69.2, 55.4, 52.6, 51.8, 44.4, 39.9, 34.3, 34.2, 32.9, 30.5, 28.6.
HRMS ($C_{28}H_{39}ClN_2O_5$): *m/z* (positive mode) = 519.2616 (found $[M+H]^+$), 519.2620 (calc.).

S14

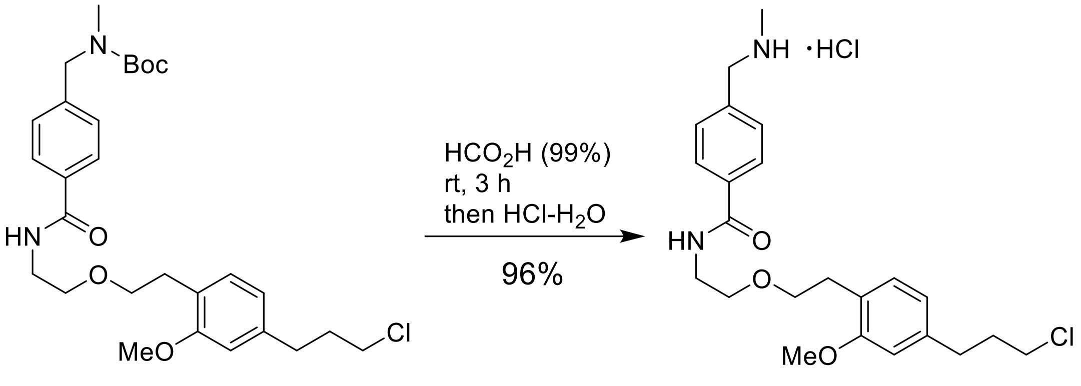

S13 **S14**

Compound **S13** (323 mg, 0.62 mmol) was dissolved in 99% formic acid (3 mL), and the resulting solution was stirred at rt for 3 h. It was then diluted with water and evaporated to dryness, then redissolved in the mixture of 1,4-dioxane (8 mL) and conc. HCl (0.5 mL), evaporated, chased the solid residue twice with methanol and freeze-dried from 1,4-dioxane, yielding 271 mg (96%) of **S14** as white solid (purity 93-94%, HPLC).

^{1}H NMR (400 MHz, $CDCl_3$): δ 9.85 (br.s, 2H), 7.71 (d, *J* = 7.8 Hz, 2H), 7.58 (d, *J* = 7.8 Hz, 2H), 7.13 (br.s, 1H), 7.06 (d, *J* = 7.4 Hz, 1H), 6.71 – 6.65 (m, 2H), 4.07 (br.s, 2H), 3.78 (s, 3H), 3.73 – 3.58 (m, 6H), 3.52 (t, *J* = 6.4 Hz, 2H), 2.88 (t, *J* = 7.1 Hz, 2H), 2.72 (t, *J* = 7.4 Hz, 2H), 2.60 (br.s, 3H), 2.12 – 1.98 (m, 3H).

^{13}C NMR (101 MHz, $CDCl_3$): δ 167.1, 157.8, 140.5, 135.6, 133.4, 130.6, 130.5, 128.0, 124.7, 120.5, 110.9, 70.7, 69.0, 55.4, 52.3, 44.5, 40.1, 34.2, 32.9, 32.5, 30.5.

HRMS ($C_{23}H_{31}ClN_2O_3$): *m/z* (positive mode) = 419.2100 (found $[M+H]^+$), 419.2096 (calc.).

20-HTL2

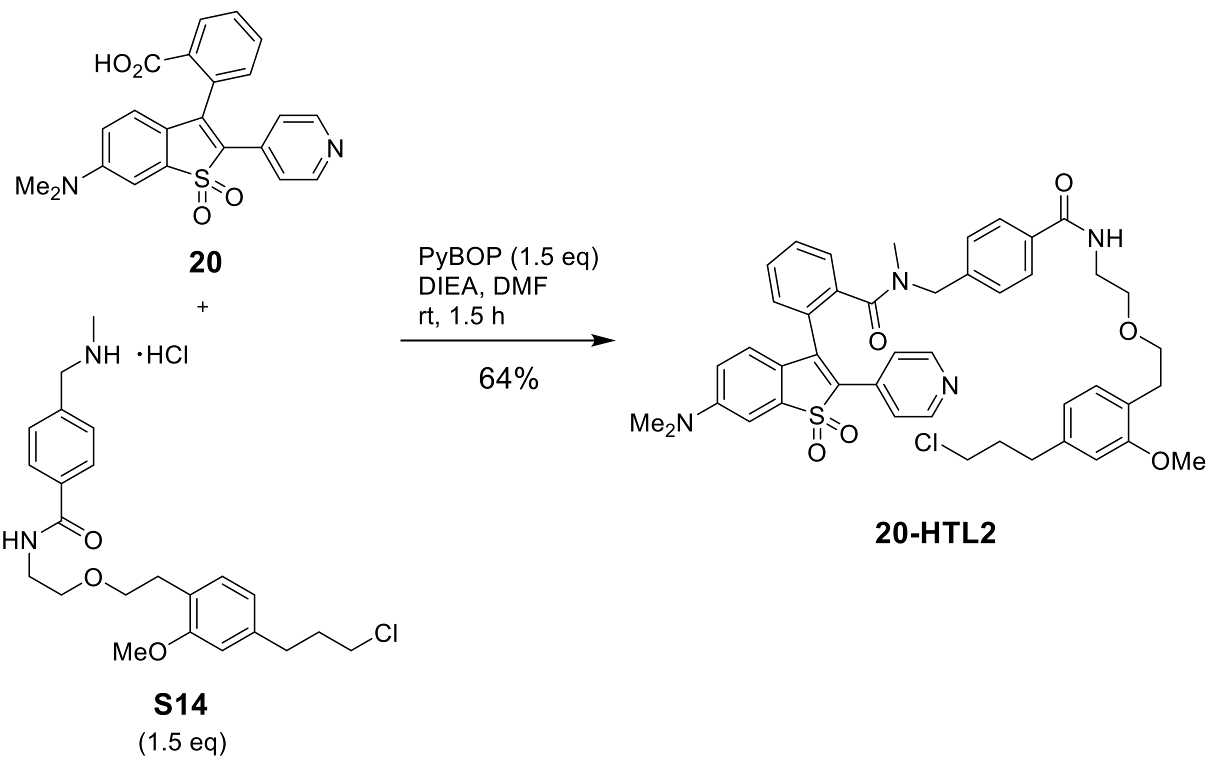

Benzotriazole-1-yl-oxy-tris-pyrrolidino-phosphonium hexafluorophosphate (PyBOP; 15 mg, 28.6 µmol, 1.5 equiv, freshly dissolved in 50 µL DMF) was added to a stirred solution of **20** (7.7 mg, 19 µmol), **S14** (13 mg, 28.6 µmol, 1.5 equiv) and *N*,*N*-diisopropylethylamine (DIEA; 40 µL) in anhydrous DMSO (0.15 mL), and the resulting mixture was stirred at rt for 1.5 h. The solvents were removed *in vacuo*, and the product was isolated by preparative HPLC (ThermoFisher Hypersil Gold C18 250×21.2 mm 5 µm, solvent flow rate 18 mL/min, gradient 30% to 80% A:B, A – acetonitrile + 0.1% (v/v) HCO_2H, B – water + 0.1% (v/v) HCO_2H) and freeze-dried from 1,4-dioxane to give 9.8 mg (64%) of **20-HTL2** as orange-yellow solid.

^{1}H NMR (400 MHz, $CDCl_3$): δ 8.42 – 8.33 (m, 2H), 7.62 (d, *J* = 8.1 Hz, 2H), 7.58 – 7.53 (m, 1H), 7.47 – 7.40 (m, 2H), 7.25 – 7.21 (m, 2H), 7.18 (d, *J* = 8.0 Hz, 2H), 7.13 (d, *J* = 2.4 Hz, 1H), 7.09 (d, *J* = 7.9 Hz, 1H), 6.85 (d, *J* = 8.6 Hz, 1H), 6.67 (dd, *J* = 5.9, 1.7 Hz, 3H), 6.54 (dd, *J* = 8.8, 2.4 Hz, 2H), 4.62 – 4.38 (m, 2H), 3.80 (s, 3H), 3.68 (t, *J* = 6.9 Hz, 2H), 3.66 – 3.59 (m, 4H), 3.50 (t, *J* = 6.5 Hz, 2H), 3.05 (s, 6H), 2.90 (t, *J* = 7.1 Hz, 2H), 2.70 (t, *J* = 7.4 Hz, 2H), 2.41 (br.s, 3H), 2.08 – 1.98 (m, 2H). Mixture of 2 rotameric forms in ~80:20 ratio (only signals of the major rotamer are listed).

^{13}C NMR (101 MHz, $CDCl_3$): δ 168.7, 167.3, 157.8, 152.2, 150.3, 150.1, 142.0, 140.5, 139.9, 138.3, 135.94, 135.90, 133.90, 130.85, 130.68, 130.66, 130.6, 130.09, 130.05, 129.3, 128.9, 127.9, 127.7, 127.5, 126.4, 125.7, 124.8, 122.3, 122.2, 120.5, 119.6, 114.7, 110.9, 104.7, 70.7,

69.2, 55.4, 50.4, 44.5, 44.4, 40.53, 40.49, 40.0, 35.9, 34.2, 33.1, 32.9, 30.49, 30.45 (mixture of major and minor rotameric forms).

HRMS ($C_{45}H_{47}ClN_4O_6S$): *m/z* (positive mode) = 807.2973 (found $[M+H]^+$), 807.2978 (calc.).

N-Me-SNAP ligand ($CPCF_3$-NHMe) S16

S15

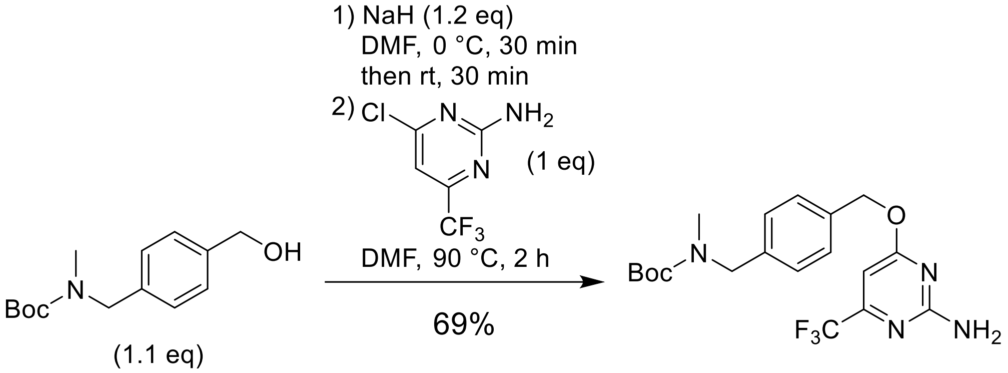

S15

Following the procedure described in [S29], sodium hydride (87 mg of 60 wt% in mineral oil; 2.18 mmol, 1.2 equiv) was suspended in dry DMF (4 mL), cooled in ice-water bath (under argon) in a 25 mL round-bottom flask. *tert*-Butyl 4-(hydroxymethyl)benzyl(methyl)carbamate (502 mg, 2 mmol, 1.1 equiv) was added to the suspension, and it was stirred at 0 °C until hydrogen evolution subsided (~30 min) and then at rt for 30 min. To the resulting nearly clear brownish solution, 2-amino-4-chloro-6-(trifluoromethyl)pyrimidine (360 mg, 1.82 mmol) was added in one portion, the reaction flask was briefly degassed on a Schlenk line and placed in a preheated 90 °C oil bath. The mixture was stirred at 90 °C for 2 h, cooled down to rt, diluted with water (75 mL) and extracted with EtOAc (3×25 mL), the combined extracts were washed with brine and dried over Na_2SO_4. The less polar product was isolated by flash column chromatography (25 g Interchim SiHP 30 µm cartridge, gradient 20% to 80% EtOAc/hexane), yielding 516 mg (69%) of **S15** as viscous colorless oil.

^{1}H NMR (400 MHz, $CDCl_3$): 7.38 (d, *J* = 8.0 Hz, 2H), 7.25 (br.d, *J* = 7.6 Hz, 2H), 6.43 (s, 1H), 5.59 (br.s, 2H), 5.36 (s, 2H), 4.43 (s, 2H), 2.82 (app.d, *J* = 13.2 Hz, 3H), 1.48 (s, 9H).
^{19}F NMR (376 MHz, $CDCl_3$): δ -70.82.
^{13}C NMR (101 MHz, $CDCl_3$): δ 171.1, 163.3, 157.0 (q, *J* = 34.9 Hz), 156.3, 155.9, 138.6, 134.8, 128.6, 128.0, 127.6, 120.7 (q, *J* = 274.6 Hz), 95.6 (q, *J* = 3.2 Hz), 79.9, 68.3, 52.5, 51.8, 34.1, 28.6.
HRMS ($C_{19}H_{23}F_3N_4O_3$): *m/z* (positive mode) = 413.1793 (found $[M+H]^+$), 413.1795 (calc.).

S16

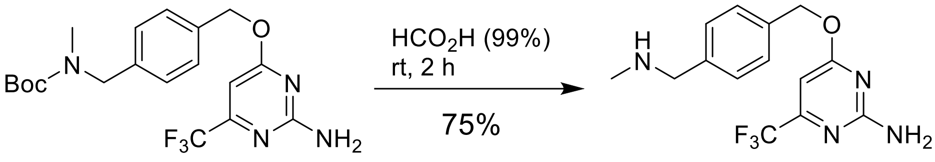

S15 **S16**

A solution of **S15** (350 mg, 0.85 mmol) in 99% formic acid (4 mL) was stirred at rt for 2 h. The mixture was then diluted with water (1:1), the solvents were removed on a rotavapor and the residue was chased with water twice. Free base was recovered from the resulting crude formate salt by flash column chromatography (25 g Interchim SiHP 30 µm cartridge, gradient 0% to 80% A/B, A = CH_2Cl_2 – ethanol - 25% aq. NH_3 80:20:2, B = CH_2Cl_2) and freeze-dried from 1,4-dioxane to give 199 mg (75%) of **S16** as white solid.

^{1}H NMR (400 MHz, DMSO-d_6): δ 7.43 – 7.37 (m, 2H), 7.35 – 7.30 (m, 2H), 7.26 (br.s, 2H), 6.43 (s, 1H), 5.35 (s, 2H), 3.63 (s, 2H), 2.24 (s, 3H).
^{19}F NMR (376 MHz, DMSO-d_6): δ -69.56.
^{13}C NMR (101 MHz, DMSO-d_6): δ 170.4, 163.7, 155.9 (q, J = 34.0 Hz), 140.9, 134.1, 128.3, 127.9, 120.7 (q, J = 274.7 Hz), 92.9 (q, J = 3.2 Hz), 67.5, 54.8, 35.6.
HRMS ($C_{14}H_{15}F_3N_4O$): *m/z* (positive mode) = 313.1267 (found $[M+H]^+$), 313.1271 (calc.).

20-SNAP

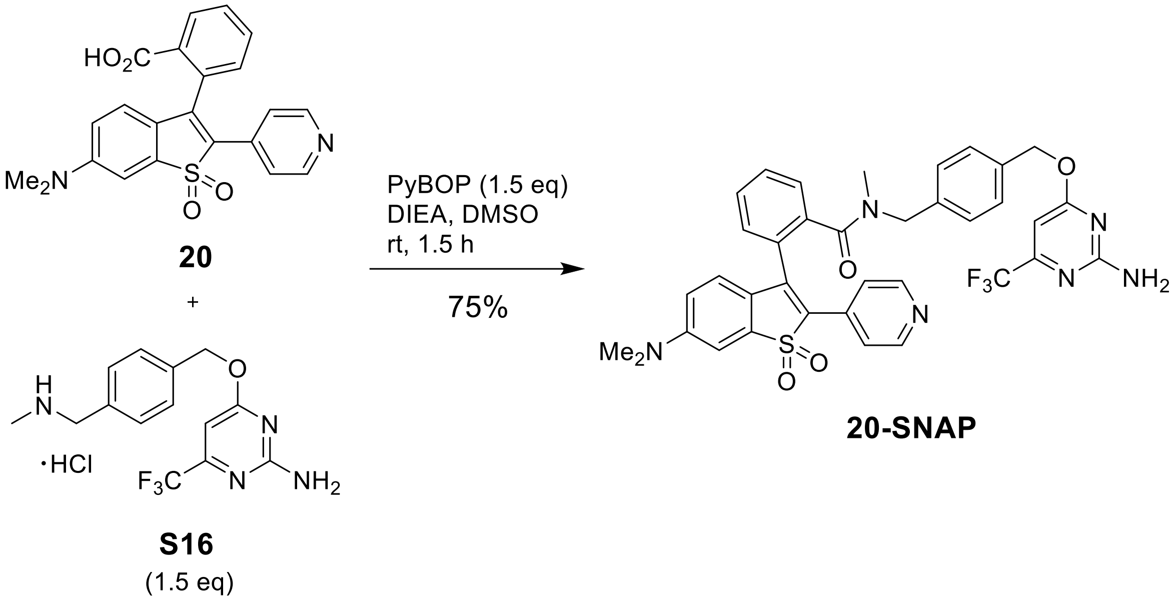

Benzotriazole-1-yl-oxy-tris-pyrrolidino-phosphonium hexafluorophosphate (PyBOP; 19.2 mg, 36.9 µmol, 1.5 equiv) was added to a stirred solution of **20** (10 mg, 24.6 µmol), **AB1-996** (14 mg, 36.9 µmol, 1.5 equiv) and *N*,*N*-diisopropylethylamine (DIEA; 70 µL) in anhydrous DMSO (0.3 mL), and the resulting mixture was stirred at rt for 1.5 h. The volatiles were removed *in vacuo*, and the product was isolated by preparative HPLC (ThermoFisher Hypersil Gold C18 250×21.2 mm 5 µm, solvent flow rate 18 mL/min, gradient 20% to 80% A:B, A – acetonitrile + 0.1% (v/v) HCO_2H, B – water + 0.1% (v/v) HCO_2H) to give 13 mg (75%) of **20-SNAP** as orange-yellow solid.

^{1}H NMR (400 MHz, CD_3CN): δ 8.36 – 8.26 (m, 2H), 7.67 – 7.45 (m, 4H), 7.30 (d, *J* = 7.8 Hz, 2H), 7.25 – 7.19 (m, 3H), 7.06 (d, *J* = 8.0 Hz, 2H), 6.82 (d, *J* = 8.7 Hz, 1H), 6.66 (dd, *J* = 8.7, 2.5 Hz, 1H), 6.46 (s, 1H), 5.80 (br.s, 2H), 5.42 – 5.30 (m, 3H), 4.50 (d, *J* = 14.5 Hz, 1H), 4.40 (d, *J* = 14.5 Hz, 1H), 3.05 (s, 6H), 2.43 (s, 3H). Mixture of 2 rotameric forms in ~85:15 ratio (only signals of the major rotamer are listed).

^{19}F NMR (376 MHz, CD_3CN): δ -71.40.

^{13}C NMR (101 MHz, CD_3CN): δ 150.9, 130.9, 130.6, 129.7, 128.7, 126.8, 123.2, 116.0, 105.2, 95.3, 68.7, 50.5, 40.8, 36.5 (indirect detection from a gHSQC experiment, only H-coupled carbons are resolved).

HRMS ($C_{36}H_{31}F_3N_6O_4S$): *m/z* (positive mode) = 701.2162 (found $[M+H]^+$), 701.2152 (calc.).

N-Me-CLIP ligand (BC-NHMe) S18

S17

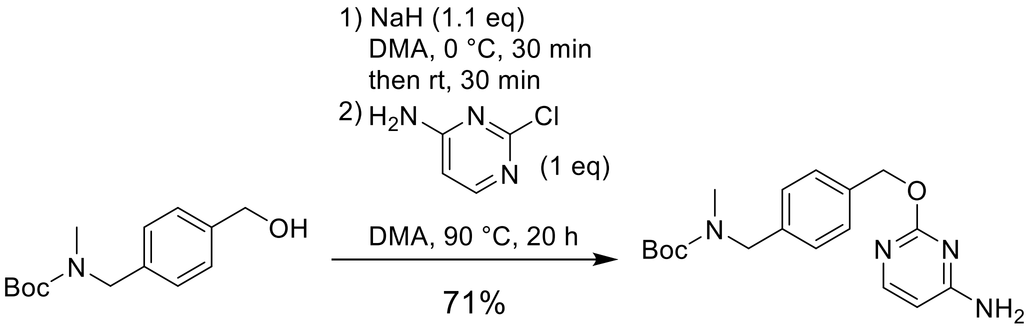

S17

Following the procedure described in [S29], sodium hydride (53 mg of 60 wt% in mineral oil; 1.32 mmol, 1.1 equiv) was suspended in dry DMA (2.4 mL), cooled in ice-water bath (under argon) in a 25 mL round-bottom flask. *tert*-Butyl 4-(hydroxymethyl)benzyl(methyl)carbamate (300 mg, 1.2 mmol, 1 equiv) was added to the suspension, and it was stirred at 0 °C until hydrogen evolution subsided (~30 min) and then at rt for 30 min. To the resulting solution, 4-amino-2-

chloropyrimidine (156 mg, 1.2 mmol) was added in one portion, the reaction flask was briefly degassed on a Schlenk line and placed in a preheated 90 °C oil bath. The mixture was stirred at 90 °C overnight (20 h), the resulting light green suspension was cooled down to rt, diluted with water (150 mL) and extracted with EtOAc (3×30 mL), the combined extracts were washed with water (2×100 mL), brine and dried over Na_2SO_4. The less polar product was isolated by flash column chromatography (25 g Interchim SiHP 30 µm cartridge, gradient 50% to 100% EtOAc/hexane) and dried *in vacuo*, yielding 295 mg (71%) of **S17** as white foam.

^{1}H NMR (400 MHz, $CDCl_3$): δ 7.99 (d, *J* = 5.7 Hz, 1H), 7.41 (d, *J* = 7.8 Hz, 2H), 7.20 (d, *J* = 7.6 Hz, 2H), 6.09 (d, *J* = 5.7 Hz, 1H), 5.34 (s, 2H), 5.22 (s, 2H), 4.41 (s, 2H), 2.81 (app.d, *J* = 13.7 Hz, 3H), 1.47 (s, 9H).
^{13}C NMR (101 MHz, $CDCl_3$): δ 165.1, 165.0, 157.3, 137.7, 136.0, 128.2, 127.7, 127.3, 99.7, 79.8, 68.3, 52.5, 51.8, 34.0, 28.5.
HRMS ($C_{18}H_{24}N_4O_3$): *m/z* (positive mode) = 345.1917 (found $[M+H]^+$), 345.1921 (calc.).

S18

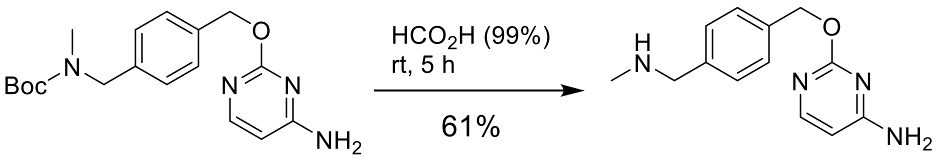

S17 **S18**

Compound **S17** (295 mg, 0.86 mmol) was dissolved in 99% formic acid (3 mL), and the resulting solution was stirred at rt for 5 h. It was then diluted with water and evaporated to dryness, then redissolved in methanol-CH_2Cl_2 mixture and evaporated on silica. The product was isolated by flash column chromatography (25 g Interchim SiHP 30 µm cartridge, gradient 0% to 80% A/B, A = CH_2Cl_2 – ethanol –25% aq. NH_3 60:40:4, B = CH_2Cl_2) and freeze-dried from aqueous 1,4-dioxane, yielding 127 mg (61%) of **S18** as white solid.

^{1}H NMR (400 MHz, DMSO-*d*$_6$): δ 7.86 (d, *J* = 5.7 Hz, 1H), 7.34 (d, *J* = 8.2 Hz, 2H), 7.29 (d, *J* = 8.2 Hz, 2H), 6.86 (br.s, 2H), 6.08 (d, *J* = 5.7 Hz, 1H), 5.22 (s, 2H), 3.62 (s, 2H), 2.24 (s, 3H).
^{13}C NMR (101 MHz, DMSO-*d*$_6$): δ 165.4, 164.7, 156.2, 140.3, 135.6, 127.8, 127.64, 127.58, 126.3, 99.5, 67.1, 54.8, 35.6.
HRMS ($C_{13}H_{16}N_4O$): *m/z* (positive mode) = 245.1408 (found $[M+H]^+$), 245.1397 (calc.).

20-CLIP

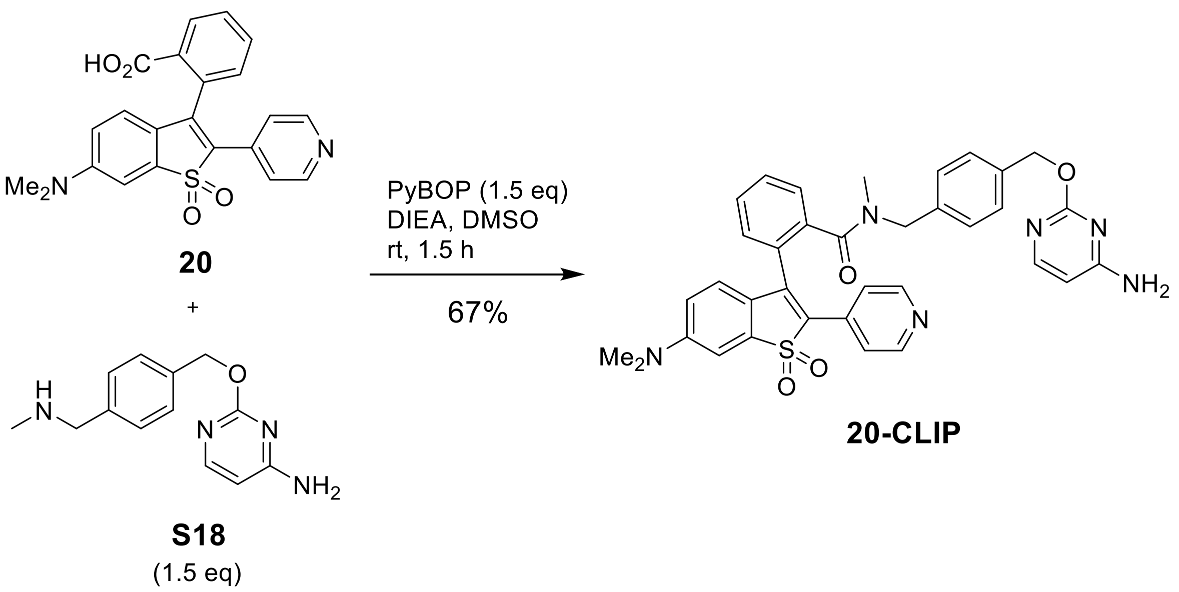

Benzotriazole-1-yl-oxy-tris-pyrrolidino-phosphonium hexafluorophosphate (PyBOP; 19.2 mg, 36.9 µmol, 1.5 equiv) was added to a stirred solution of **20** (10 mg, 24.6 µmol), **S18** (9 mg, 36.9 µmol, 1.5 equiv) and *N*,*N*-diisopropylethylamine (DIEA; 50 µL) in anhydrous DMSO (0.2 mL), and the resulting mixture was stirred at rt for 1.5 h. The volatiles were removed *in vacuo*, and the product was isolated by preparative HPLC (ThermoFisher Hypersil Gold C18 250×21.2 mm 5 µm, solvent flow rate 18 mL/min, gradient 10% to 60% A:B, A – acetonitrile + 0.1% (v/v) HCO_2H, B – water + 0.1% (v/v) HCO_2H) and freeze-dried from 1,4-dioxane to give 10.5 mg (67%) of **20-CLIP** as yellow-orange solid.

^{1}H NMR (400 MHz, DMSO-d_6): δ 8.43 – 8.33 (m, 2H), 7.89 (d, J = 5.7 Hz, 1H), 7.64 – 7.47 (m, 4H), 7.31 (d, J = 2.4 Hz, 1H), 7.25 – 7.17 (m, 3H), 6.99 (d, J = 7.9 Hz, 2H), 6.87 (br.s, 2H), 6.79 (d, J = 8.7 Hz, 1H), 6.69 (dd, J = 8.8, 2.5 Hz, 1H), 6.10 (d, J = 5.7 Hz, 1H), 5.22 (s, 2H), 4.54 (d, J = 14.4 Hz, 1H), 4.30 (d, J = 14.4 Hz, 1H), 3.05 (s, 6H), 2.62 (s, 0.45H, N–CH_3, minor rotamer), 2.45 (s, 2.55H, N–CH_3, major rotamer). Mixture of 2 rotameric forms in ~85:15 ratio (only signals of the major rotamer are listed).
^{13}C NMR (101 MHz, DMSO-d_6): δ 167.8, 165.5, 164.7, 156.2, 152.1, 149.8, 142.0, 137.9, 136.2, 136.0, 135.5, 135.4, 130.1, 129.9, 129.7, 129.6, 129.2, 128.0, 127.7, 125.4, 121.8, 118.4, 114.9, 104.5, 99.6, 66.9, 49.1, 40.1, 35.5 (mixture of major and minor rotameric forms).
HRMS ($C_{35}H_{32}N_6O_4S$): *m/z* (positive mode) = 317.1178 (found $[M+2H]^{2+}$), 317.1176 (calc.).

Preparation of fluorescent ligands of large Stokes dye SiX

SiX-Halo

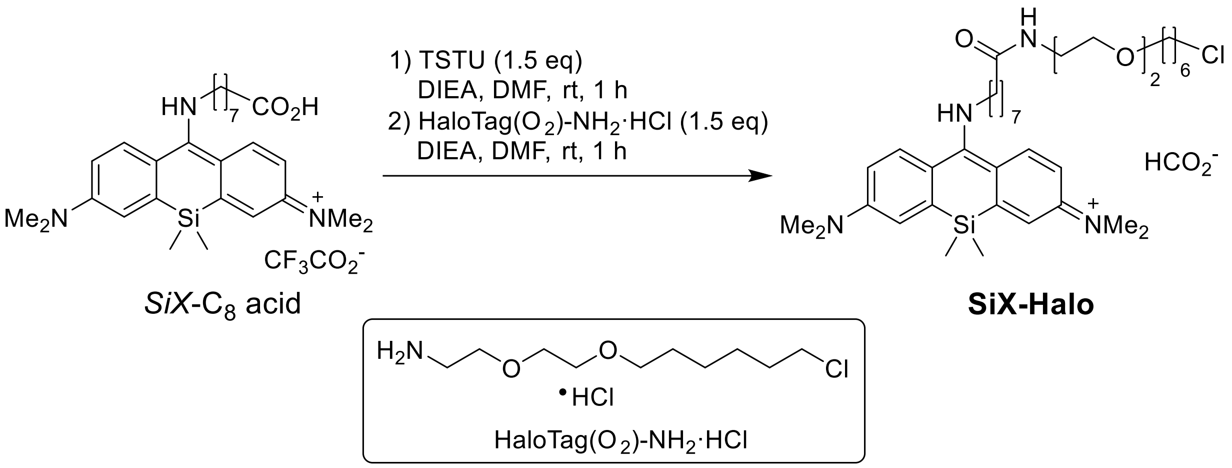

N,*N*,*N′*,*N′*-Tetramethyl-*O*-(*N*-succinimidyl)uronium tetrafluoroborate (TSTU; 13 mg, 42.7 µmol, 1.5 equiv) in 50 µL dry DMF was added to the solution of *SiX*-C_8 acid (trifluoroacetate salt, 16.5 mg, 28.5 µmol; known compound[S30]) and *N*,*N*-diisopropylethylamine (DIEA; 60 µL) in DMF (150 µL). The reaction mixture was stirred at rt for 1 h (LC-MS control showed complete conversion into the NHS ester). A solution of 2-(2-(6-chlorohexyloxy)ethoxy)ethanamine hydrochloride (HaloTag(O2) amine hydrochloride[S31]; 11 mg, 42.7 µmol, 1.5 equiv) in DMF (100 µL) was then added followed by DIEA (50 µL). The reaction mixture was stirred at rt for 1 h, and the volatiles were removed *in vacuo*. The product was isolated by preparative HPLC (Interchim Uptisphere PhC4 5 µm 250×21.2 mm, gradient 30/70 to 90/10 A:B, A = 0.1% HCO_2H in acetonitrile, B = 0.1% HCO_2H in water, detection at 460 nm) and freeze-dried from aq. dioxane. Got 12 mg (63%) of **SiX-Halo** as viscous orange oil (formate salt).

^{1}H NMR (400 MHz, $CDCl_3$): δ 8.64 (br.s, 1H), 8.12 (d, *J* = 8.7 Hz, 1H), 7.50 (d, *J* = 8.9 Hz, 1H), 6.93 (d, *J* = 2.8 Hz, 1H), 6.88 – 6.79 (m, 2H), 6.75 (dd, *J* = 8.9, 2.8 Hz, 1H), 6.27 (br.t, *J* = 5.8 Hz, 1H), 3.96 (t, *J* = 7.2 Hz, 2H), 3.66 – 3.41 (m, 12H), 3.13 (s, 6H), 3.07 (s, 6H), 2.13 (t, *J* = 7.6 Hz, 2H), 1.93 – 1.85 (m, 2H), 1.84 – 1.72 (m, 2H), 1.65 – 1.53 (m, 4H), 1.51 – 1.23 (m, 10H), 0.48 (s, 6H); minor signals correspond to the free base form of **SiX-Halo** and are not listed.
^{13}C NMR (101 MHz, $CDCl_3$): δ 173.4, 151.7, 151.3, 141.9, 138.2, 131.1, 130.1, 116.2, 115.1, 113.5, 111.4, 71.4, 70.4, 70.2, 70.0, 50.7, 45.2, 40.2, 40.1, 39.2, 36.7, 32.7, 32.6, 29.6, 29.2, 28.8, 28.5, 26.8, 26.5, 25.6, 25.5, -2.1.
HRMS ($C_{37}H_{59}ClN_4O_3Si$): *m/z* (positive mode) = 671.4129 (found $[M+H]^+$), 671.4118 (calc.).

SiX-HTL2

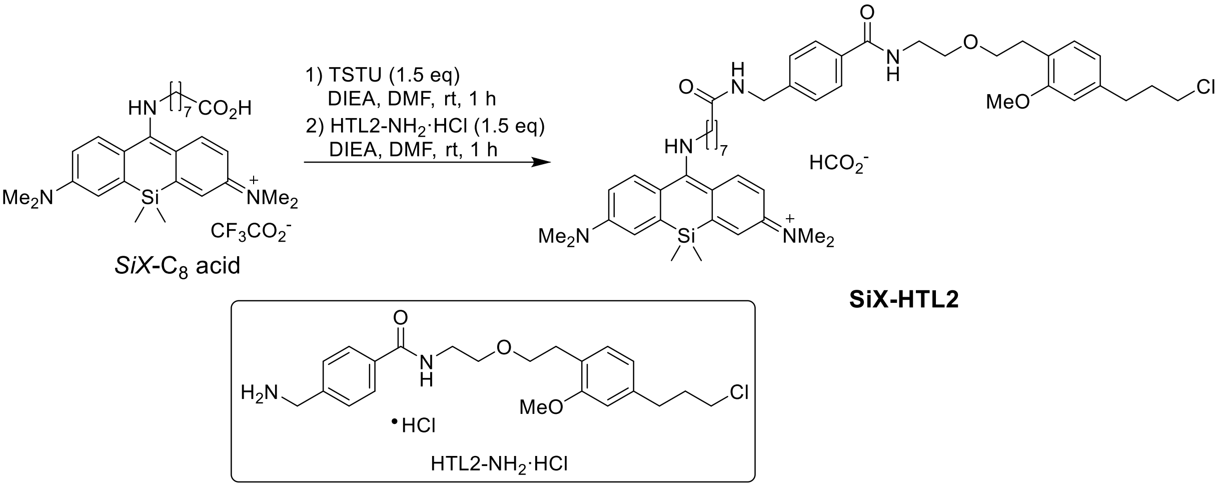

N,*N*,*N′*,*N′*-Tetramethyl-*O*-(*N*-succinimidyl)uronium tetrafluoroborate (TSTU; 13 mg, 42.7 µmol, 1.5 equiv) in 50 µL dry DMF was added to the solution of *SiX*-C_8 acid (trifluoroacetate salt, 16.5 mg, 28.5 µmol; known compound[S30]) and *N*,*N*-diisopropylethylamine (DIEA; 60 µL) in DMF (150 µL). The reaction mixture was stirred at rt for 1 h (LC-MS control showed complete conversion into the NHS ester). A solution of 4-(aminomethyl)-*N*-(2-(4-(3-chloropropyl)-2-methoxyphenethoxy)ethyl)benzamide hydrochloride (HTL2 amine hydrochloride[S28]; 19 mg, 42.7 µmol, 1.5 equiv) in DMF (100 µL) was then added followed by DIEA (50 µL). The reaction mixture was stirred at rt for 1 h, and the volatiles were removed *in vacuo*. The product was isolated by preparative HPLC (Interchim Uptisphere PhC4 5 µm 250×21.2 mm, gradient 30/70 to 90/10 A:B, A = 0.1% HCO_2H in acetonitrile, B = 0.1% HCO_2H in water, detection at 460 nm), repurified by preparative HPLC (Thermo Fisher Scientific Hypersil Gold C18 5 µm 250×20 mm, gradient 30/70 to 100/0 A:B, A = 0.1% HCO_2H in acetonitrile, B = 0.1% HCO_2H in water, detection at 460 nm) and freeze-dried from aq. dioxane. Got 15 mg (62%) of **SiX-HTL2** as viscous orange oil (formate salt).

^{1}H NMR (400 MHz, $CDCl_3$): δ 8.57 (br.s, 1H), 8.09 (d, *J* = 8.9 Hz, 1H), 7.70 – 7.64 (m, 2H), 7.49 (d, *J* = 8.9 Hz, 1H), 7.40 (t, *J* = 5.8 Hz, 1H), 7.36 – 7.29 (m, 2H), 7.11 – 7.03 (m, 1H), 6.93 (d, *J* = 2.8 Hz, 1H), 6.83 – 6.72 (m, 2H), 6.71 – 6.64 (m, 3H), 4.41 (d, *J* = 5.8 Hz, 2H), 3.95 (t, *J* = 7.0 Hz, 2H), 3.79 (s, 3H), 3.66 (t, *J* = 7.0 Hz, 2H), 3.64 – 3.58 (m, 4H), 3.52 (t, *J* = 6.4 Hz, 2H), 3.12 (s, 6H), 3.05 (s, 6H), 2.87 (t, *J* = 7.0 Hz, 2H), 2.72 (t, *J* = 7.5 Hz, 2H), 2.22 (t, *J* = 7.5 Hz, 2H), 2.10 – 2.00 (m, 2H), 1.91 – 1.80 (m, 2H), 1.70 – 1.55 (m, 2H), 1.36 – 1.21 (m, 6H), 0.48 (s, 6H); minor signals correspond to the free base form of **SiX-HTL2** and are not listed.
^{13}C NMR (101 MHz, $CDCl_3$): δ 173.8, 173.6, 167.4, 157.8, 151.6, 151.3, 142.9, 141.8, 140.5, 138.3, 133.4, 131.0, 130.6, 130.5, 127.8, 127.5, 127.3, 126.1, 124.8, 121.5, 120.5, 116.2, 115.1,

113.0, 111.4, 110.9, 70.6, 69.2, 55.4, 50.3, 44.5, 43.0, 40.1, 39.8, 36.3, 34.2, 32.9, 30.48, 30.45, 28.8, 28.3, 28.0, 26.2, 25.4, -2.1.

HRMS ($C_{49}H_{66}ClN_5O_4Si$): *m/z* (positive mode) = 852.4637 (found $[M+H]^+$), 852.4645 (calc.).

Supplementary references

[S1] Kompa, J.; Bruins, J.; Glogger, M.; Wilhelm, J.; Frei, M. S.; Tarnawski, M.; D'Este, E.; Heilemann, M.; Hiblot, J.; Johnsson, K. Exchangeable HaloTag Ligands for Super-Resolution Fluorescence Microscopy. *J. Am. Chem. Soc.* **2023**, *145(5)*, 3075-3083; https://doi.org/10.1021/jacs.2c11969.

[S2] Butkevich, A. N.; Ta, H.; Ratz, M.; Stoldt, S.; Jakobs, S.; Belov, V. N.; Hell, S. W. Two-Color 810 nm STED Nanoscopy of Living Cells with Endogenous SNAP-Tagged Fusion Proteins. *ACS Chem. Biol.* **2017**, *13(2)*, 475-480; https://doi.org/10.1021/acschembio.7b00616.

[S3] Sahl, S. J.; Matthias, J.; Kaushik, I.; Weber, M.; Khan, T. A.; Brüser, C.; Jakobs, S.; Becker, S.; Griesinger, C.; Broichhagen, J.; Hell, S. W. Direct optical measurement of intramolecular distances with angstrom precision. *Science* **2024**, *386(6718)*, 180-187; https://doi.org/10.1126/science.adj7368.

[S4] Rao, M. L. N.; Ramakrishna, B. S. Rhodium-Catalyzed Directing-Group-Assisted Aldehydic C–H Arylations with Aryl Halides. *Eur. J. Org. Chem.* **2017**, *2017(34)*, 5080-5093; https://doi.org/10.1002/ejoc.201700881.

[S5] Rong, B.; Xu, G.; Yan, H.; Zhang, S.; Wu, Q.; Zhu, N.; Fang, Z.; Duan, J.; Guo, K. Synthesis of benzofuro- and benzothieno[2,3-*c*]pyridines *via* copper-catalyzed [4 + 2] annulation of ketoxime acetates with acetoacetanilide. *Org. Chem. Front.* **2021**, *8*, 2939-2943; https://doi.org/10.1039/D1QO00094B.

[S6] Wang, D.; Cui, S. Rh(III)-catalyzed aldehyde C–H bond functionalization of salicylaldehydes with arylboronic acids. *Tetrahedron* **2015**, *71(45)*, 8511-8516; https://doi.org/10.1016/j.tet.2015.09.053.

[S7] Huo, J.; Fu, Y.; Tang, M. J.; Liu, P.; Dong, G. Escape from Palladium: Nickel-Catalyzed Catellani Annulation. *J. Am. Chem. Soc.* **2023**, *145(20)*, 11005-11011; https://doi.org/10.1021/jacs.3c03780.

[S8] Knorr, G.; Bossi, M. L.; Butkevich, A. N.; Hell, S. W. Synthesis of Thioxanthone 10,10-Dioxides and Sulfone-Fluoresceins via Pd-Catalyzed Sulfonylative Homocoupling. *Org. Lett.* **2024**, *26(4)*, 945-949; https://doi.org/10.1021/acs.orglett.3c04300.

[S9] Grazia Cabiddu, M.; Cabiddu, S.; Cadoni, E.; Demontis, S.; Fattuoni, C.; Melis, S. A convenient synthesis of benzothiophene derivatives. *Tetrahedron* **2002**, *58(22)*, 4529-4533; https://doi.org/10.1016/S0040-4020(02)00399-X.

[S10] Ma, Y.; Wang, K.; Zhang, D.; Sun, P. Solvent Controlled Transformation between Sulfonyl Hydrazides and Alkynes: Divergent Synthesis of Benzo[*b*]thiophene-1,1-dioxides and (*E*)-β-iodo Vinylsulfones. *Adv. Synth. Catal.* **2019**, *361(3)*, 597-602; https://doi.org/10.1002/adsc.201801258.

[S11] Tian, Y.; Qi, J.; Sun, C.; Yin, D.; Wang, X.; Xiao, Q. One-pot synthesis of 4-methylisoquinolines *via* a sequential Pd-catalyzed Heck reaction and intramolecular cyclization. *Org. Biomol. Chem.* **2013**, *11*, 7262-7266; https://doi.org/10.1039/C3OB41680A.

[S12] Liu, G.; Zhang, H.; Huang, Y.; Han, Z.; Liu, G.; Liu, Y.; Dong, X.-Q.; Zhang, X. Efficient synthesis of chiral 2,3-dihydro-benzo[b]thiophene 1,1-dioxides via Rh-catalyzed hydrogenation. *Chem. Sci.* **2019**, *10*, 2507-2512; https://doi.org/10.1039/c8sc05397a.

[S13] Okazaki, T.; Yamamoto, H.; Kitagawa, T. Ring-closure reaction of 2-benzoylbenzenediazonium salts in 1-butyl-3-methylimidazolium ionic liquids. *Arkivoc* **2018**, *ii*, 50-59; https://doi.org/10.24820/ark.5550190.p010.195.

[S14] Wang, M.; Yuen, O. Y.; So, C. M. Palladium-Catalyzed Desulfinative Cross-Coupling of Polyhalogenated Aryl Triflates with Aryl Sulfinate Salts: Inversion of Traditional Chemoselectivity. *Chin. J. Chem.* **2023**, *41(8)*, 909-914; https://doi.org/10.1002/cjoc.202200695.

[S15] Otsuka, R.; Maruhashi, K.; Ohwada, T. Latent Brønsted Base Solvent-Assisted Amide Formation from Amines and Acid Chlorides. *Synthesis* **2018**, *50(10)*, 2041-2057; https://doi.org/10.1055/s-0037-1609342.

[S16] Ma, L.; Li, S.; Zheng, H.; Chen, J.; Lin, L.; Ye, X.; Chen, Z.; Xu, Q.; Chen, T.; Yang, J.; Qiu, N.; Wang, G.; Peng, A.; Ding, Y.; Wie, Y.; Chen, L. Synthesis and biological activity of novel barbituric and thiobarbituric acid derivatives against non-alcoholic fatty liver disease. *Eur. J. Med. Chem.* **2011**, *46(6)*, 2003-2010; https://doi.org/10.1016/j.ejmech.2011.02.033.

[S17] Jahnsen, B.; Studer, A. Perfluoroalkylation of Aryl-*N*,*N*-dimethyl Hydrazones Using Hypervalent Iodine(III) Reagents or Perfluoroalkyl Iodides. *J. Org. Chem.* **2017**, *82(22)*, 11703-11710; https://doi.org/10.1021/acs.joc.7b00934.

[S18] Morimoto, T.; Fuji, K.; Tsutsumi, K.; Kakiuchi, K. CO-Transfer Carbonylation Reactions. A Catalytic Pauson−Khand-Type Reaction of Enynes with Aldehydes as a Source of Carbon Monoxide. *J. Am. Chem. Soc.* **2002**, *124(15)*, 3806-3807; https://doi.org/10.1021/ja0126881.

[S19] Levin, V. V.; Gritsenko, R. T.; Dilman, A. D.; Belyakov, P. A.; Korlyukov, A. A.; Arkhipov, D. E.; Struchkova, M. I.; Tartakovsky, V. A. Chelation-assisted pentafluorophenylation of oximes. *Mendeleev Commun.* **2010**, *20*, 220-222; https://doi.org/10.1016/j.mencom.2010.06.013.

[S20] Higson, S.; Subrizi, F.; Sheppard, T. D.; Hailes, H. C. Chemical cascades in water for the synthesis of functionalized aromatics from furfurals. *Green Chem.* **2016**, *18*, 1855-1858; https://doi.org/10.1039/C5GC02935J.

[S21] Ji, Y.; Sweeney, J.; Zoglio, J.; Gorin, D. J. Catalytic Methyl Transfer from Dimethylcarbonate to Carboxylic Acids. *J. Org. Chem.* **2013**, *78(22)*, 11606-11611; https://doi.org/10.1021/jo401941v.

[S22] Song, X.; Bian, H.; Wang, C.; Hu, M.; Li, N.; Xiao, Y. Development and applications of a near-infrared dye–benzylguanine conjugate to specifically label SNAP-tagged proteins. *Org. Biomol. Chem.* **2017**, *15*, 8091-8101; https://doi.org/10.1039/C3OB41680A.

[S23] Neklesa, T. K.; Tae, H. S.; Schneekloth, A. R.; Stulberg, M. J.; Corson, T. W.; Sundberg, T. B.; Raina, K.; Holley, S. A.; Crews, C. M. Small-molecule hydrophobic tagging–induced degradation of HaloTag fusion proteins. *Nat. Chem. Biol.* **2011**, *7*, 538-543; https://doi.org/10.1038/nchembio.597.

[S24] Gabbey, A. L.; Michel, N. W. M.; Hughes, J. M. E.; Campeau, L.-C.; Rousseaux, S. A. L. Synthesis of α-Aryl Secondary Amides via Nickel-Catalyzed Reductive Coupling of Redox-Active Esters. *Org. Lett.* **2022**, *24(17)*, 3173-3178; https://doi.org/10.1021/acs.orglett.2c00918.

[S25] Sheta, A. M.; Fernández, S.; Liu, C.; Dubed-Bandomo, G. C.; Lloret-Fillol, J. An Electrocatalytic Cascade Reaction for the Synthesis of Ketones Using CO_2 as a CO Surrogate. *Angew. Chem. Int. Ed.* **2024**, *63(23)*, e202403674; https://doi.org/10.1002/anie.202403674.

[S26] Klapars, A.; Buchwald, S. L. Copper-Catalyzed Halogen Exchange in Aryl Halides: An Aromatic Finkelstein Reaction. *J. Am. Chem. Soc.* **2002**, *124(50)*, 14844-14845; https://doi.org/10.1021/ja028865v.

[S27] Zhao, W.; Wurz, R. P.; Peters, J. C.; Fu, G. C. Photoinduced, Copper-Catalyzed Decarboxylative C–N Coupling to Generate Protected Amines: An Alternative to the Curtius Rearrangement. *J. Am. Chem. Soc.* **2017**, *139(35)*, 12153-12156; https://doi.org/10.1021/jacs.7b07546.

[S28] Shields, B. C.; Yan, H.; Lim, S. S. X.; Burwell, S. C. V.; Cammarata, C. M.; Fleming, E. A.; Yousefzadeh, S. A.; Goldenshtein, V. Z.; Kahuno, E. W.; Vagadia, P. P.; Loughran, M. H.; Zhiquan, L.; McDonnell, M. E.; Scalabrino, M. L.; Thapa, M.; Hawley, T. M.; Field, G. D.; Hull, C.; Schiltz, G. E.; Glickfeld, L. L.; Reitz, A. B.; Tadross, M. R. DART.2: bidirectional synaptic pharmacology with thousandfold cellular specificity. *Nat. Methods* **2024**, *21*, 1288-1297; https://doi.org/10.1038/s41592-024-02292-9.

[S29] Waterloo, L.; Hübner, H.; Fierro, F.; Pfeiffer, T.; Brox, R.; Löber, S.; Weikert, D.; Niv, M. Y.; Gmeiner, P. Discovery of 2-Aminopyrimidines as Potent Agonists for the Bitter Taste Receptor TAS2R14. *J. Med. Chem.* **2023**, *66(5)*, 3499-3521; https://doi.org/10.1021/jacs.7b07546.

[S30] Butkevich, A. N.; Lukinavičius, G.; D'Este, E.; Hell, S. W. Cell-Permeant Large Stokes Shift Dyes for Transfection-Free Multicolor Nanoscopy. *J. Am. Chem. Soc.* **2017**, *139(36)*, 12378-12381; https://doi.org/10.1021/jacs.7b06412.

[S31] Singh, V.; Wang, S.; Kool, E.T. Genetically encoded multispectral labeling of proteins with polyfluorophores on a DNA backbone. *J. Am. Chem. Soc.* **2013**, *135(16)*, 6184-6191; https://doi.org/10.1021/ja4004393.
