# Supplementary material for: Synthesis of Benzo[b]thiophene 1,1-Dioxides via Pd-Catalyzed Sulfinylation of Aryl Triflates and Their Use as Large Stokes Shift Fluorophores for Multicolor Live-Cell Imaging with Self-Labeling Tags
Source: JACS Au. 2026 Apr 14;6(4):2396–406. doi: 10.1021/jacsau.6c00024 (PMC13126164; doi:10.1021/jacsau.6c00024)
Supplement: Supplementary file 2 [file au6c00024_si_002.pdf]

Supplementary information

Synthesis of benzo[*b*]thiophene 1,1-dioxides via Pd-catalyzed sulfinylation of aryl triflates and their use as large Stokes shift fluorophores for multicolor live-cell imaging with self-labeling tags

Alexey N. Butkevich[a†*], Mariano L. Bossi[a], Jasmine Hubrich[a] and Stefan W. Hell[a,b*]

[a] Department of Optical Nanoscopy, Max Planck Institute for Medical Research, Jahnstraße 29, 69120 Heidelberg, Germany.

[b] Department of NanoBiophotonics, Max Planck Institute for Multidisciplinary Sciences, Am Faßberg 11, 37077 Göttingen, Germany.

[†] Present address: Facility for Synthetic Chemistry, Max Planck Institute for Multidisciplinary Sciences, Am Faßberg 11, 37077 Göttingen, Germany.

* Corresponding authors' e-mails: alexey.butkevich@mr.mpg.de, stefan.hell@mpinat.mpg.de.

NMR spectra

1H (400.15 MHz, CDCl3)

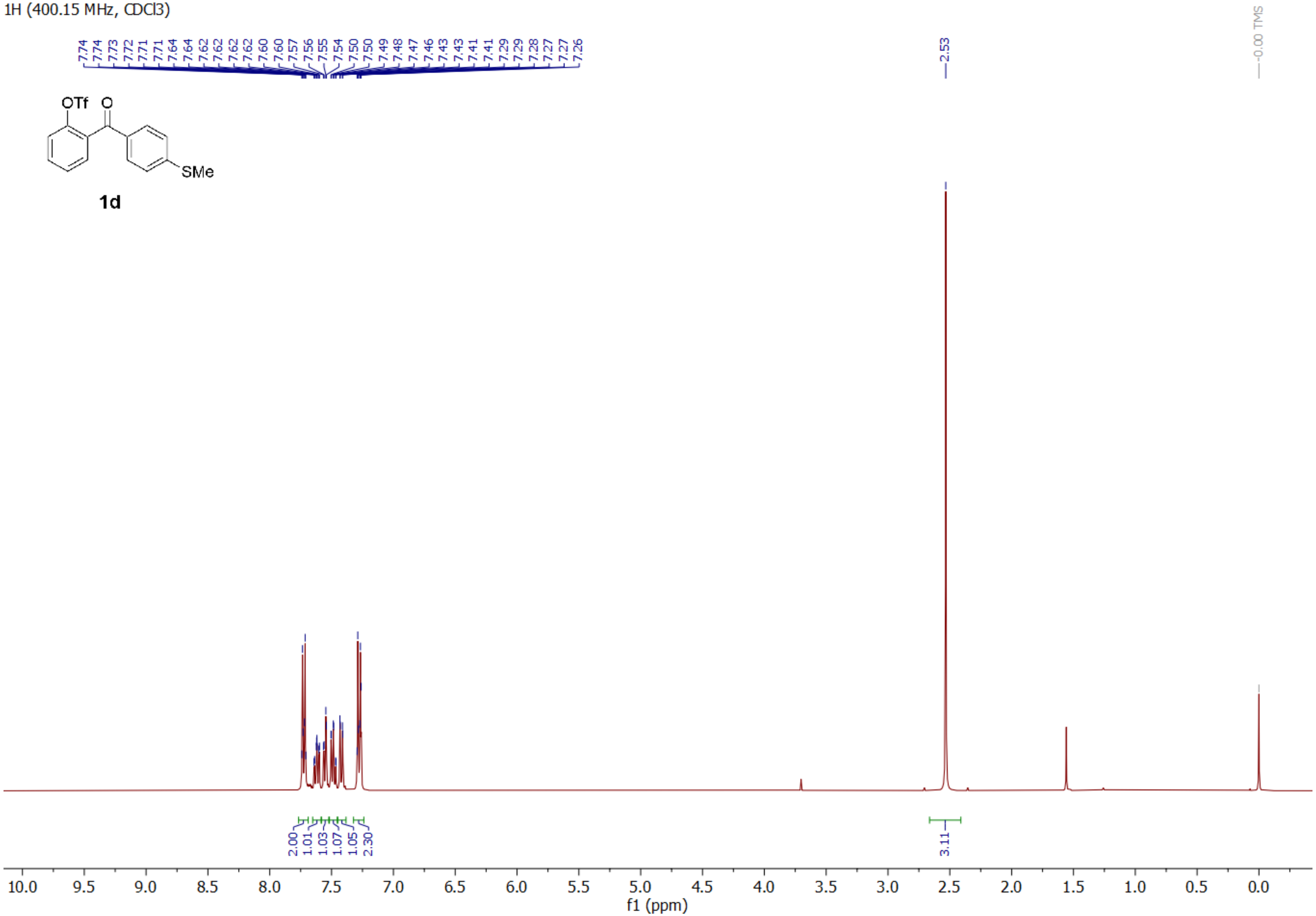

1d

13C (100.63 MHz, CDCl3)

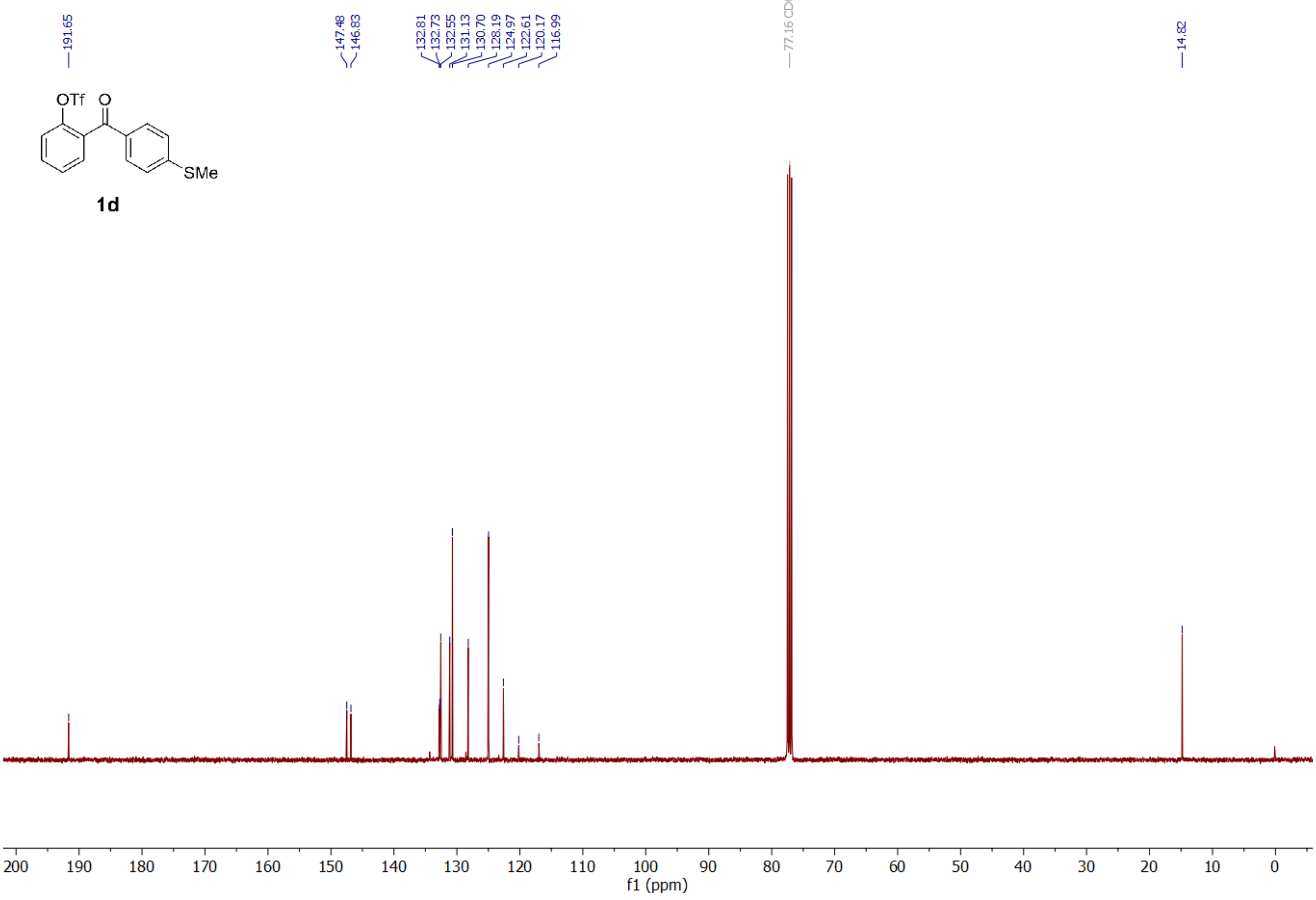

1d

19F (376.48 MHz, CDCl3)

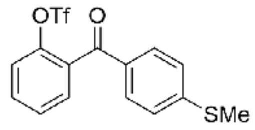

1d

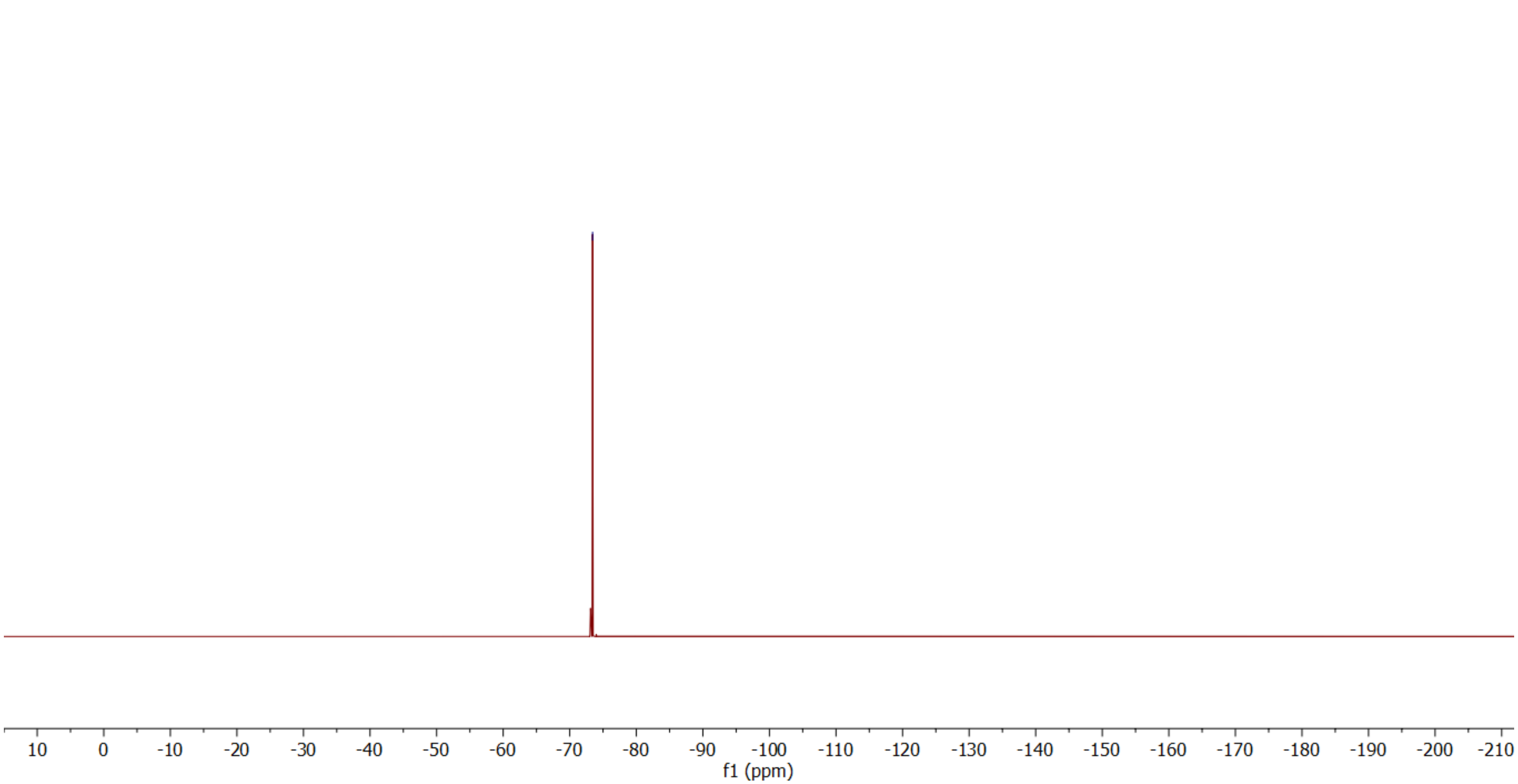

1H (400.15 MHz, CDCl3)

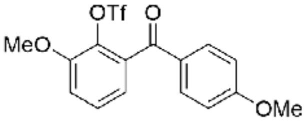

1g

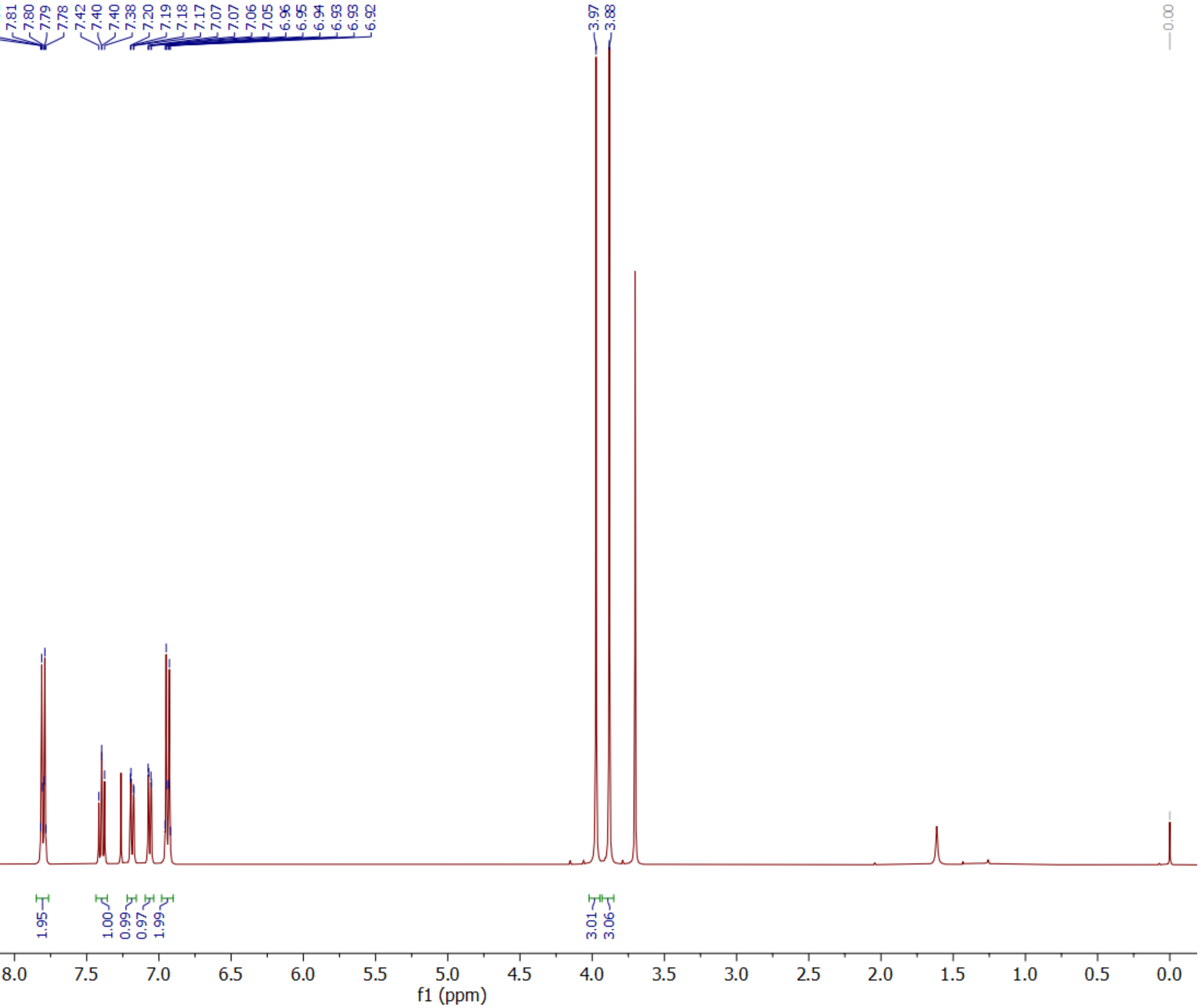

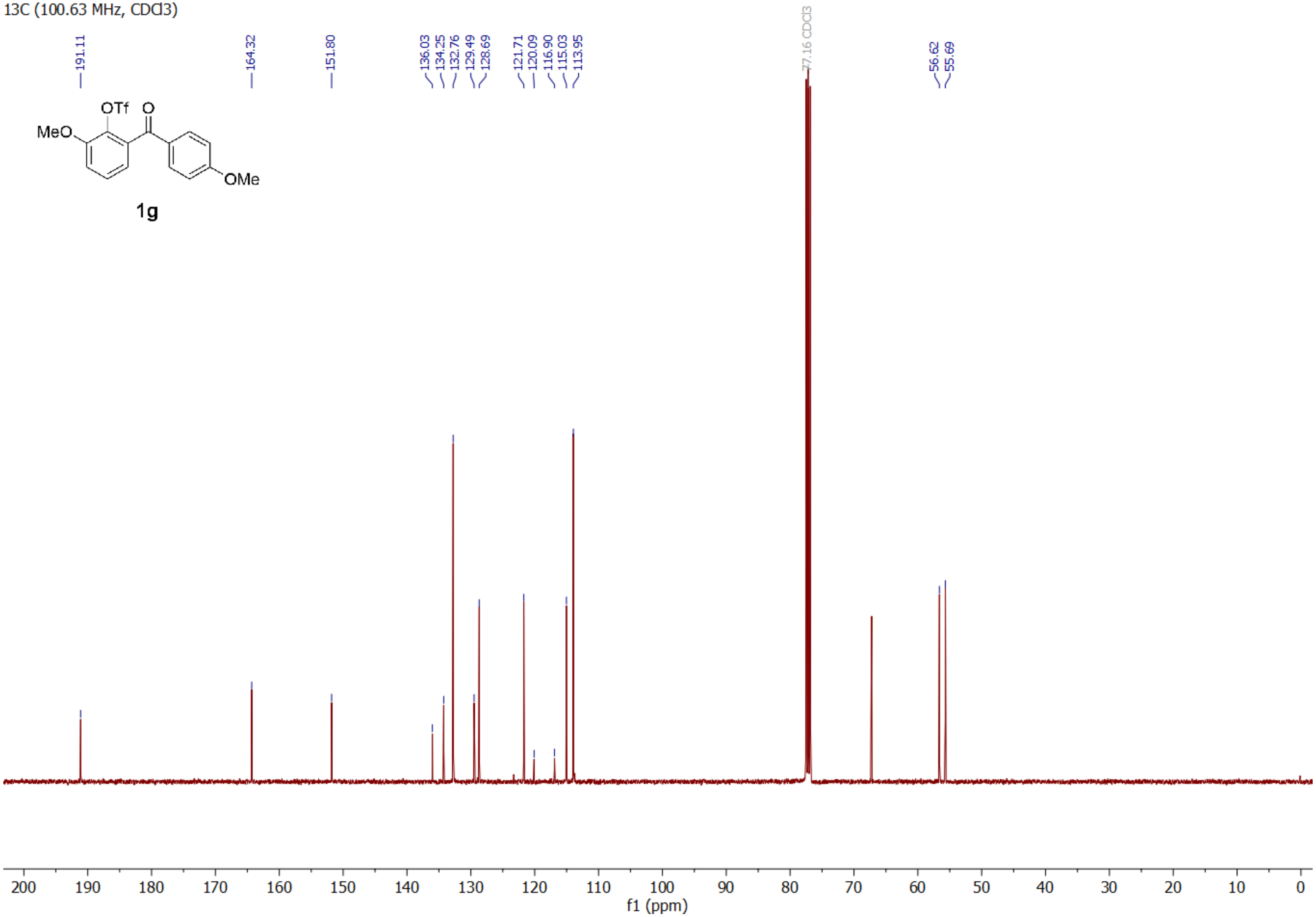
13C (100.63 MHz, CDCl3)
191.11
164.32
151.80
136.03
134.25
132.76
129.49
128.69
121.71
120.09
116.90
115.03
113.95
77.16 CDCl3
56.62
55.69
OTf
O
MeO
OMe
1g
200
190
180
170
160
150
140
130
120
110
100
90
80
70
60
50
40
30
20
10
0
f1 (ppm)

19F (376.48 MHz, CDCl3)

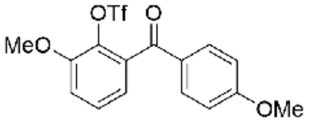

1g

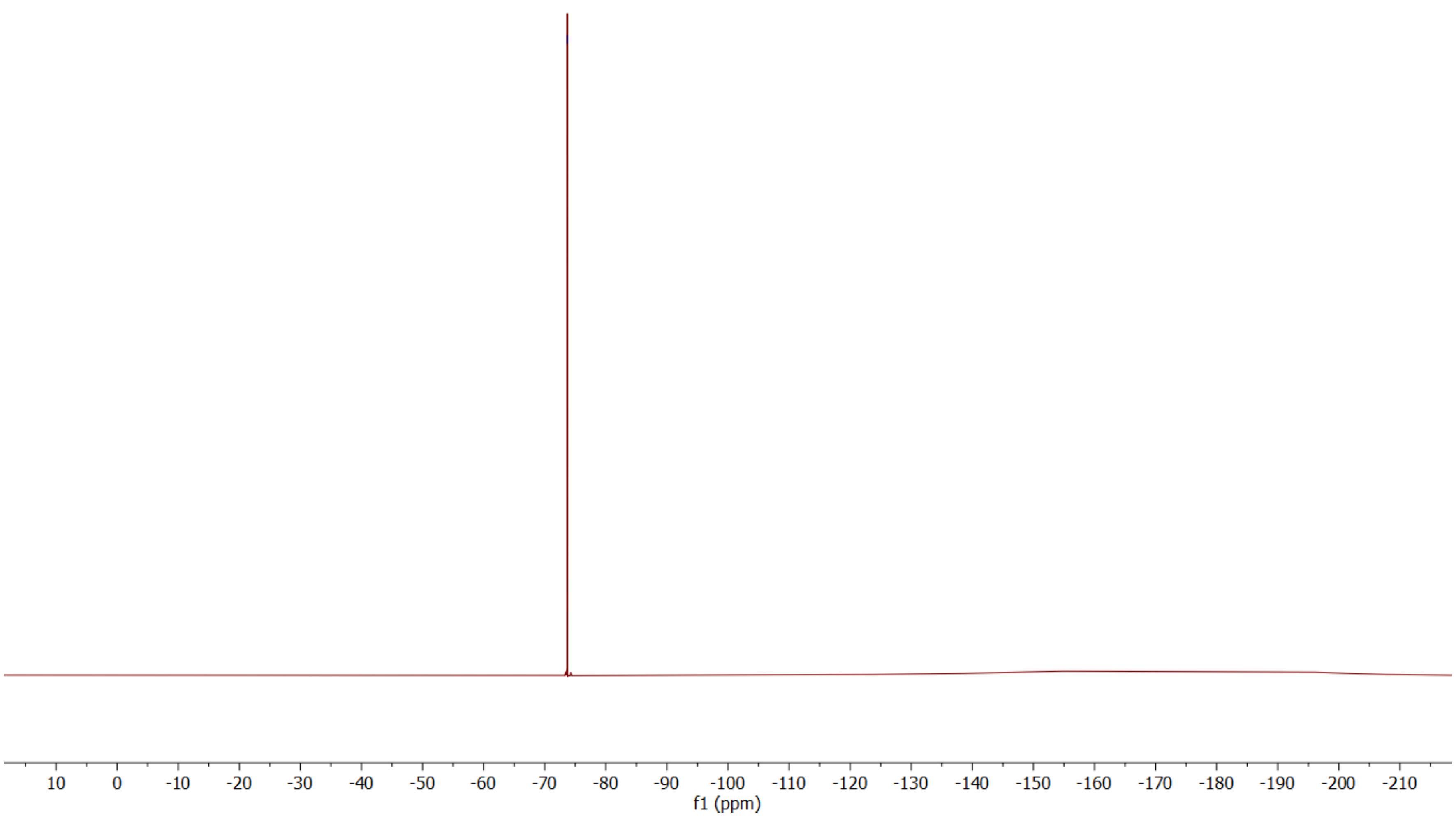

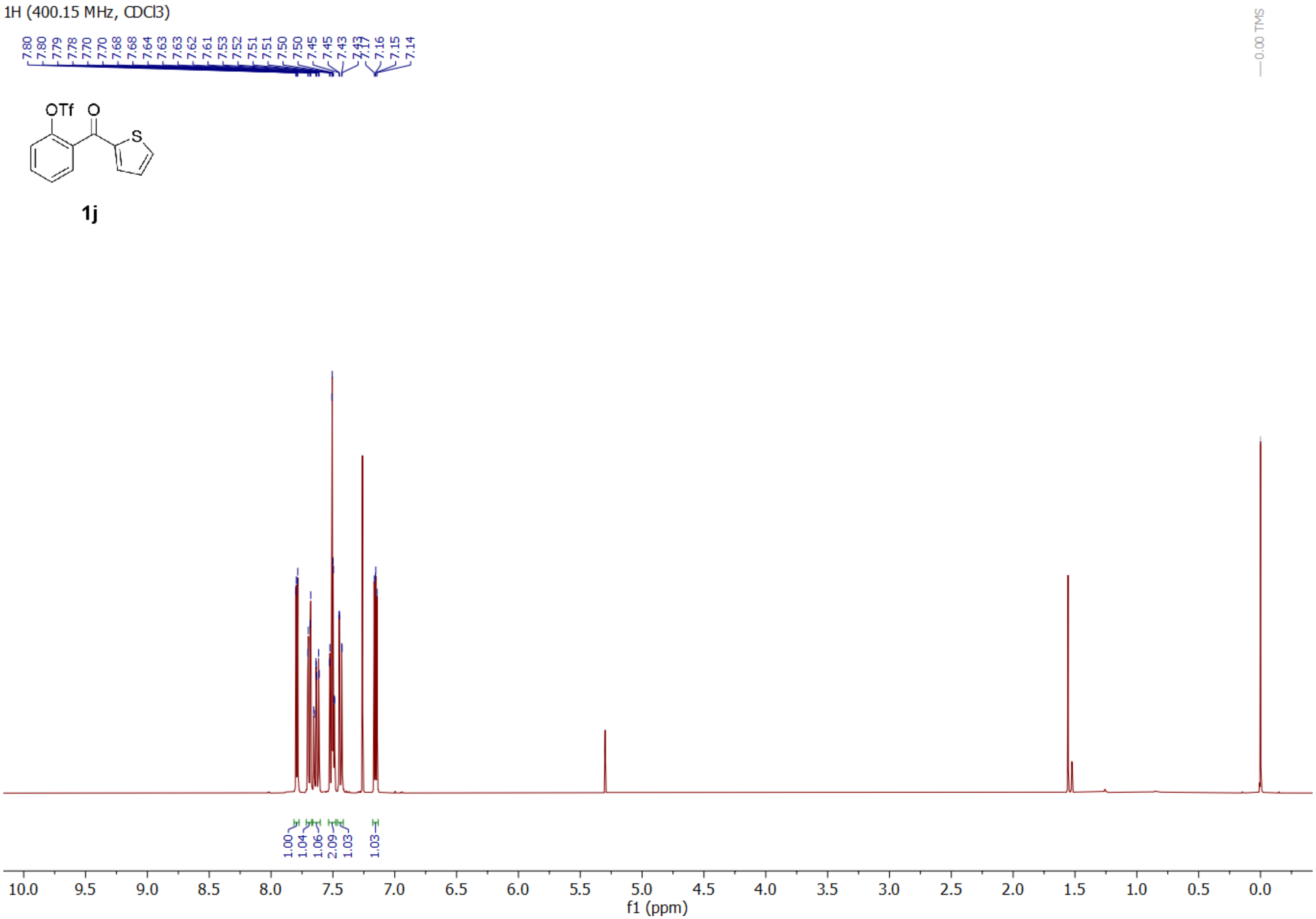
1H (400.15 MHz, CDCl3)
7.80 7.80 7.79 7.78 7.70 7.70 7.68 7.68 7.64 7.63 7.63 7.62 7.61 7.53 7.52 7.51 7.51 7.50 7.50 7.45 7.45 7.43 7.43 7.17 7.16 7.15 7.14
0.00 TMS
OTf
O
S
1.00
1.04
1.06
2.09
1.03
1.03
f1 (ppm)

1j

13C (100.63 MHz, CDCl3)

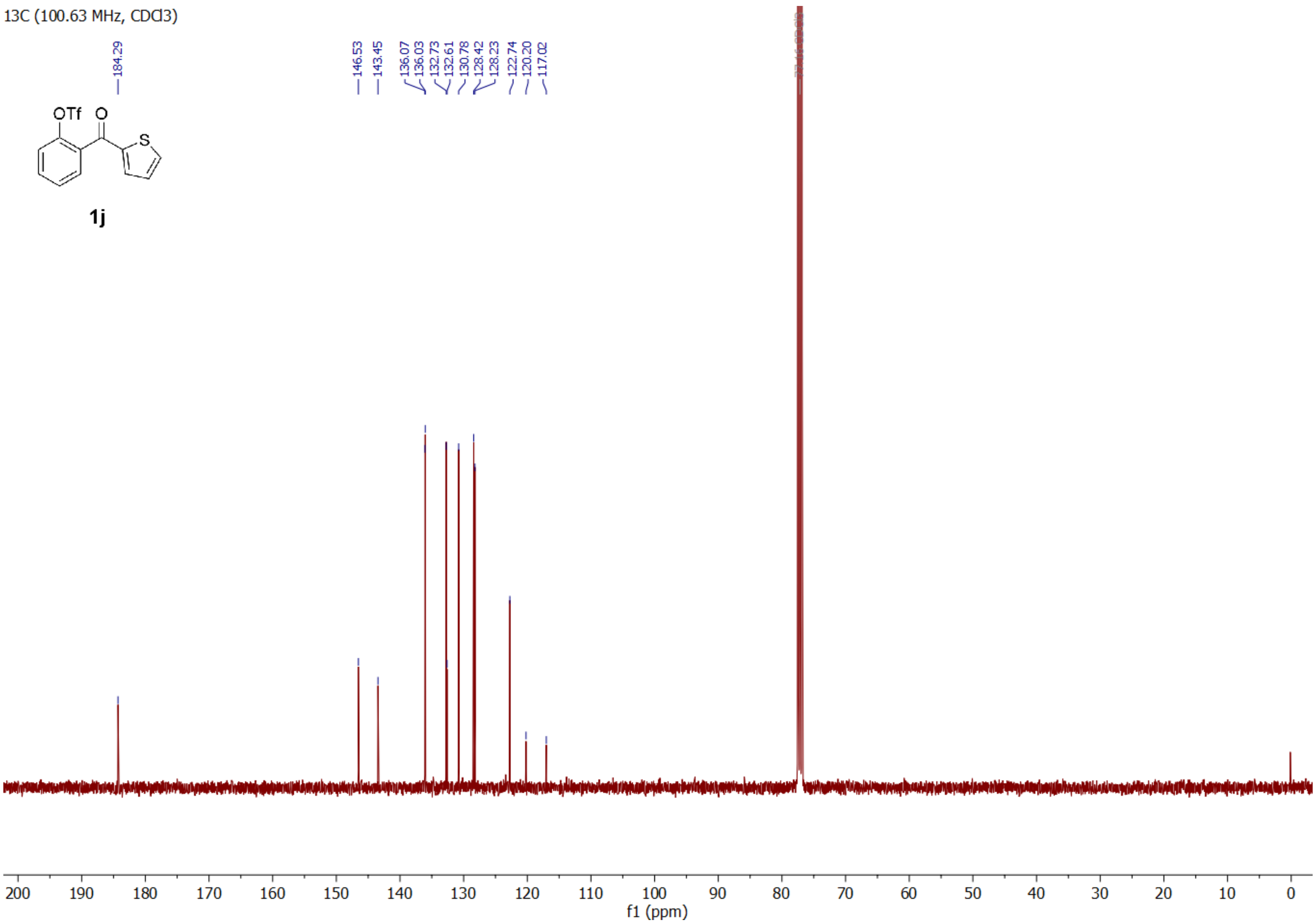

19F (376.48 MHz, CDCl3)

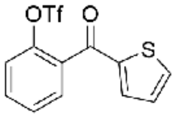

1j

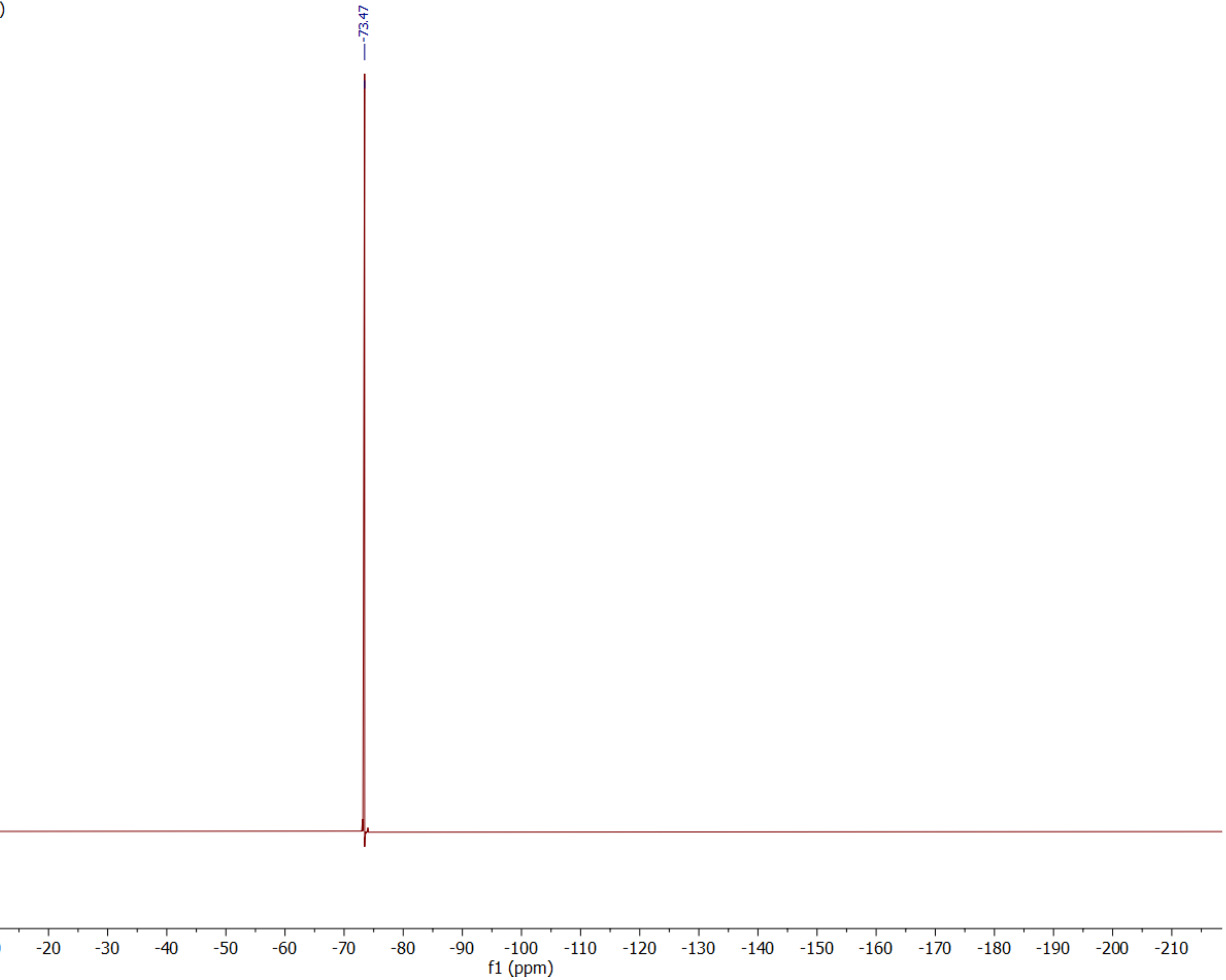

1H (400.15 MHz, CDCl3)

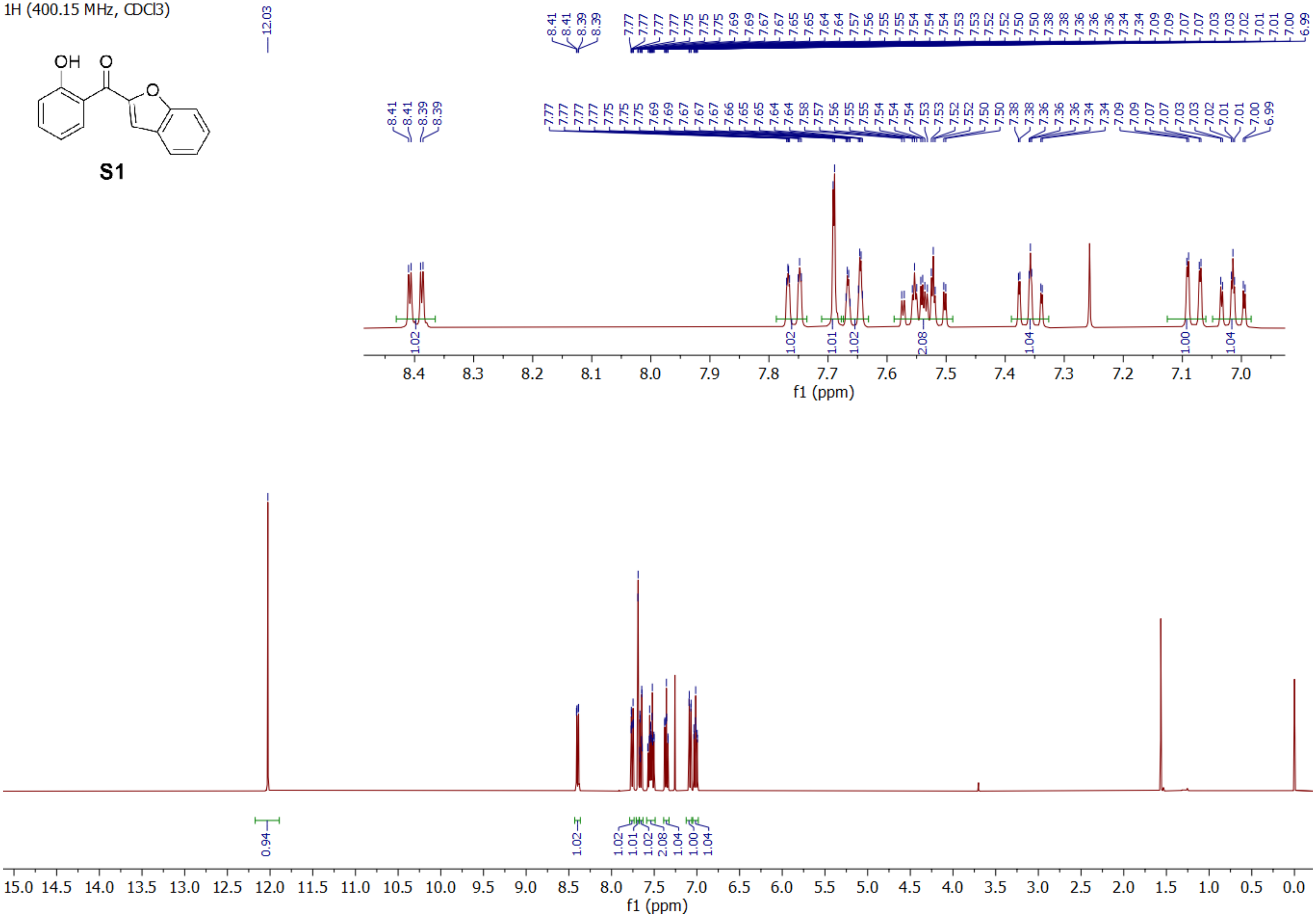

S1

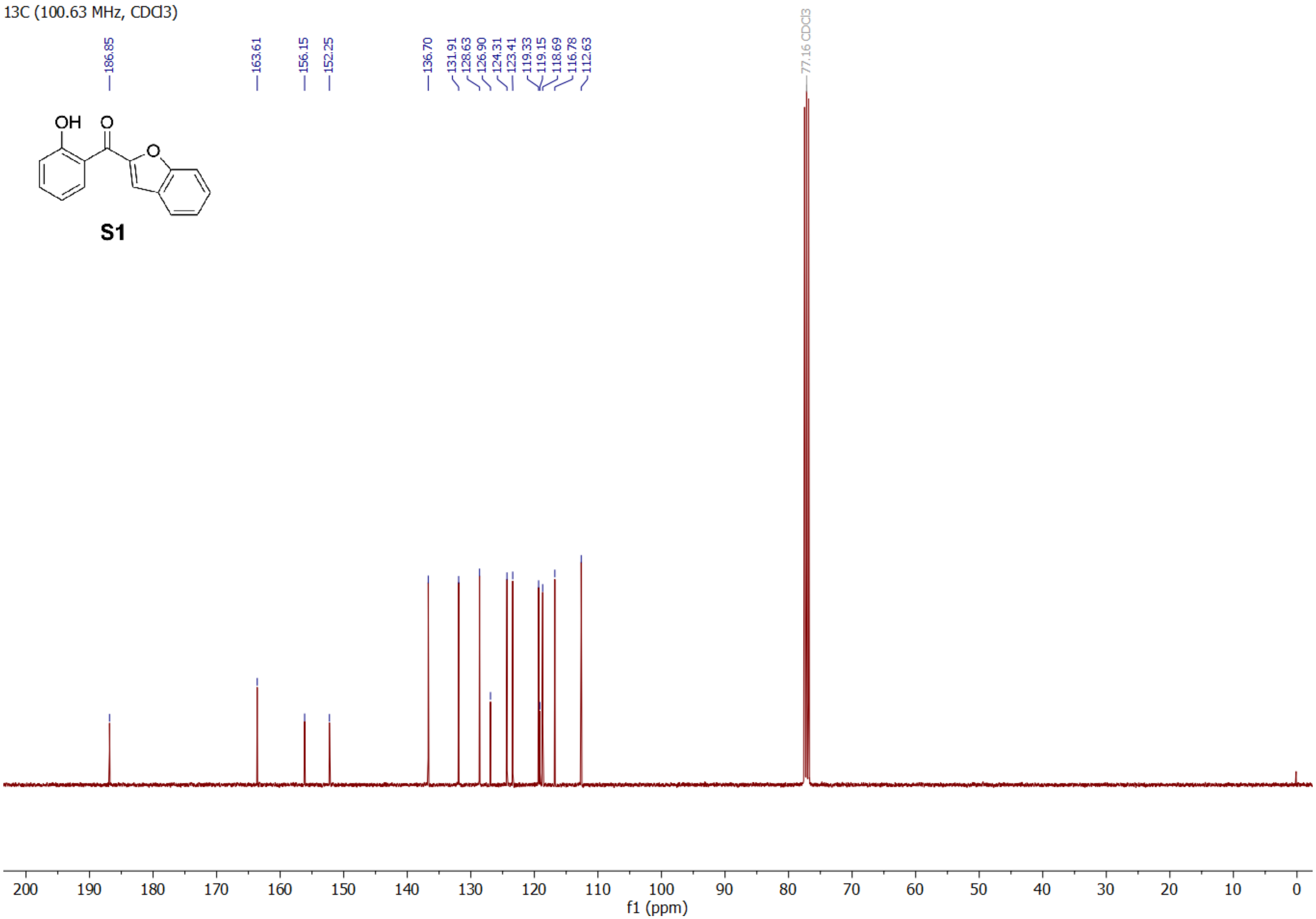
13C (100.63 MHz, CDCl3)
186.85
163.61
156.15
152.25
136.70
131.91
128.63
126.90
124.31
123.41
119.33
119.15
118.69
116.78
112.63
77.16 CDCl3
OH
O
O
S1
200 190 180 170 160 150 140 130 120 110 100 90 80 70 60 50 40 30 20 10 0
f1 (ppm)

1H (400.15 MHz, CDCl3)

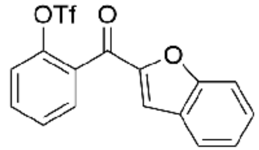

1k

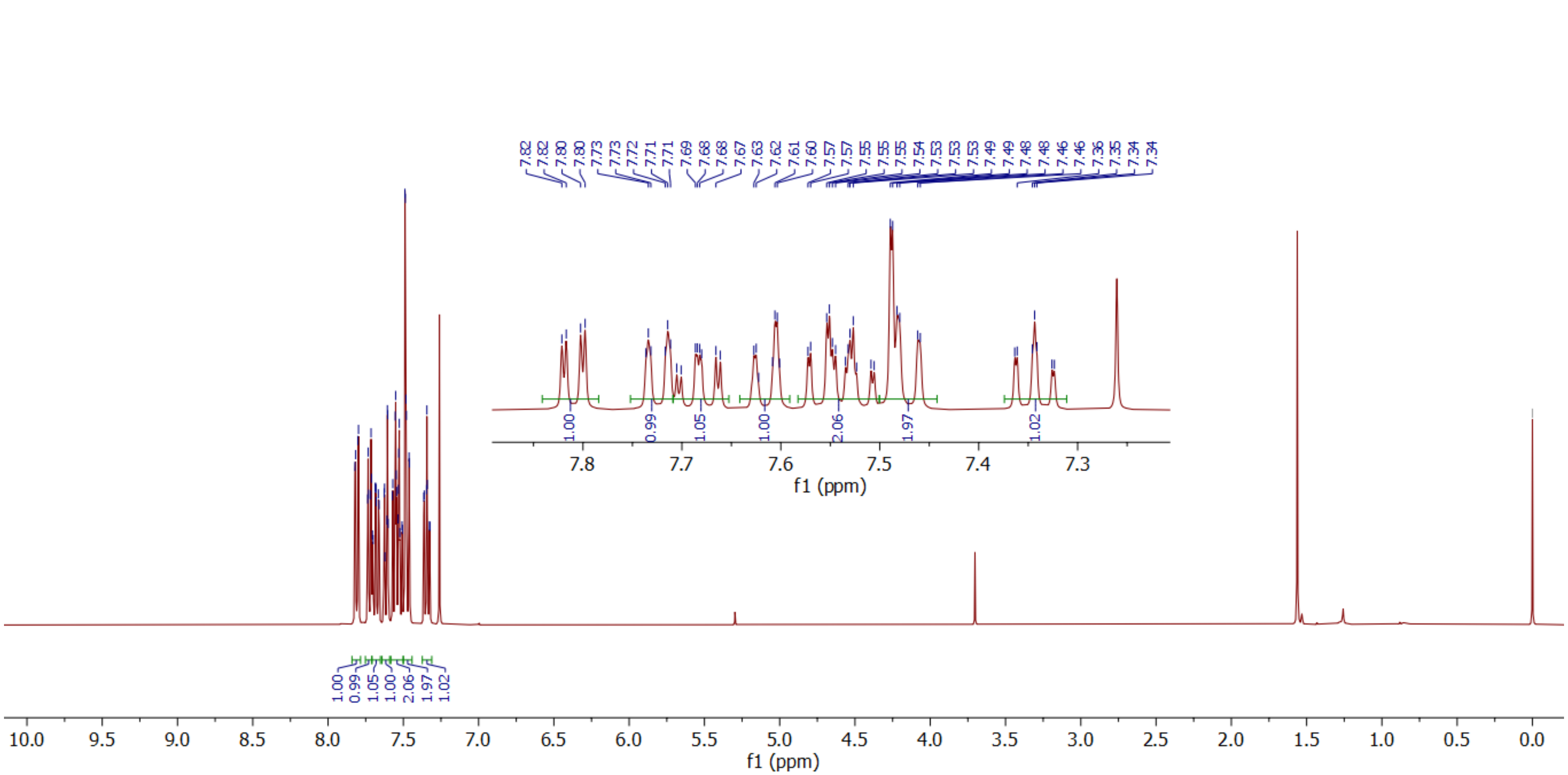

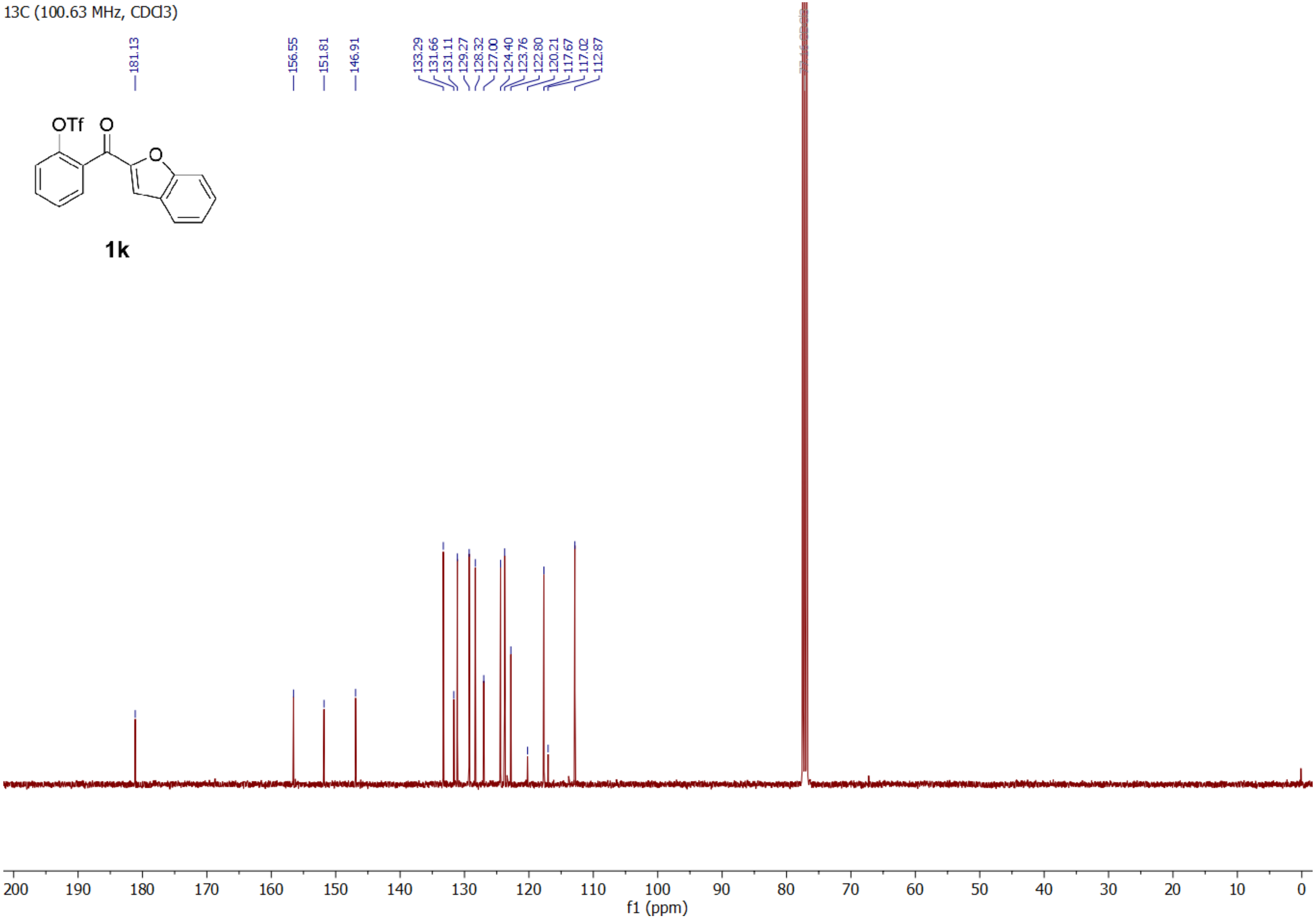

13C (100.63 MHz, CDCl3)
181.13
156.55
151.81
146.91
133.29
131.66
131.11
129.27
128.32
127.00
124.40
123.76
122.80
120.21
117.67
117.02
112.87
OTf
O
O
1k
200
190
180
170
160
150
140
130
120
110
100
90
80
70
60
50
40
30
20
10
0
f1 (ppm)

19F (376.48 MHz, CDCl3)

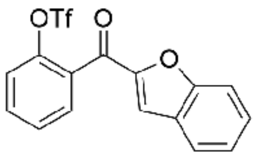

1k

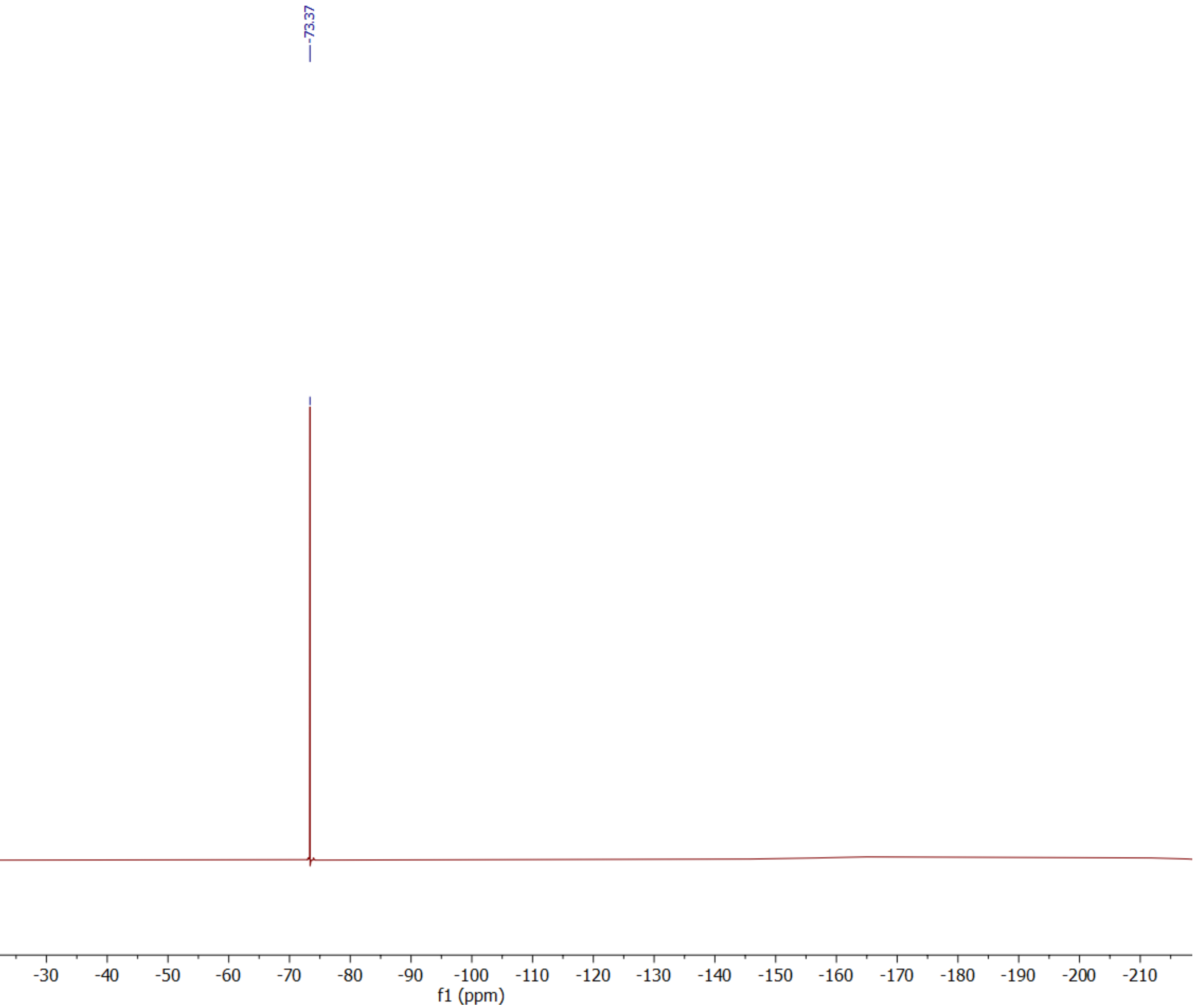

1H (400.15 MHz, CDCl3)

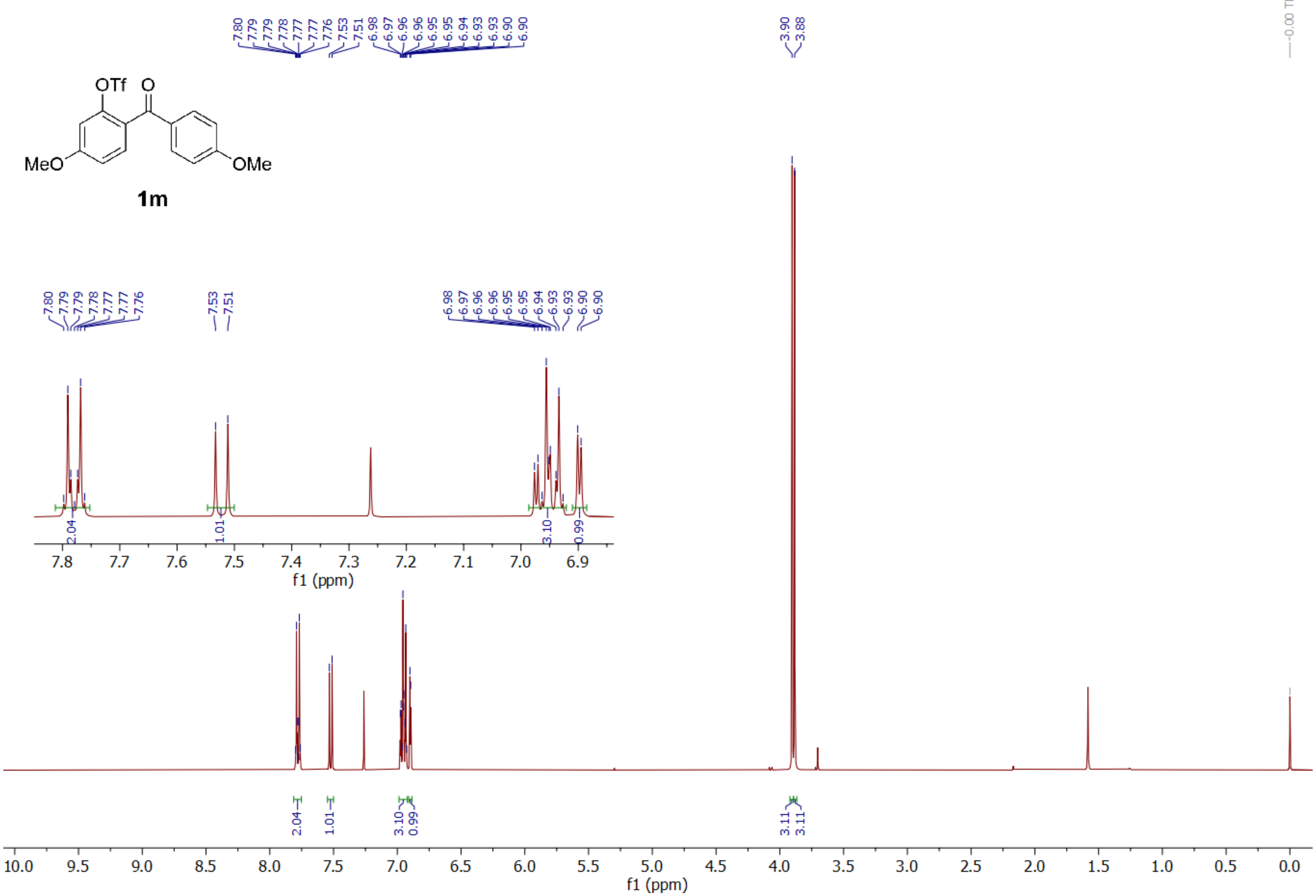

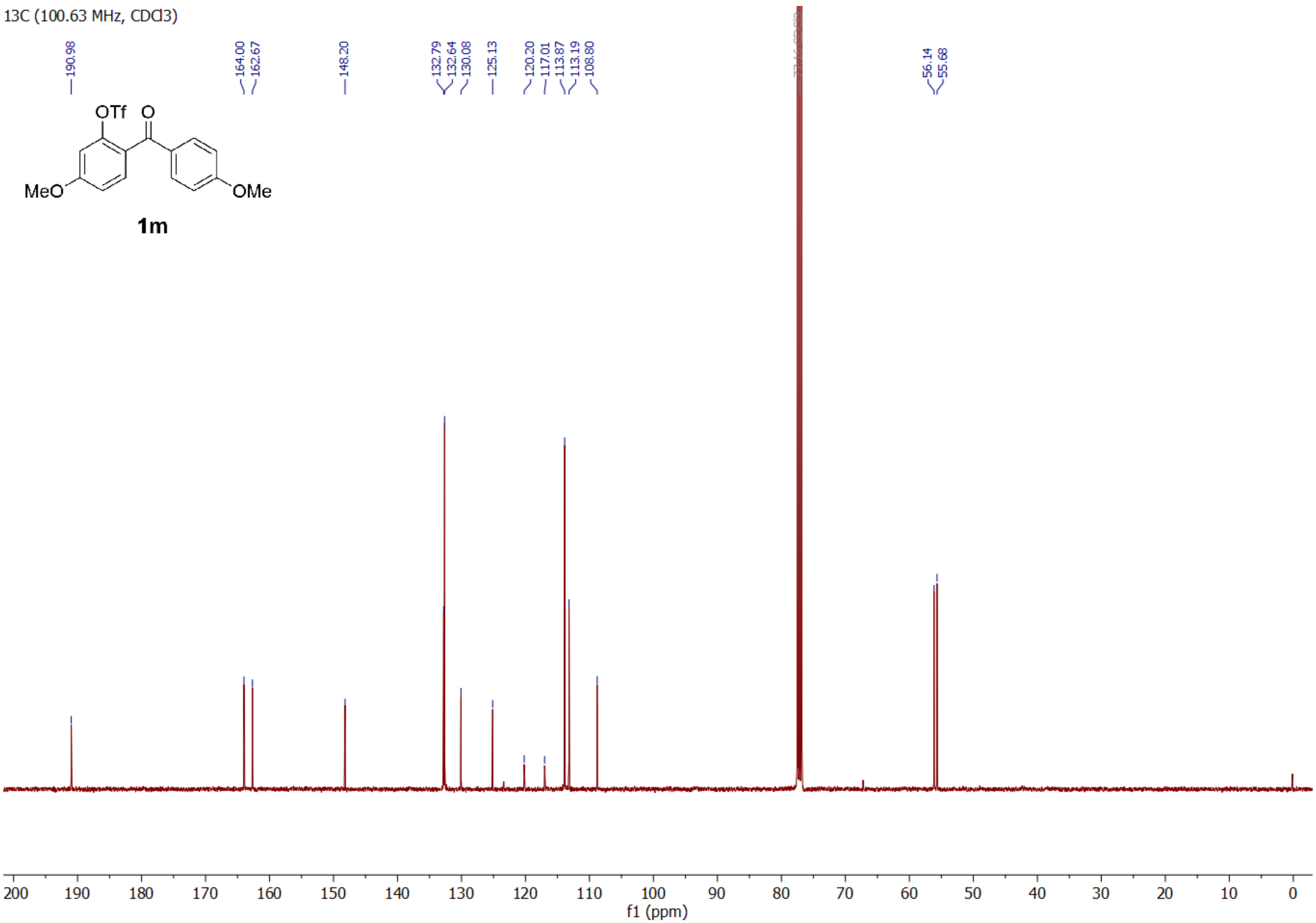
13C (100.63 MHz, CDCl3)
190.98
164.00
162.67
148.20
132.79
132.64
130.08
125.13
120.20
117.01
113.87
113.19
108.80
56.14
55.68
OTf
O
MeO
OMe
1m
f1 (ppm)

19F (376.48 MHz, CDCl3)

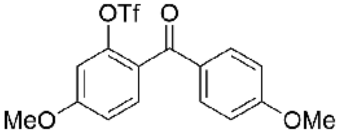

1m

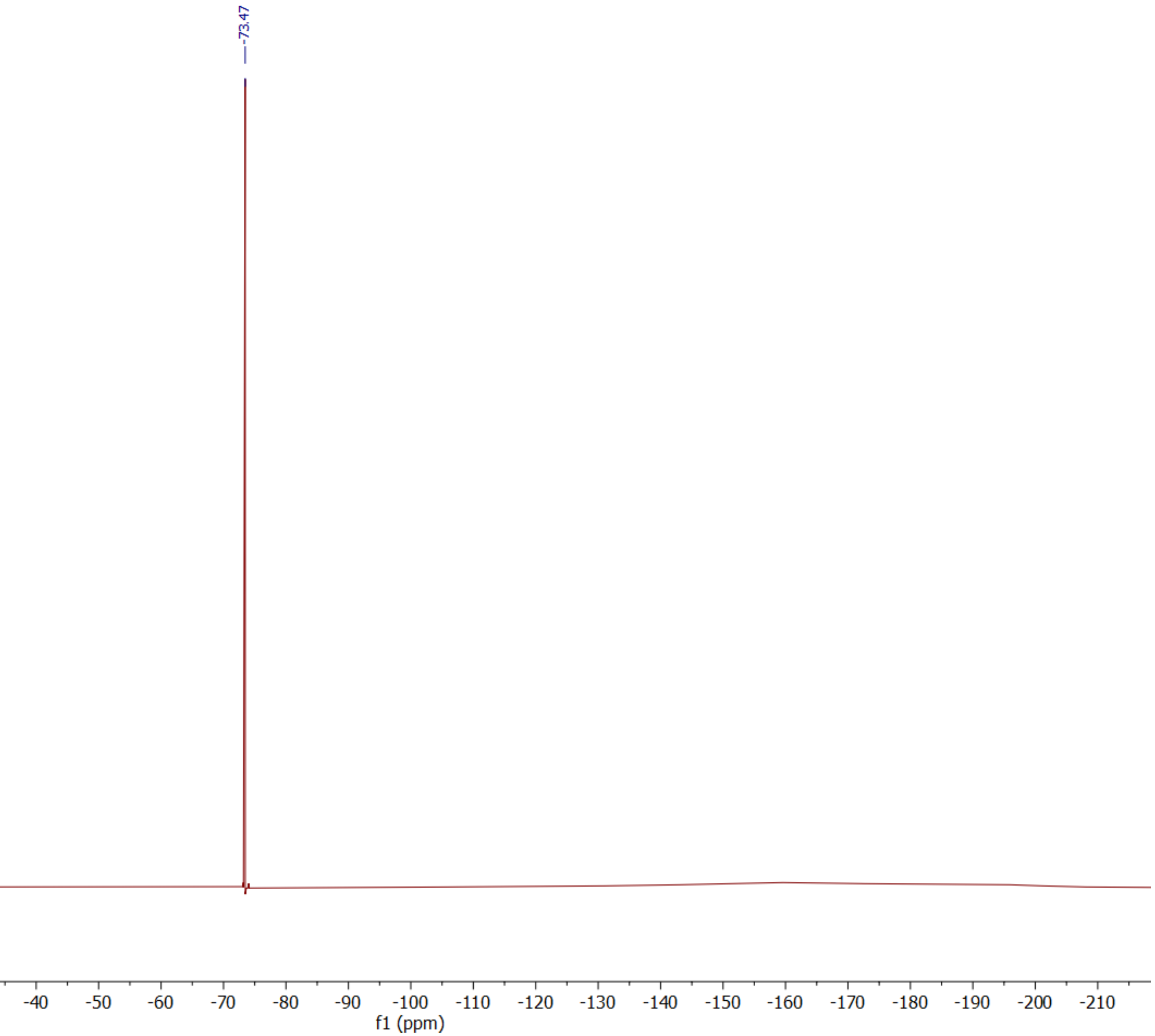

1H (400.15 MHz, CDCl3)

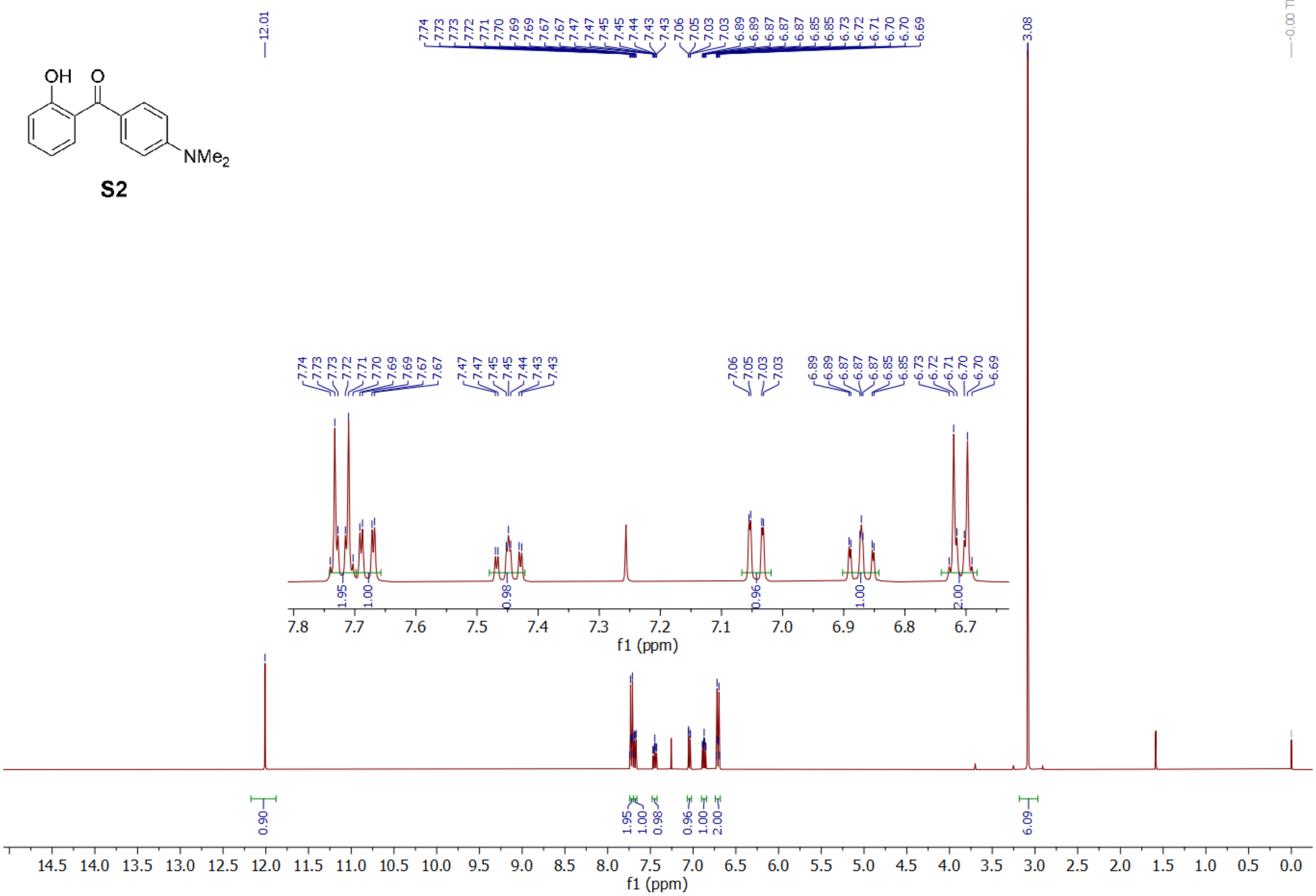

S2

13C (100.63 MHz, CDCl3)

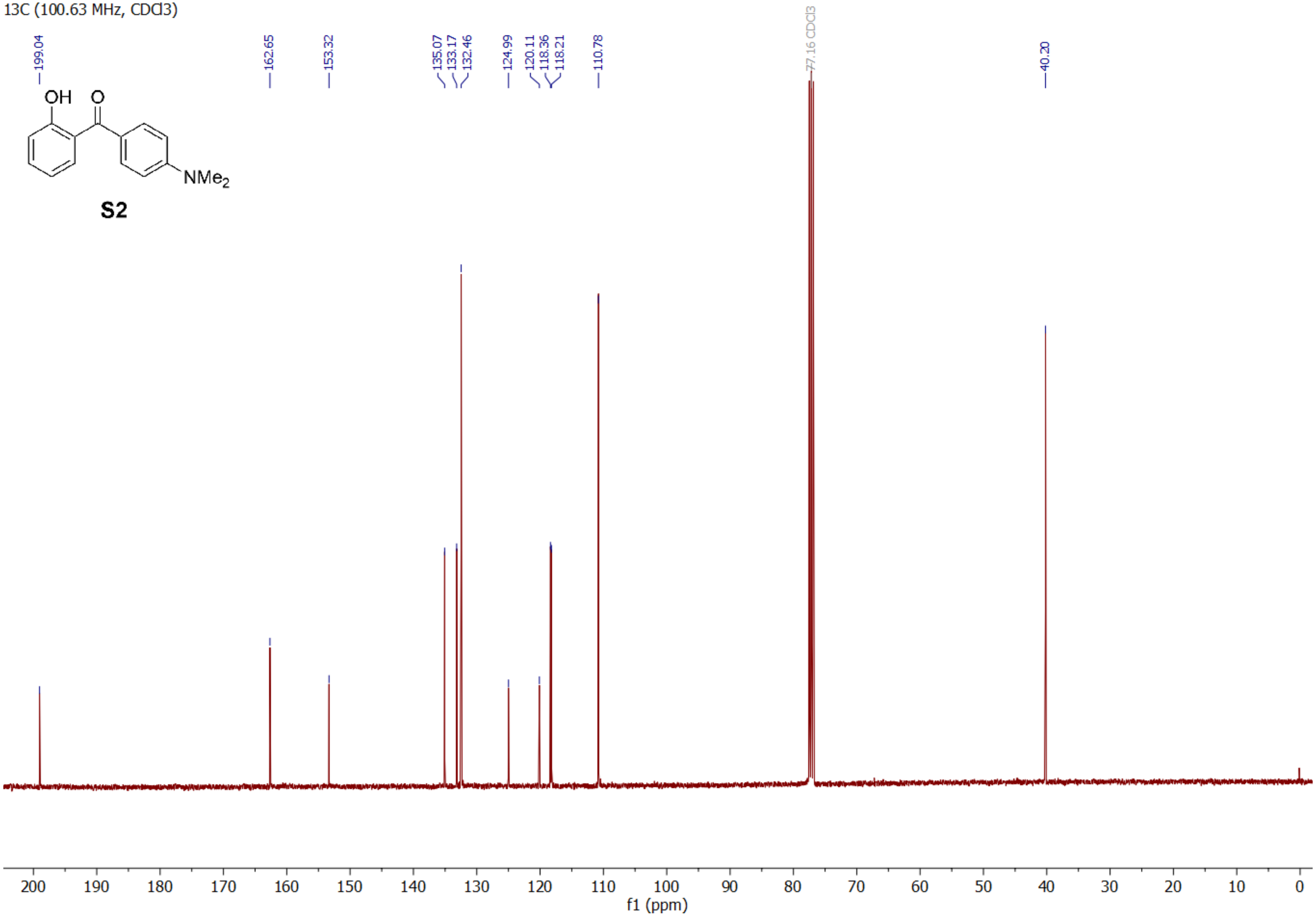

1H (400.15 MHz, CDCl3)

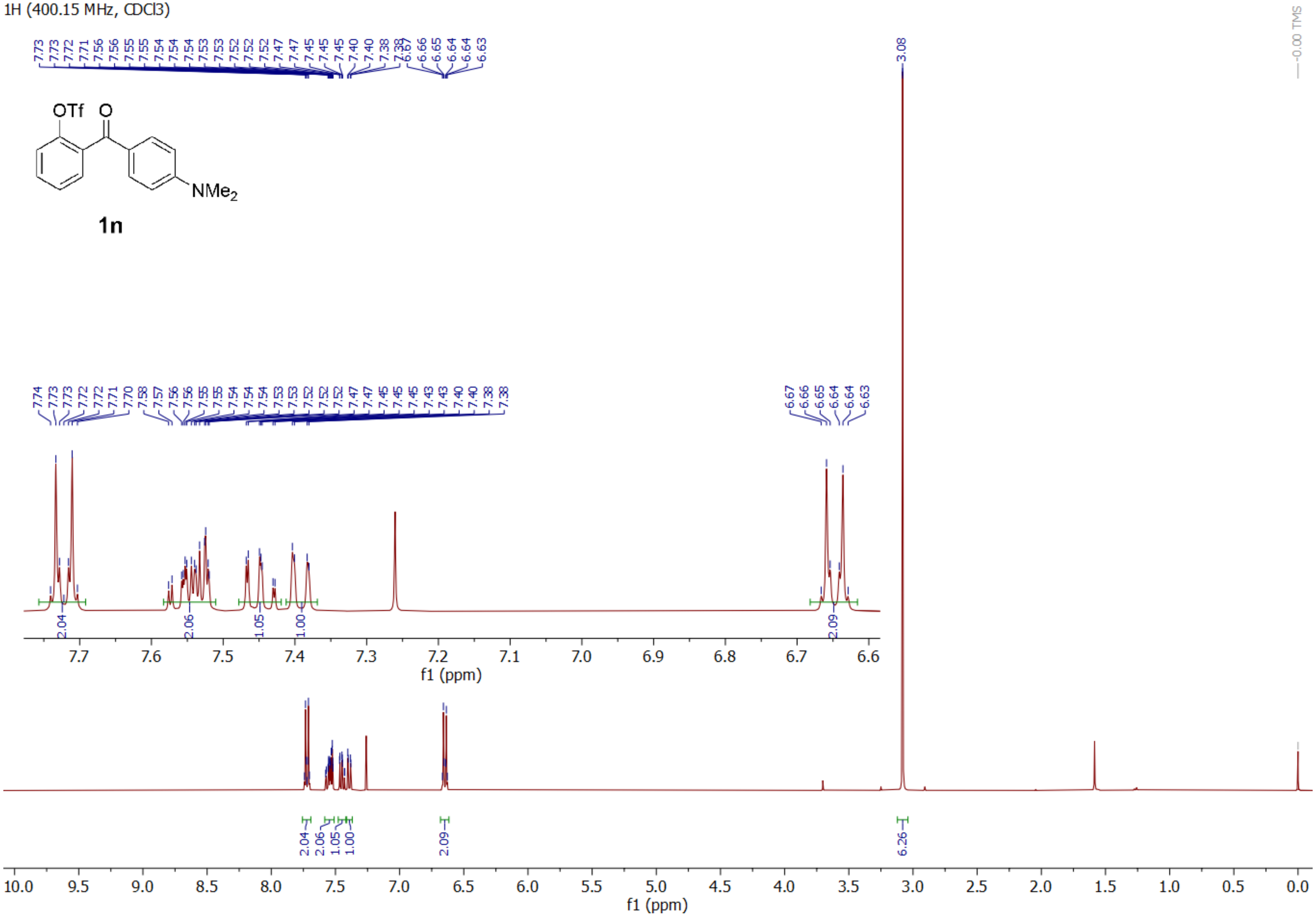

13C (100.63 MHz, CDCl3)

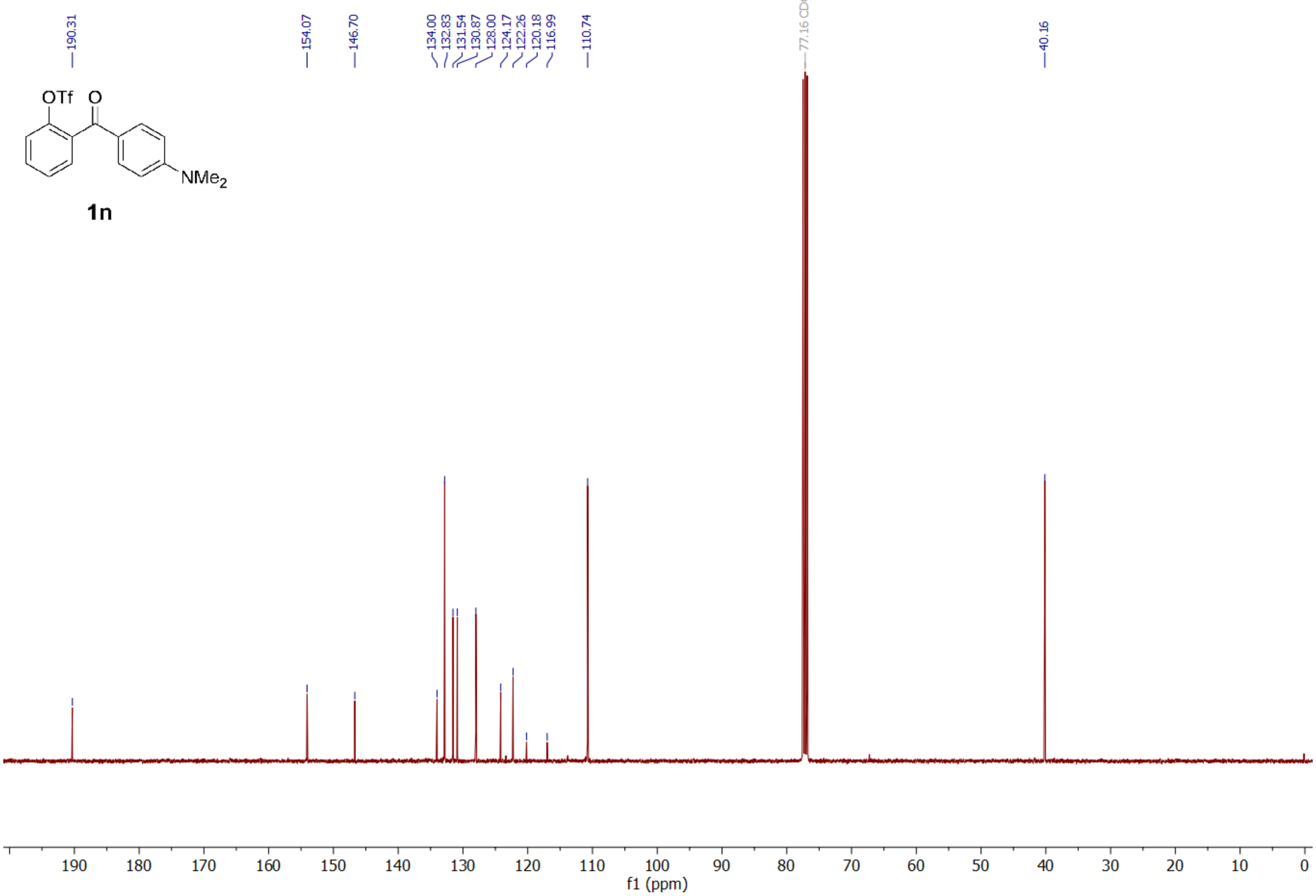

19F (376.48 MHz, CDCl3)

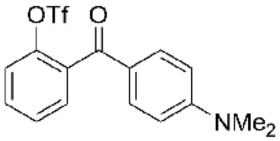

1n

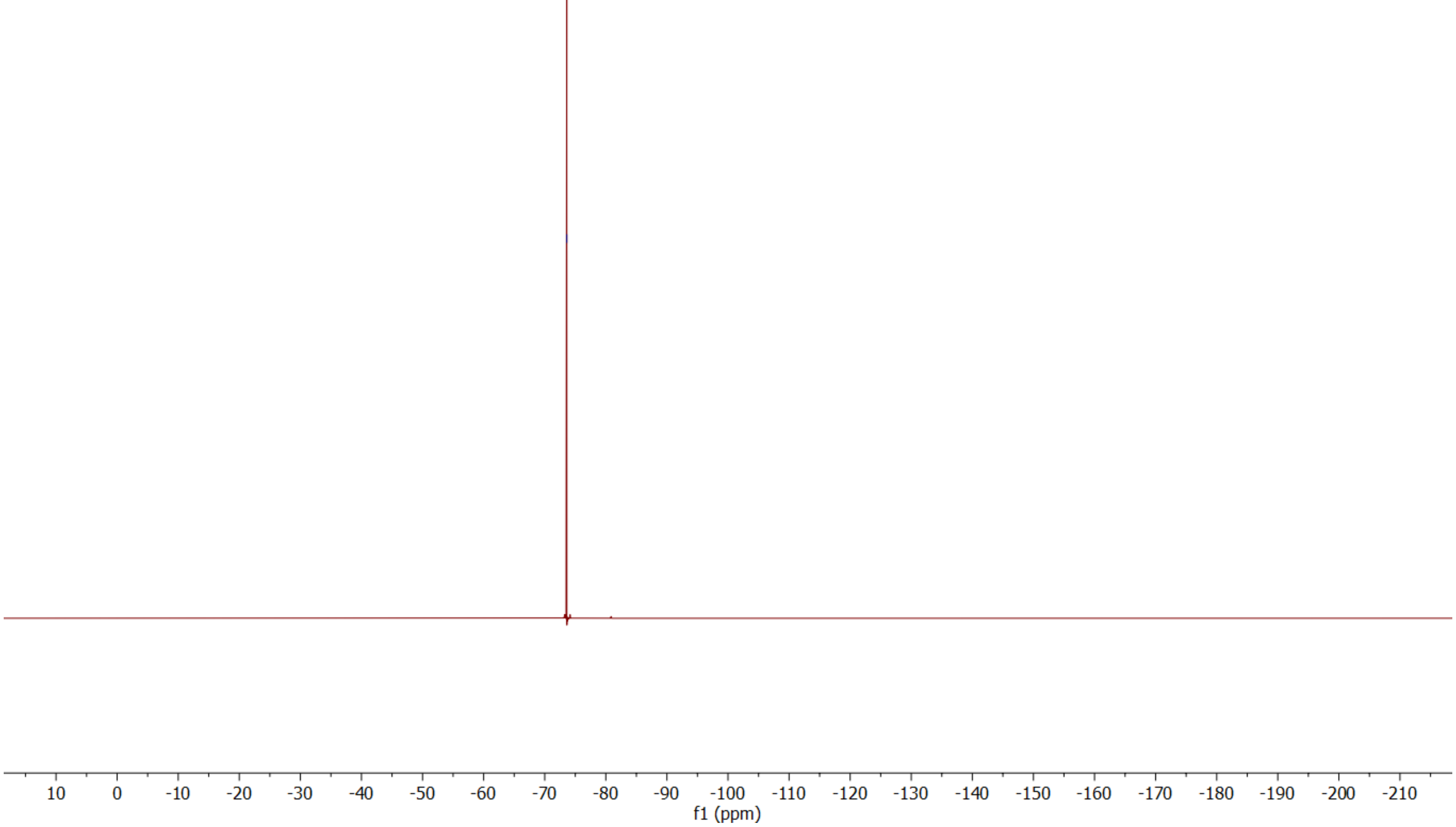

1H (400.15 MHz, CDCl3)

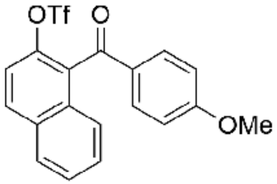

1p

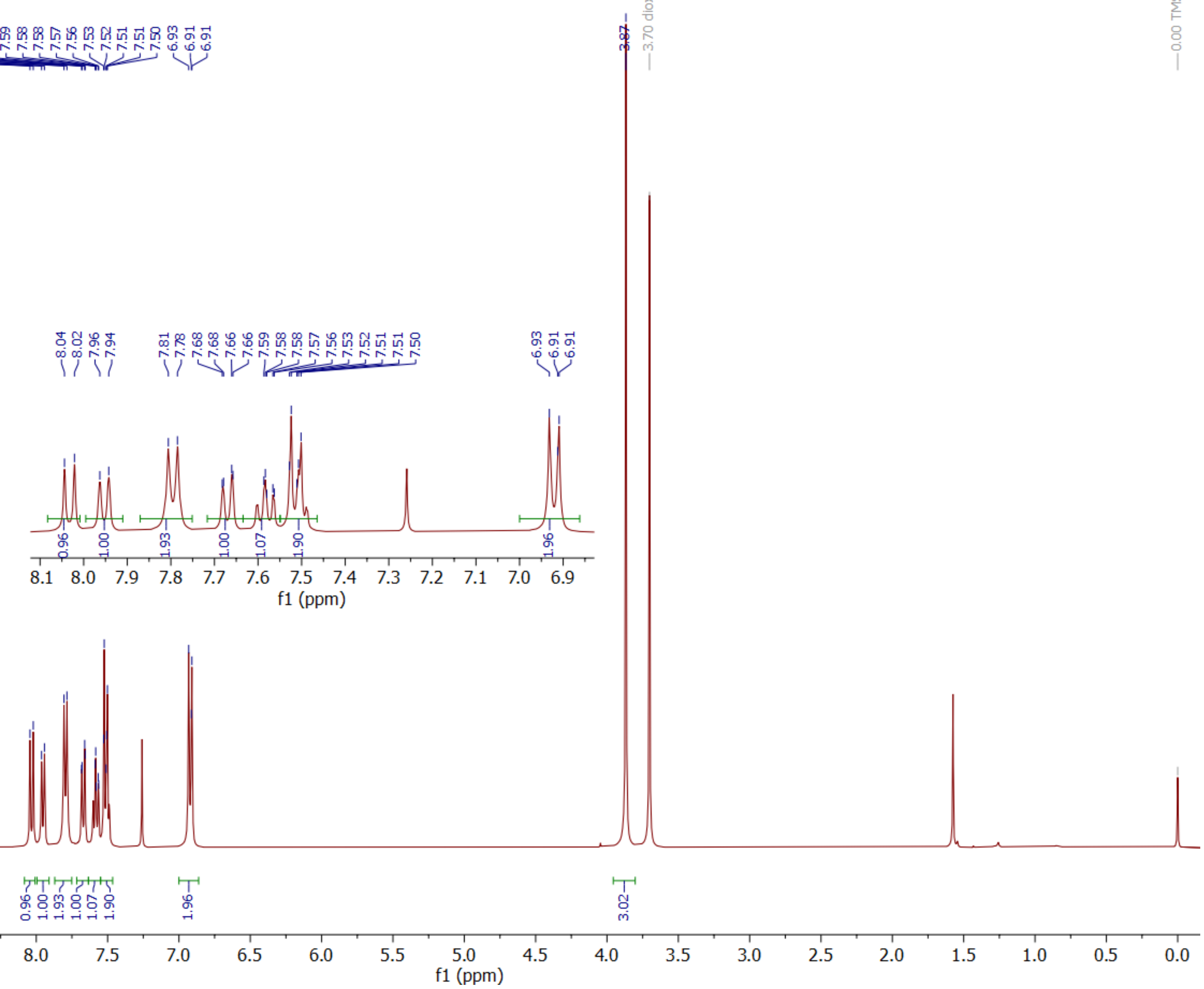

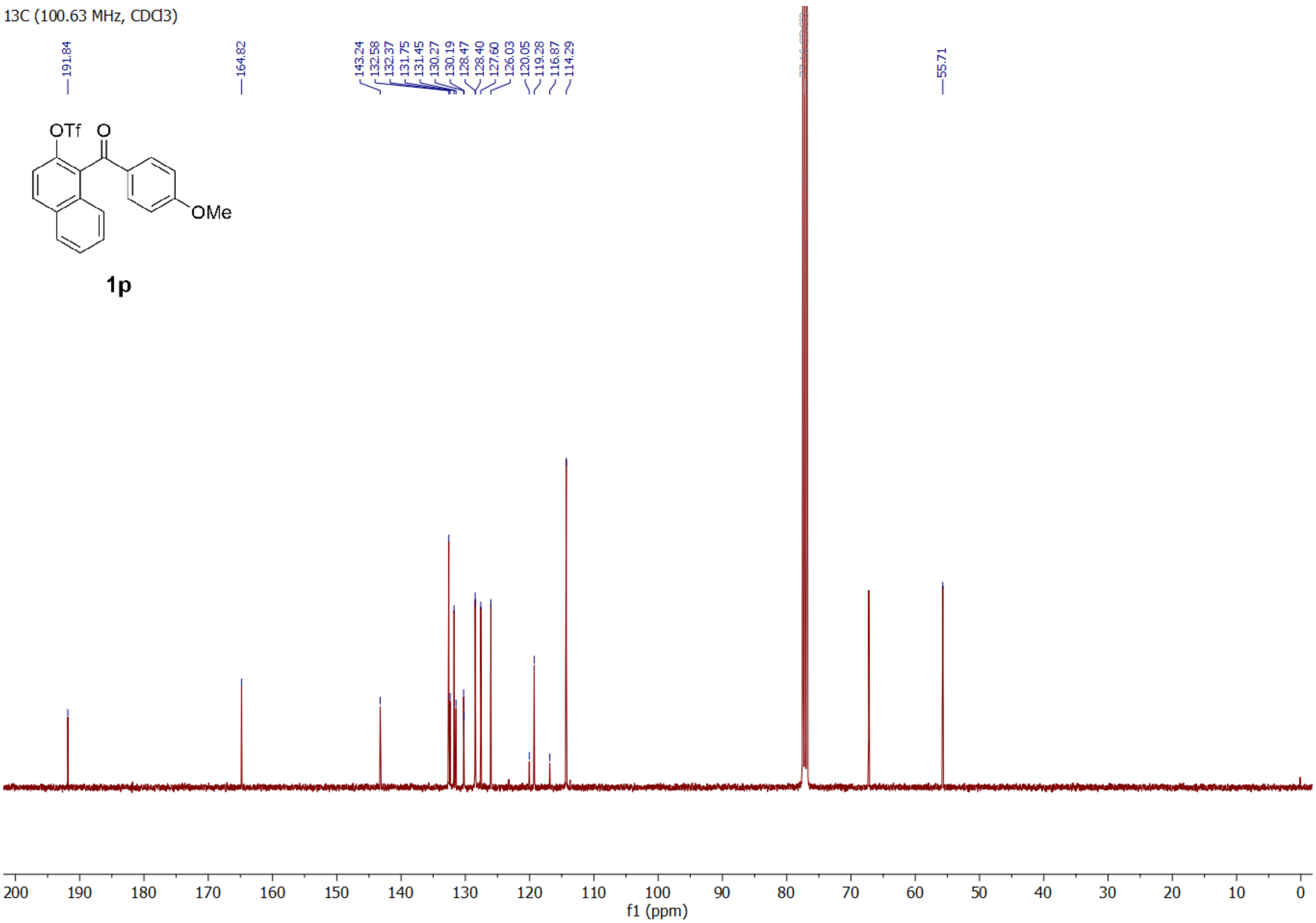
13C (100.63 MHz, CDCl3)
191.84
164.82
143.24
132.58
132.37
131.75
131.45
130.27
130.19
128.47
128.40
127.60
126.03
120.05
119.28
116.87
114.29
55.71
OTf
O
OMe
1p
200
190
180
170
160
150
140
130
120
110
100
90
80
70
60
50
40
30
20
10
0
f1 (ppm)

19F (376.48 MHz, CDCl3)

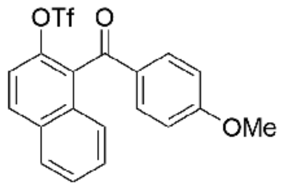

1p

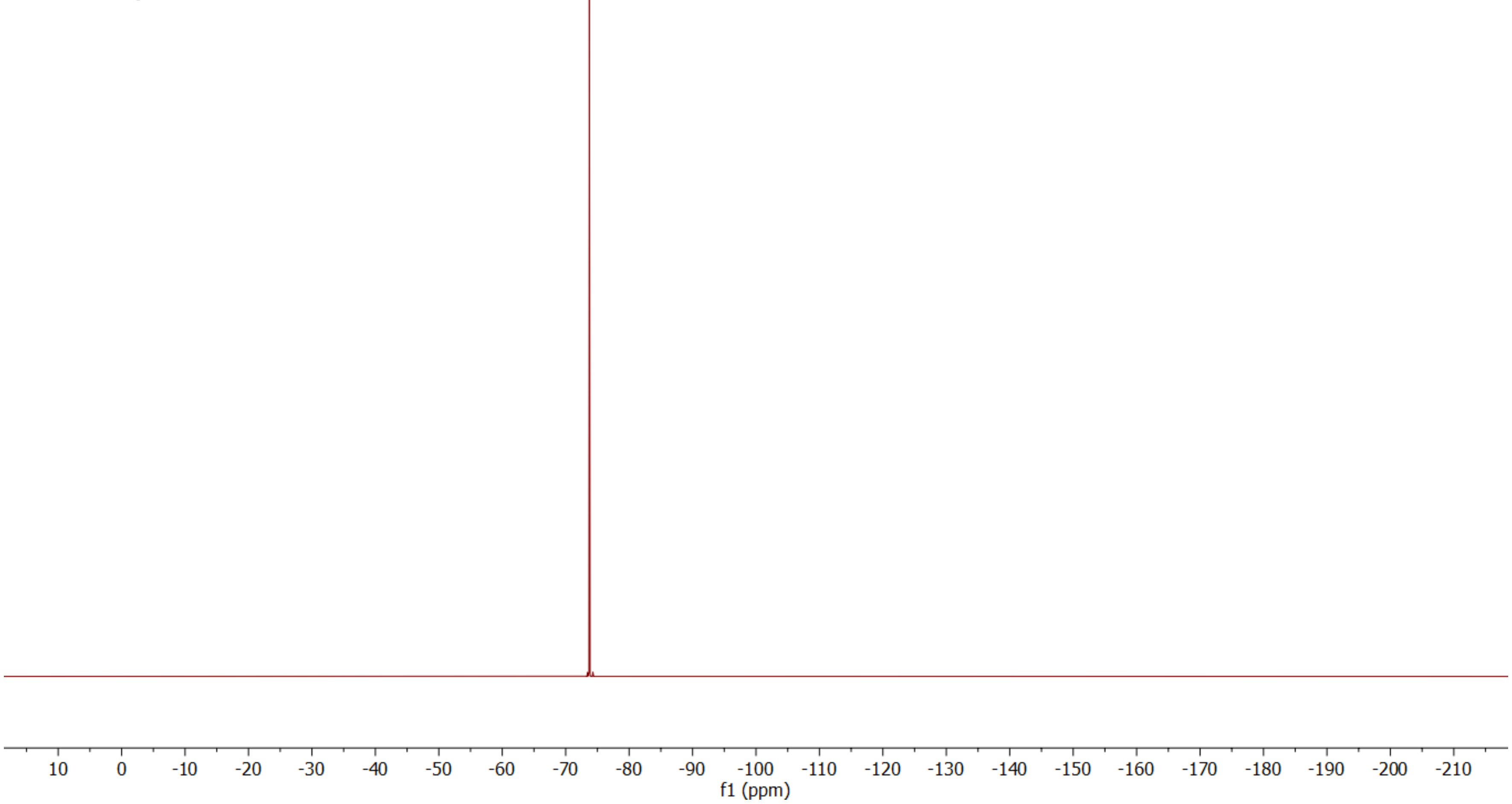

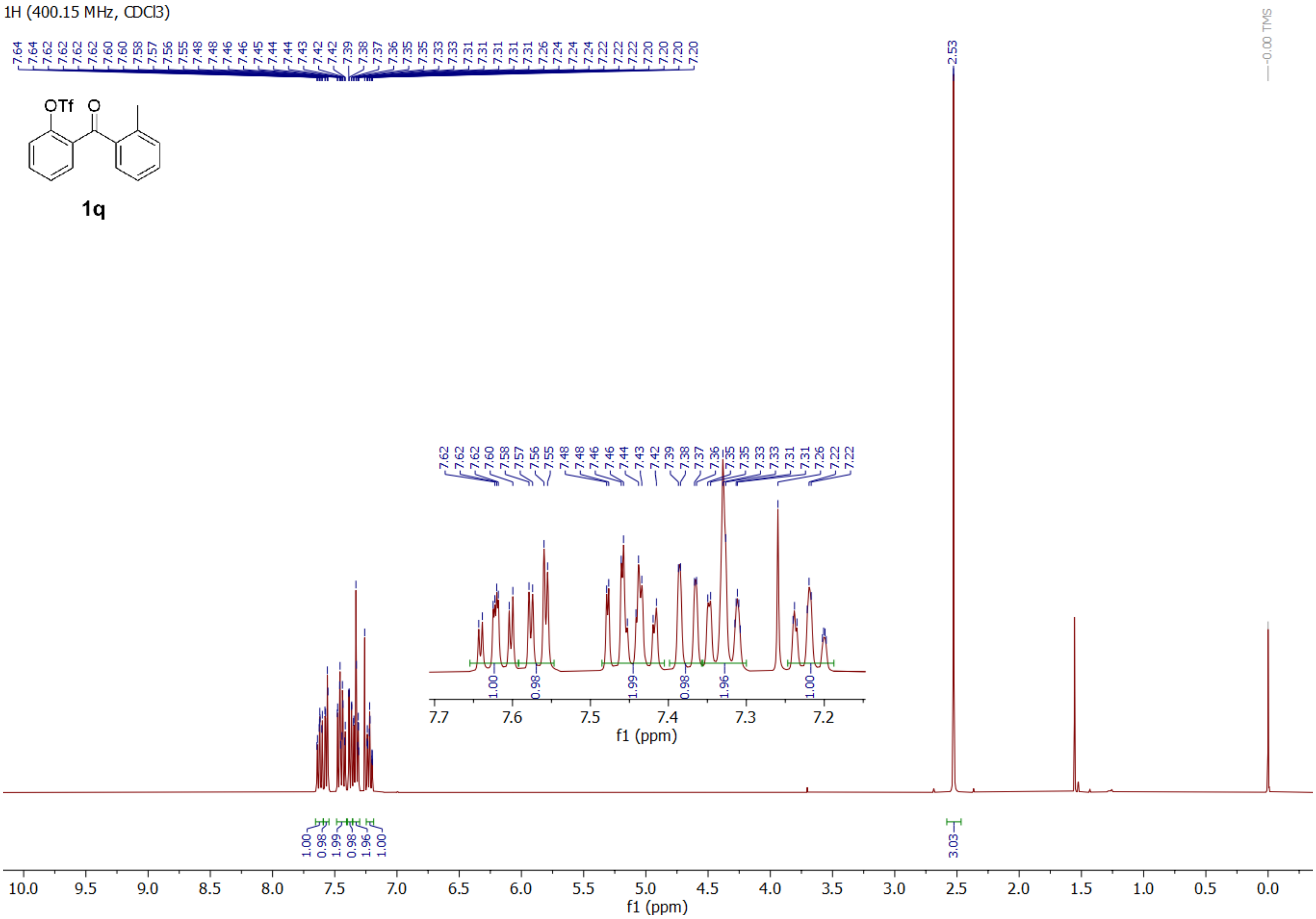
1H (400.15 MHz, CDCl3)
7.64 7.64 7.62 7.62 7.62 7.60 7.60 7.58 7.57 7.56 7.55 7.48 7.48 7.46 7.46 7.45 7.44 7.44 7.43 7.42 7.42 7.39 7.38 7.37 7.36 7.35 7.35 7.33 7.33 7.31 7.31 7.31 7.31 7.26 7.24 7.24 7.22 7.22 7.22 7.20 7.20 7.20 7.20
2.53
0.00 TMS
OTf
O
1q
1.00 0.98 1.99 0.98 1.96 1.00
3.03
f1 (ppm)

13C (100.63 MHz, CDCl3)

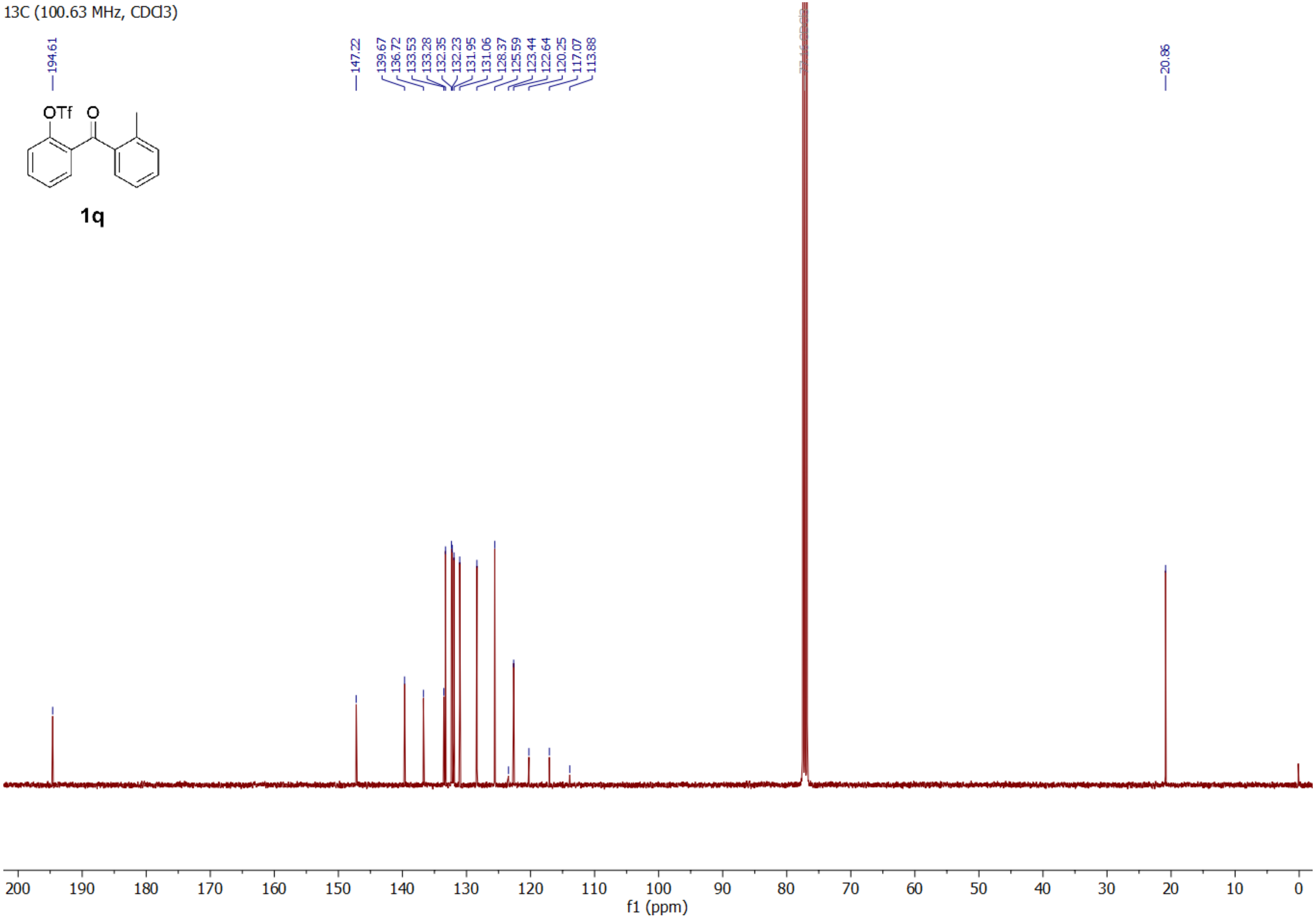

1q

19F (376.48 MHz, CDCl3)

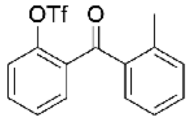

1q

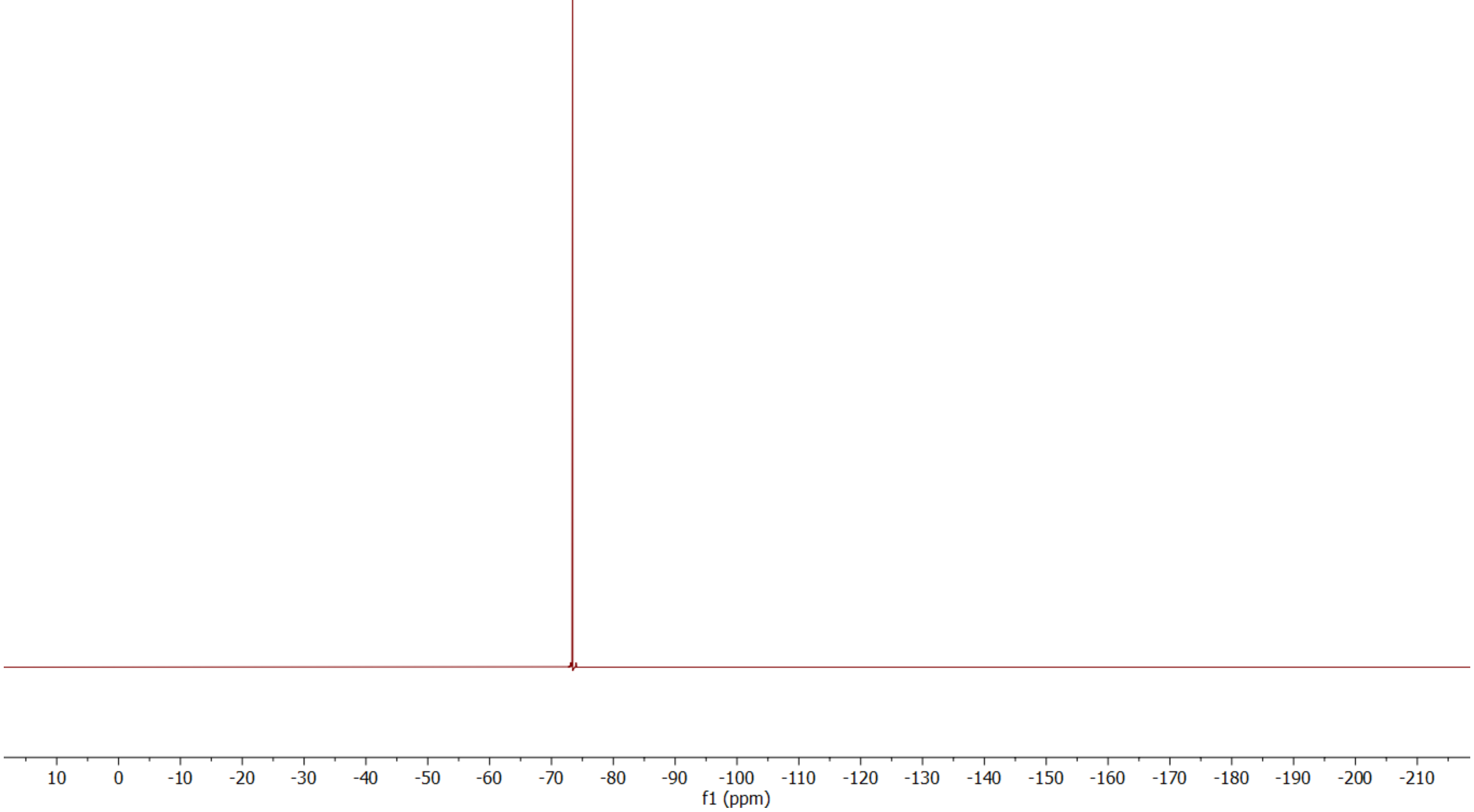

1H (400.15 MHz, CDCl3)

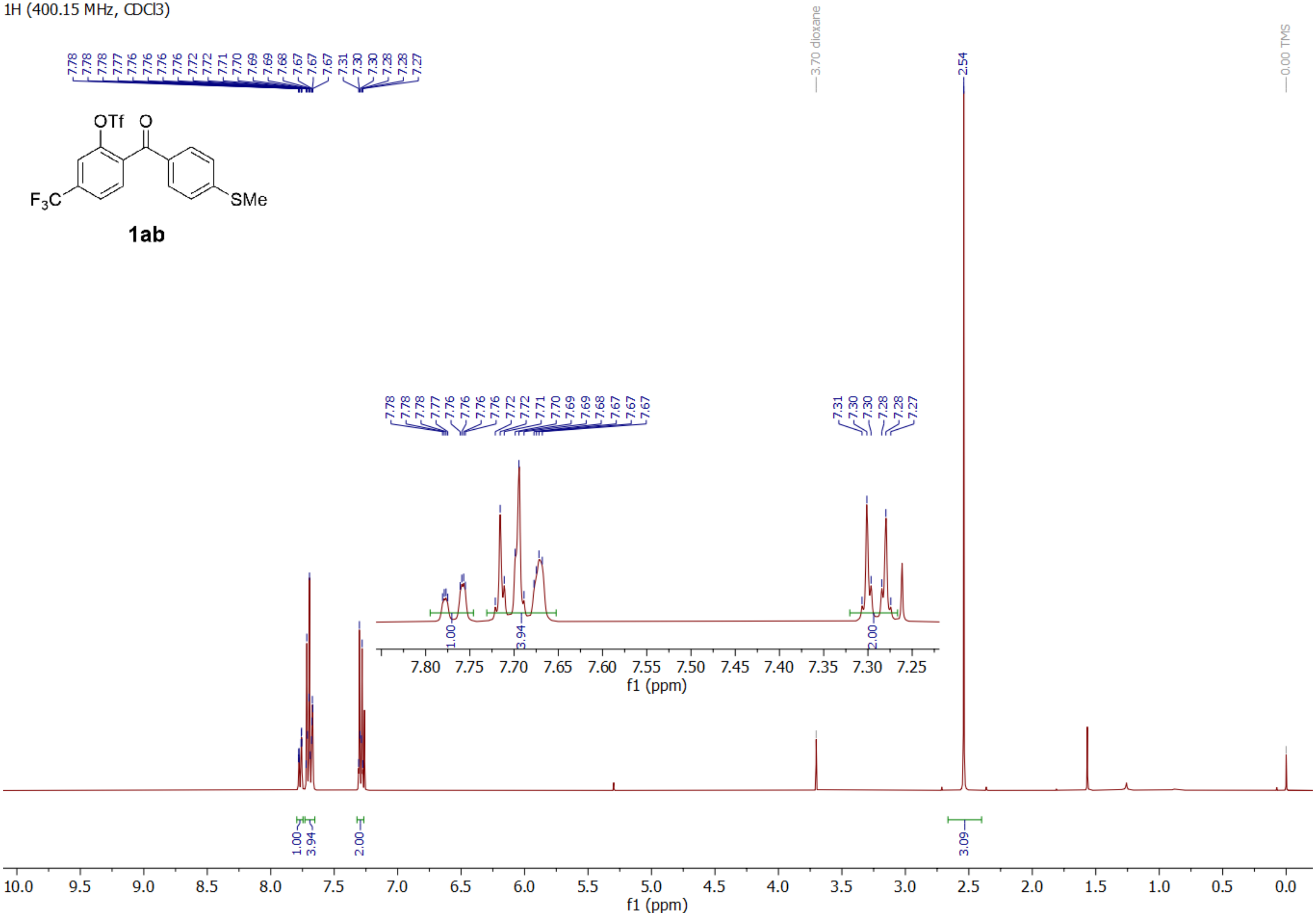

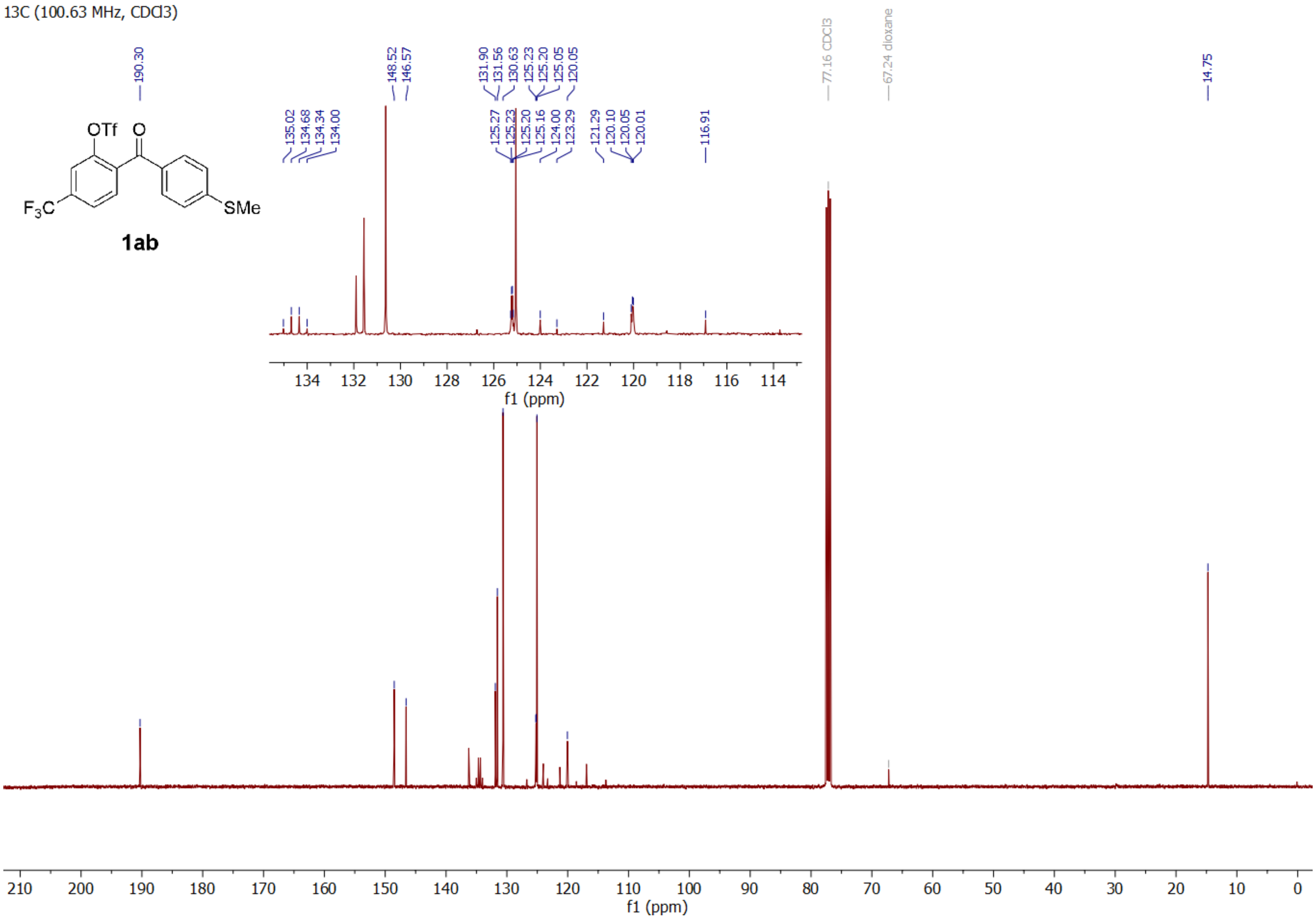
13C (100.63 MHz, CDCl3)
190.30
148.52
146.57
131.90
131.56
130.63
125.23
125.20
125.05
120.05
77.16 CDCl3
67.24 dioxane
14.75
135.02
134.68
134.34
134.00
125.27
125.23
125.20
125.16
124.00
123.29
121.29
120.10
120.05
120.01
116.91
OTf
O
F3C
SMe
1ab
f1 (ppm)
f1 (ppm)

19F (376.48 MHz, CDCl3)

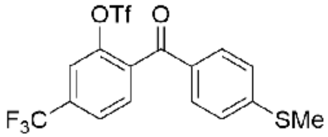

1ab

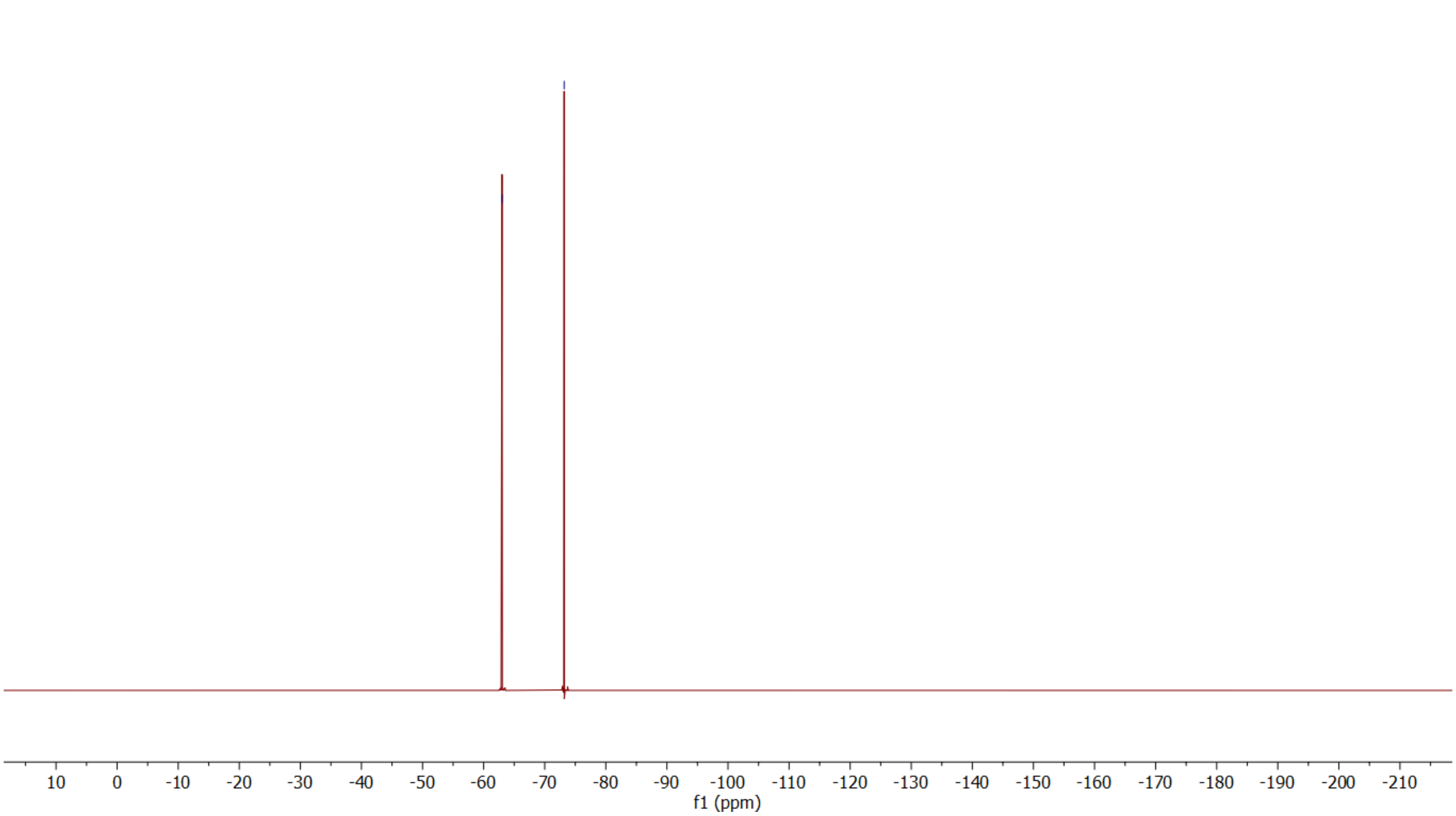

1H (400.15 MHz, CDCl3)

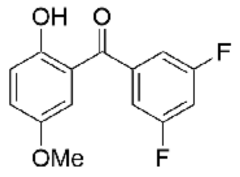

S4

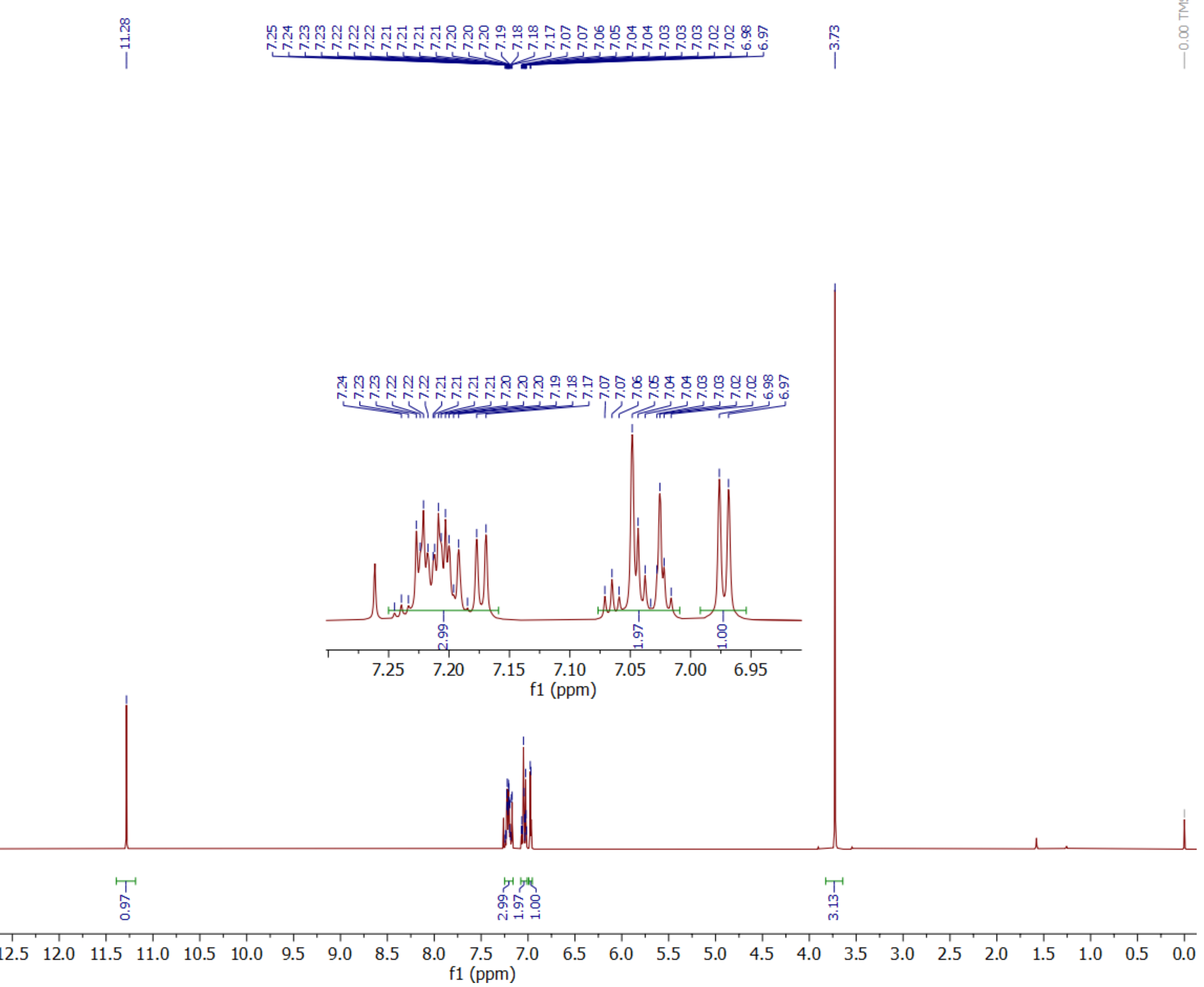

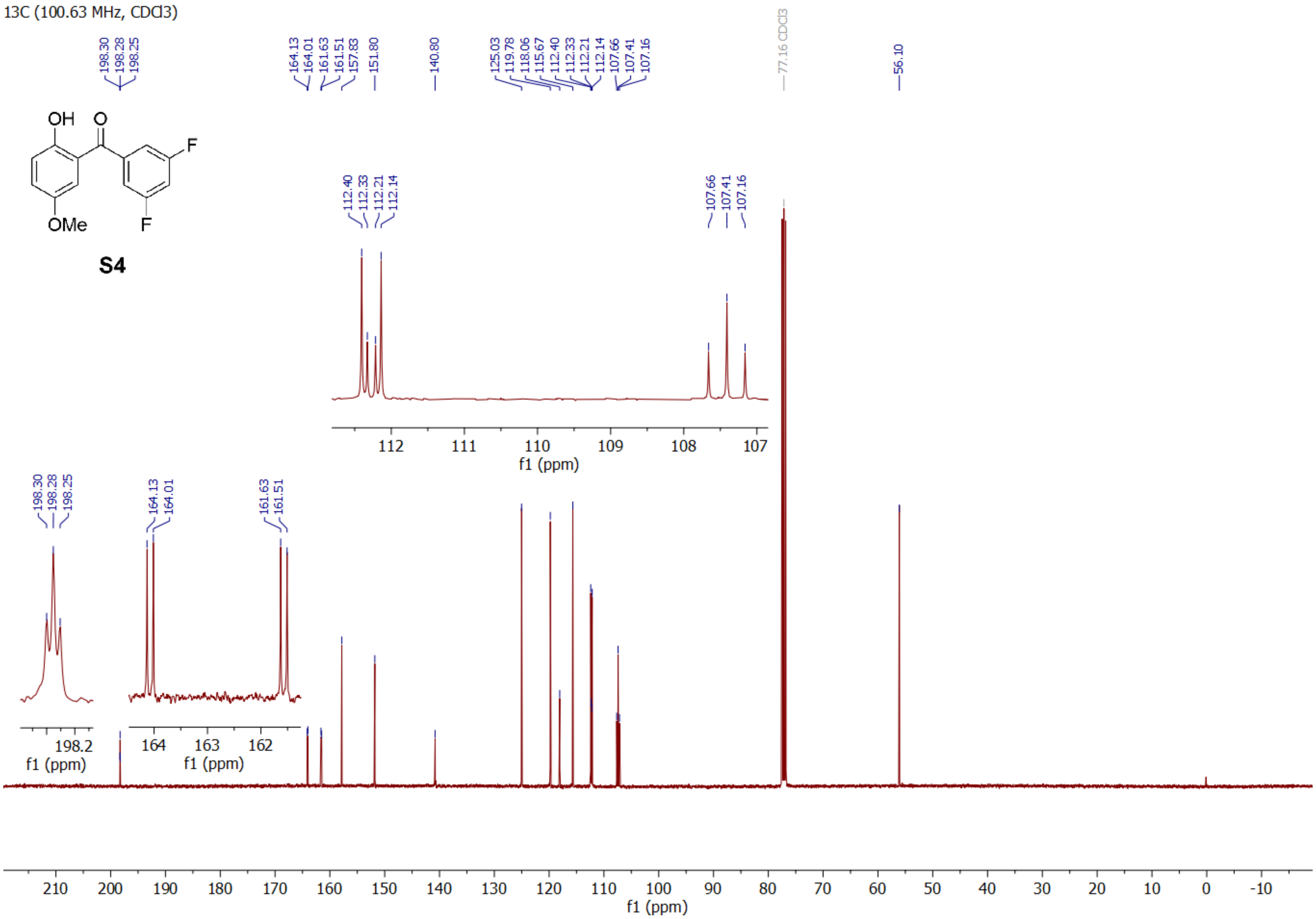
13C (100.63 MHz, CDCl3)
198.30
198.28
198.25
164.13
164.01
161.63
161.51
157.83
151.80
140.80
125.03
119.78
118.06
115.67
112.40
112.33
112.21
112.14
107.66
107.41
107.16
77.16 CDCl3
56.10
OH
O
F
OMe
F
S4
f1 (ppm)

19F (376.48 MHz, CDCl3)

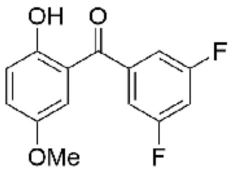

S4

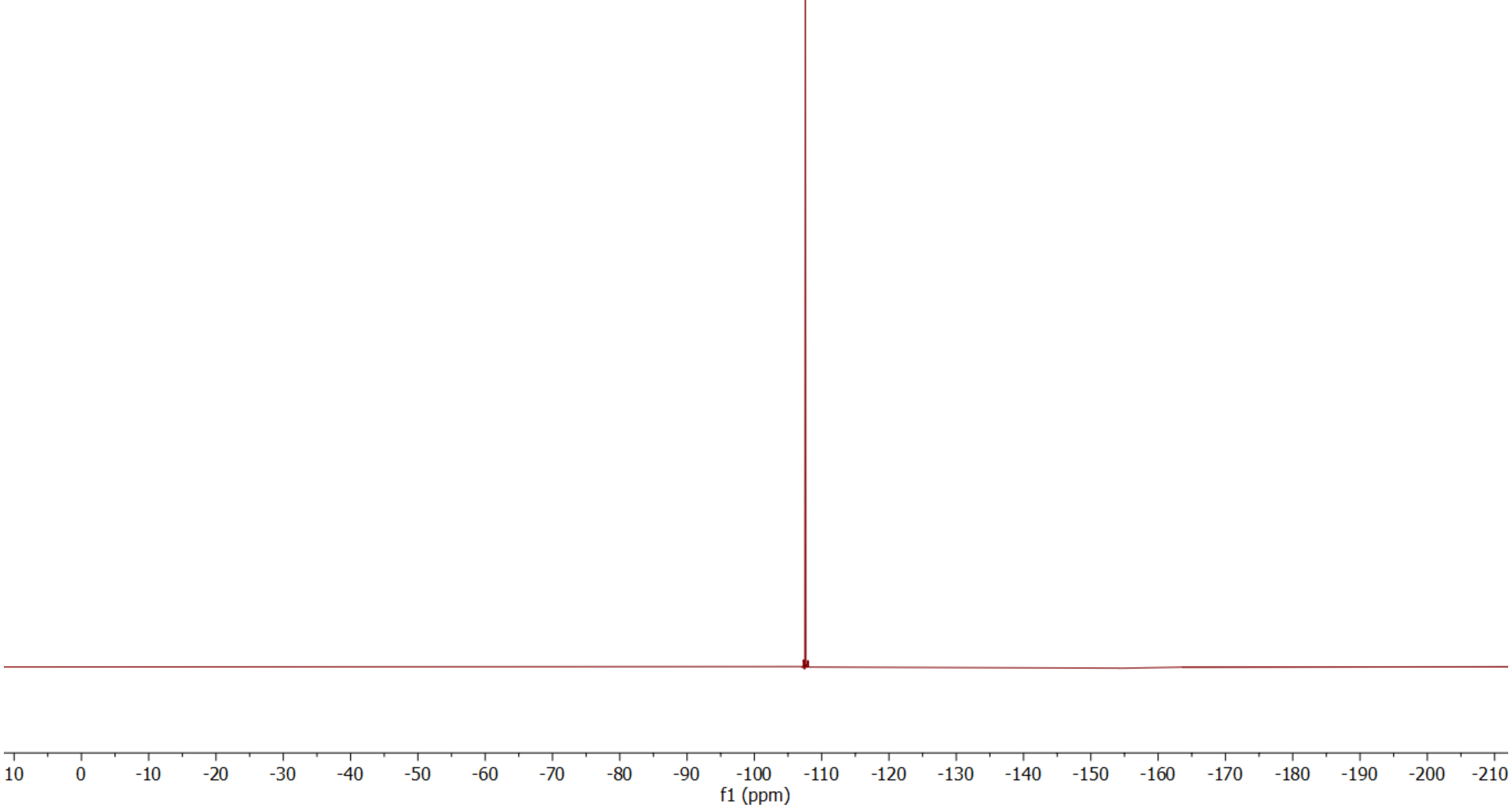

1H (400.15 MHz, CDCl3)

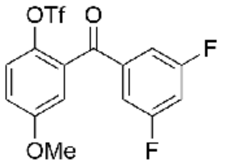

1ad

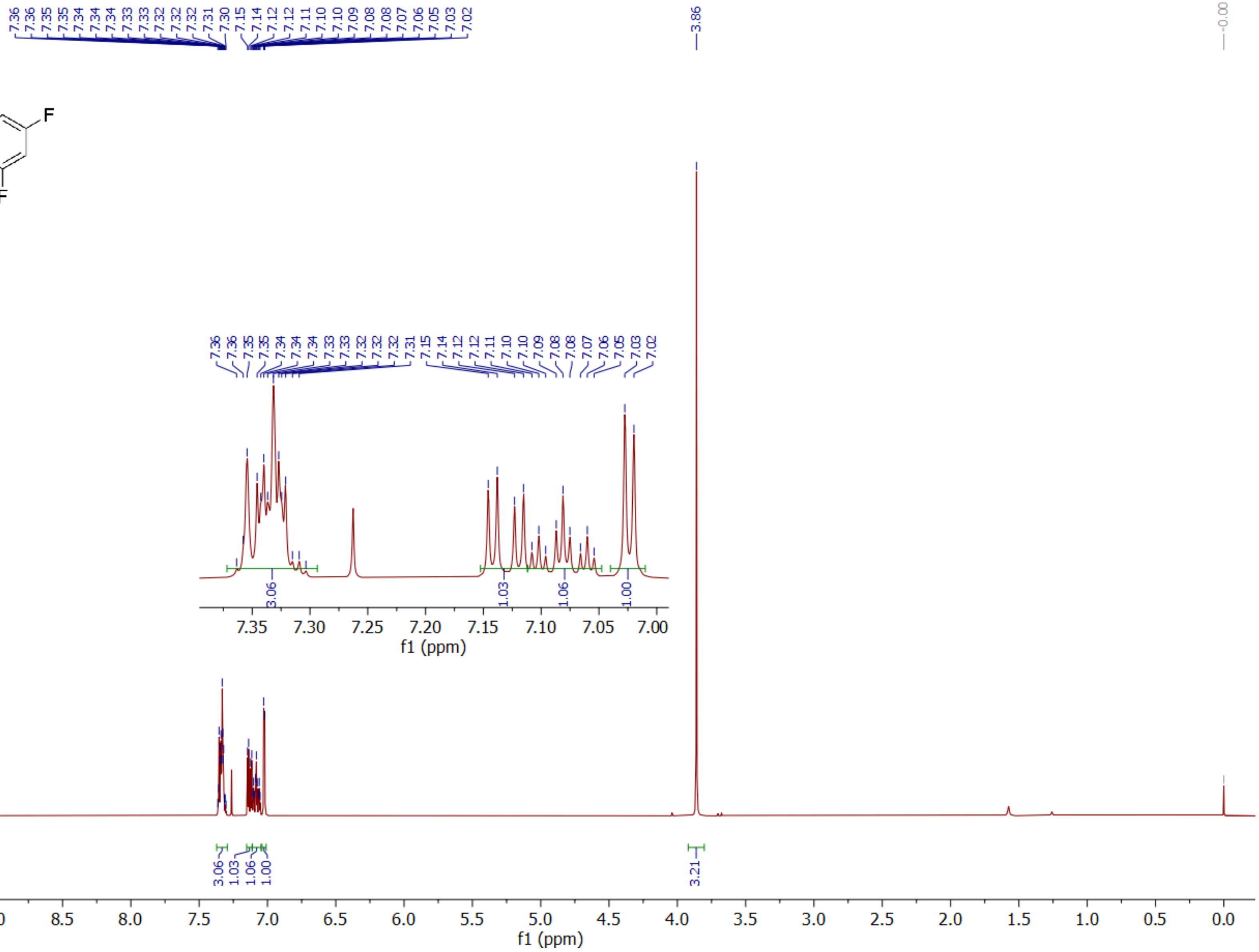

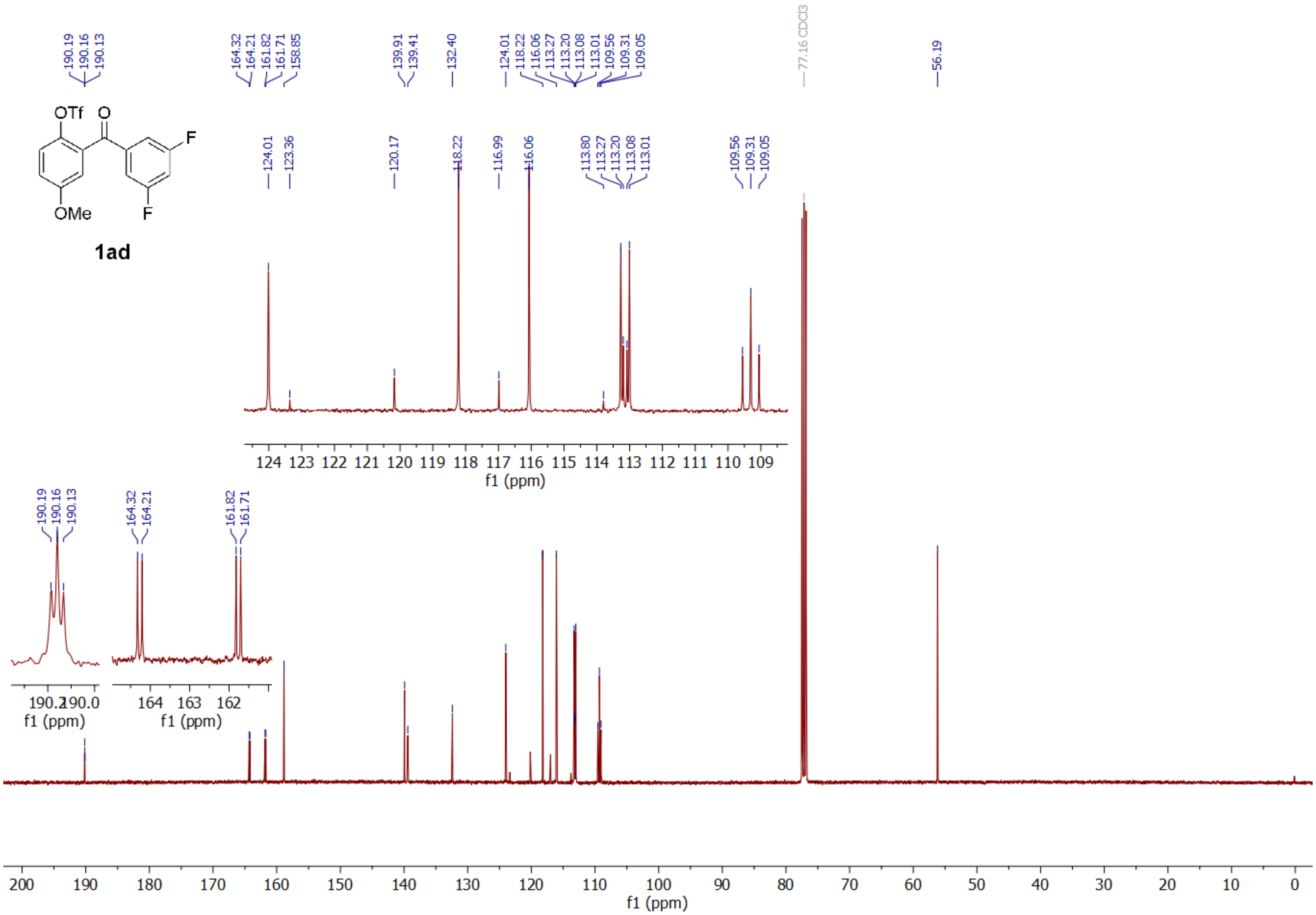
190.19
190.16
190.13
164.32
164.21
161.82
161.71
158.85
139.91
139.41
132.40
124.01
118.22
116.06
113.27
113.20
113.08
113.01
109.56
109.31
109.05
77.16 CDCl3
56.19
OTf
O
F
F
OMe
f1 (ppm)

1ad

19F (376.48 MHz, CDCl3)

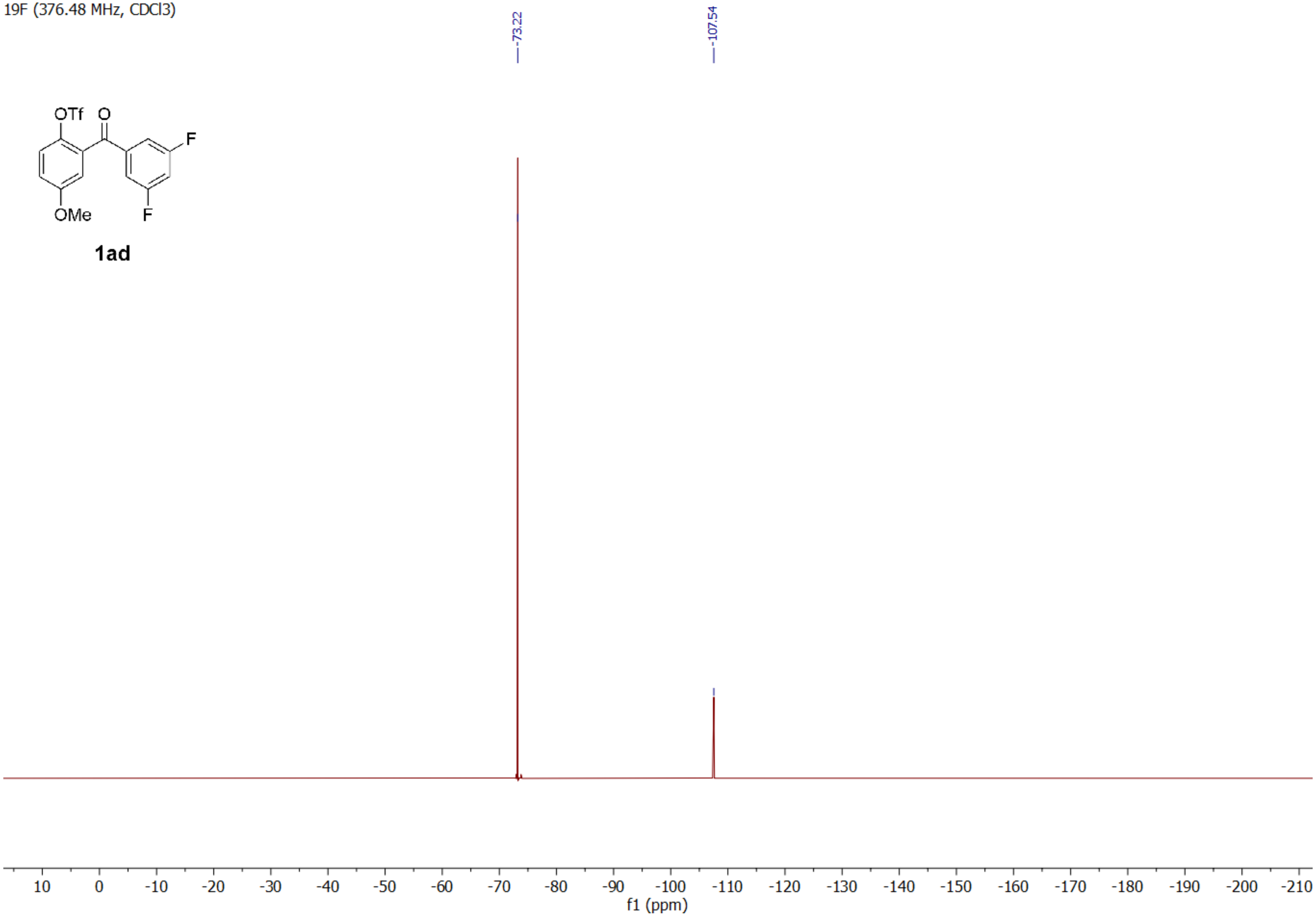

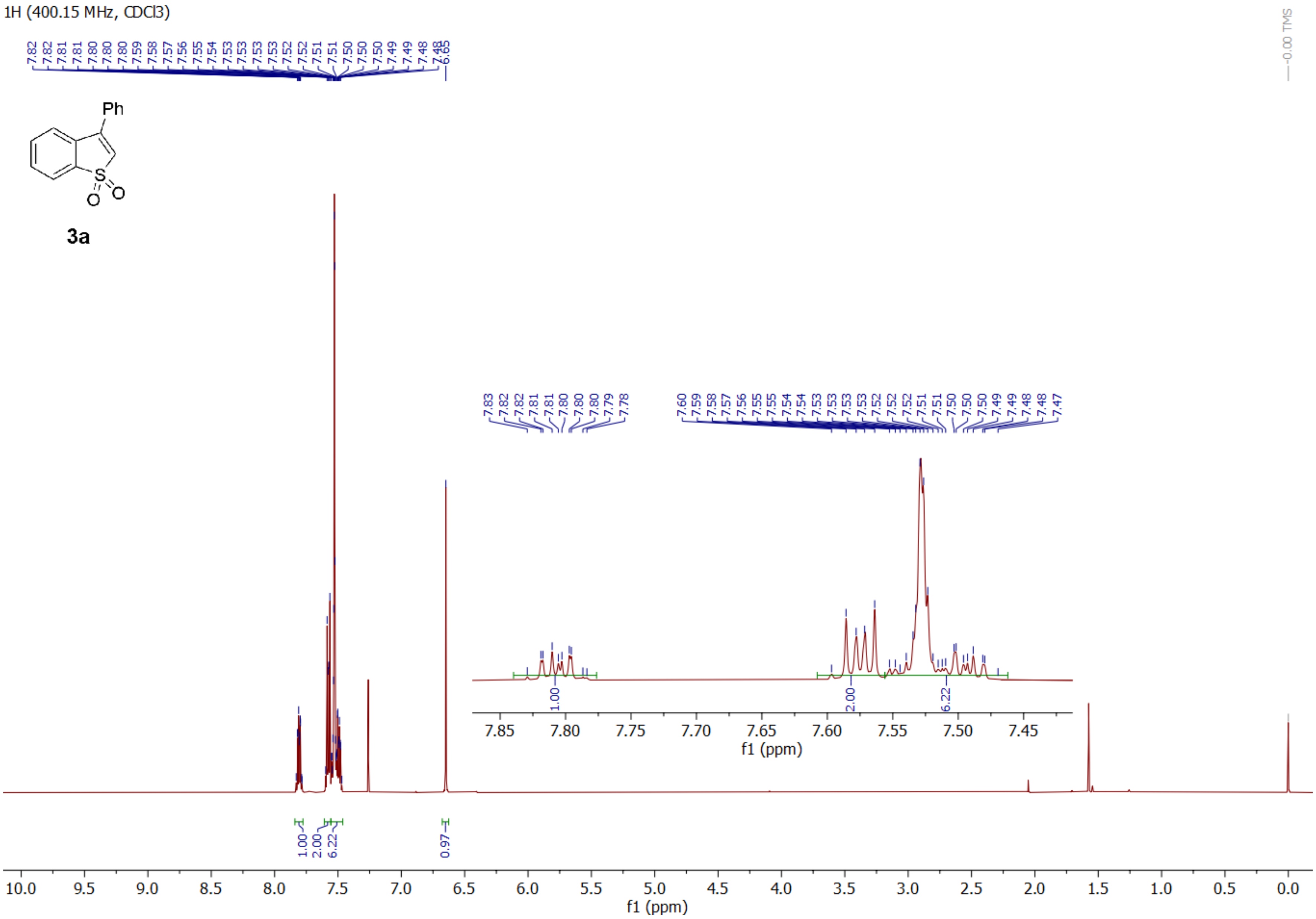
1H (400.15 MHz, CDCl3)
Ph
S
O
O
3a
0.00 TMS
1.00
2.00
6.22
0.97
f1 (ppm)

13C (100.63 MHz, CDCl3)

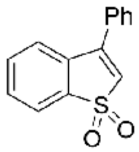

3a

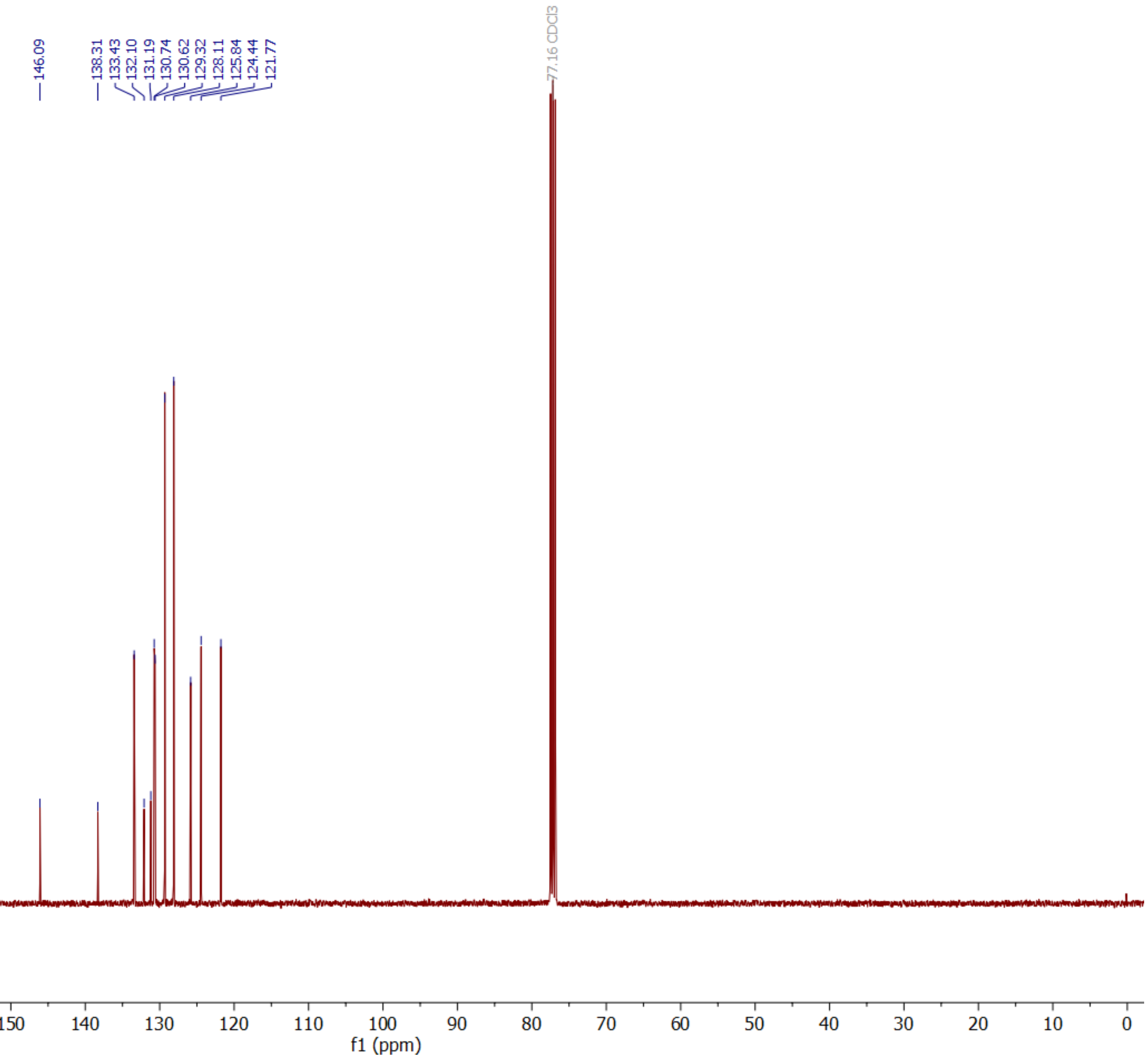

1H (400.15 MHz, CDCl3)

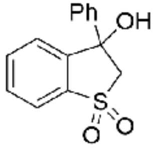

2'a

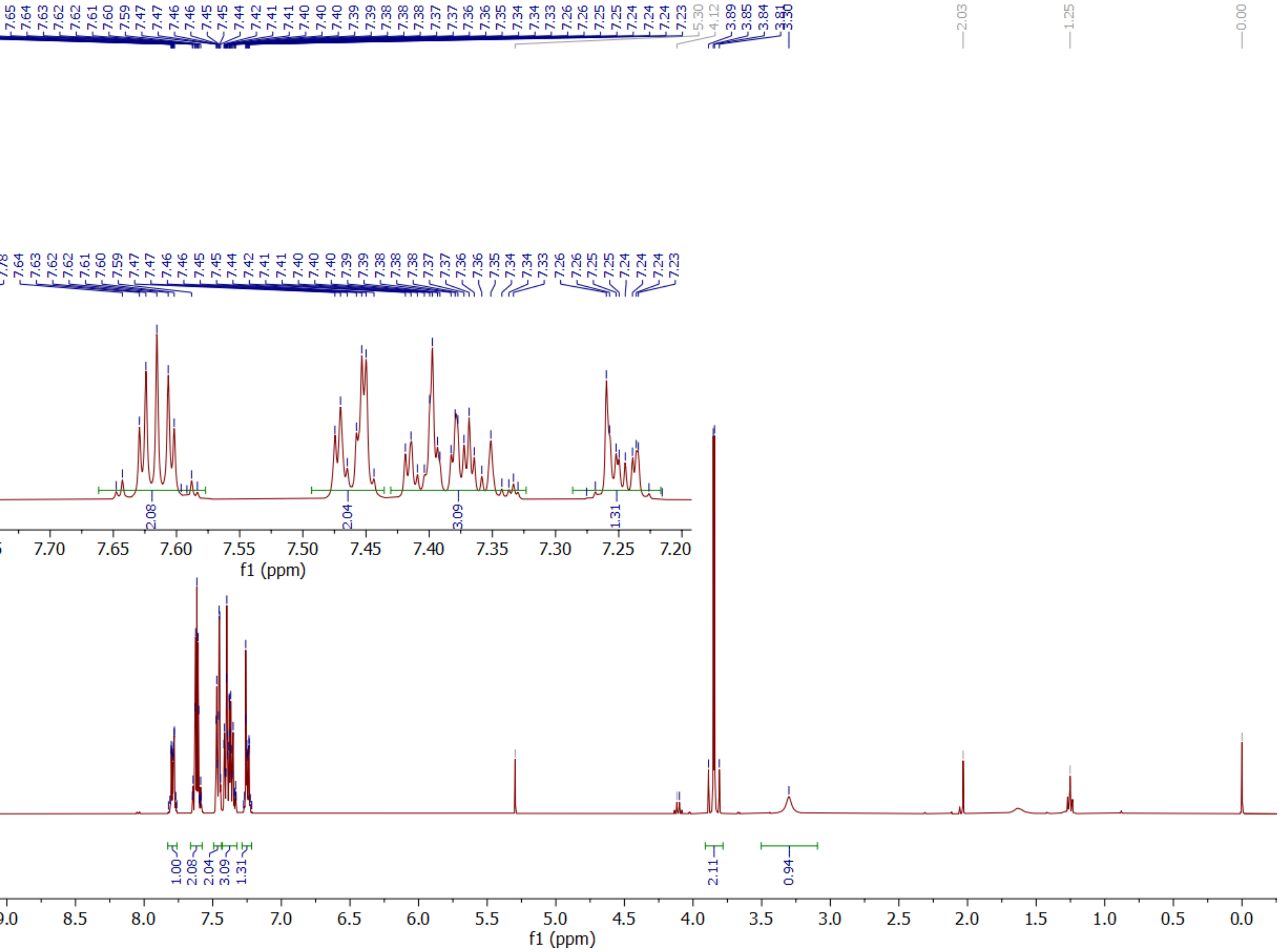

13C (100.63 MHz, CDCl3)

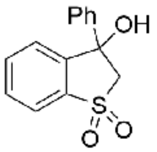

2'a

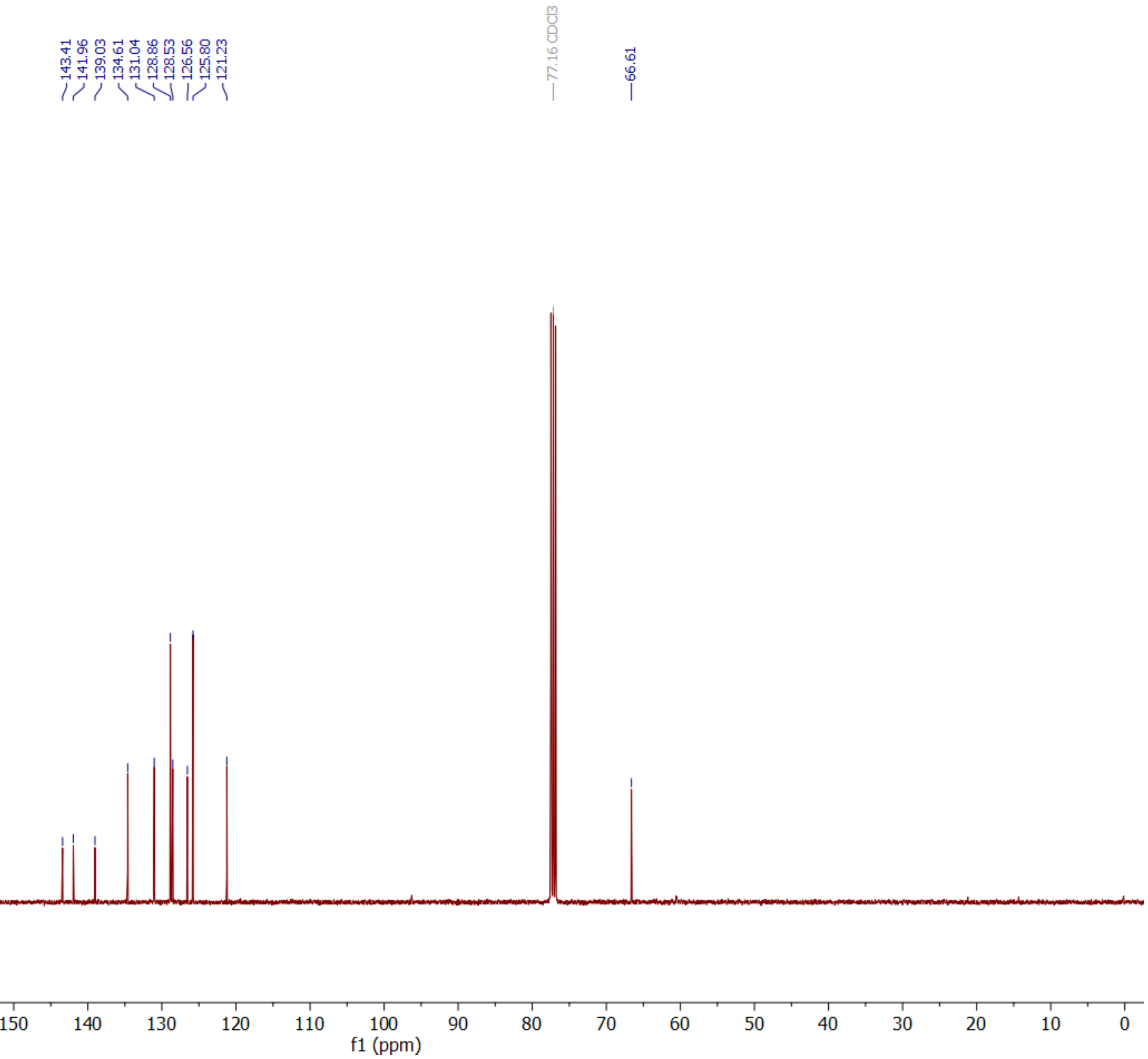

1H (400.15 MHz, CDCl3)

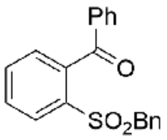

2b

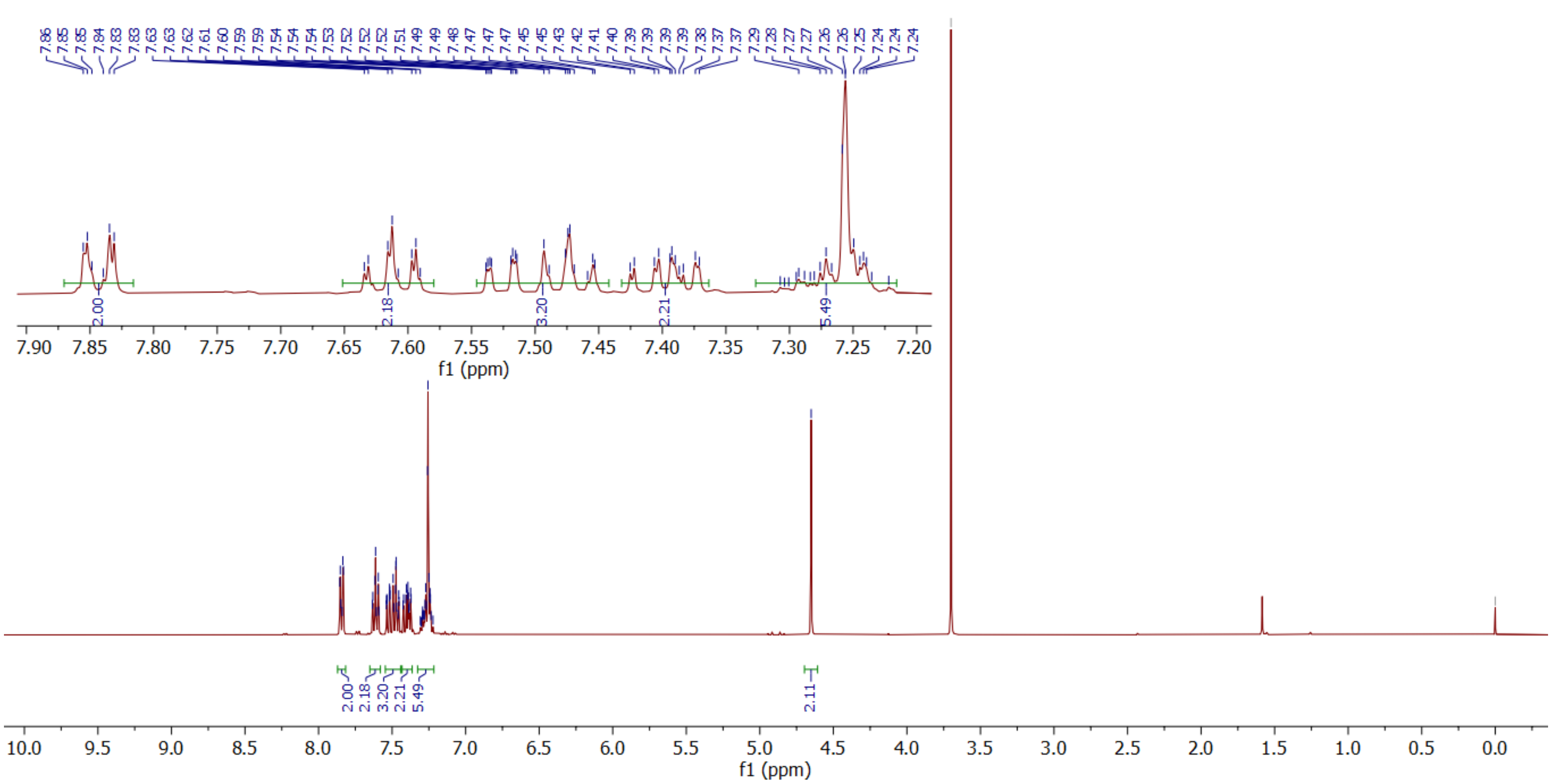

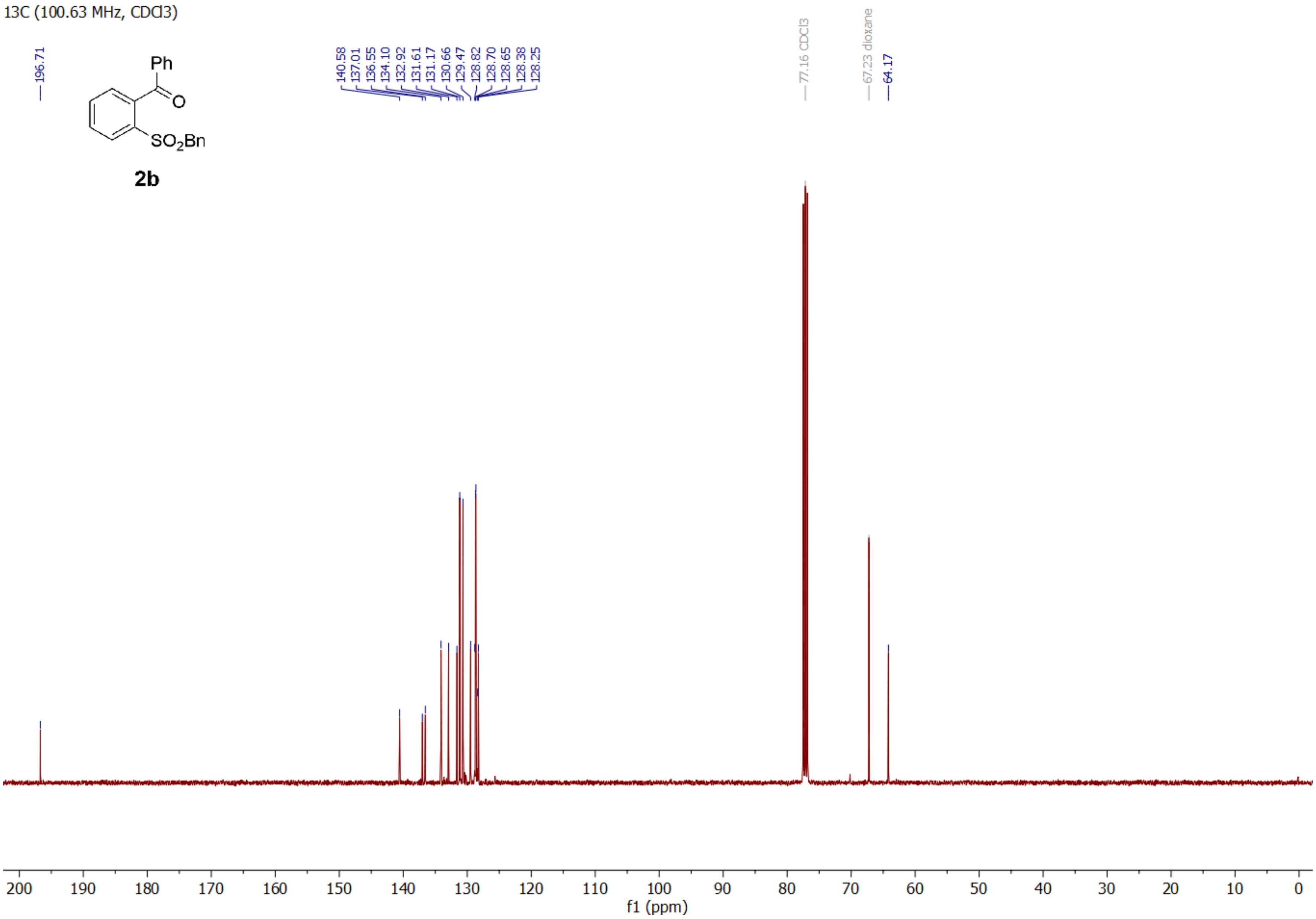
13C (100.63 MHz, CDCl3)
196.71
Ph
O
SO2Bn
2b
140.58
137.01
136.55
134.10
132.92
131.61
131.17
130.66
129.47
128.82
128.70
128.65
128.38
128.25
77.16 CDCl3
67.23 dioxane
64.17
200 190 180 170 160 150 140 130 120 110 100 90 80 70 60 50 40 30 20 10 0
f1 (ppm)

1H (400.15 MHz, CDCl3)

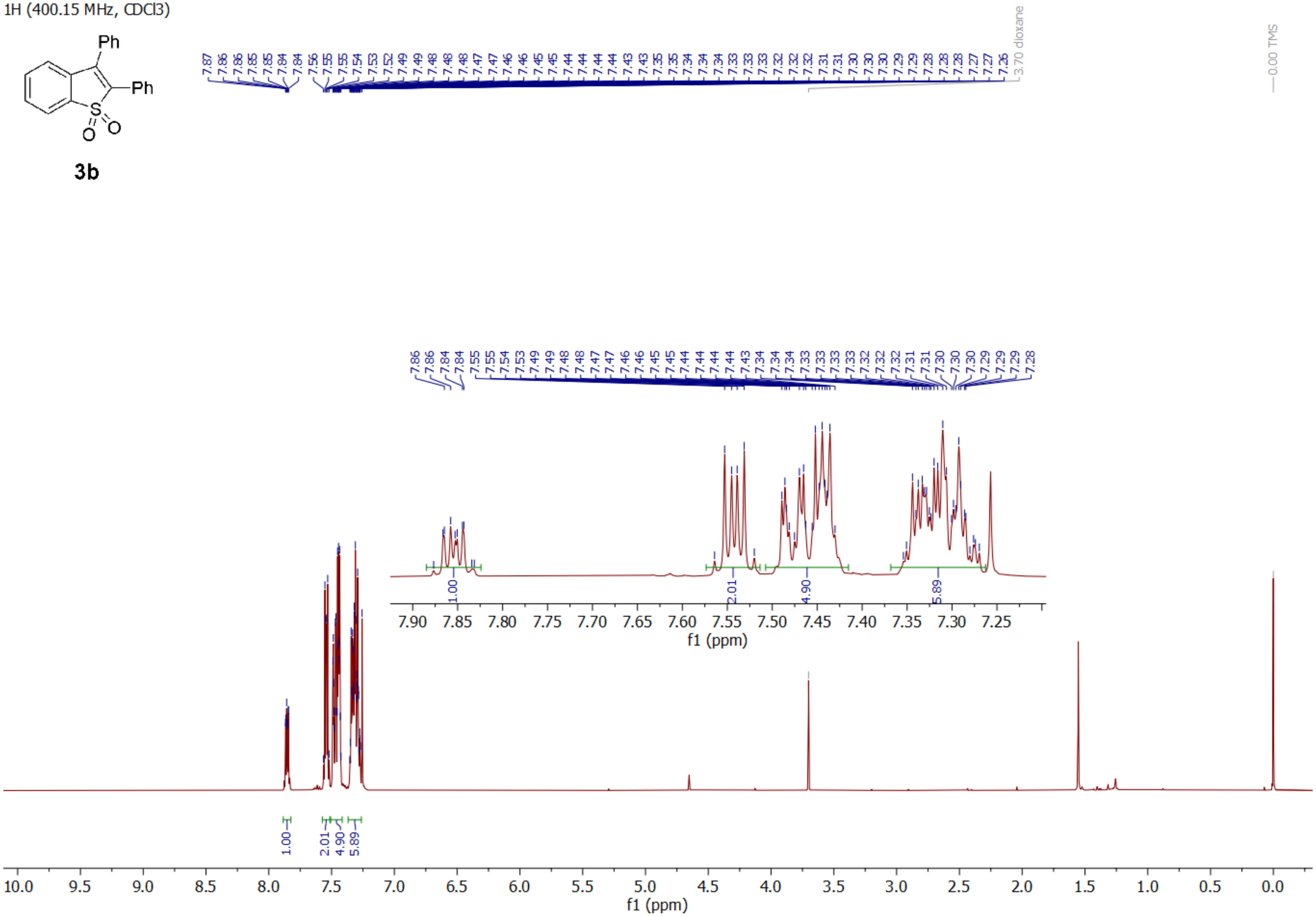

3b

13C (100.63 MHz, CDCl3)

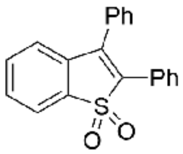

3b

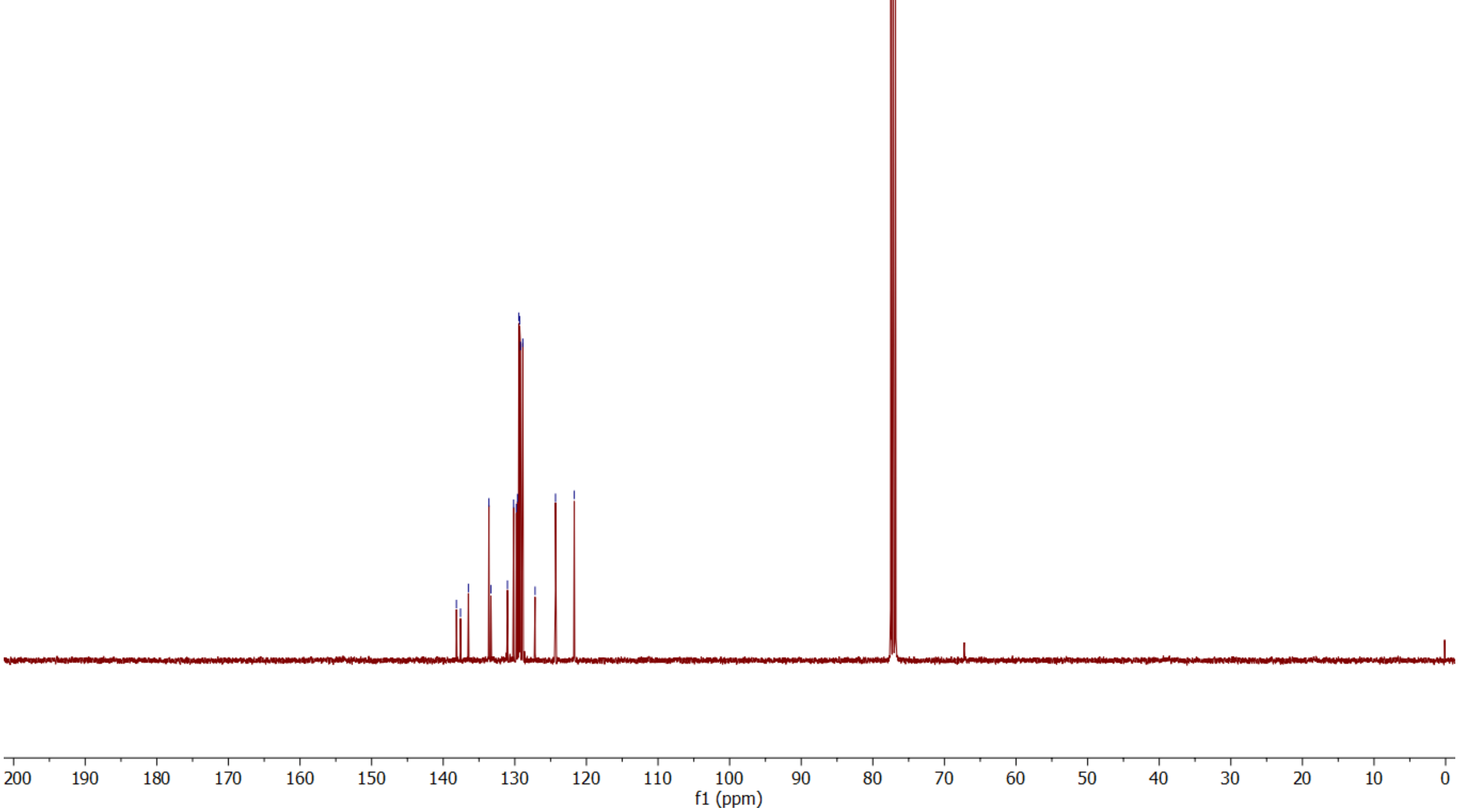

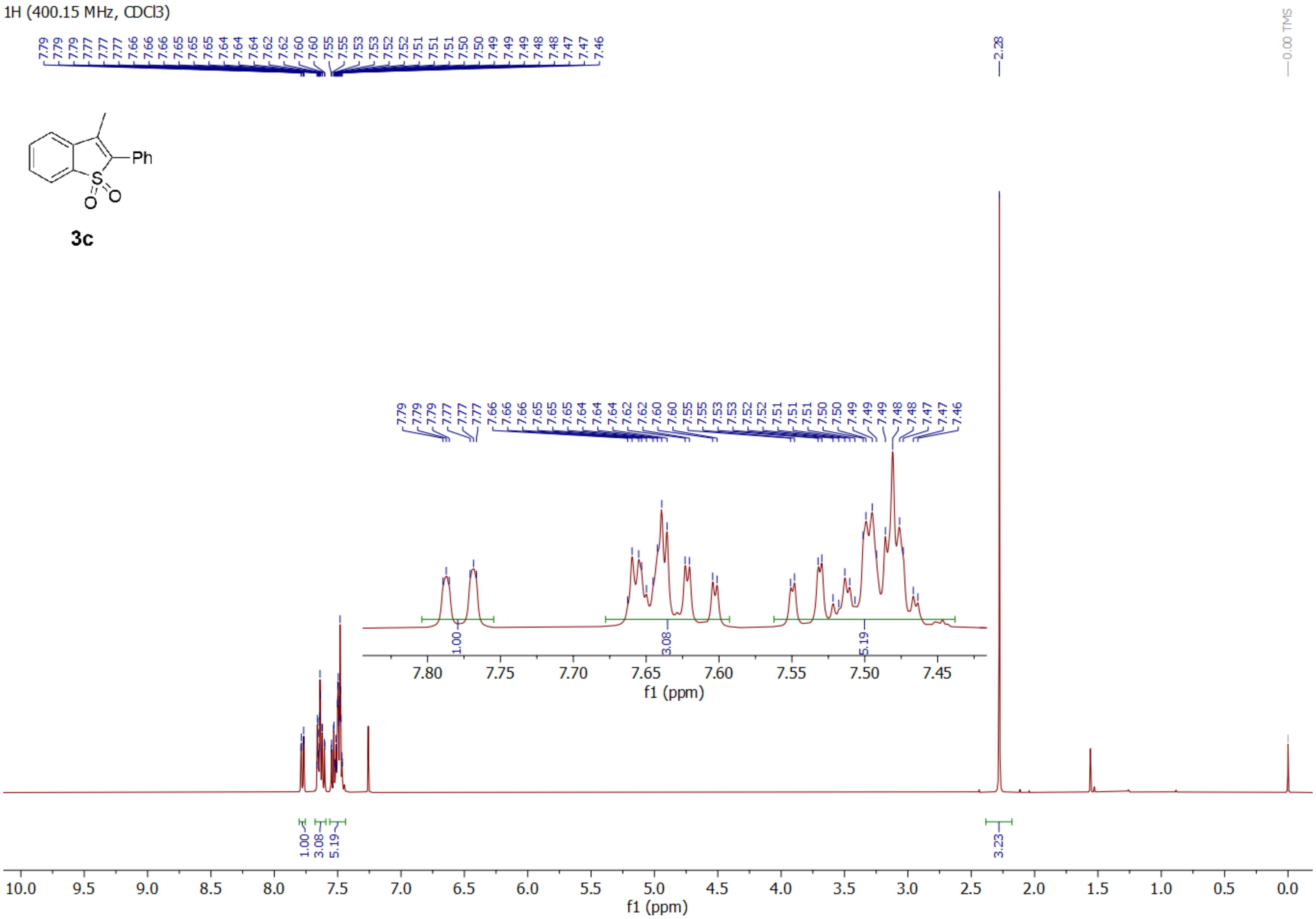

3c

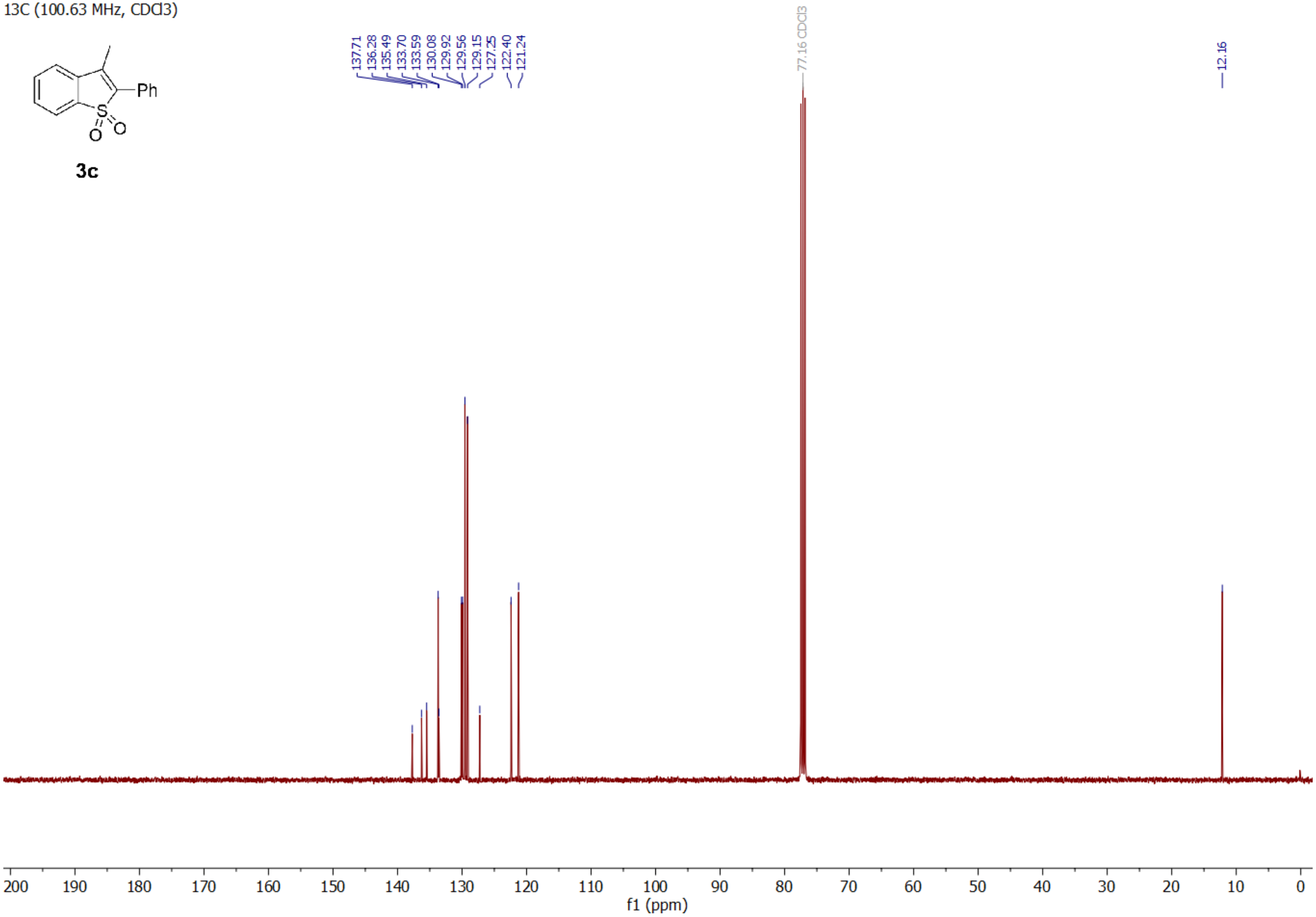
13C (100.63 MHz, CDCl3)
Ph
S
O
O
3c
137.71
136.28
135.49
133.70
133.59
130.08
129.92
129.56
129.15
127.25
122.40
121.24
77.16 CDCl3
12.16
200
190
180
170
160
150
140
130
120
110
100
90
80
70
60
50
40
30
20
10
0
f1 (ppm)

1H (400.15 MHz, CDCl3)

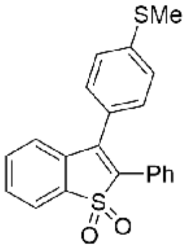

3d

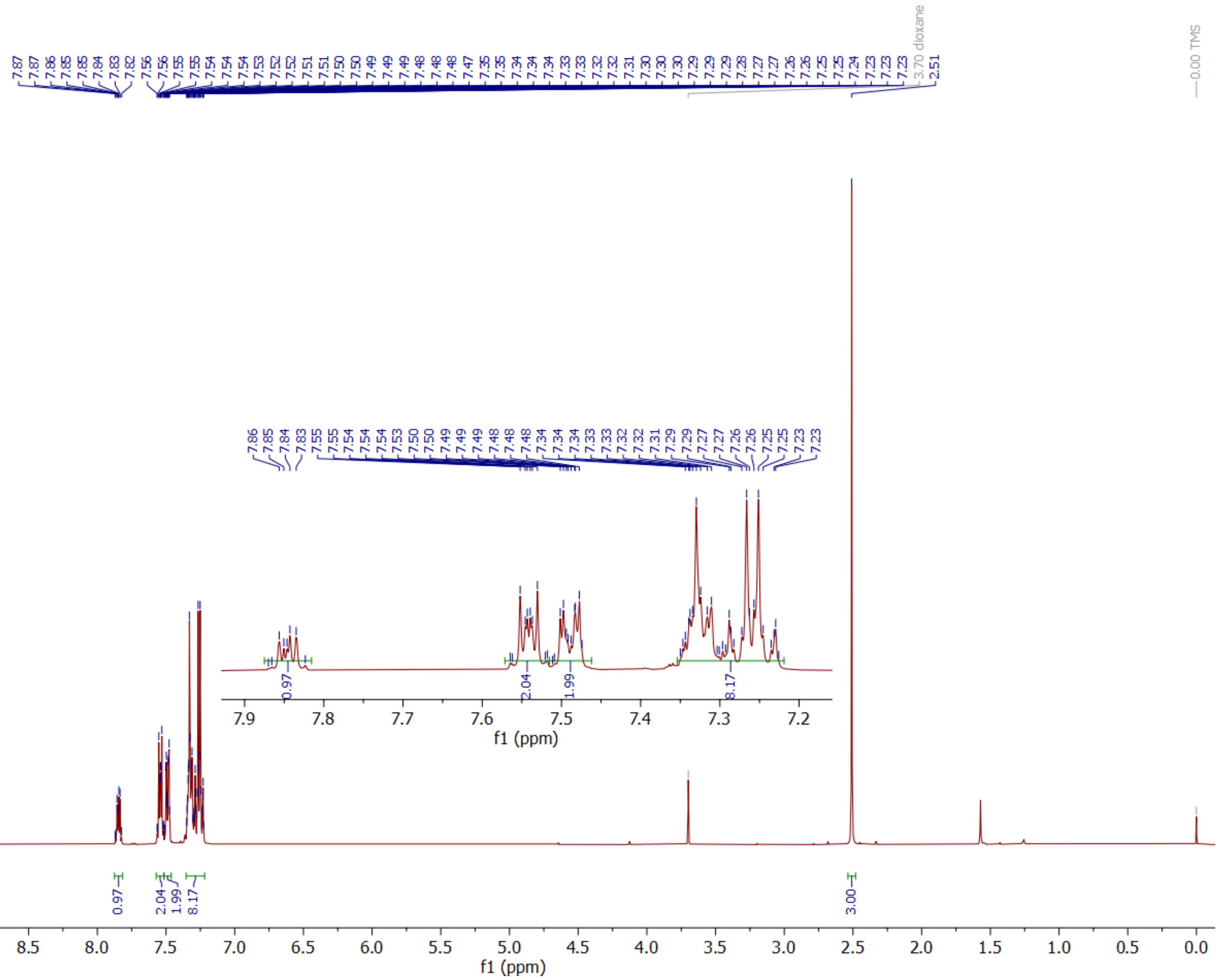

13C (100.63 MHz, CDCl3)

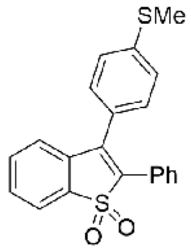

3d

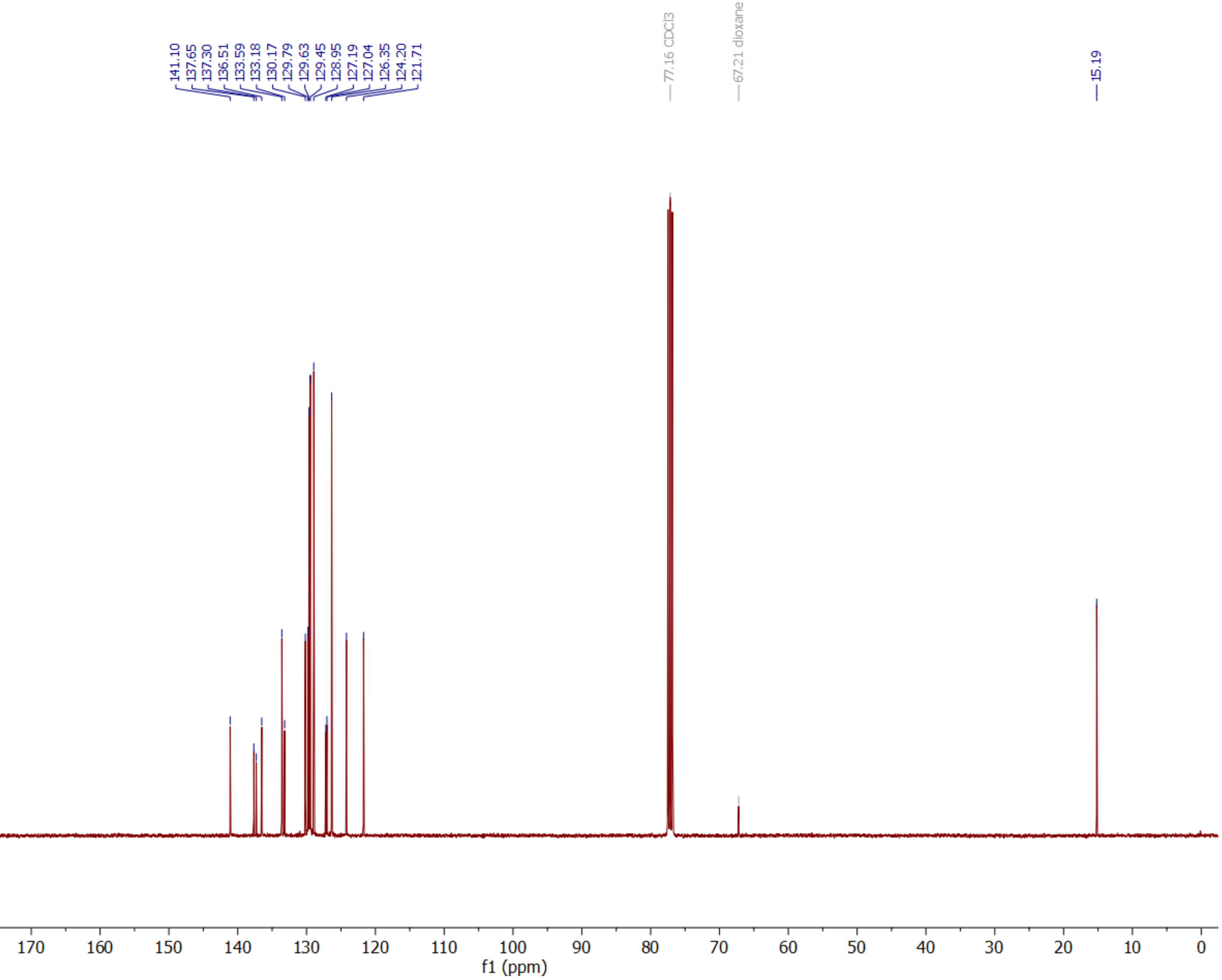

1H (400.15 MHz, CDCl3)

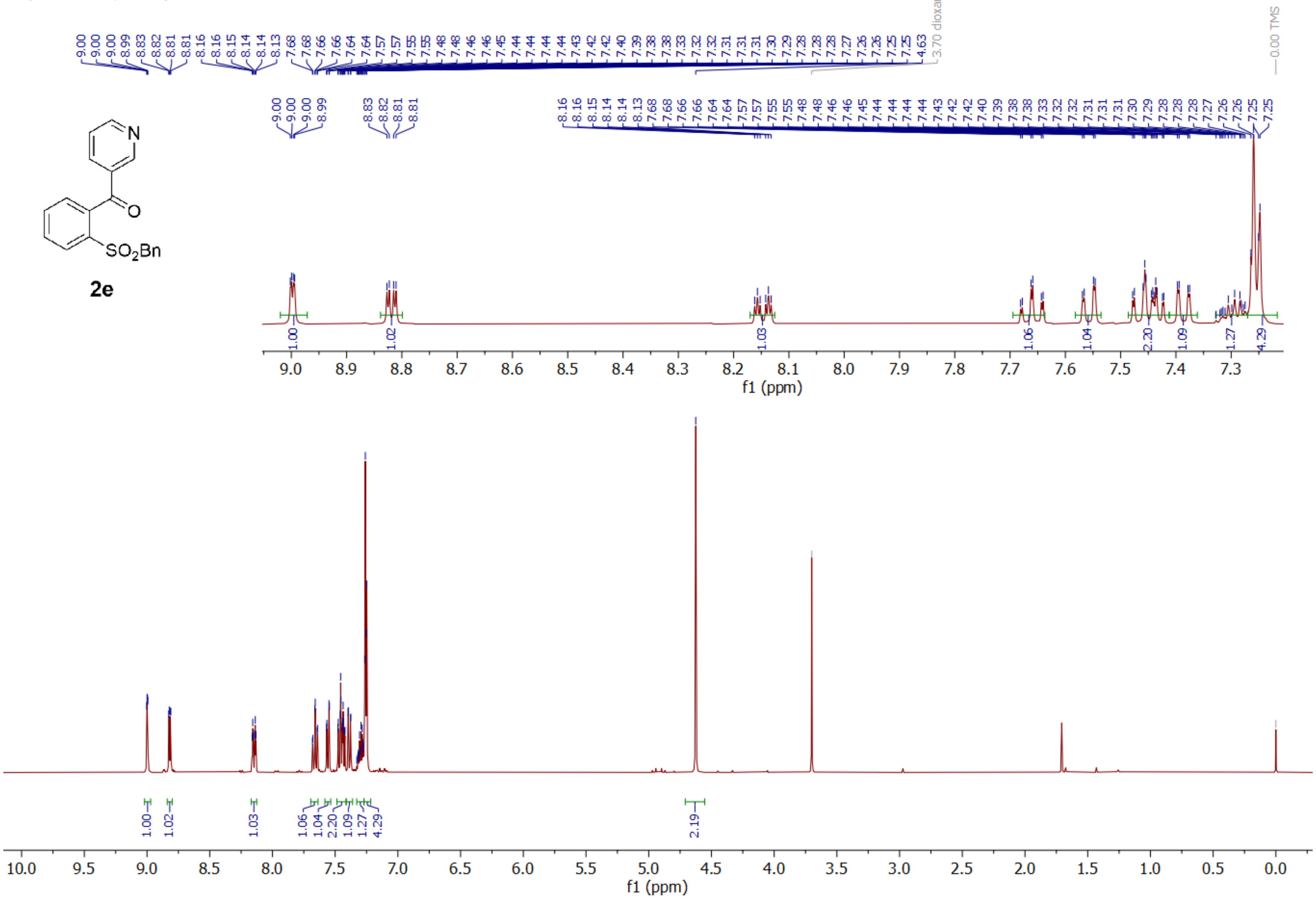

2e

13C (100.63 MHz, CDCl3)

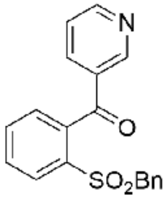

2e

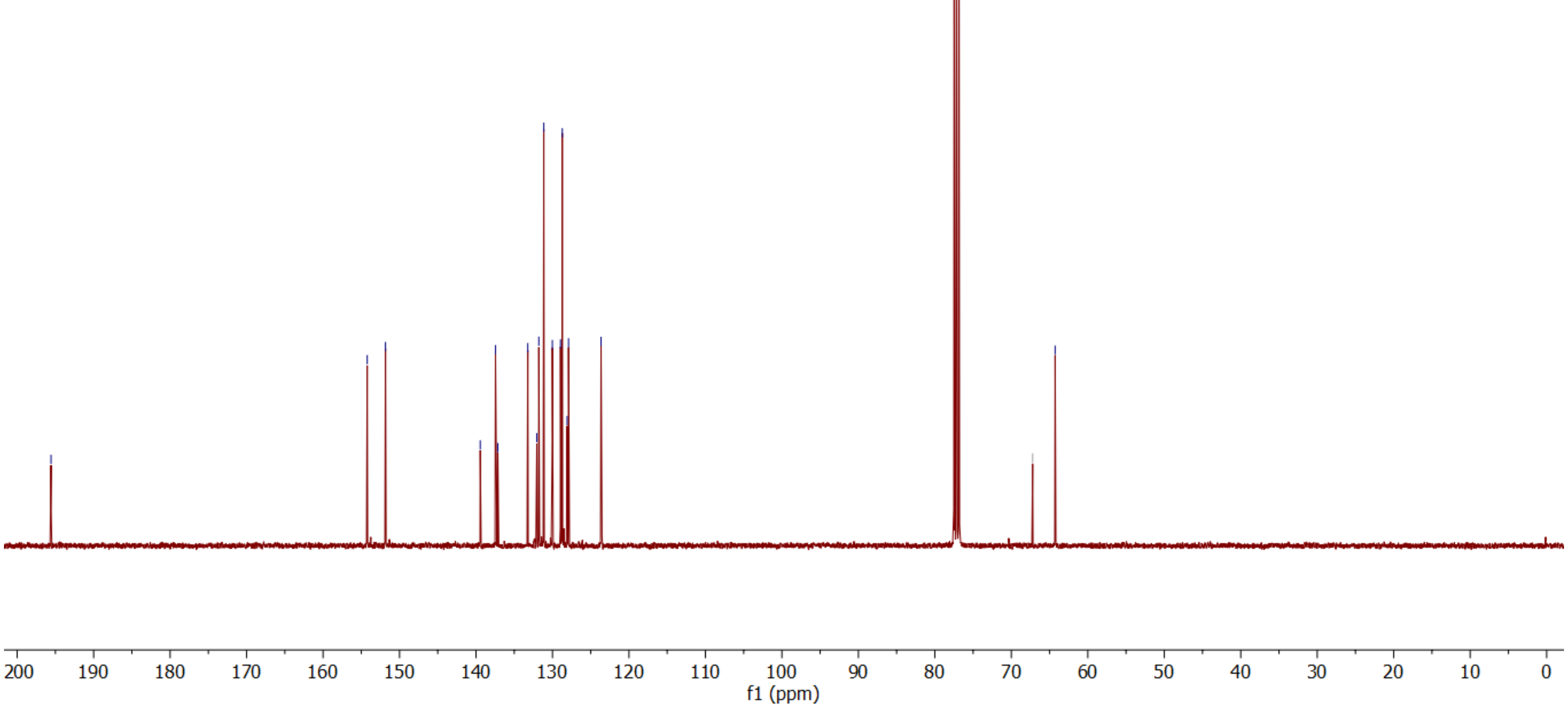

1H (400.15 MHz, CDCl3)

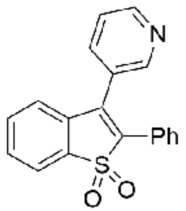

3e

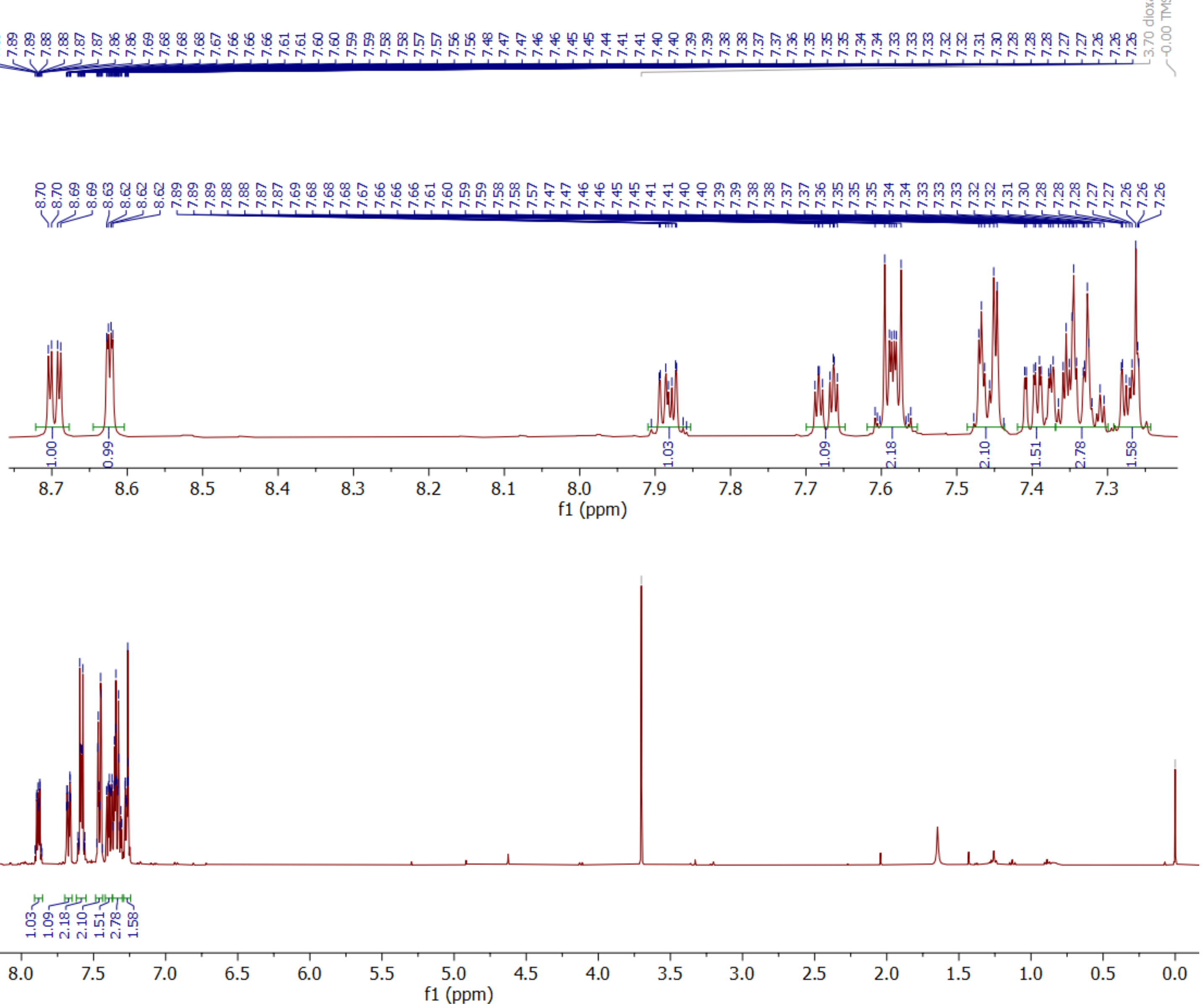

13C (100.63 MHz, CDCl3)

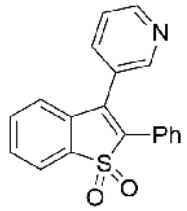

3e

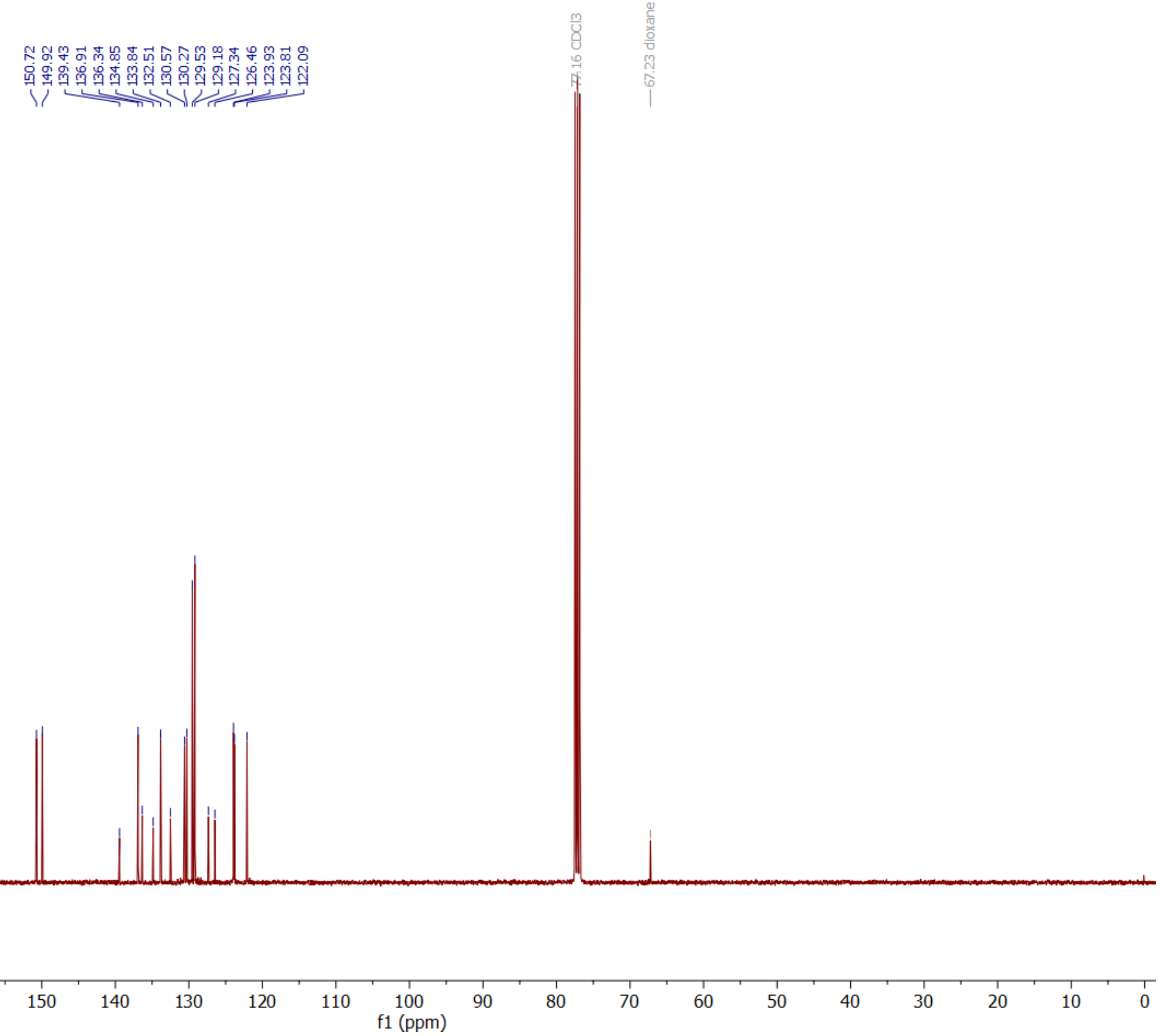

1H (400.15 MHz, CDCl3)

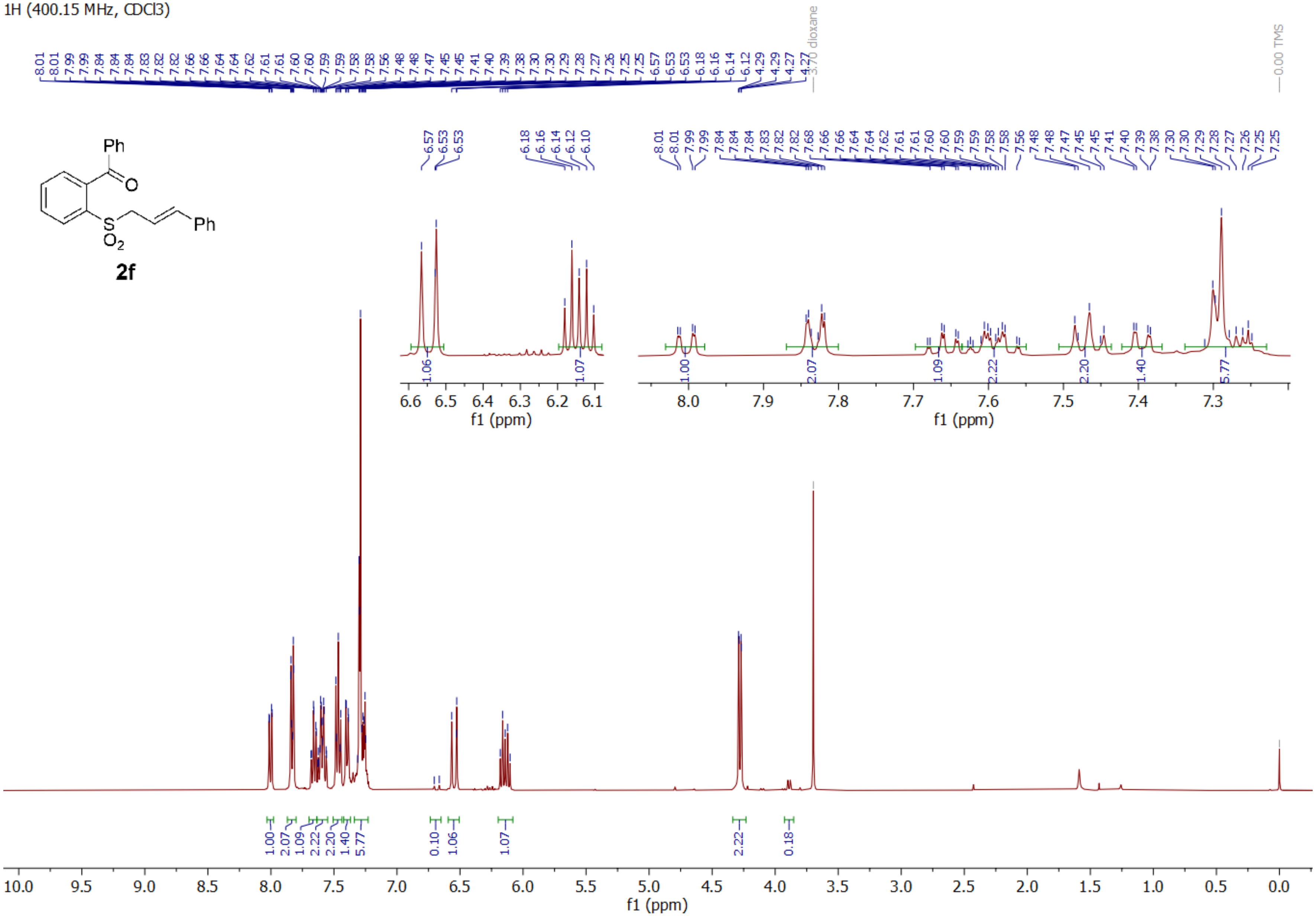

13C (100.63 MHz, CDCl3)

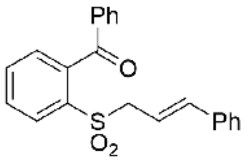

2f

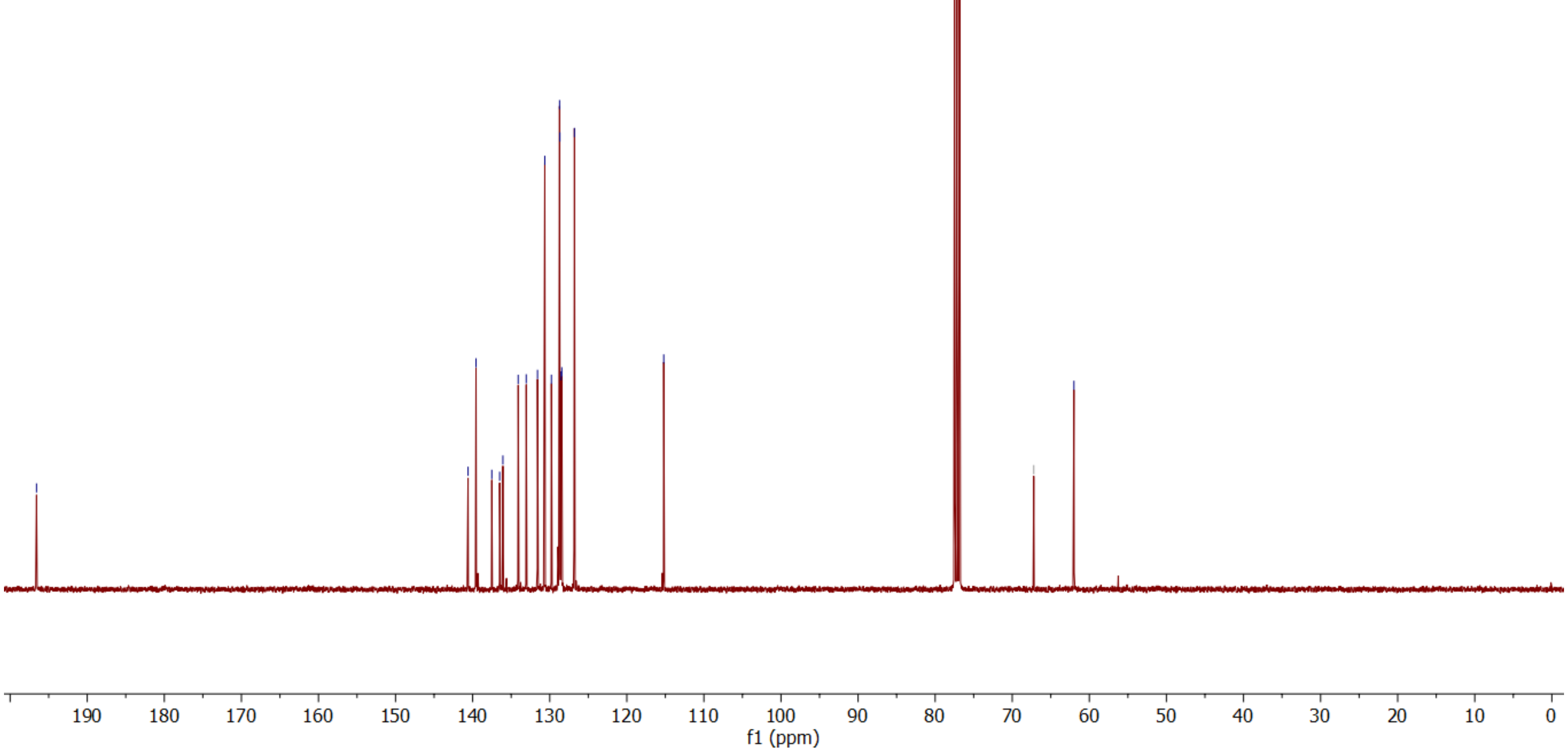

1H (400.15 MHz, CDCl3)

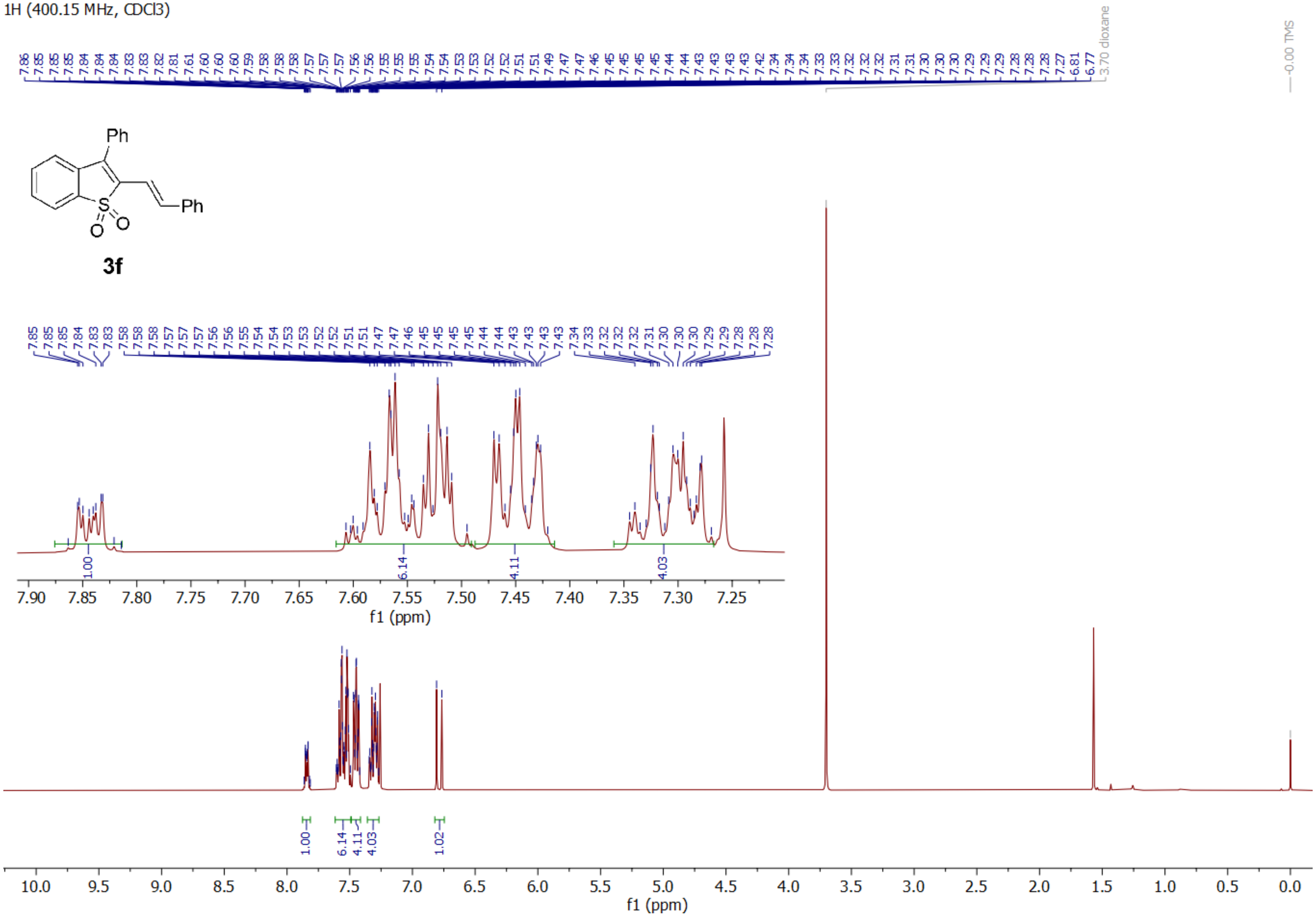

13C (100.63 MHz, CDCl3)

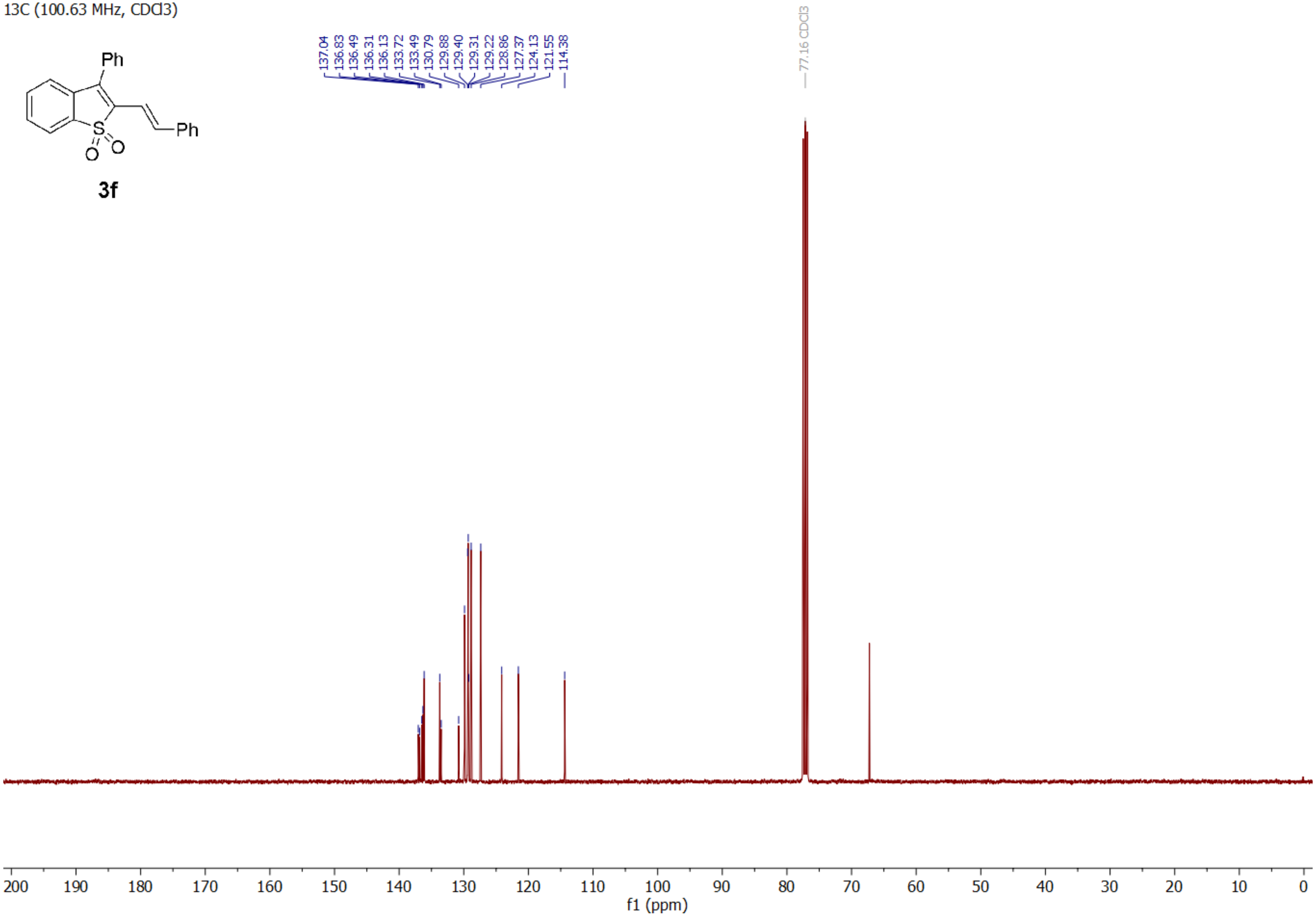

1H (400.15 MHz, CDCl3)

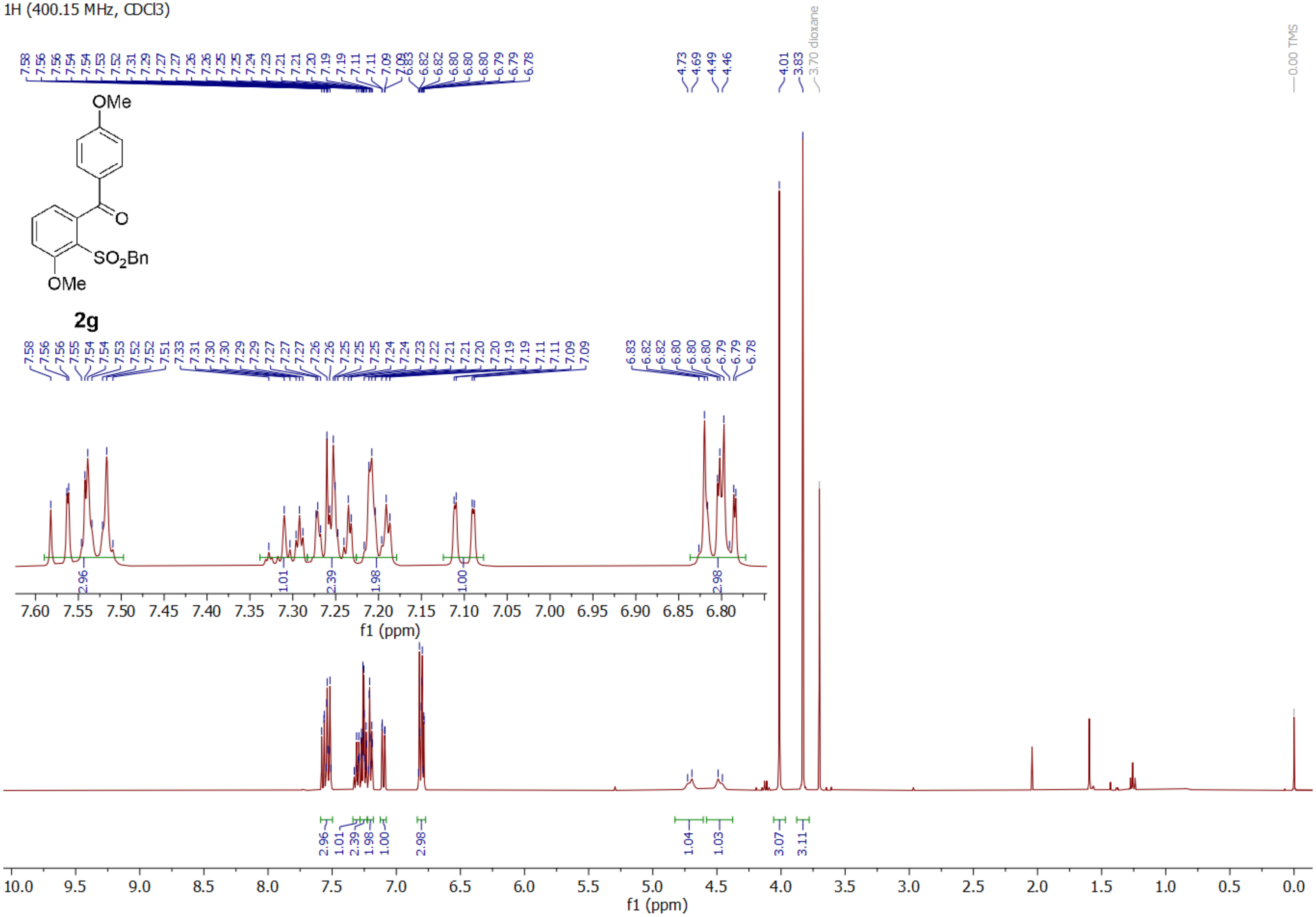

2g

13C (100.63 MHz, CDCl3)

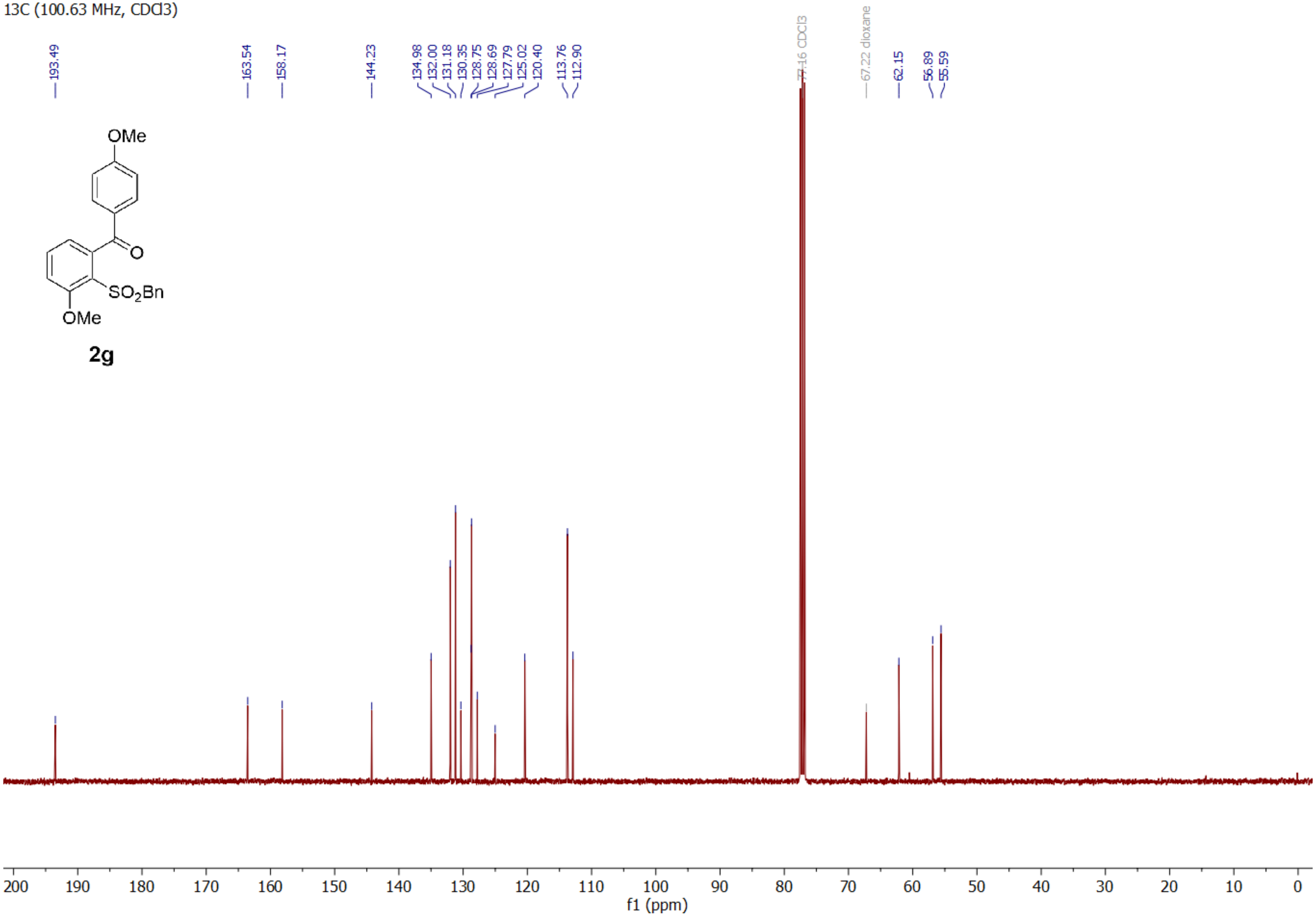

1H (400.15 MHz, CDCl3)

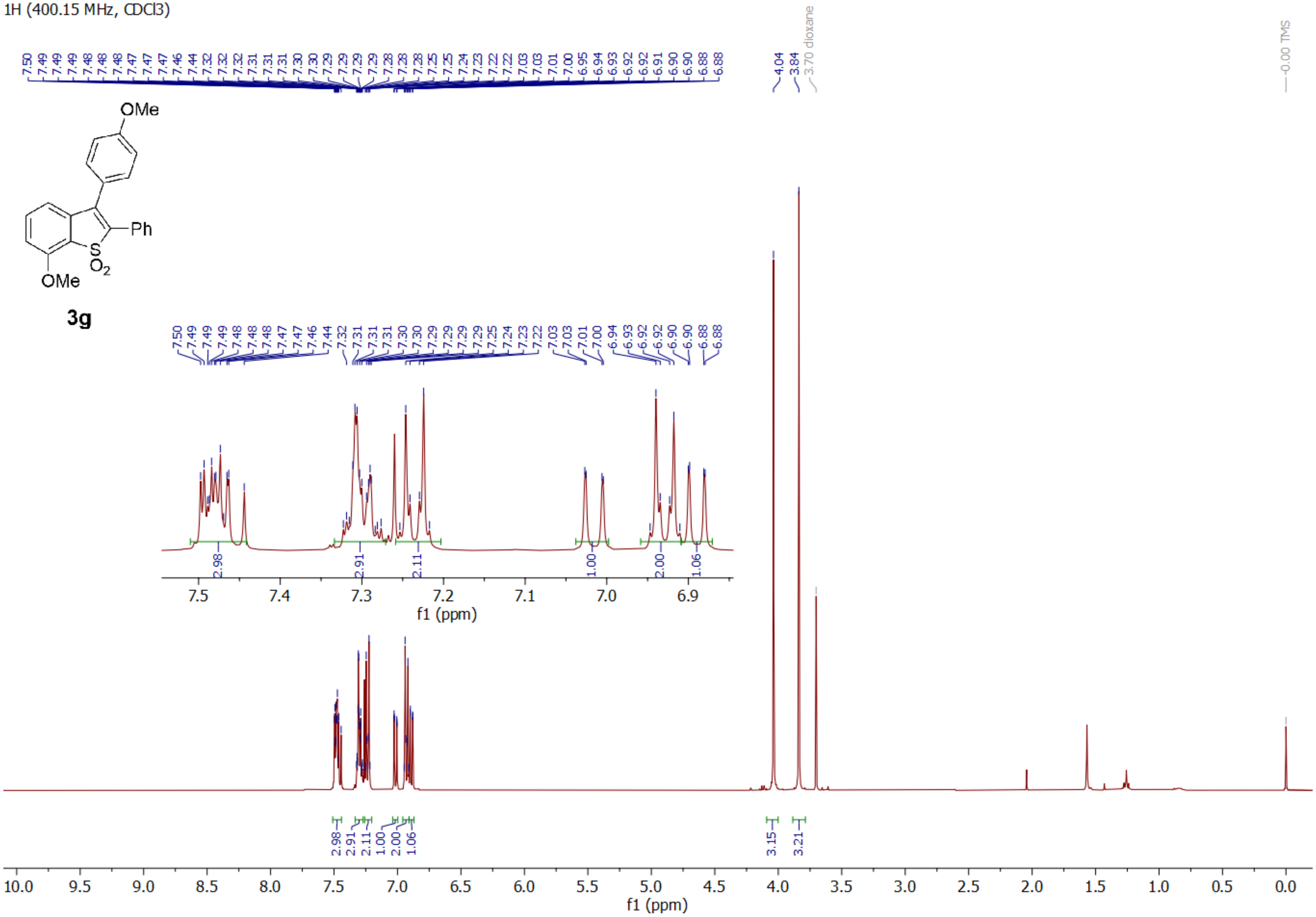

13C (100.63 MHz, CDCl3)

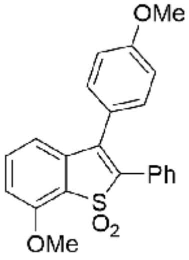

3g

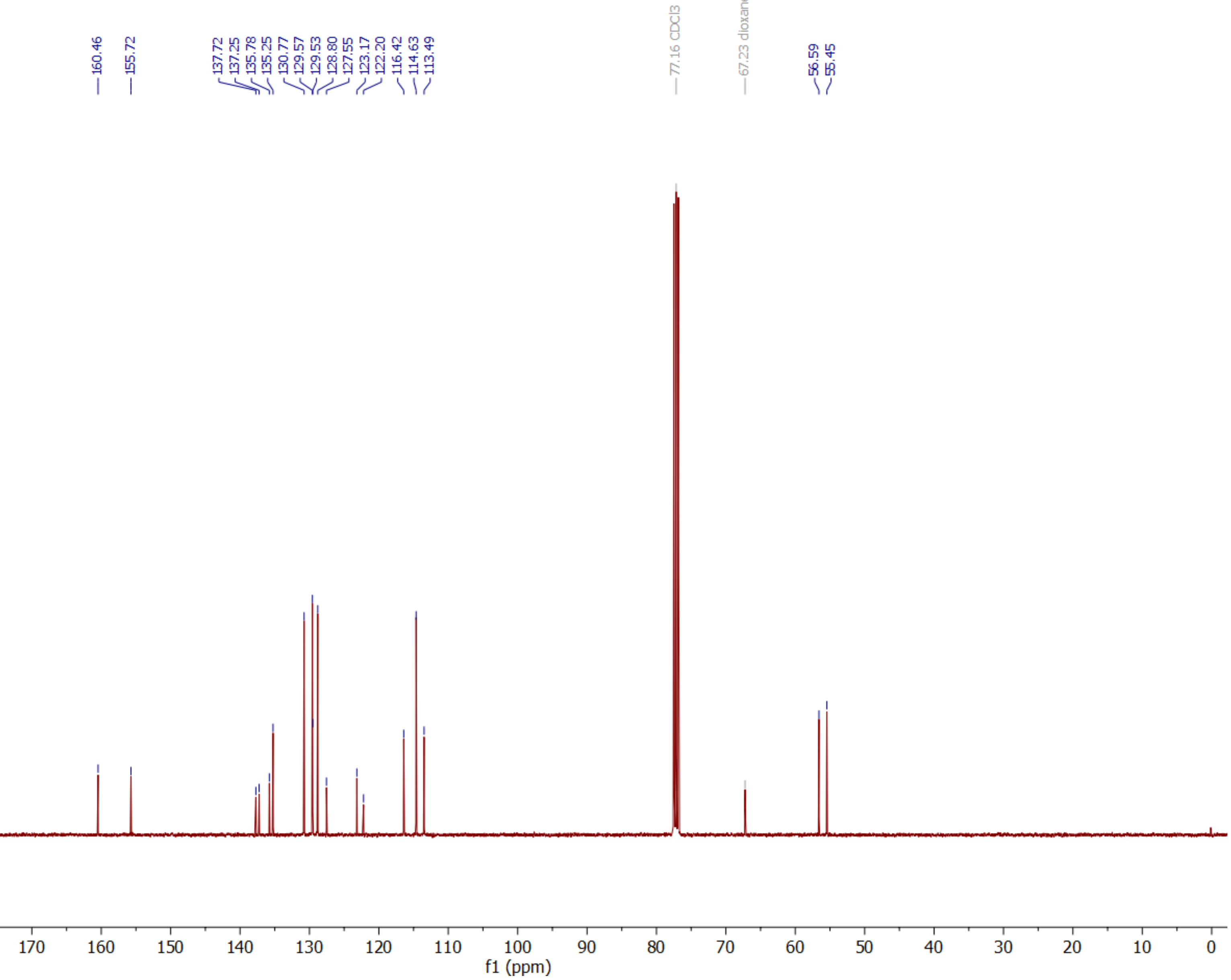

1H (400.15 MHz, CDCl3)

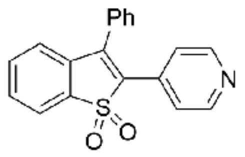

3h

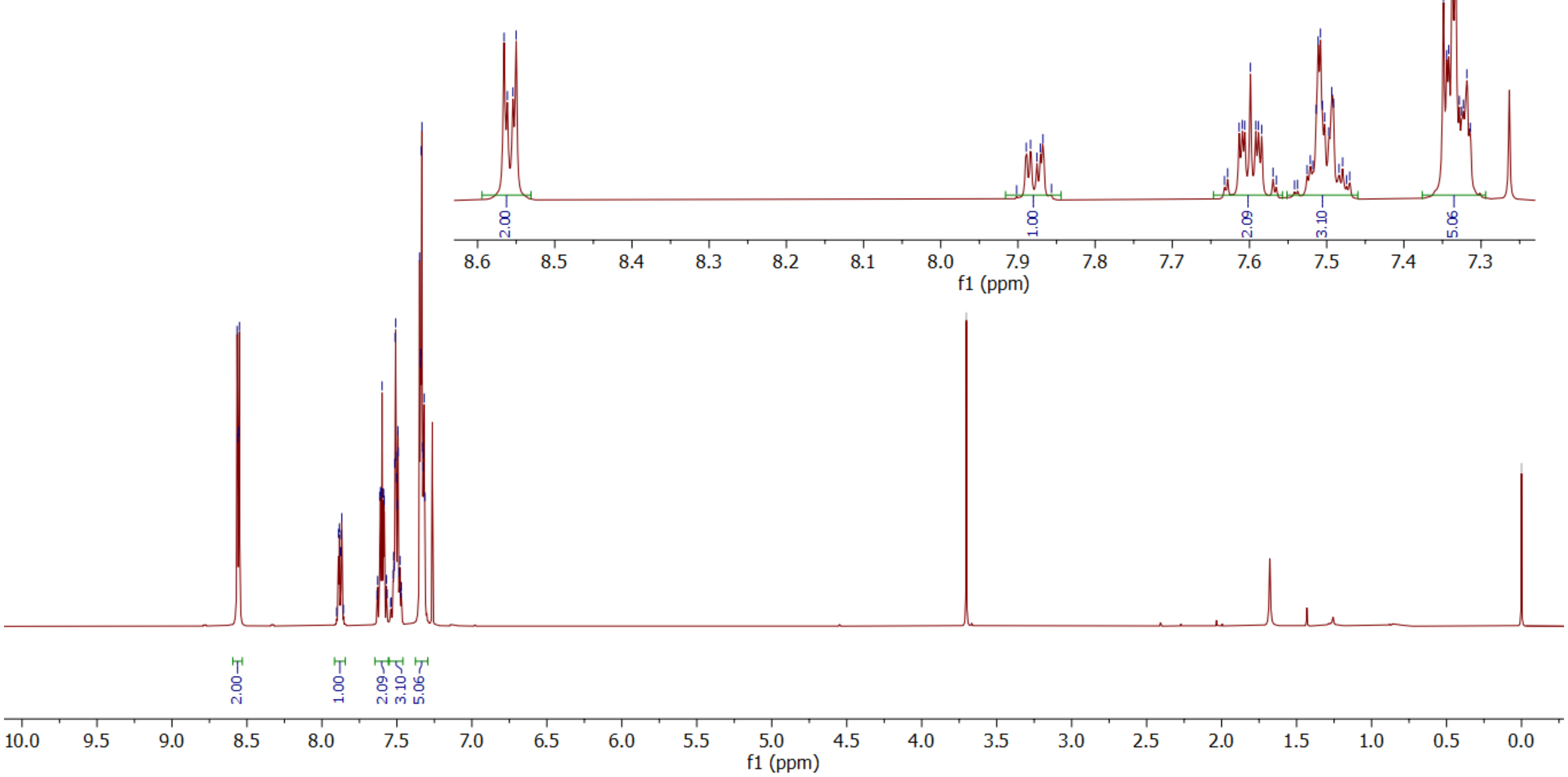

13C (100.63 MHz, CDCl3)

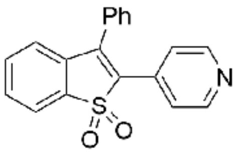

3h

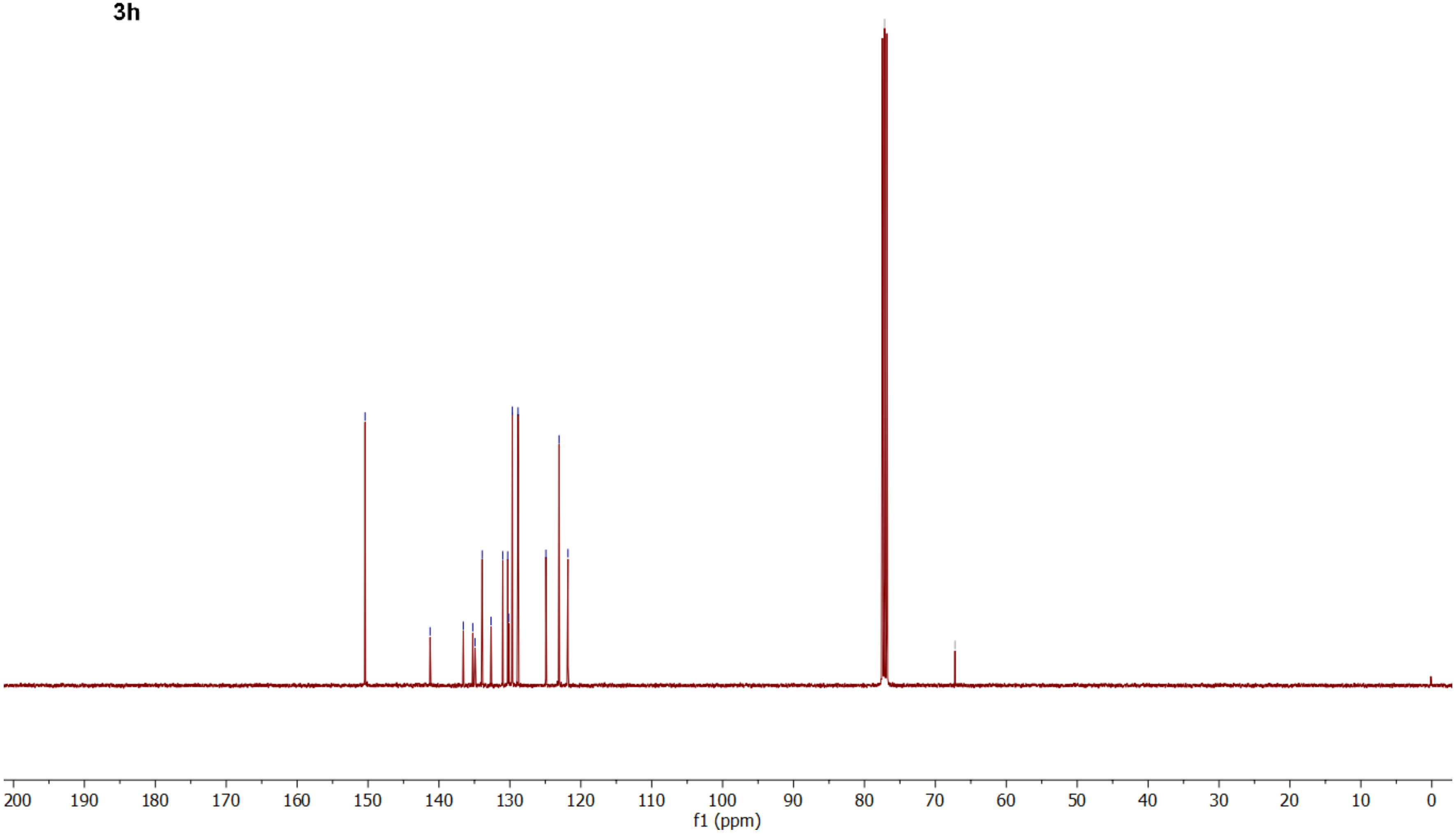

1H (400.15 MHz, CDCl3)

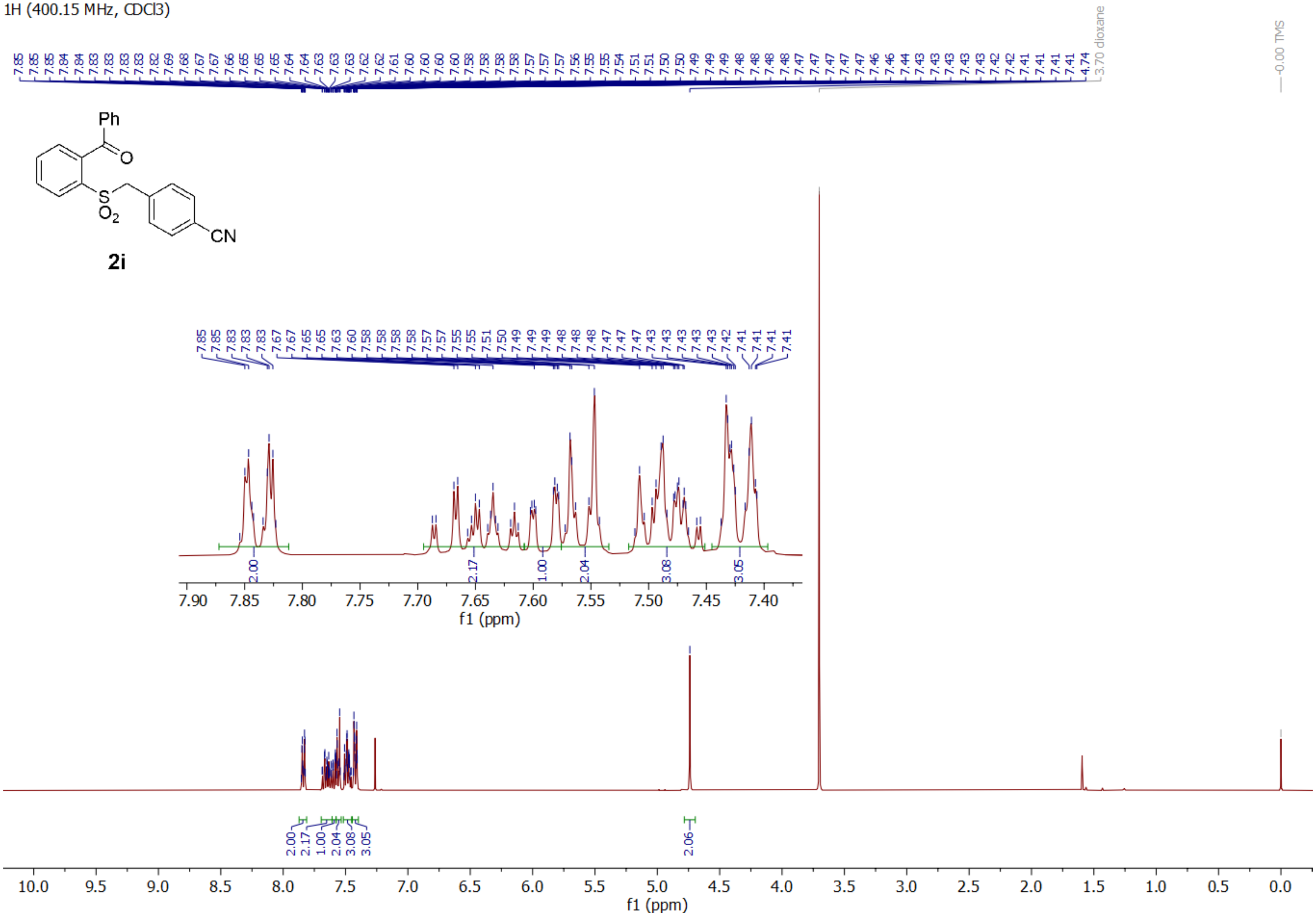

13C (100.63 MHz, CDCl3)

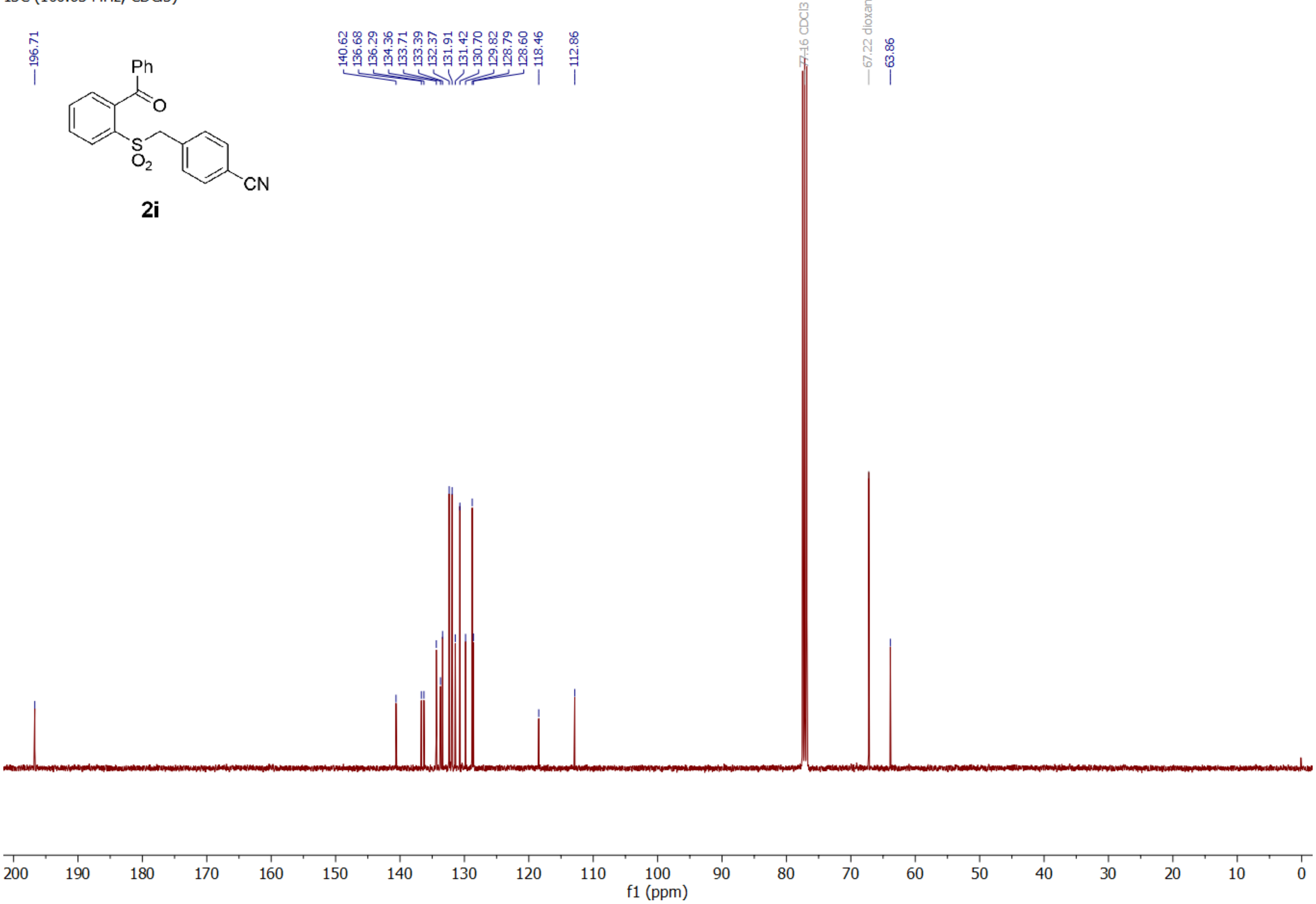

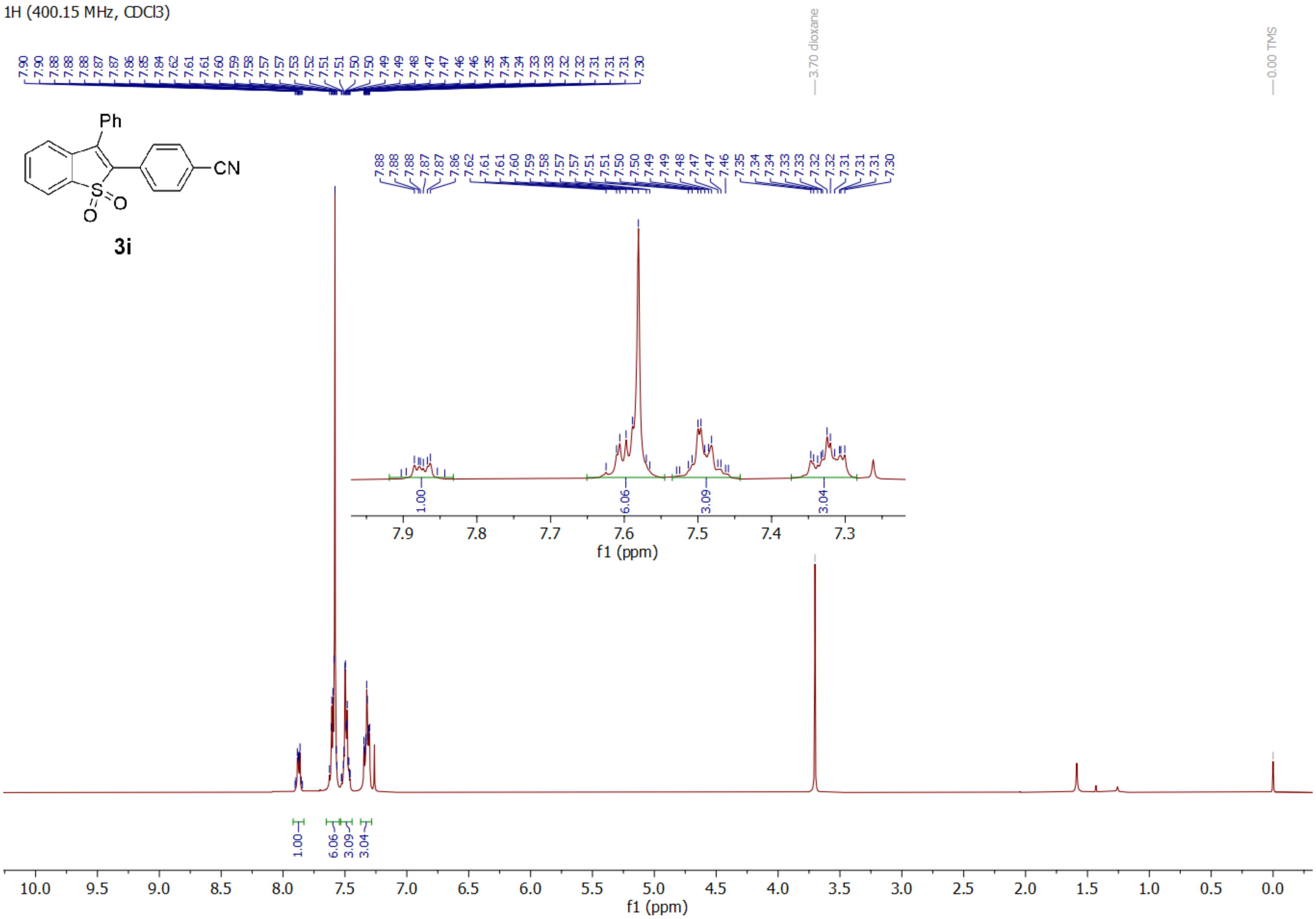

3i

13C (100.63 MHz, CDCl3)

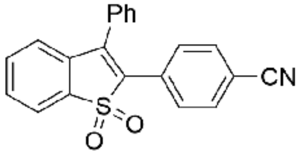

3i

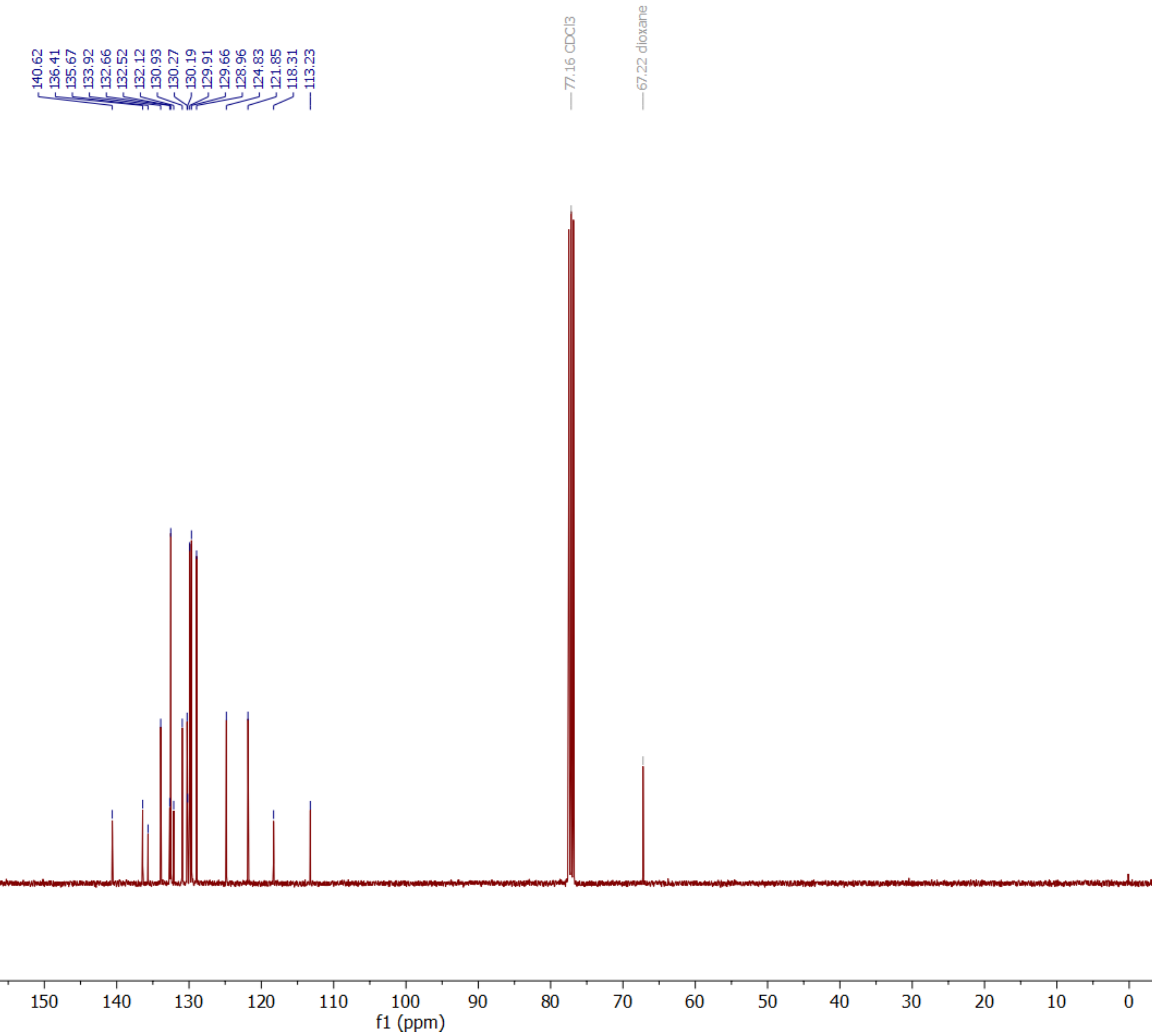

1H (400.15 MHz, CDCl3)

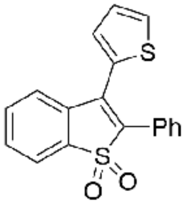

3j

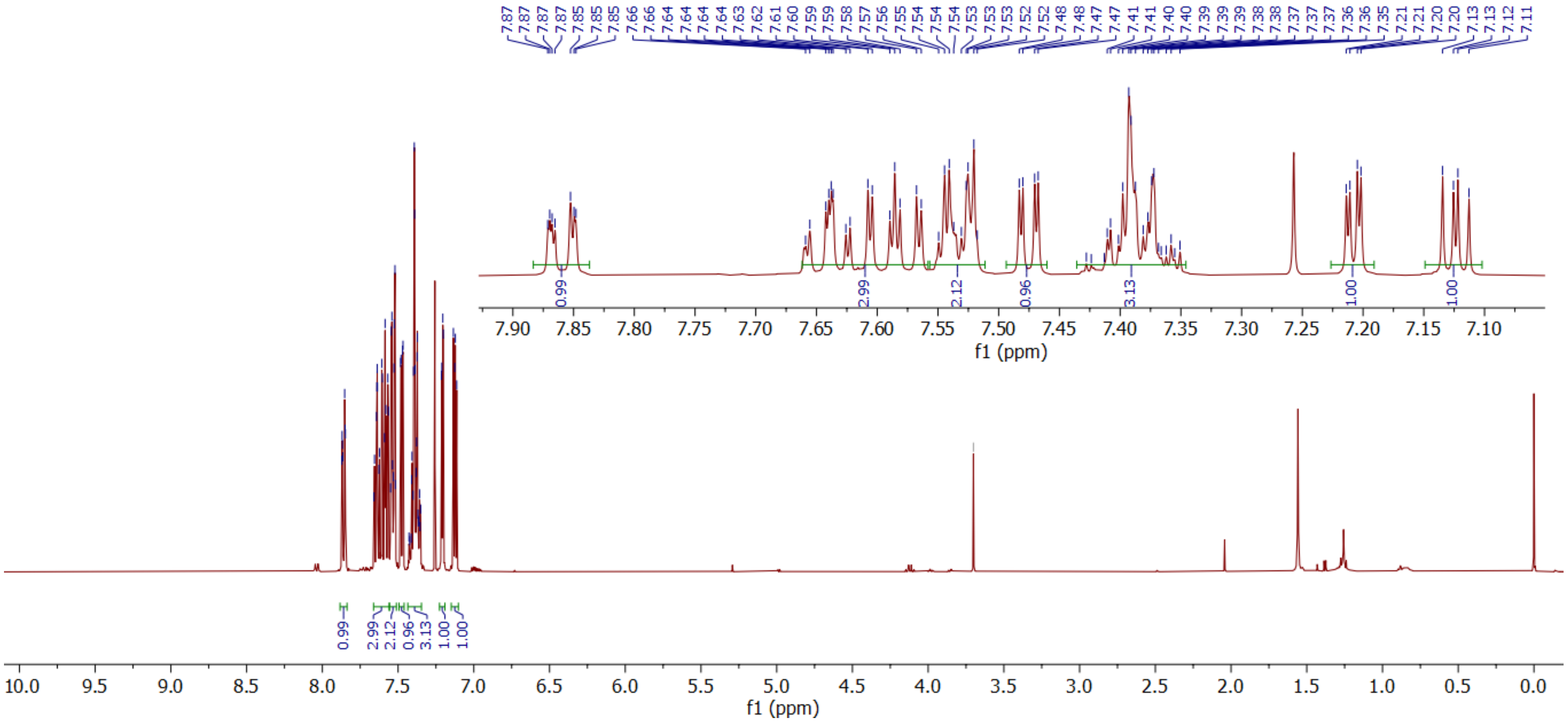

13C (100.63 MHz, CDCl3)

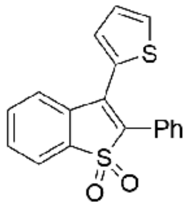

3j

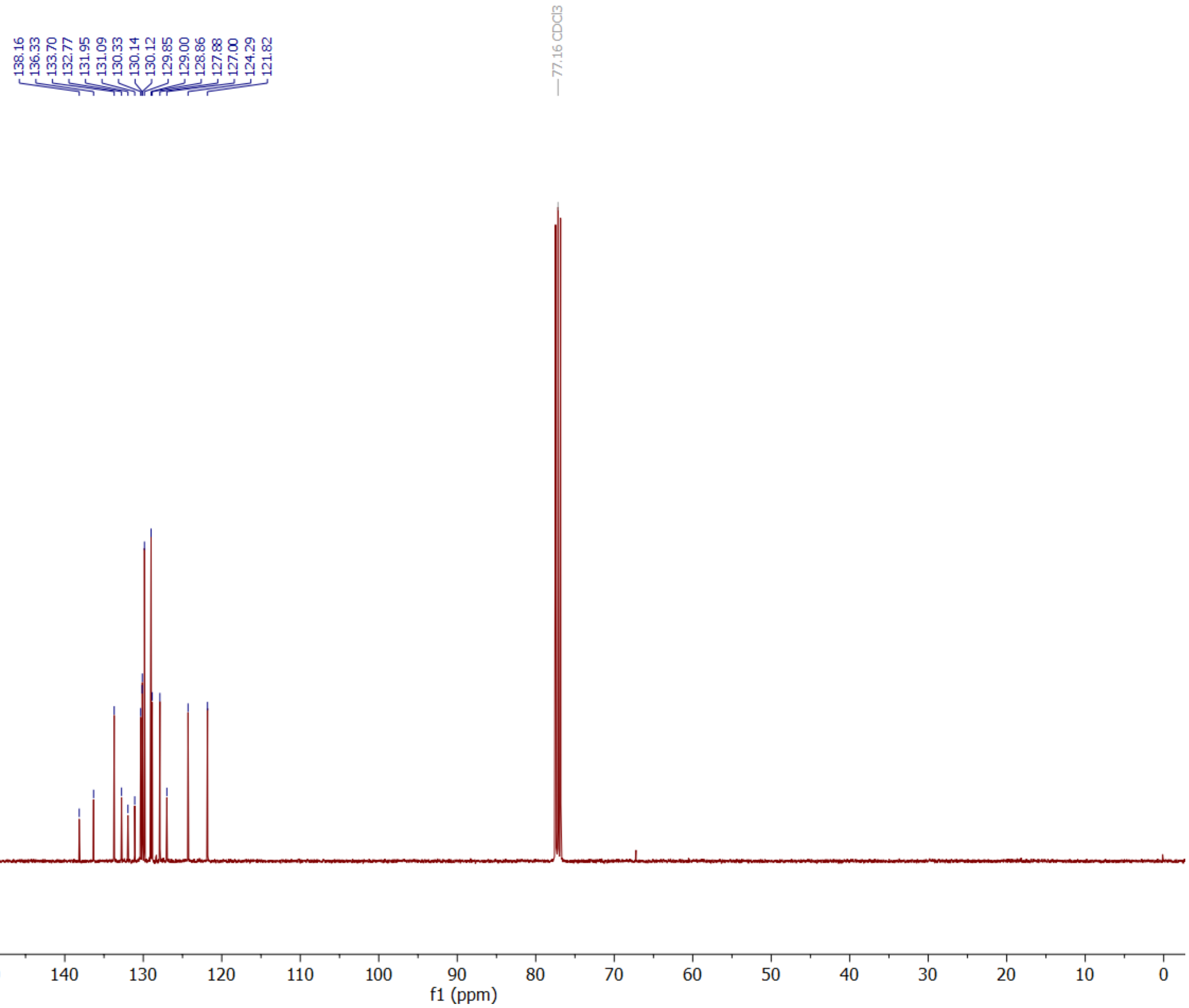

1H (400.15 MHz, CDCl3)

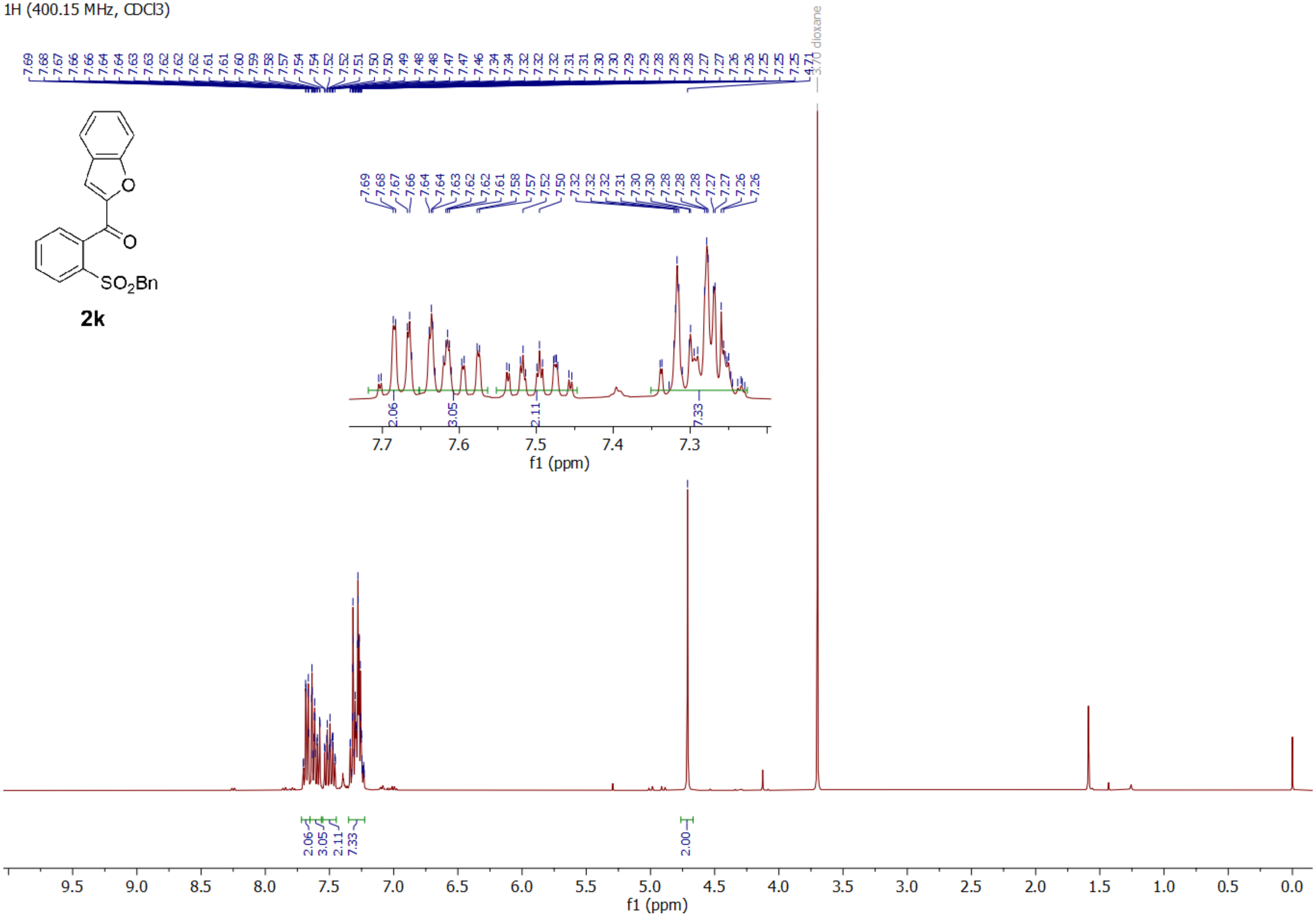

2k

13C (100.63 MHz, CDCl3)

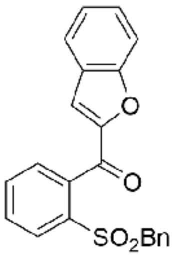

2k

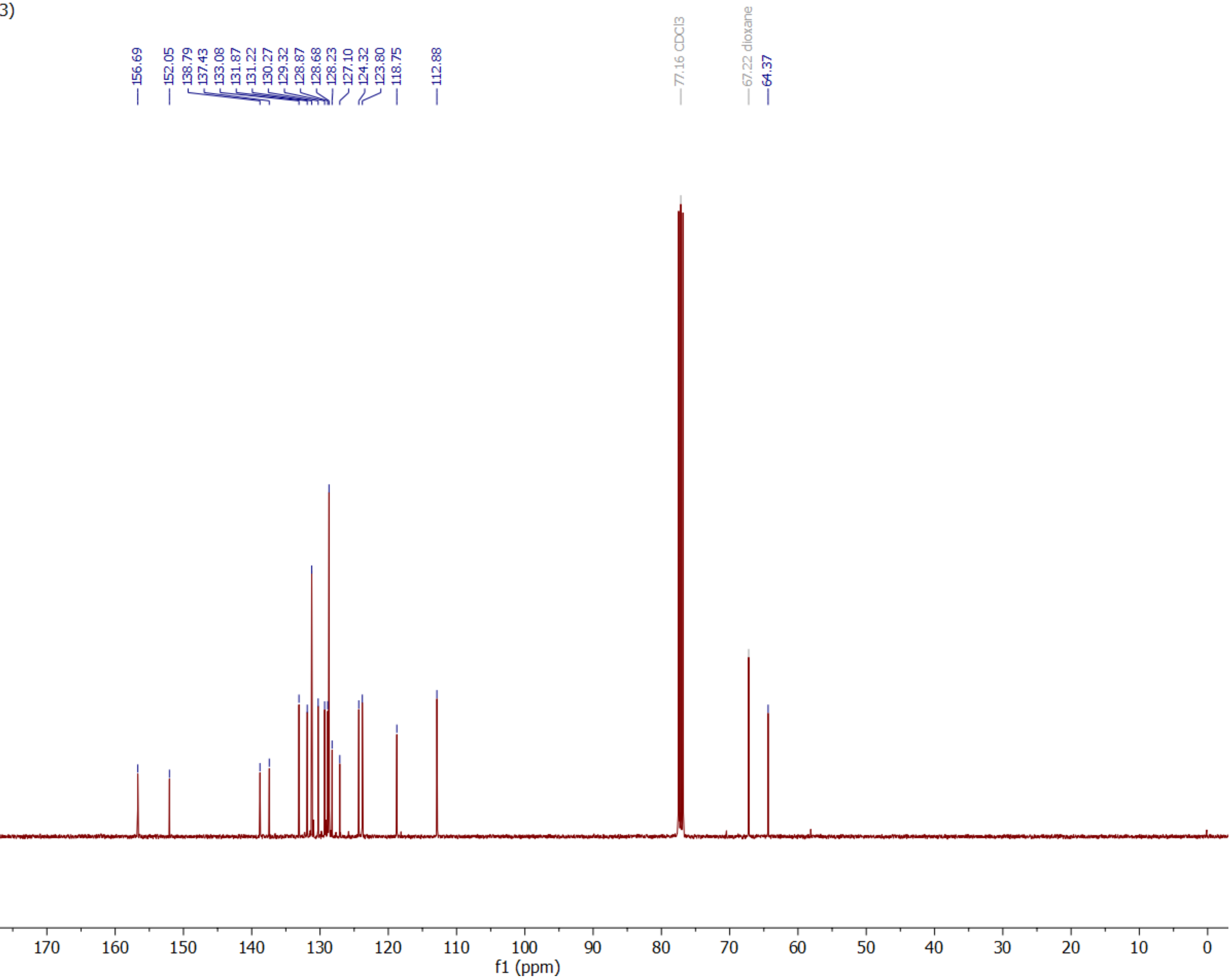

1H (400.15 MHz, CDCl3)

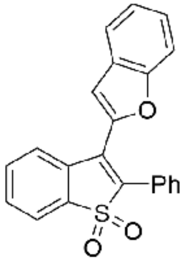

3k

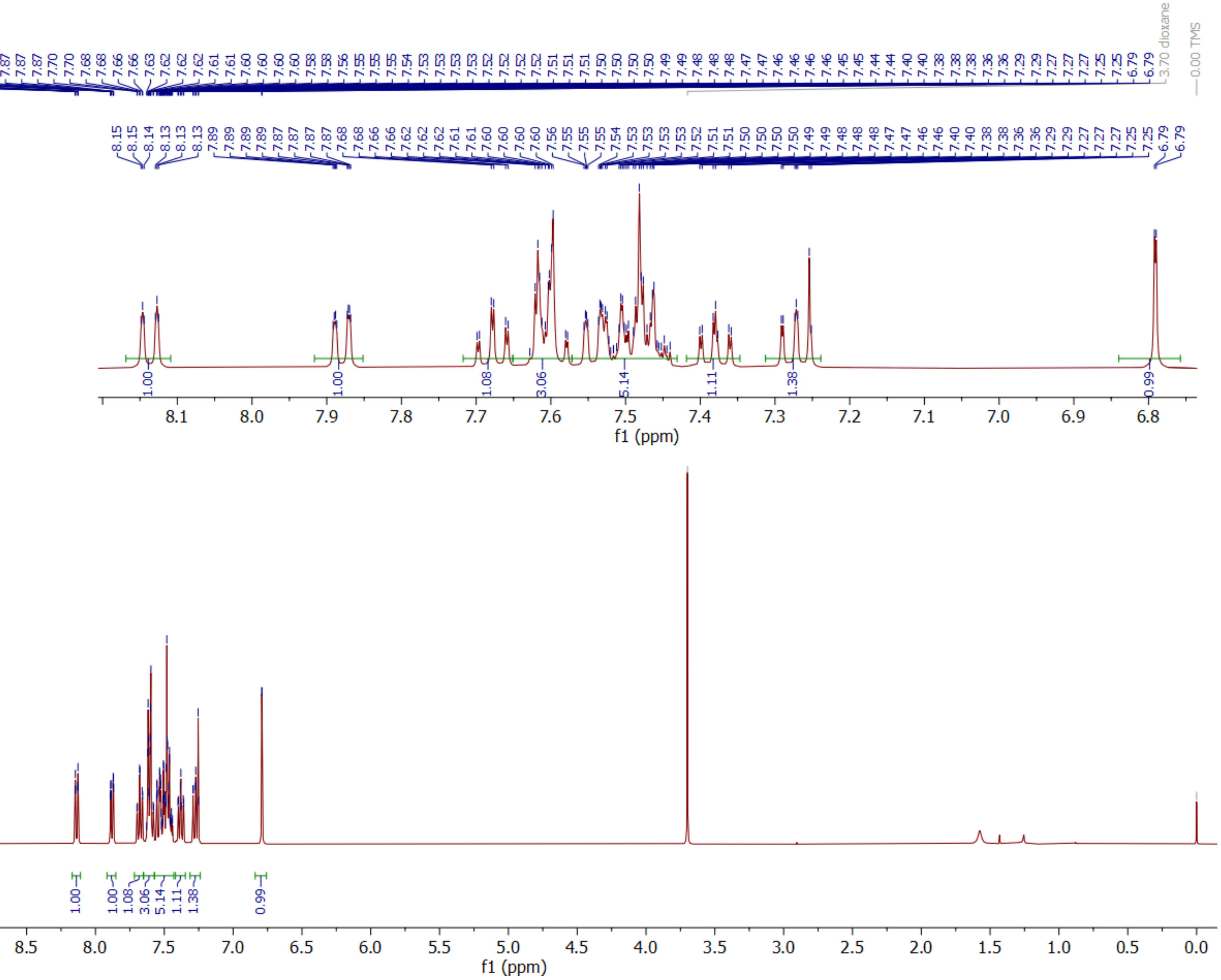

13C (100.63 MHz, CDCl3)

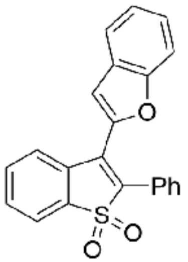

3k

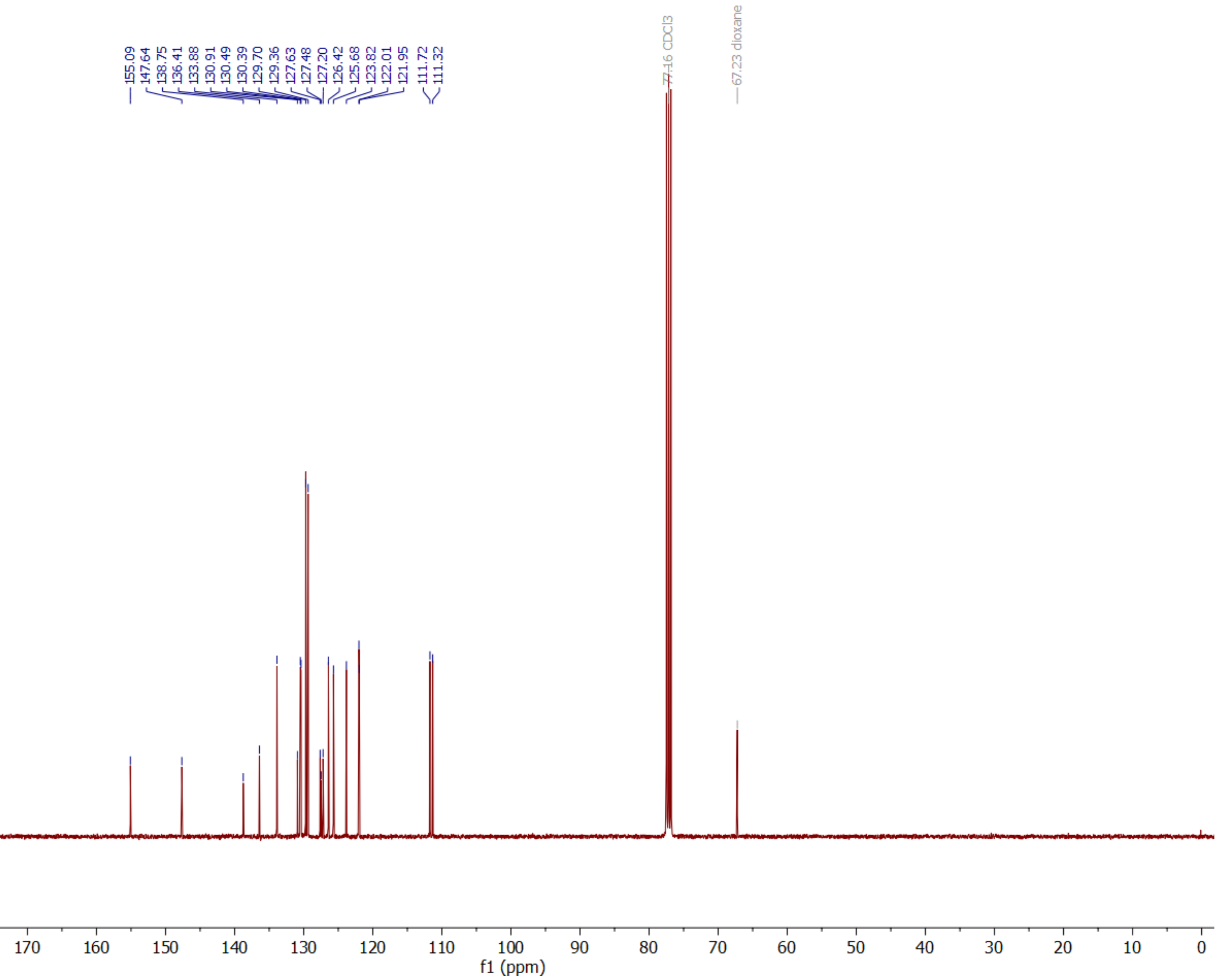

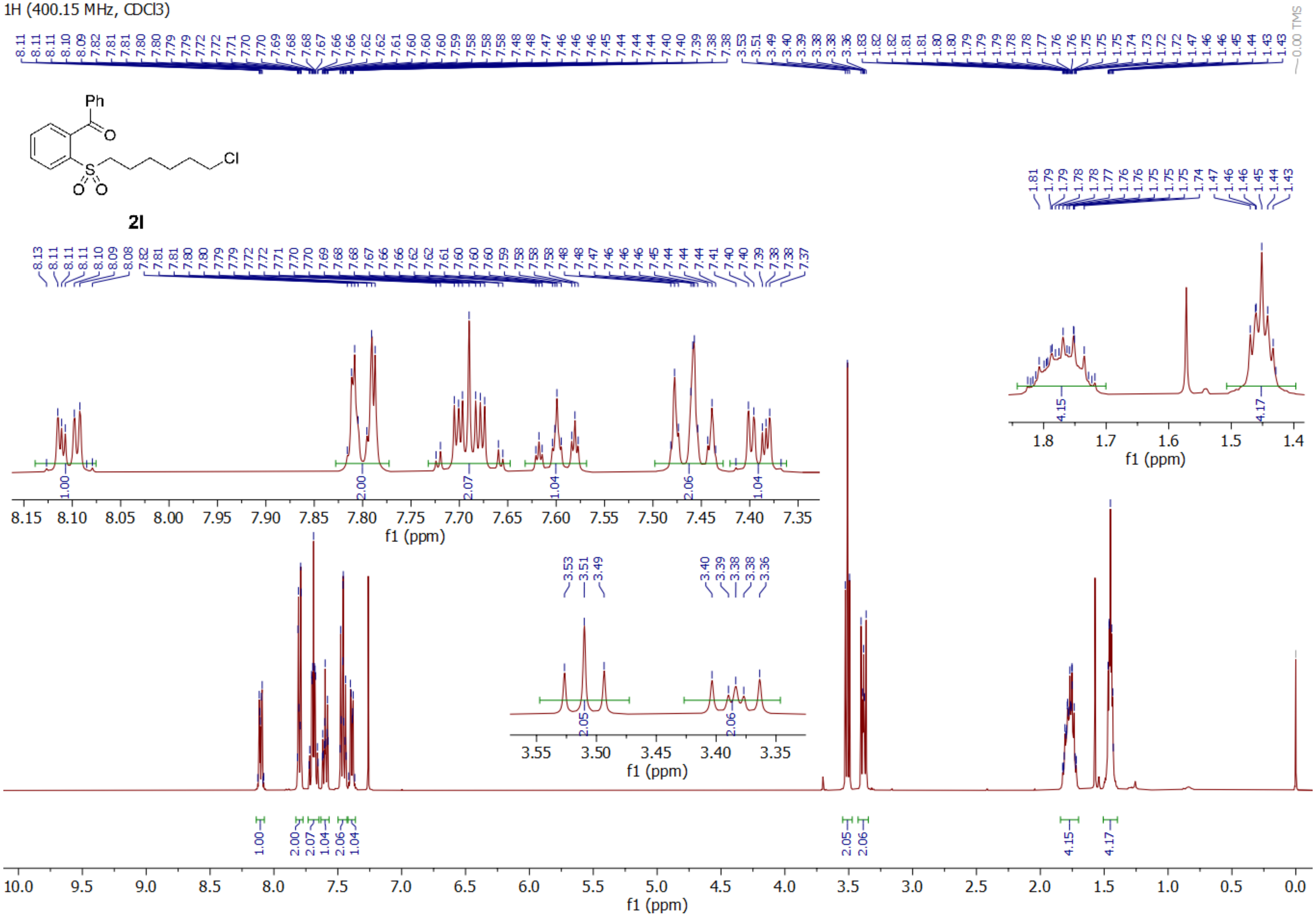

1H (400.15 MHz, CDCl3)
Ph
O
S
O O
Cl
2l
f1 (ppm)

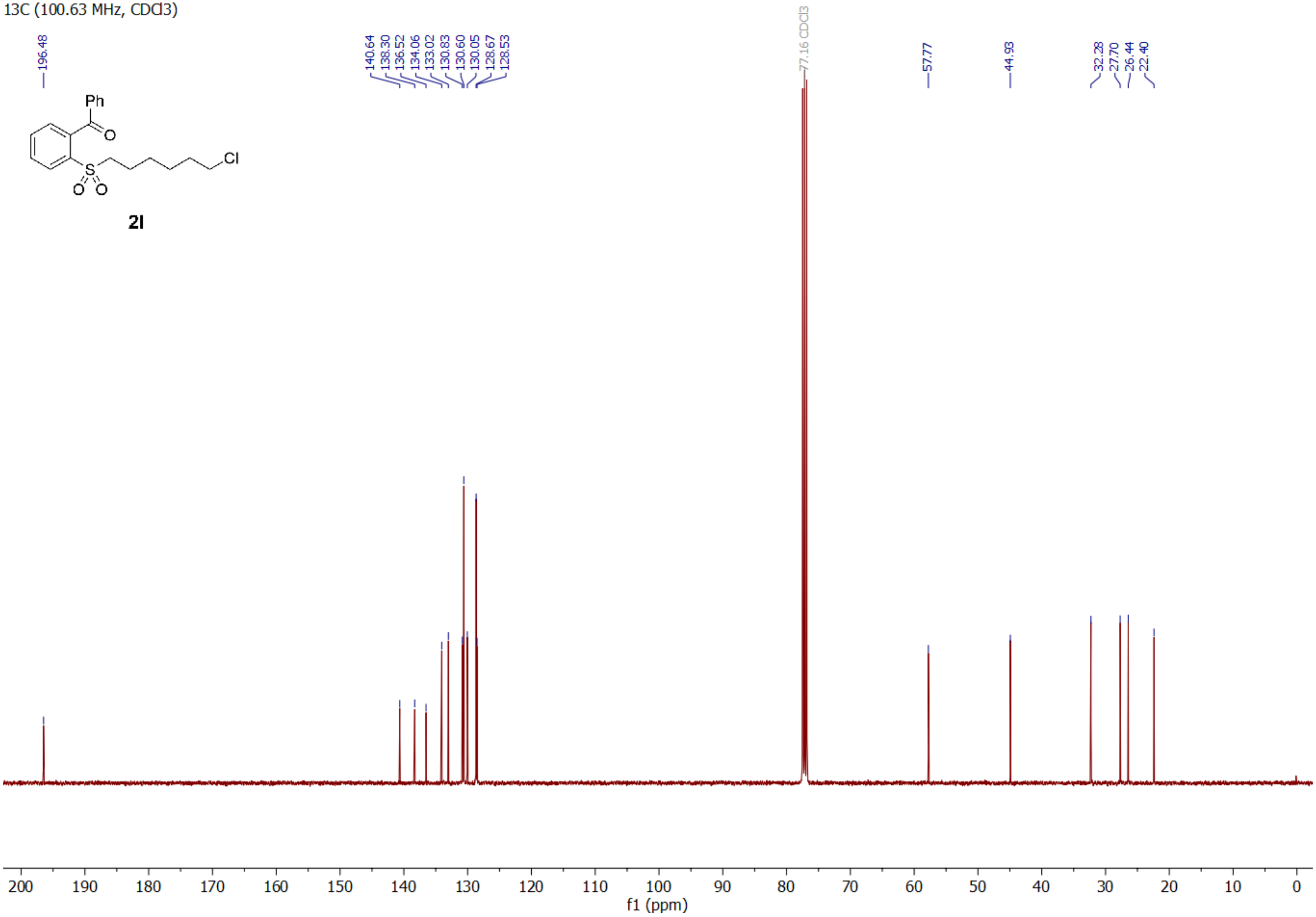
13C (100.63 MHz, CDCl3)
196.48
140.64
138.30
136.52
134.06
133.02
130.83
130.60
130.05
128.67
128.53
77.16 CDCl3
57.77
44.93
32.28
27.70
26.44
22.40
Ph
O
S
O
O
Cl
2l
200
190
180
170
160
150
140
130
120
110
100
90
80
70
60
50
40
30
20
10
0
f1 (ppm)

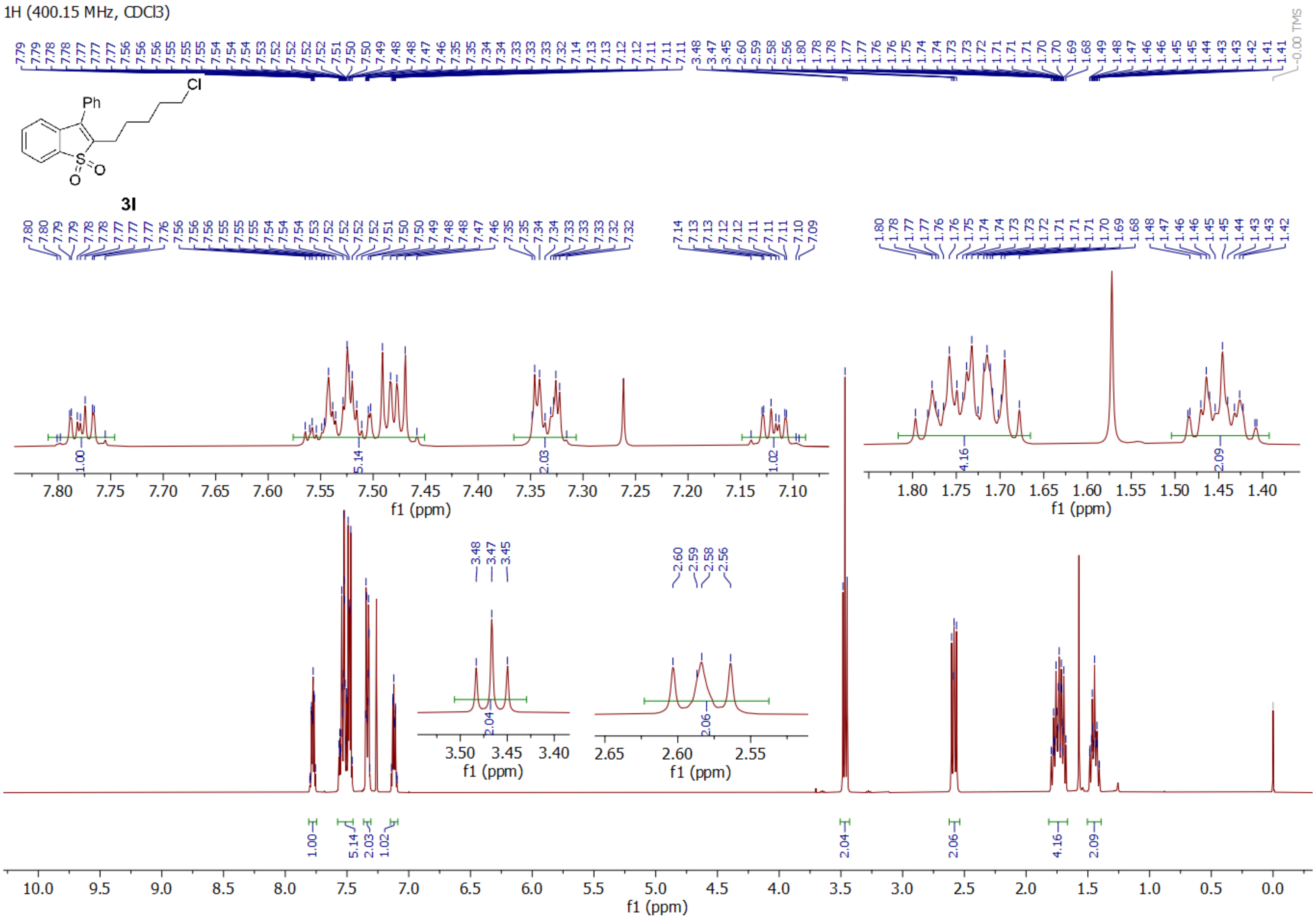
1H (400.15 MHz, CDCl3)
Ph
Cl
S
O
O
3l
f1 (ppm)
-0.00 TMS

13C (100.63 MHz, CDCl3)

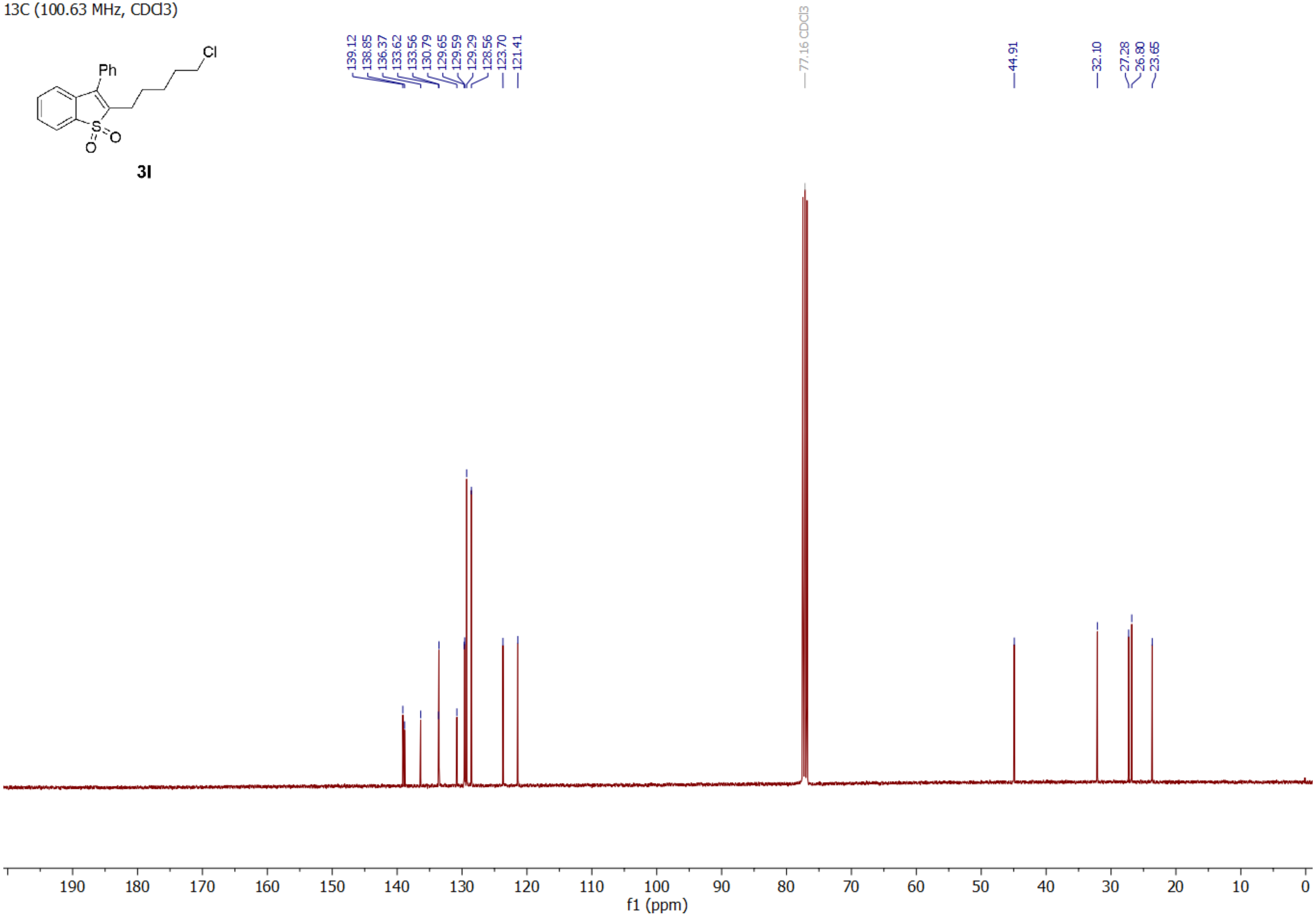

1H (400.15 MHz, CDCl3)

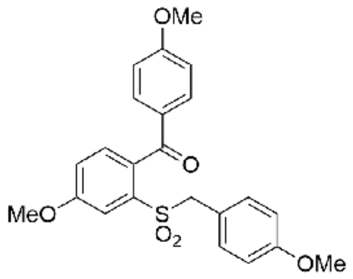

2m

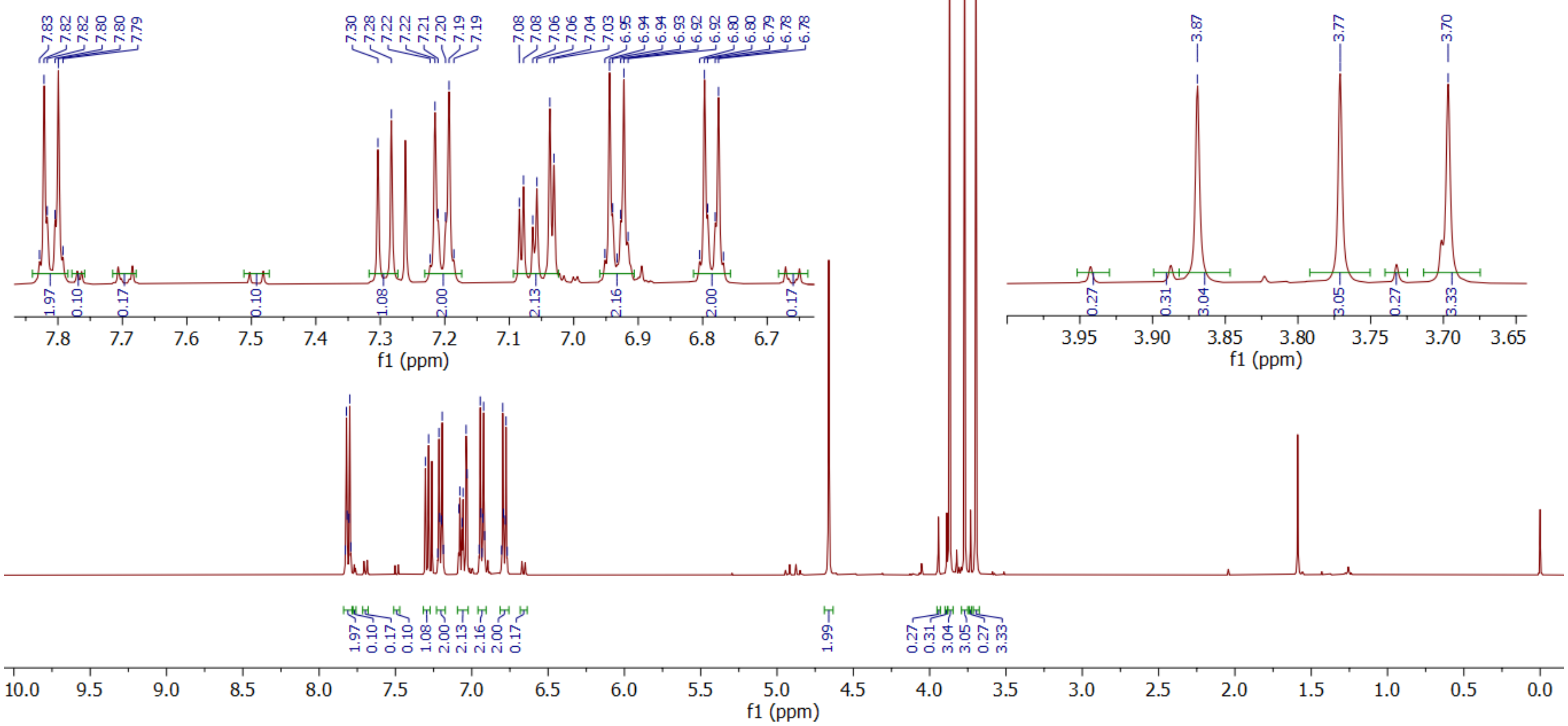

13C (100.63 MHz, CDCl3)

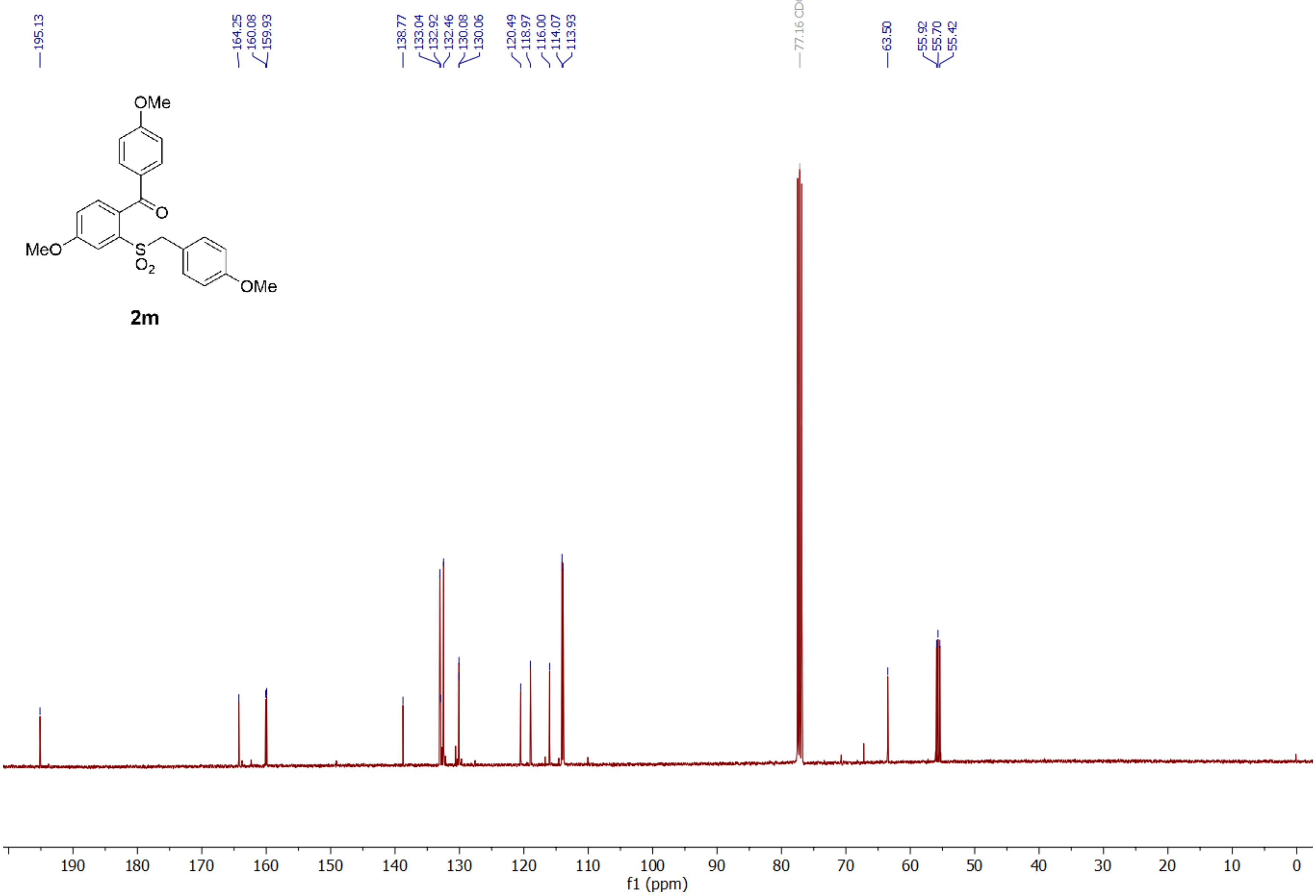

1H (400.15 MHz, CDCl3)

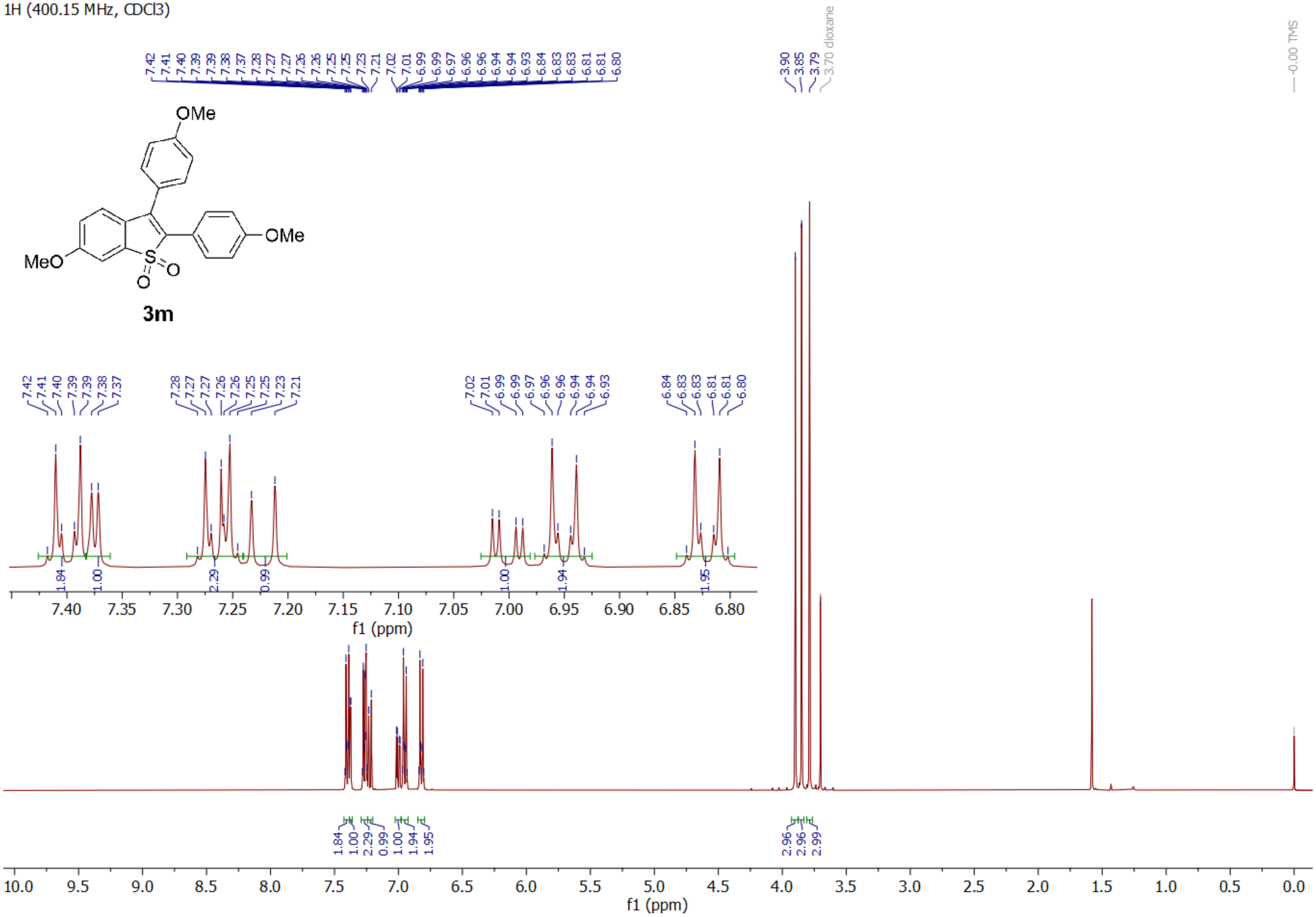

13C (100.63 MHz, CDCl3)

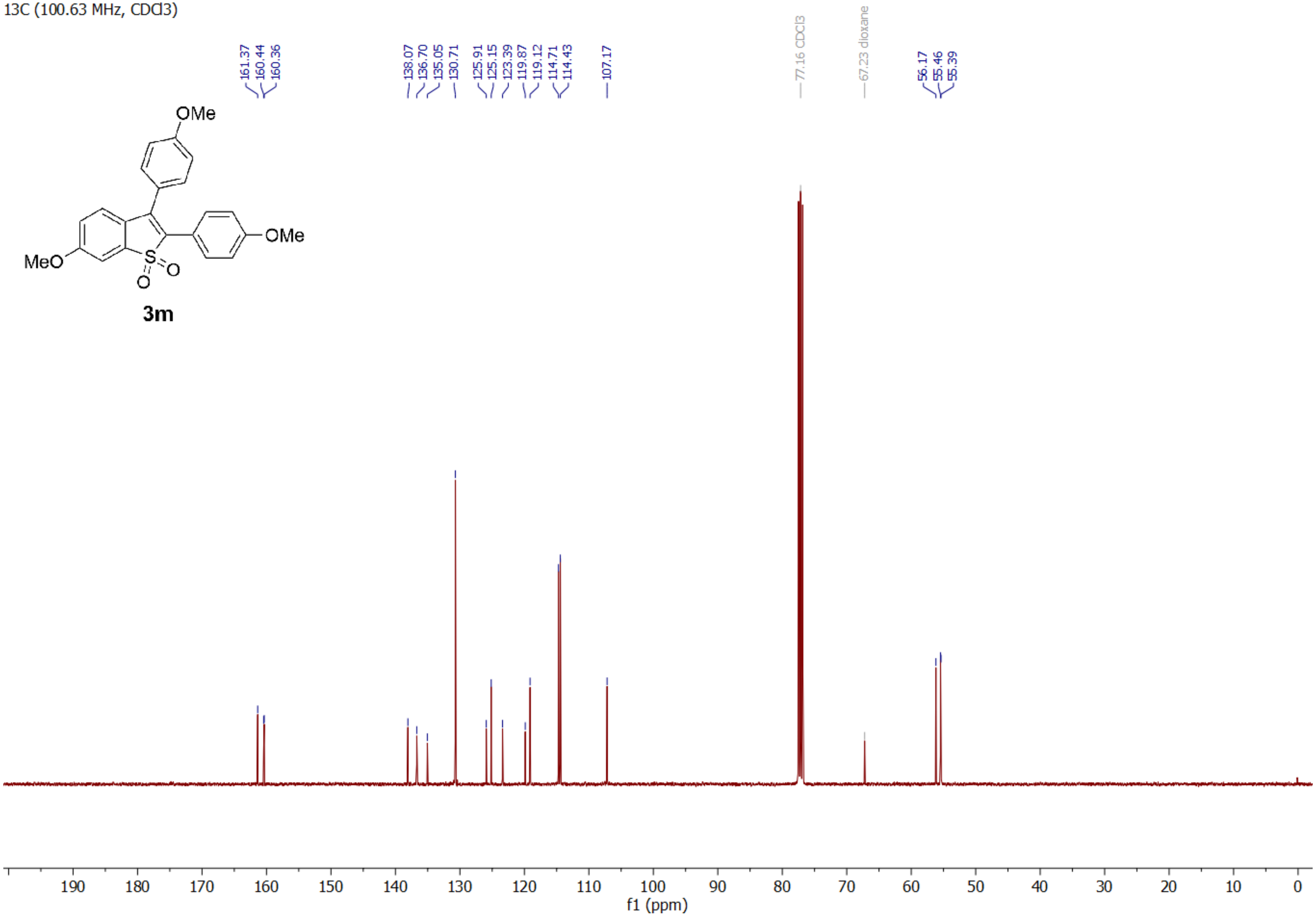

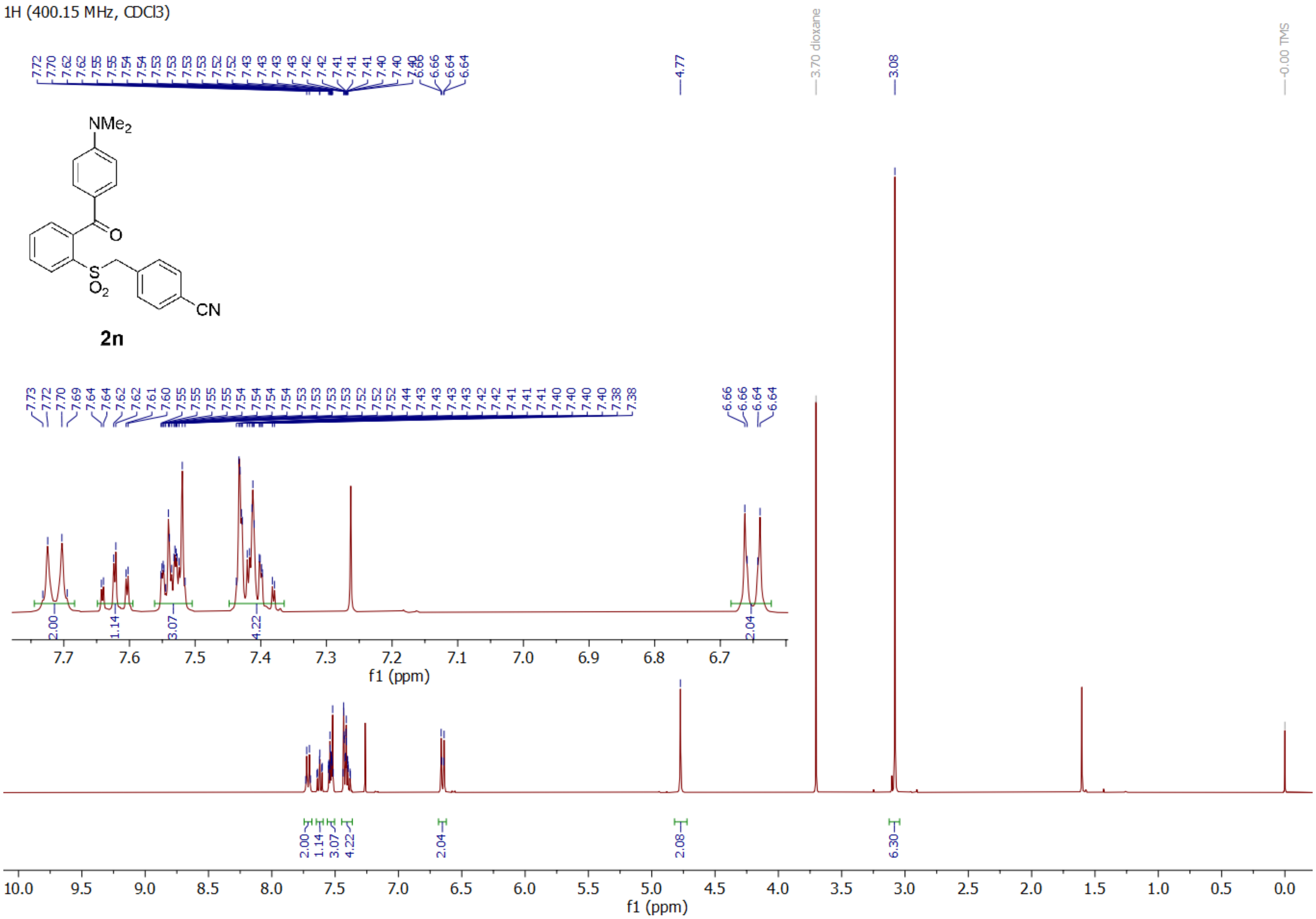
1H (400.15 MHz, CDCl3)
7.72 7.70 7.62 7.62 7.55 7.55 7.54 7.54 7.53 7.53 7.53 7.53 7.52 7.52 7.43 7.43 7.43 7.43 7.42 7.42 7.41 7.41 7.41 7.40 7.40 6.66 6.66 6.64 6.64
4.77
3.70 dioxane
3.08
0.00 TMS
NMe2
O
S
O2
CN
2n
7.73 7.72 7.70 7.69 7.64 7.64 7.62 7.62 7.61 7.60 7.55 7.55 7.55 7.55 7.54 7.54 7.54 7.54 7.53 7.53 7.53 7.53 7.52 7.52 7.52 7.44 7.43 7.43 7.43 7.43 7.42 7.42 7.41 7.41 7.41 7.40 7.40 7.40 7.40 7.38 7.38
6.66 6.66 6.64 6.64
2.00 1.14 3.07 4.22 2.04
f1 (ppm)
2.00 1.14 3.07 4.22 2.04 2.08 6.30
f1 (ppm)

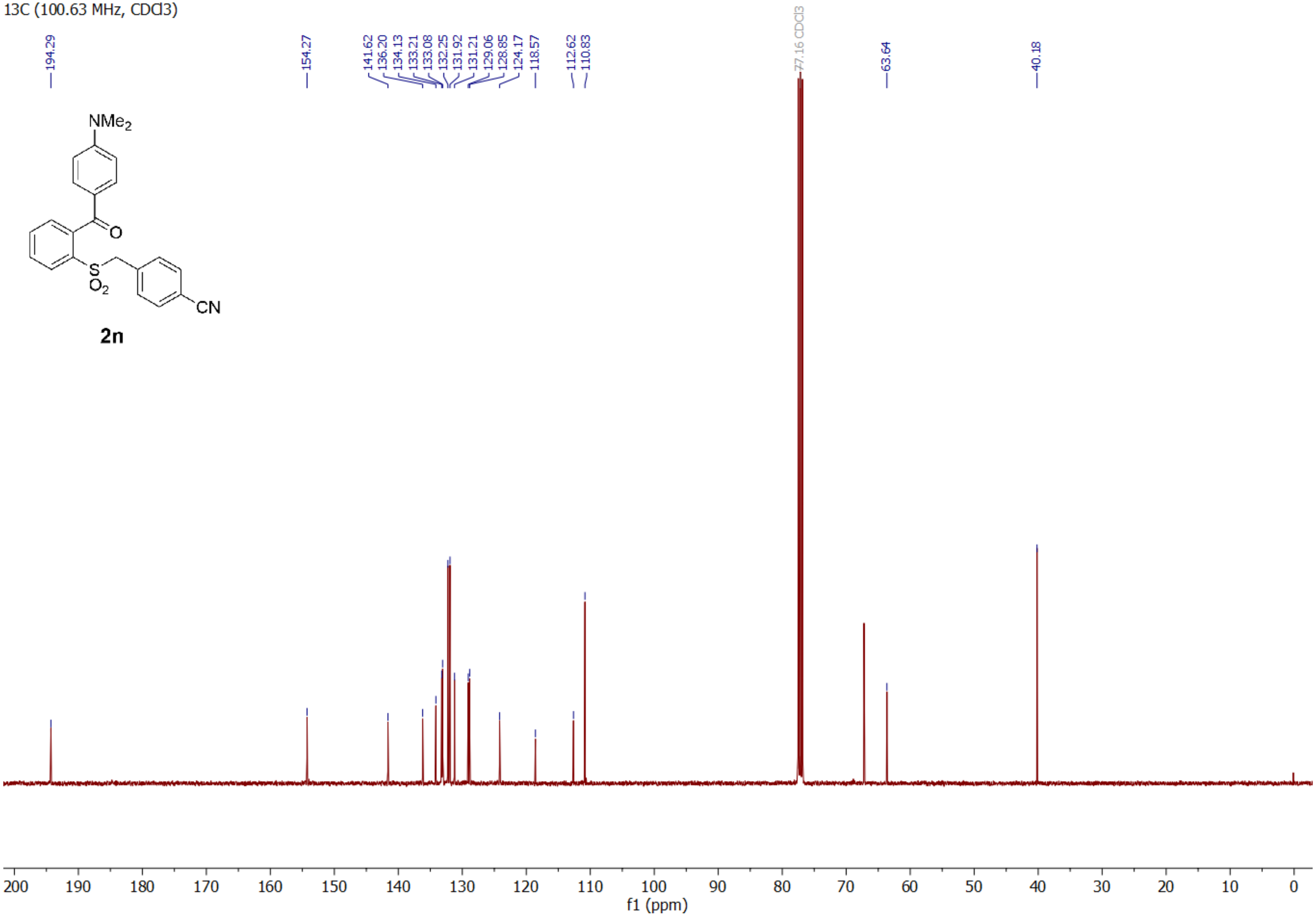

13C (100.63 MHz, CDCl3)
194.29
154.27
141.62
136.20
134.13
133.21
133.08
132.25
131.92
131.21
129.06
128.85
124.17
118.57
112.62
110.83
77.16 CDCl3
63.64
40.18
NMe2
O
S
O2
CN
200 190 180 170 160 150 140 130 120 110 100 90 80 70 60 50 40 30 20 10 0
f1 (ppm)

2n

1H (400.15 MHz, CDCl3)

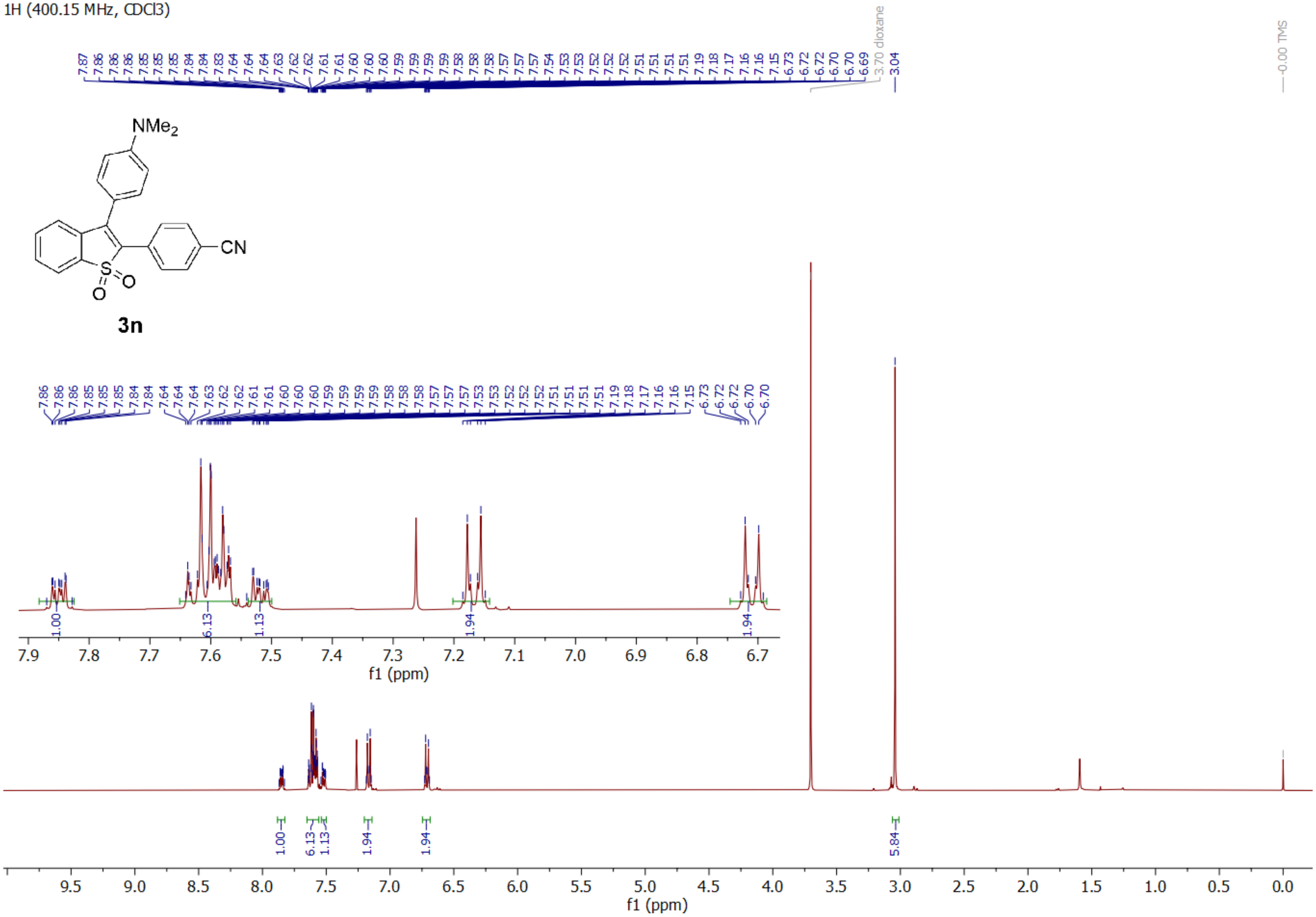

13C (100.63 MHz, CDCl3)

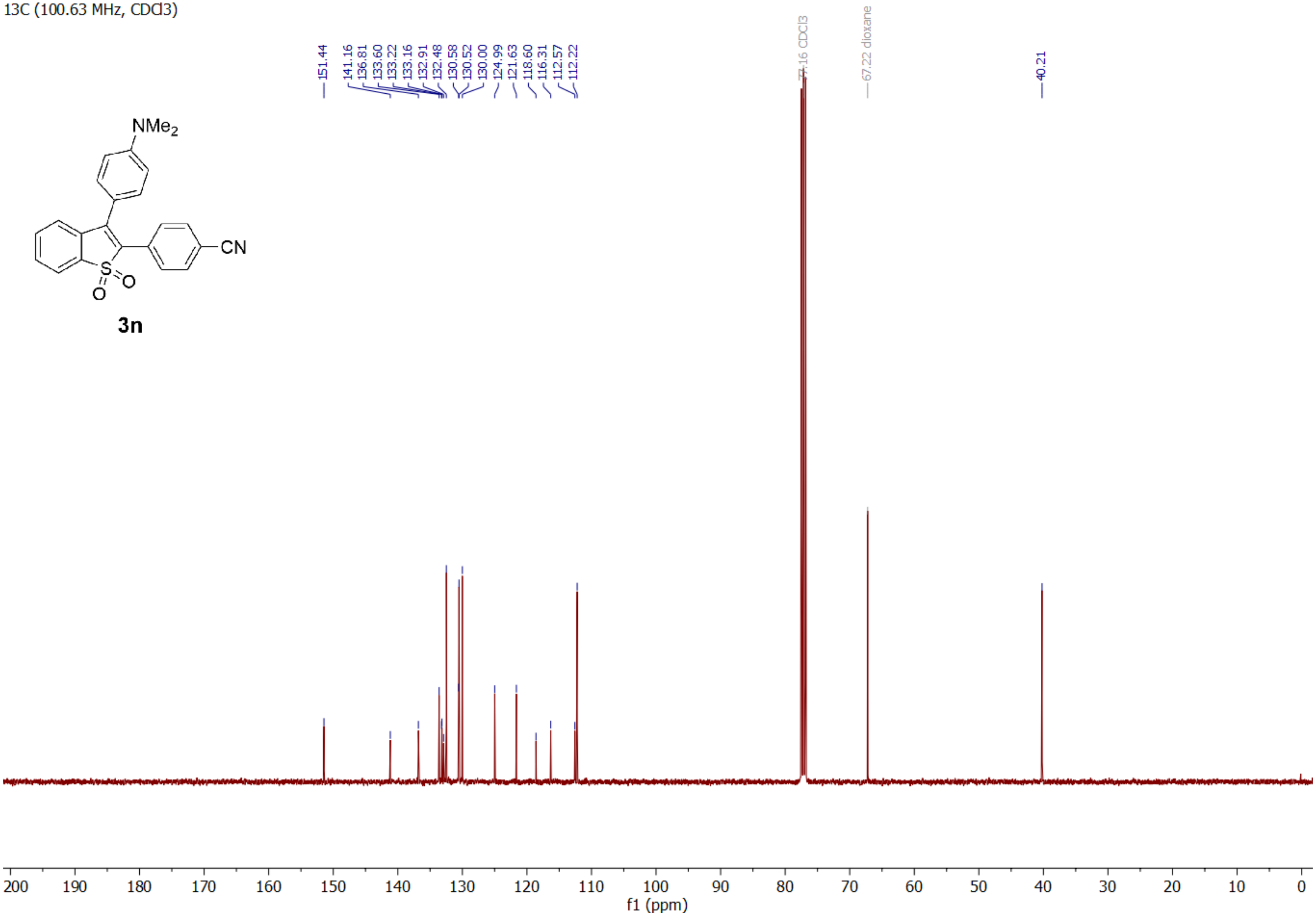

1H (400.15 MHz, CDCl3)

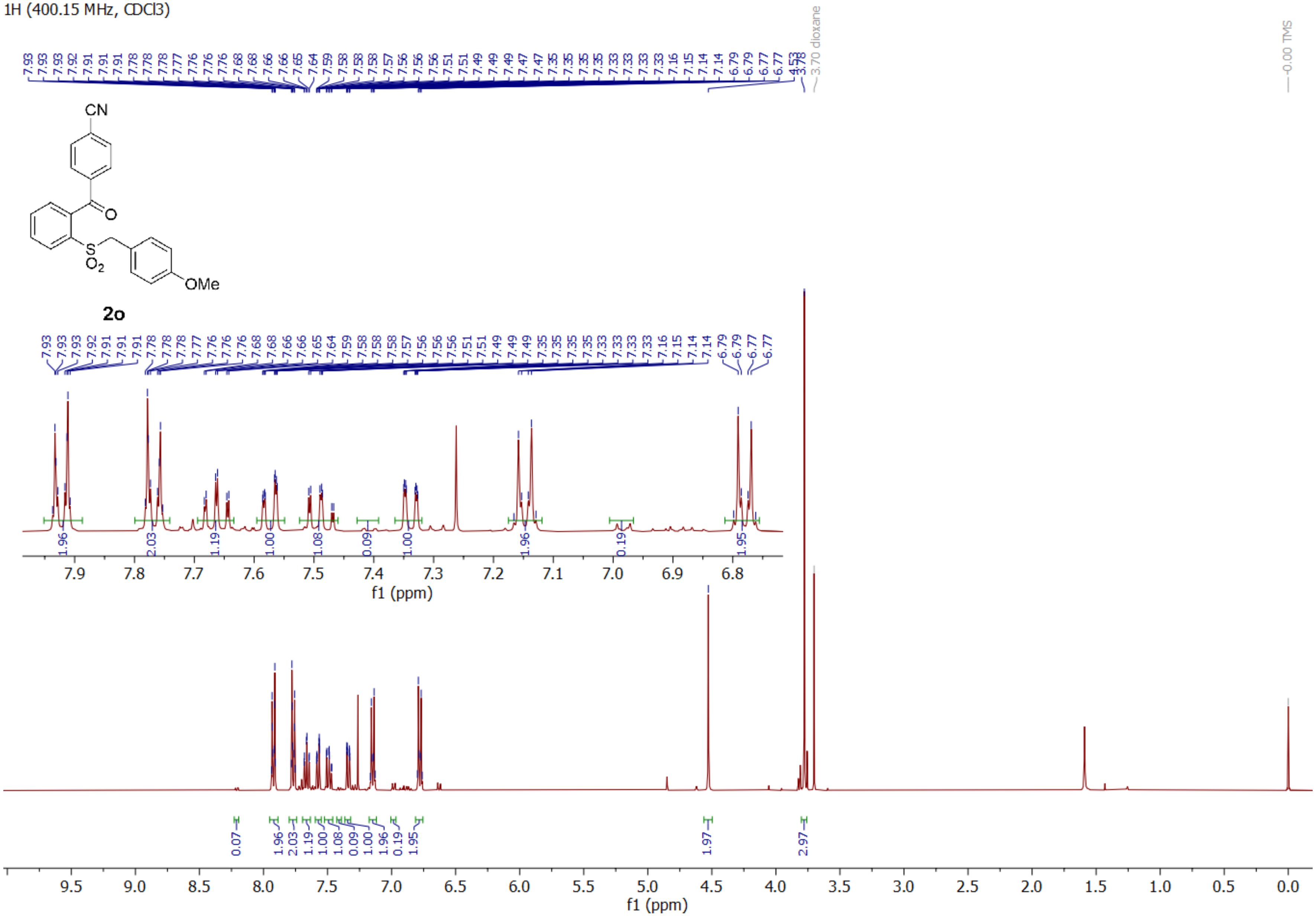

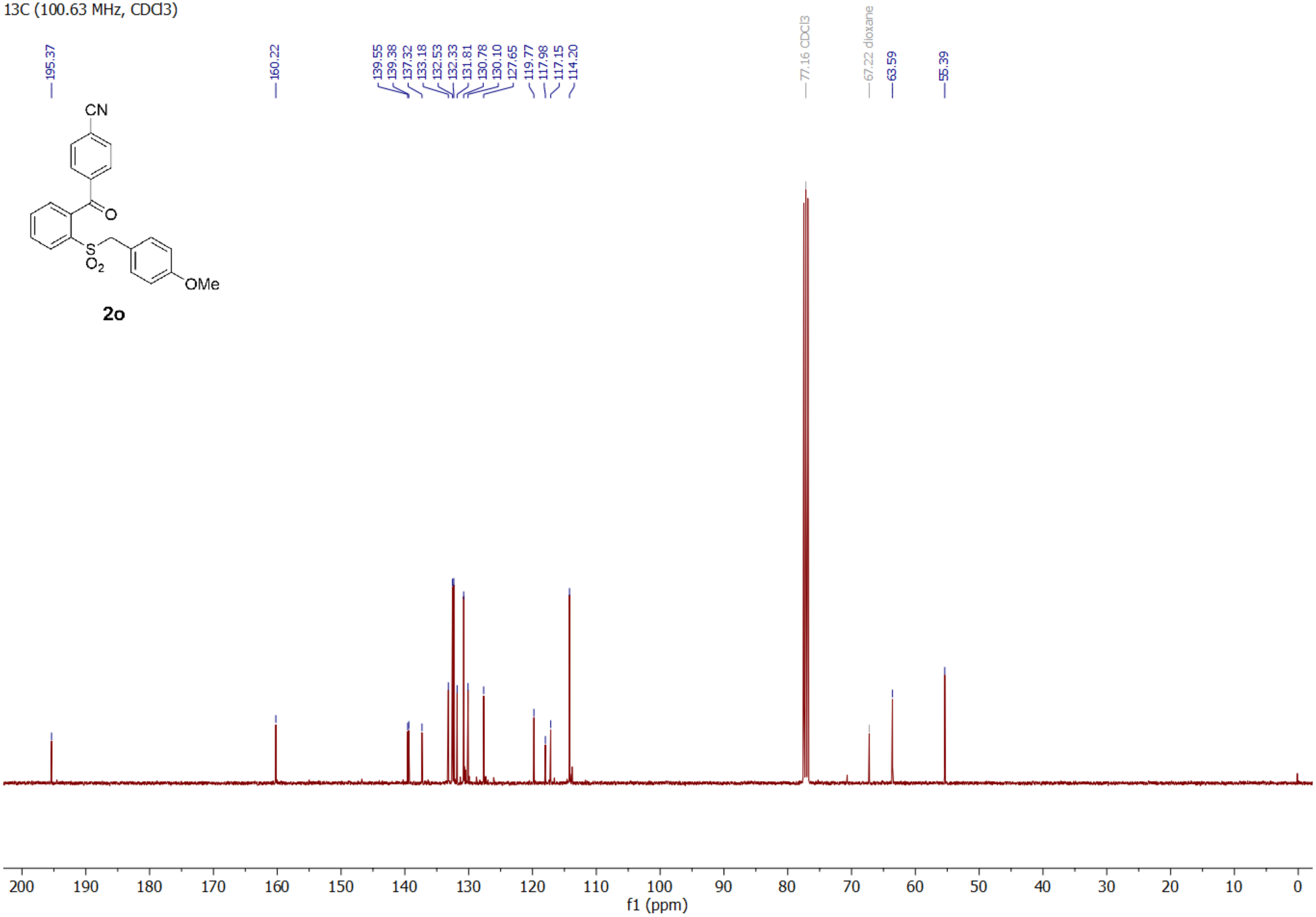
13C (100.63 MHz, CDCl3)
195.37
160.22
139.55
139.38
137.32
133.18
132.53
132.33
131.81
130.78
130.10
127.65
119.77
117.98
117.15
114.20
77.16 CDCl3
67.22 dioxane
63.59
55.39
CN
O
S
O2
OMe
2o
f1 (ppm)

1H (400.15 MHz, CDCl3)

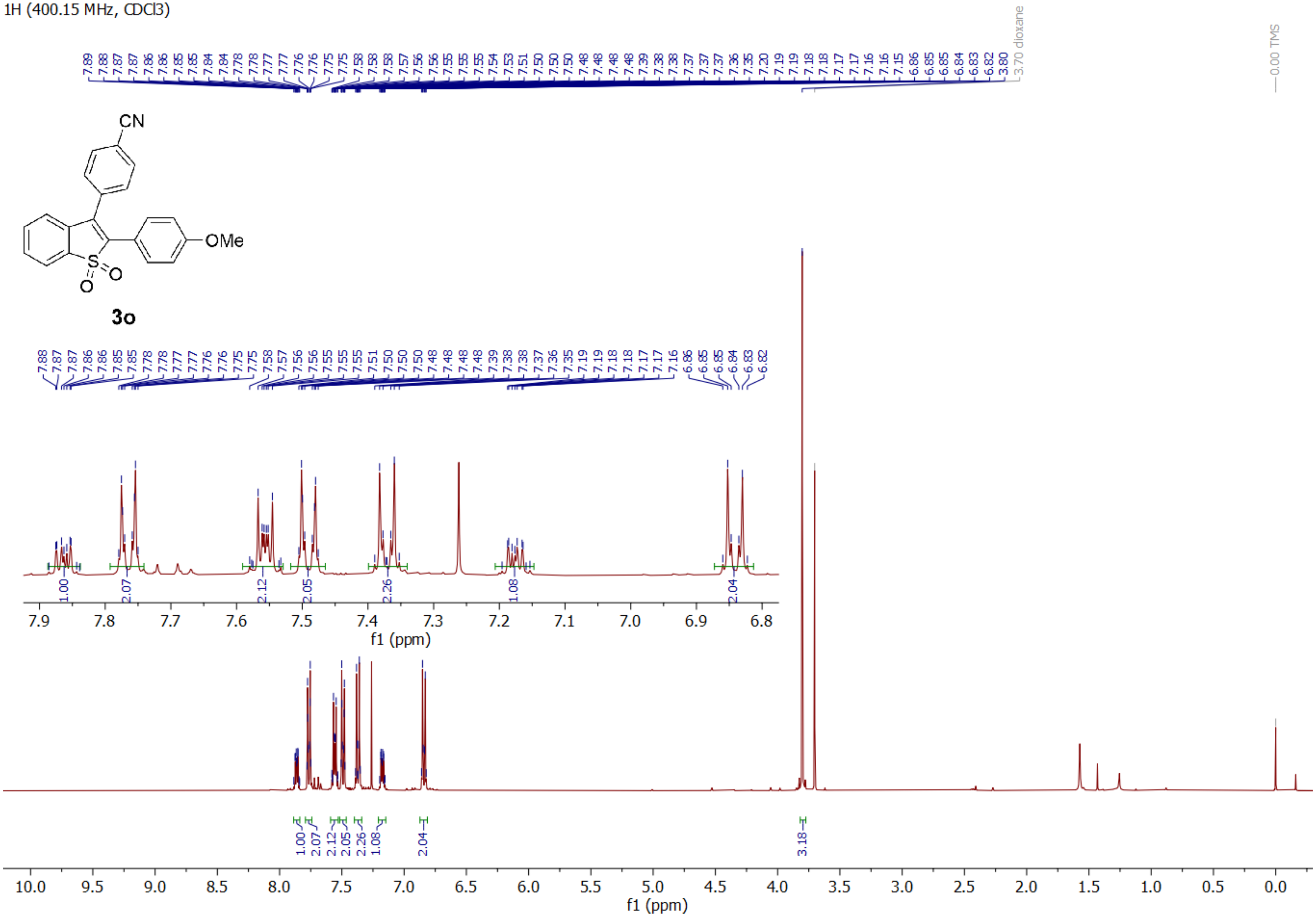

13C (100.63 MHz, CDCl3)

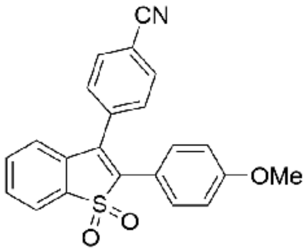

3o

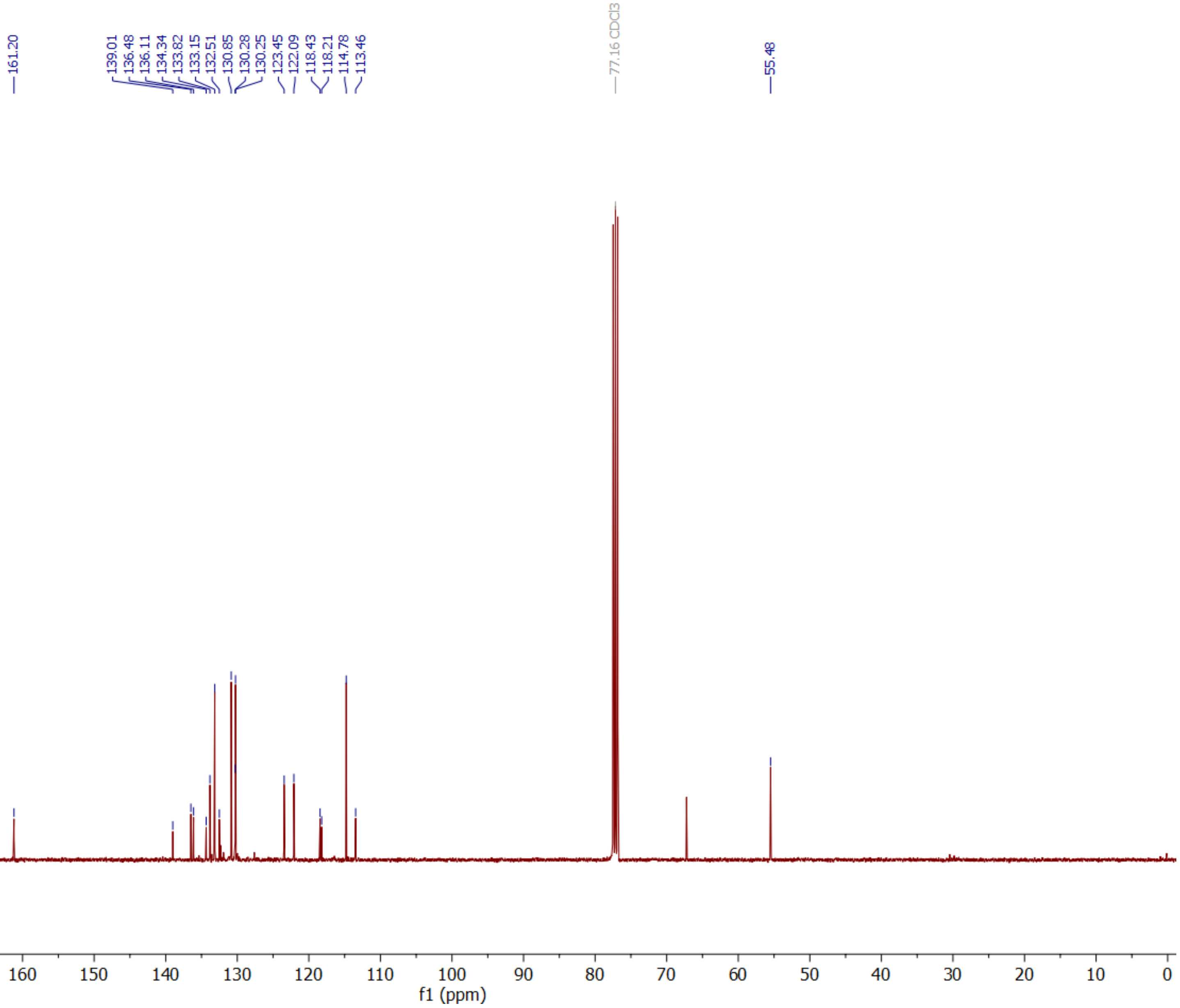

1H (400.15 MHz, CDCl3)

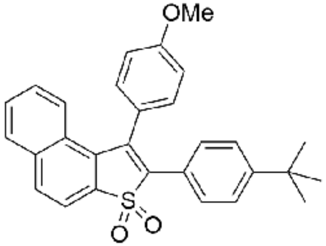

3p

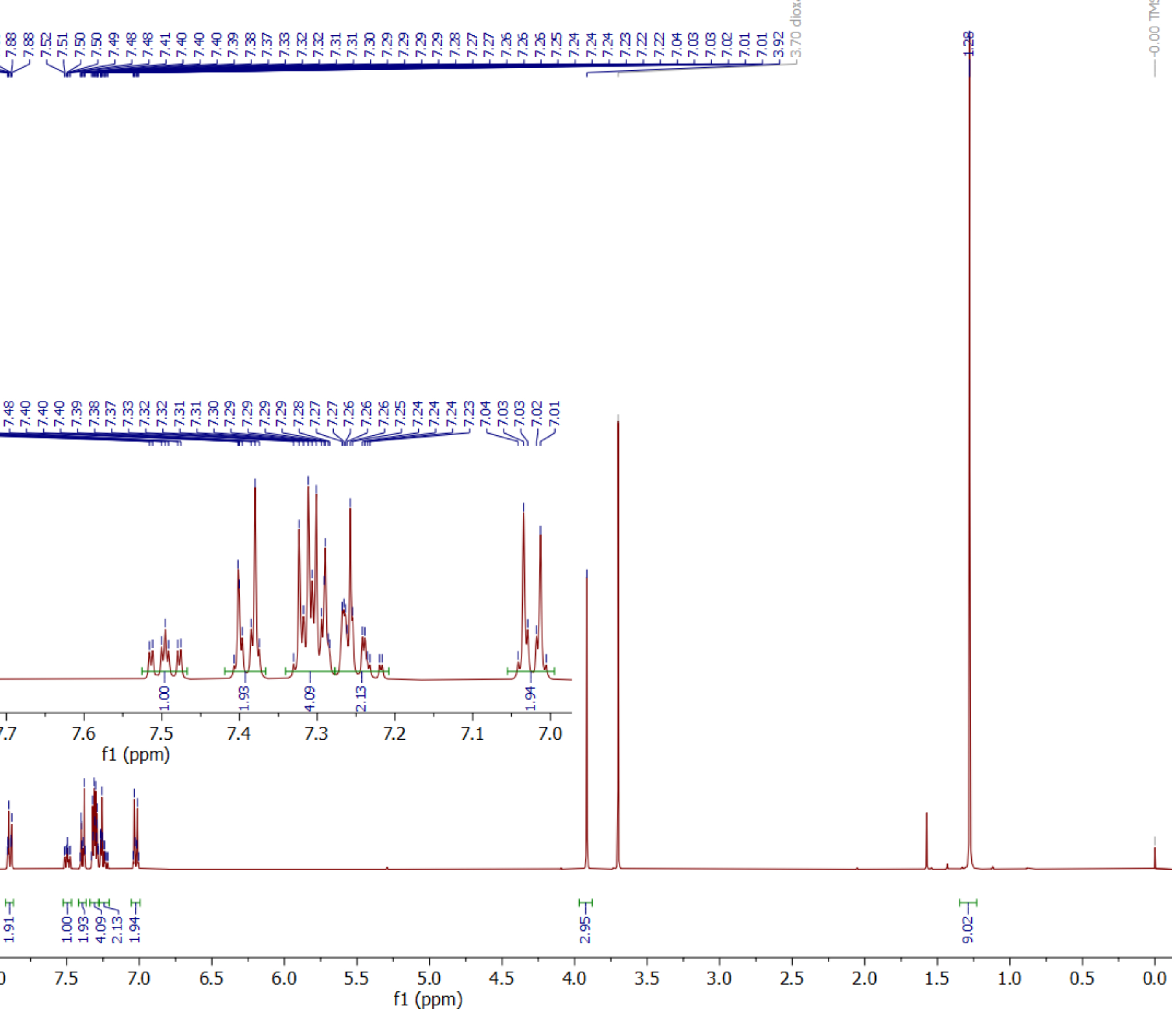

13C (100.63 MHz, CDCl3)

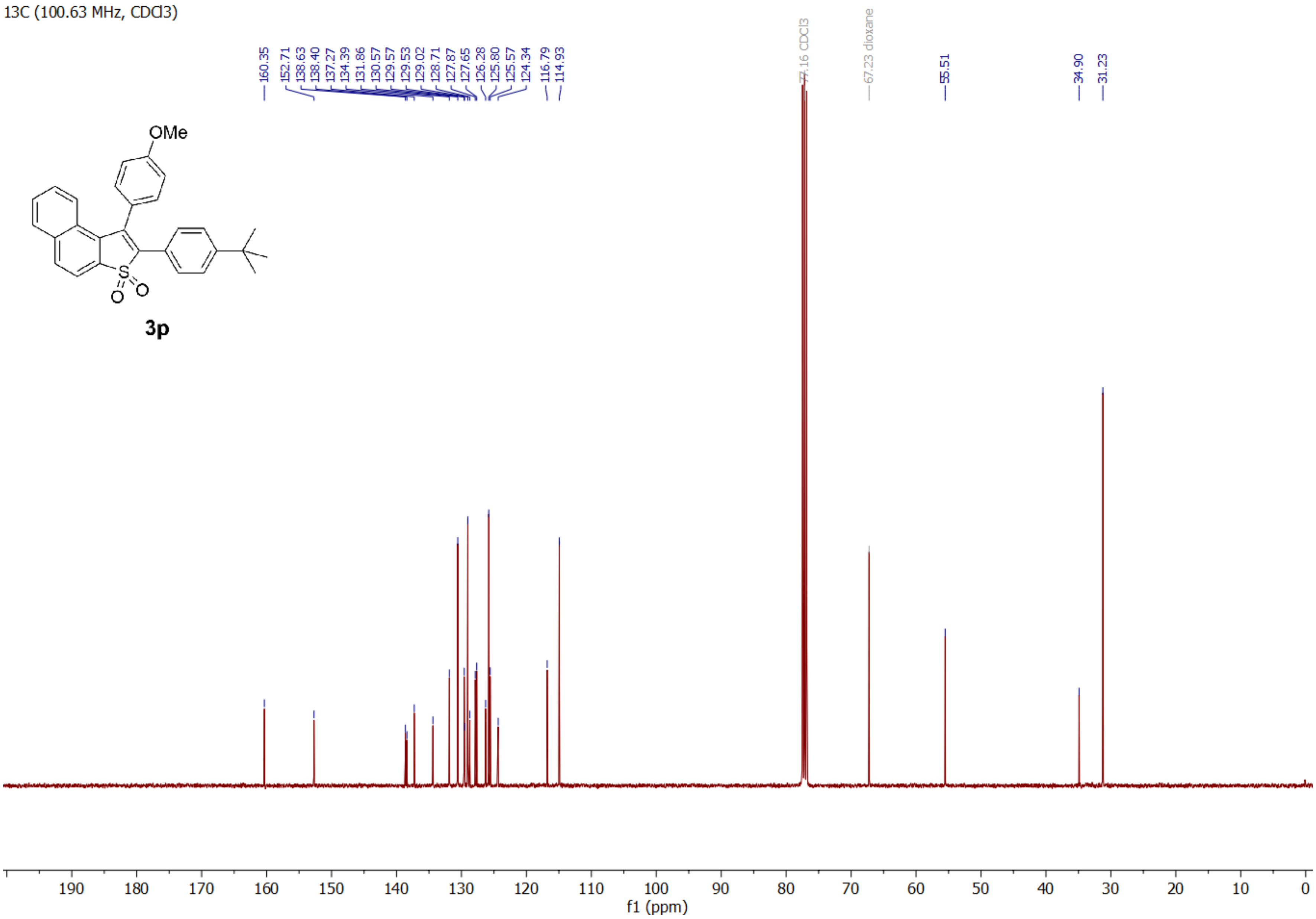

1H (400.15 MHz, CDCl3)

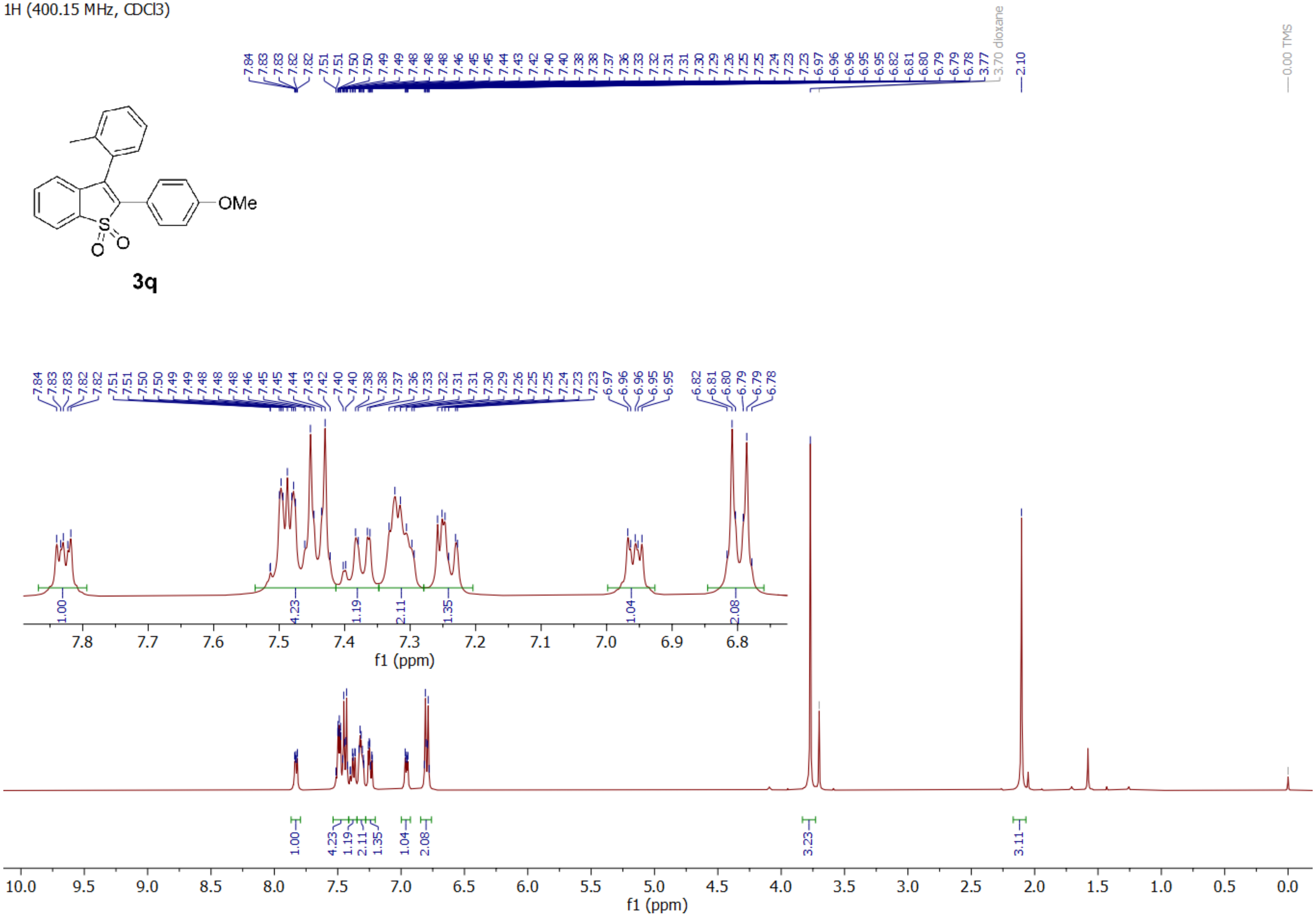

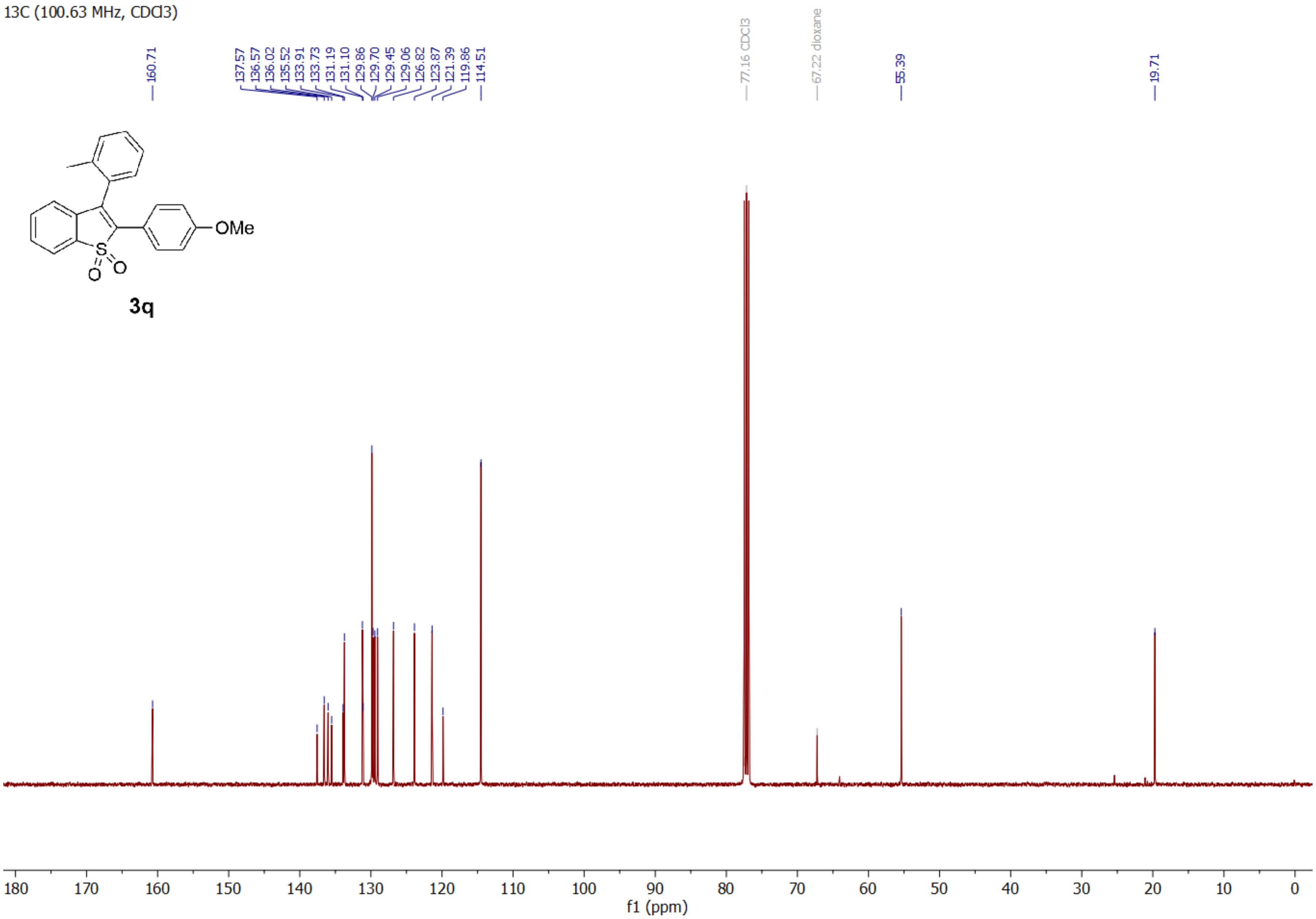
13C (100.63 MHz, CDCl3)
160.71
137.57
136.57
136.02
135.52
133.91
133.73
131.19
131.10
129.86
129.70
129.45
129.06
126.82
123.87
121.39
119.86
114.51
77.16 CDCl3
67.22 dioxane
55.39
19.71
OMe
3q
f1 (ppm)

1H (400.15 MHz, CDCl3)

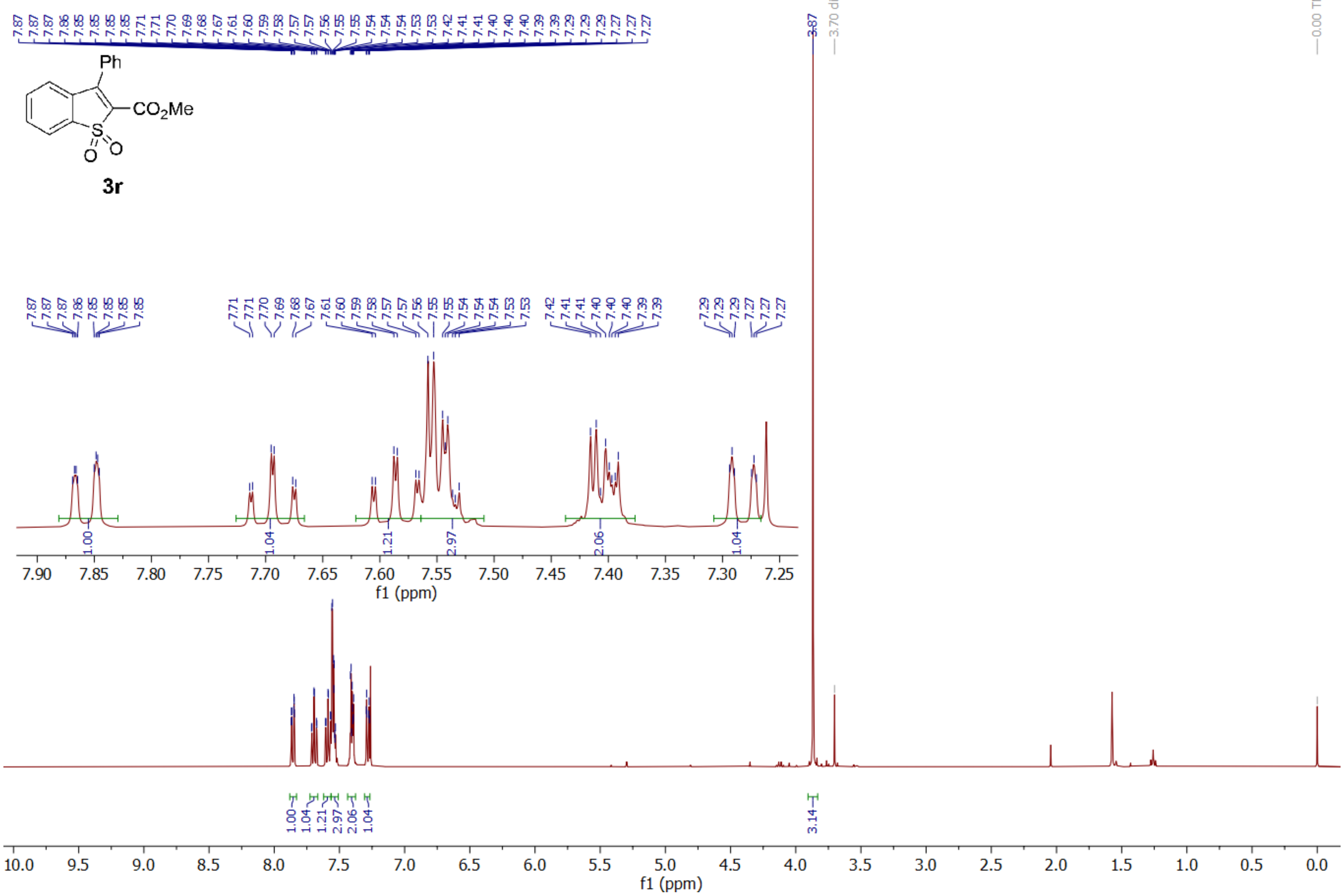

13C (100.63 MHz, CDCl3)

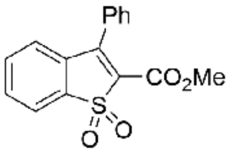

3r

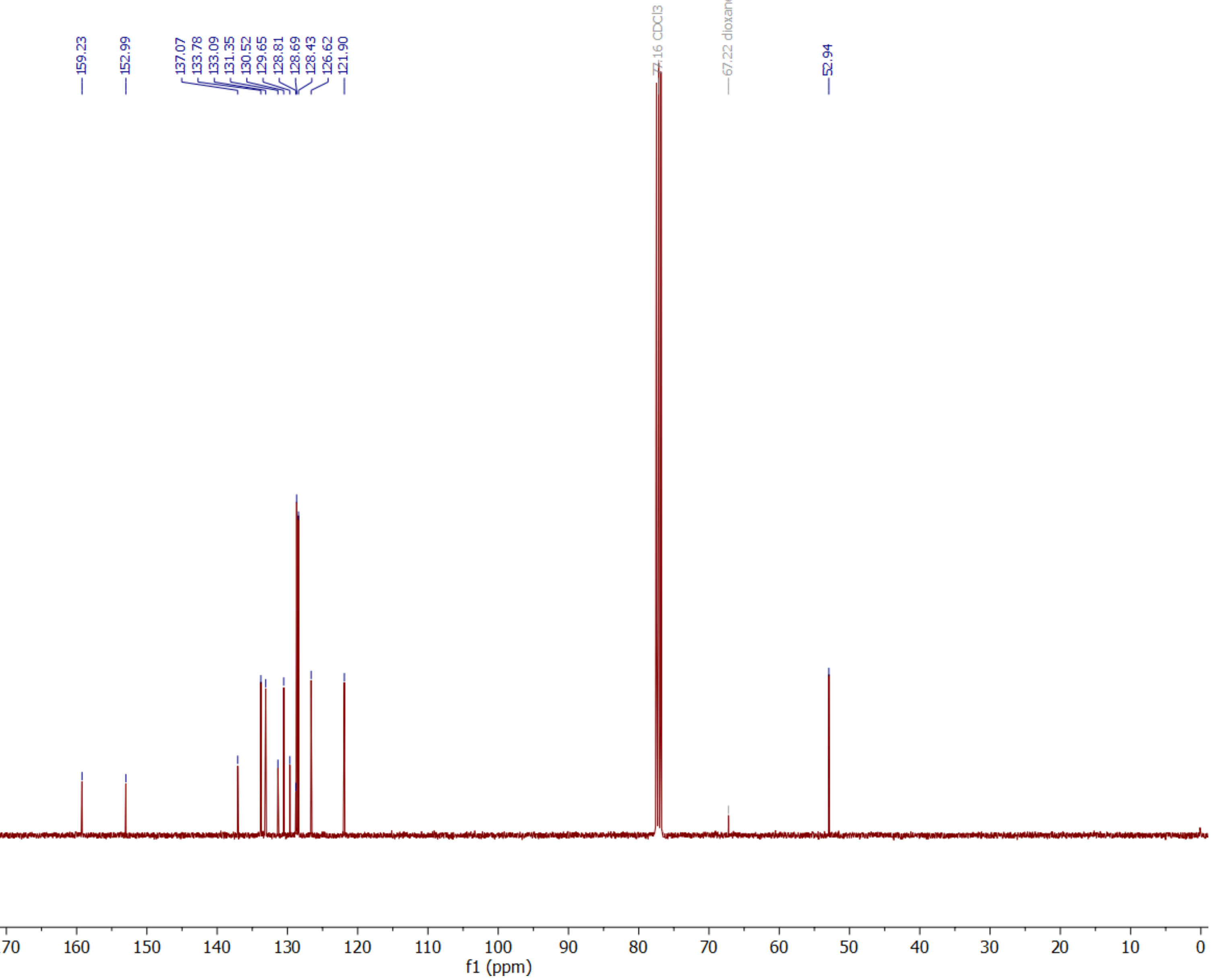

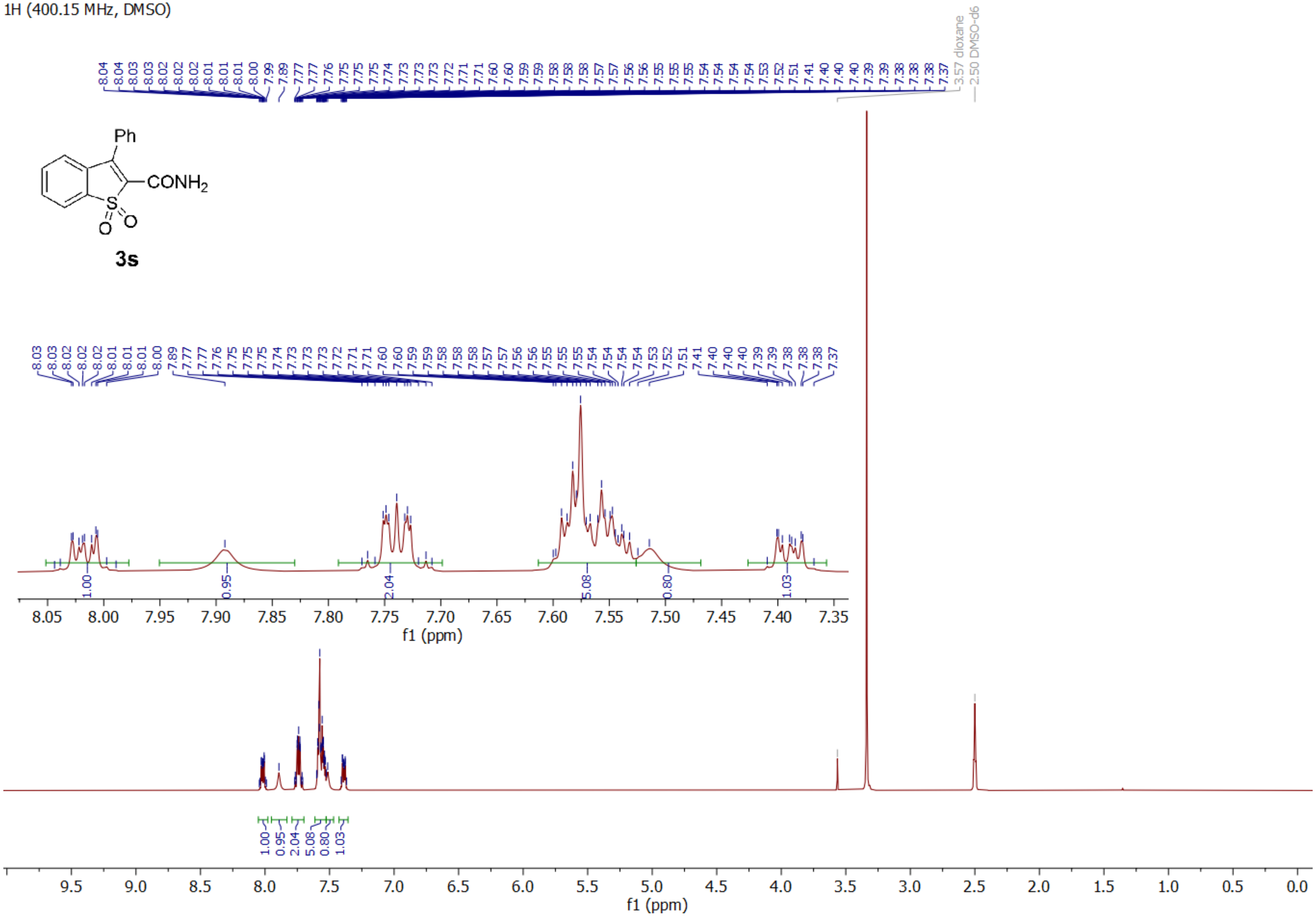

1H (400.15 MHz, DMSO)
Ph
CONH2
S
O
O
3s
3.57 dioxane
2.50 DMSO-d6
1.00
0.95
2.04
5.08
0.80
1.03
f1 (ppm)
f1 (ppm)

13C (100.63 MHz, DMSO)

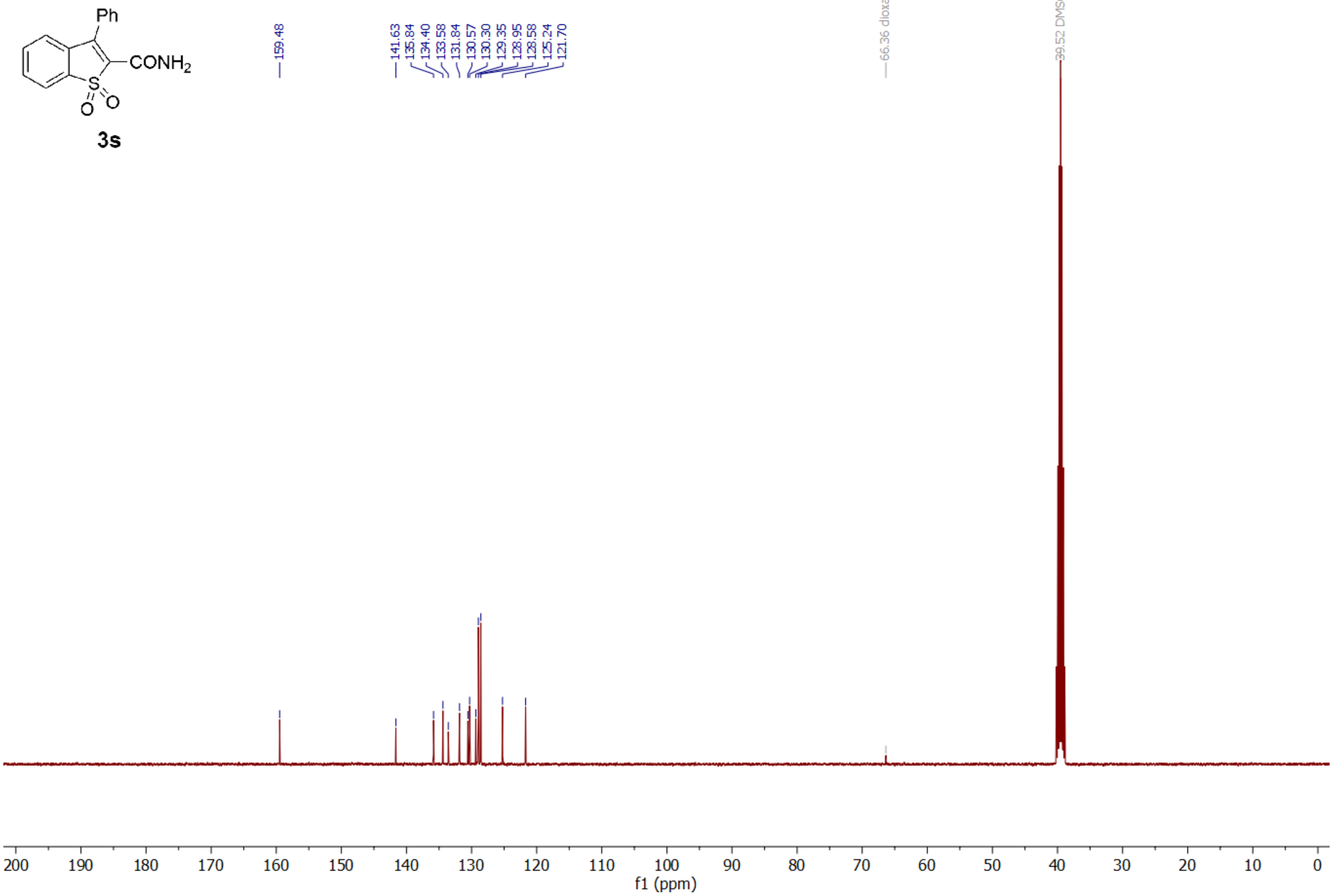

1H (400.15 MHz, CDCl3)

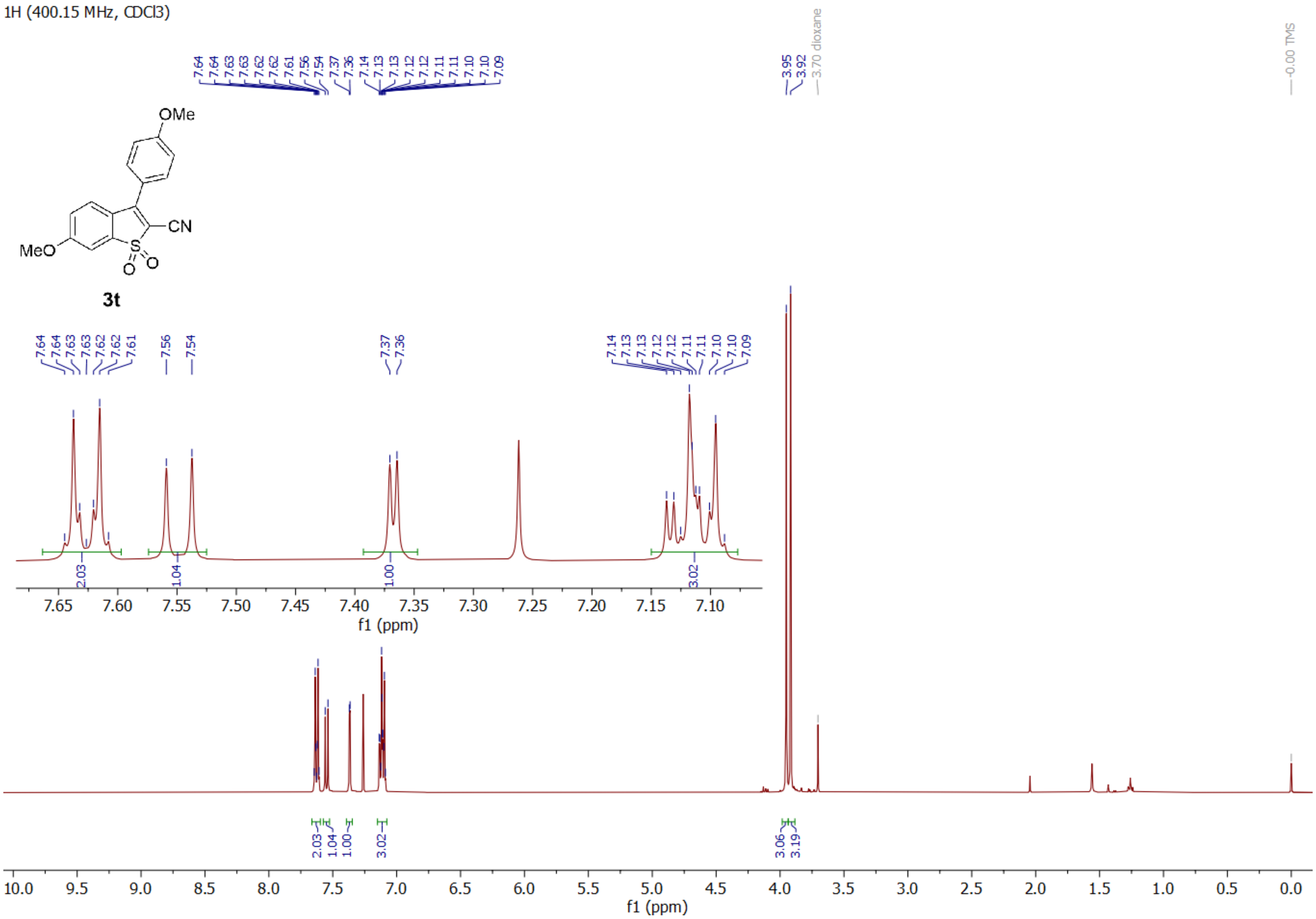

13C (100.63 MHz, CDCl3)

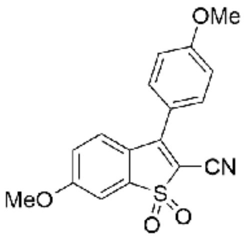

3t

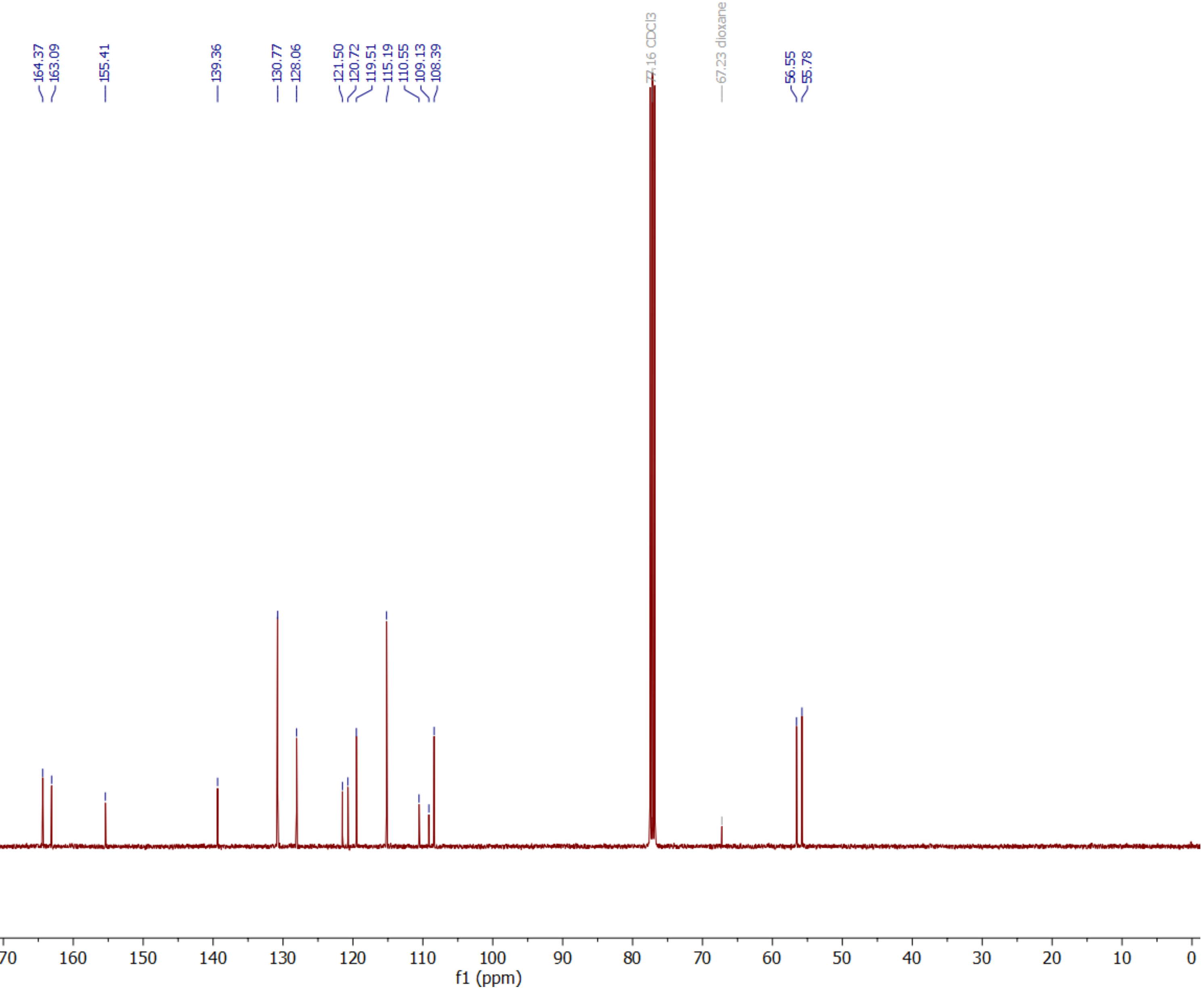

1H (400.15 MHz, CDCl3)

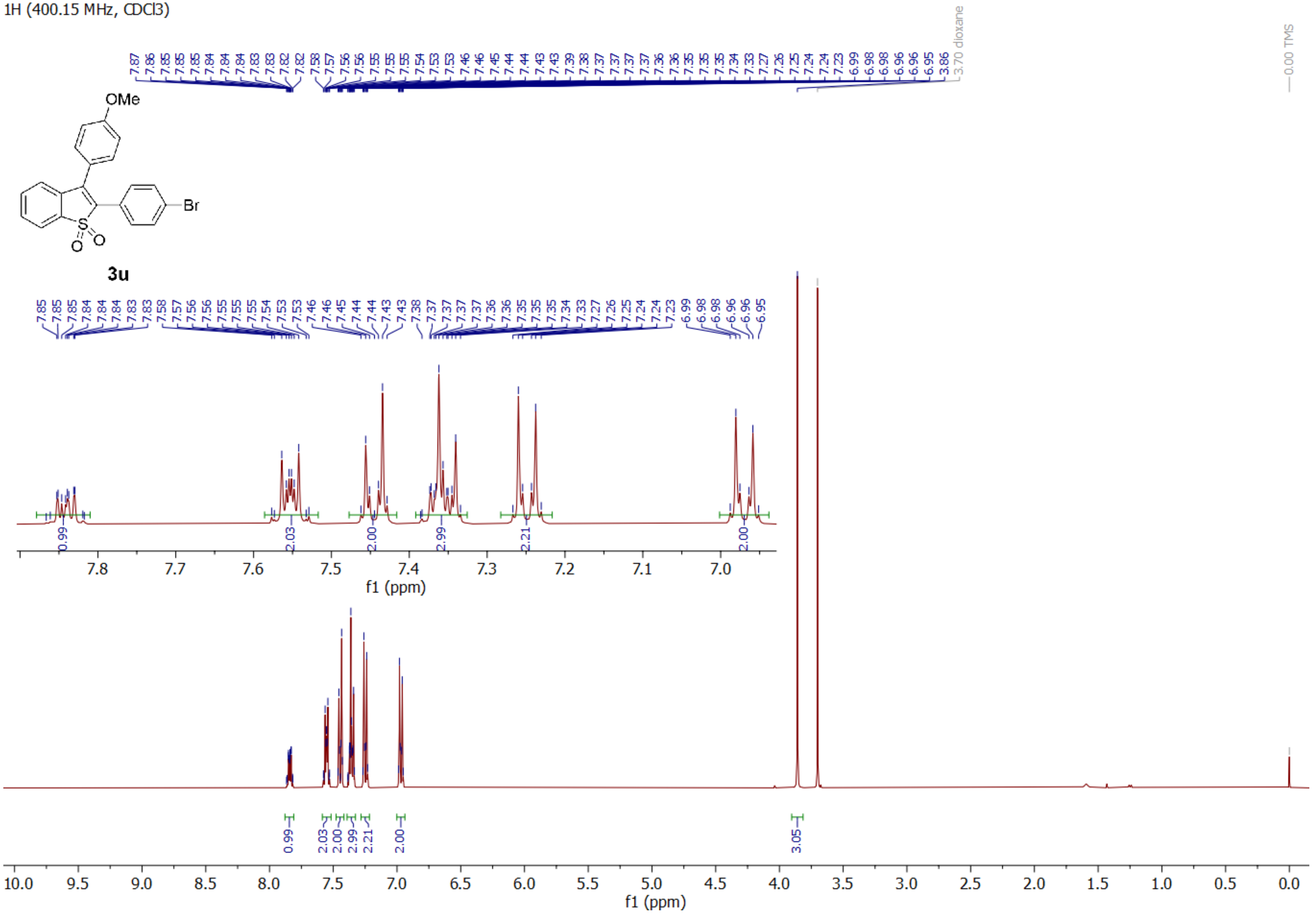

13C (100.63 MHz, CDCl3)

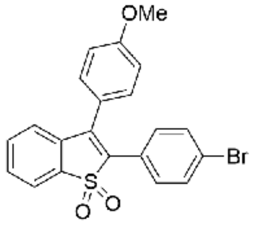

3u

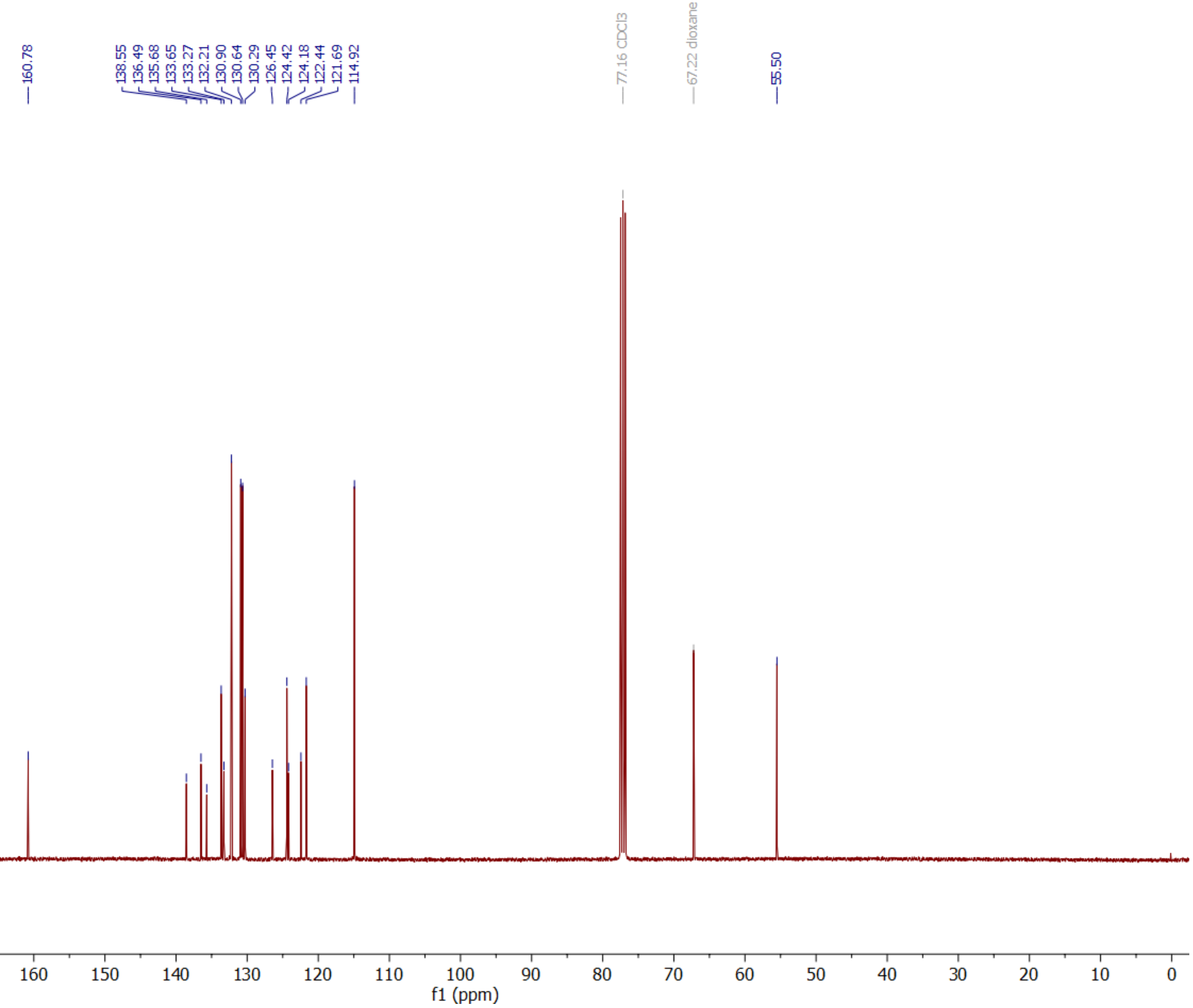

1H (400.15 MHz, CDCl3)

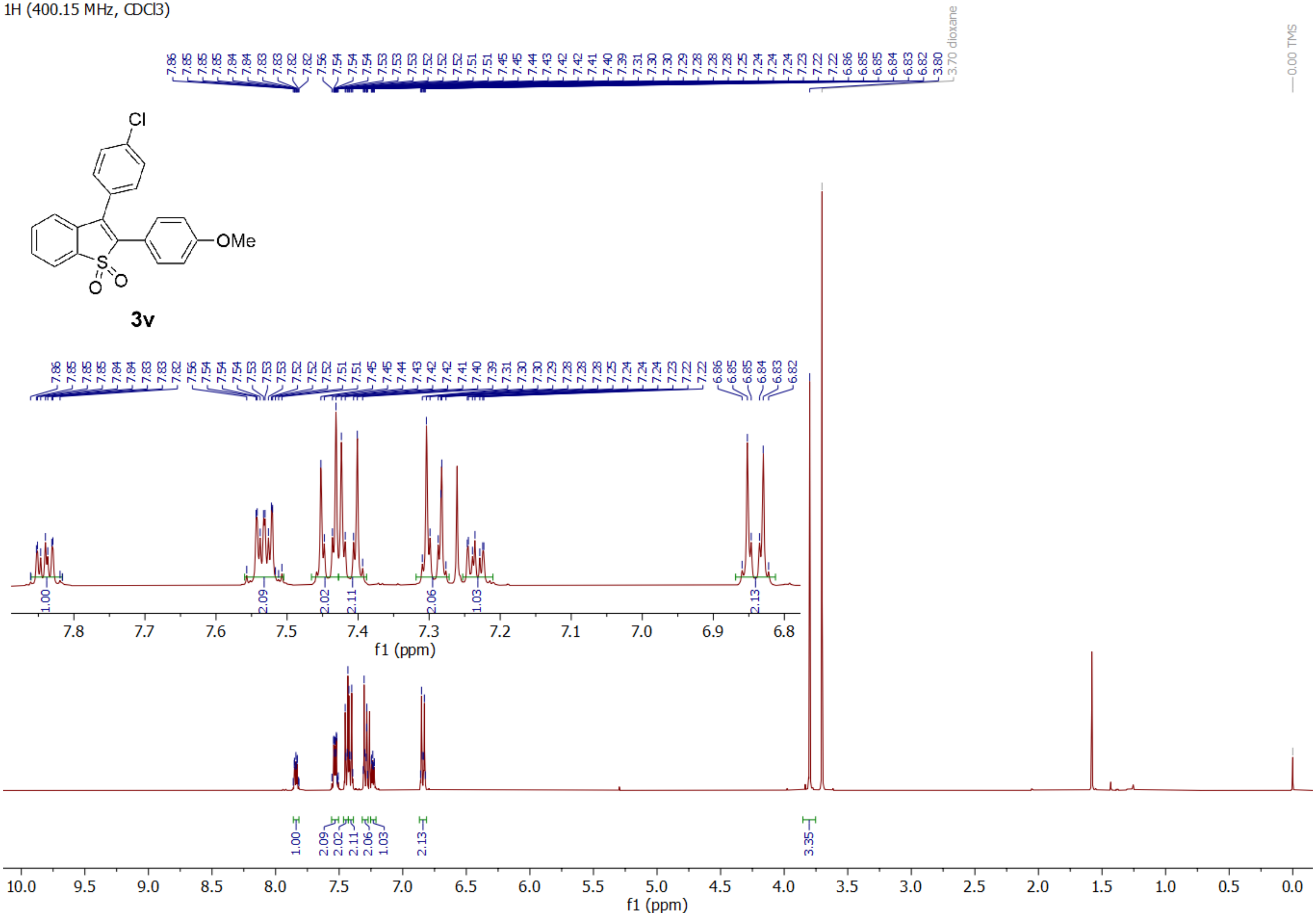

13C (100.63 MHz, CDCl3)

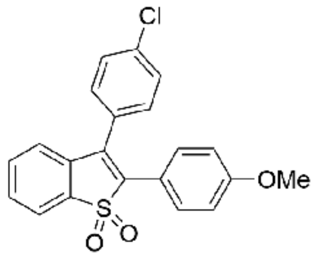

3v

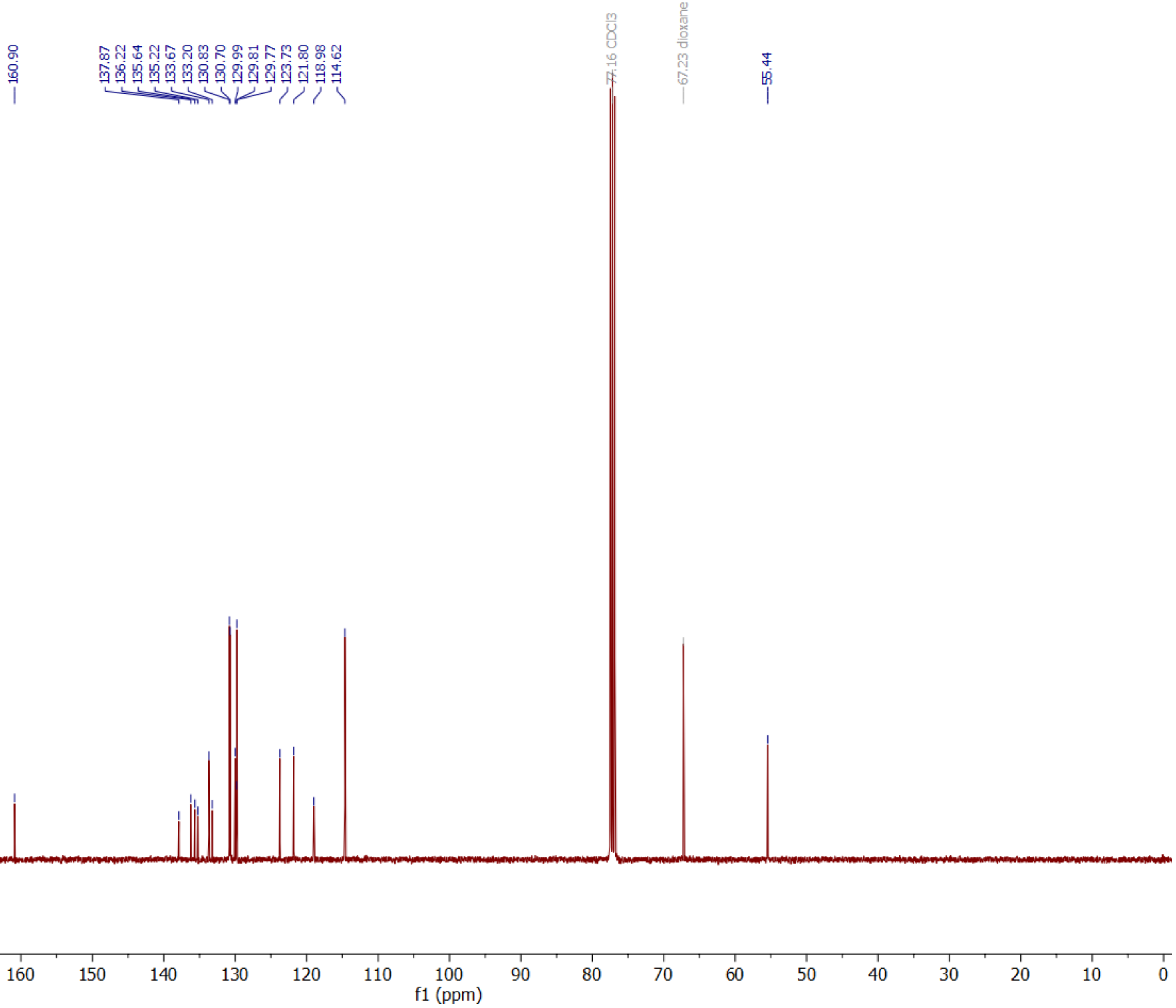

1H (400.15 MHz, CDCl3)

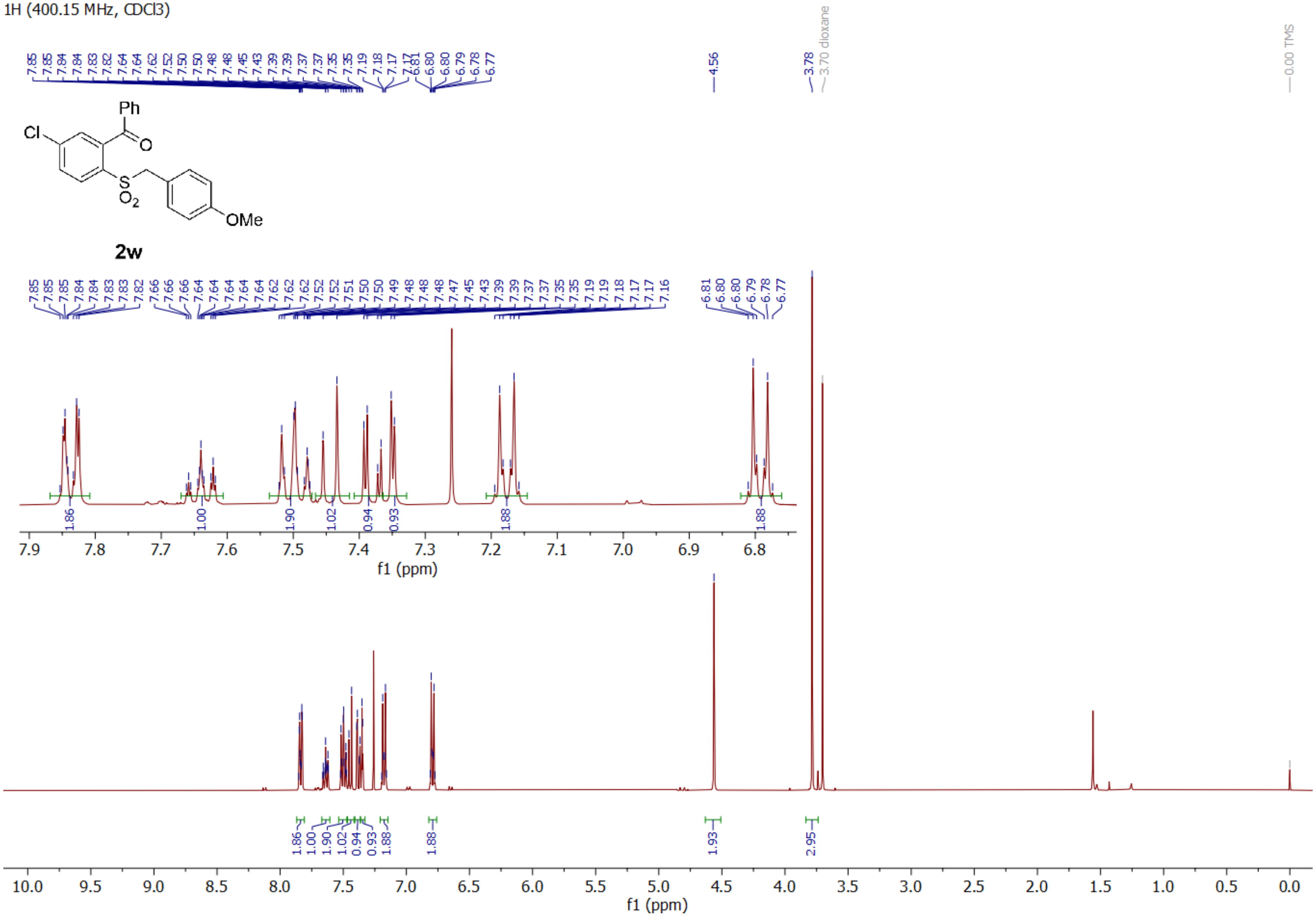

2w

13C (100.63 MHz, CDCl3)

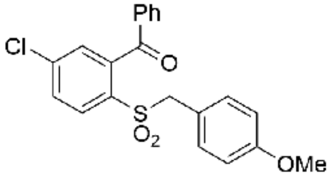

2w

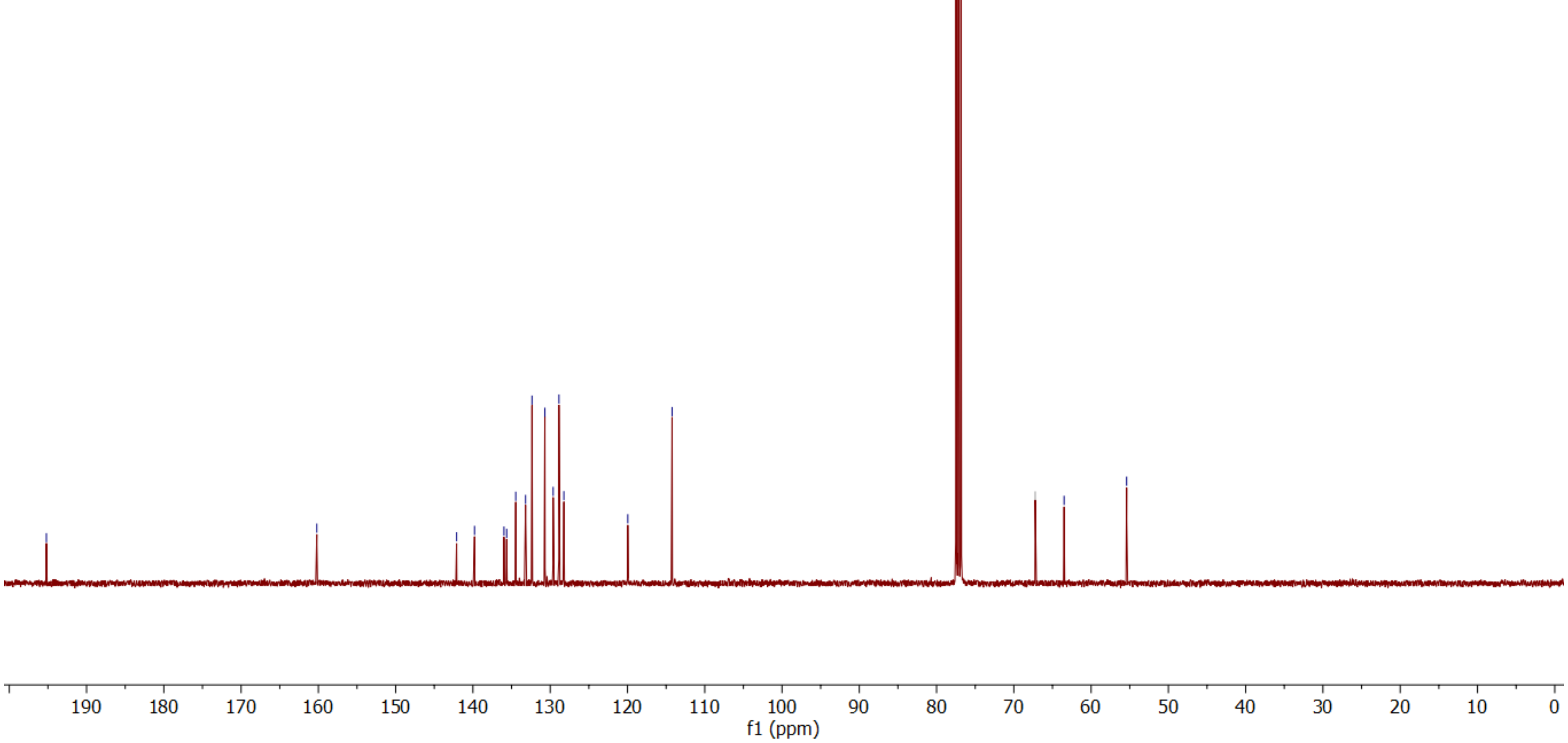

1H (400.15 MHz, CDCl3)

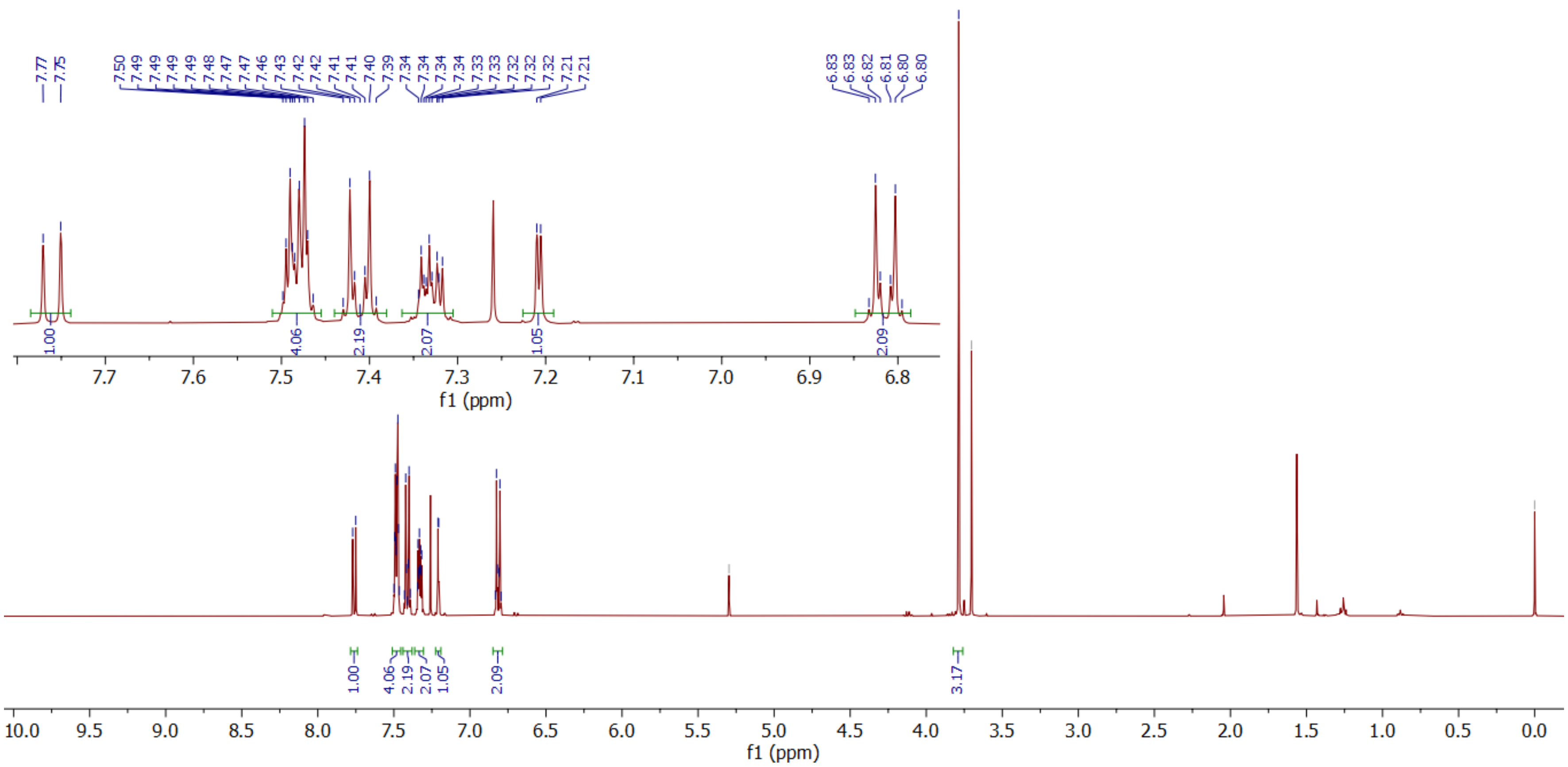

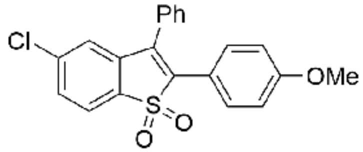

3w

13C (100.63 MHz, CDCl3)

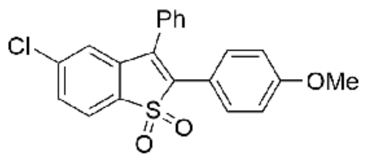

3w

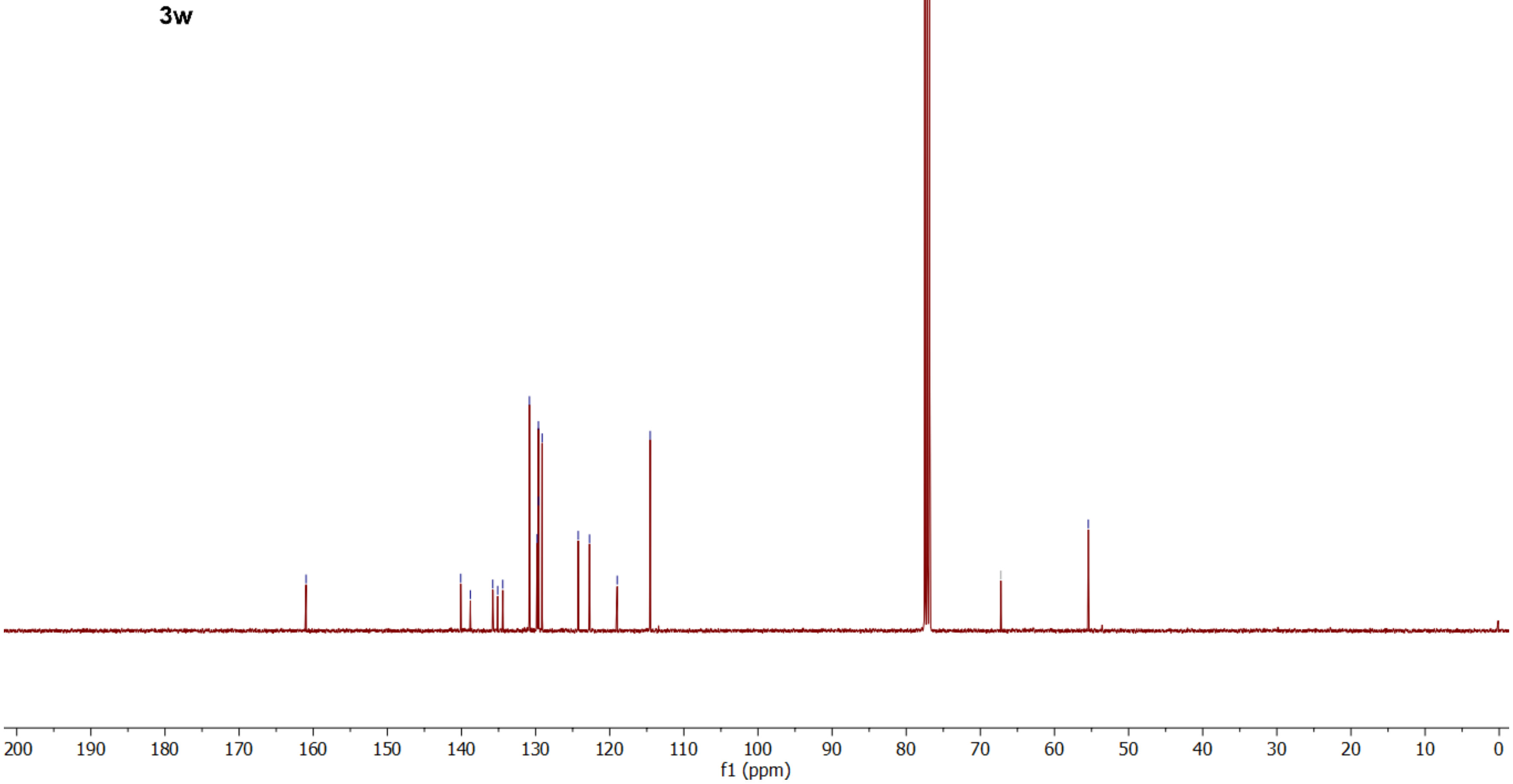

1H (400.15 MHz, Pyridine-d5, 60 °C)

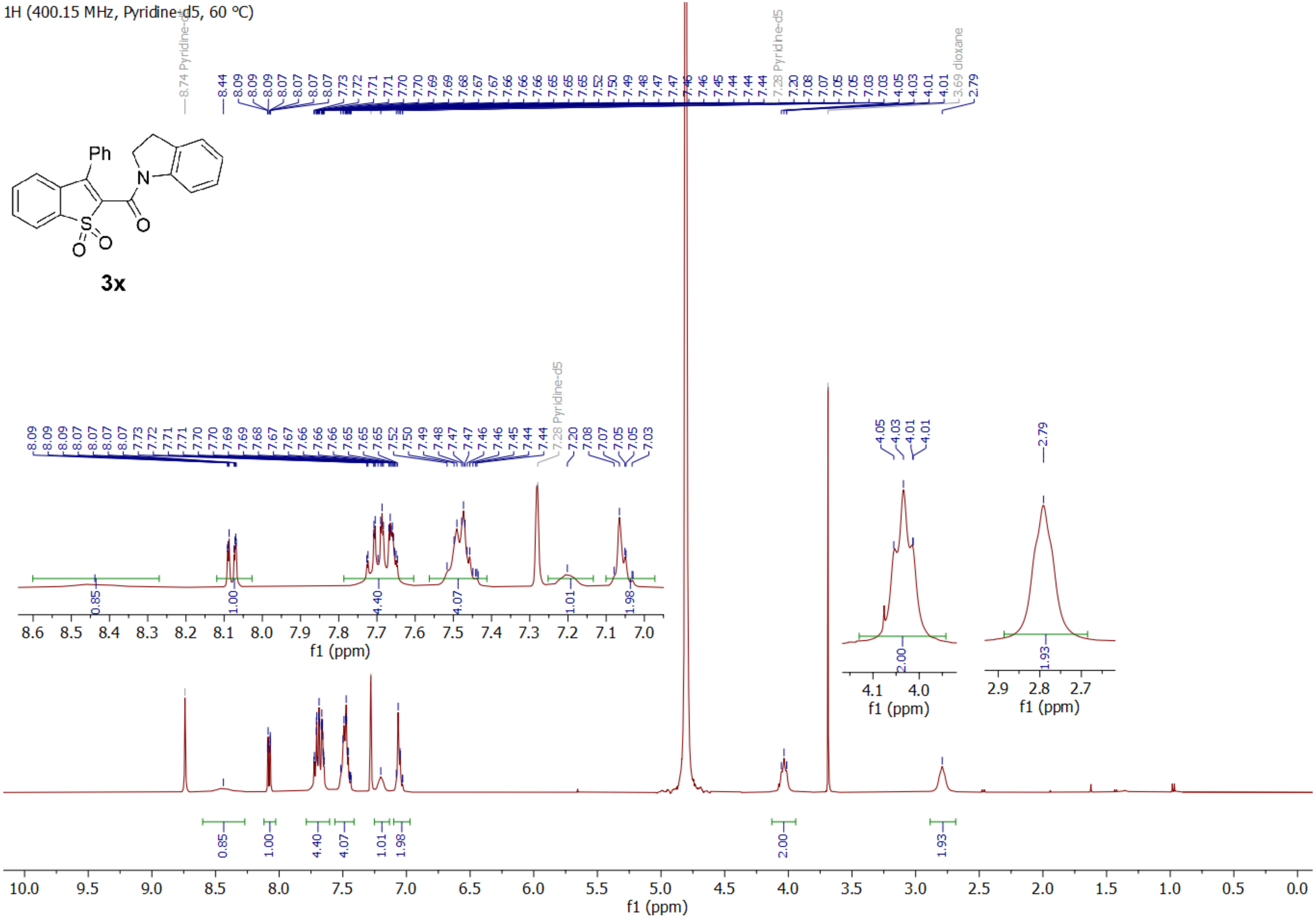

3x

13C (100.63 MHz, DMSO)

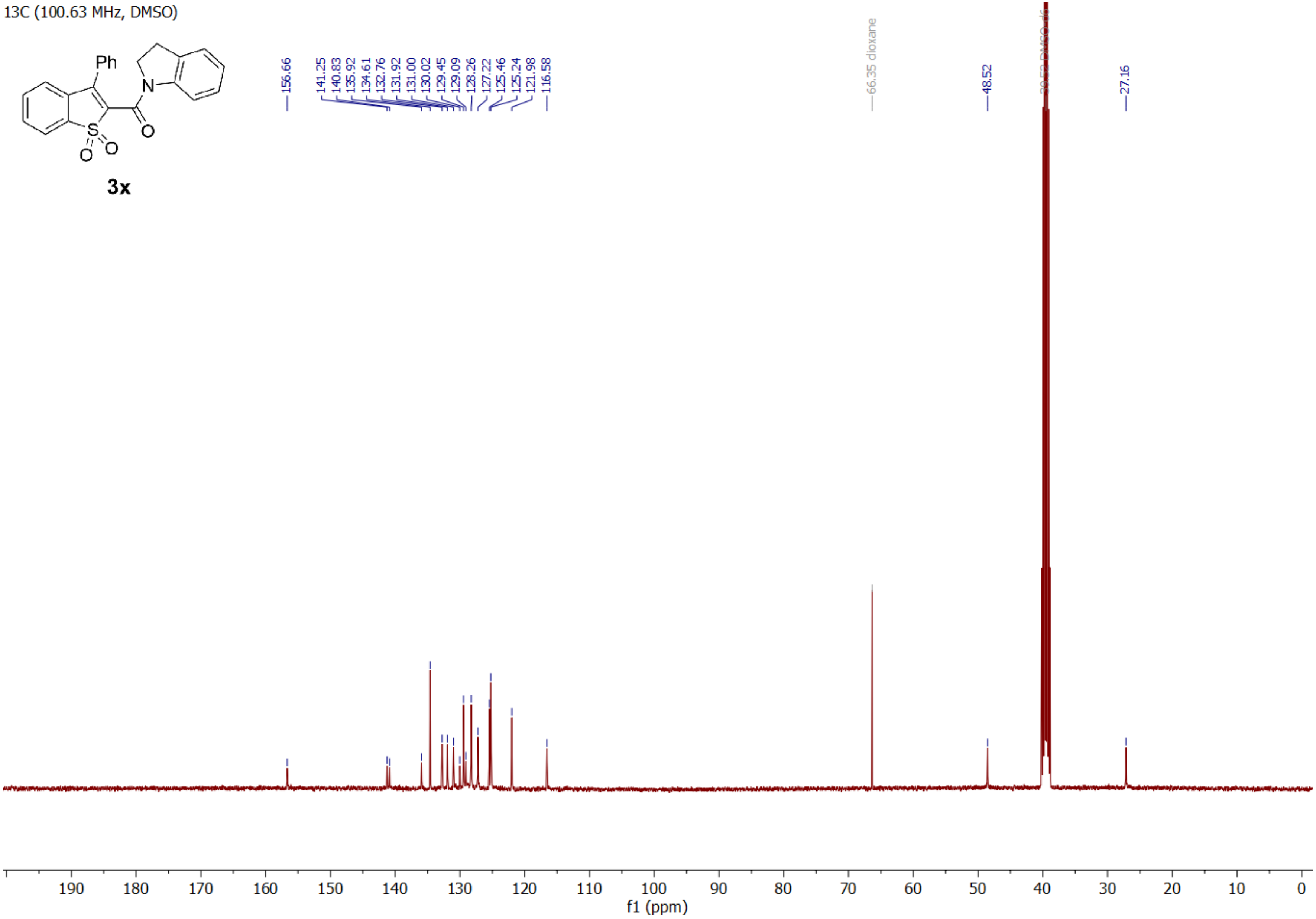

3x

1H (400.15 MHz, CDCl3)

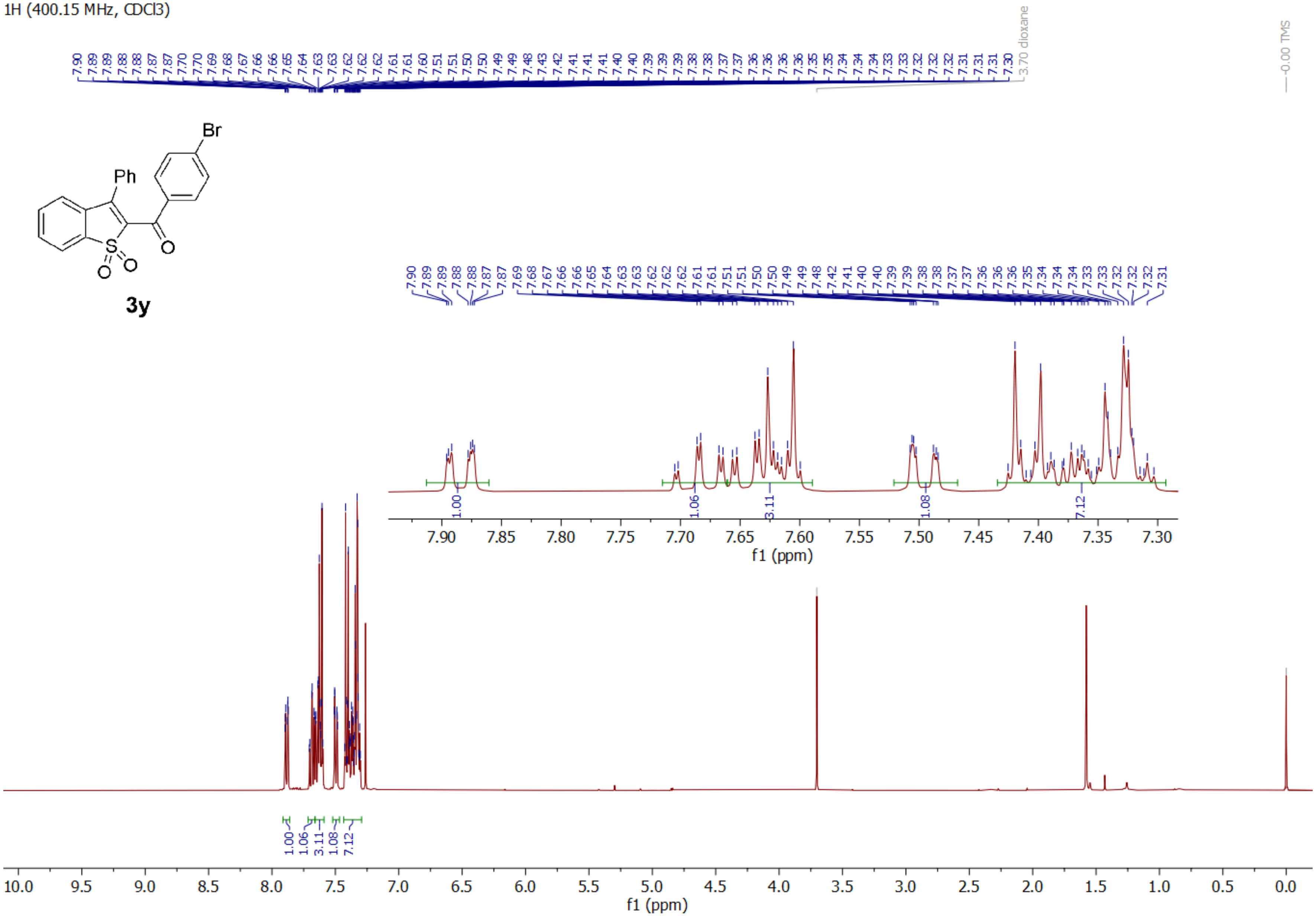

3y

13C (100.63 MHz, CDCl3)

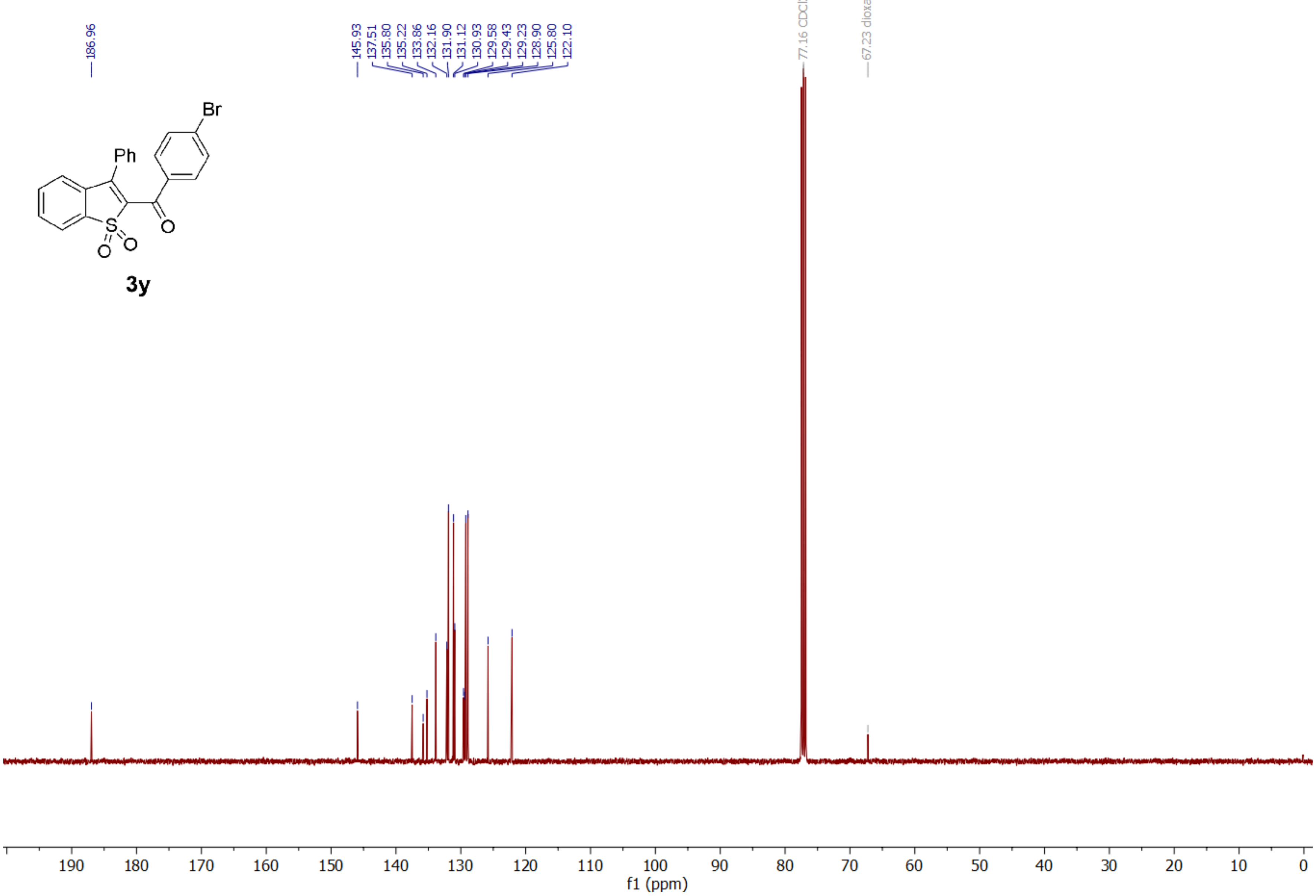

1H (400.15 MHz, CDCl3)

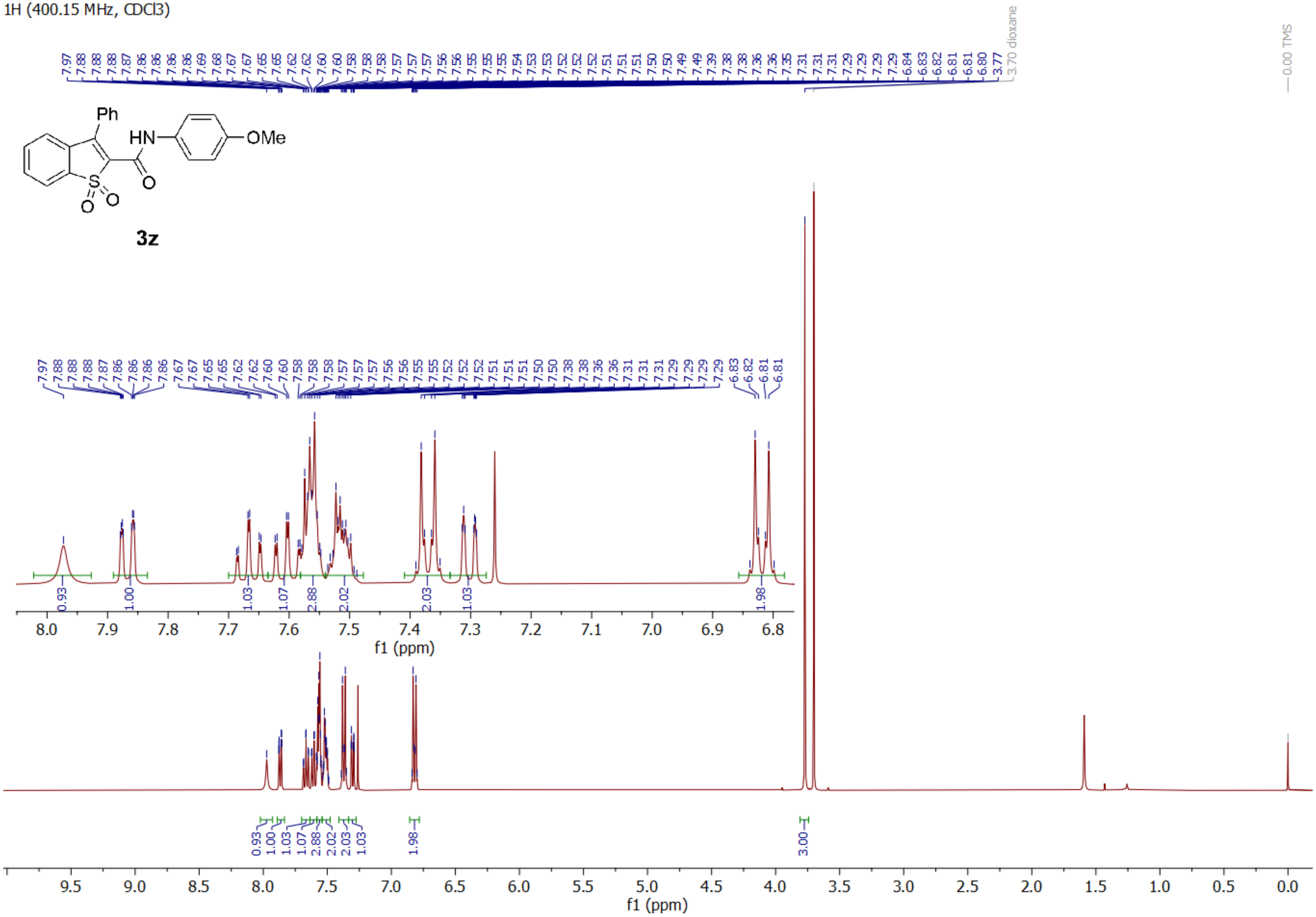

3z

13C (100.63 MHz, CDCl3)

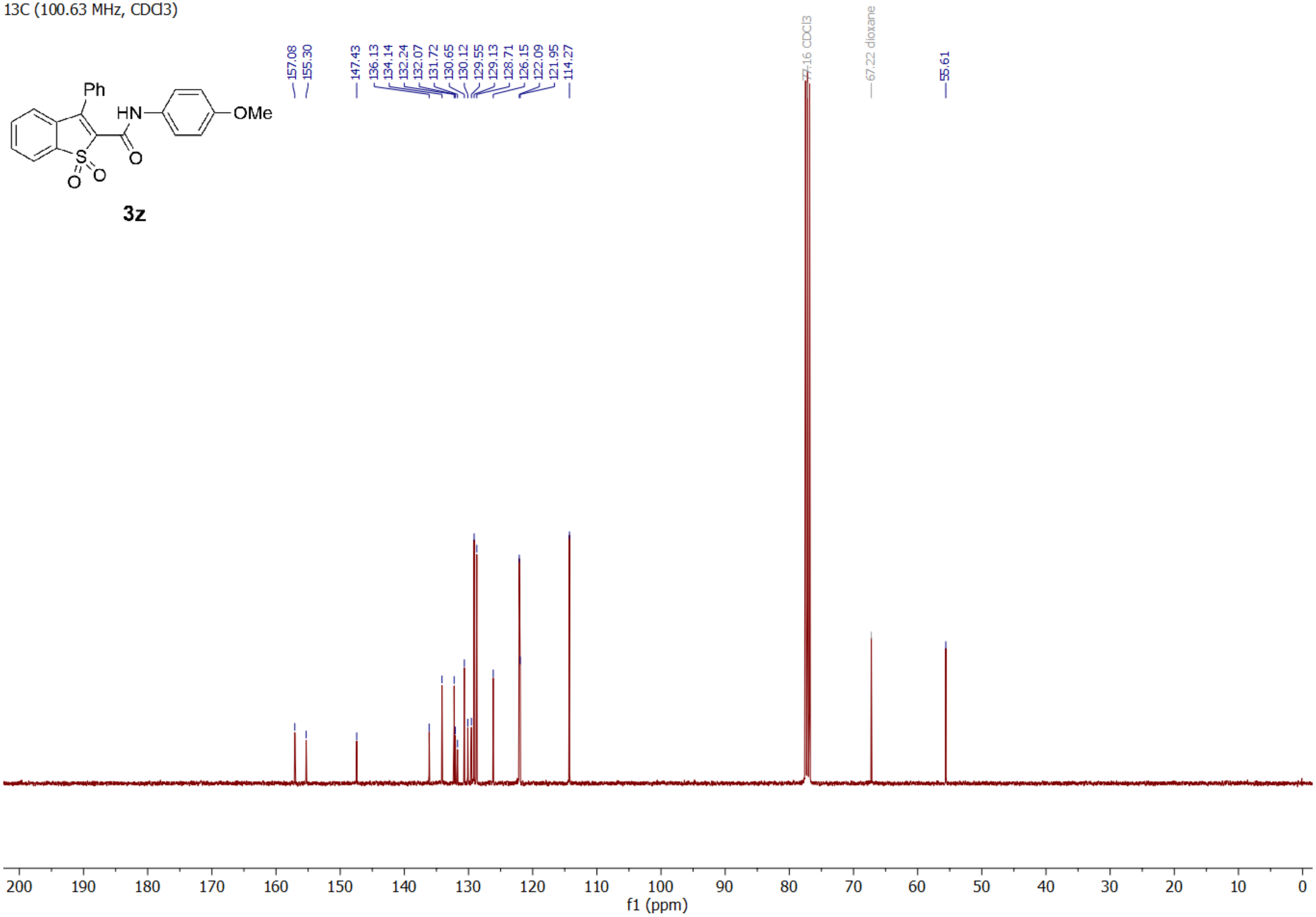

3z

1H (400.15 MHz, CDCl3)

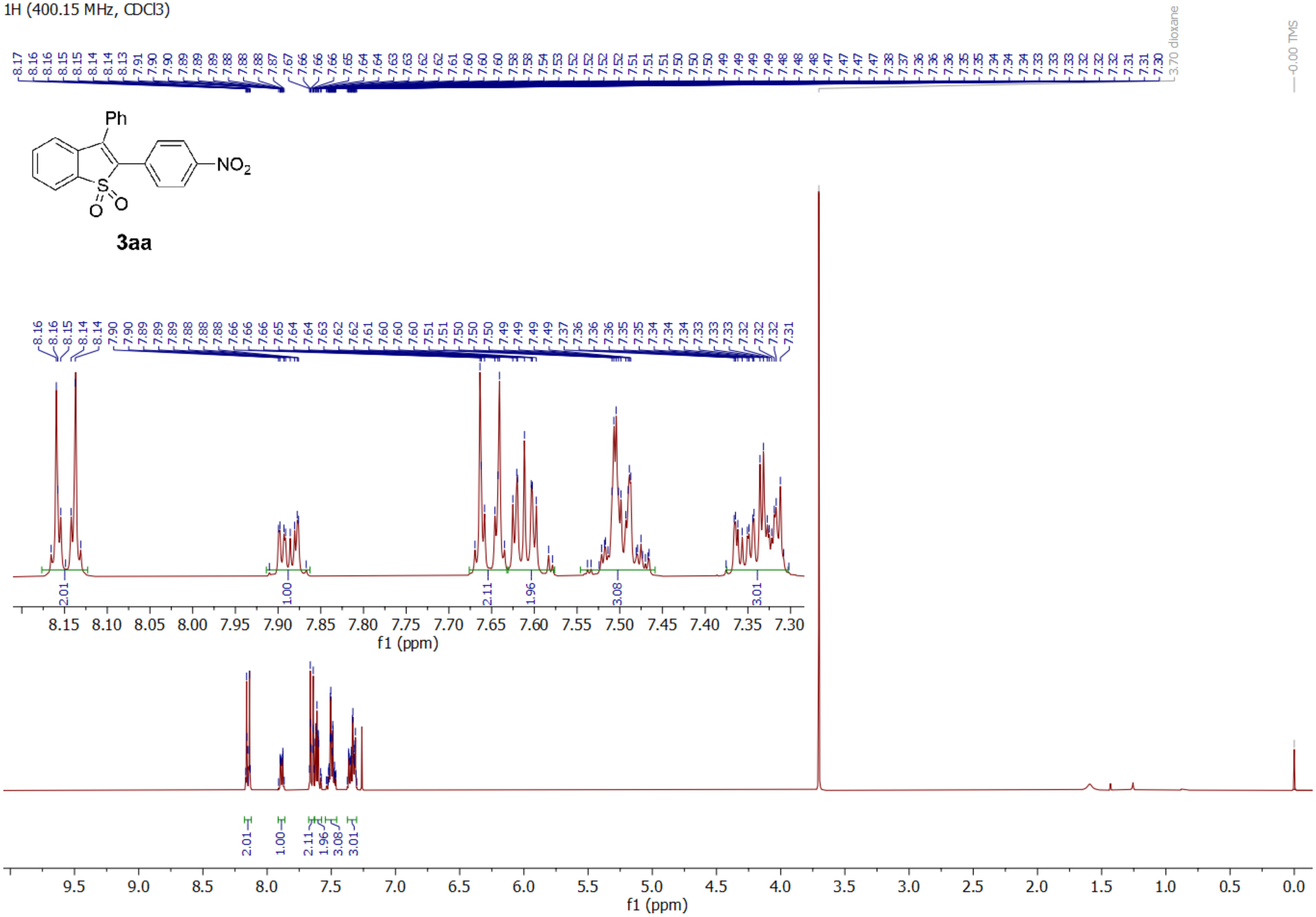

13C (100.63 MHz, CDCl3)

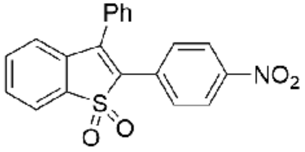

3aa

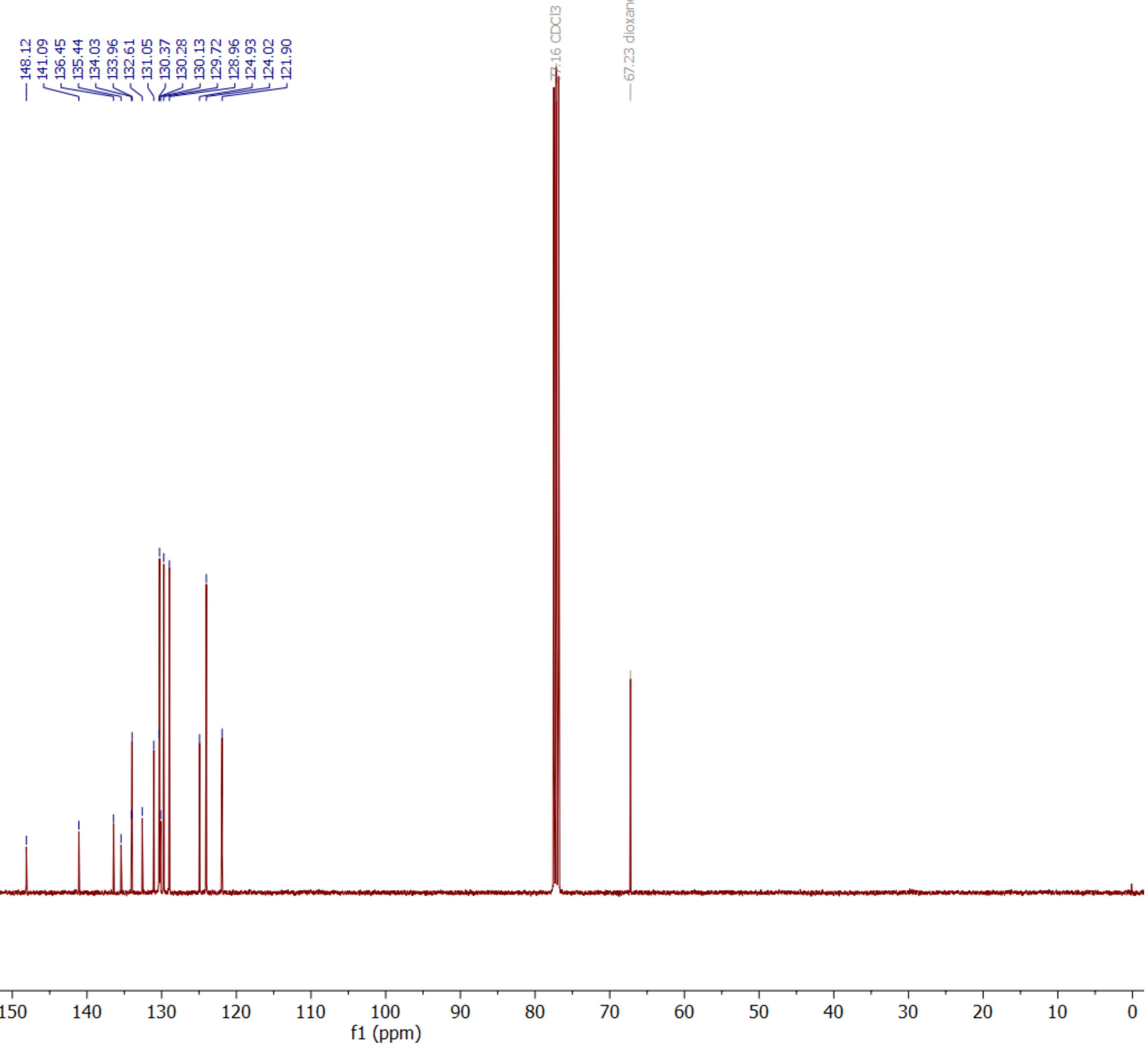

1H (400.15 MHz, CDCl3)

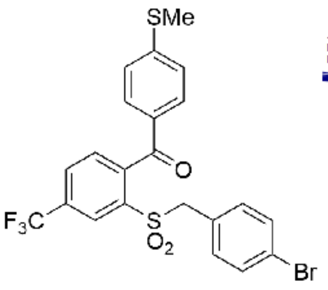

2ab

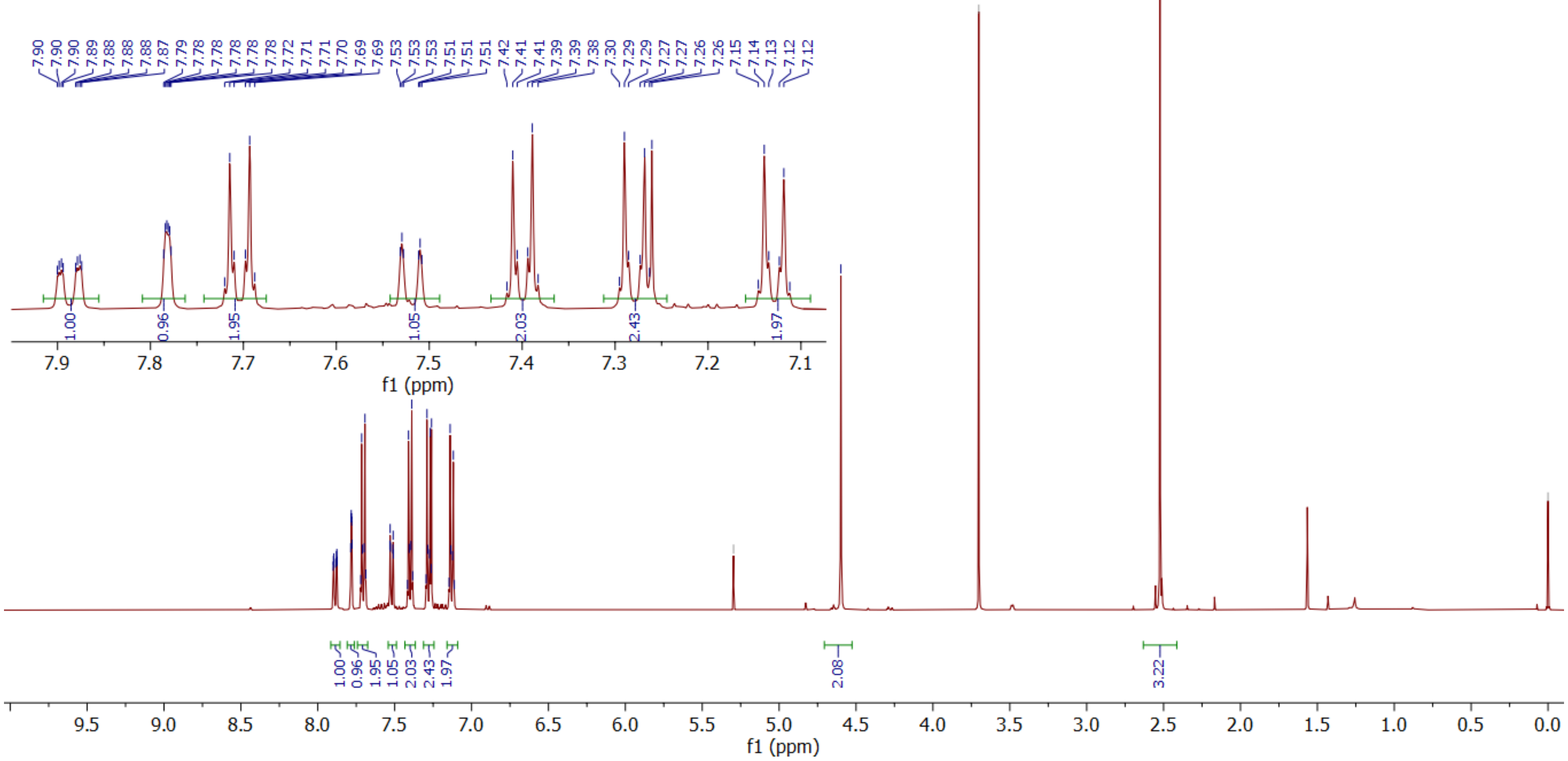

13C (100.63 MHz, CDCl3)

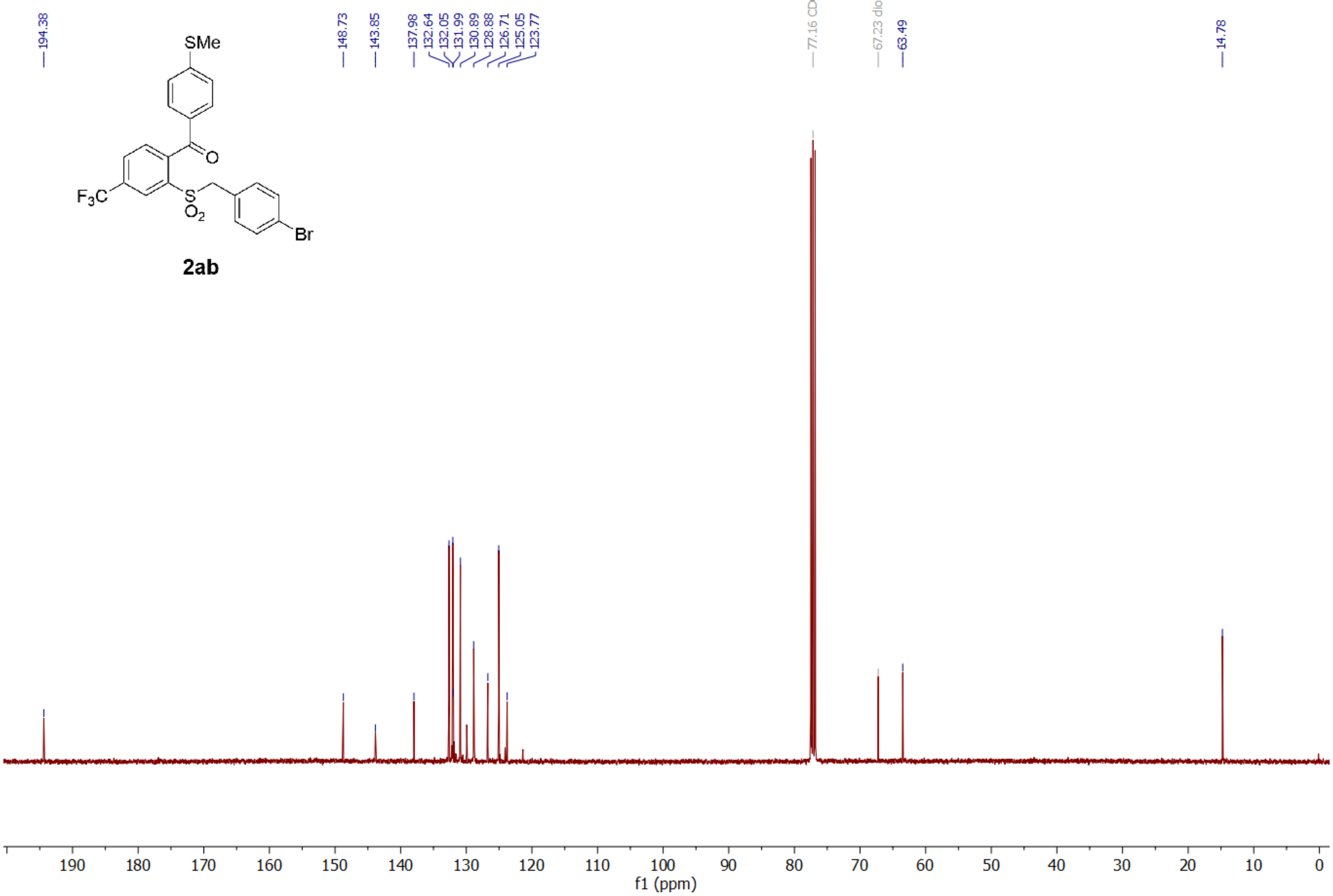

19F (376.48 MHz, CDCl3)

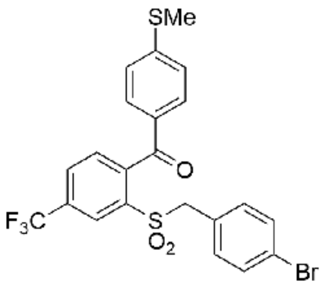

2ab

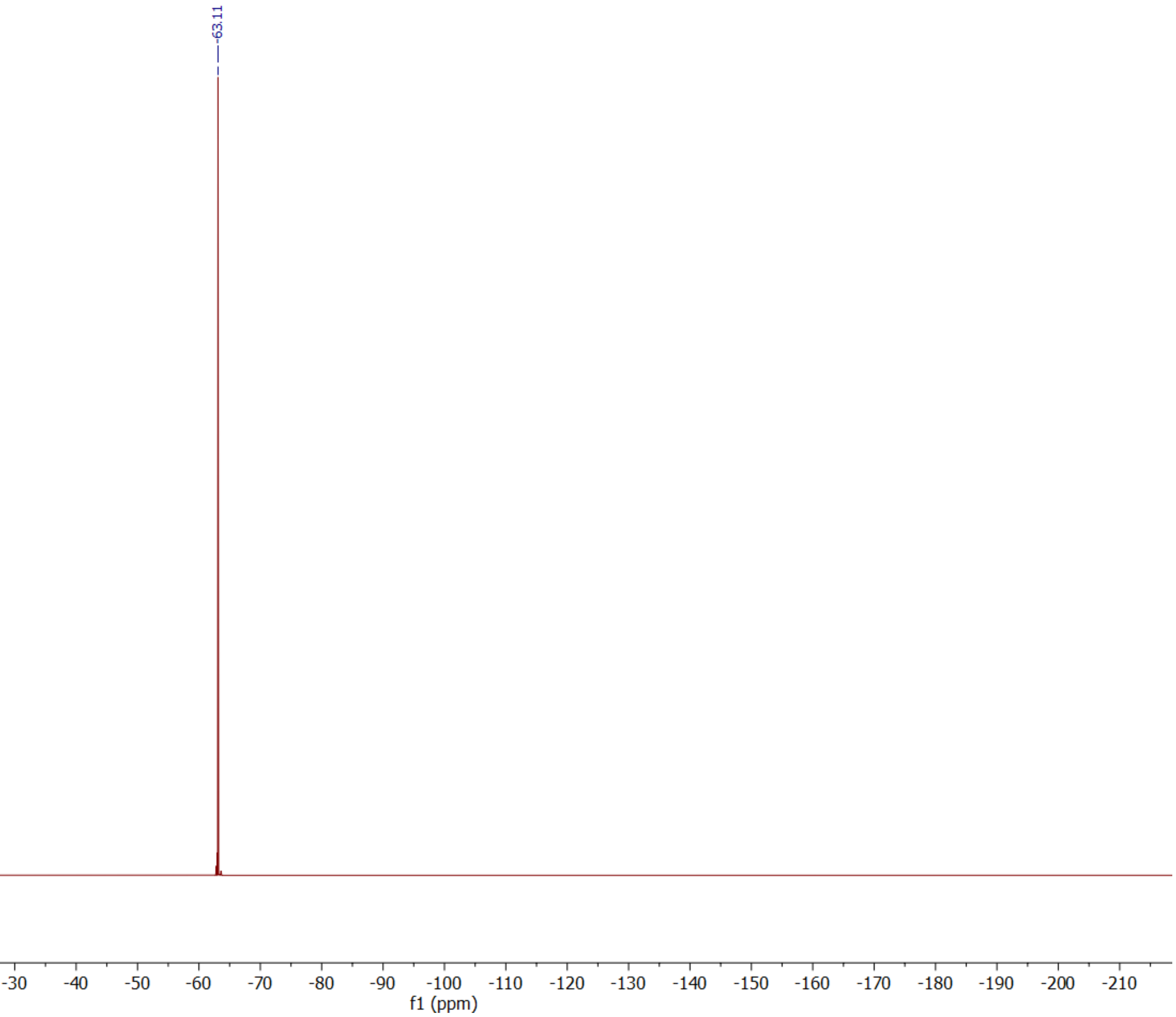

1H (400.15 MHz, CDCl3)

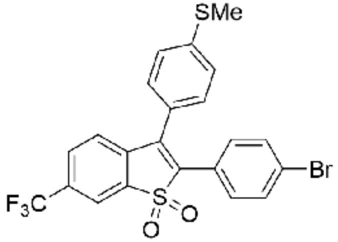

3ab

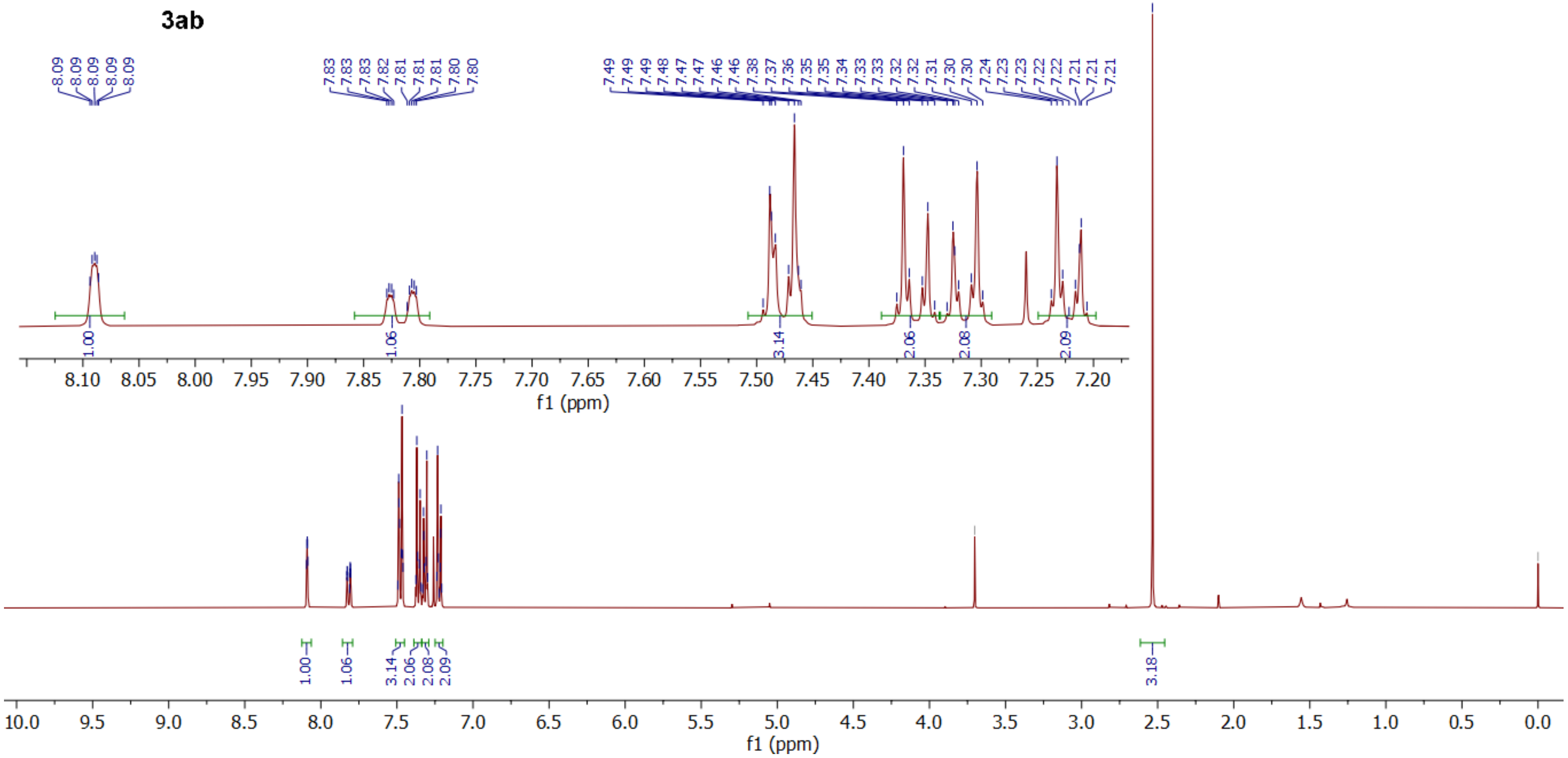

13C (100.63 MHz, CDCl3)

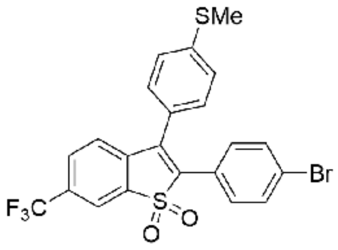

3ab

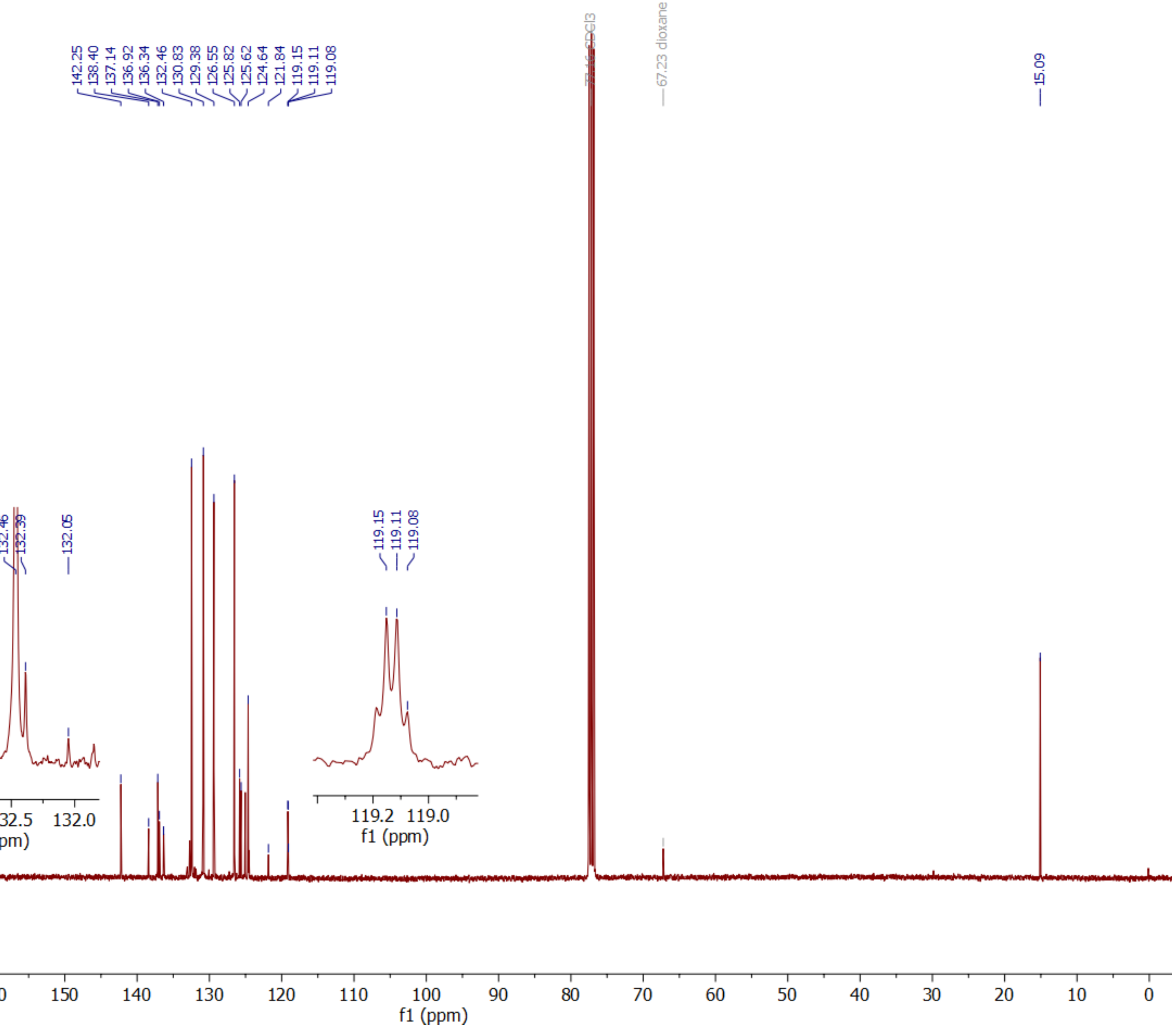

19F (376.48 MHz, CDCl3)

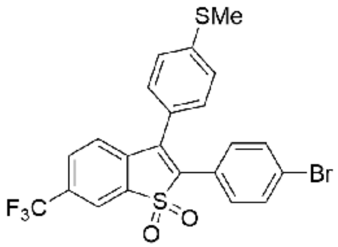

3ab

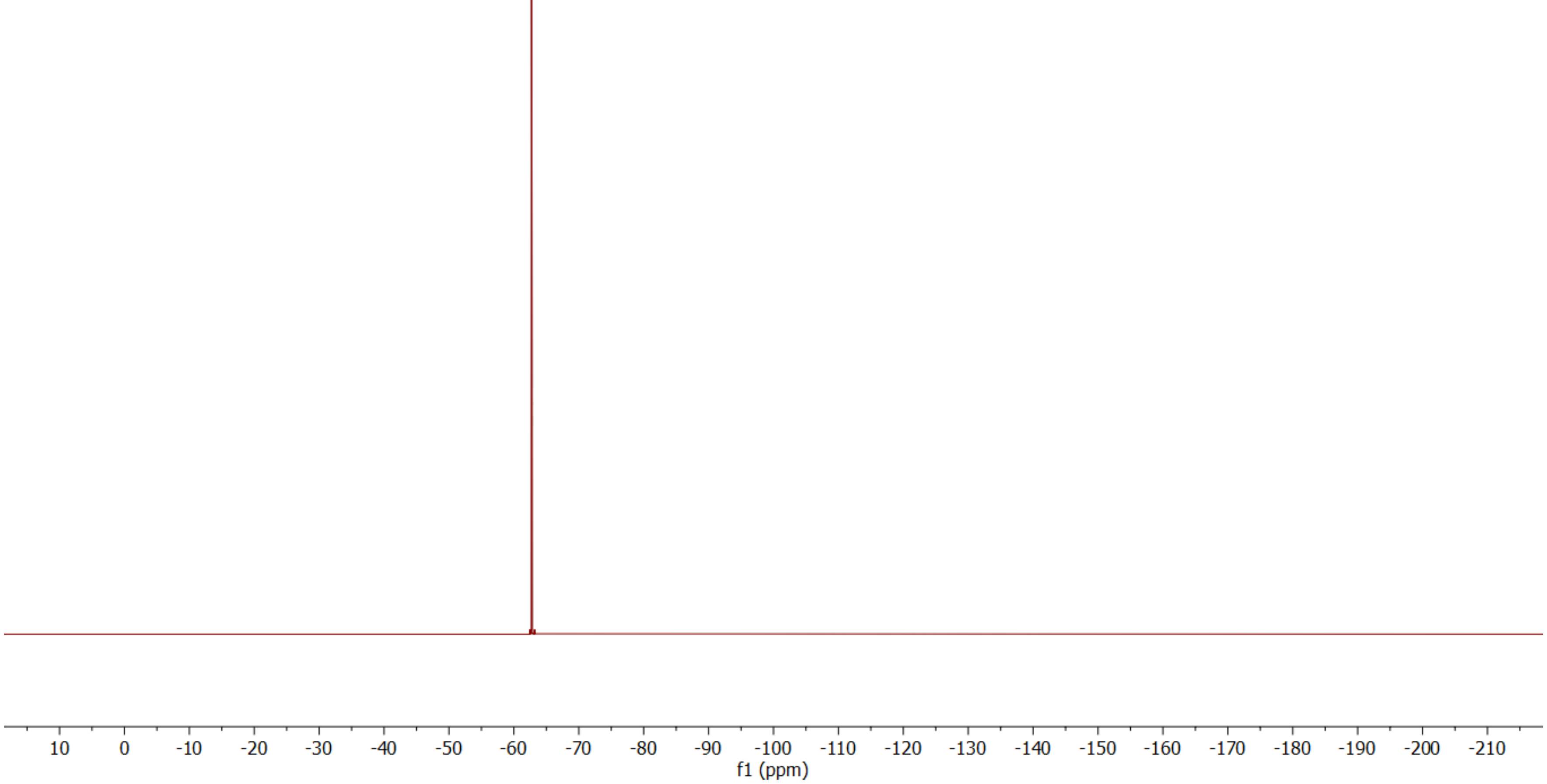

1H (400.15 MHz, CDCl3)

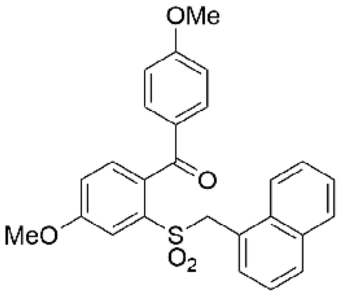

2ac

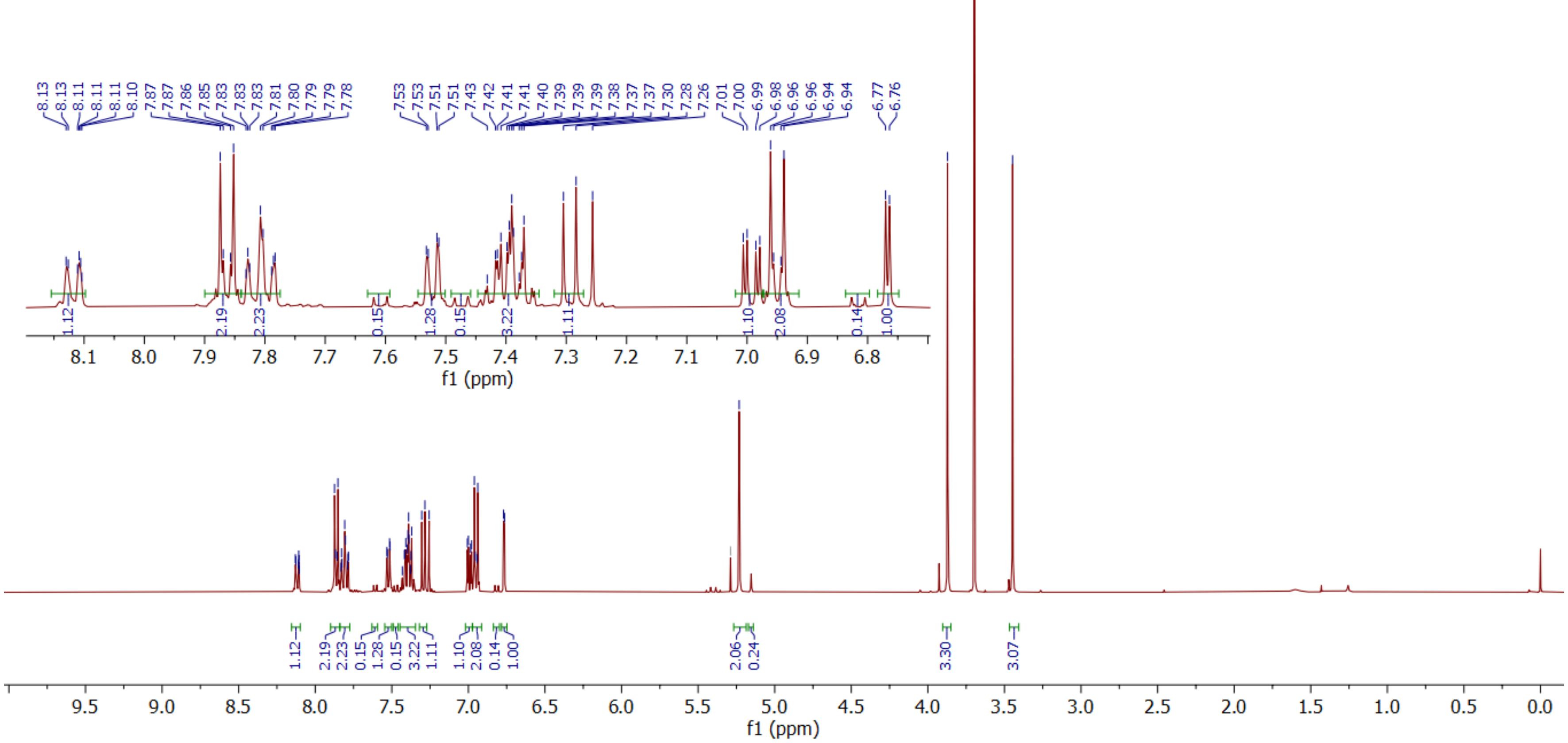

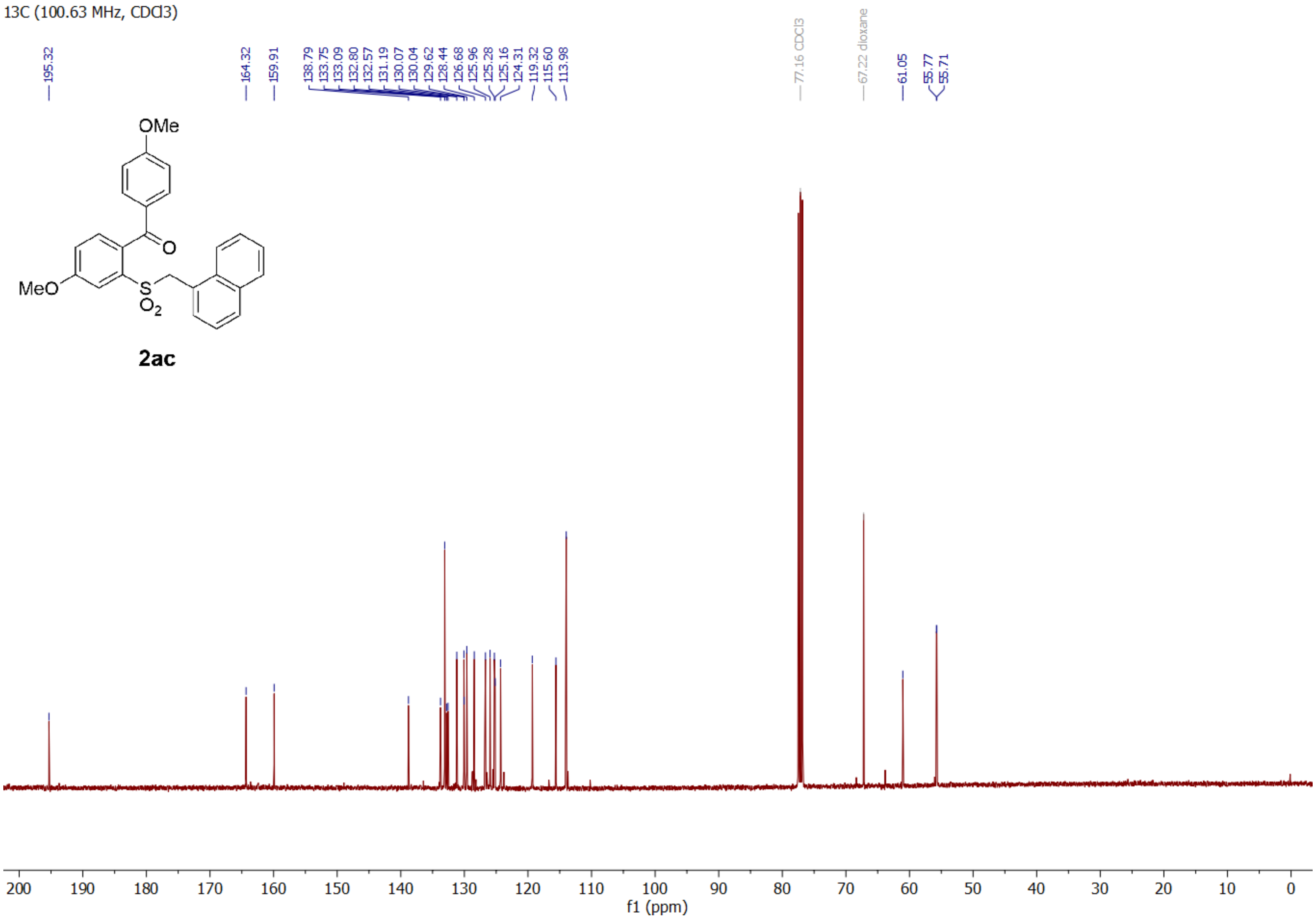
13C (100.63 MHz, CDCl3)
195.32
164.32
159.91
138.79
133.75
133.09
132.80
132.57
131.19
130.07
130.04
129.62
128.44
126.68
125.96
125.28
125.16
124.31
119.32
115.60
113.98
77.16 CDCl3
67.22 dioxane
61.05
55.77
55.71
OMe
O
MeO
S
O2
2ac
200
190
180
170
160
150
140
130
120
110
100
90
80
70
60
50
40
30
20
10
0
f1 (ppm)

1H (400.15 MHz, CDCl3)

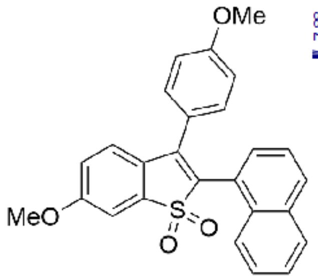

3ac

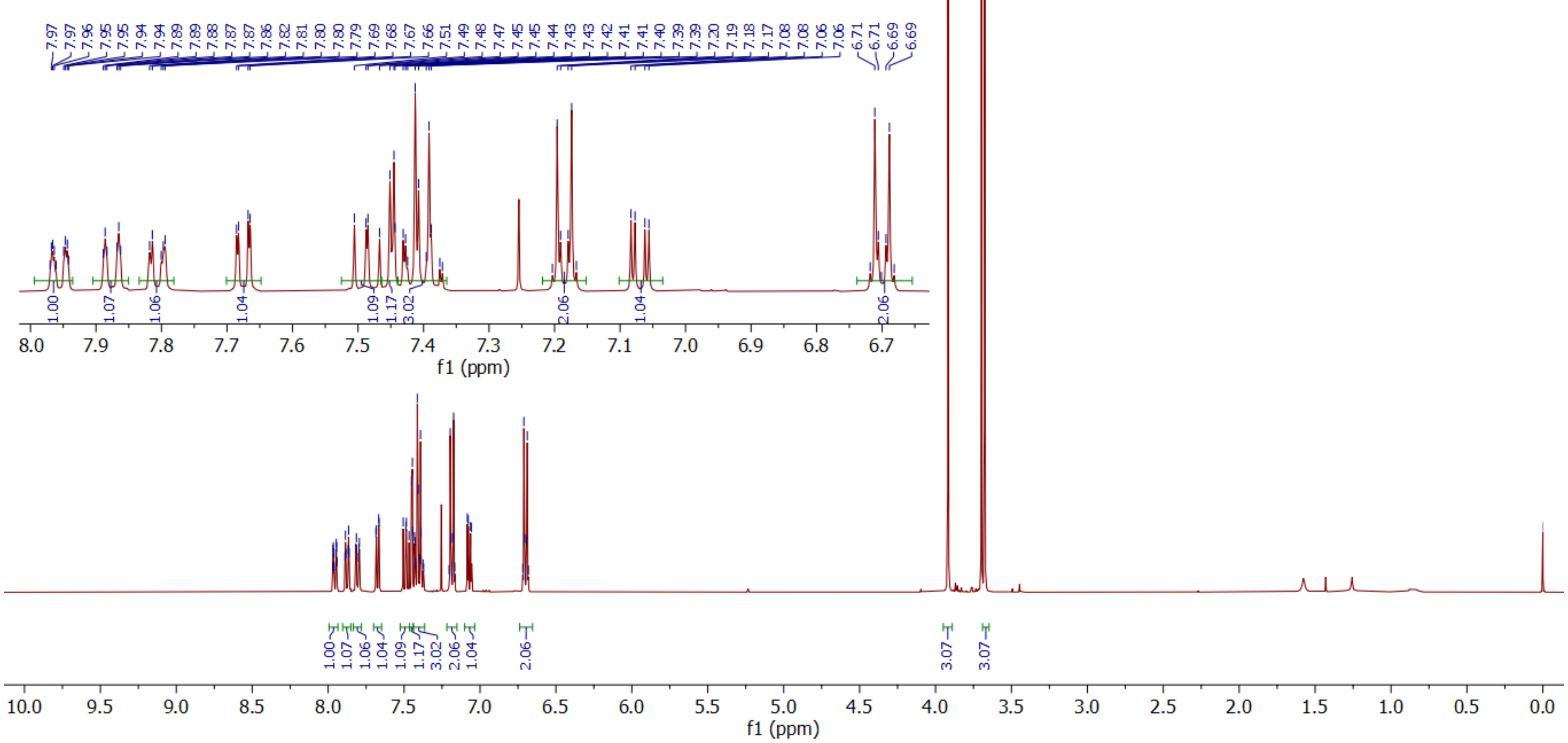

13C (100.63 MHz, CDCl3)

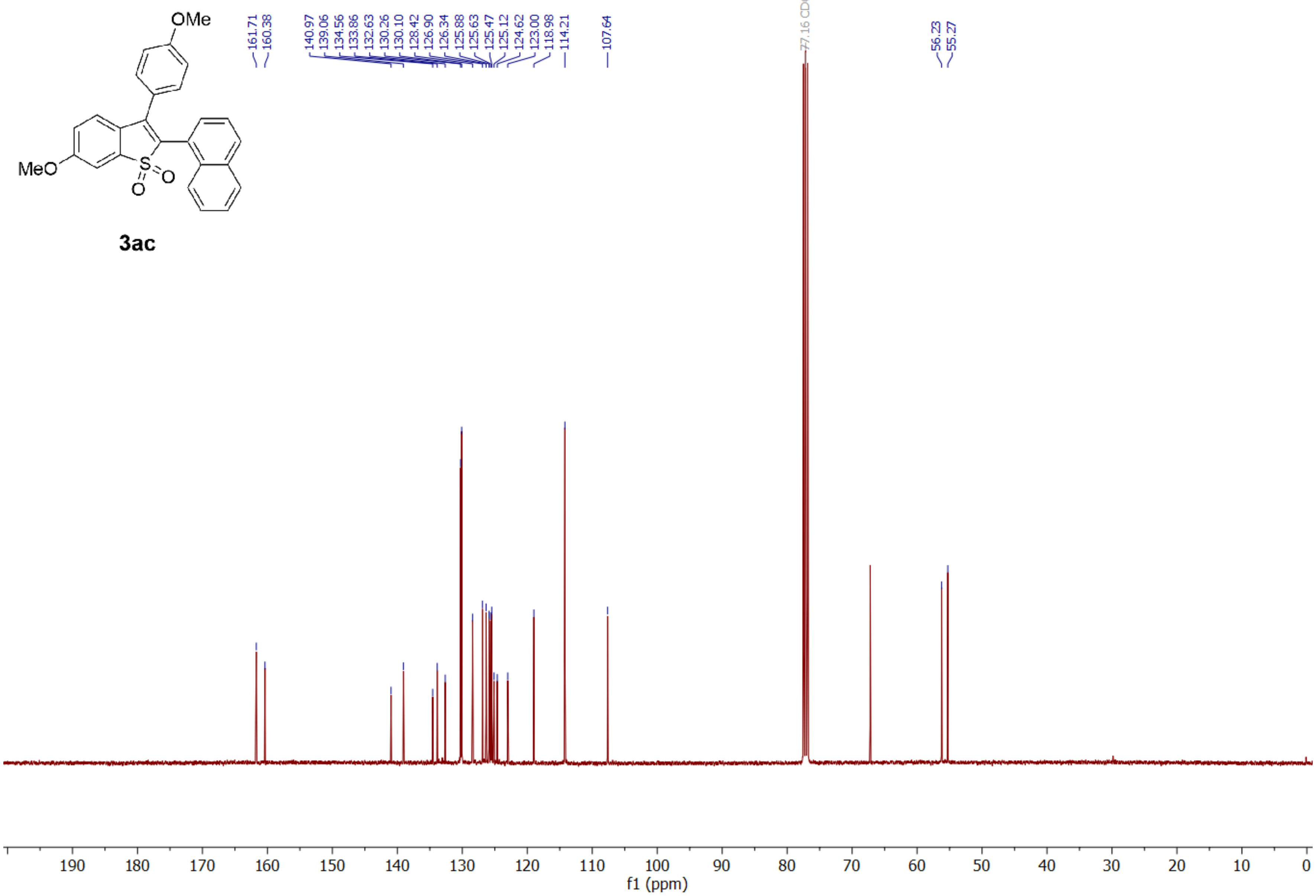

1H (400.15 MHz, CDCl3)

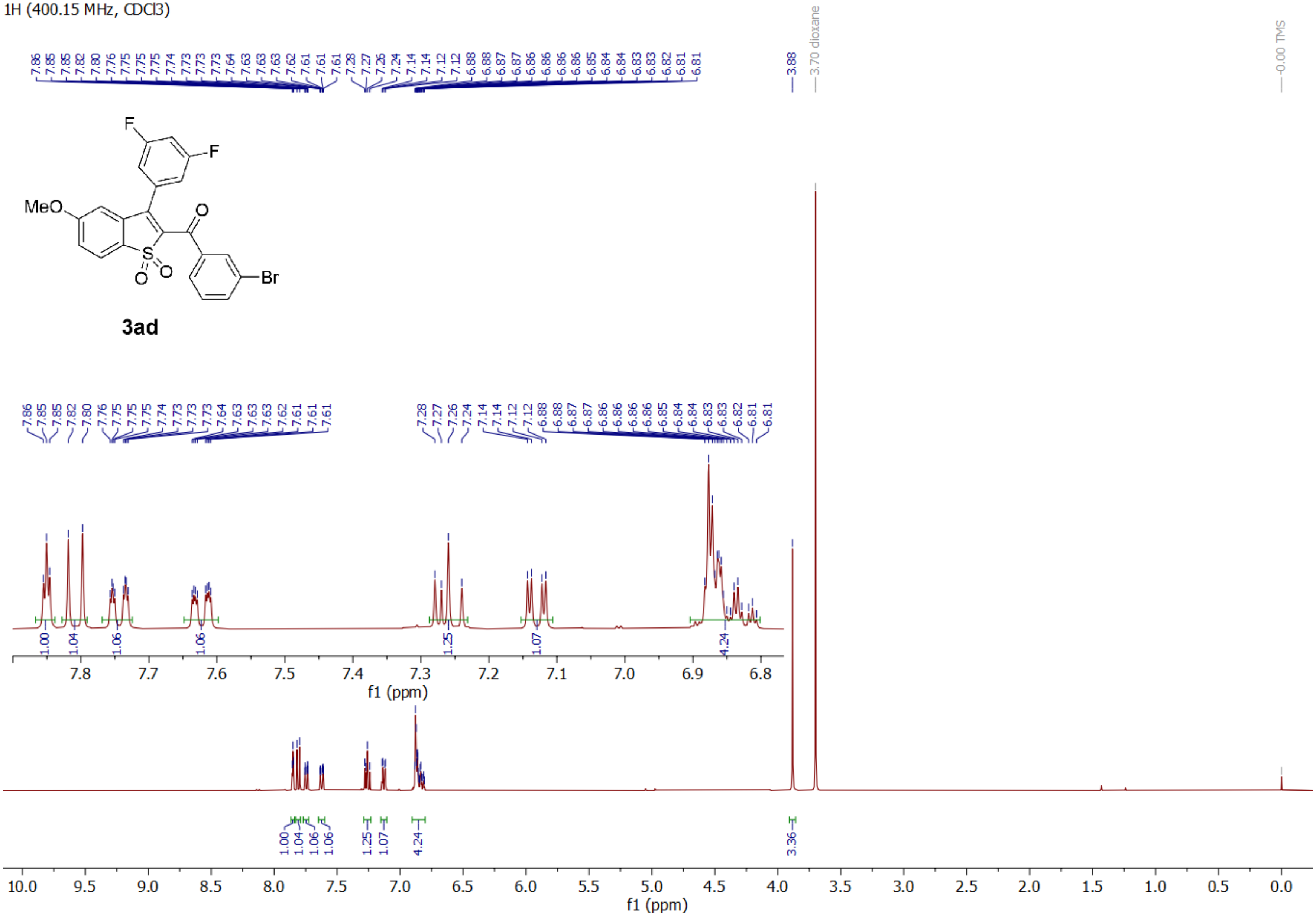

3ad

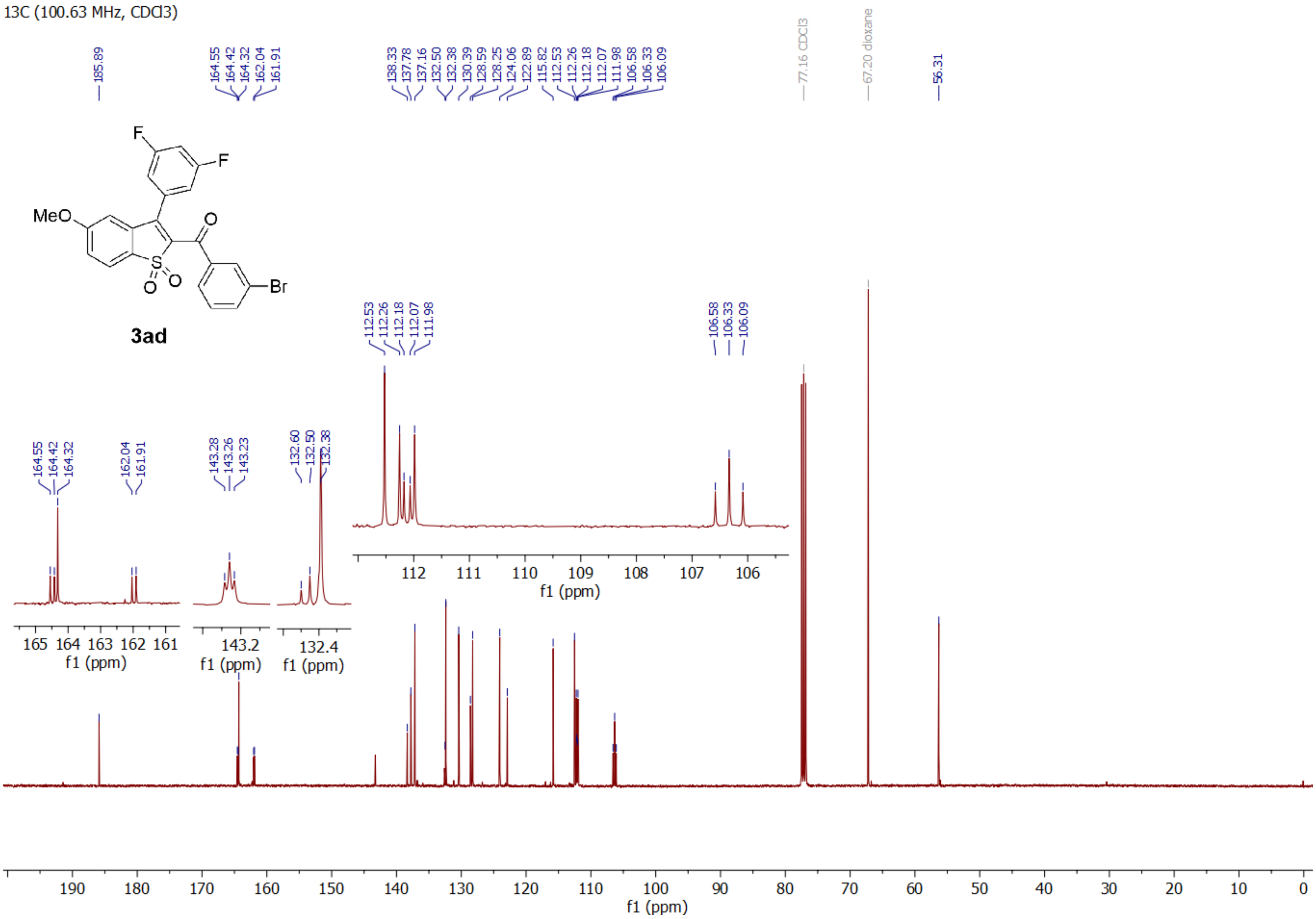

13C (100.63 MHz, CDCl3)
185.89
164.55
164.42
164.32
162.04
161.91
138.33
137.78
137.16
132.50
132.38
130.39
128.59
128.25
124.06
122.89
115.82
112.53
112.26
112.18
112.07
111.98
106.58
106.33
106.09
77.16 CDCl3
67.20 dioxane
56.31
MeO
F
O
S
Br
143.28
143.26
143.23
132.60
f1 (ppm)

3ad

19F (376.48 MHz, CDCl3)

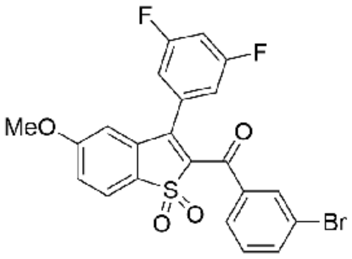

3ad

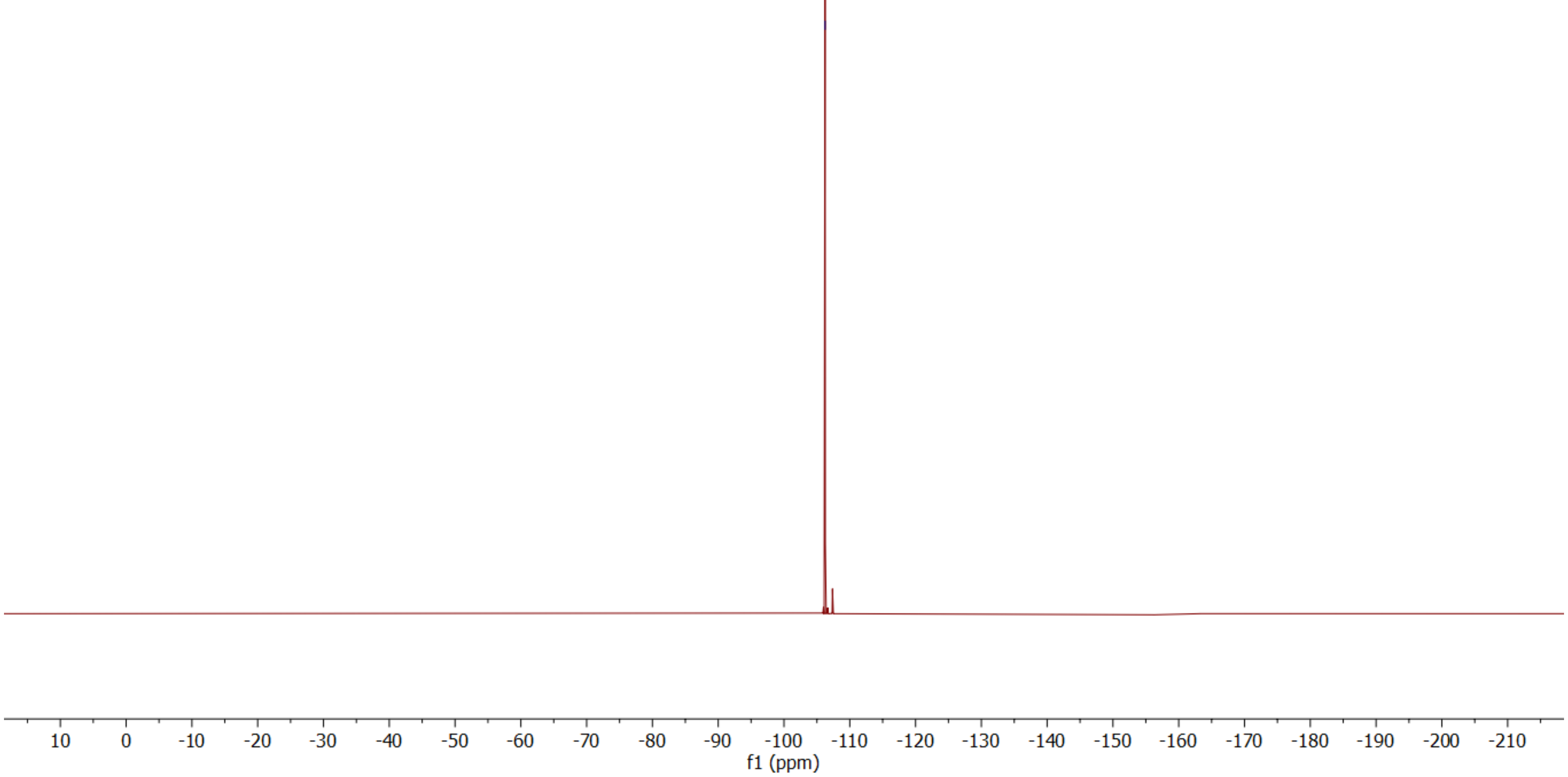

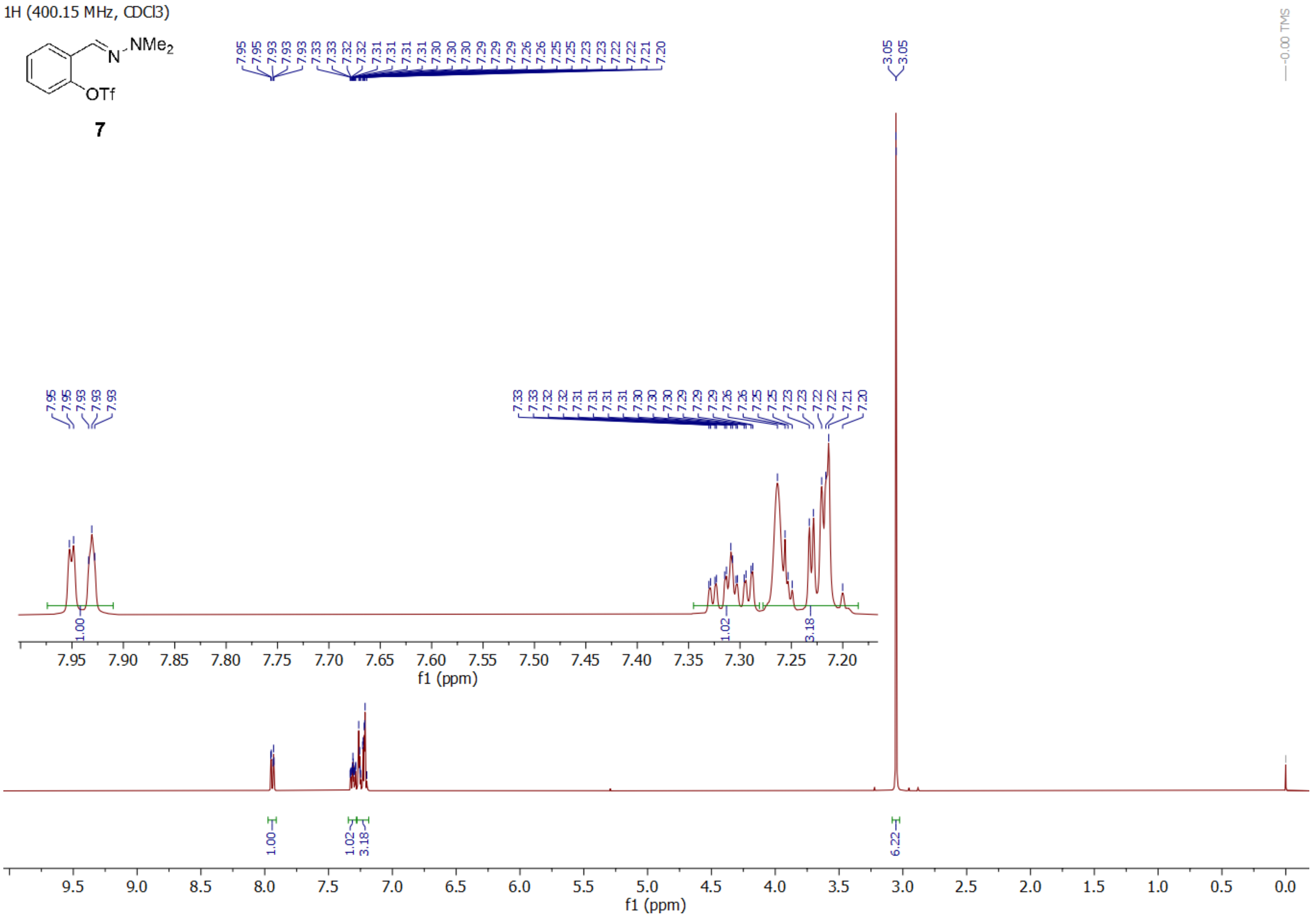
1H (400.15 MHz, CDCl3)
NMe2
N
OTf
7
f1 (ppm)
f1 (ppm)
TMS

19F (376.48 MHz, CDCl3)

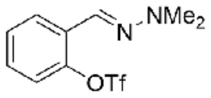

7

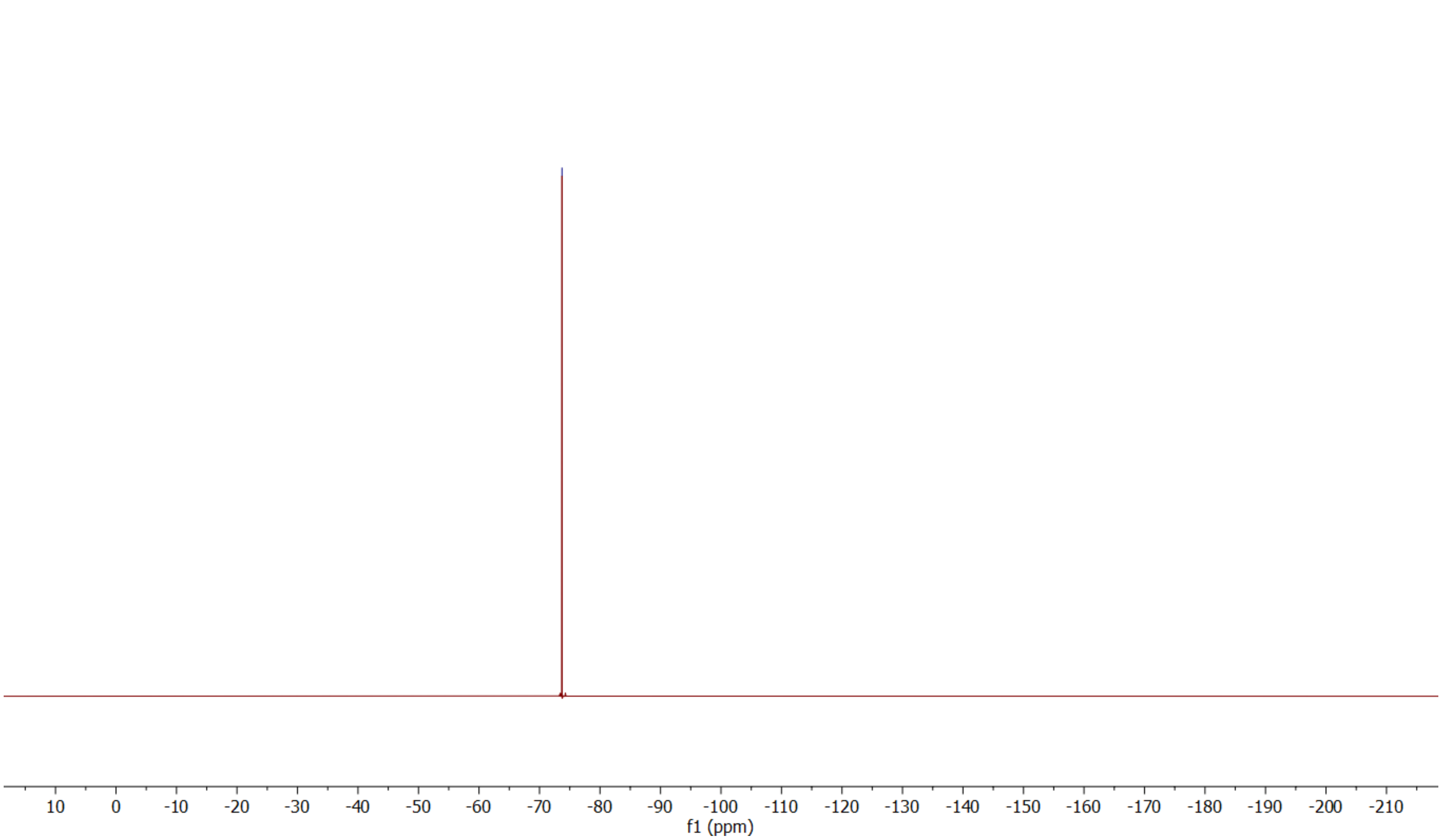

13C (100.63 MHz, CDCl3)

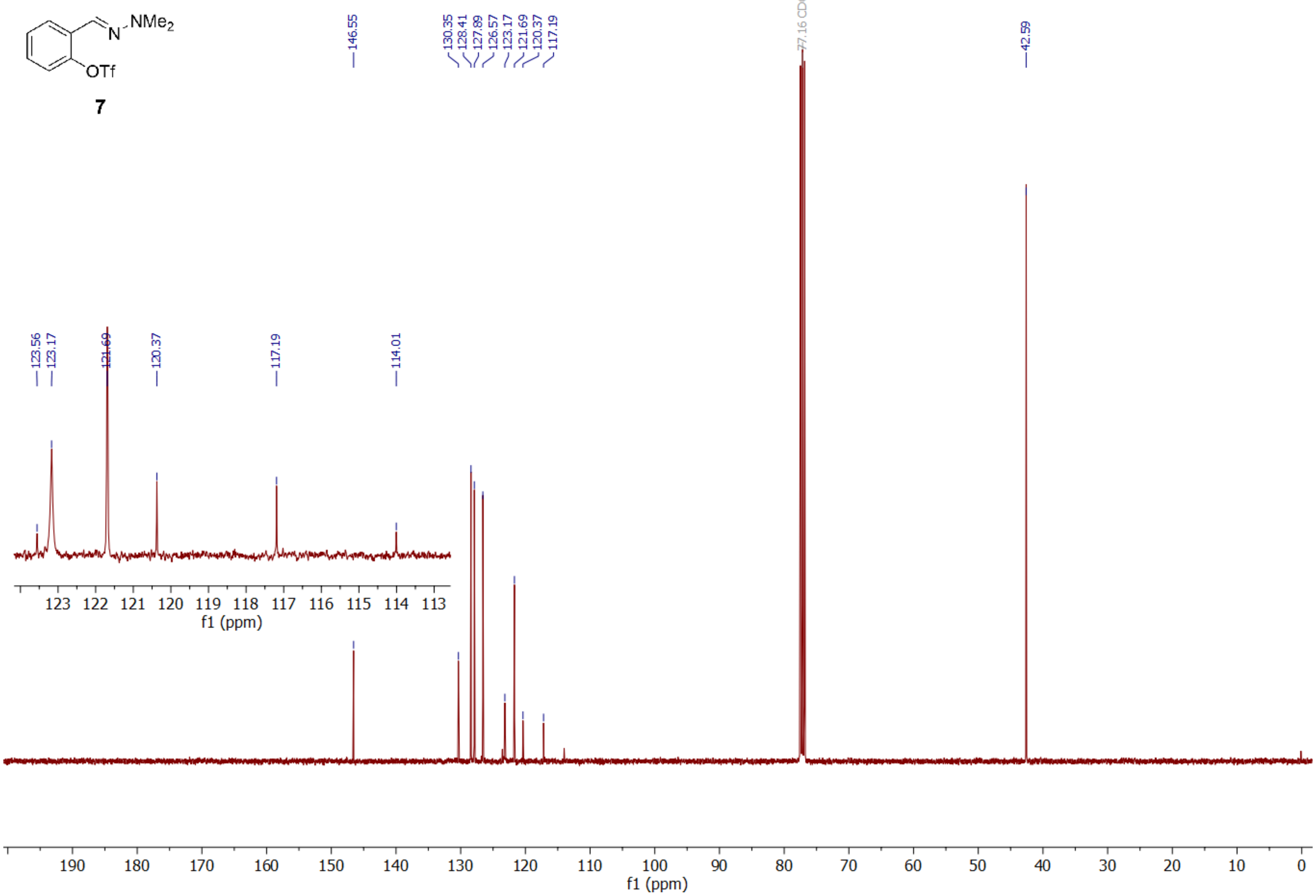

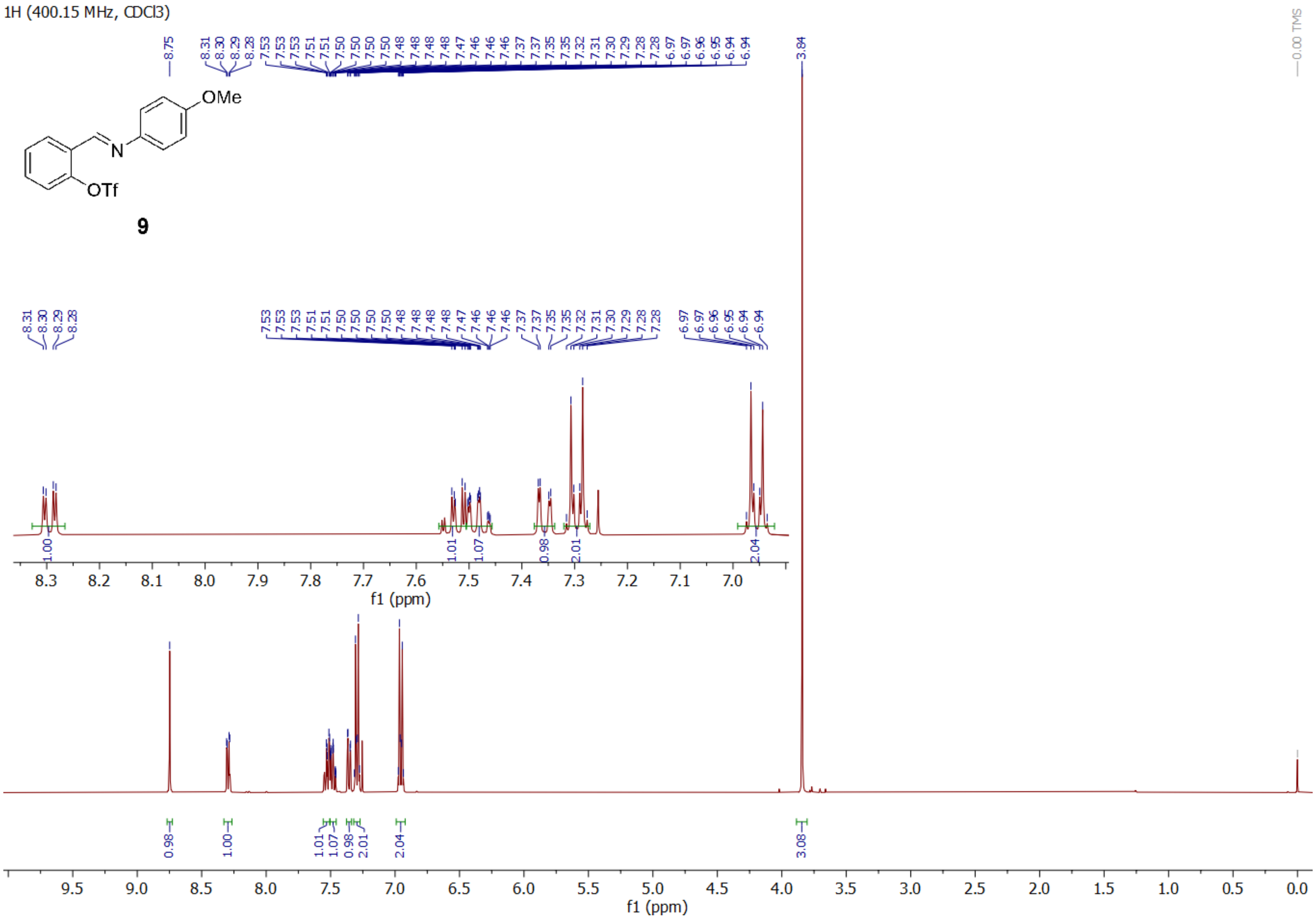
1H (400.15 MHz, CDCl3)
OMe
N
OTf
9
f1 (ppm)

13C (100.63 MHz, CDCl3)

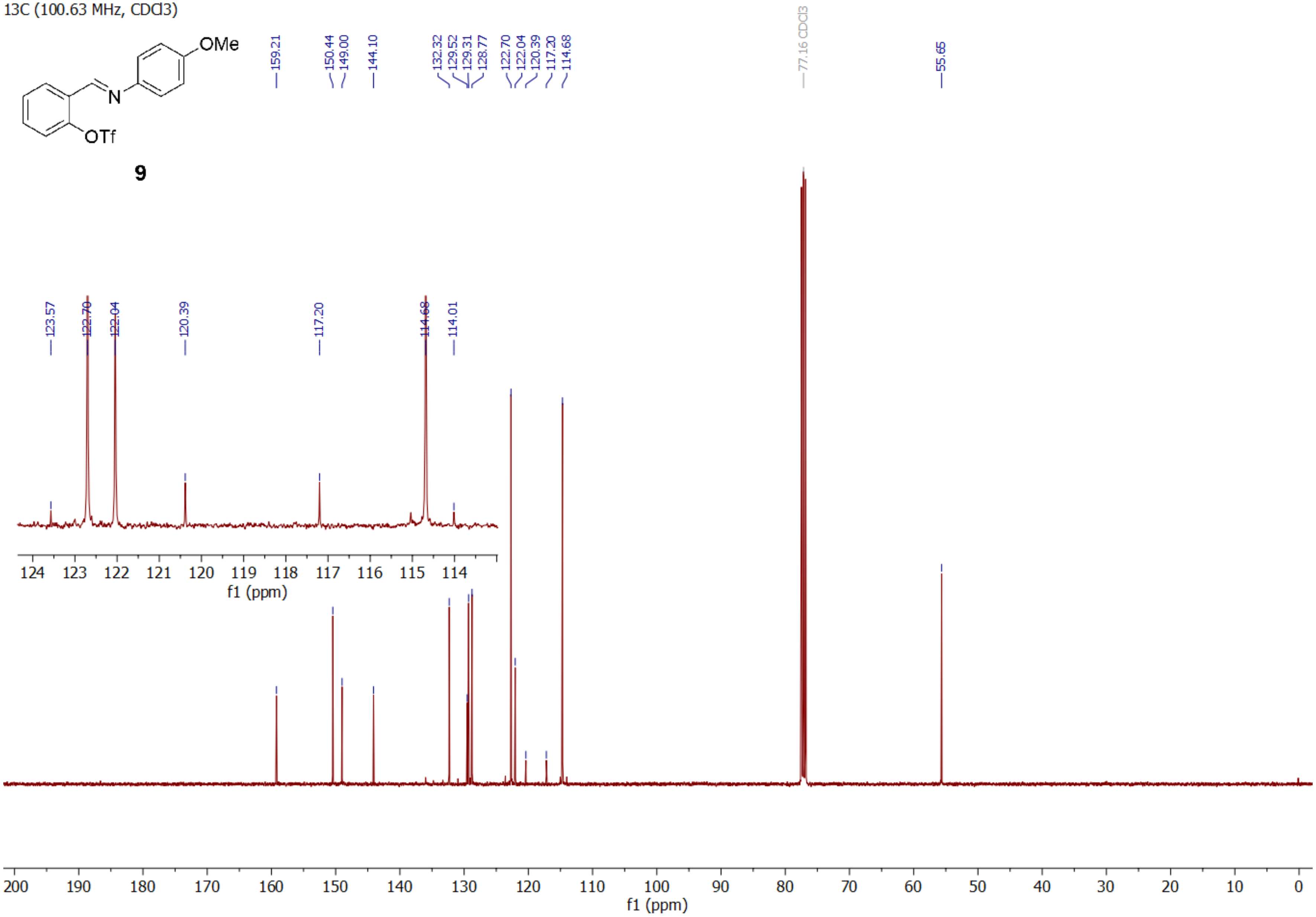

19F (376.48 MHz, CDCl3)

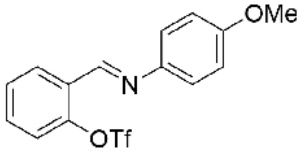

9

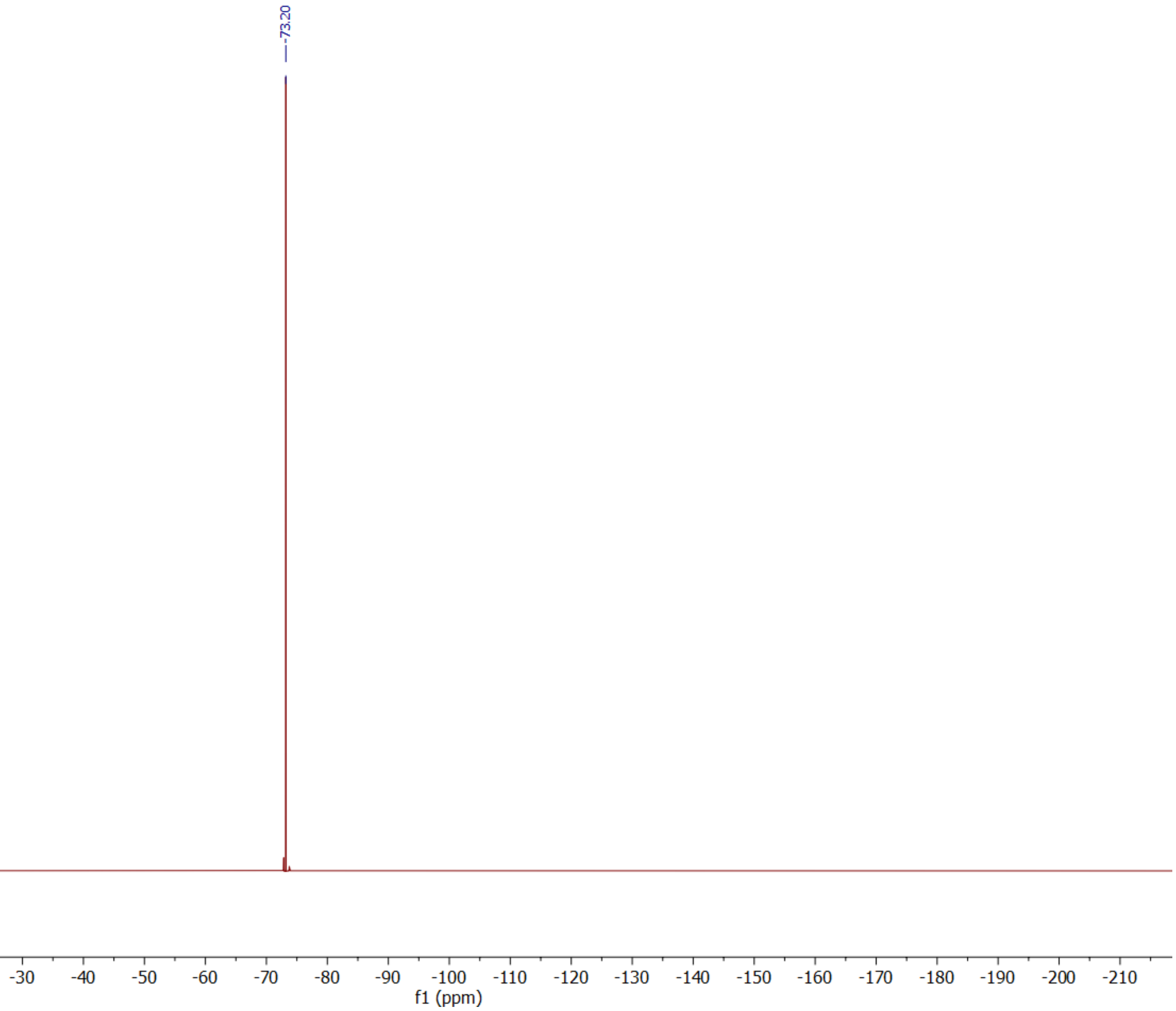

1H (400.15 MHz, CDCl3)

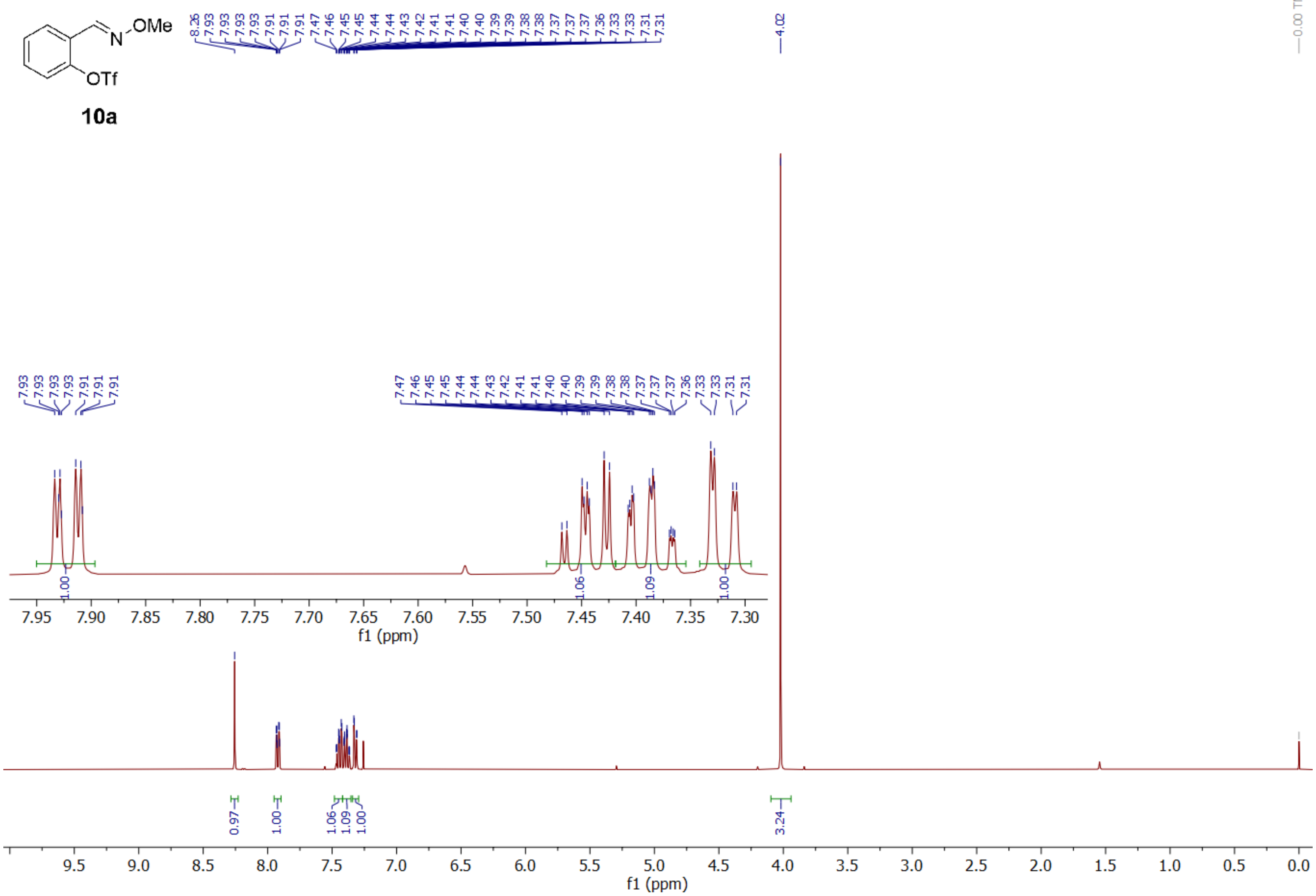

19F (376.48 MHz, CDCl3)

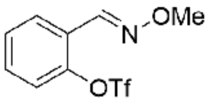

10a

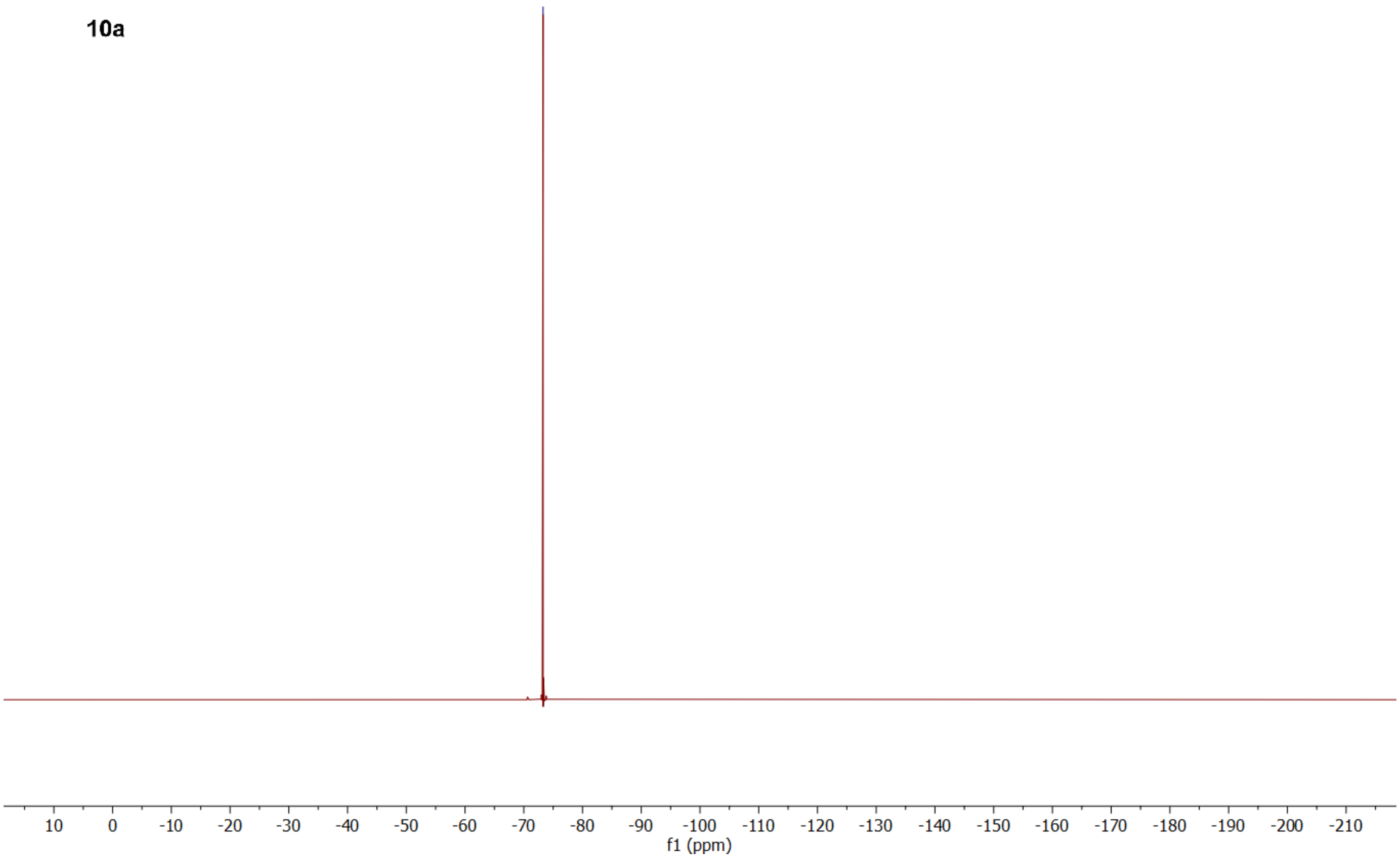

13C (100.63 MHz, CDCl3)

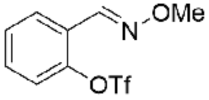

10a

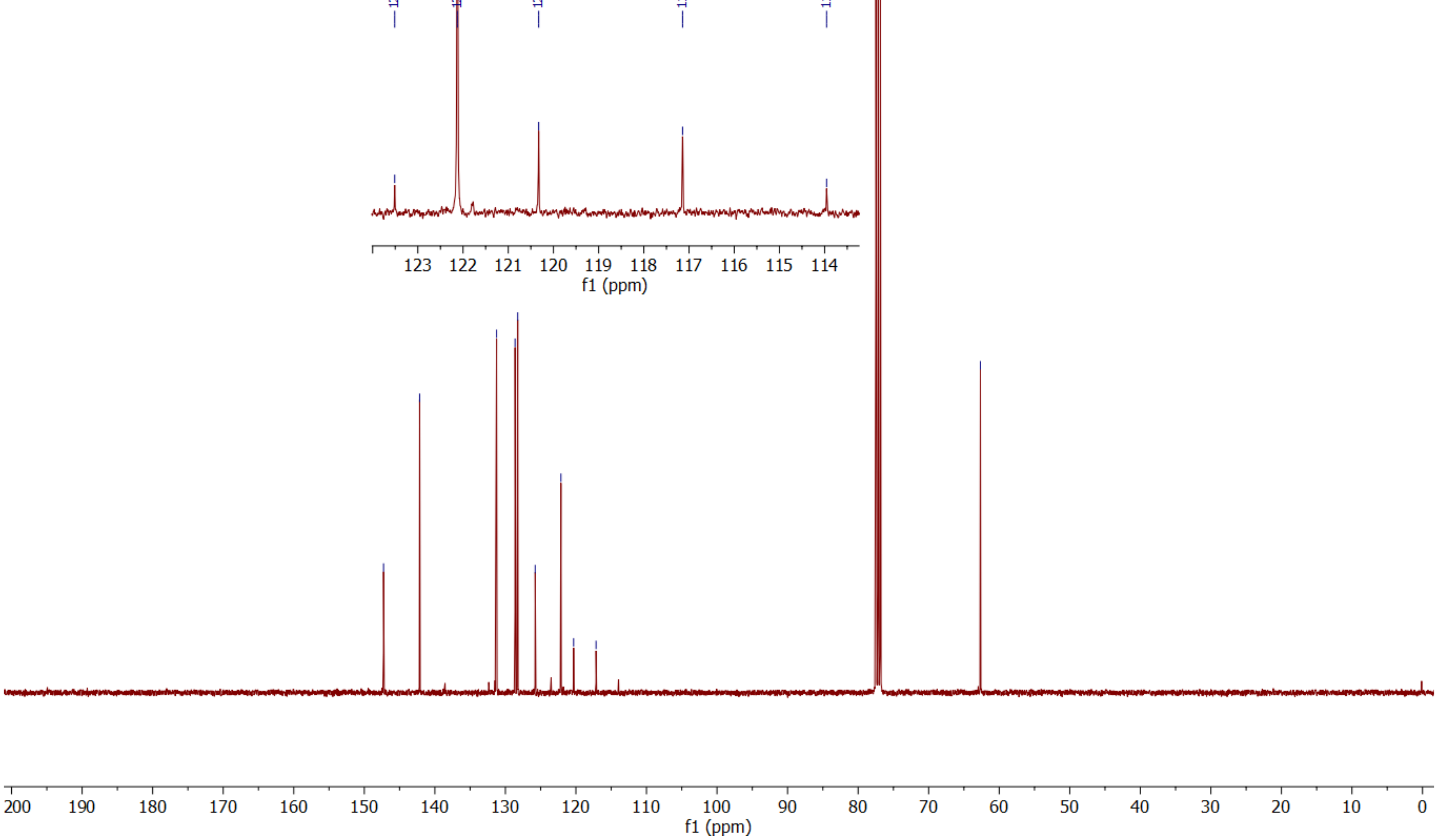

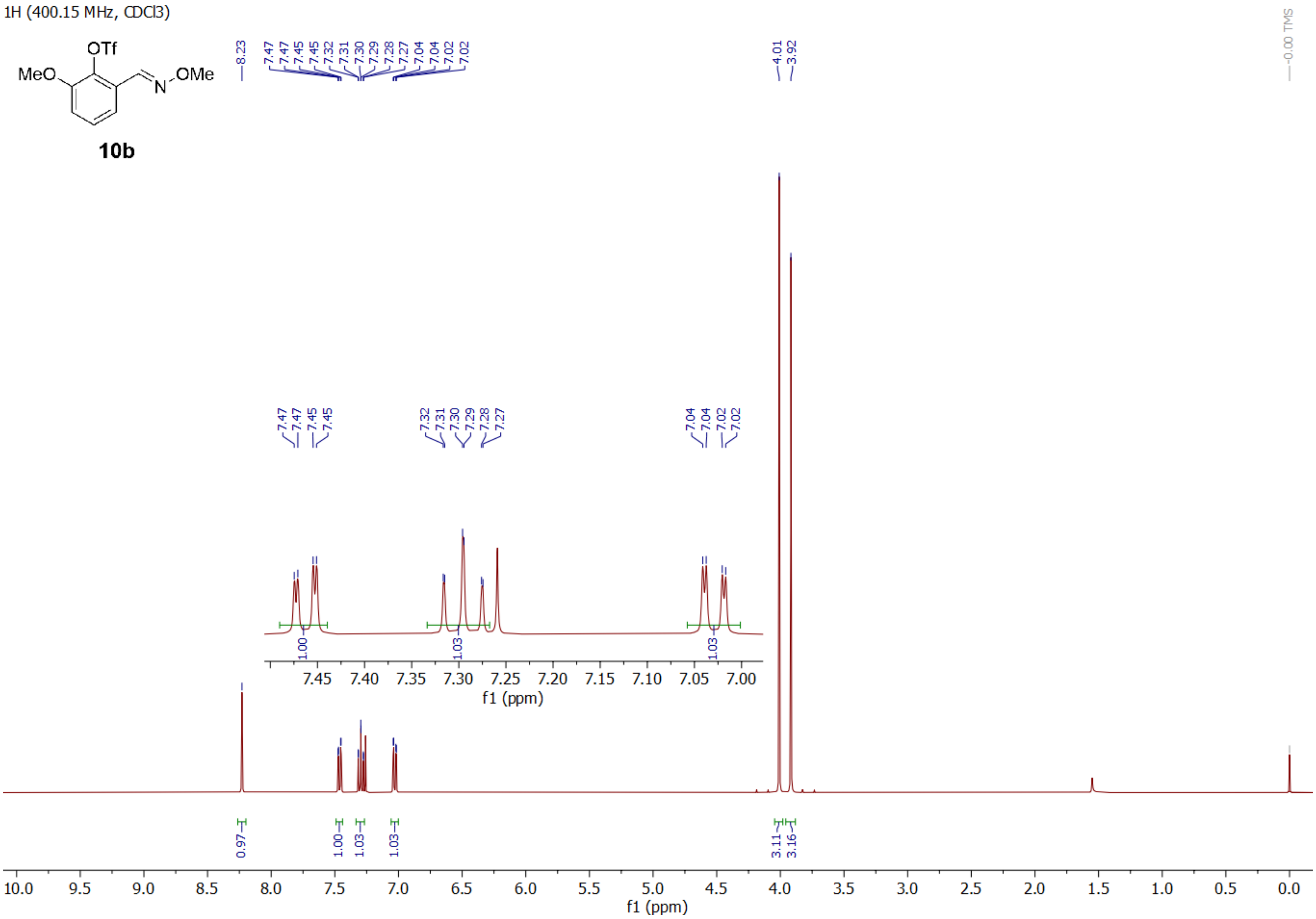

1H (400.15 MHz, CDCl3)
OTf
MeO
N
OMe
10b
8.23
7.47
7.47
7.45
7.45
7.32
7.31
7.30
7.29
7.28
7.27
7.04
7.04
7.02
7.02
4.01
3.92
0.00 TMS
0.97
1.00
1.03
1.03
3.11
3.16
f1 (ppm)

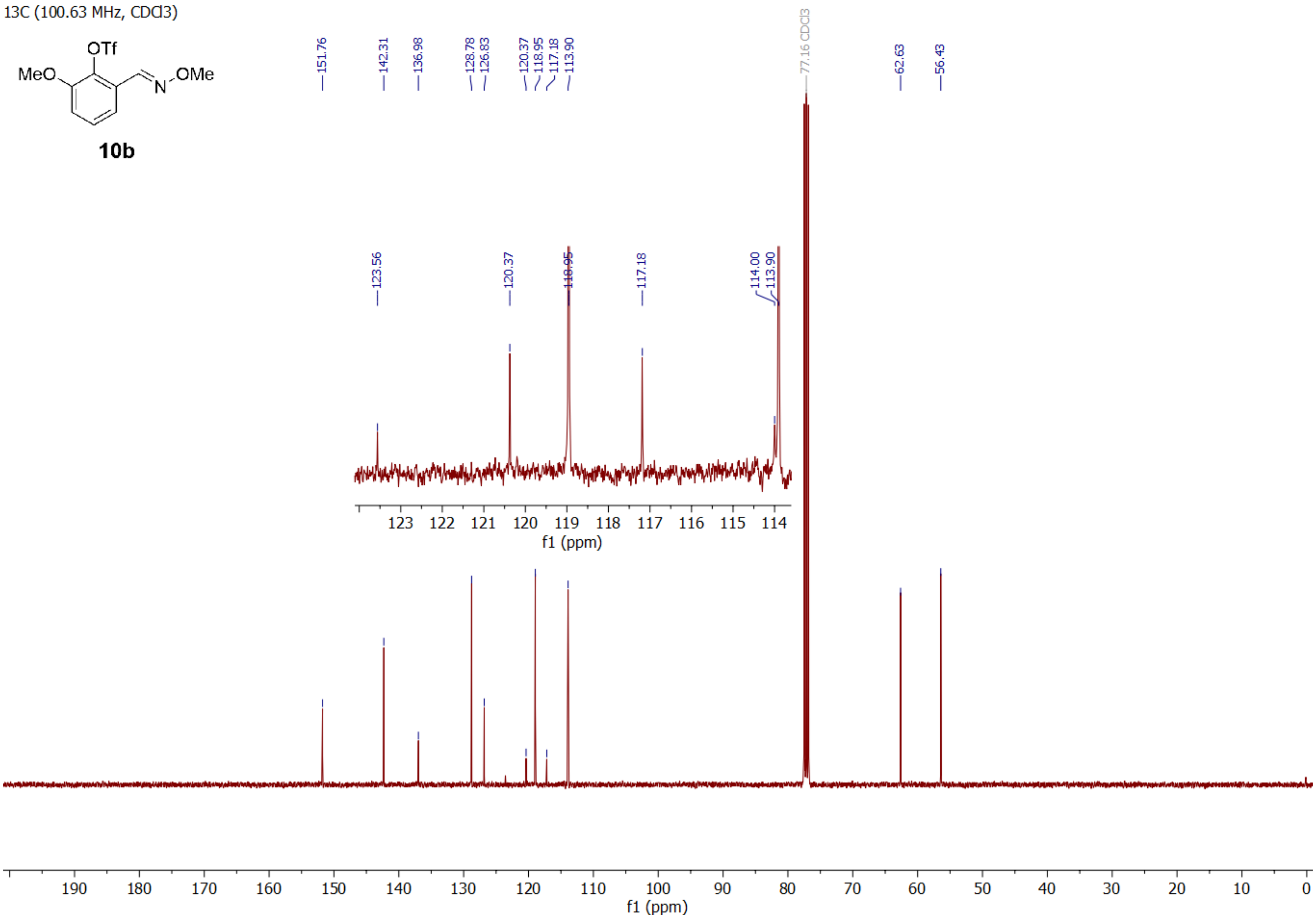
13C (100.63 MHz, CDCl3)
OTf
MeO
N
OMe
10b
151.76
142.31
136.98
128.78
126.83
120.37
118.95
117.18
113.90
77.16 CDCl3
62.63
56.43
123.56
120.37
118.95
117.18
114.00
113.90
f1 (ppm)
f1 (ppm)

19F (376.48 MHz, CDCl3)

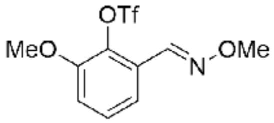

10b

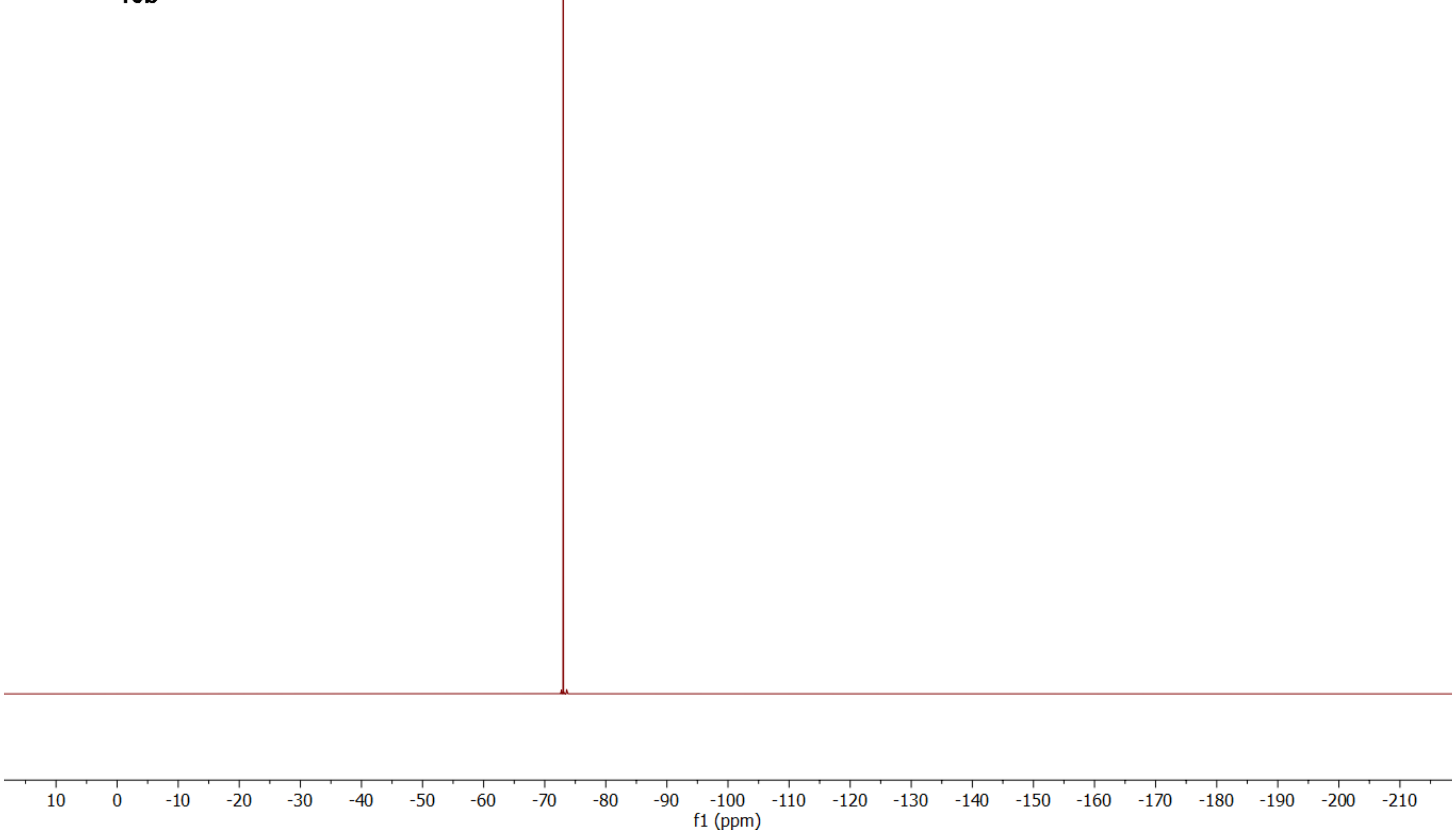

1H (400.15 MHz, CDCl3)

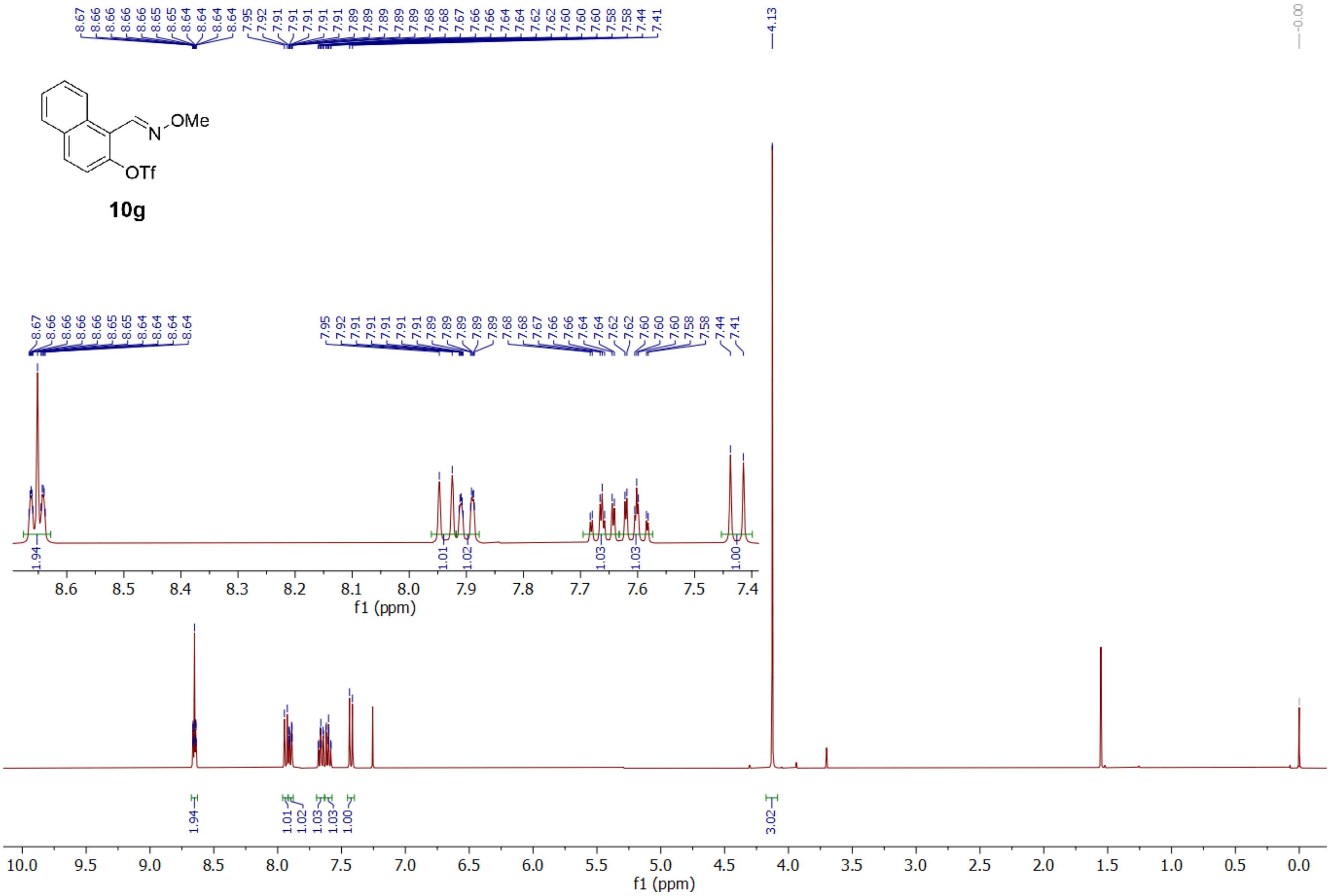

13C (100.63 MHz, CDCl3)

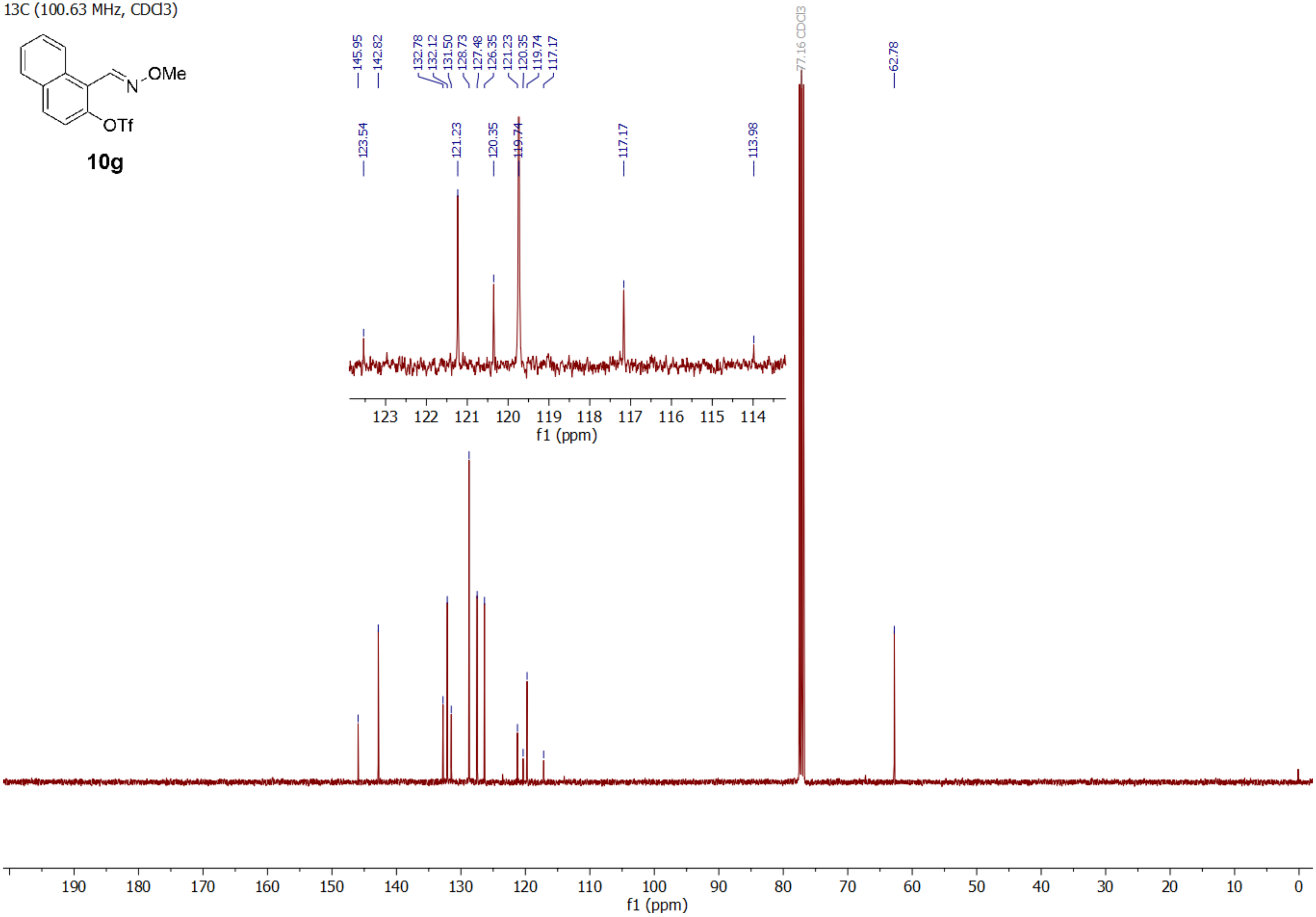

10g

19F (376.48 MHz, CDCl3)

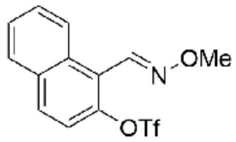

10g

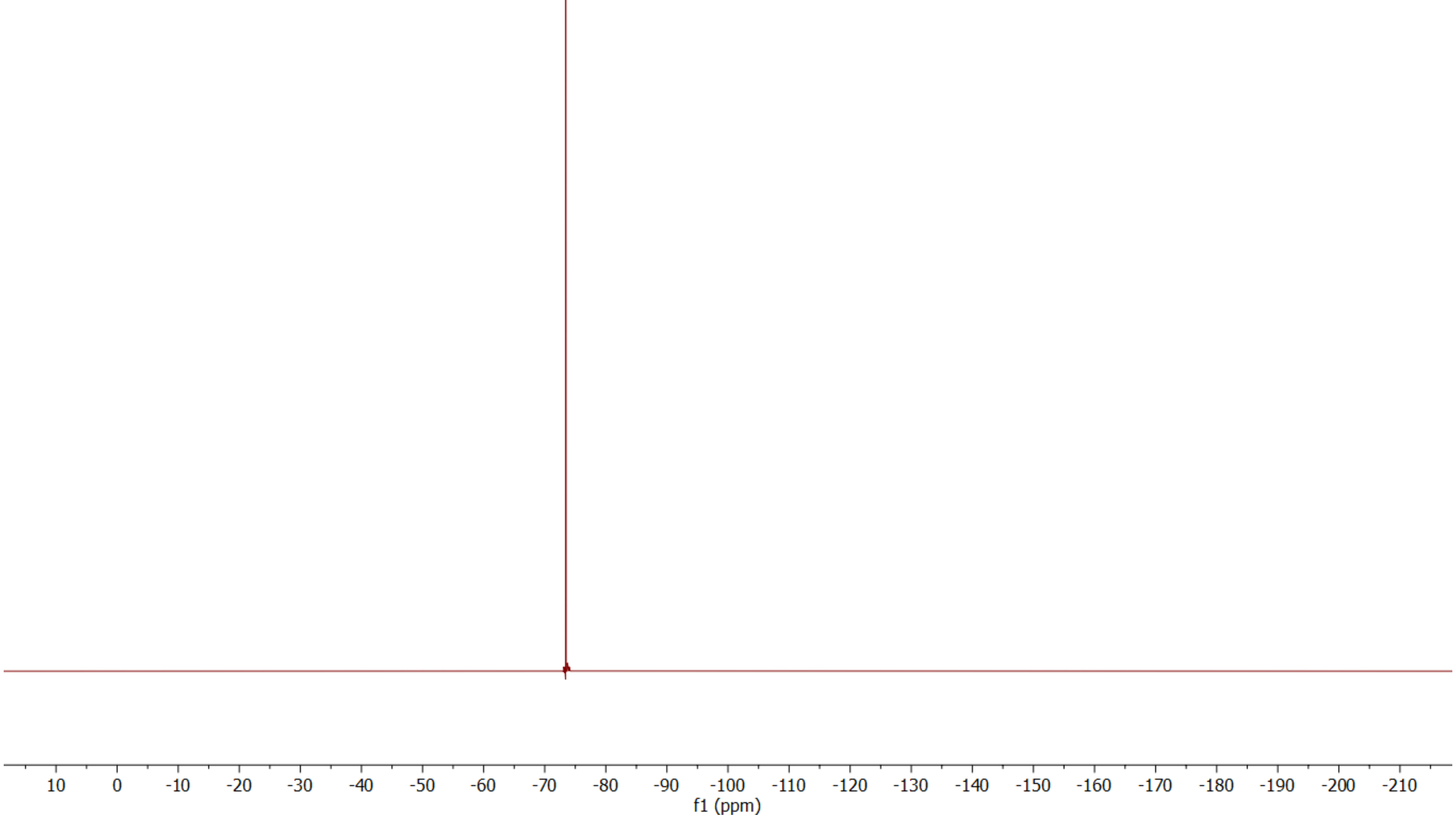

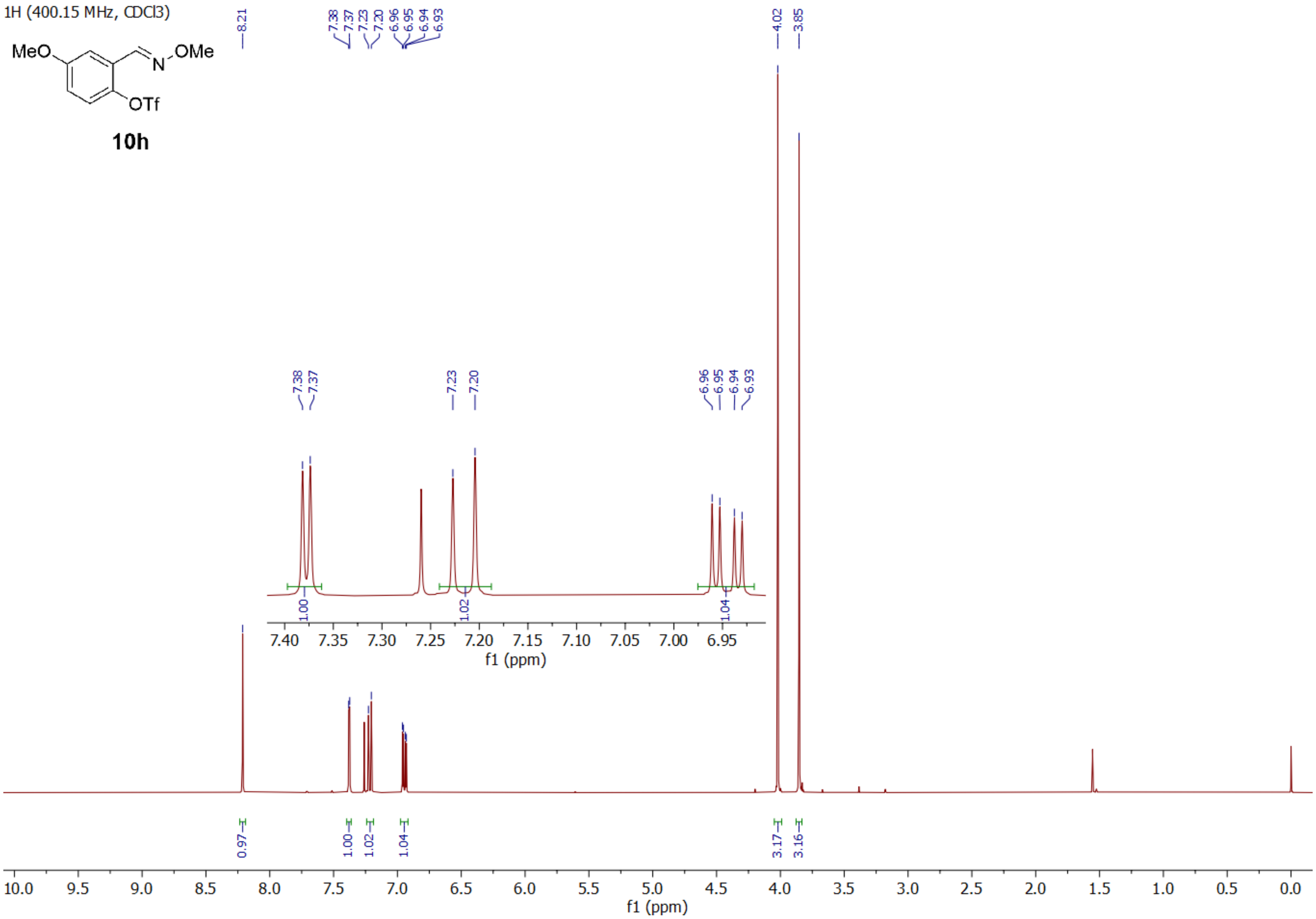
1H (400.15 MHz, CDCl3)
MeO
N
OMe
OTf
10h
8.21
7.38
7.37
7.23
7.20
6.96
6.95
6.94
6.93
4.02
3.85
0.97
1.00
1.02
1.04
3.17
3.16
f1 (ppm)

13C (100.63 MHz, CDCl3)

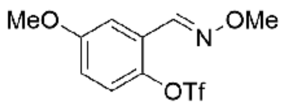

10h

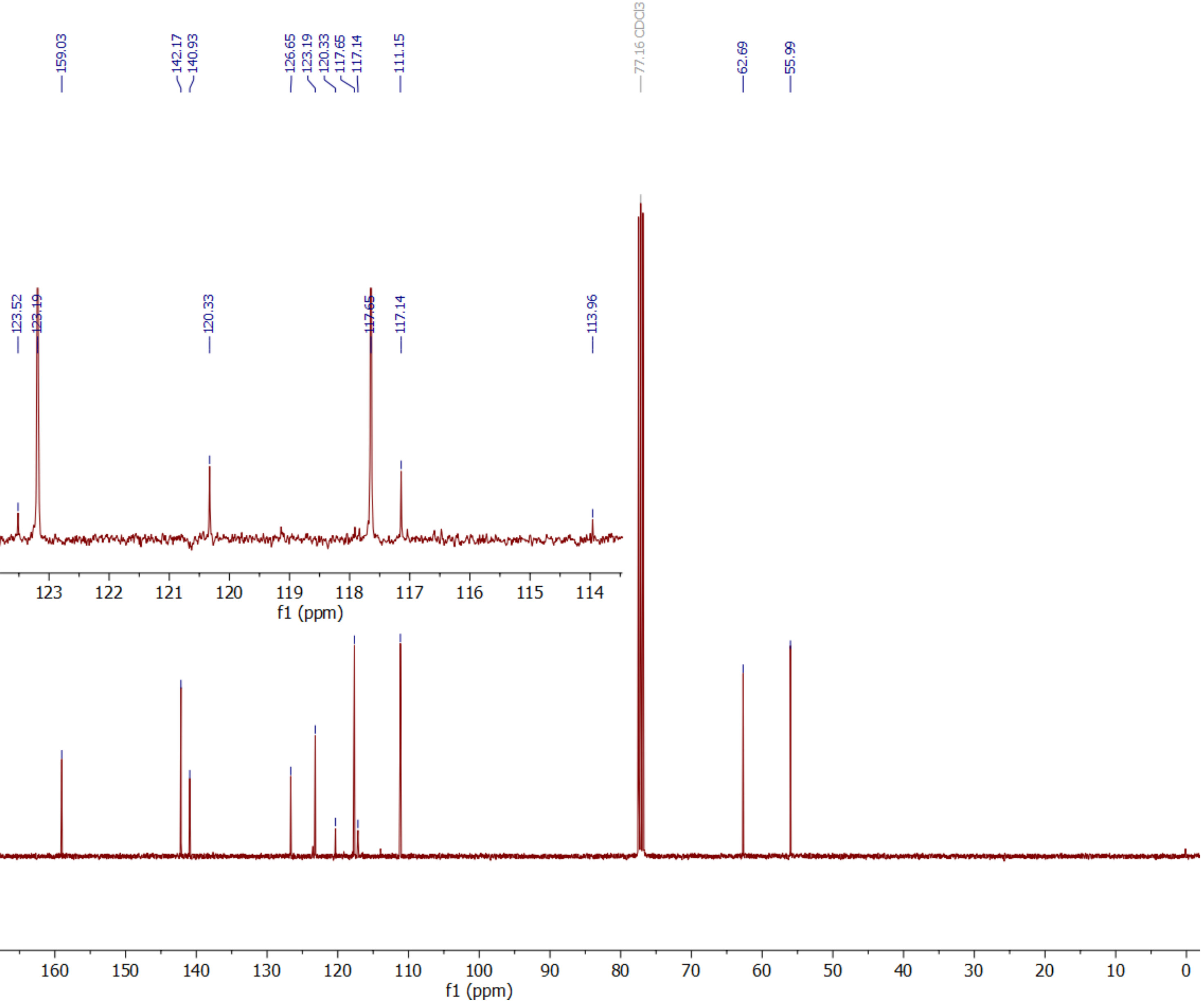

19F (376.48 MHz, CDCl3)

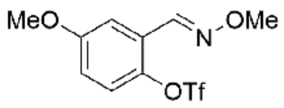

10h

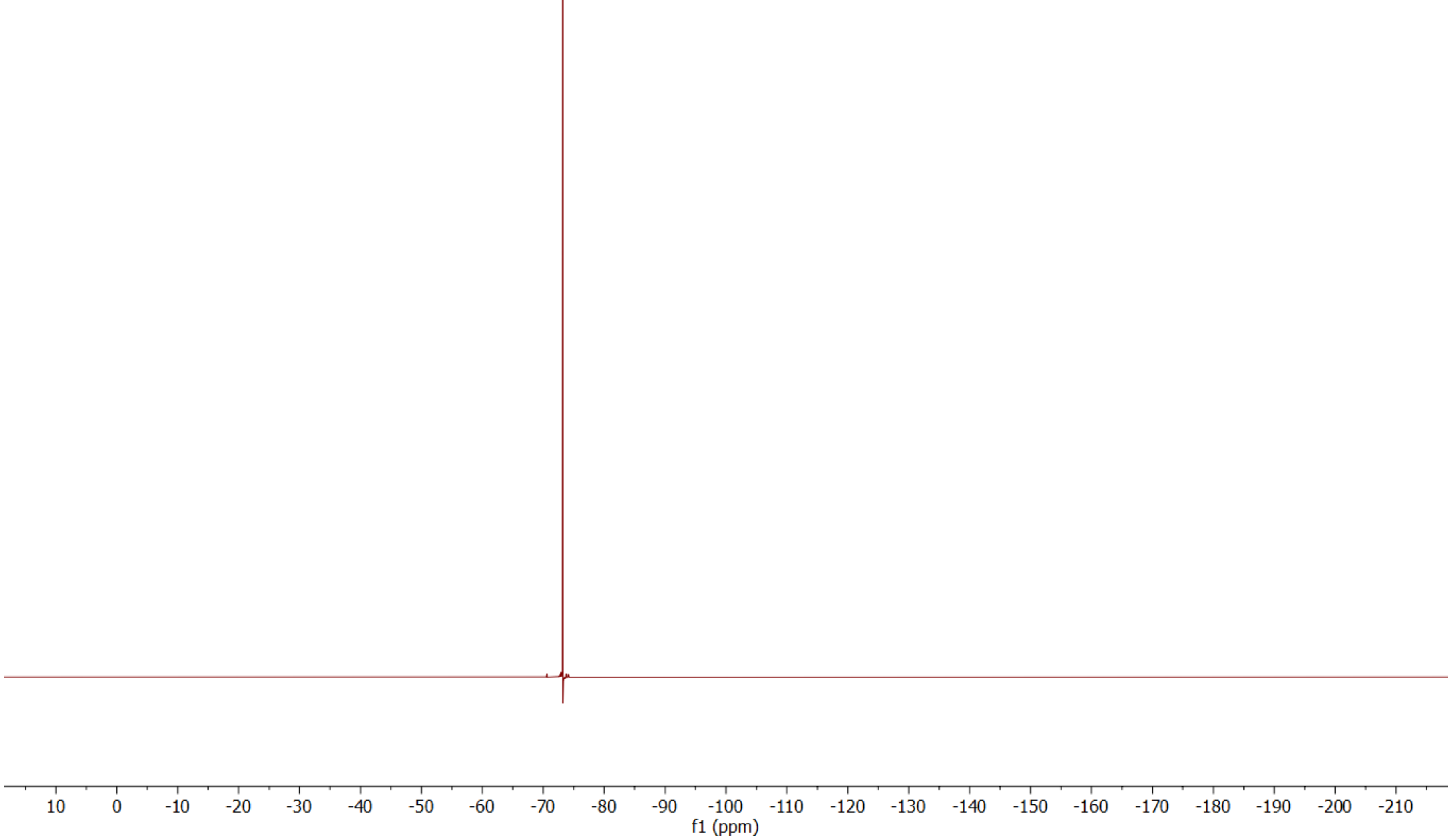

1H (400.15 MHz, CDCl3)

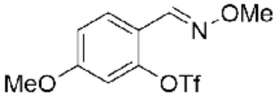

10j

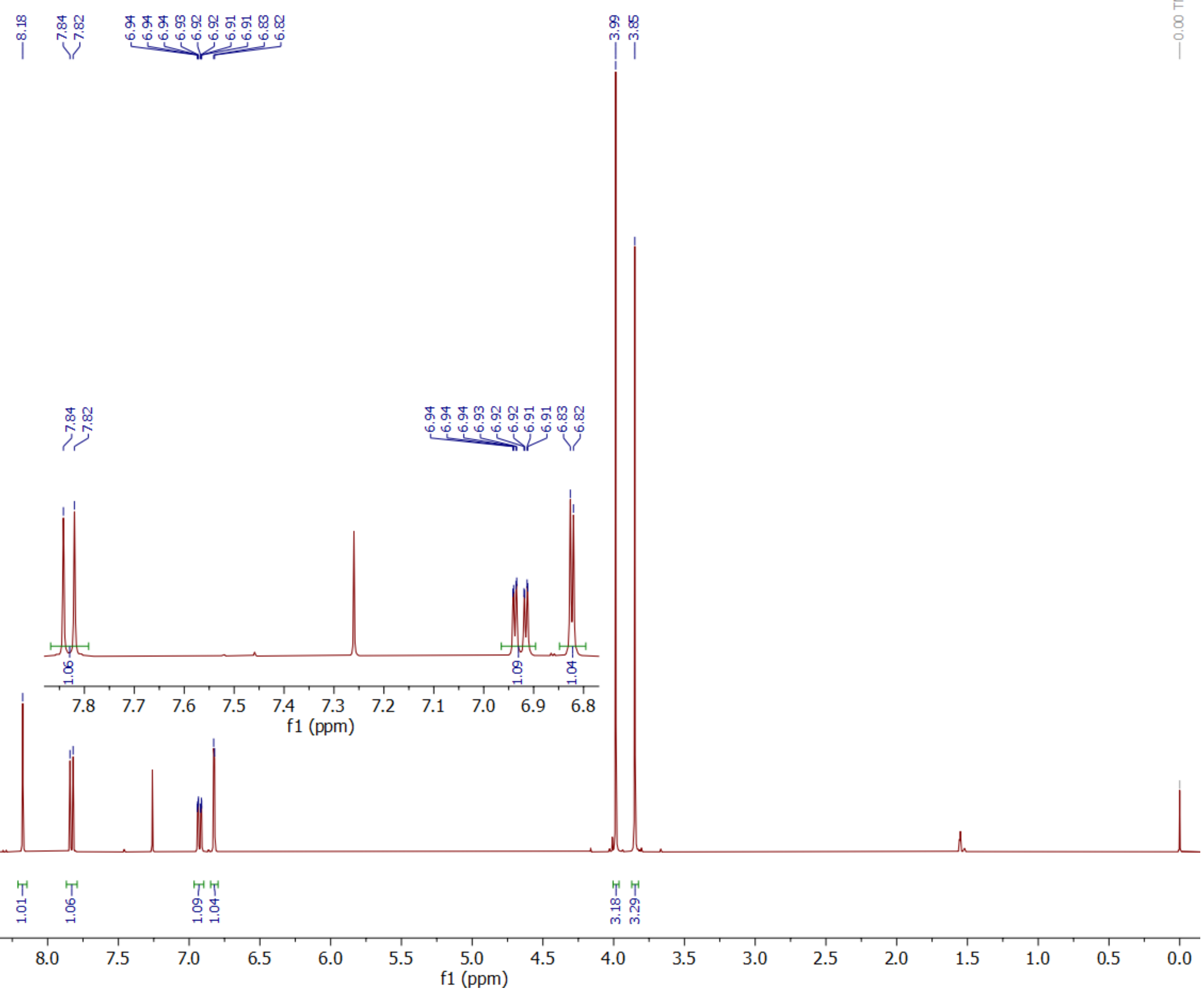

13C (100.63 MHz, CDCl3)

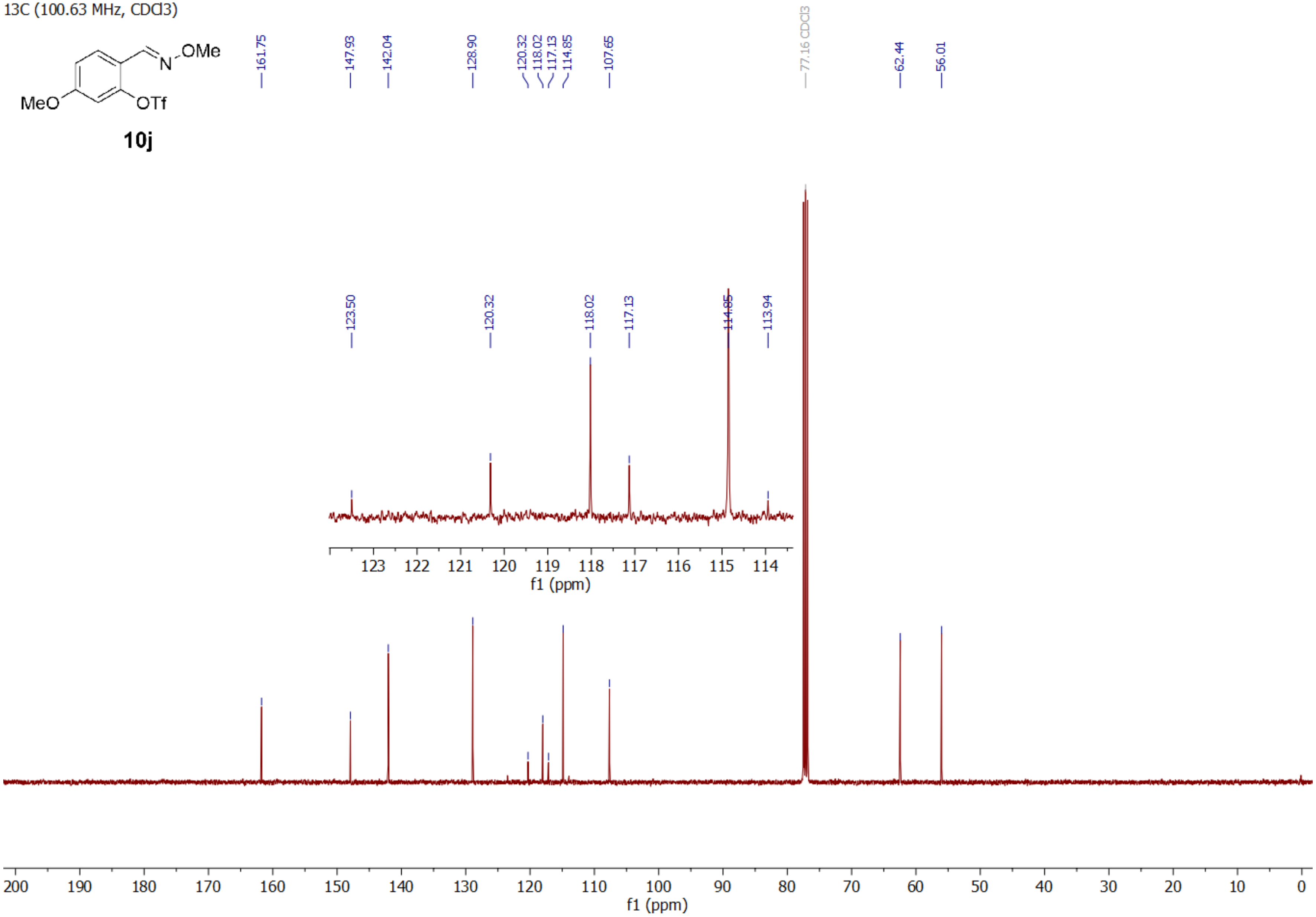

19F (376.48 MHz, CDCl3)

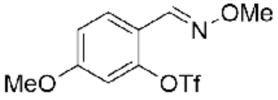

10j

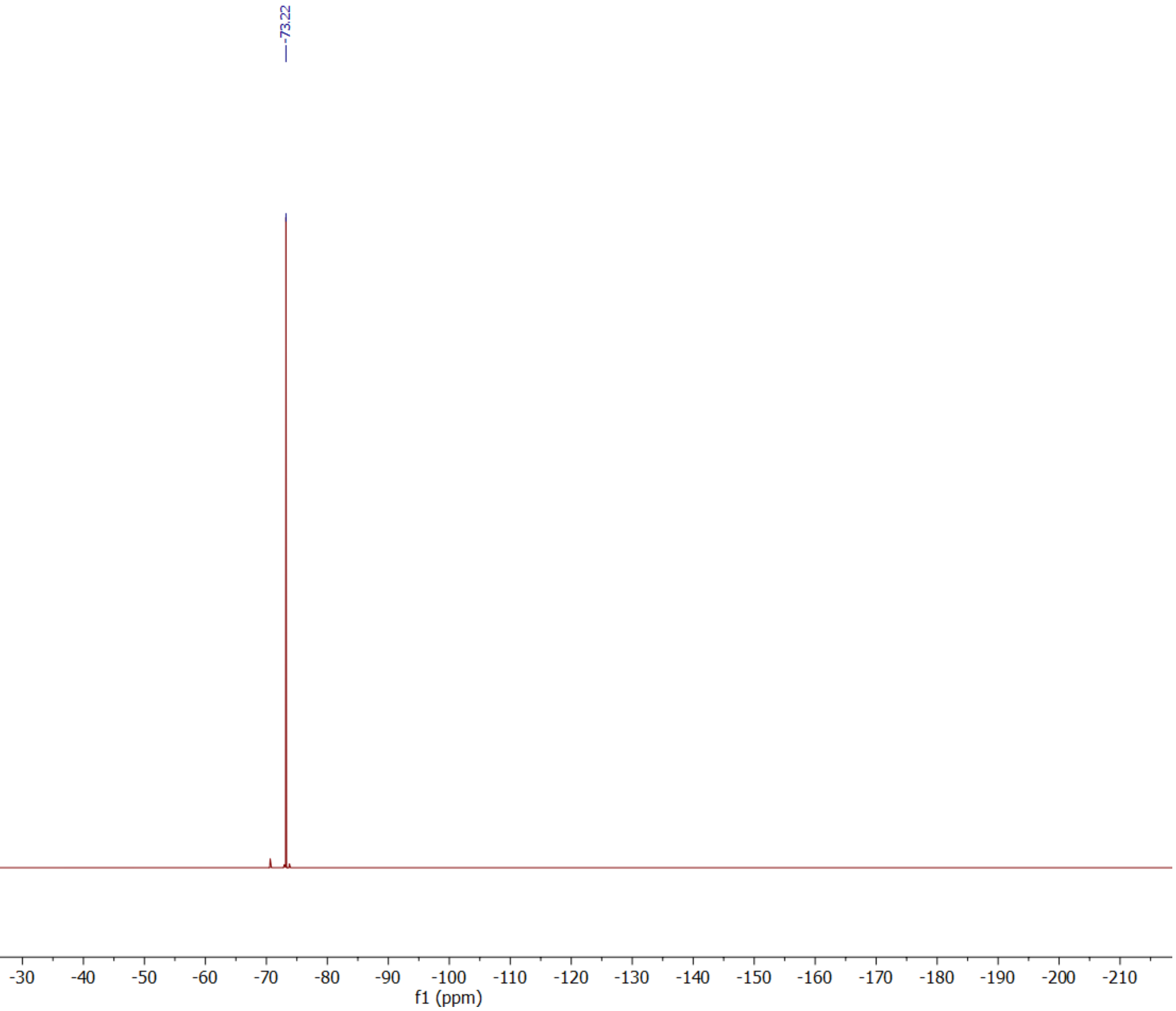

1H (400.15 MHz, CDCl3)

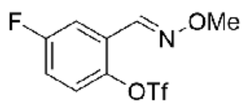

10k

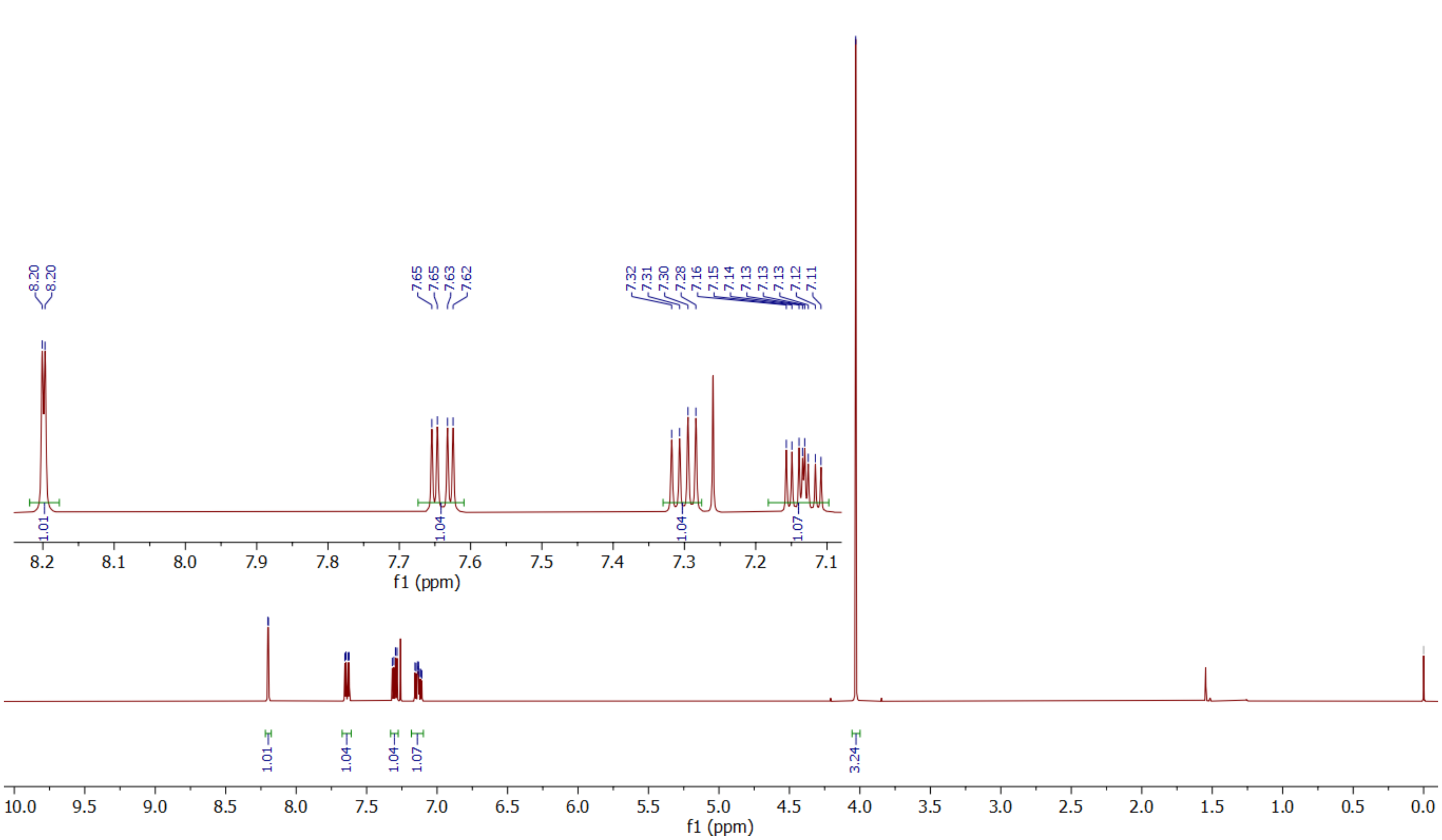

19F (376.48 MHz, CDCl3)

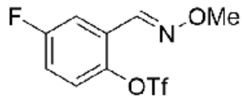

10k

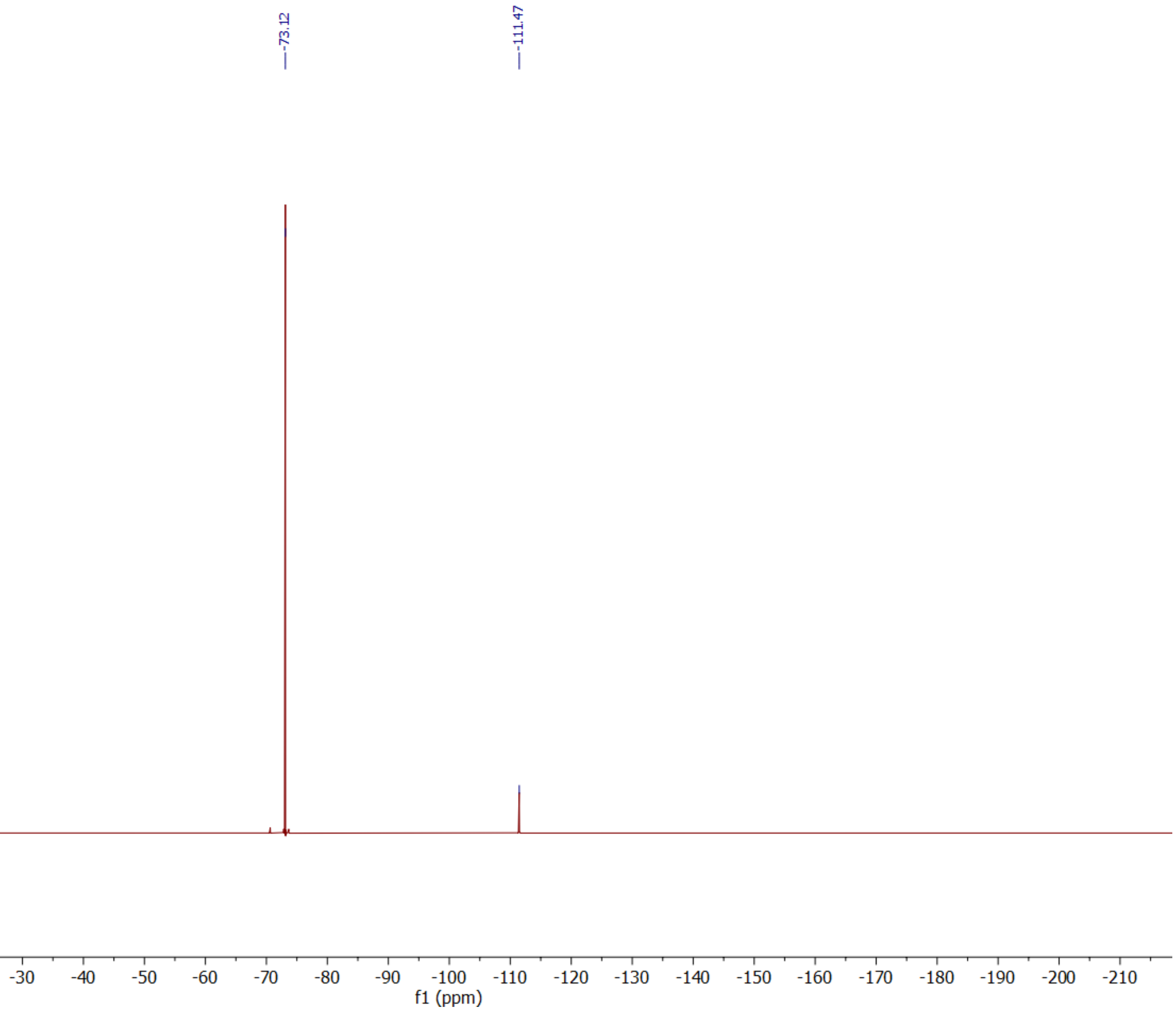

13C (100.63 MHz, CDCl3)

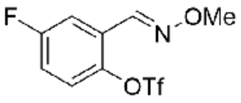

10k

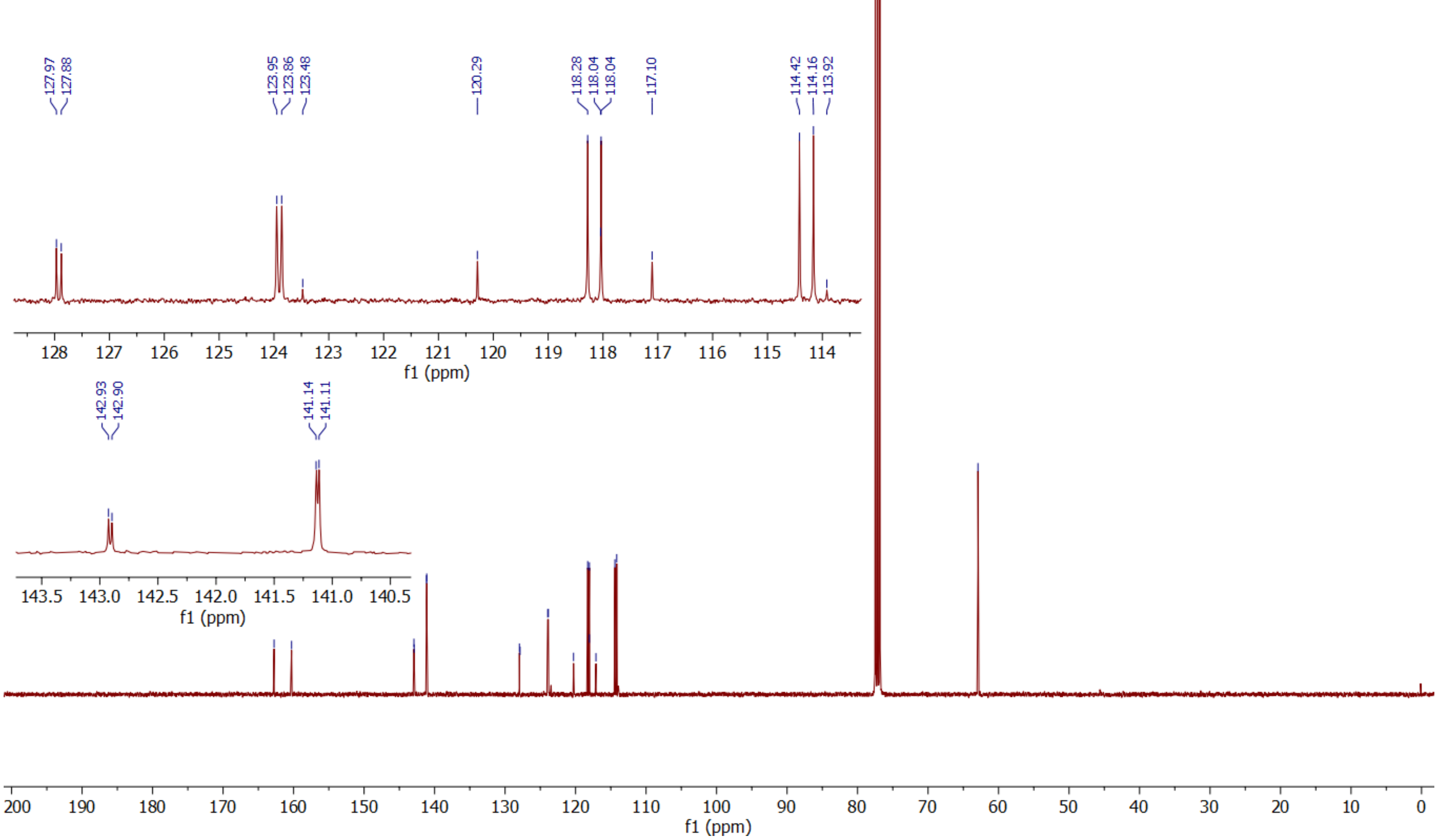

1H (400.15 MHz, CDCl3)

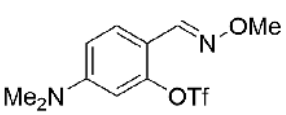

12a

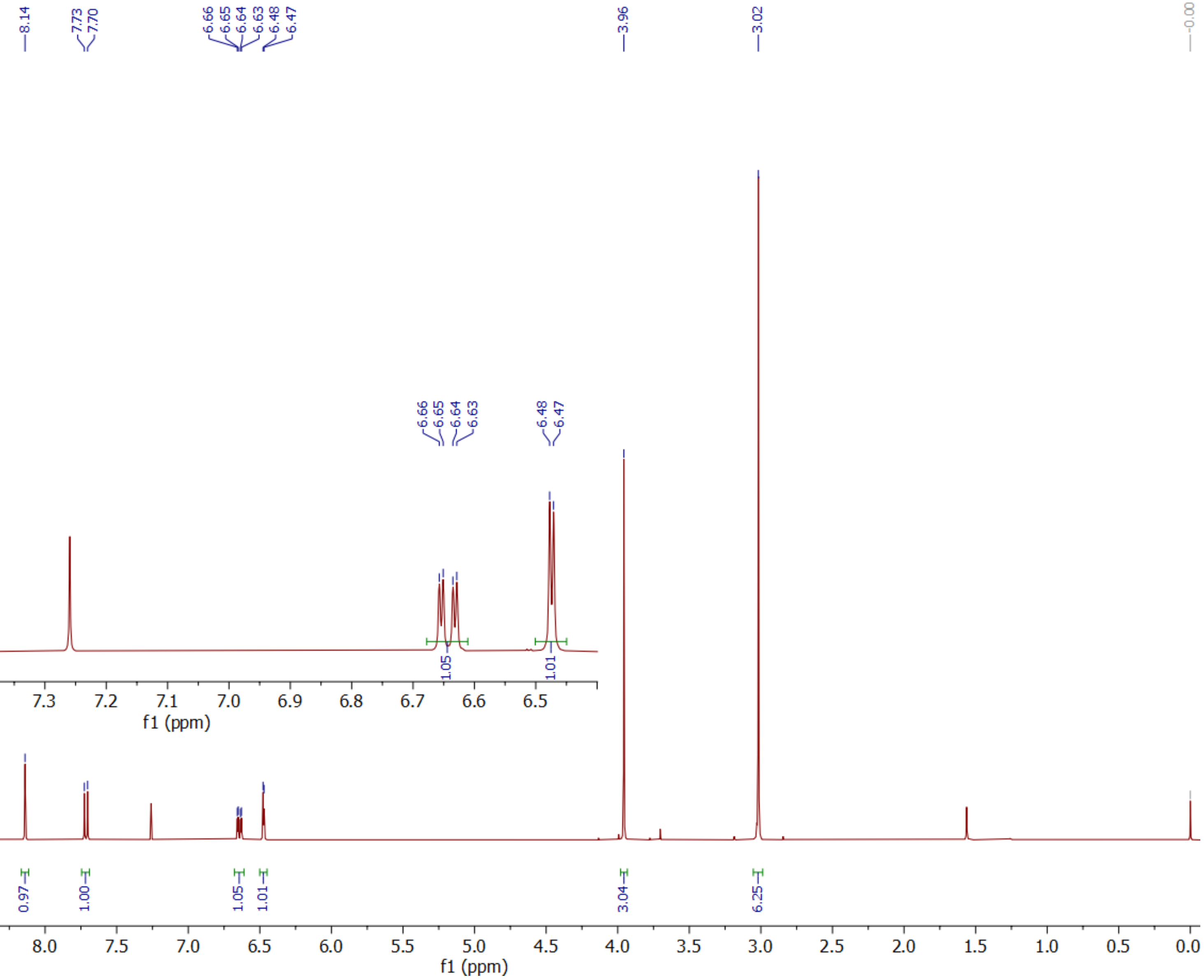

13C (100.63 MHz, CDCl3)

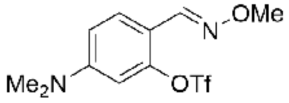

12a

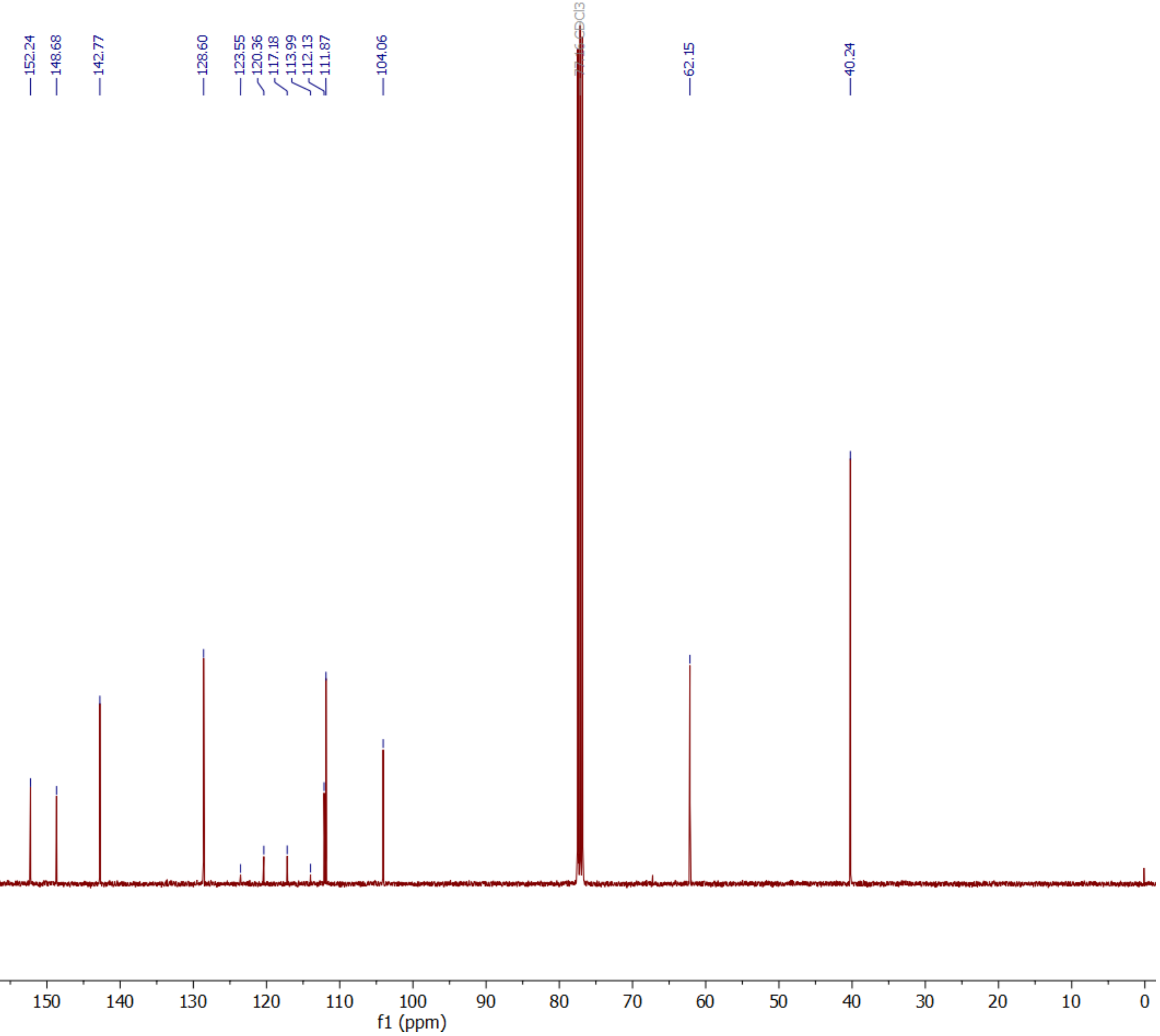

19F (376.48 MHz, CDCl3)

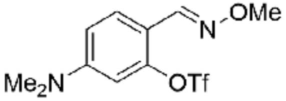

12a

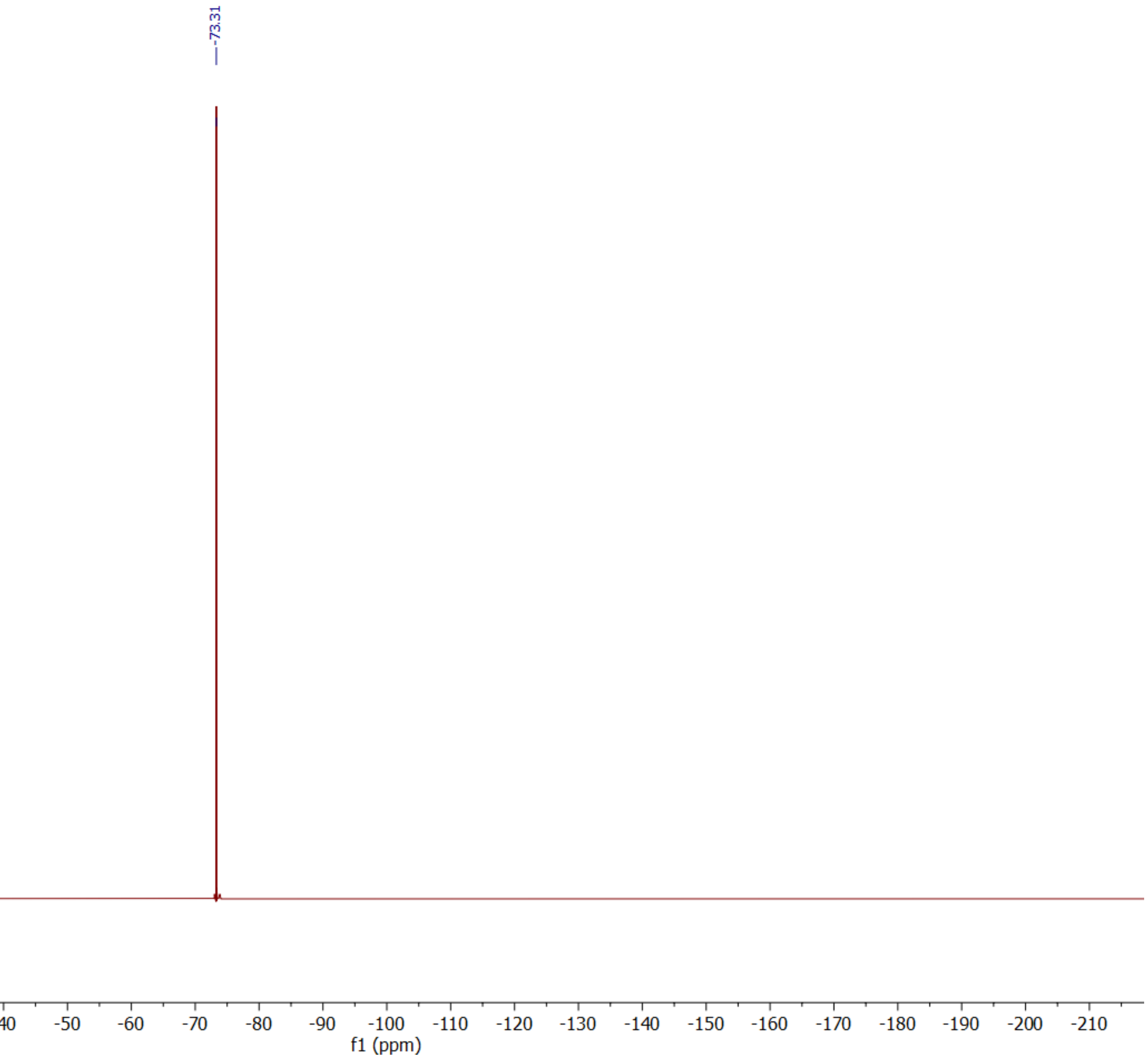

1H (400.15 MHz, CDCl3)

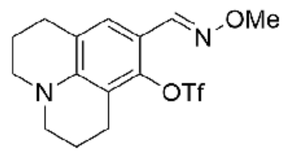

12b

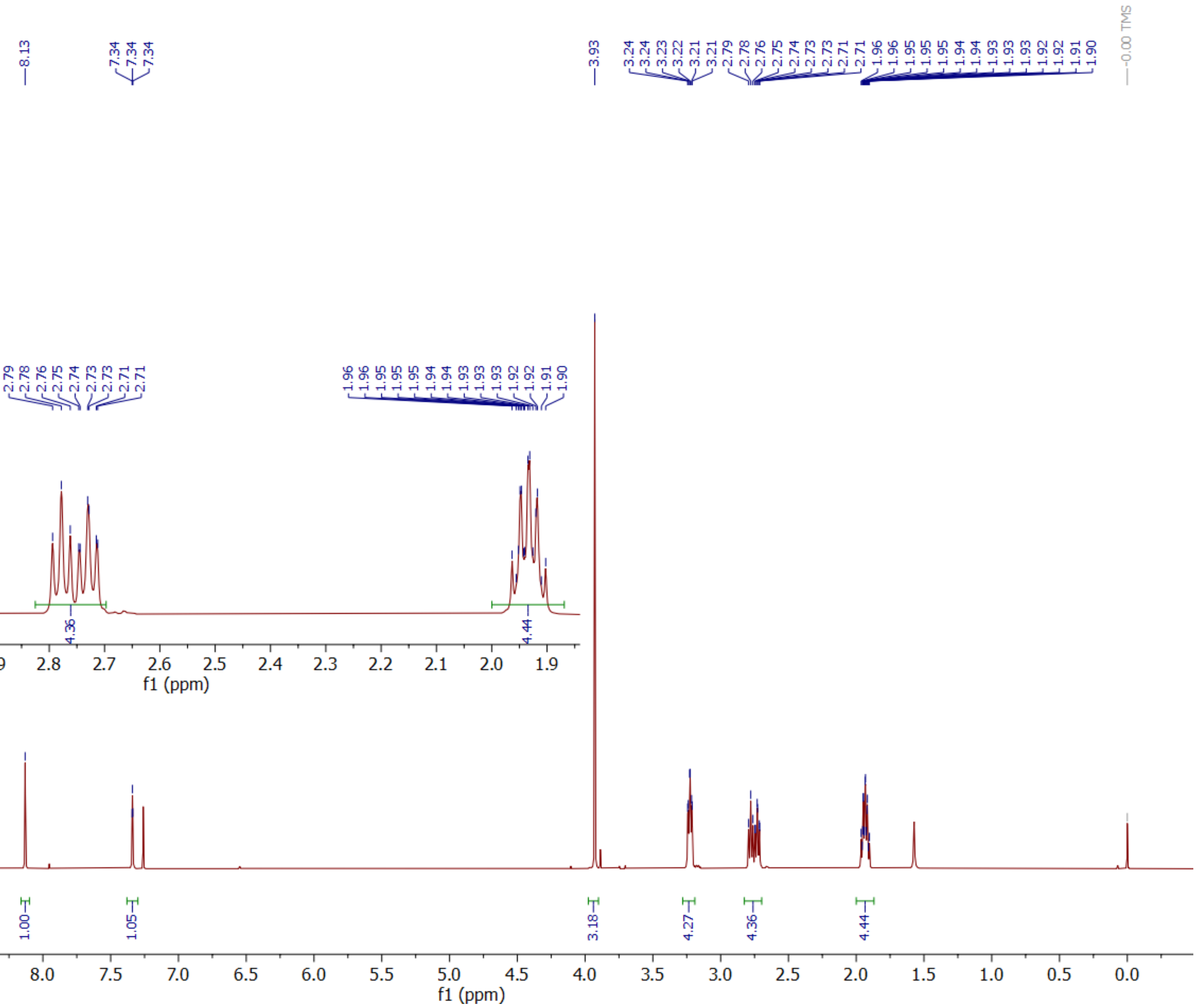

13C (100.63 MHz, CDCl3)

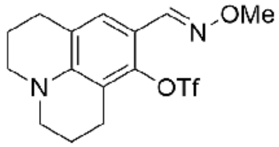

12b

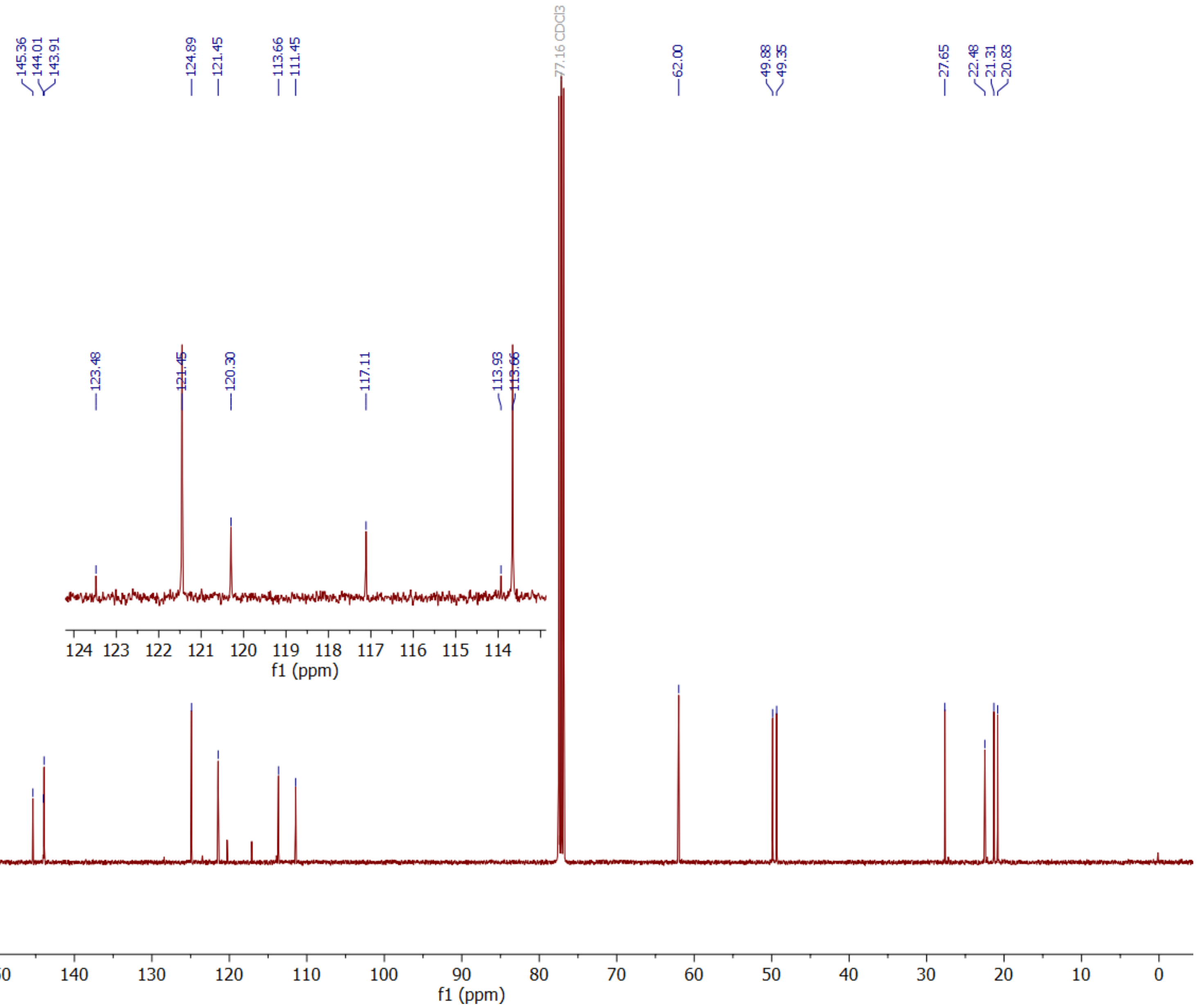

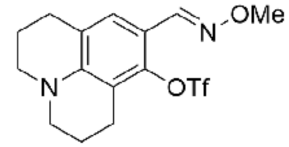
OMe
N
N
OTf

12b

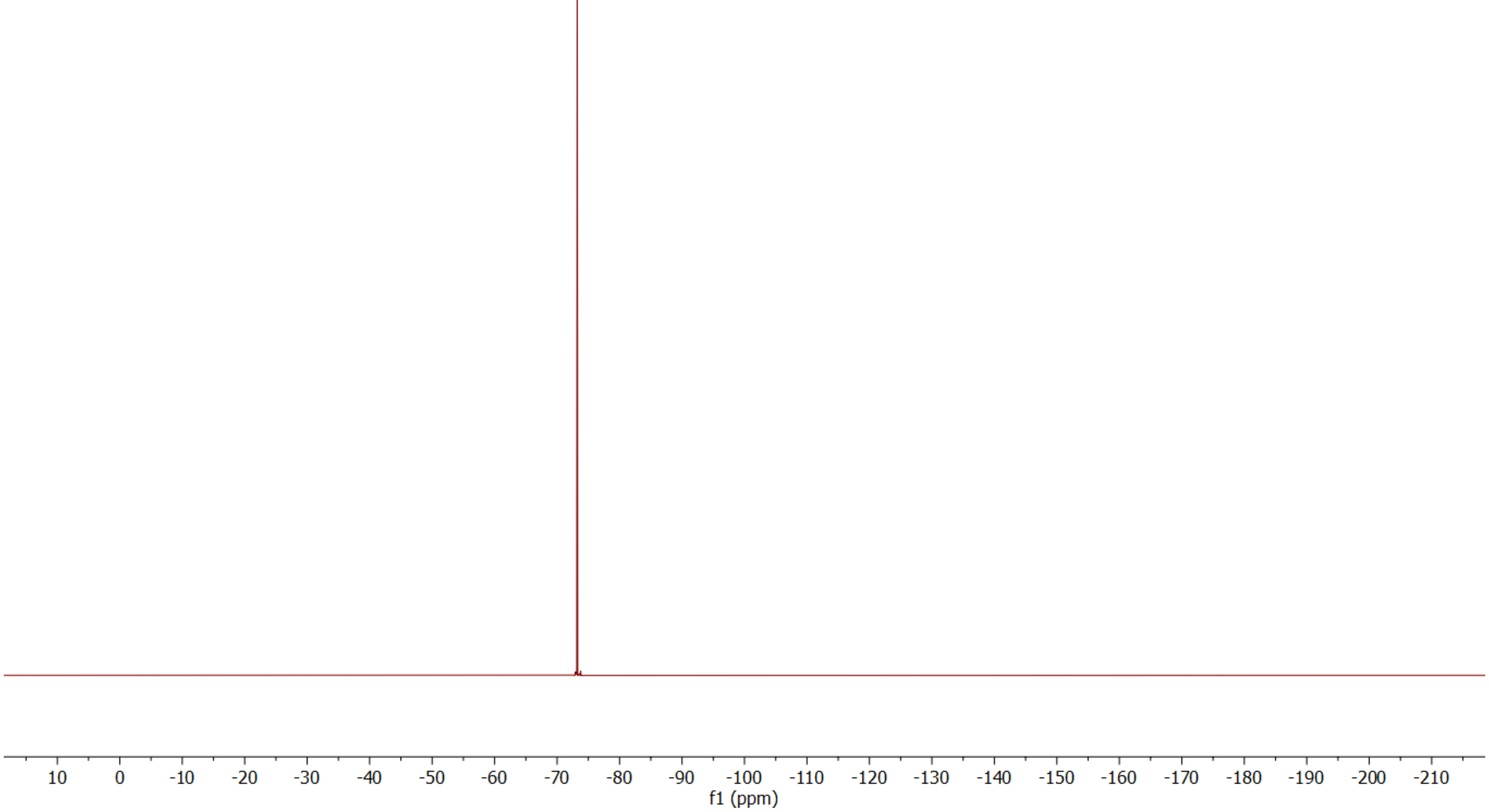
19F (376.48 MHz, CDCl3)
-73.23
10 0 -10 -20 -30 -40 -50 -60 -70 -80 -90 -100 -110 -120 -130 -140 -150 -160 -170 -180 -190 -200 -210
f1 (ppm)

1H (400.15 MHz, CDCl3)

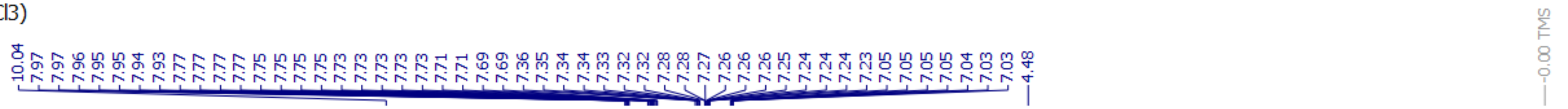

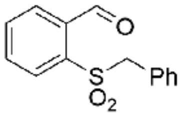

5a

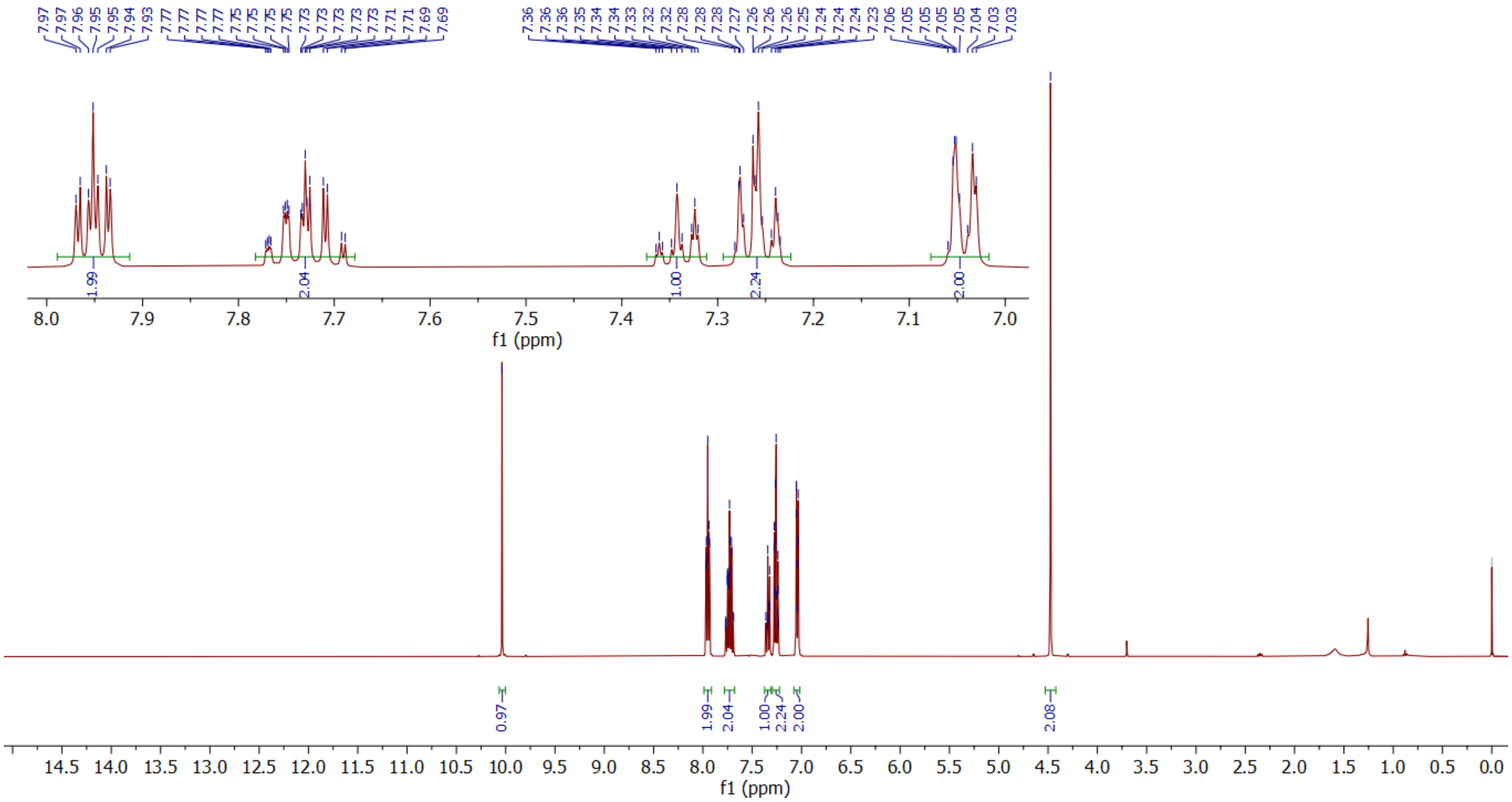

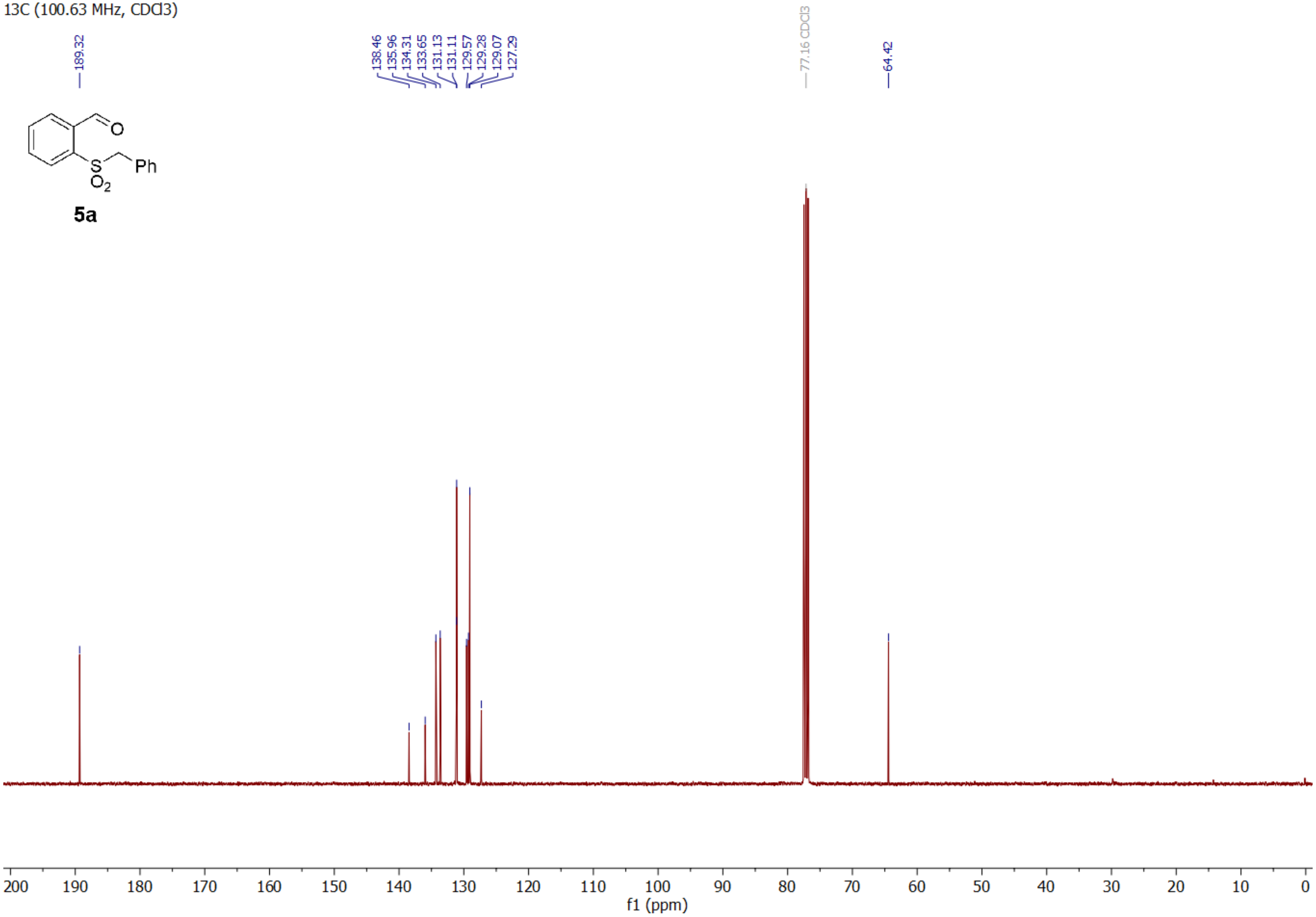
13C (100.63 MHz, CDCl3)
189.32
138.46
135.96
134.31
133.65
131.13
131.11
129.57
129.28
129.07
127.29
77.16 CDCl3
64.42
O
S
O2
Ph
5a
200 190 180 170 160 150 140 130 120 110 100 90 80 70 60 50 40 30 20 10 0
f1 (ppm)

1H (400.15 MHz, CDCl3)

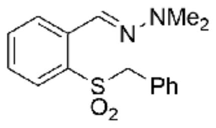

8

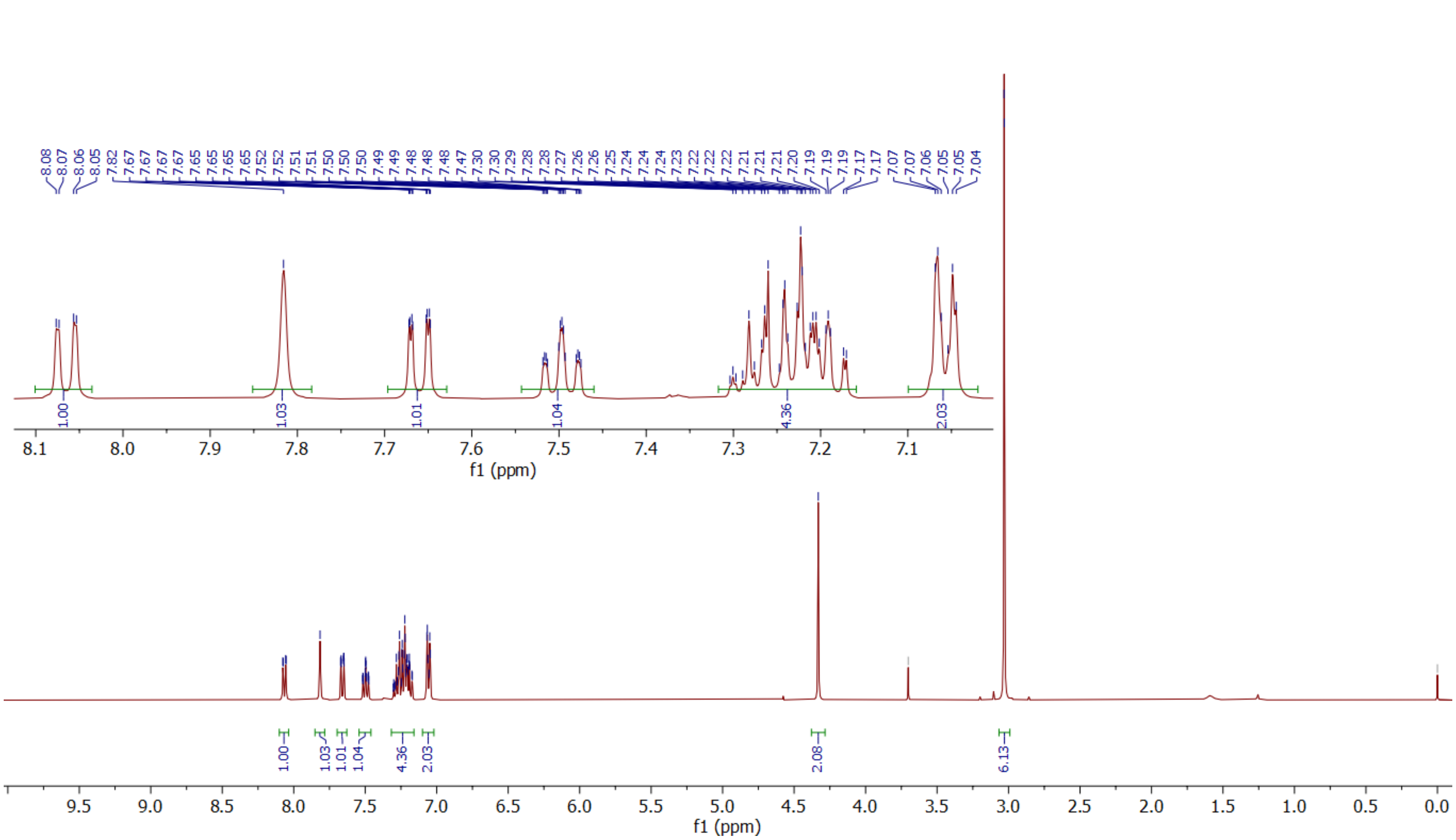

13C (100.63 MHz, CDCl3)

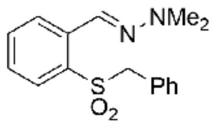

8

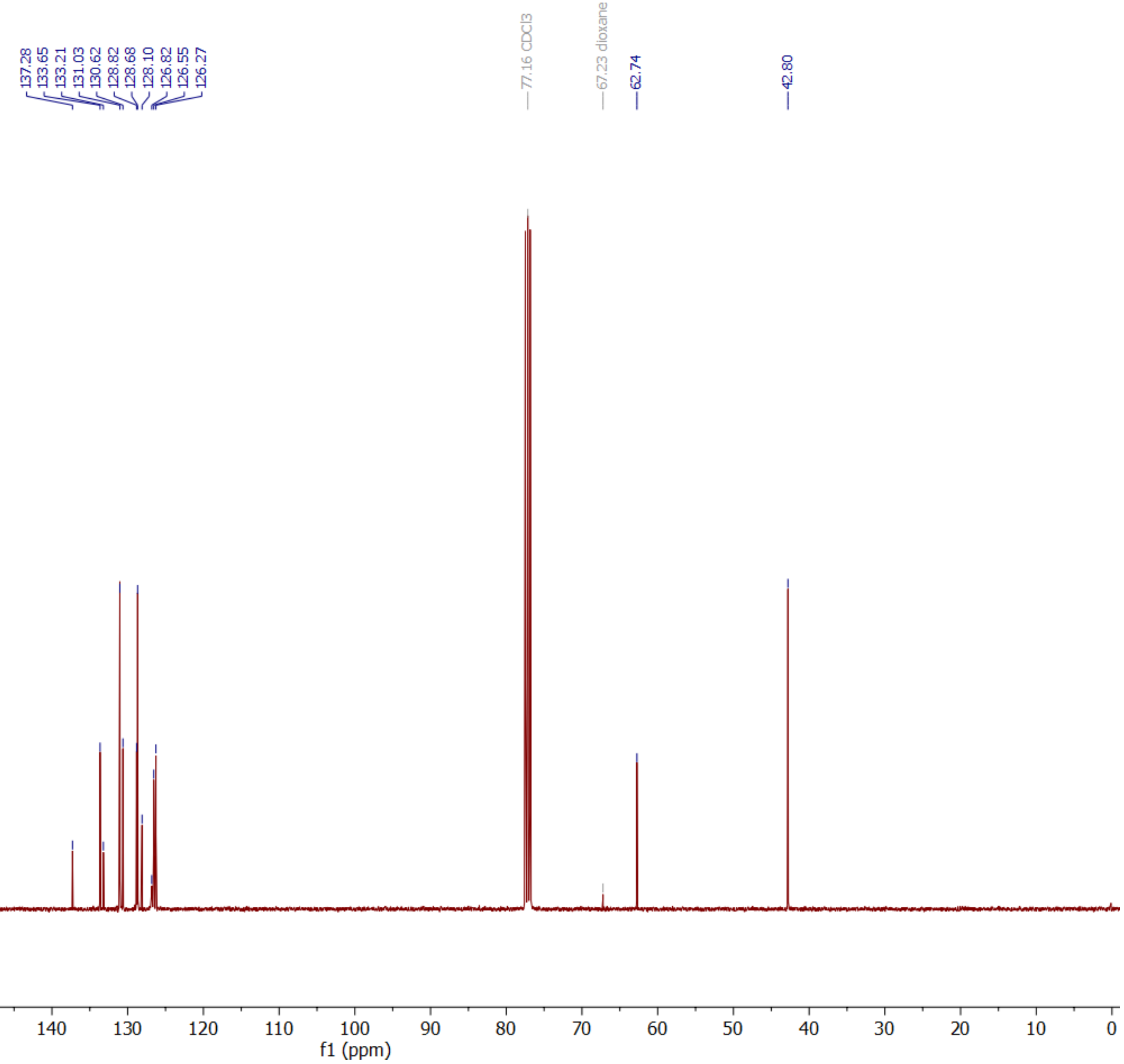

1H (400.15 MHz, CDCl3)

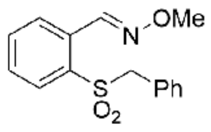

11a

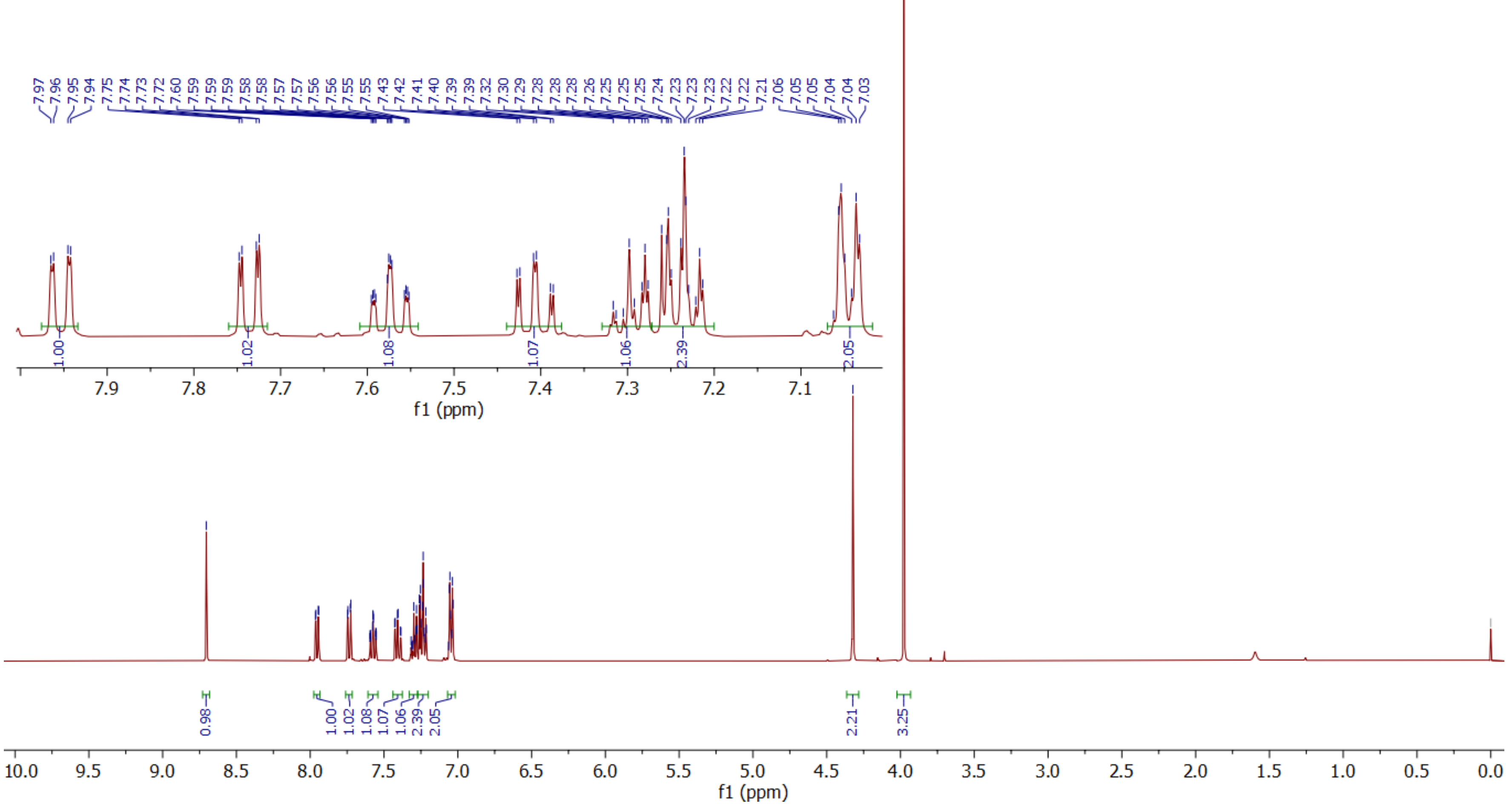

13C (100.63 MHz, CDCl3)

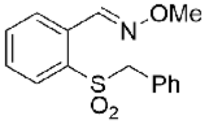

11a

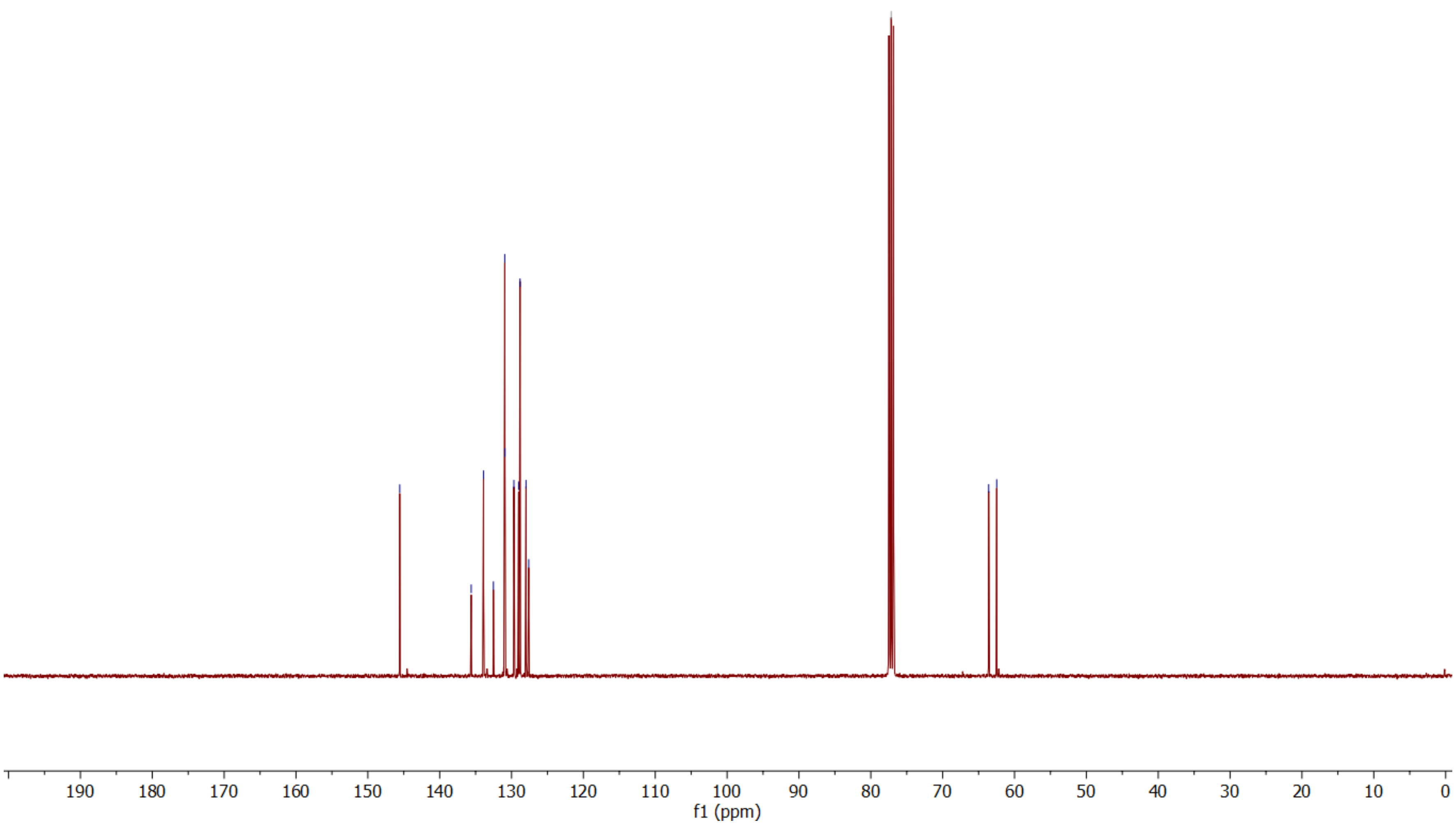

1H (400.15 MHz, CDCl3)

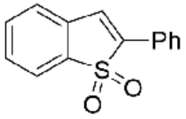

6a

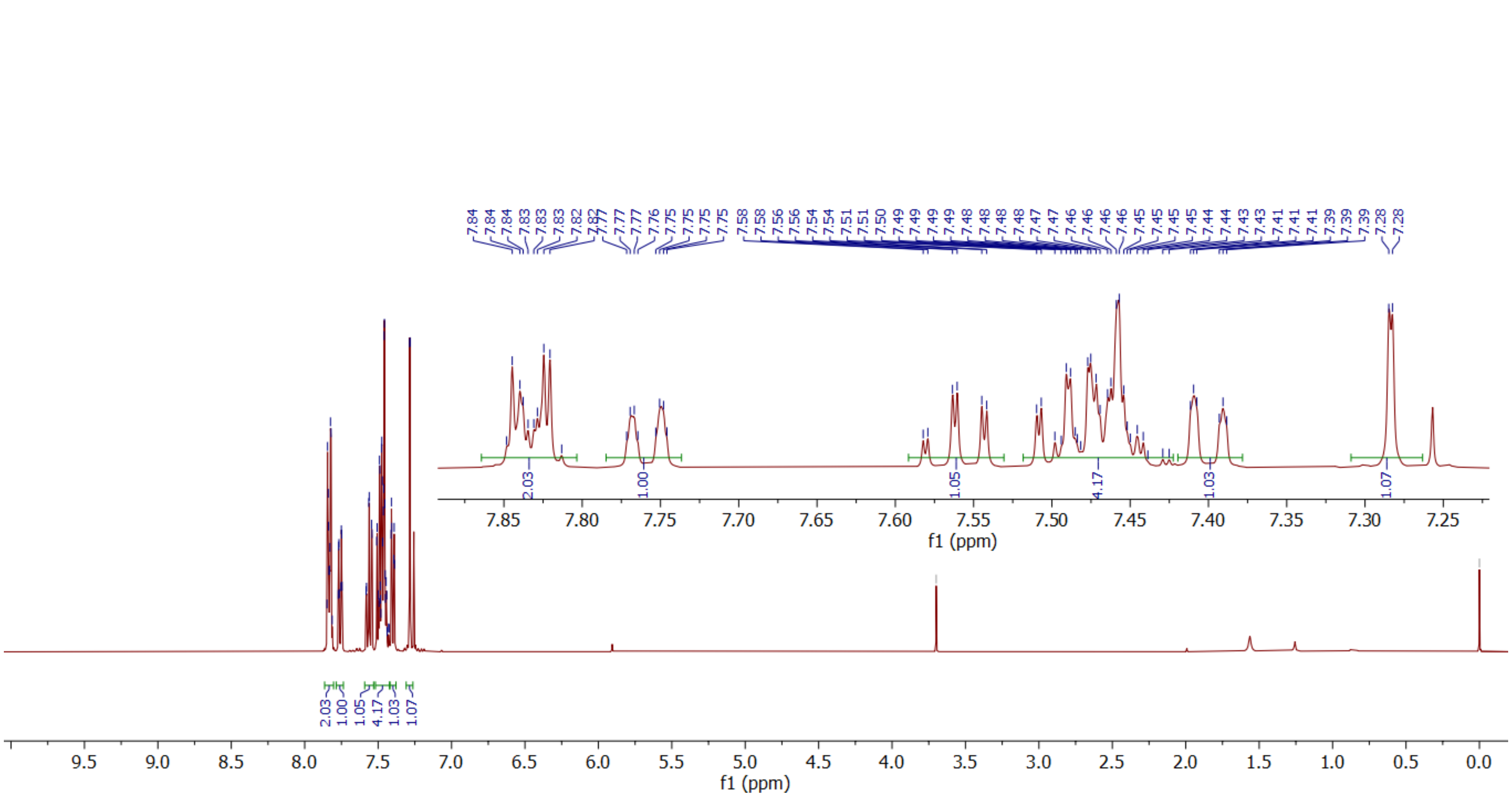

13C (100.63 MHz, CDCl3)

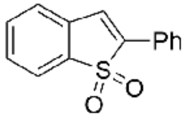

6a

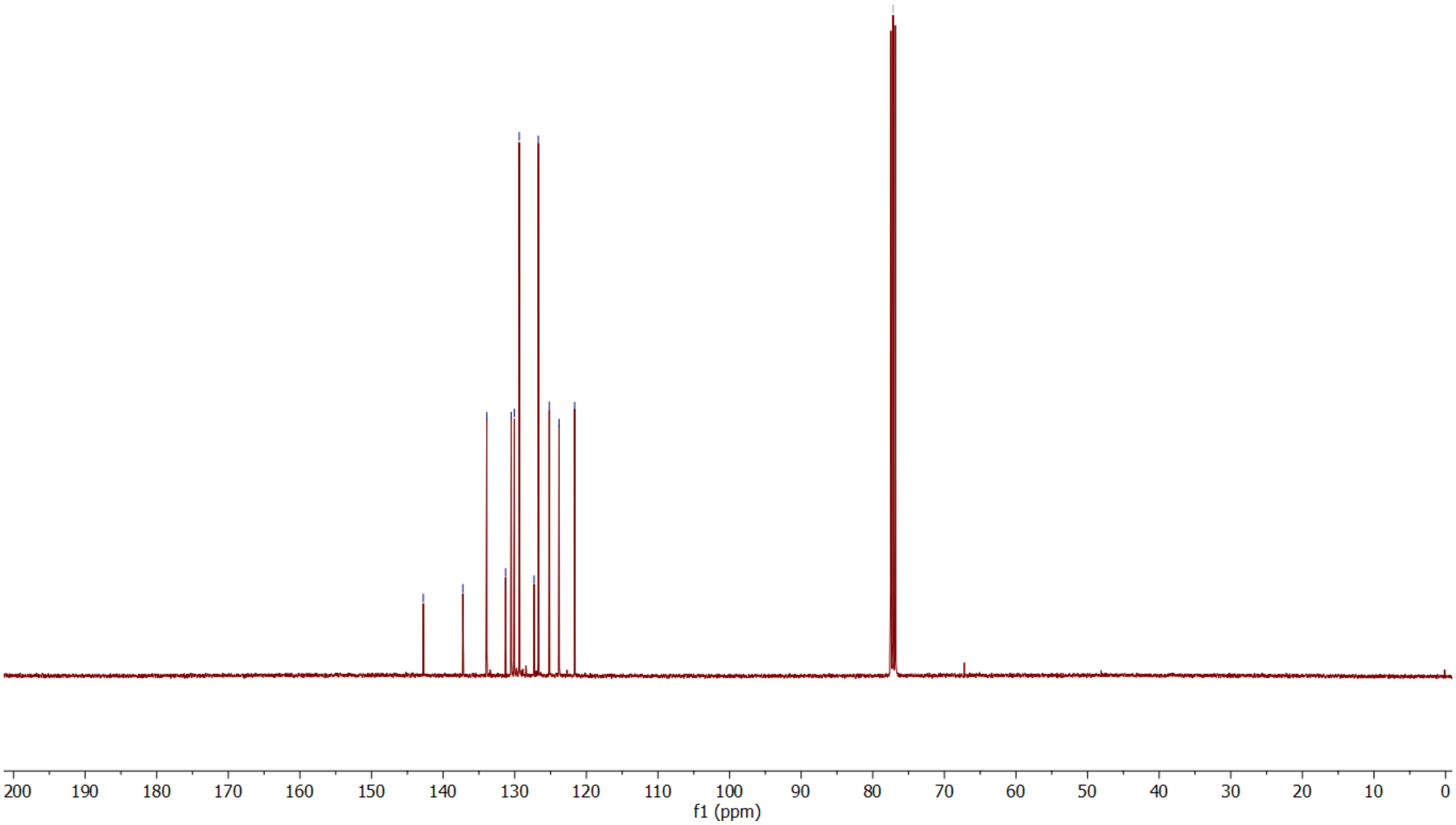

1H (400.15 MHz, CDCl3)

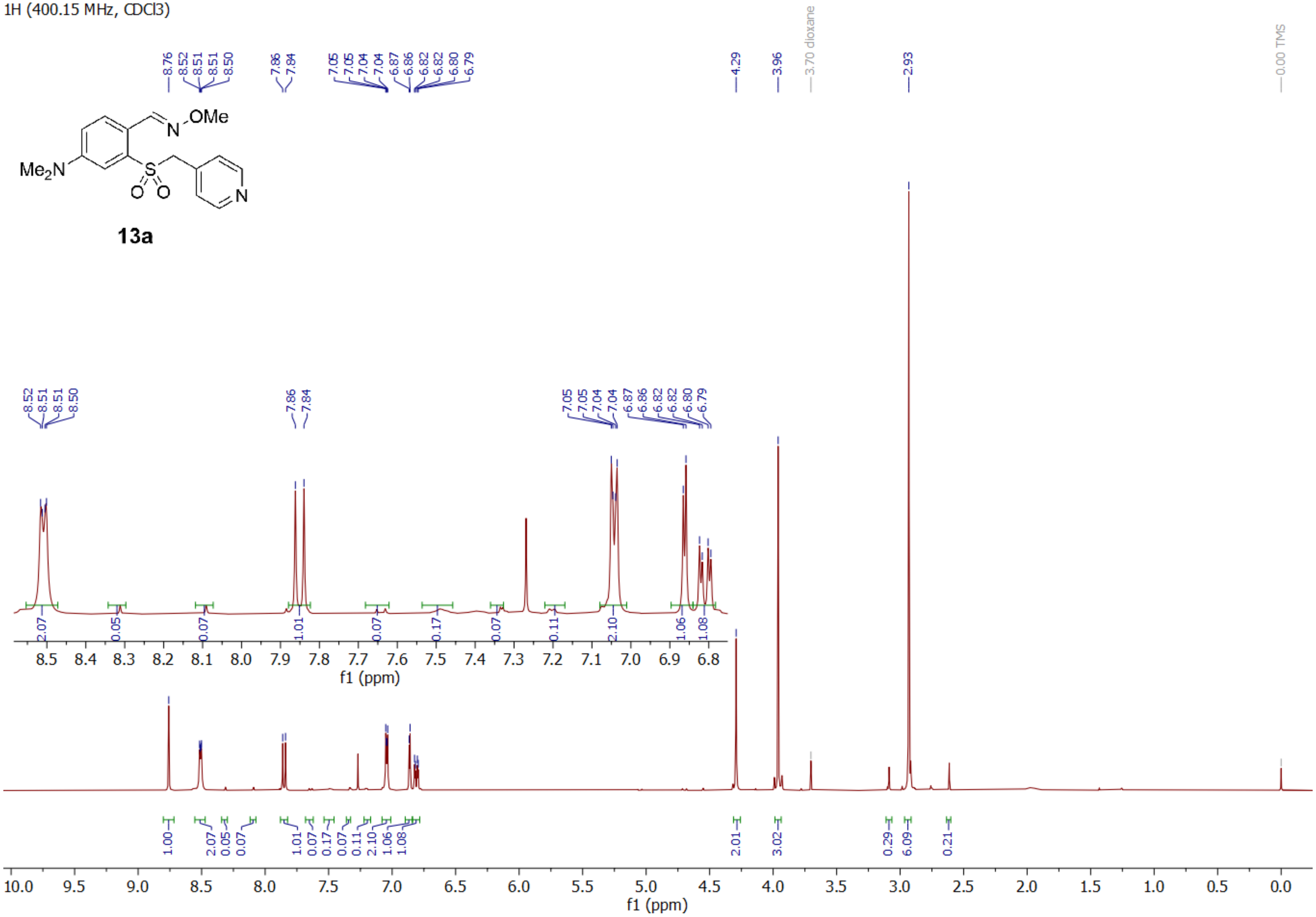

13a

13C (100.63 MHz, CDCl3)

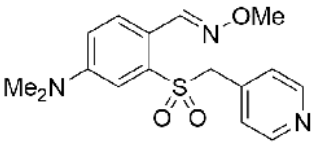

13a

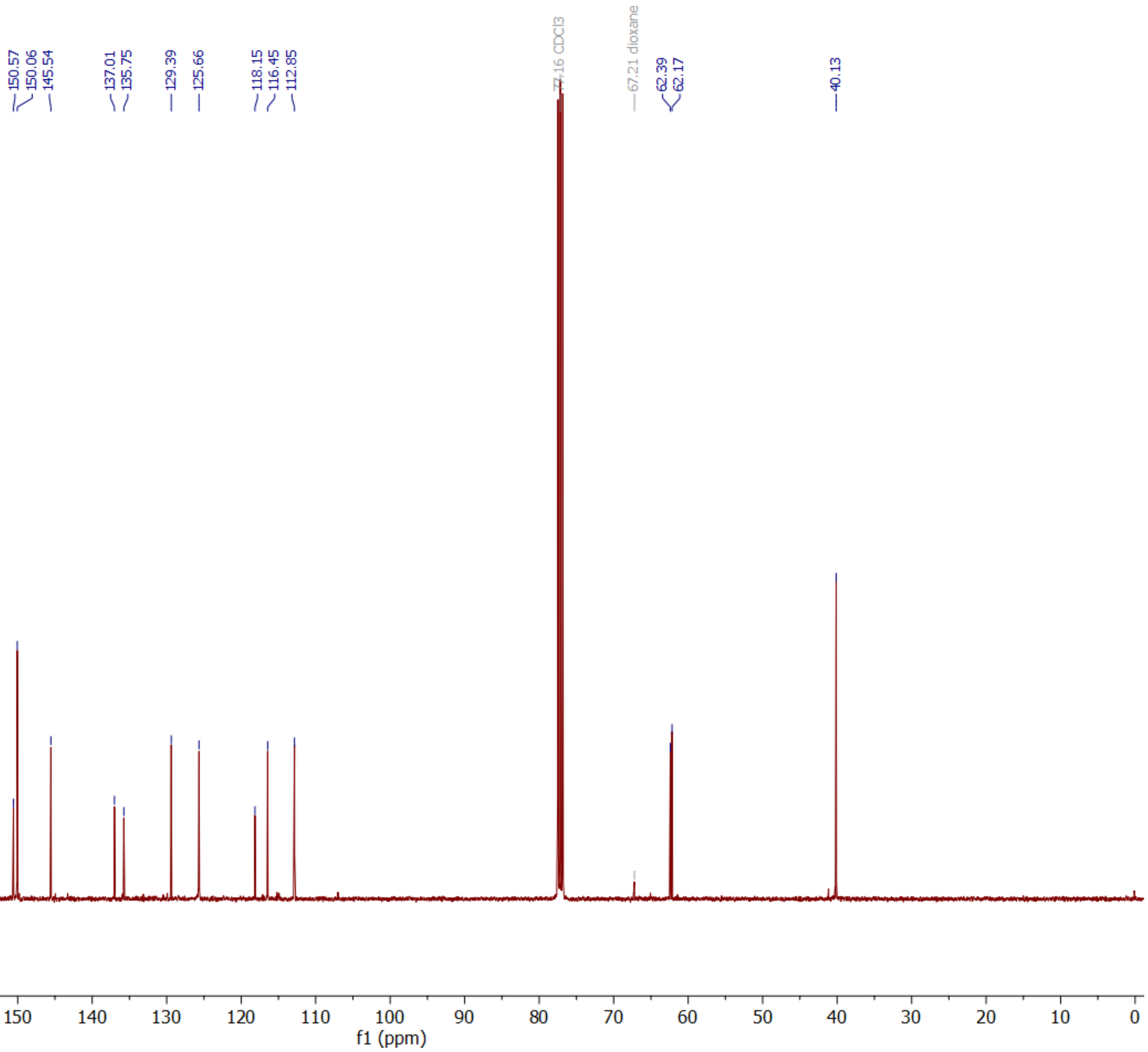

1H (400.15 MHz, DMSO)

14a

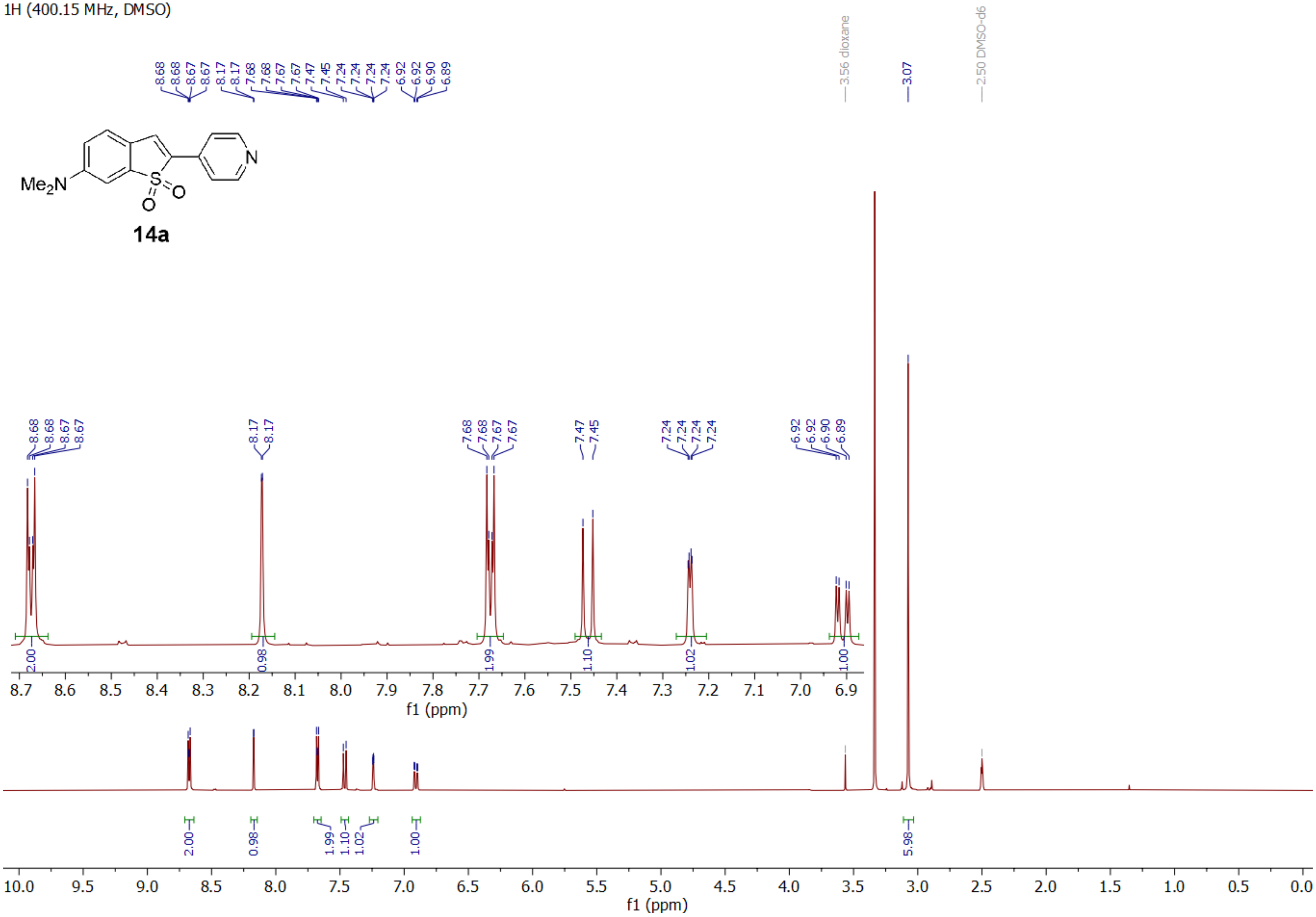

13C (100.63 MHz, DMSO)

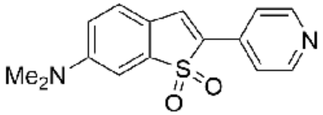

14a

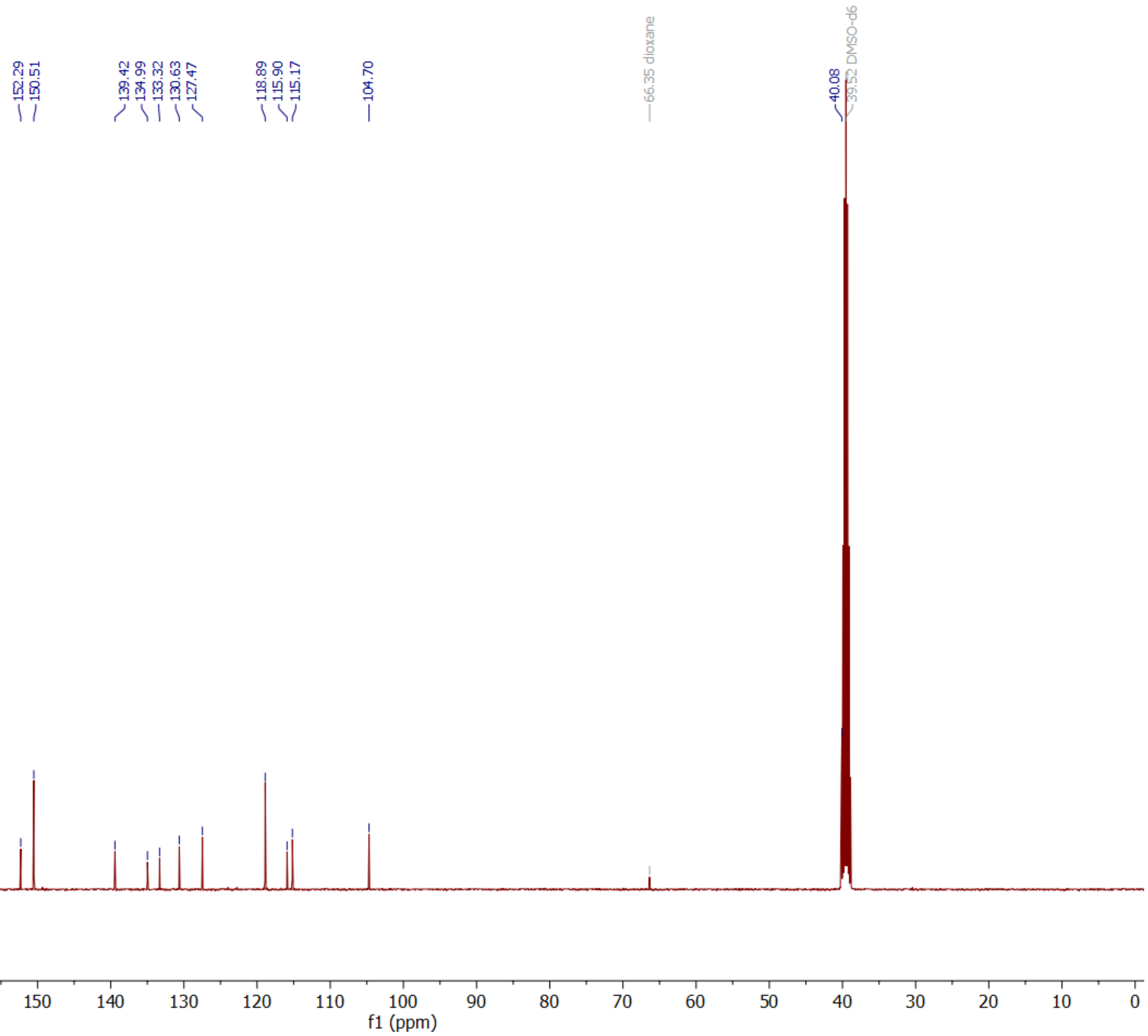

1H (400.15 MHz, DMSO)

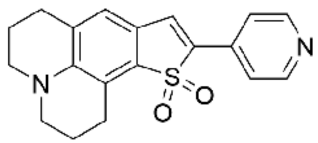

14b

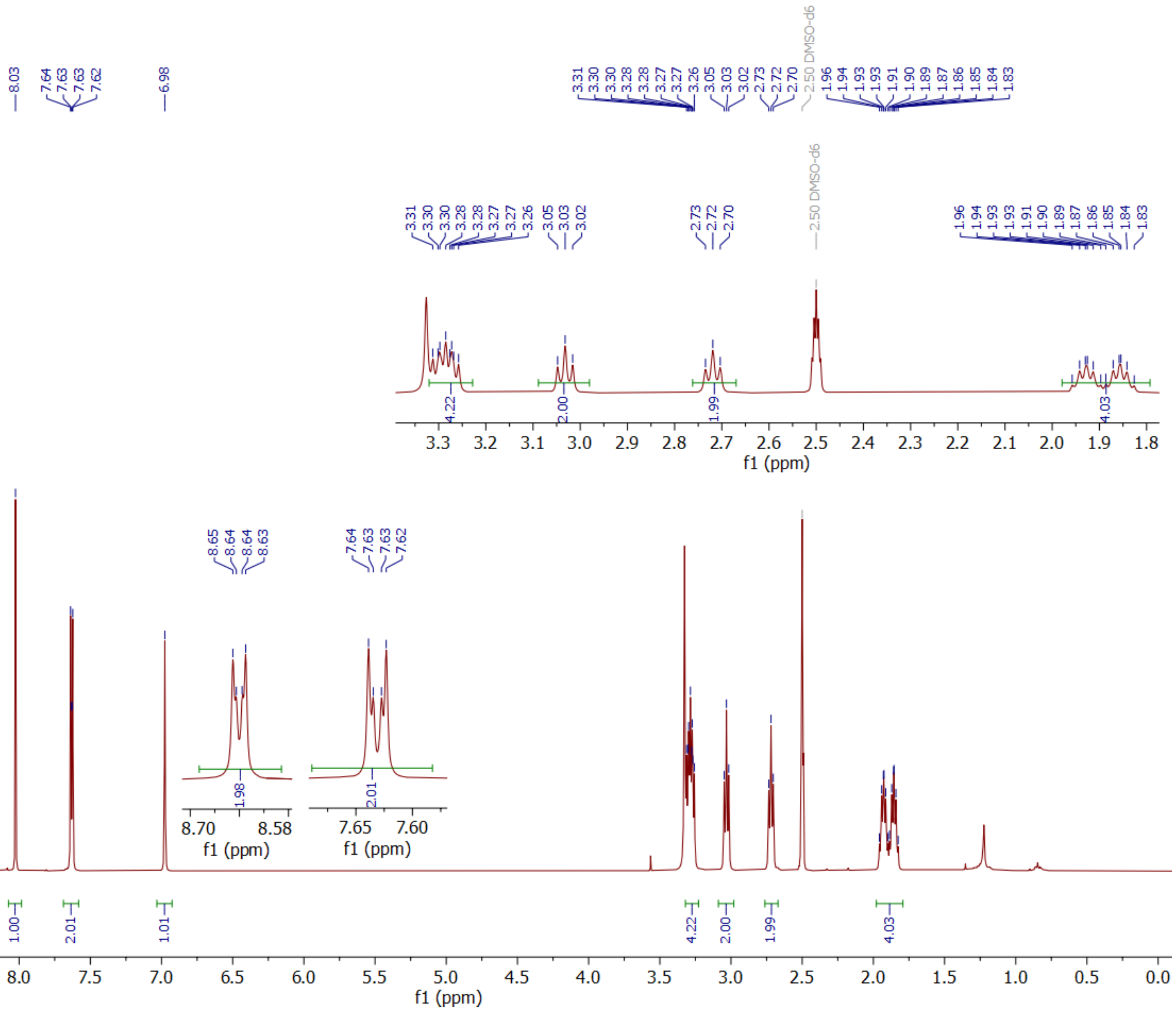

13C (100.63 MHz, DMSO)

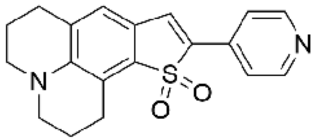

14b

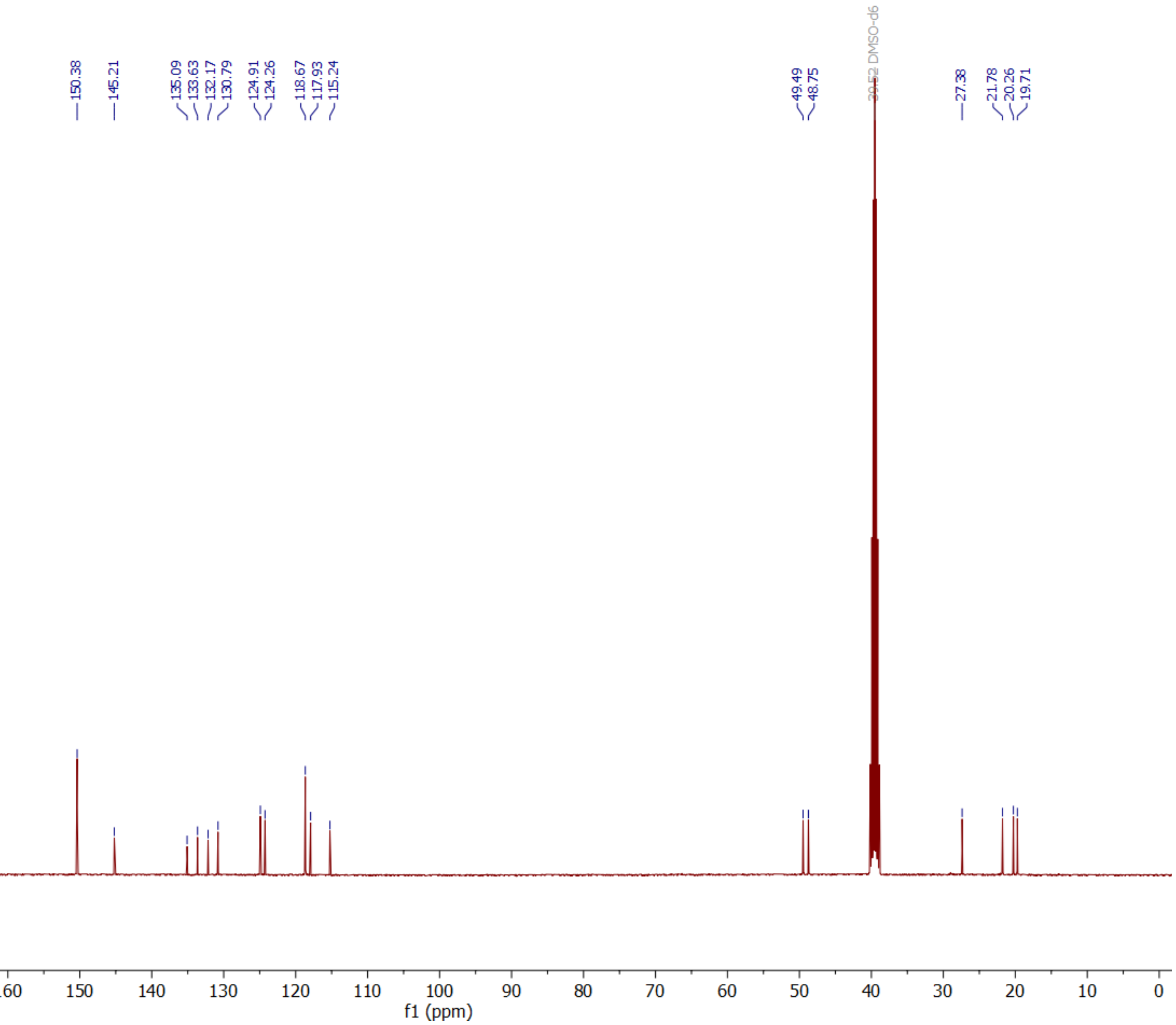

1H (400.15 MHz, DMSO)

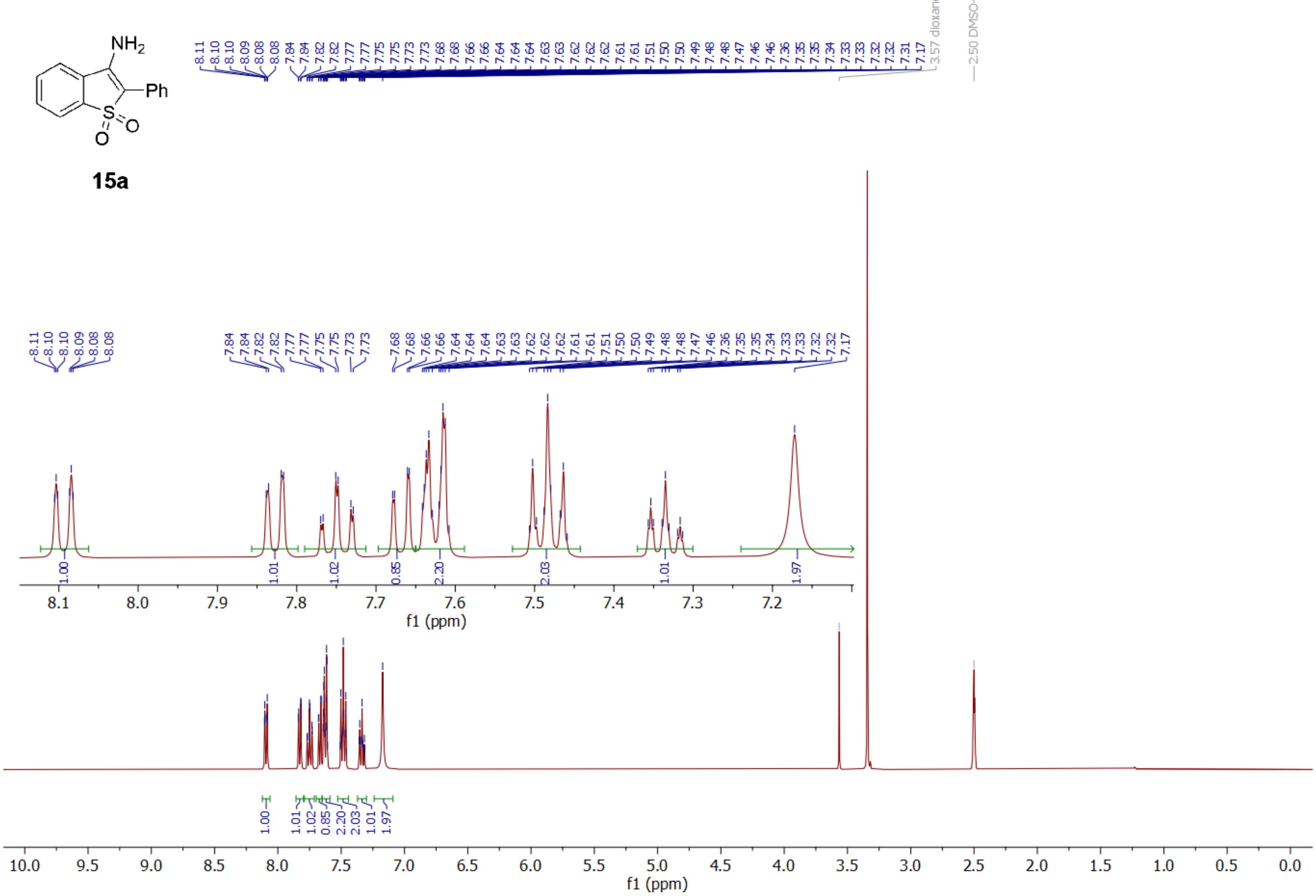

15a

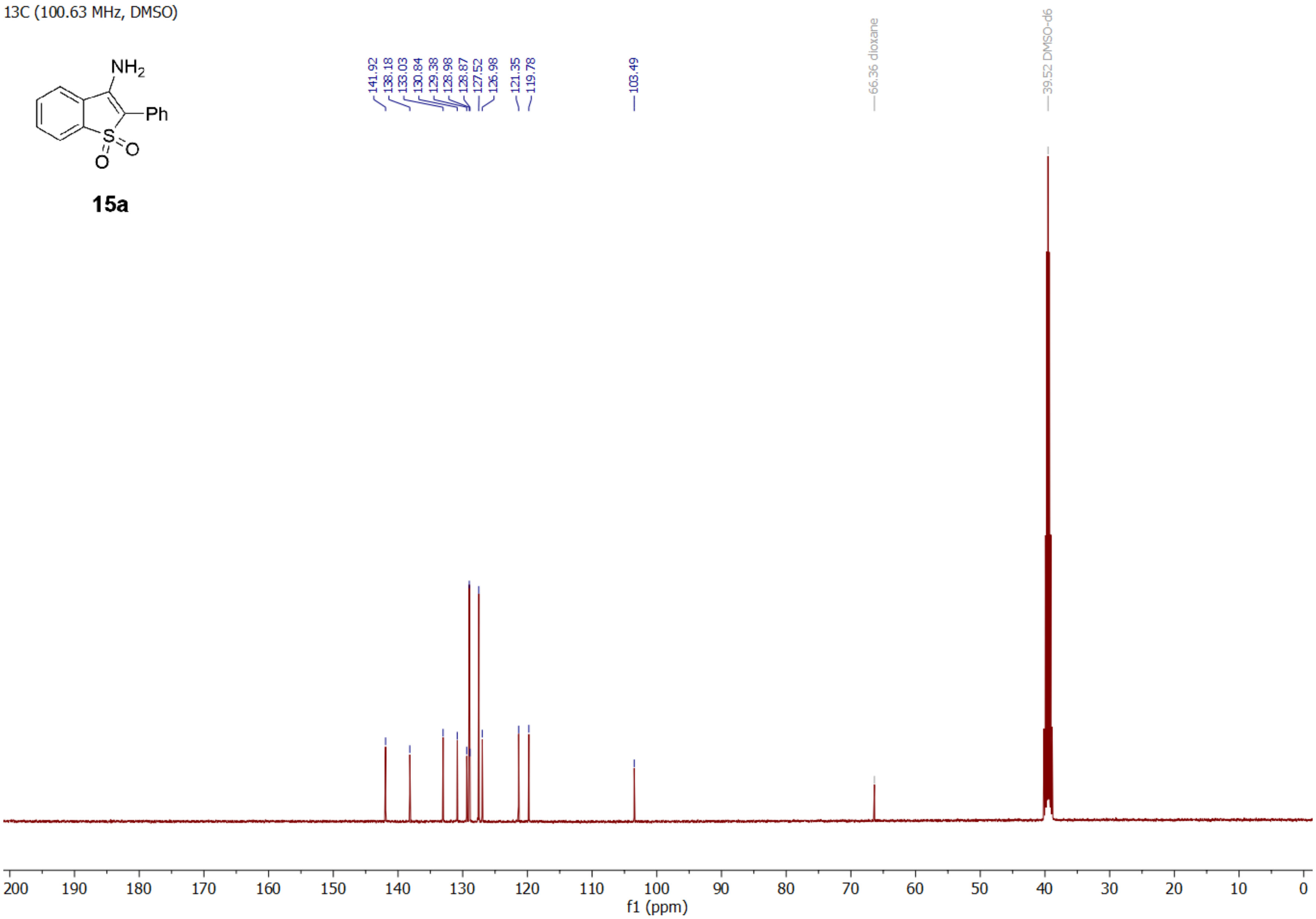
13C (100.63 MHz, DMSO)
NH2
Ph
S
O
O
15a
141.92
138.18
133.03
130.84
129.38
128.98
128.87
127.52
126.98
121.35
119.78
103.49
66.36 dioxane
39.52 DMSO-d6
200 190 180 170 160 150 140 130 120 110 100 90 80 70 60 50 40 30 20 10 0
f1 (ppm)

1H (400.15 MHz, CDCl3)

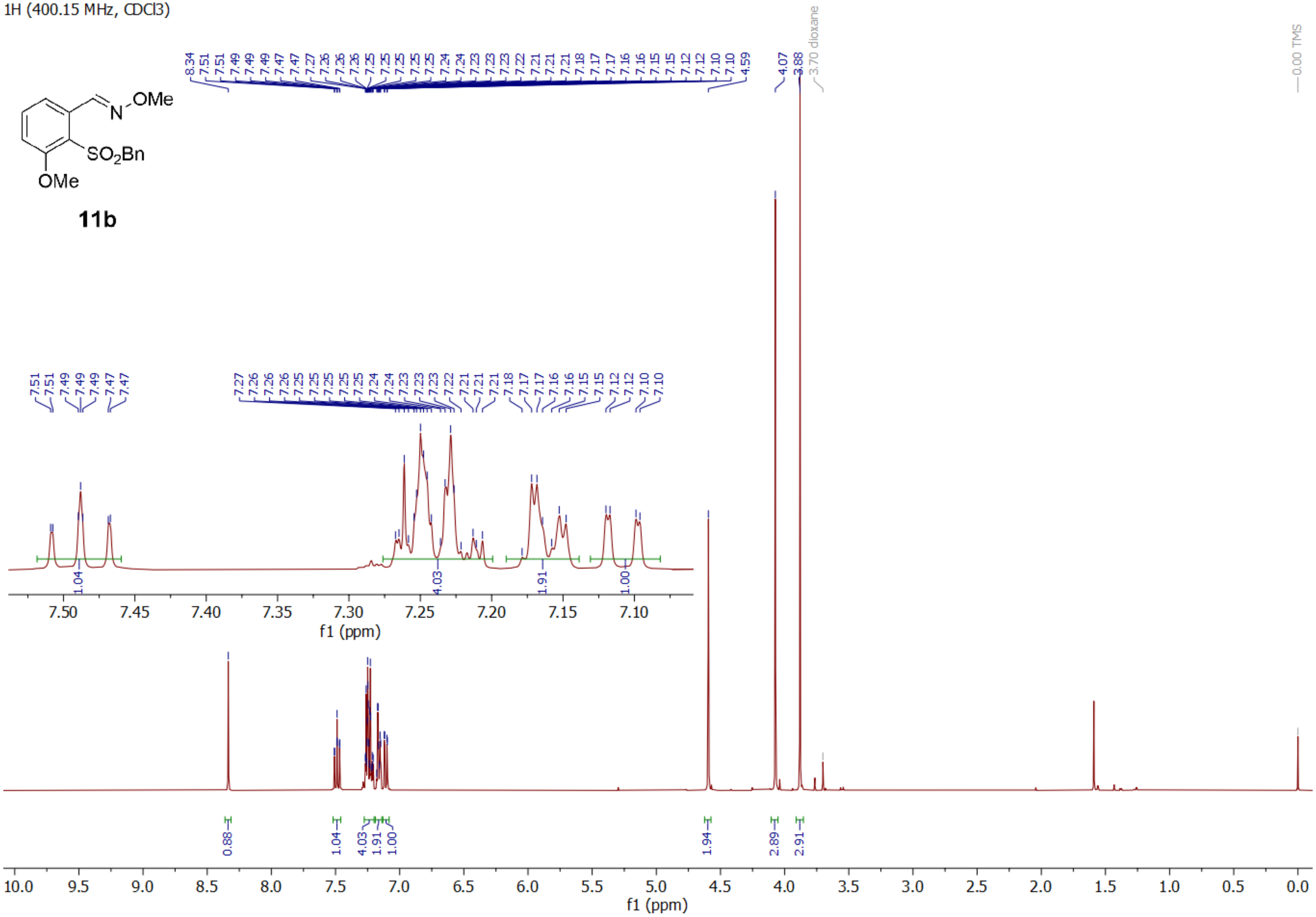

13C (100.63 MHz, CDCl3)

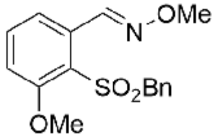

11b

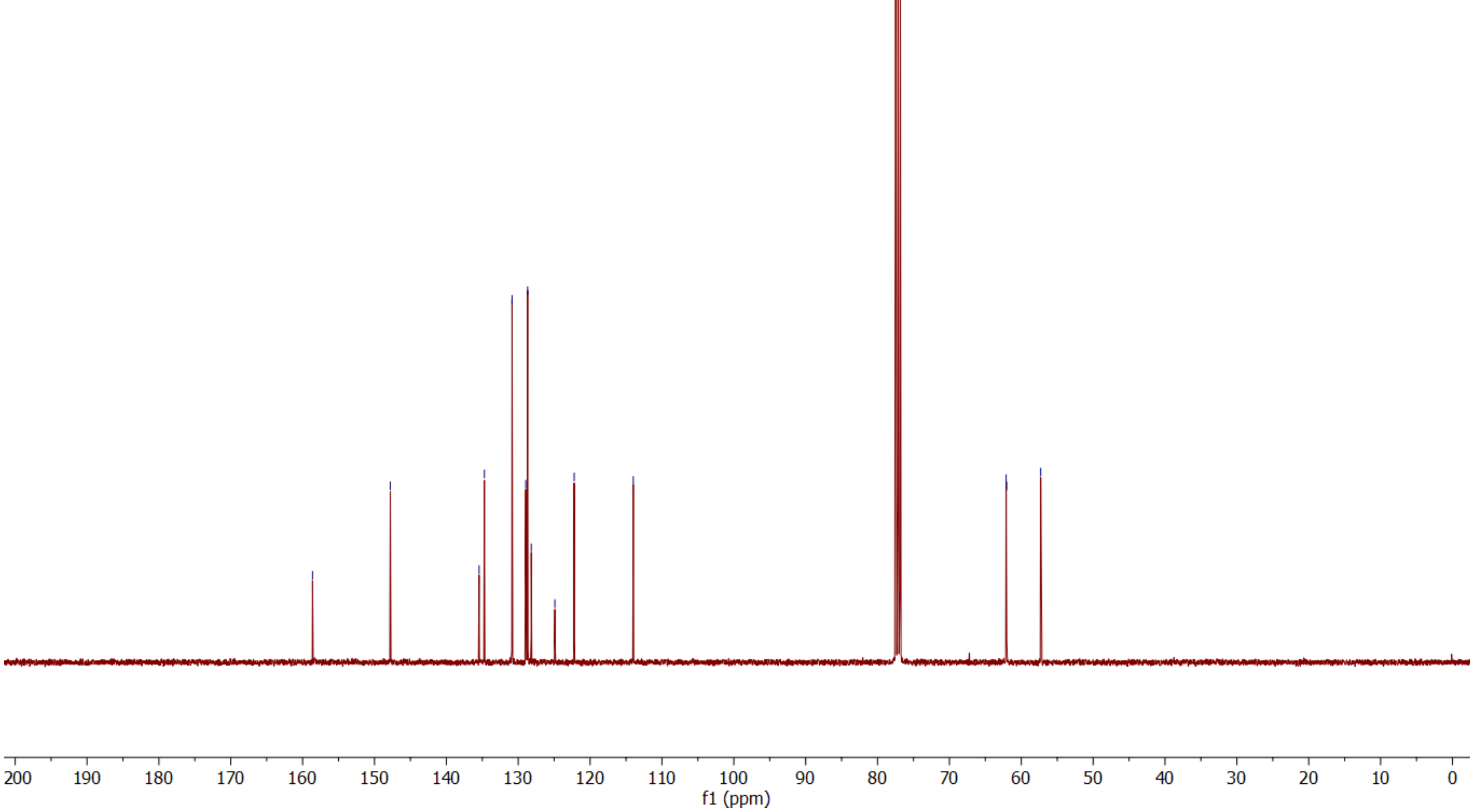

1H (400.15 MHz, DMSO)

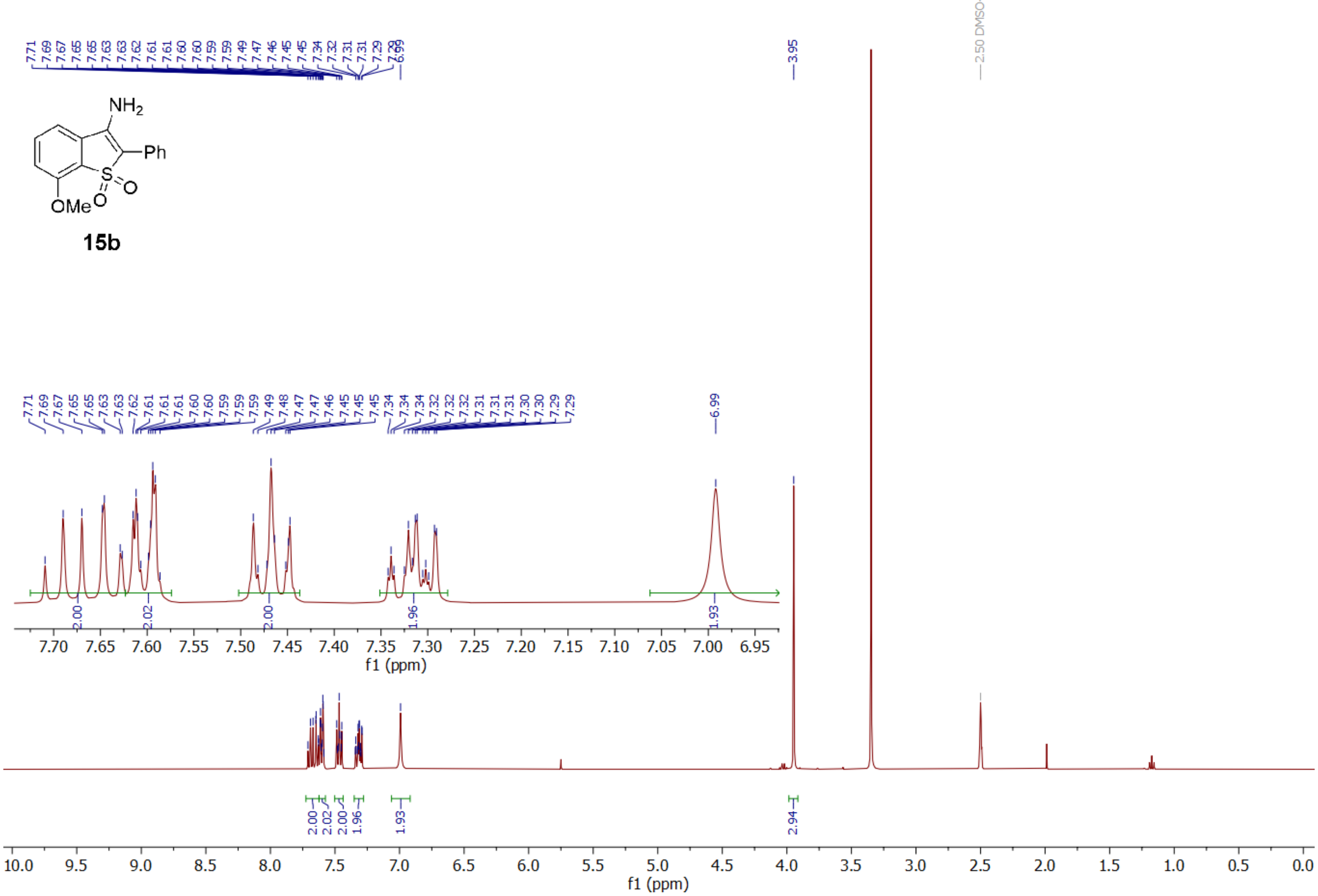

15b

13C (100.63 MHz, DMSO)

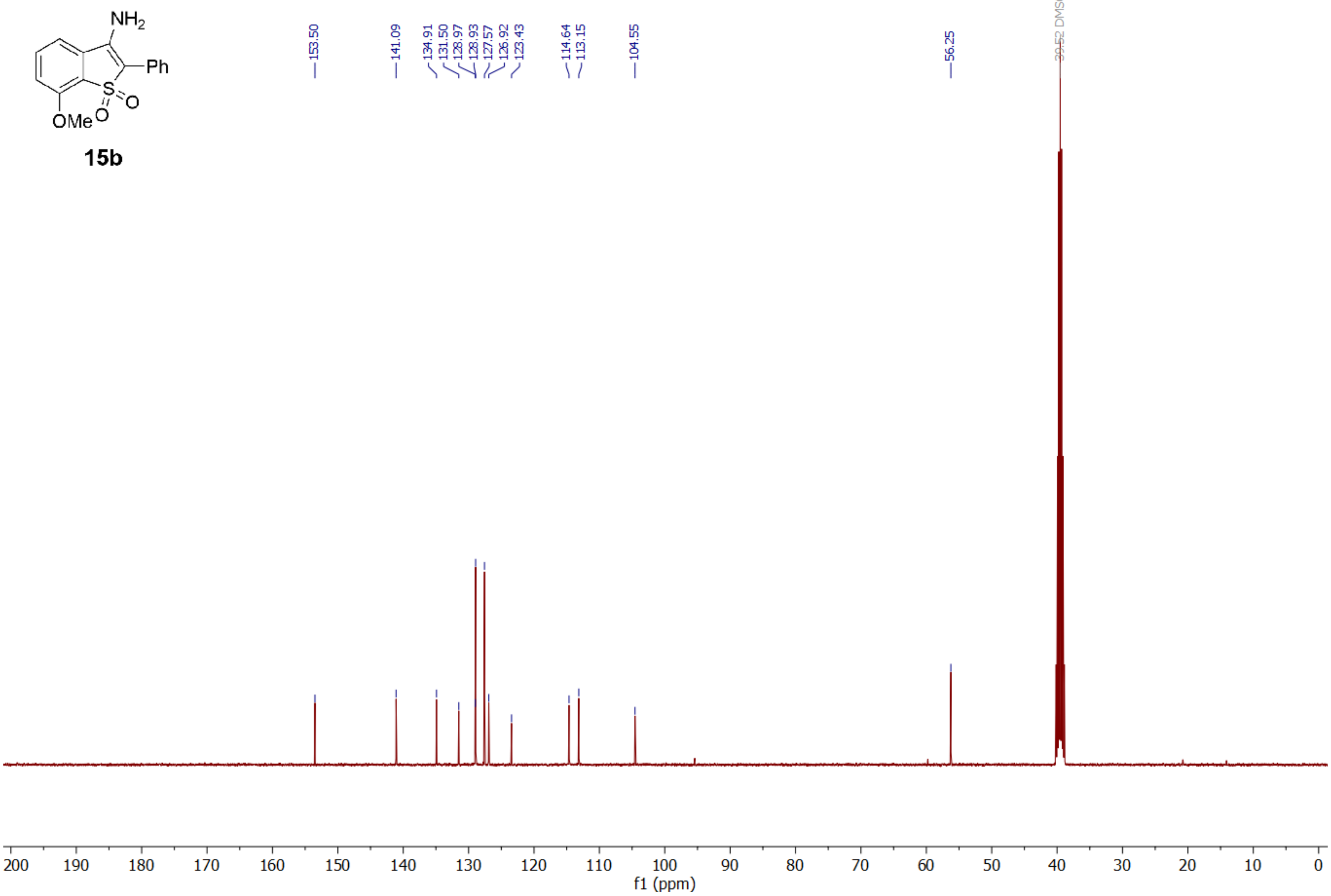

1H (400.15 MHz, CDCl3)

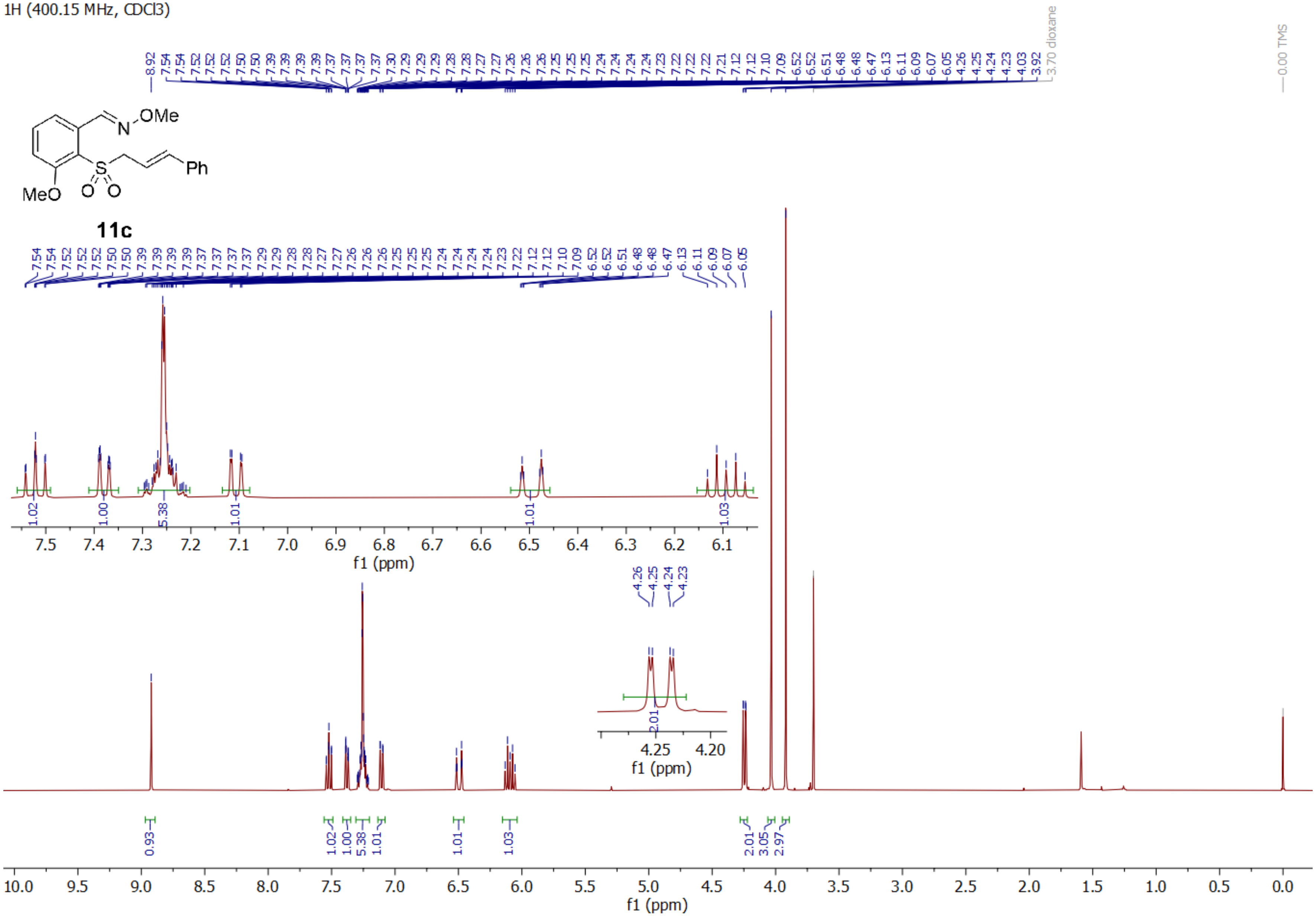

13C (100.63 MHz, CDCl3)

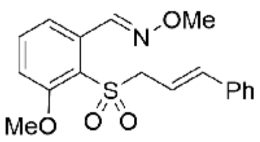

11c

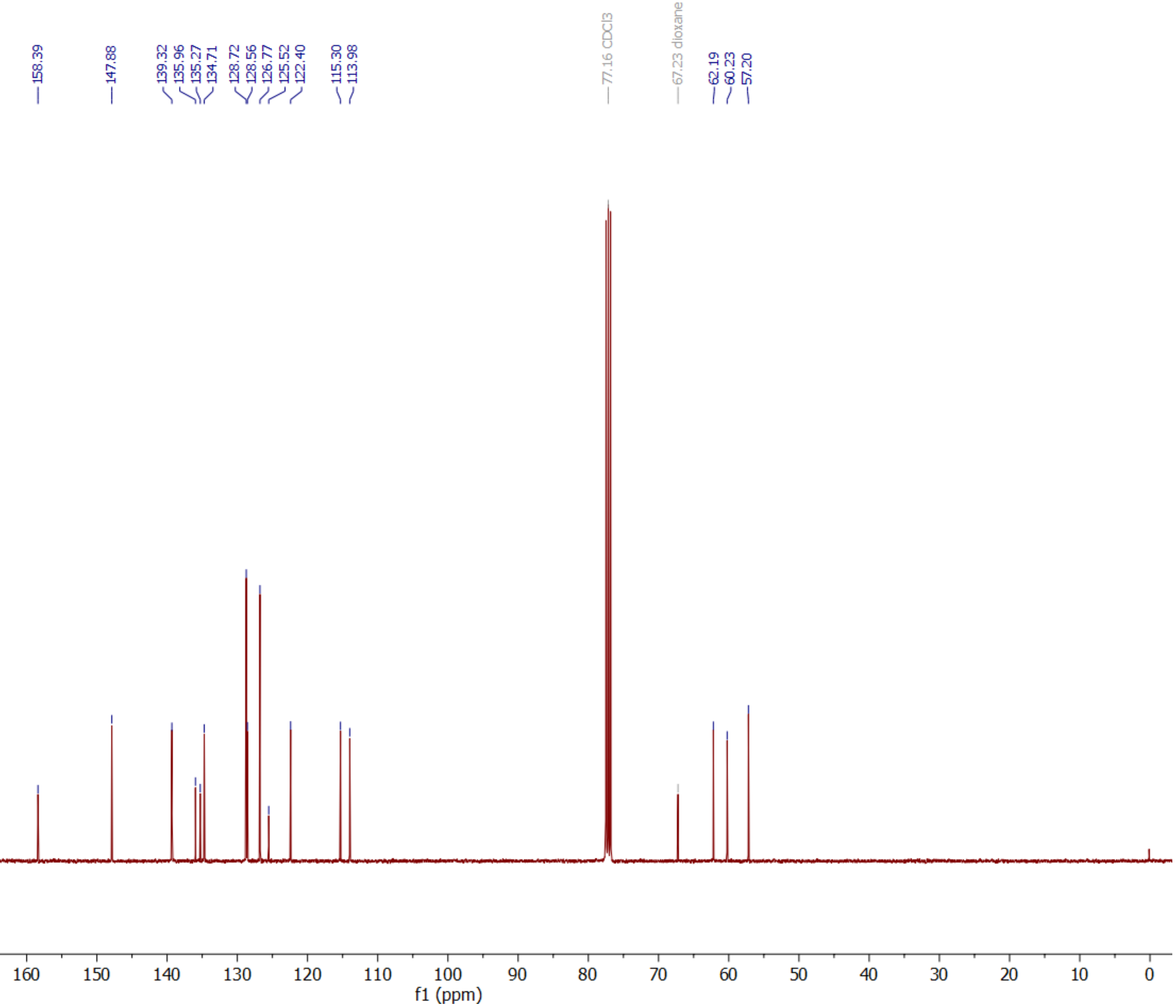

1H (400.15 MHz, DMSO)

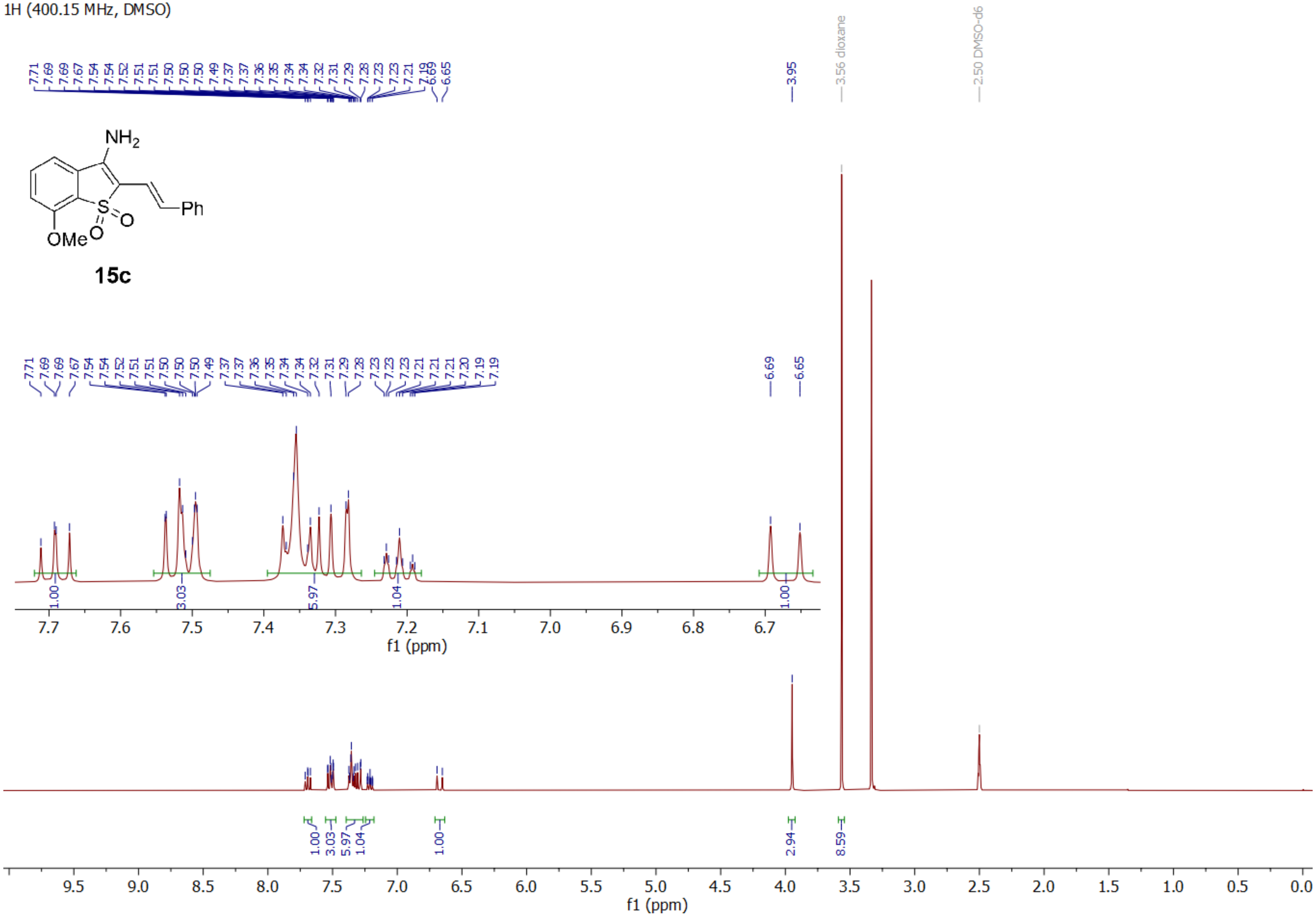

13C (100.63 MHz, DMSO)

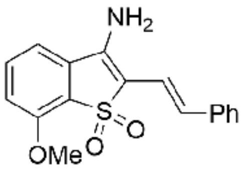

15c

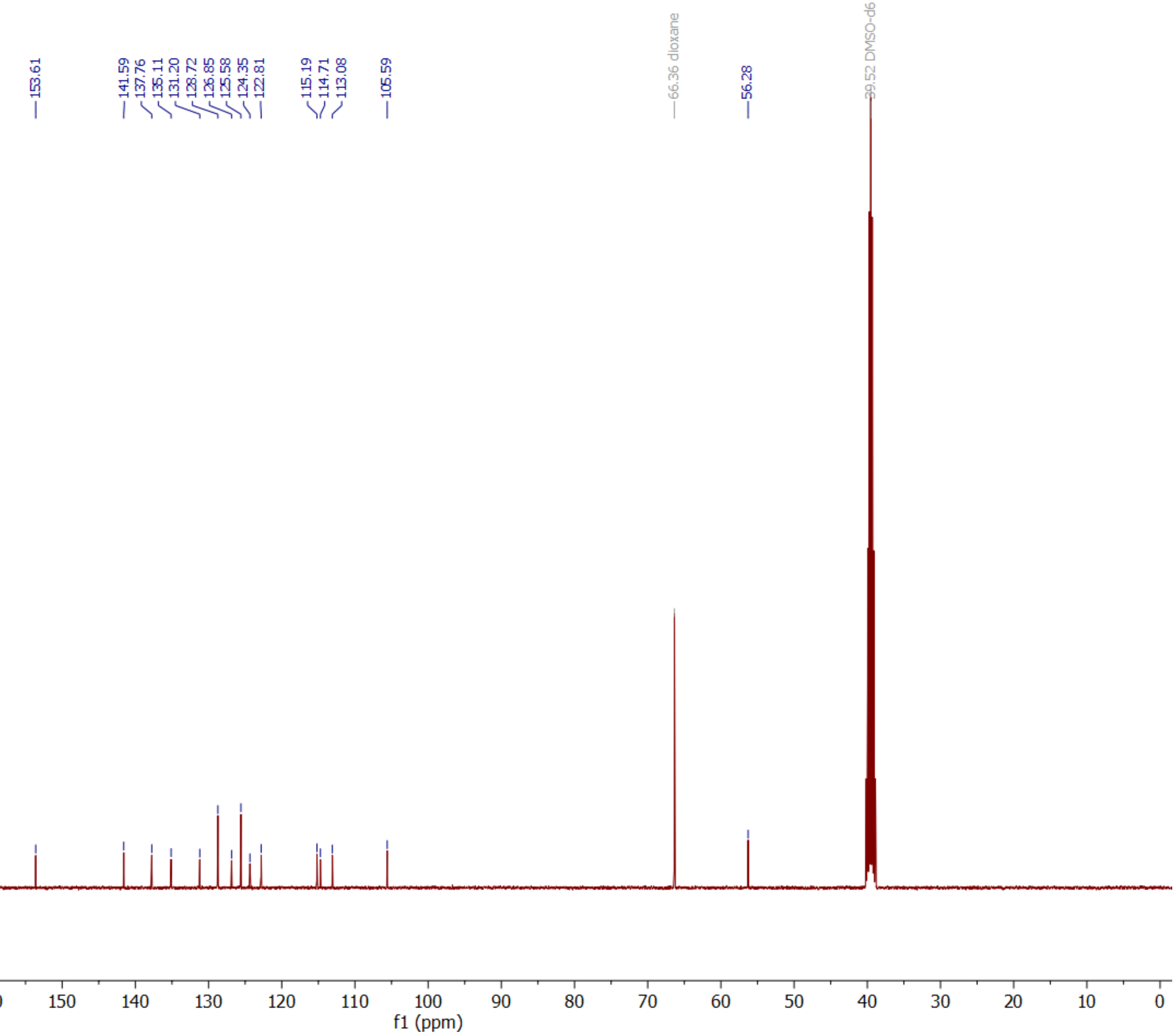

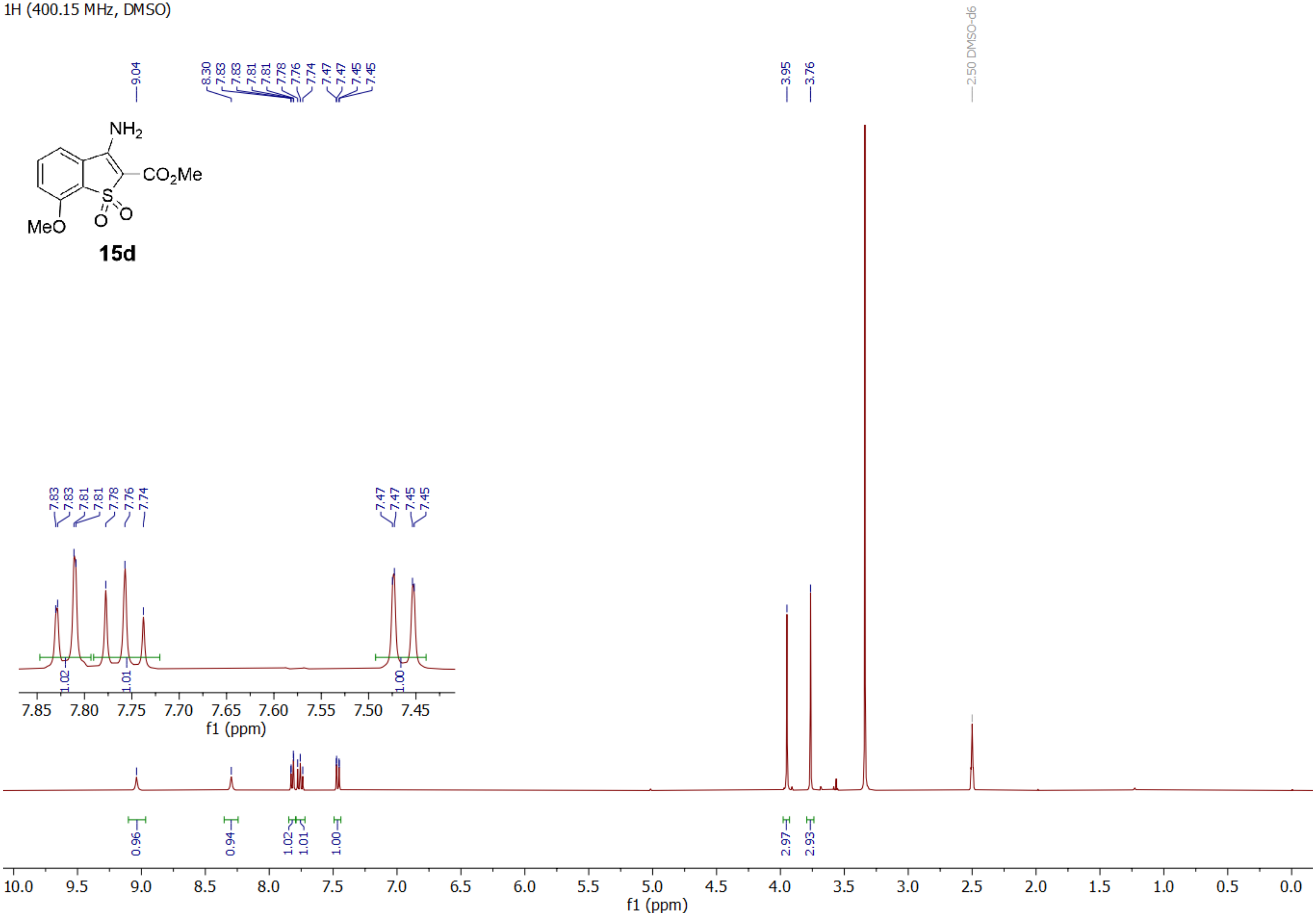
1H (400.15 MHz, DMSO)
NH2
CO2Me
MeO
15d
9.04
8.30 7.83 7.83 7.81 7.81 7.78 7.76 7.74 7.47 7.47 7.45 7.45
3.95
3.76
2.50 DMSO-d6
0.96
0.94
1.02
1.01
1.00
2.97
2.93
f1 (ppm)

13C (100.63 MHz, DMSO)

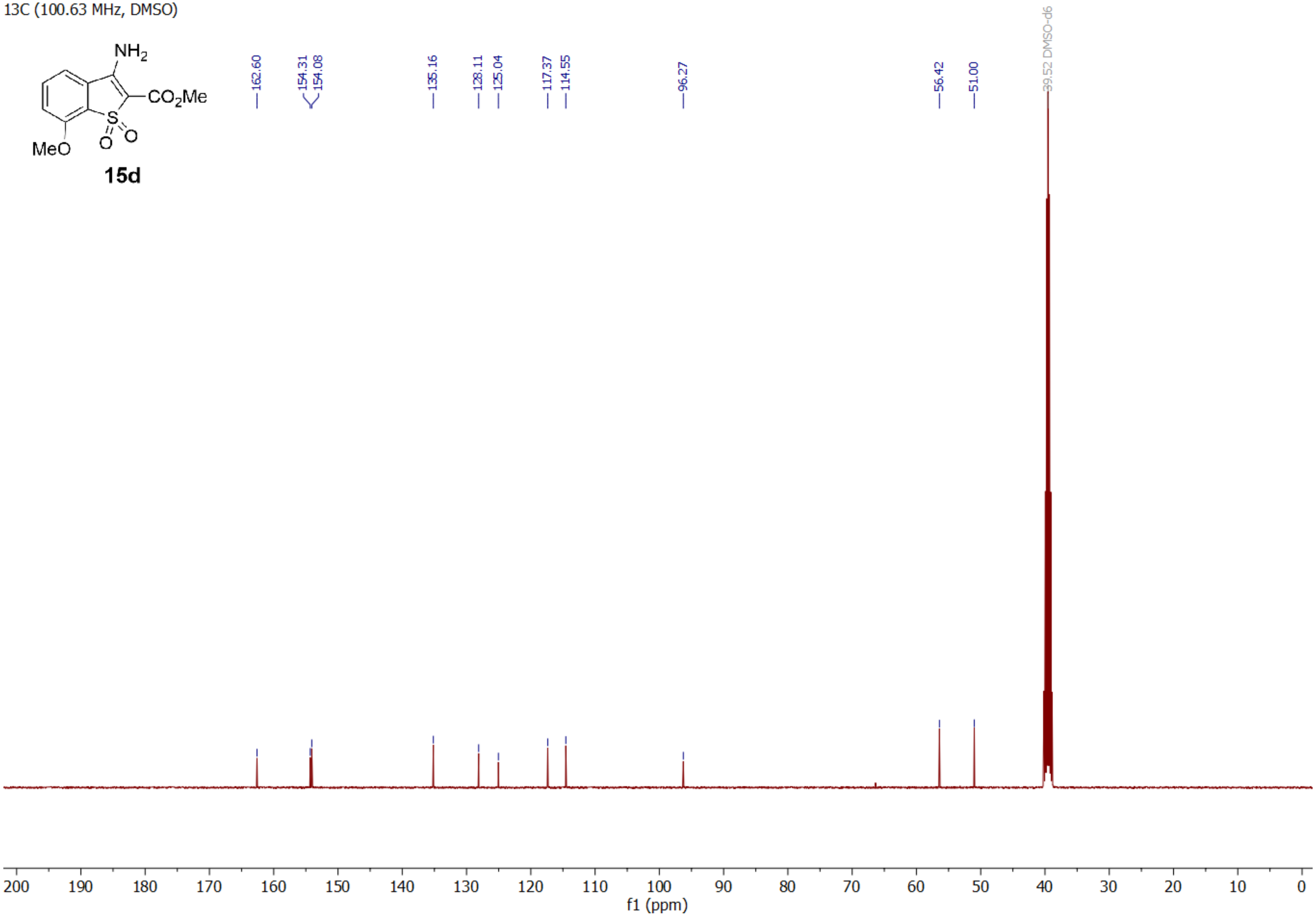

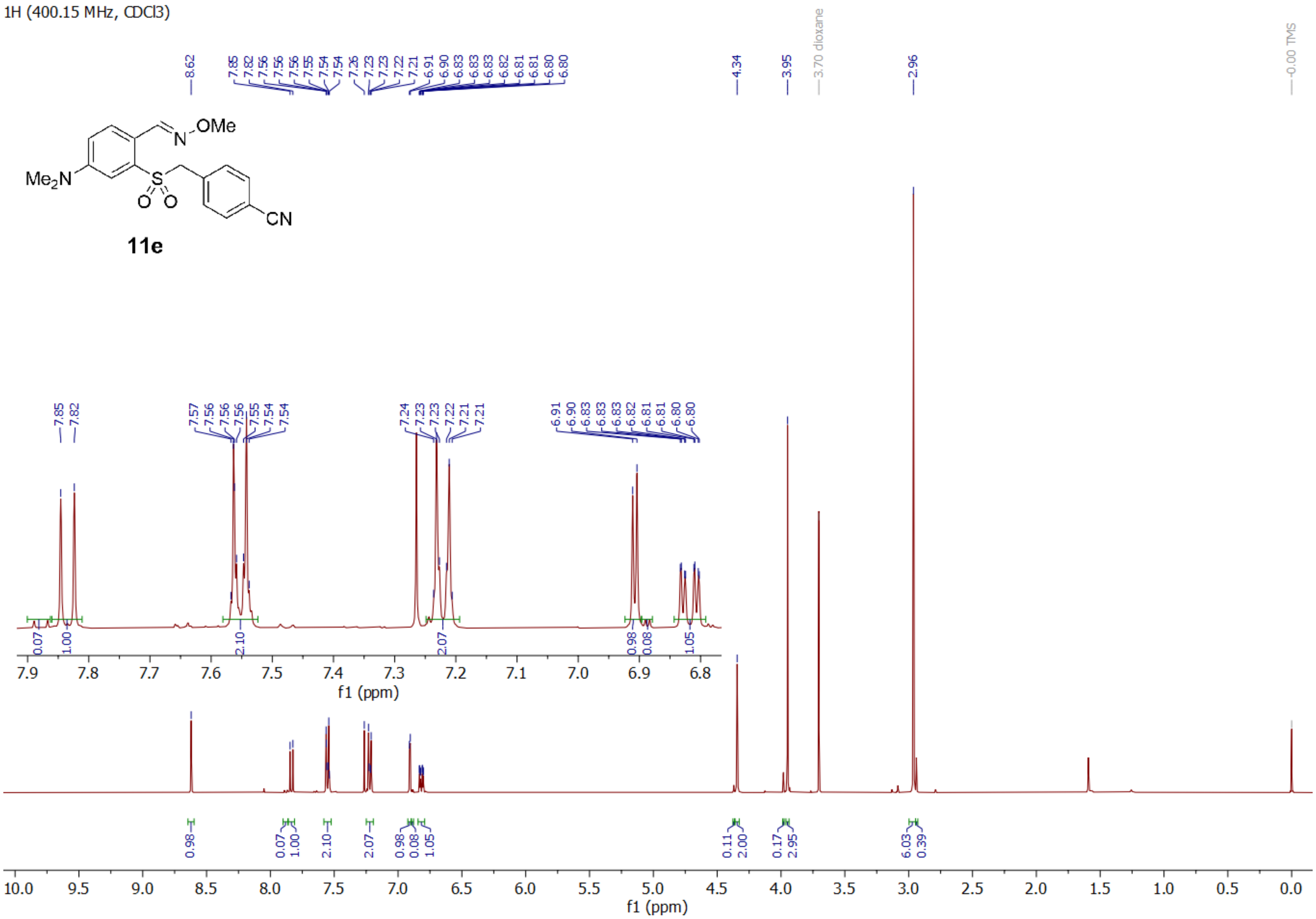

11e

13C (100.63 MHz, CDCl3)

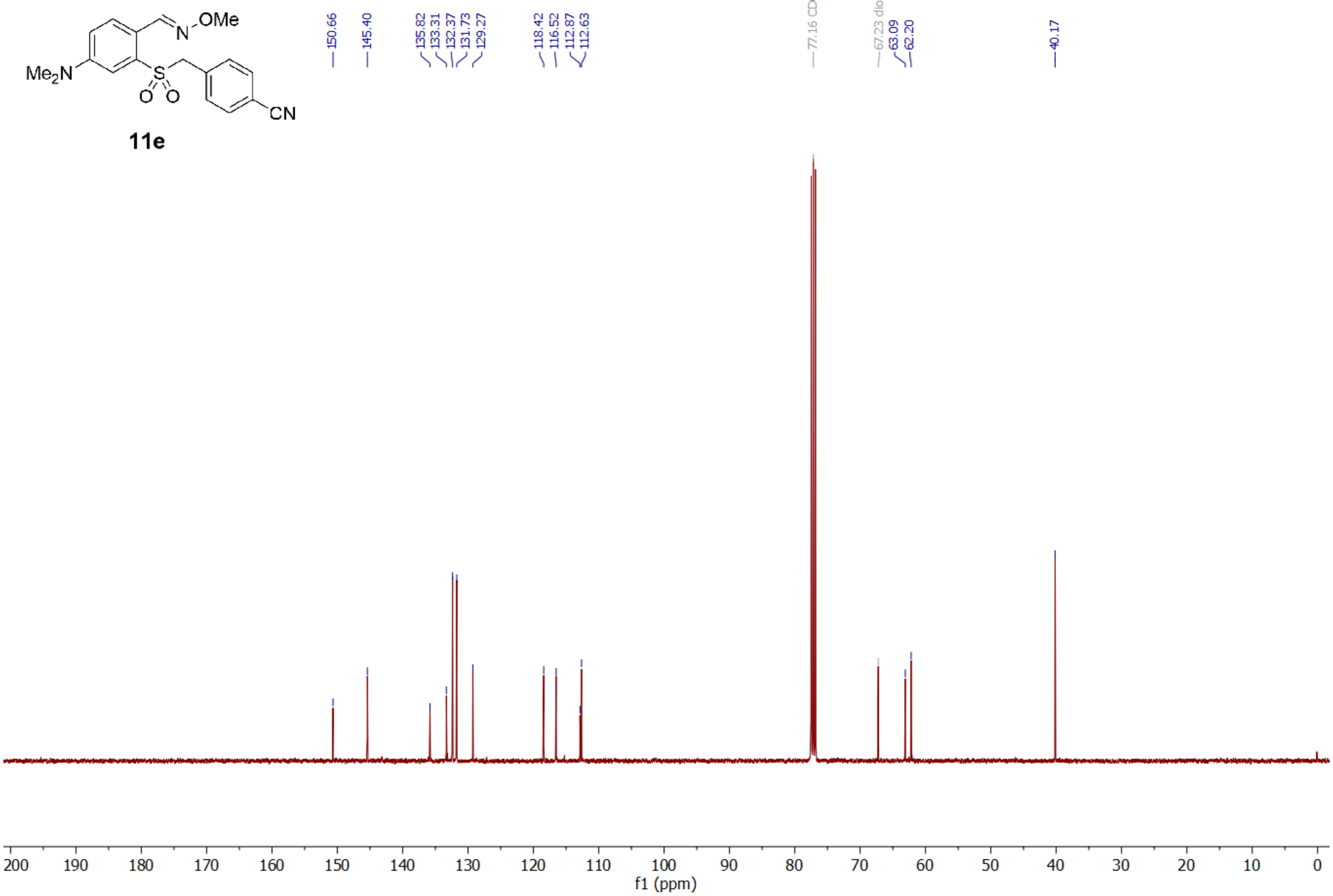

1H (400.15 MHz, DMSO)

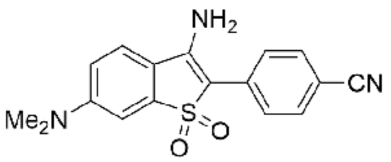

15e

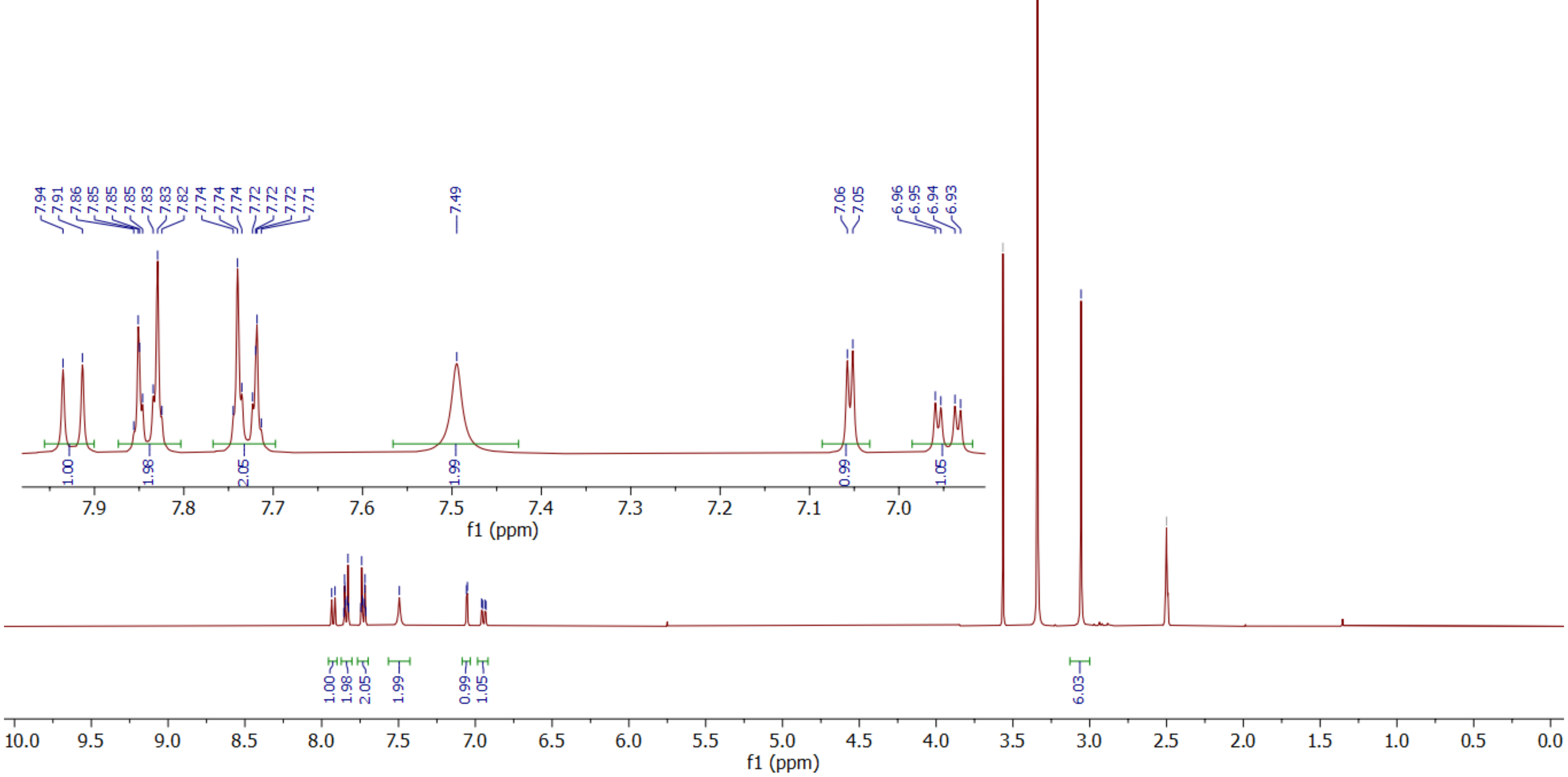

13C (100.63 MHz, DMSO)

15e

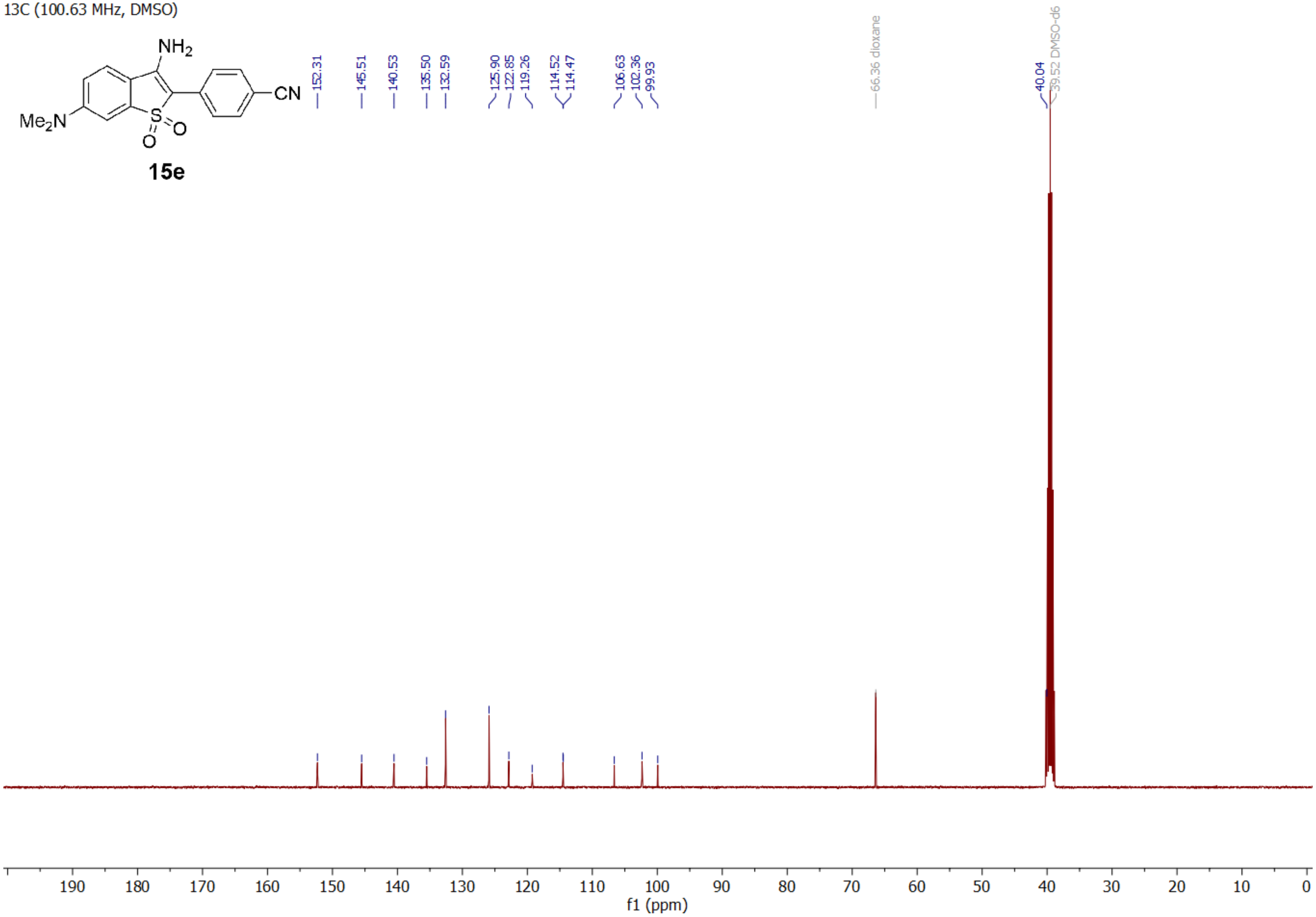

1H (400.15 MHz, CDCl3)

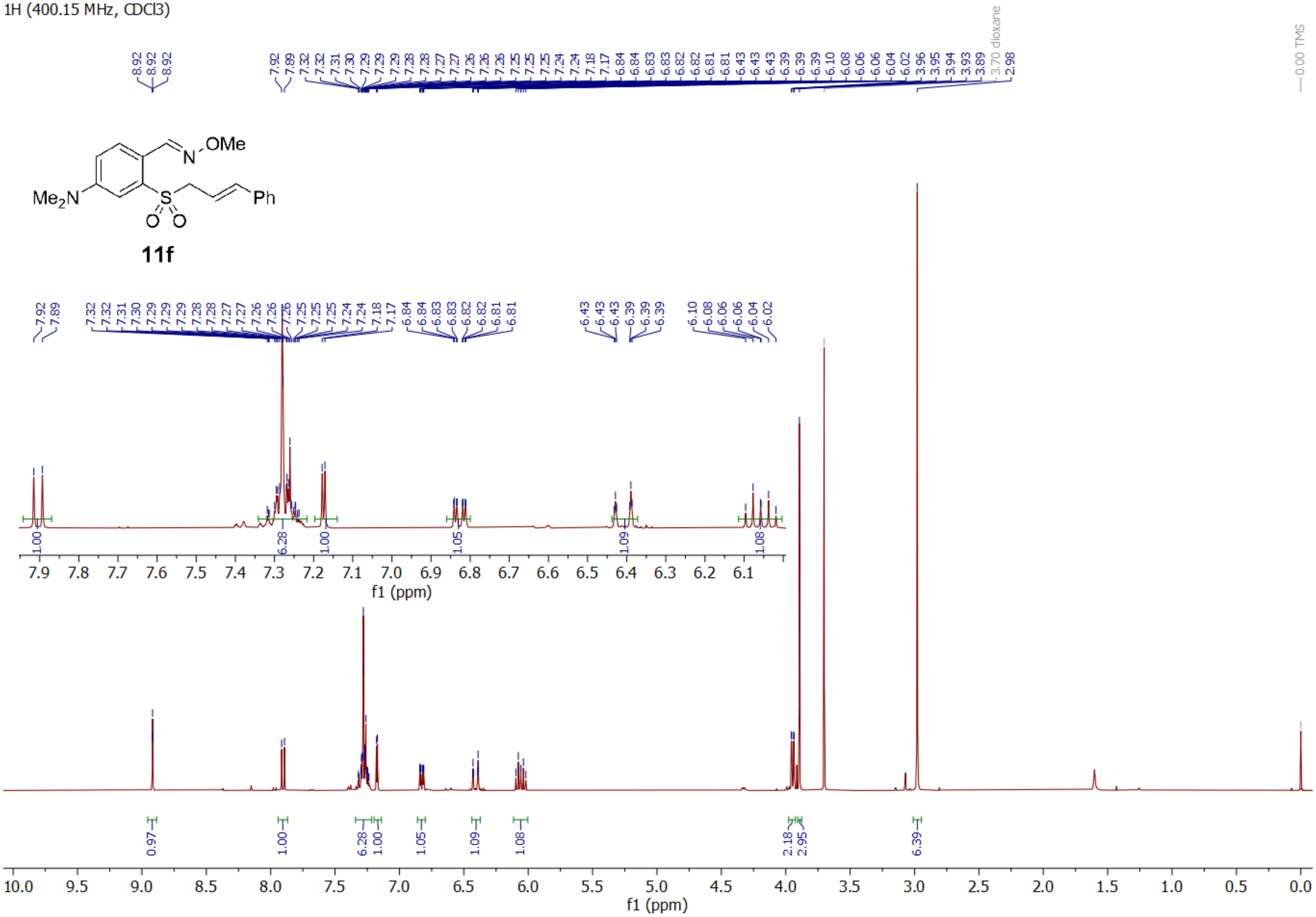

13C (100.63 MHz, CDCl3)

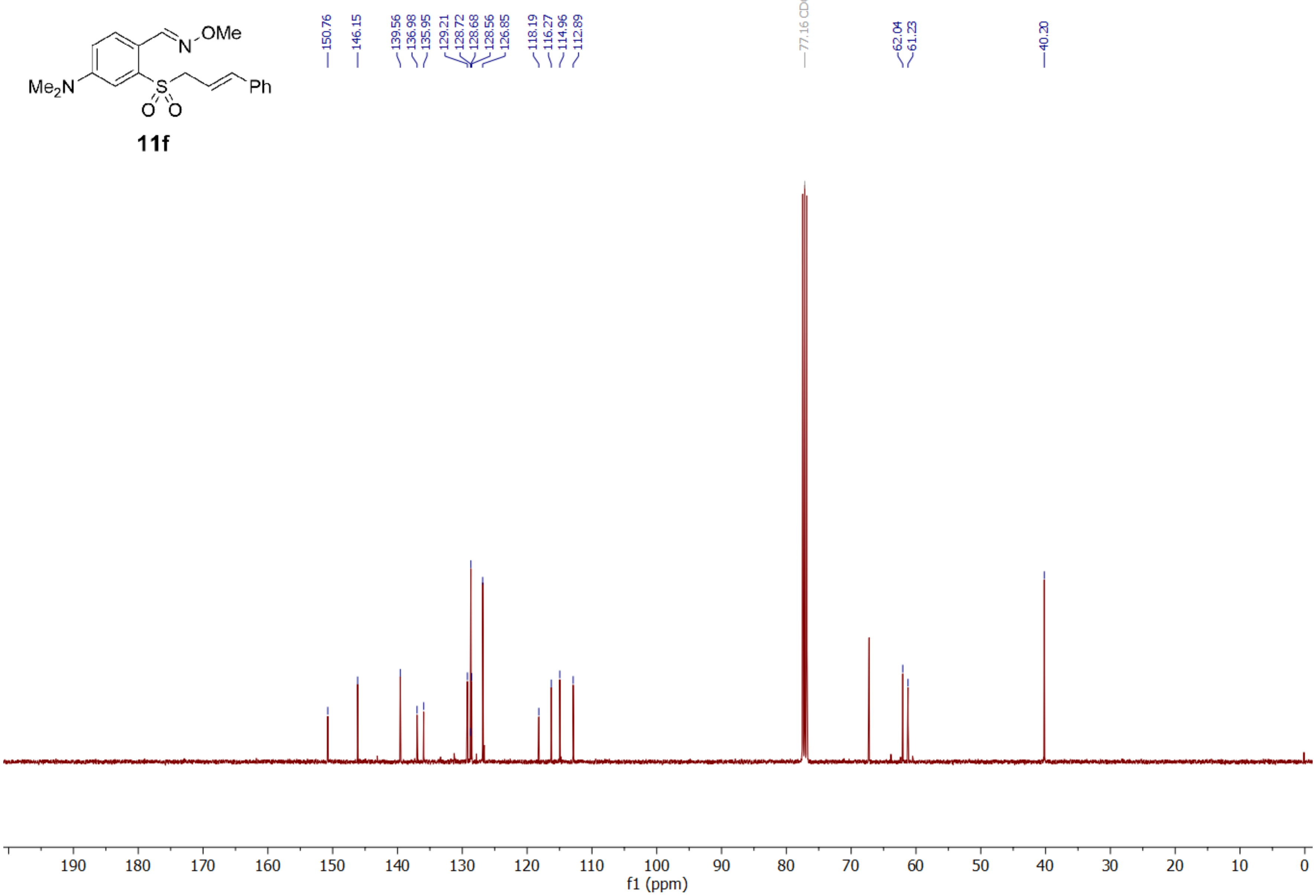

11f

1H (400.15 MHz, DMSO)

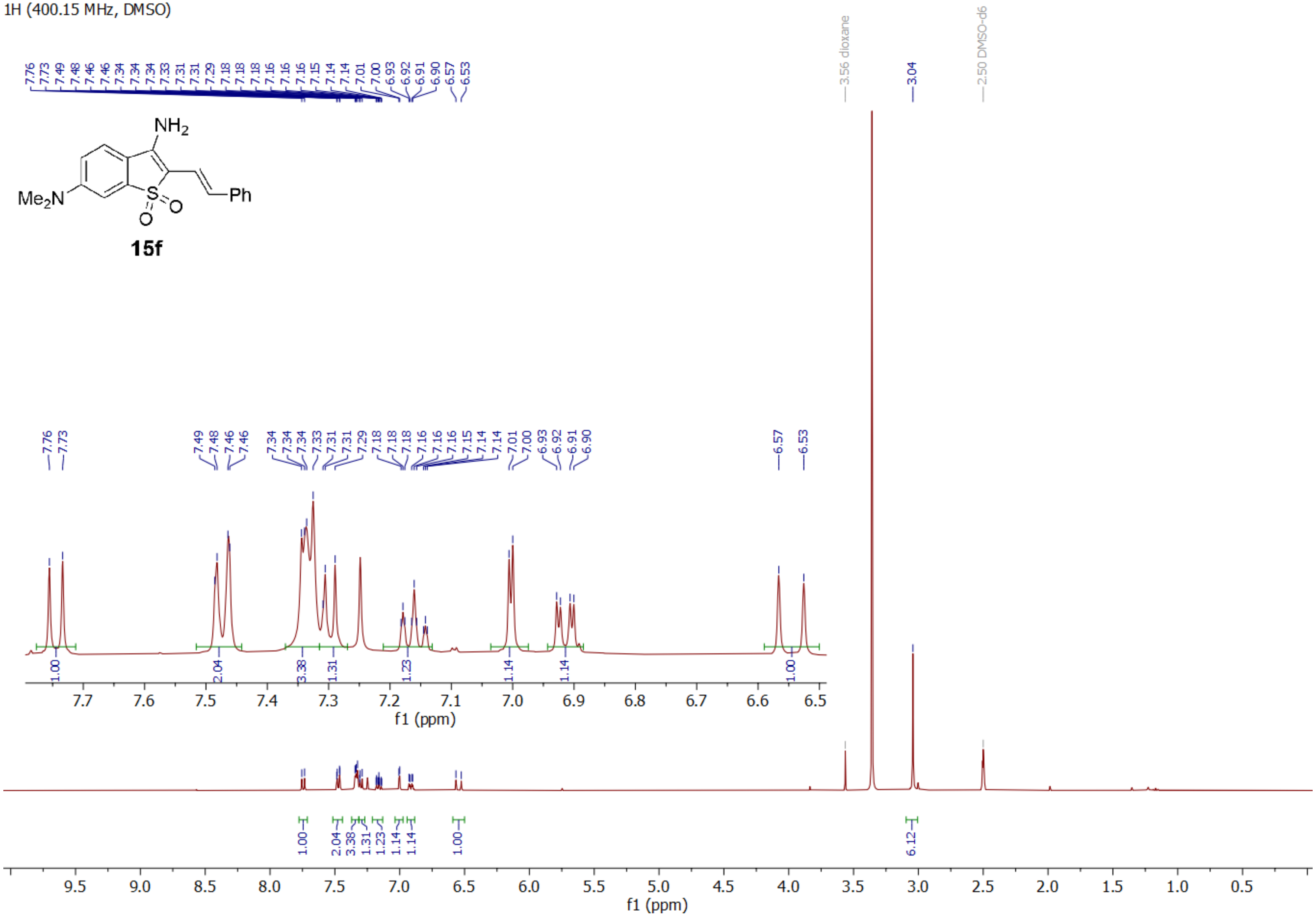

13C (100.63 MHz, DMSO)

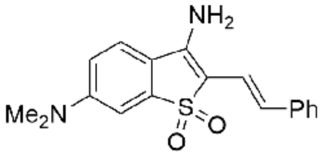

15f

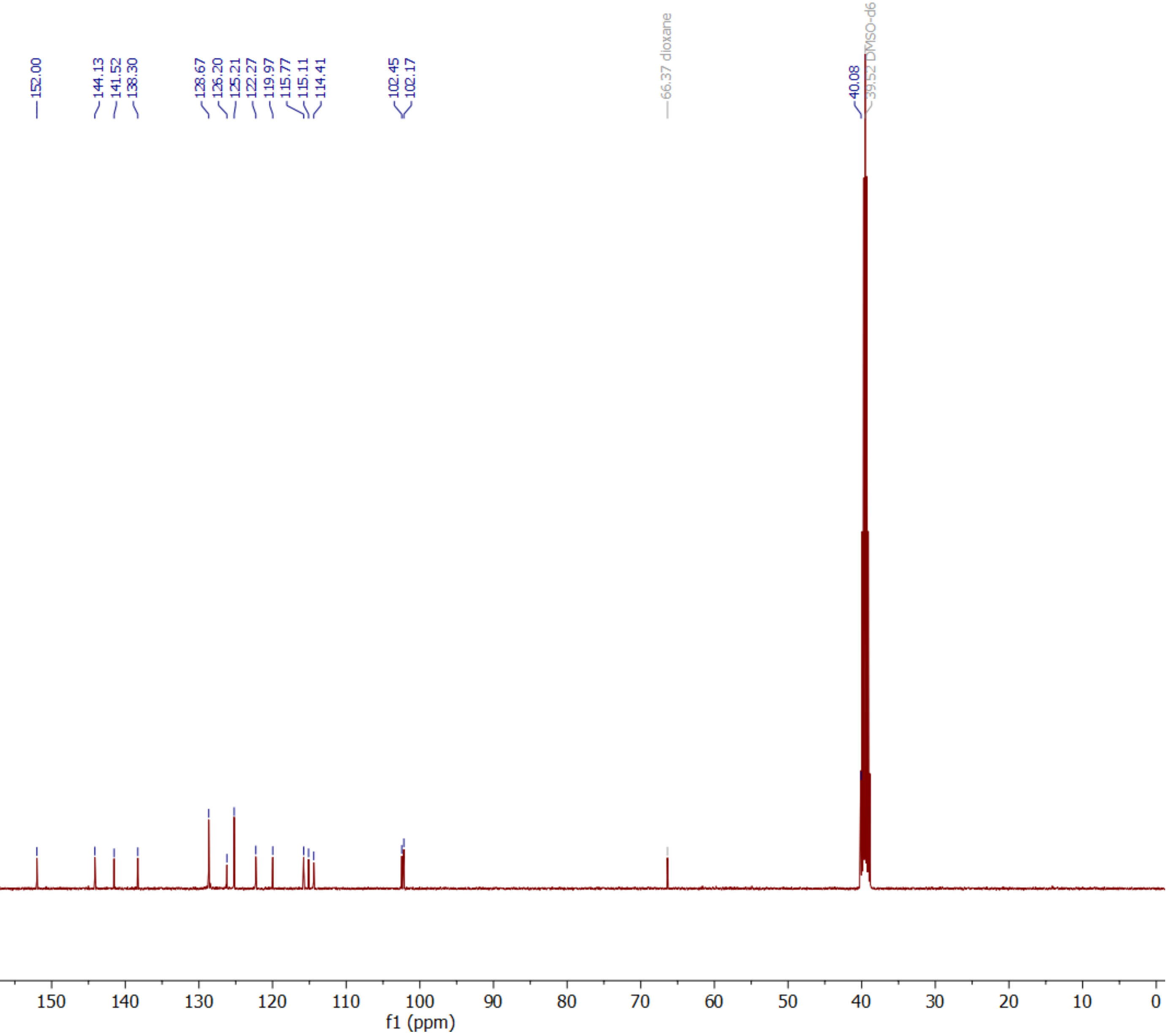

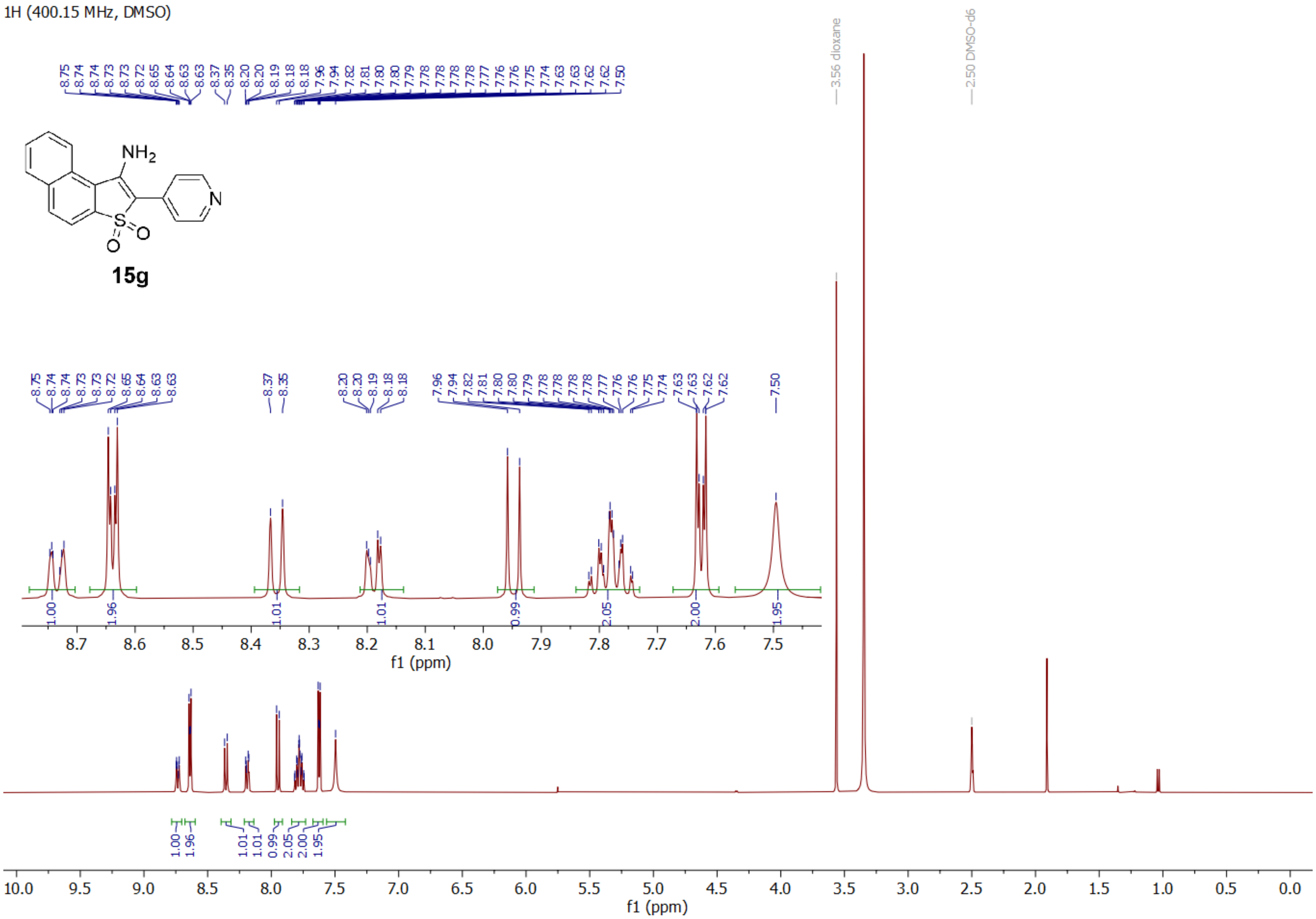
1H (400.15 MHz, DMSO)
8.75 8.74 8.74 8.73 8.73 8.72 8.65 8.64 8.63 8.63 8.37 8.35 8.20 8.20 8.19 8.18 8.18 7.96 7.94 7.82 7.81 7.80 7.80 7.79 7.78 7.78 7.78 7.78 7.77 7.76 7.76 7.75 7.74 7.63 7.63 7.62 7.62 7.50
3.56 dioxane
2.50 DMSO-d6
NH2
N
S
O
O
15g
1.00 1.96 1.01 1.01 0.99 2.05 2.00 1.95
f1 (ppm)

13C (100.63 MHz, DMSO)

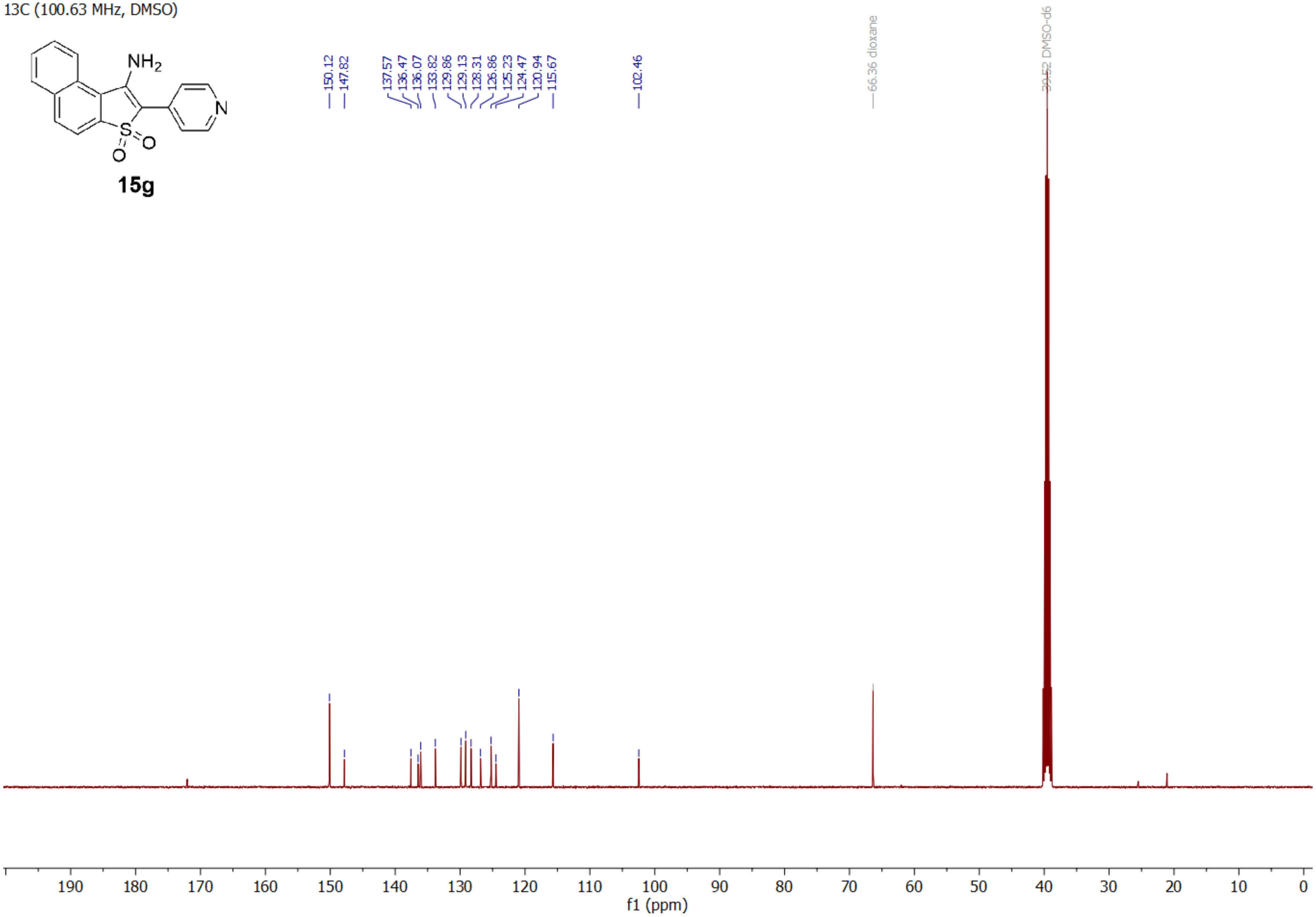

1H (400.15 MHz, CDCl3)

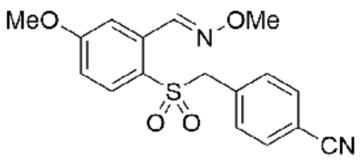

11h

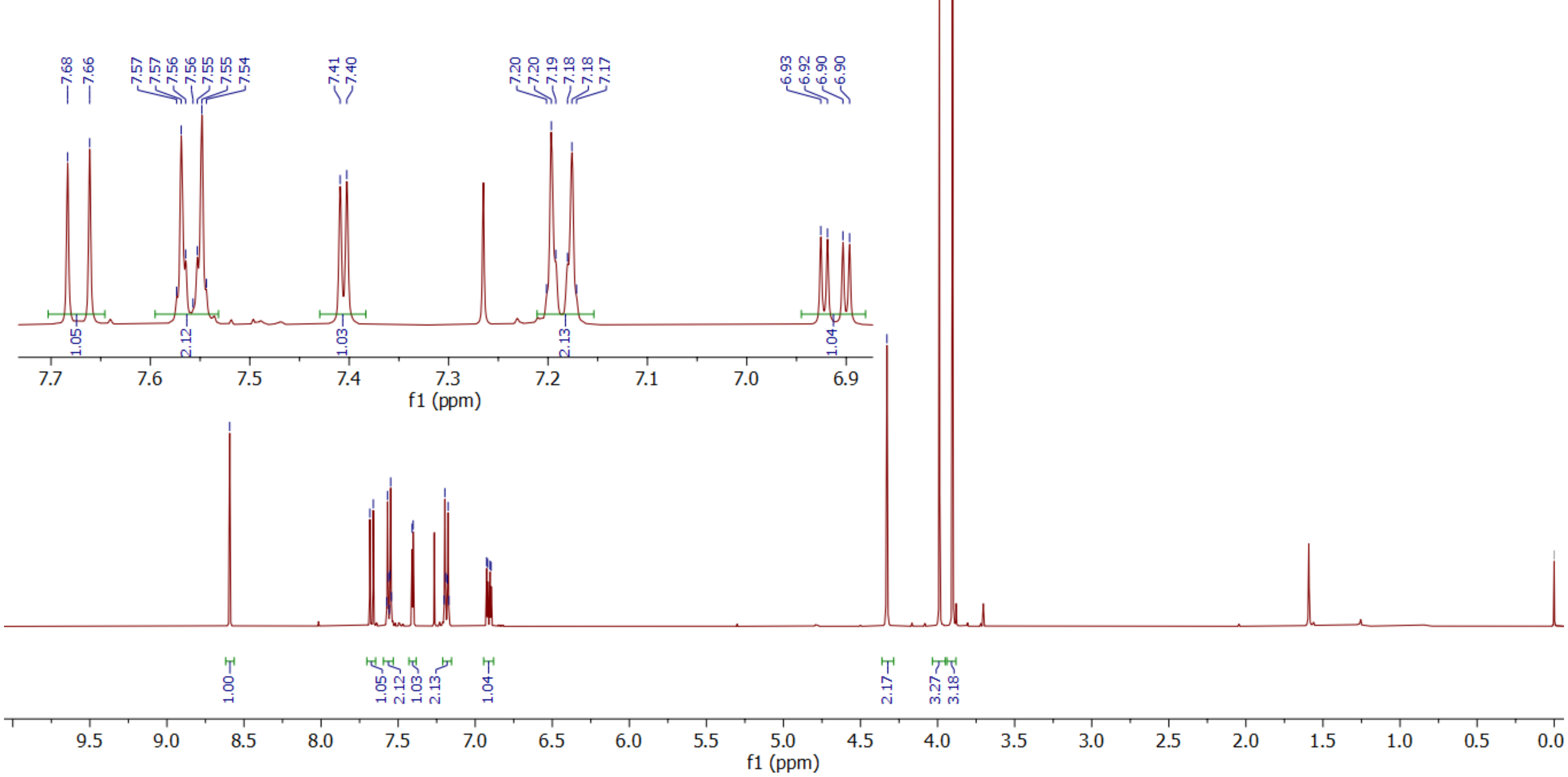

13C (100.63 MHz, CDCl3)

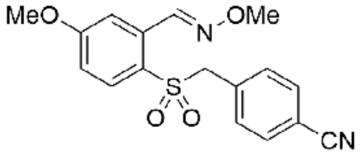

11h

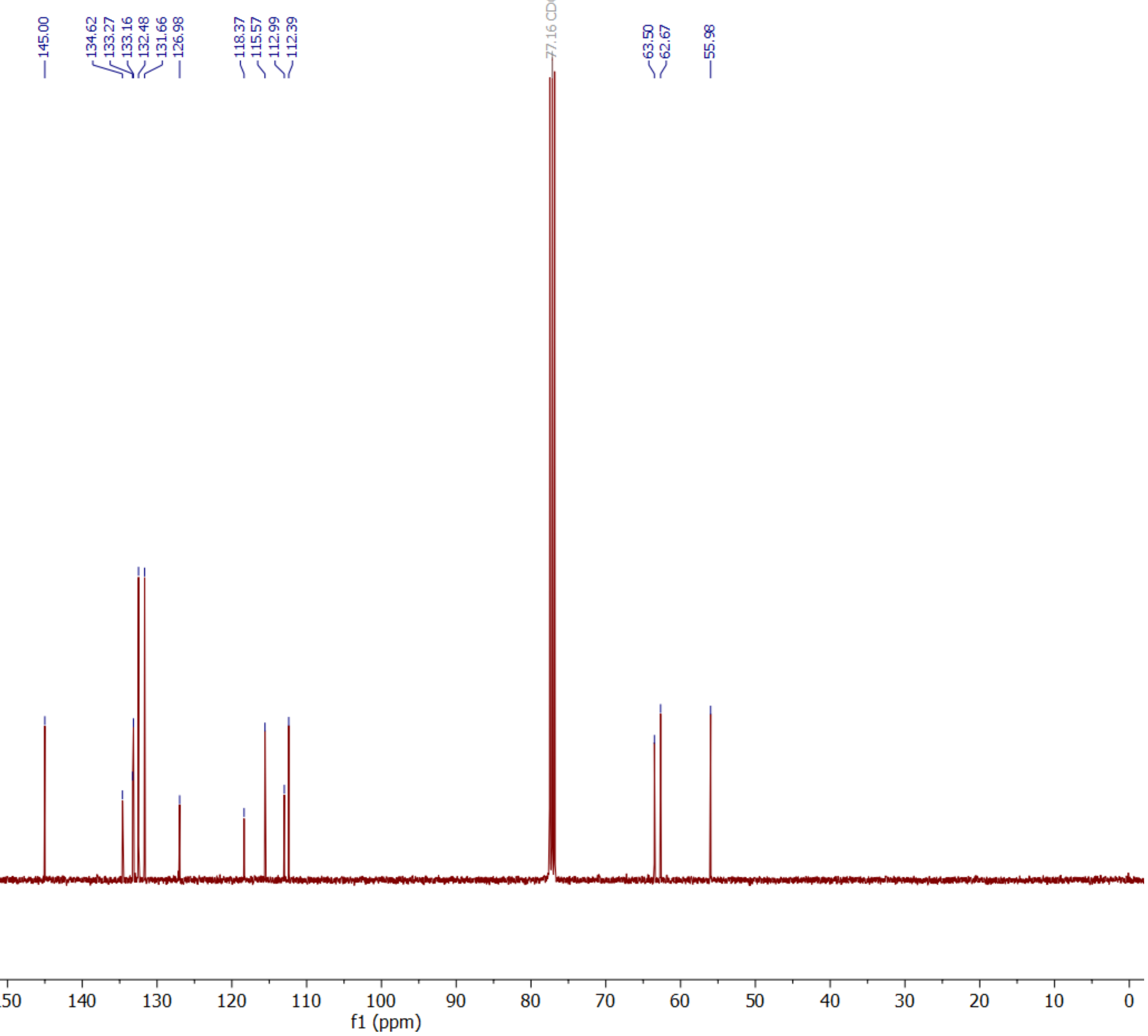

1H (400.15 MHz, Pyridine-d5)

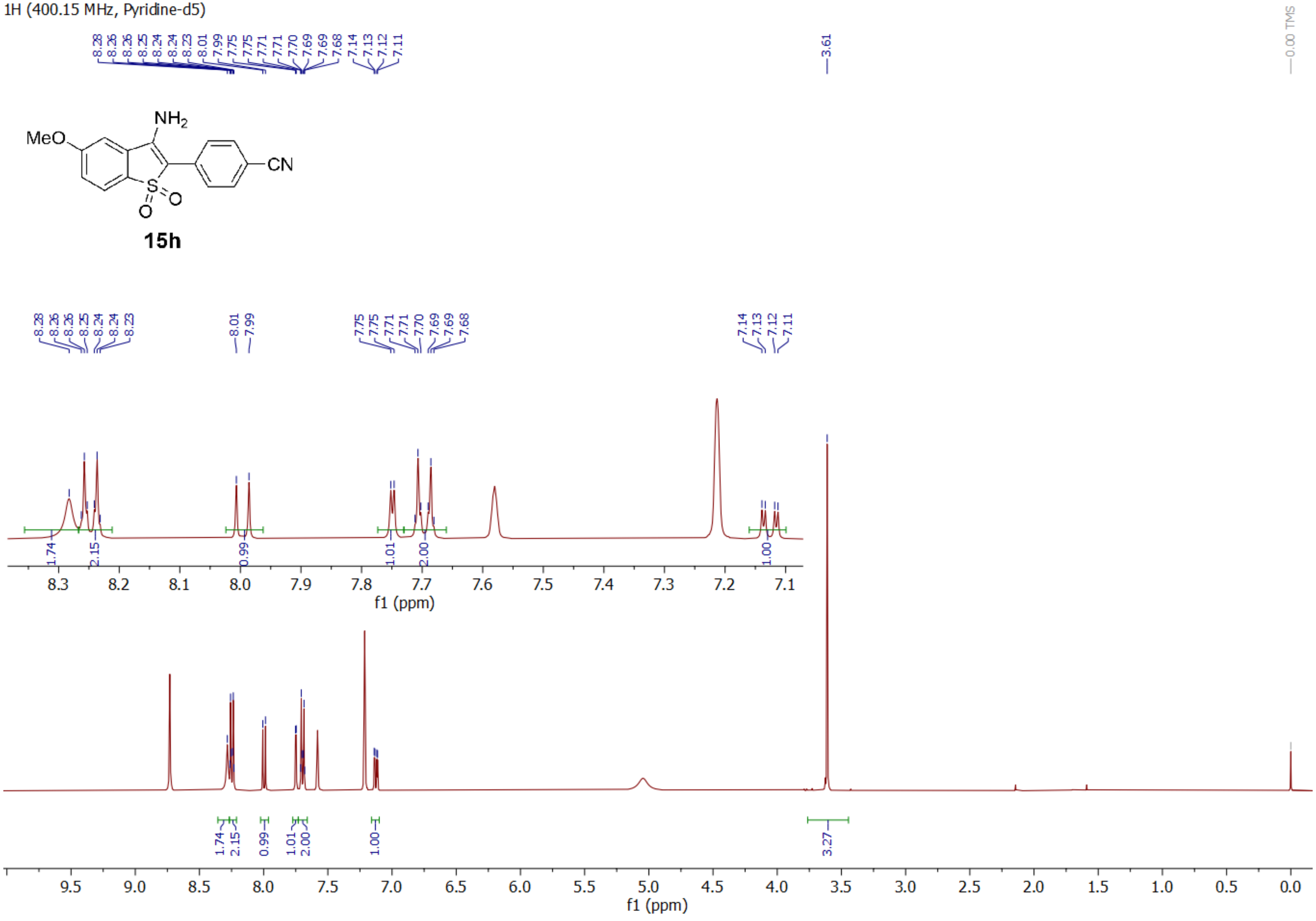

13C (100.63 MHz, Pyridine-d5)

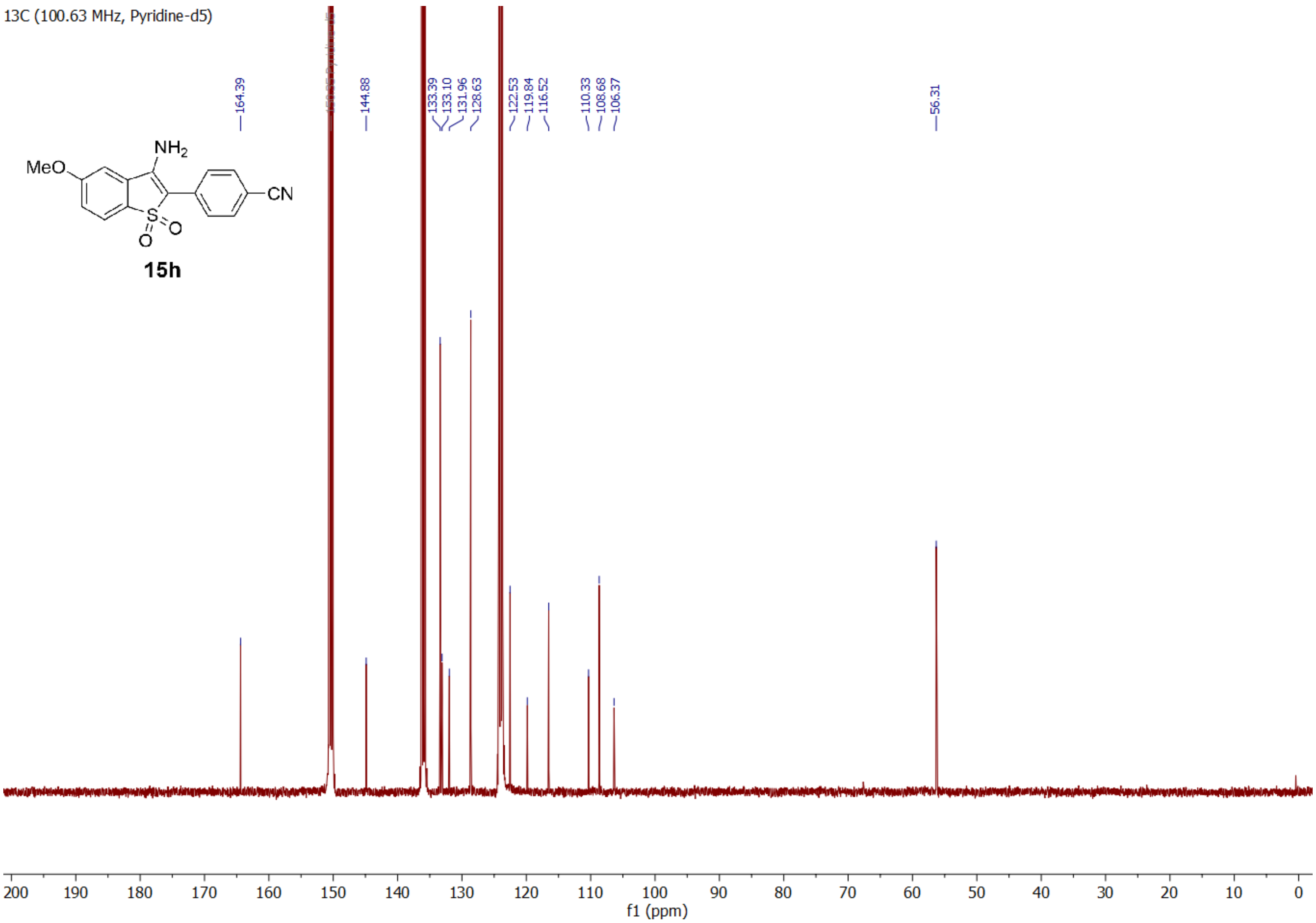

15h

1H (400.15 MHz, DMSO)

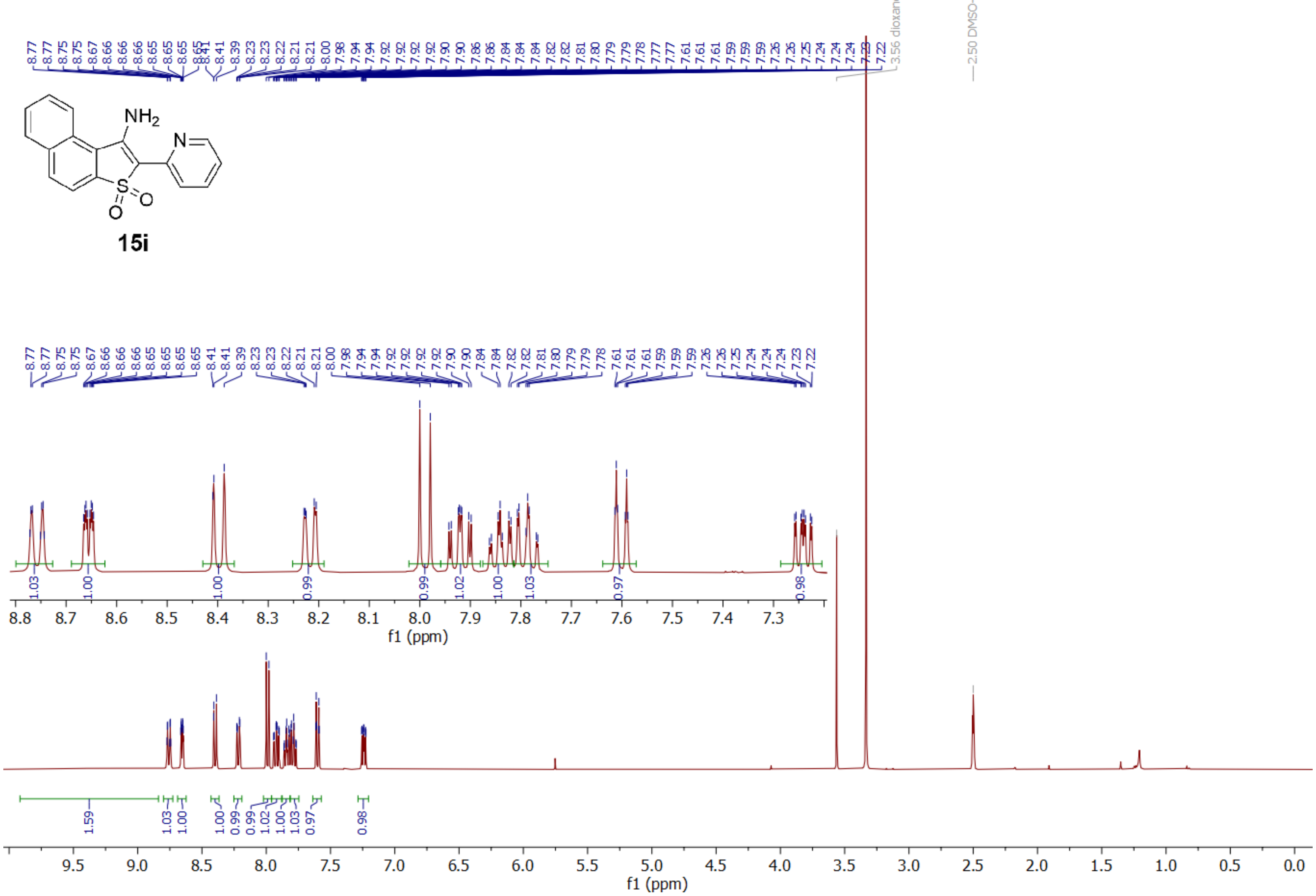

13C (100.63 MHz, DMSO)

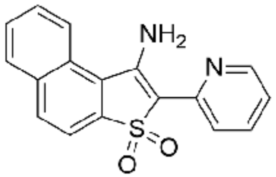

15i

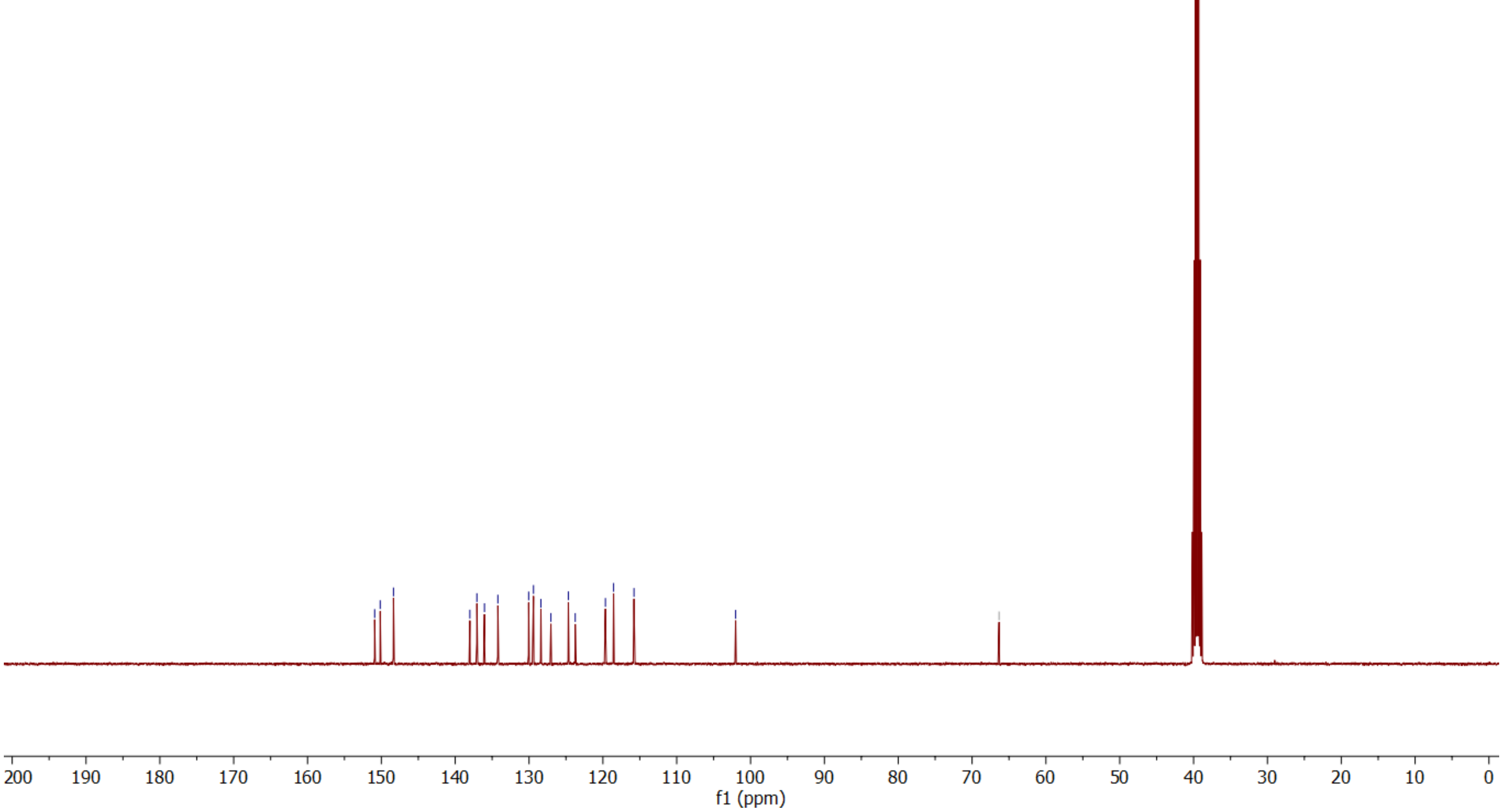

1H (400.15 MHz, CDCl3)

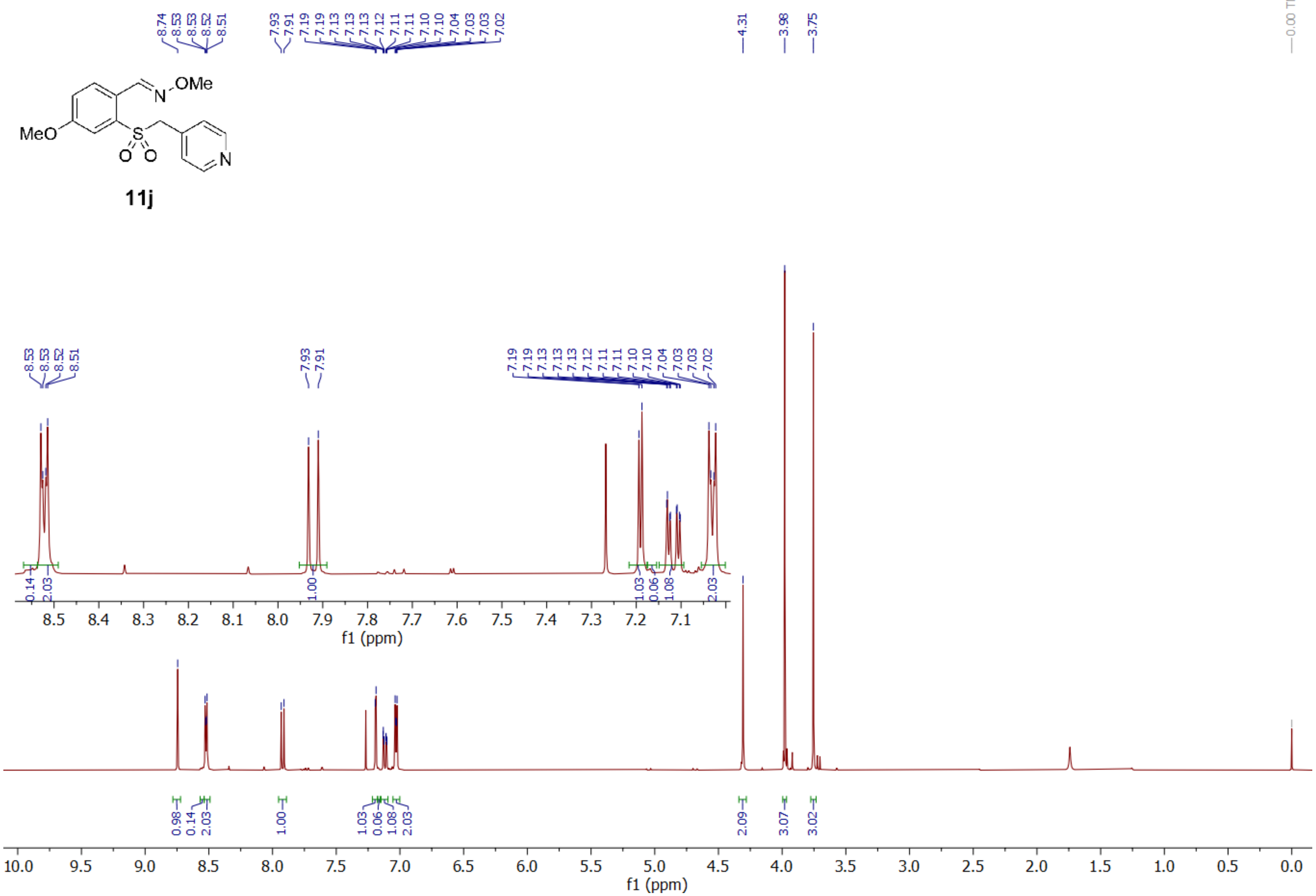

13C (100.63 MHz, CDCl3)

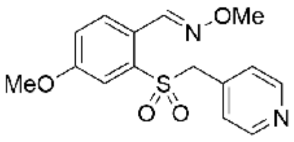

11j

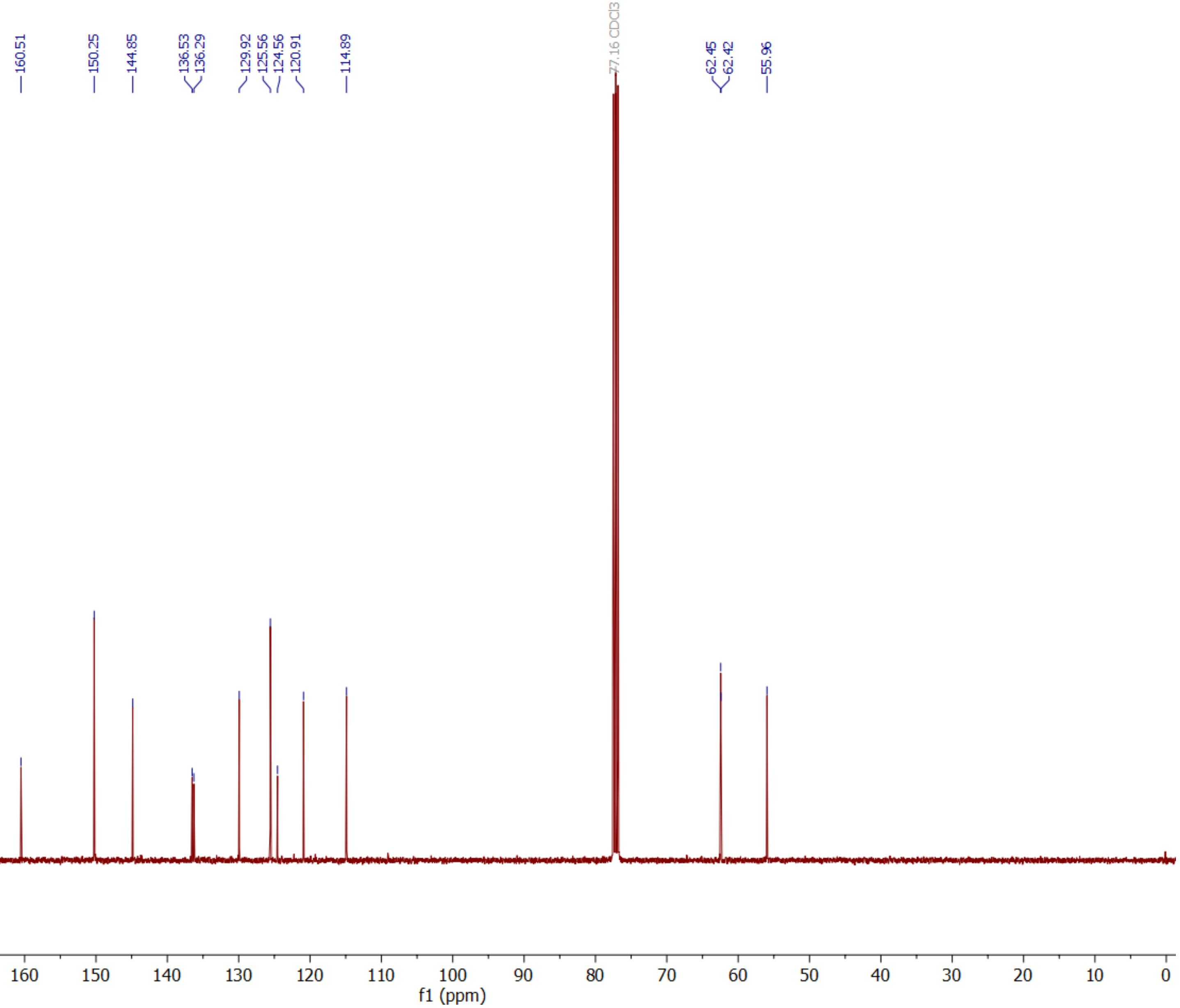

1H (400.15 MHz, DMSO)

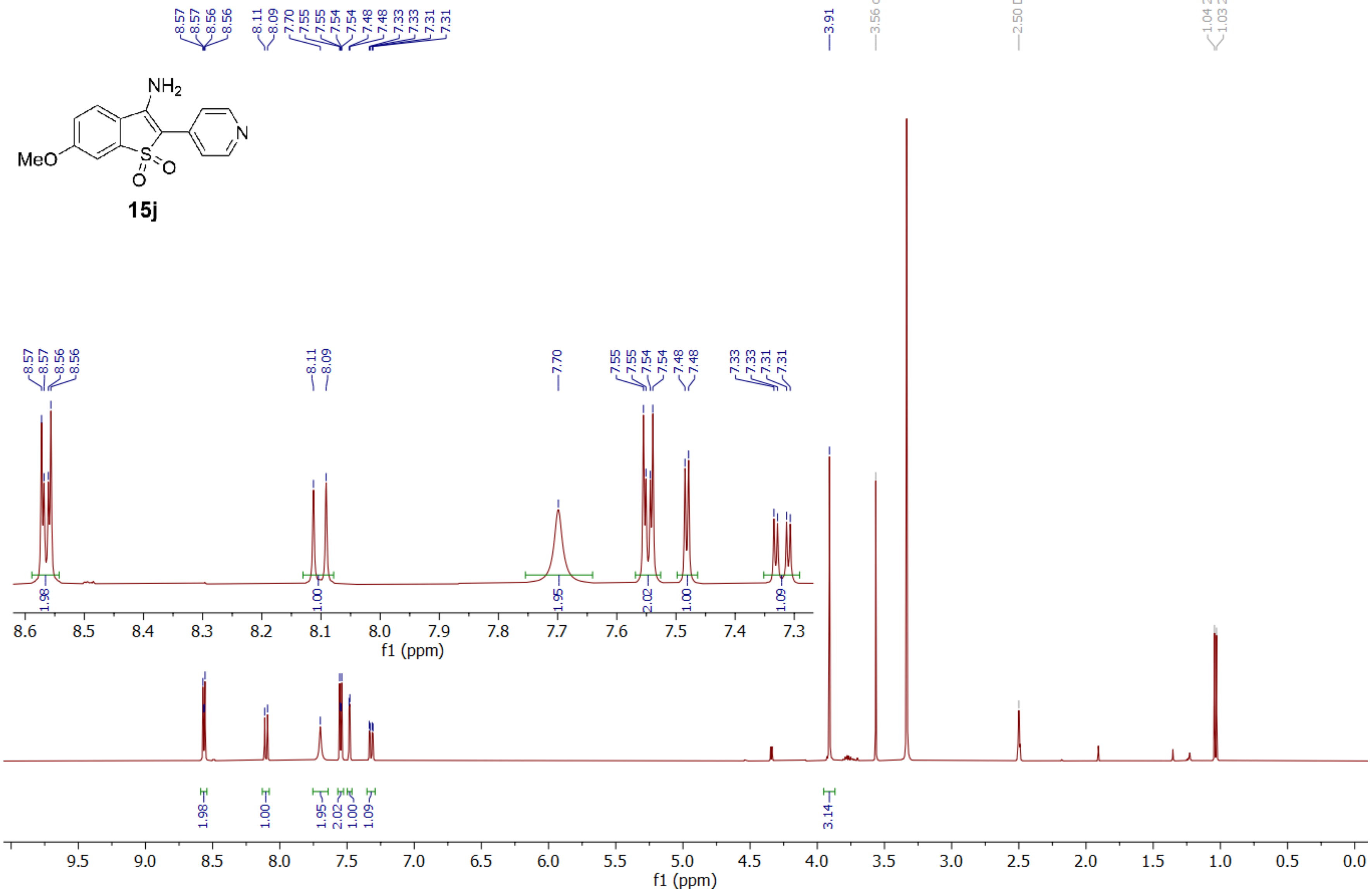

13C (100.63 MHz, DMSO)

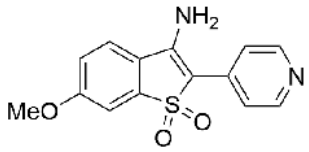

15j

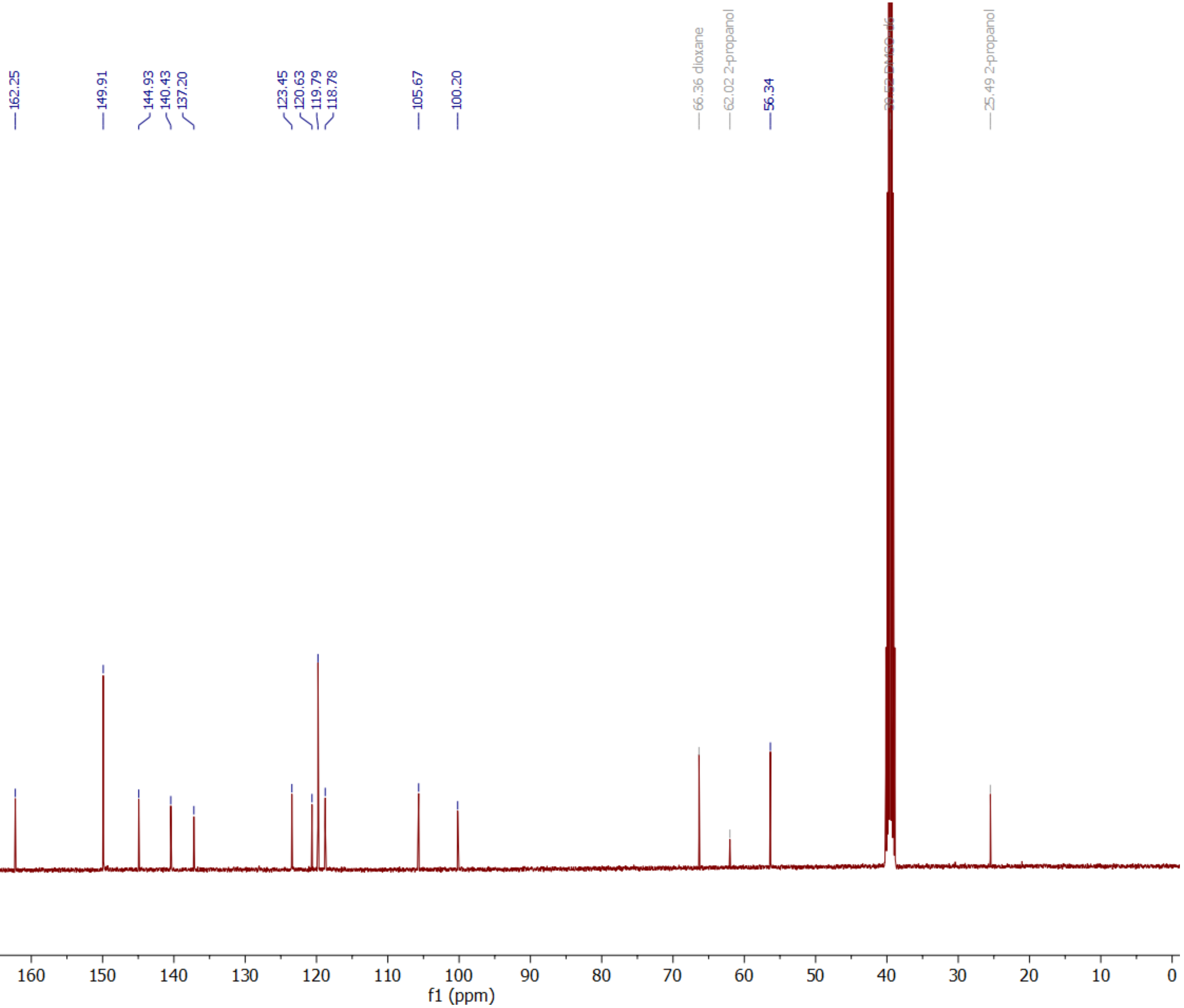

1H (400.15 MHz, CDCl3)

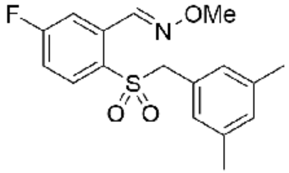

11k

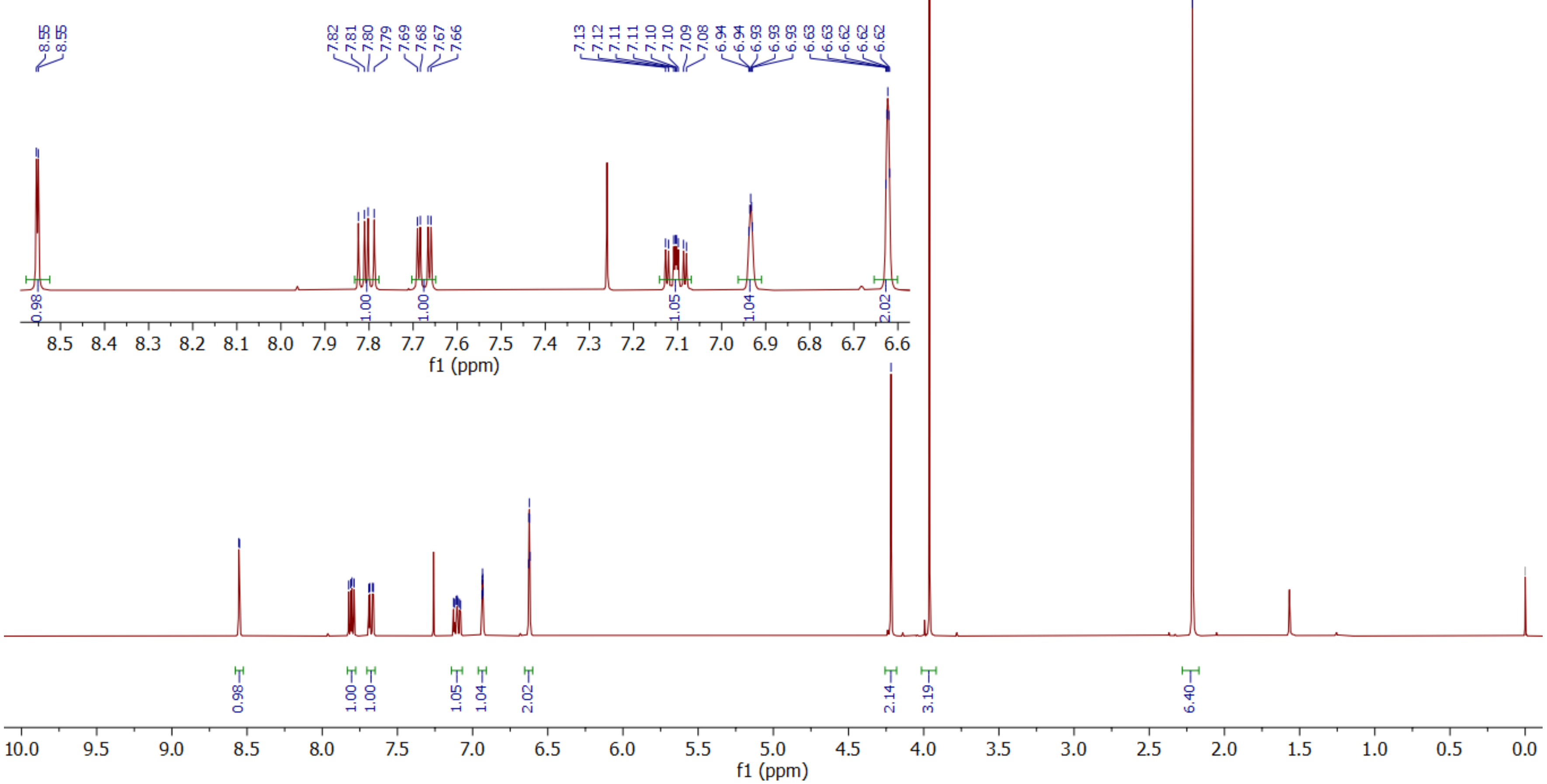

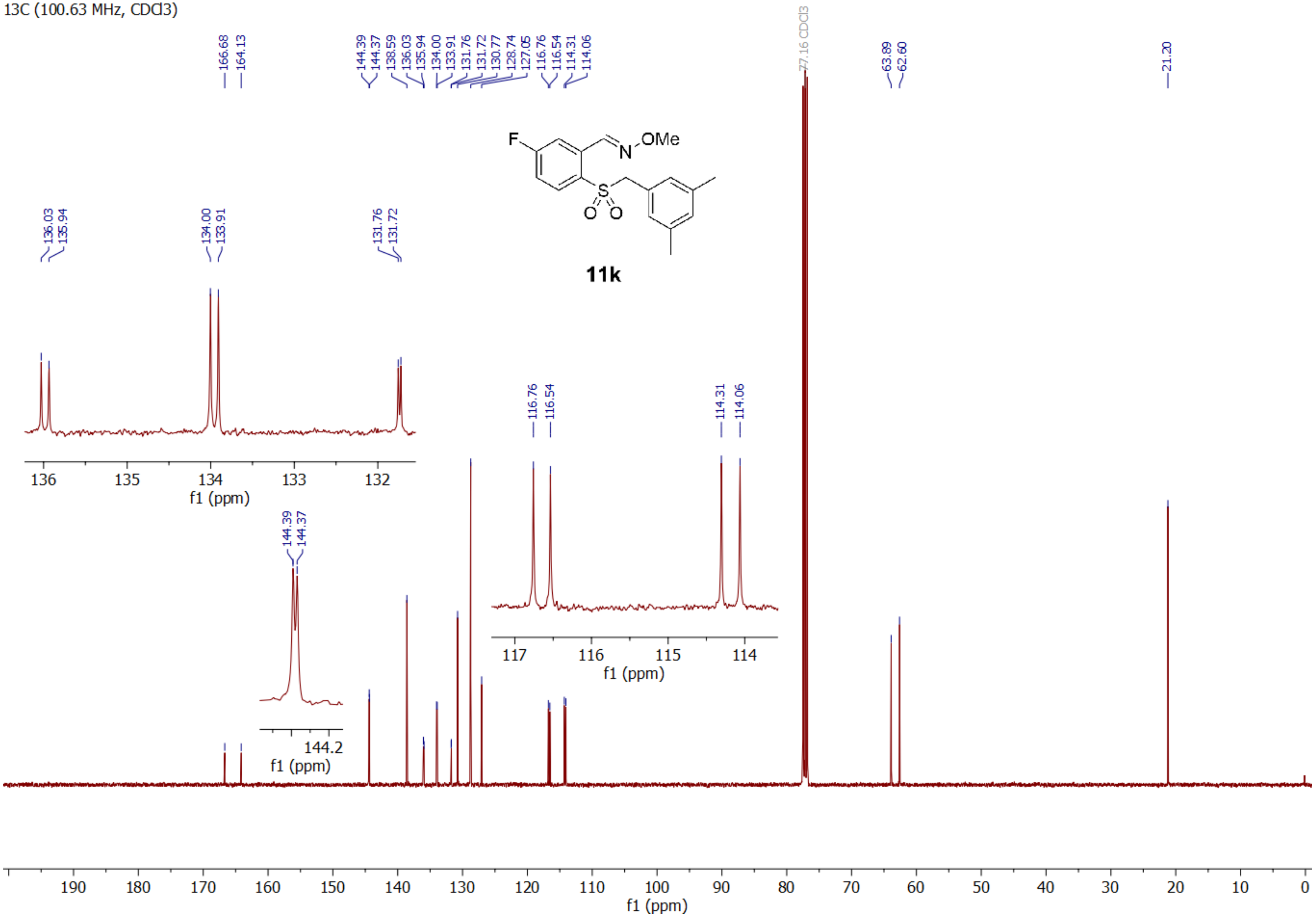
13C (100.63 MHz, CDCl3)
166.68
164.13
144.39
144.37
138.59
136.03
135.94
134.00
133.91
131.76
131.72
130.77
128.74
127.05
116.76
116.54
114.31
114.06
77.16 CDCl3
63.89
62.60
21.20
F
N
OMe
S
O
O
11k
f1 (ppm)

19F (376.48 MHz, CDCl3)

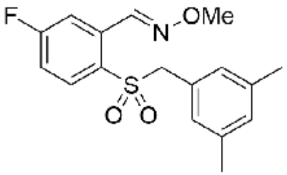

11k

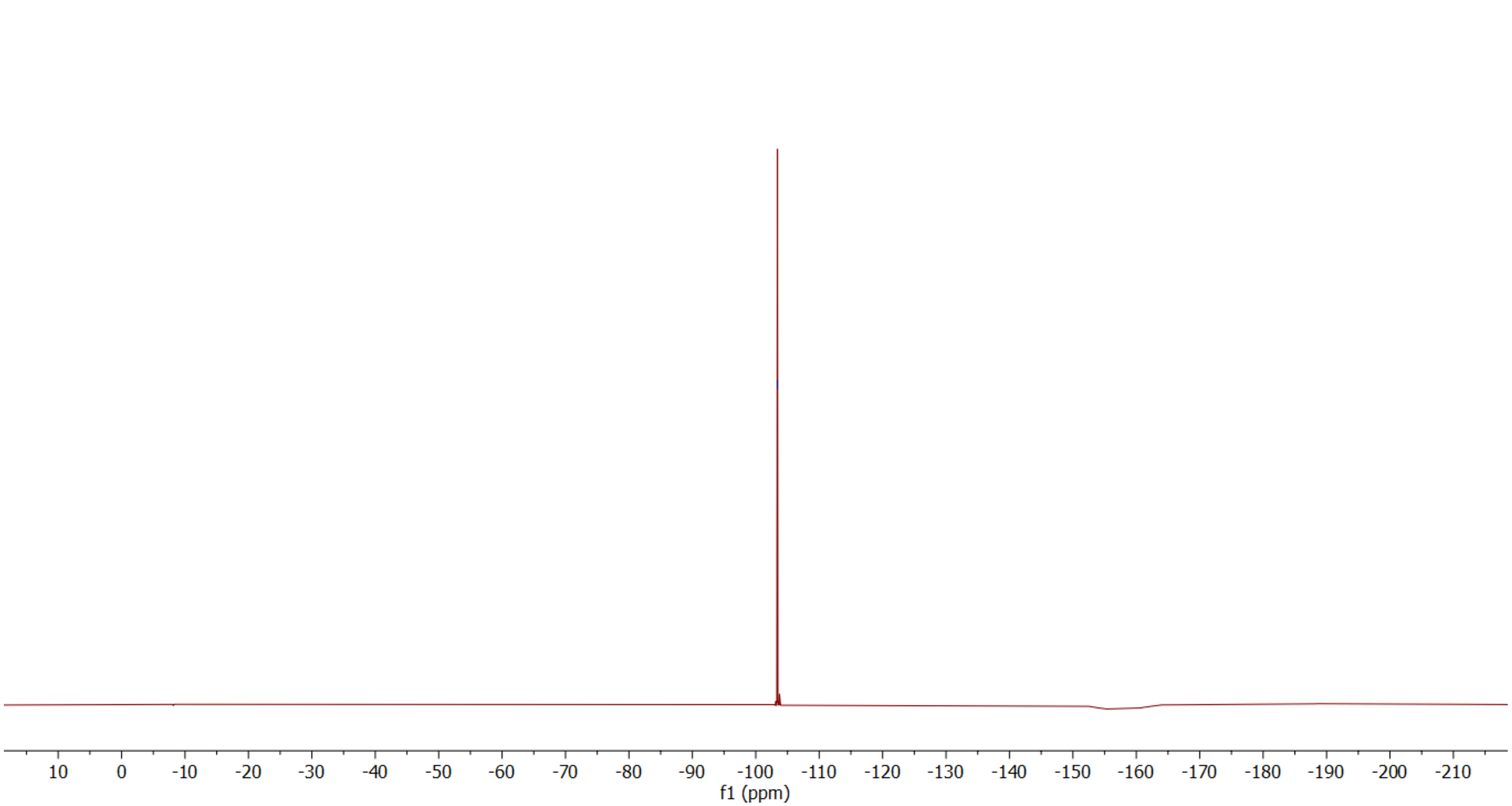

1H (400.15 MHz, DMSO)

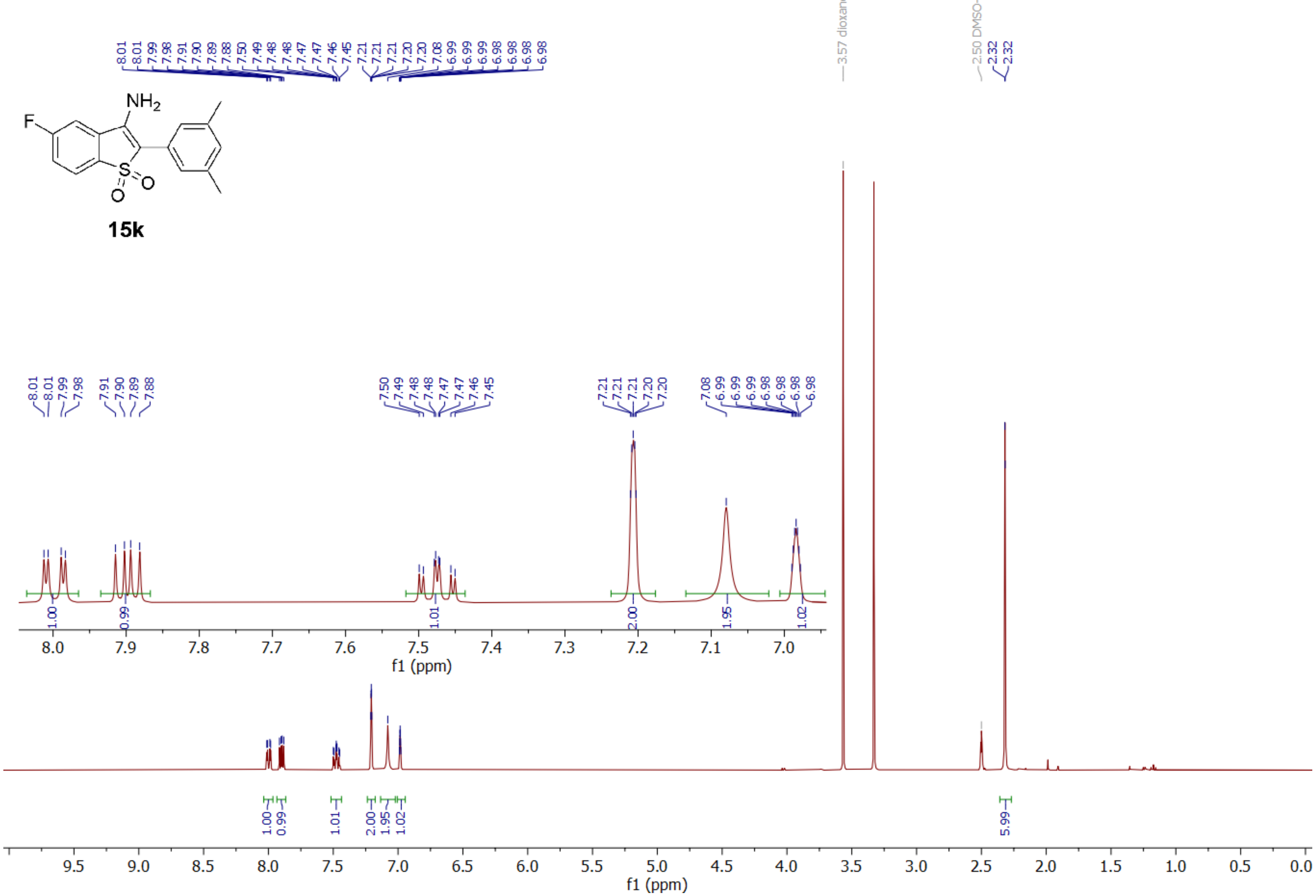

13C (100.63 MHz, DMSO)

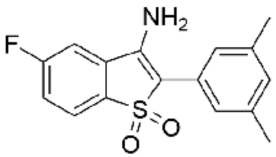

15k

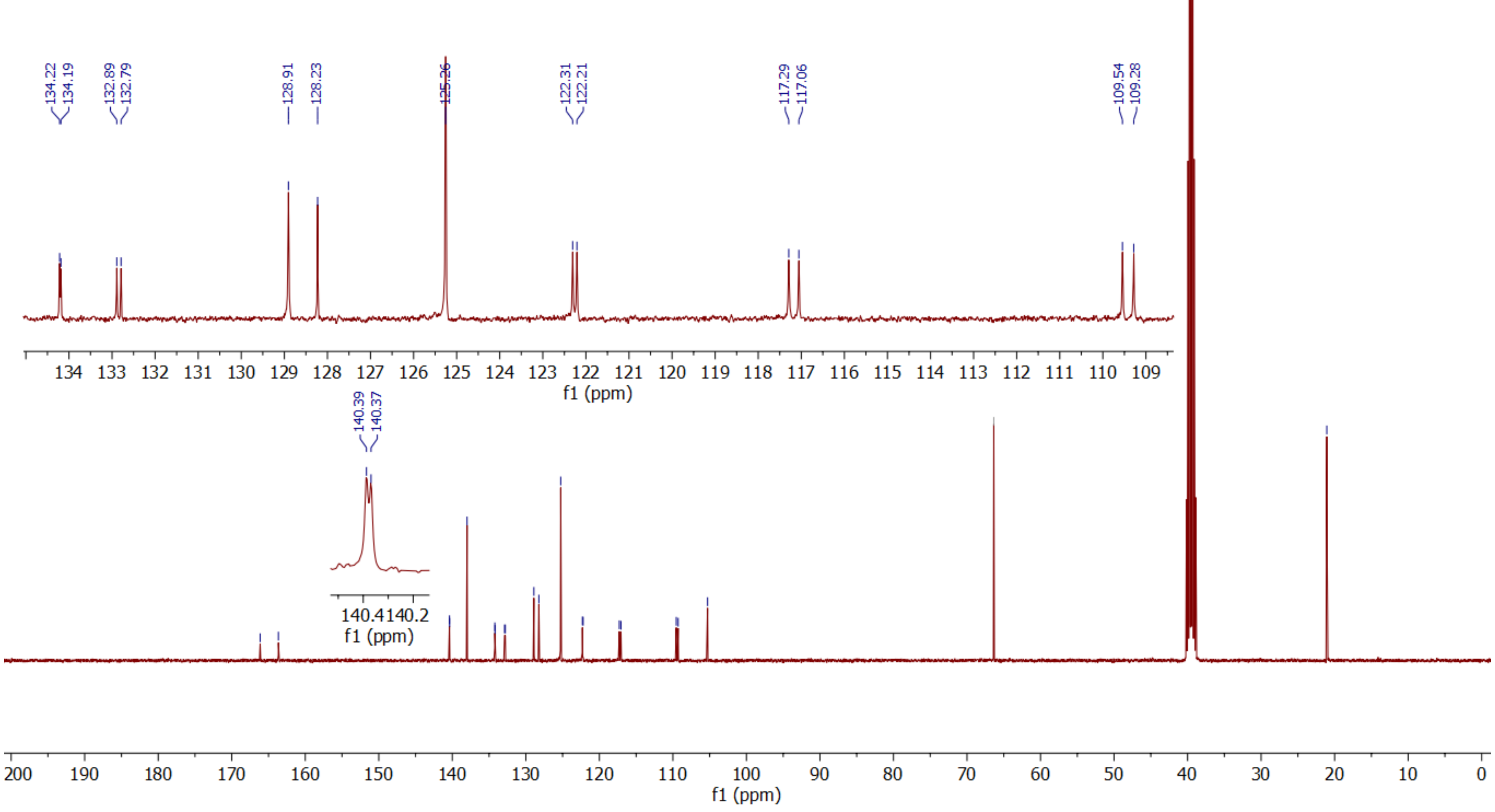

19F (376.48 MHz, DMSO)

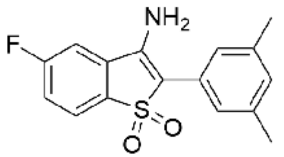

15k

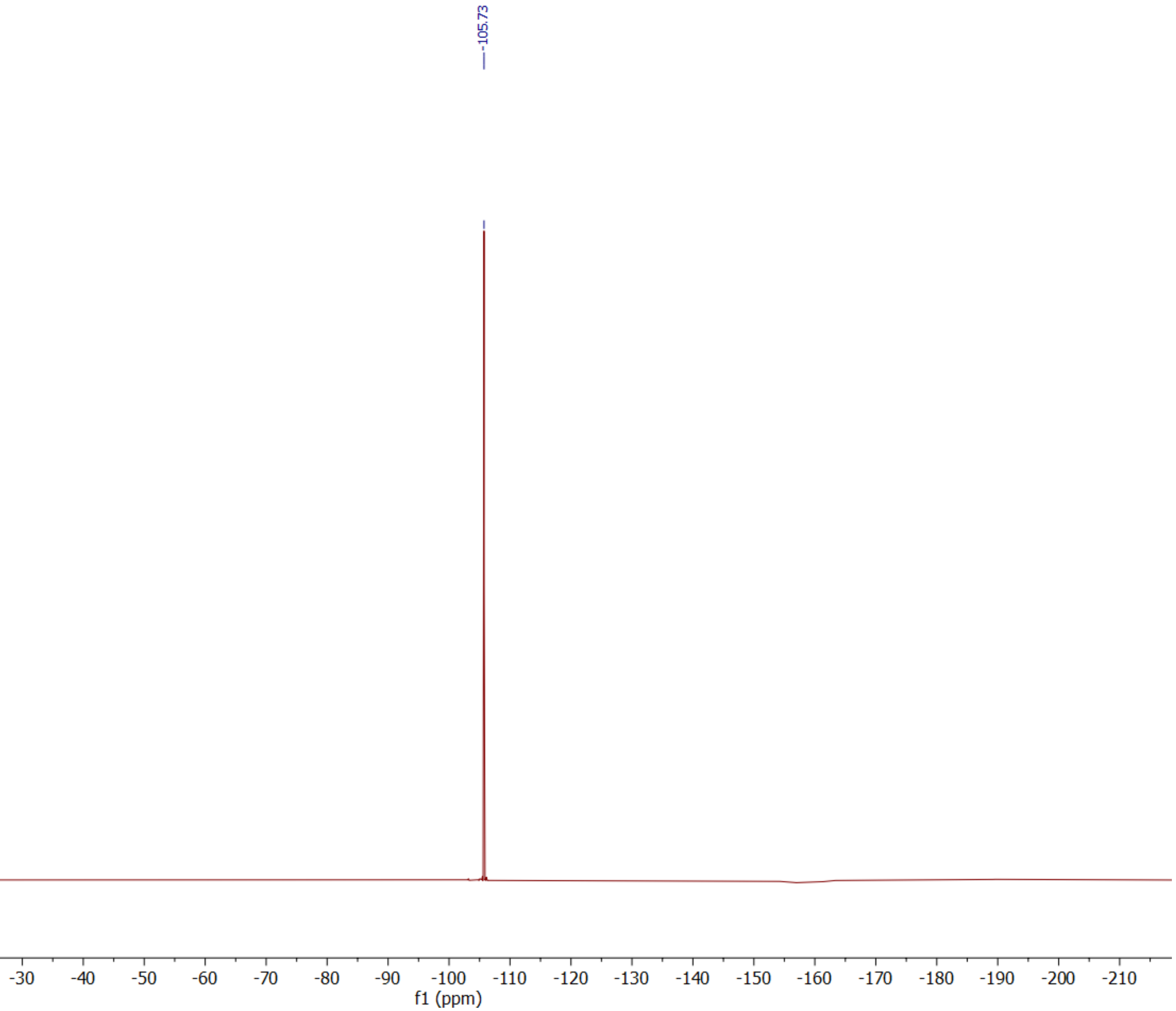

1H (400.15 MHz, CDCl3)

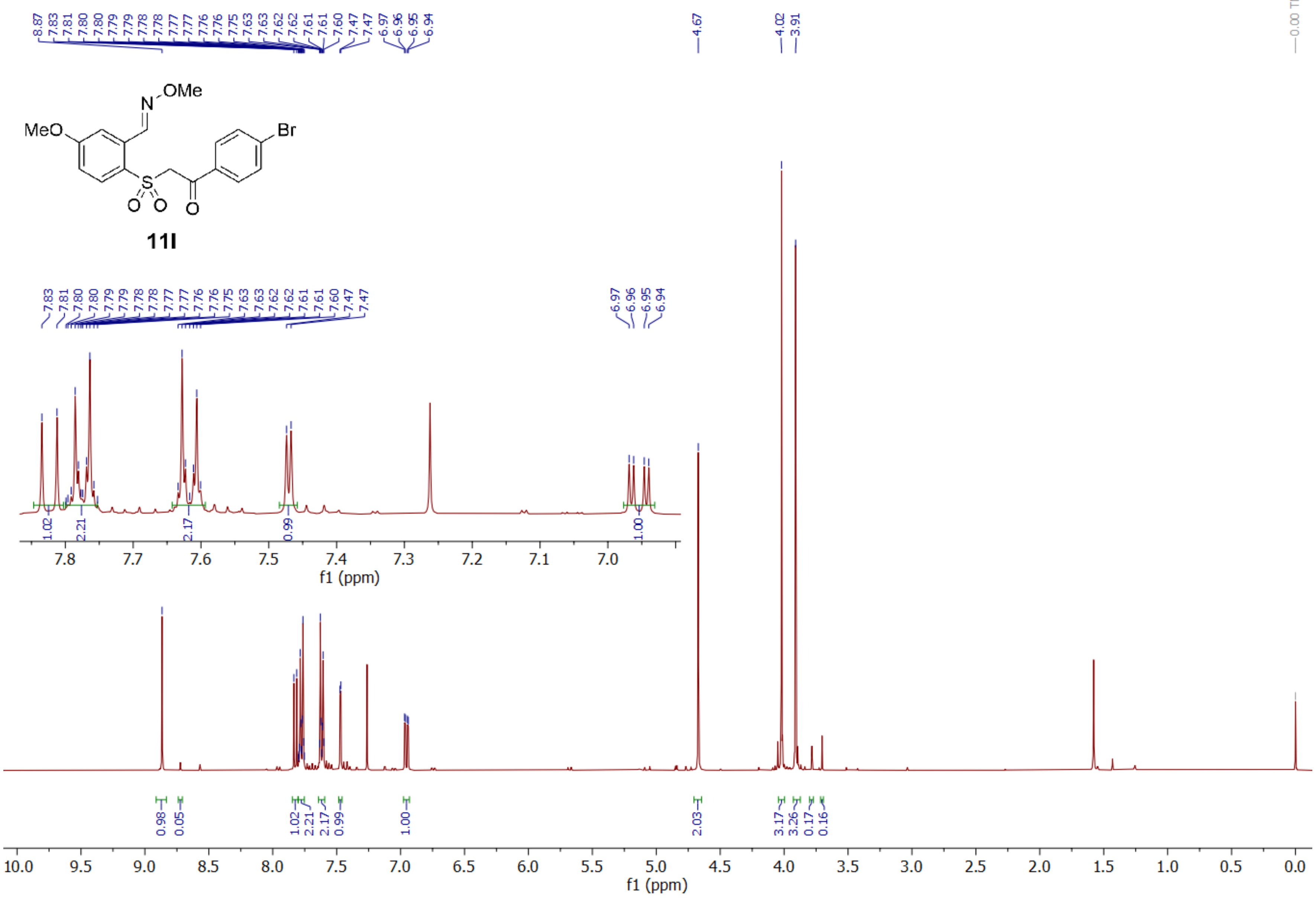

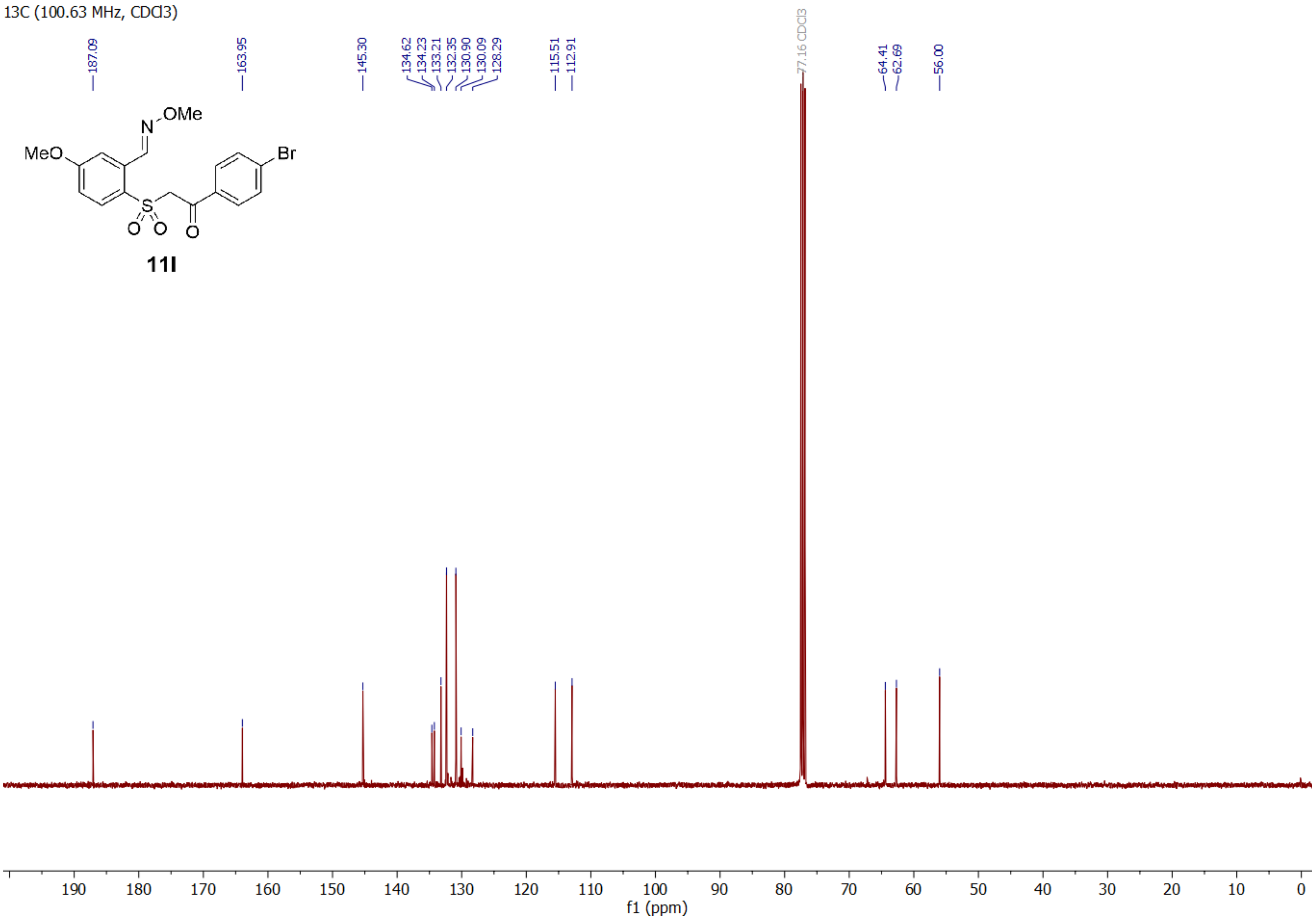
13C (100.63 MHz, CDCl3)
187.09
163.95
145.30
134.62
134.23
133.21
132.35
130.90
130.09
128.29
115.51
112.91
77.16 CDCl3
64.41
62.69
56.00
N
OMe
MeO
Br
S
O
O
O
11l
190
180
170
160
150
140
130
120
110
100
90
80
70
60
50
40
30
20
10
0
f1 (ppm)

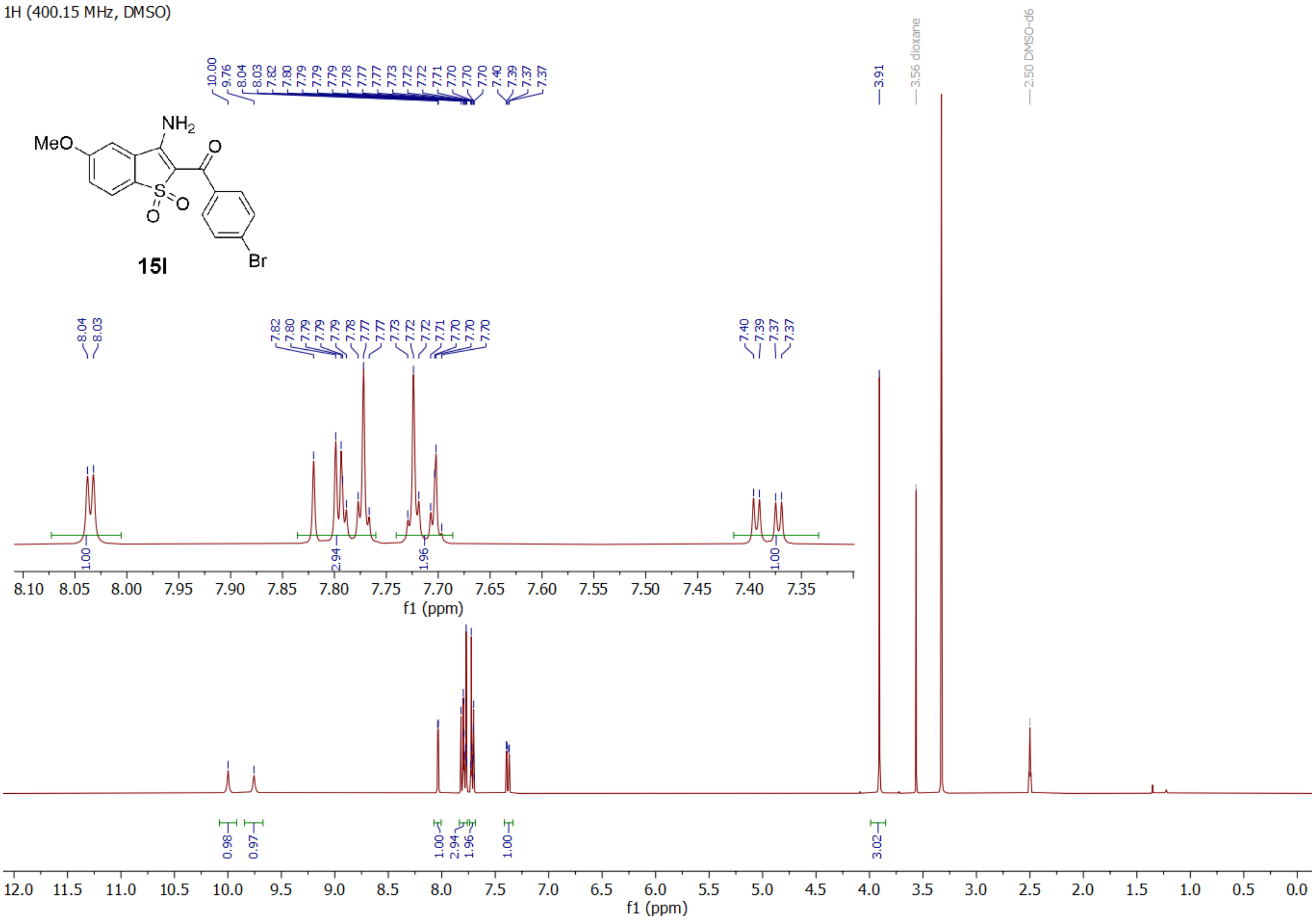
1H (400.15 MHz, DMSO)
10.00
9.76
8.04
8.03
7.82
7.80
7.79
7.79
7.79
7.78
7.77
7.77
7.73
7.72
7.72
7.71
7.70
7.70
7.70
7.40
7.39
7.37
7.37
3.91
3.56 dioxane
2.50 DMSO-d6
MeO
NH2
O
S
O
O
Br
15l
0.98
0.97
1.00
2.94
1.96
1.00
3.02
1.00
f1 (ppm)

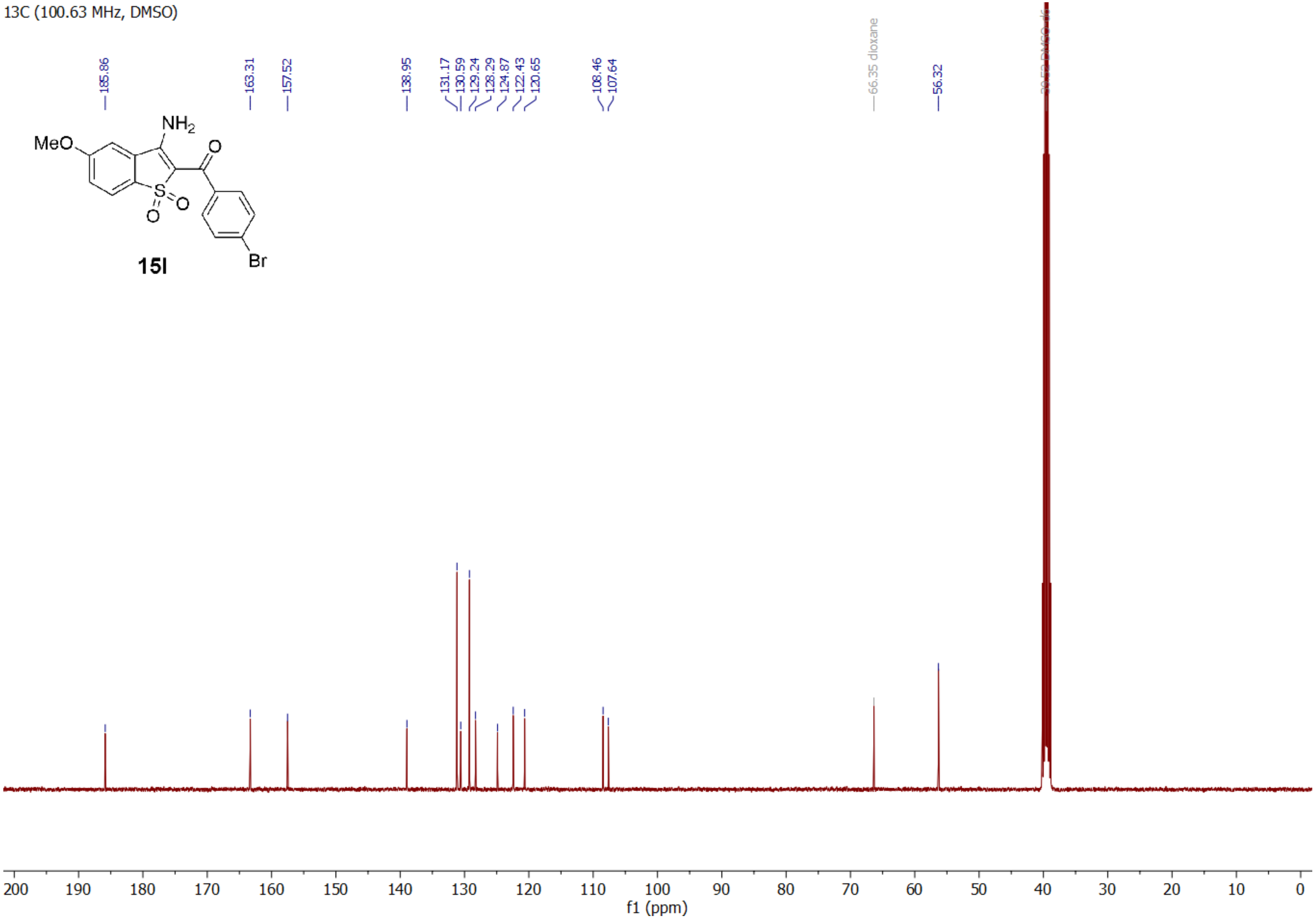

13C (100.63 MHz, DMSO)
185.86
163.31
157.52
138.95
131.17
130.59
129.24
128.29
124.87
122.43
120.65
108.46
107.64
66.35 dioxane
56.32
MeO
NH2
O
S
O
O
Br
15l
200
190
180
170
160
150
140
130
120
110
100
90
80
70
60
50
40
30
20
10
0
f1 (ppm)

1H (400.15 MHz, DMSO)

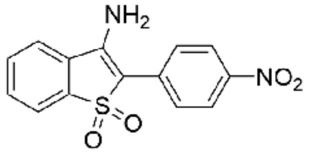

15m

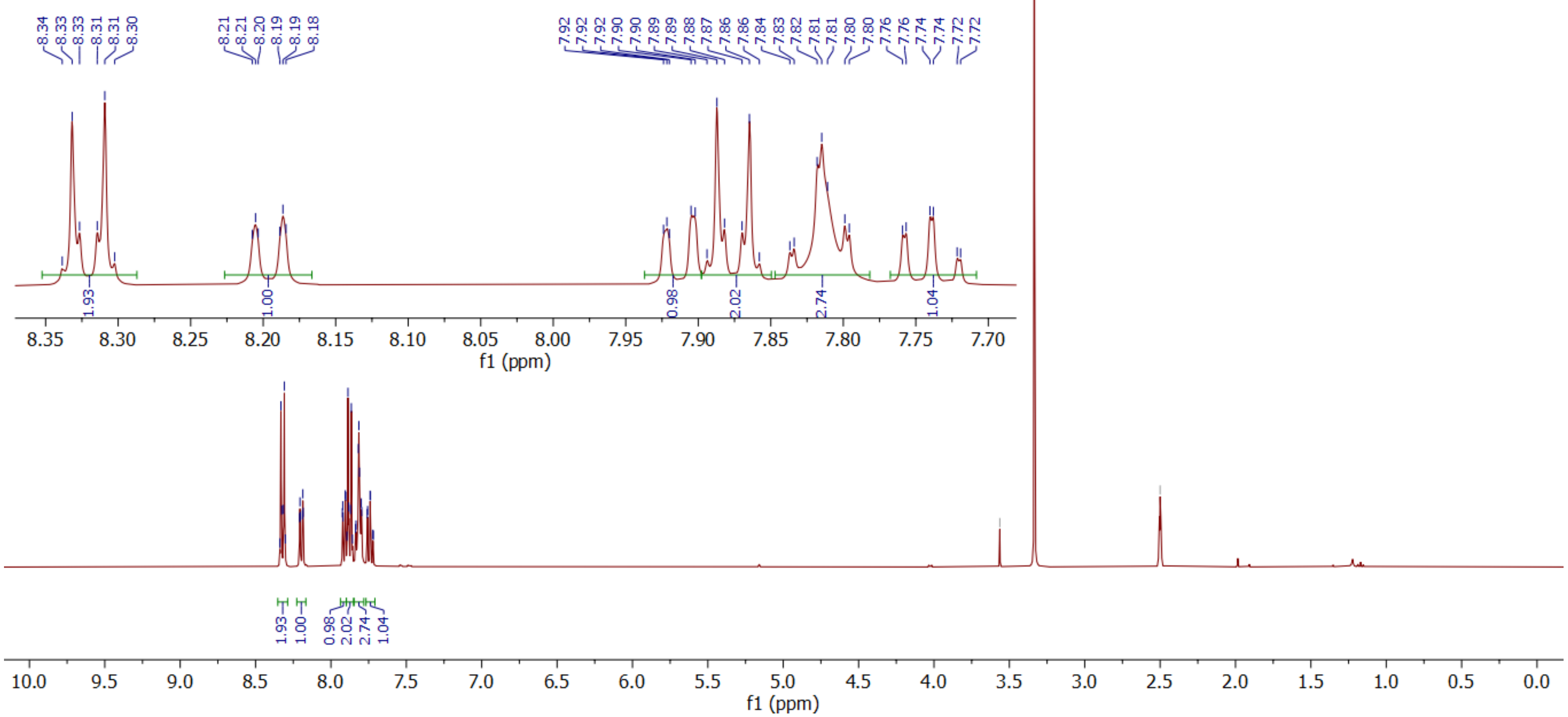

13C (100.63 MHz, DMSO)

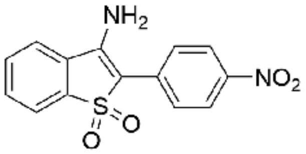

15m

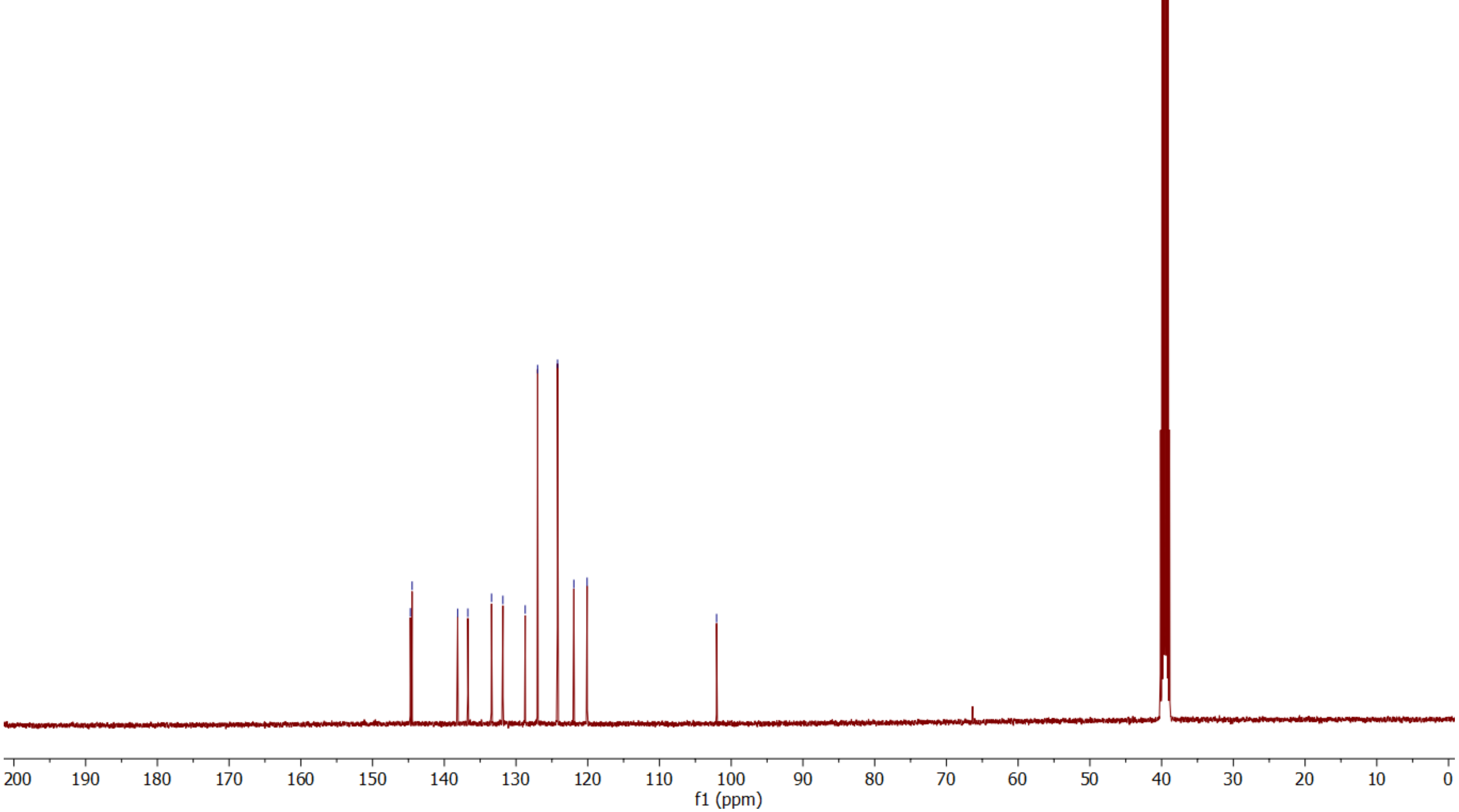

1H (400.15 MHz, DMSO)

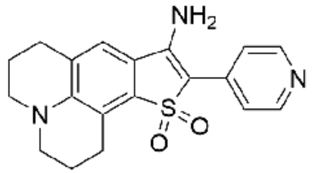

15n

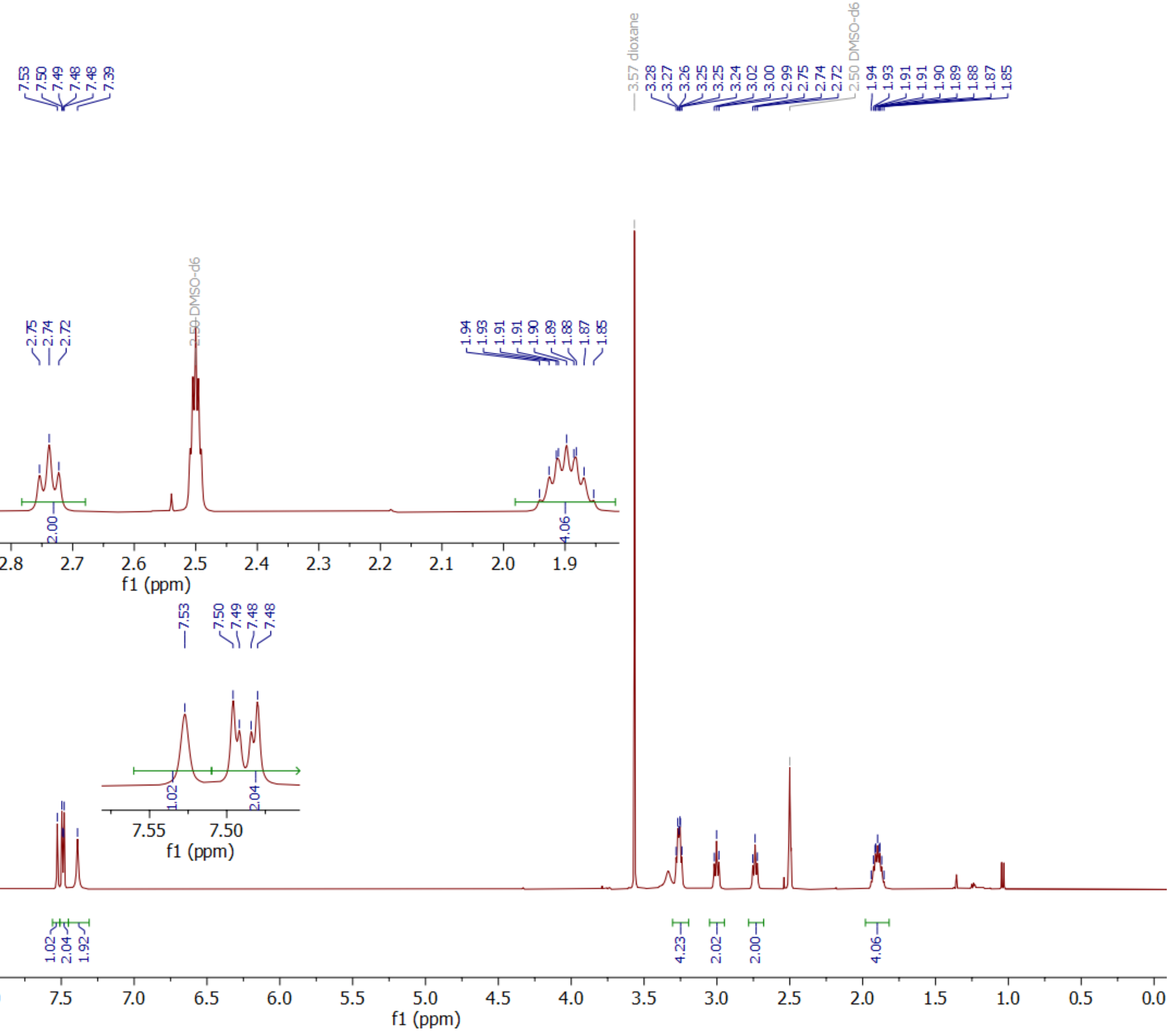

13C (100.63 MHz, DMSO)

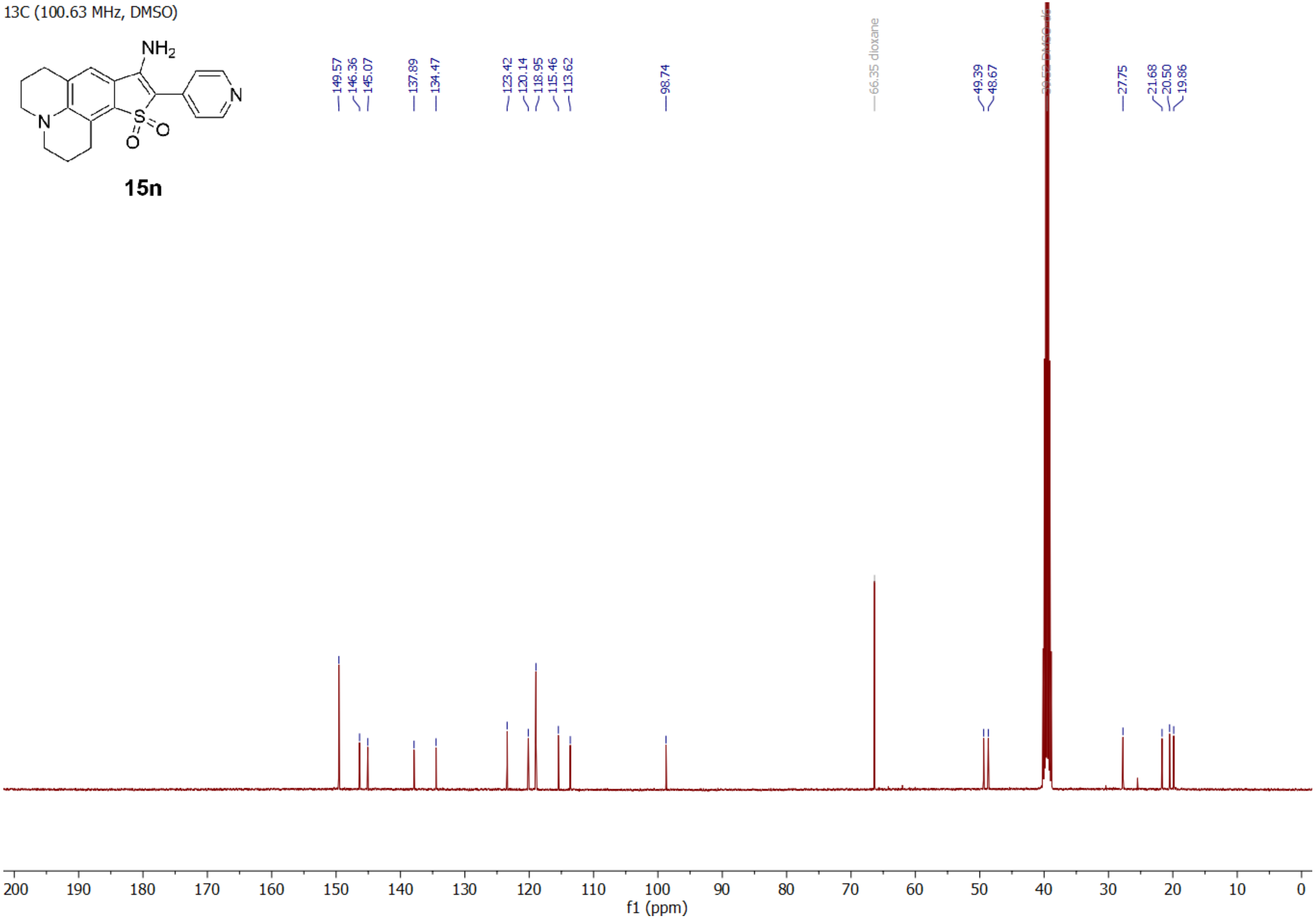

15n

1H (400.15 MHz, DMSO)

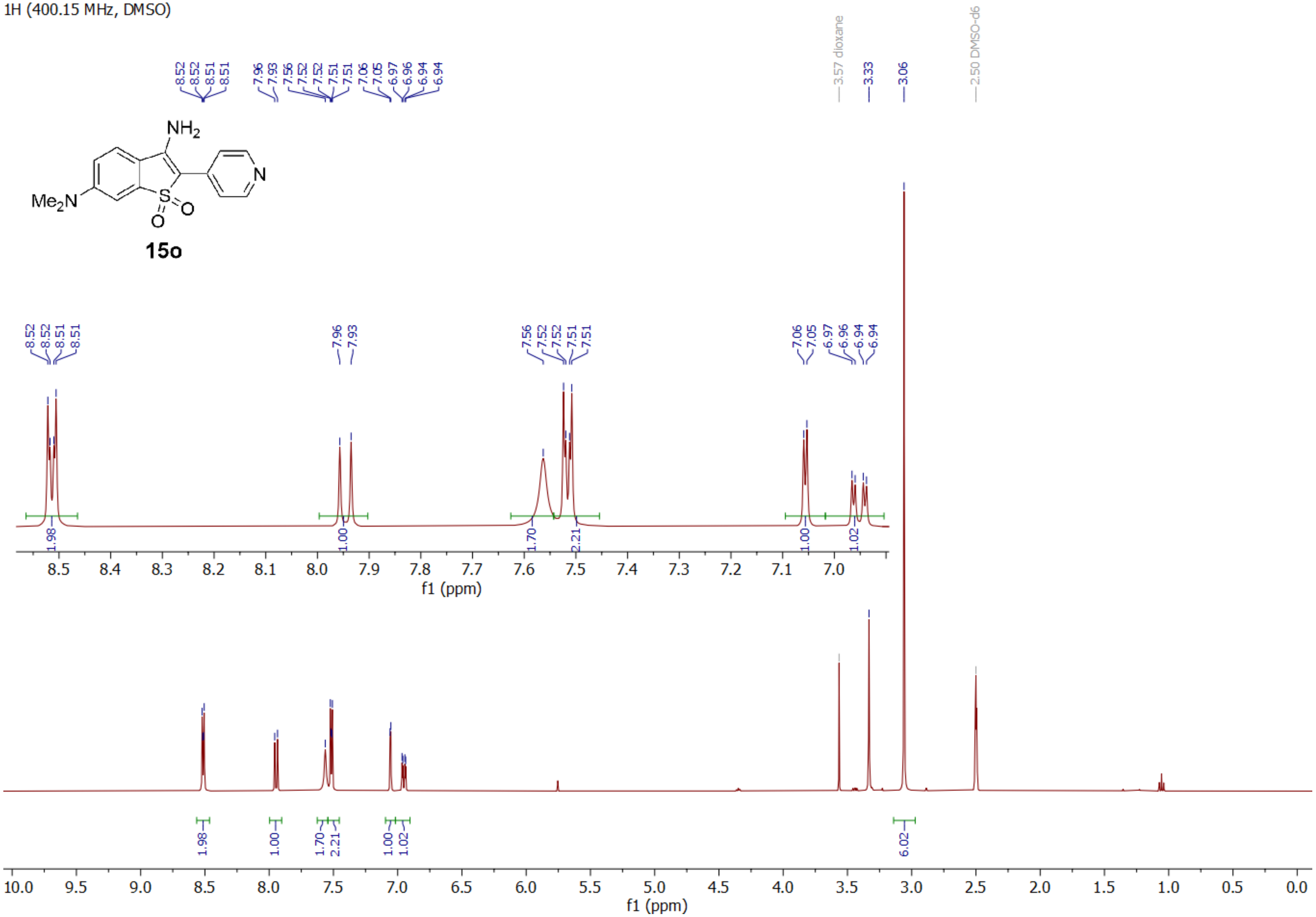

15o

13C (100.63 MHz, DMSO)

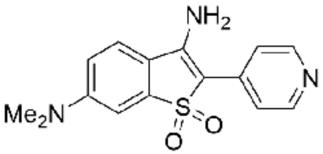

15o

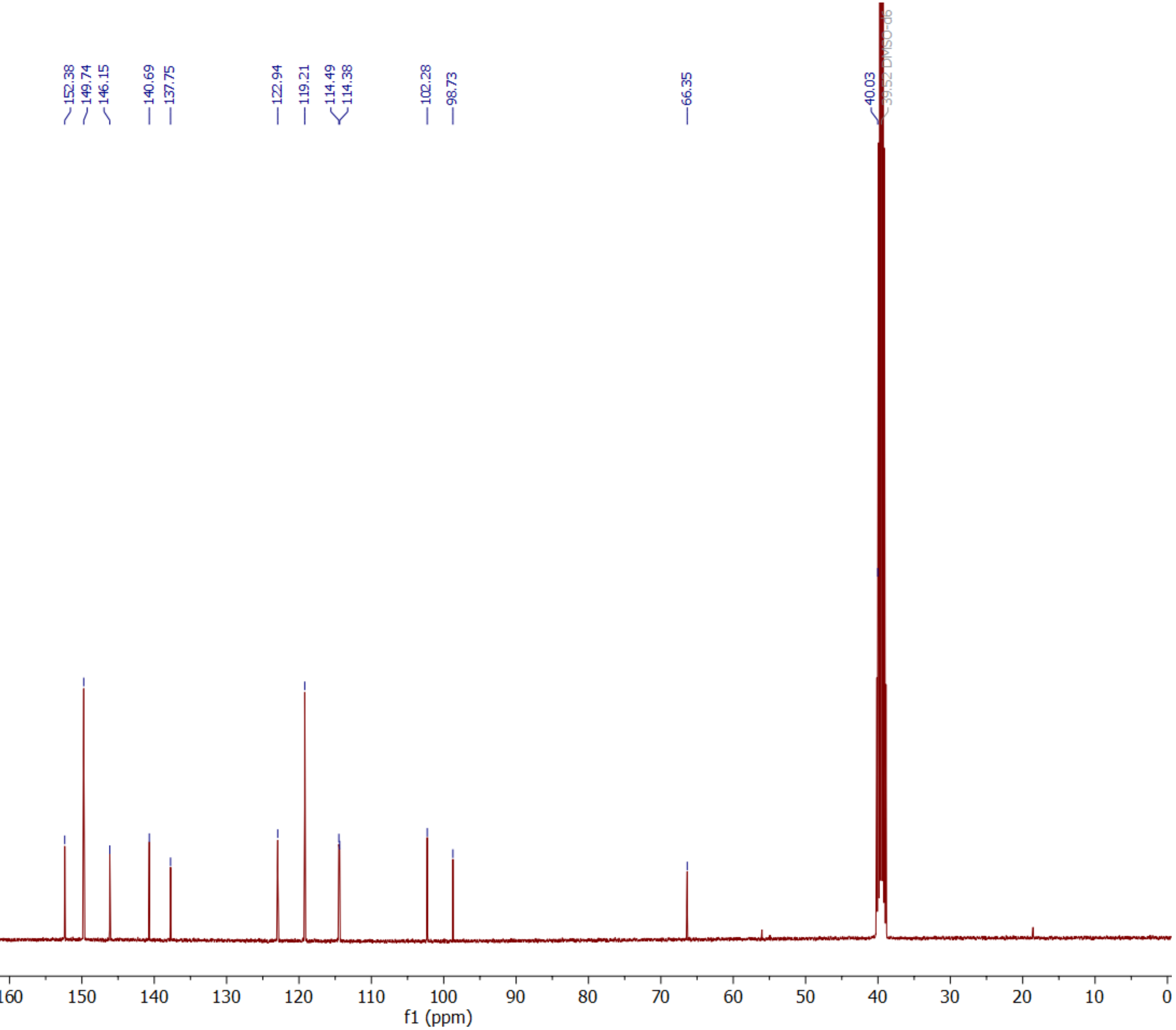

1H (400.15 MHz, DMSO)

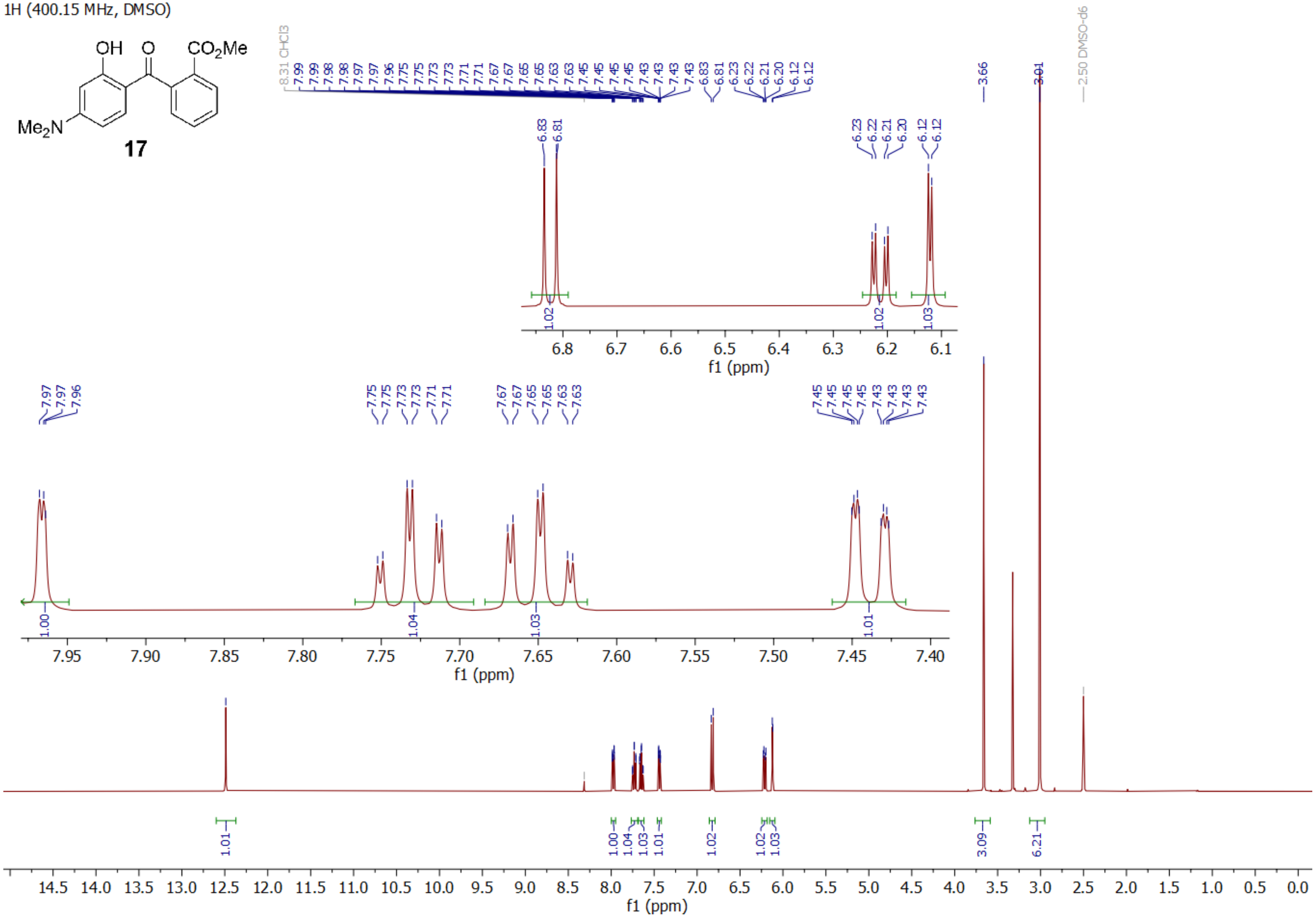

17

13C (100.63 MHz, DMSO)

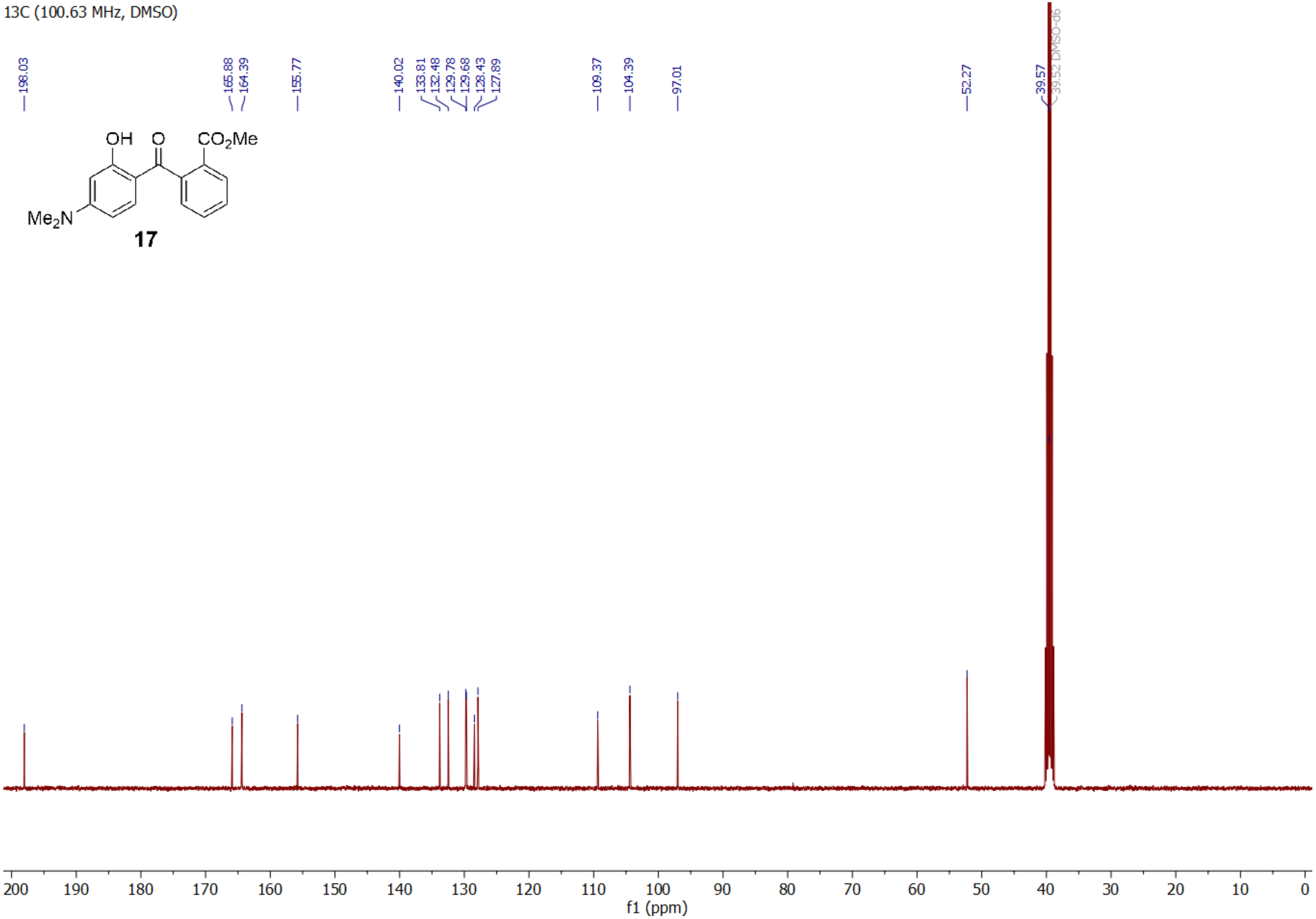

17

1H (400.15 MHz, CDCl3)

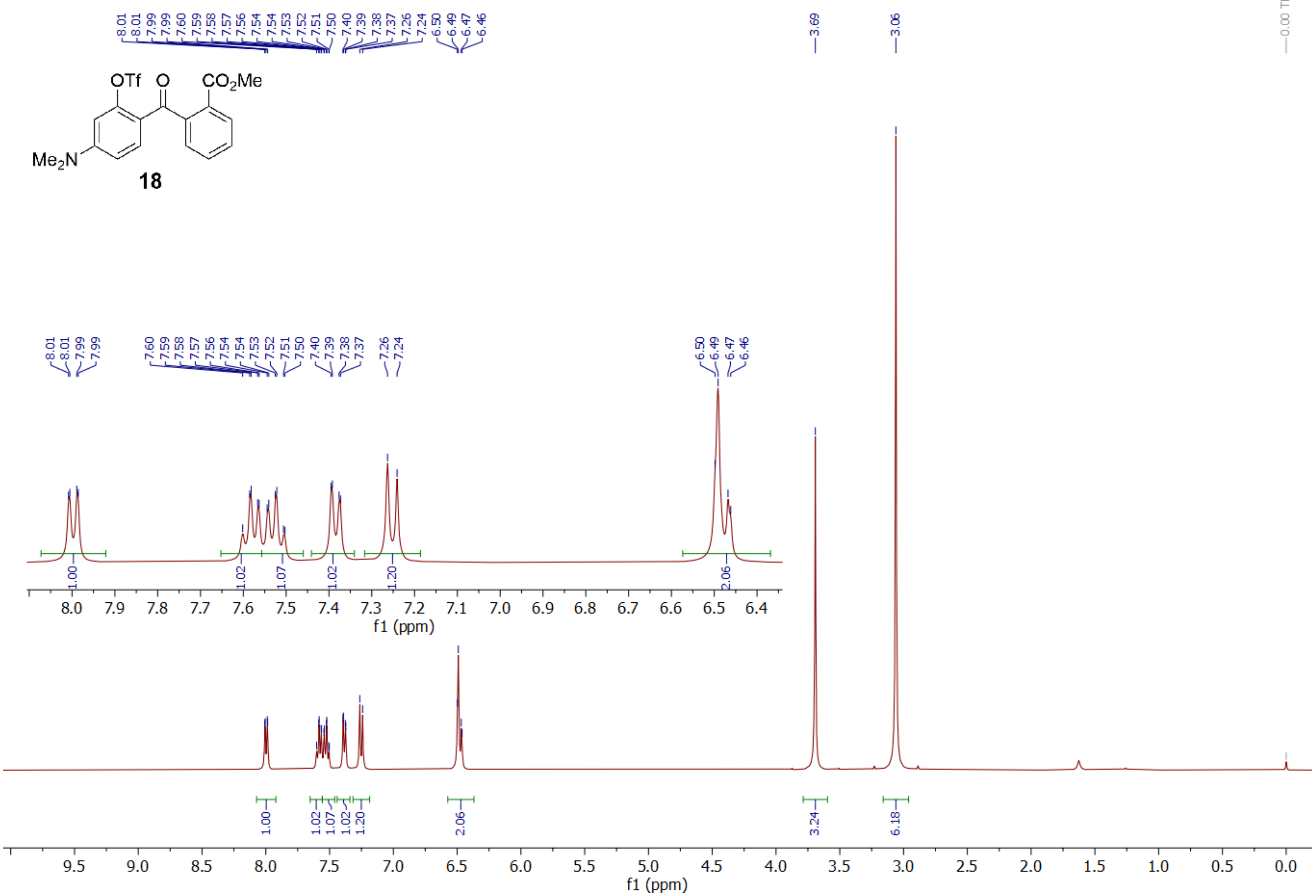

13C (100.63 MHz, CDCl3)

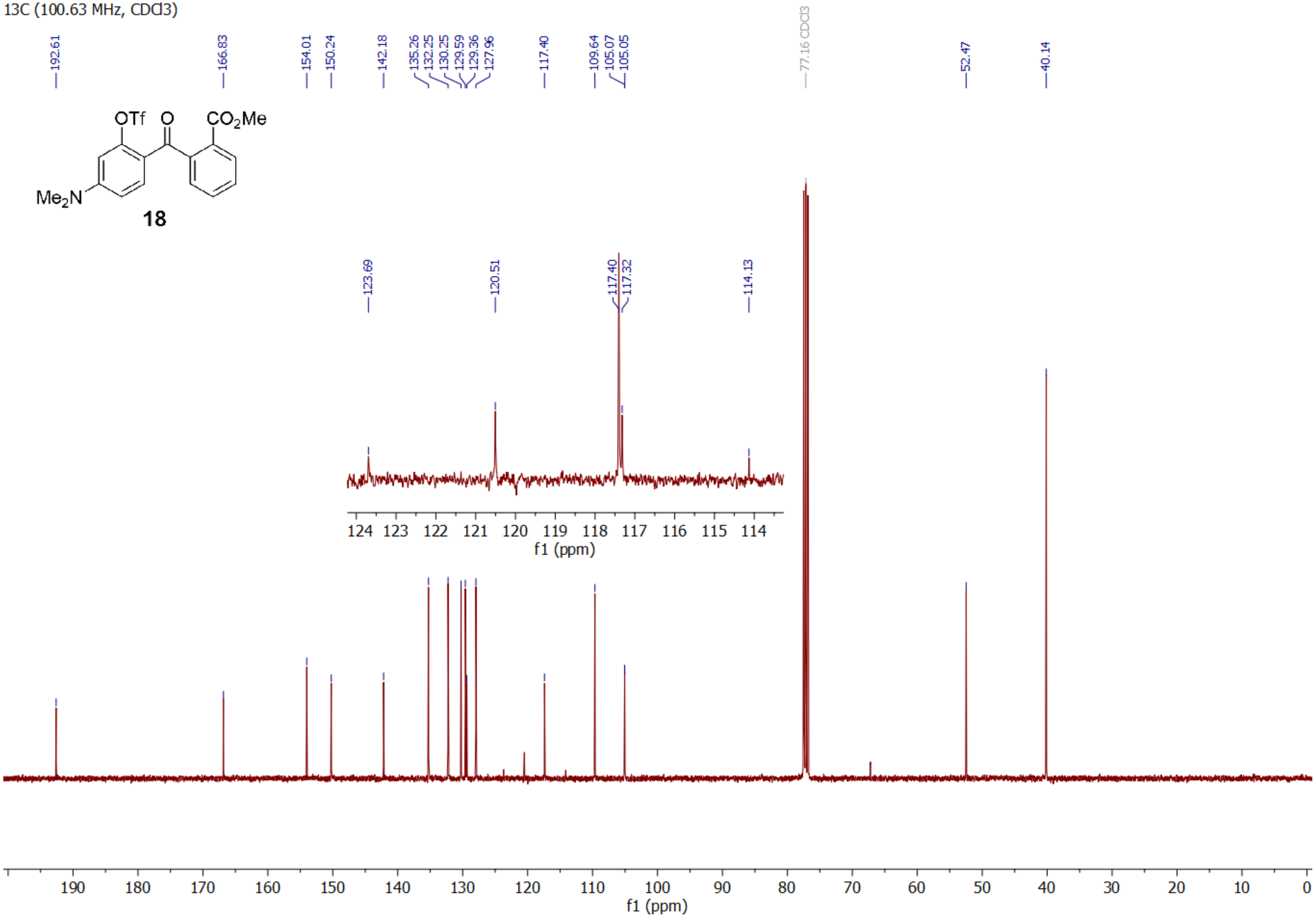

19F (376.48 MHz, CDCl3)

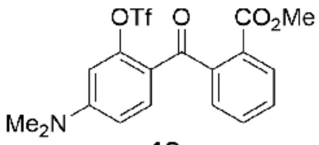

18

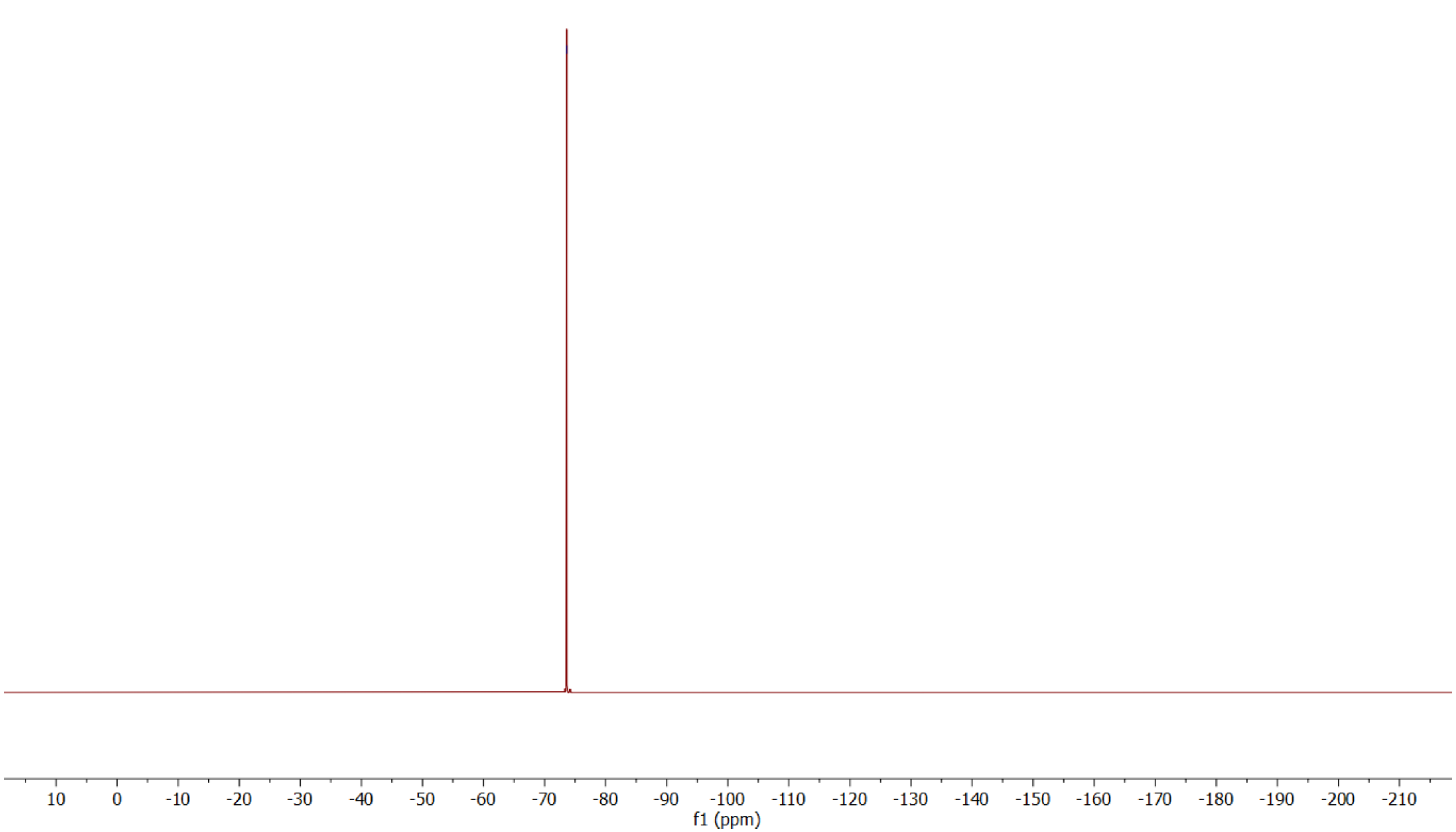

1H (400.15 MHz, CDCl3)

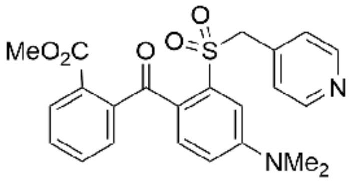

19

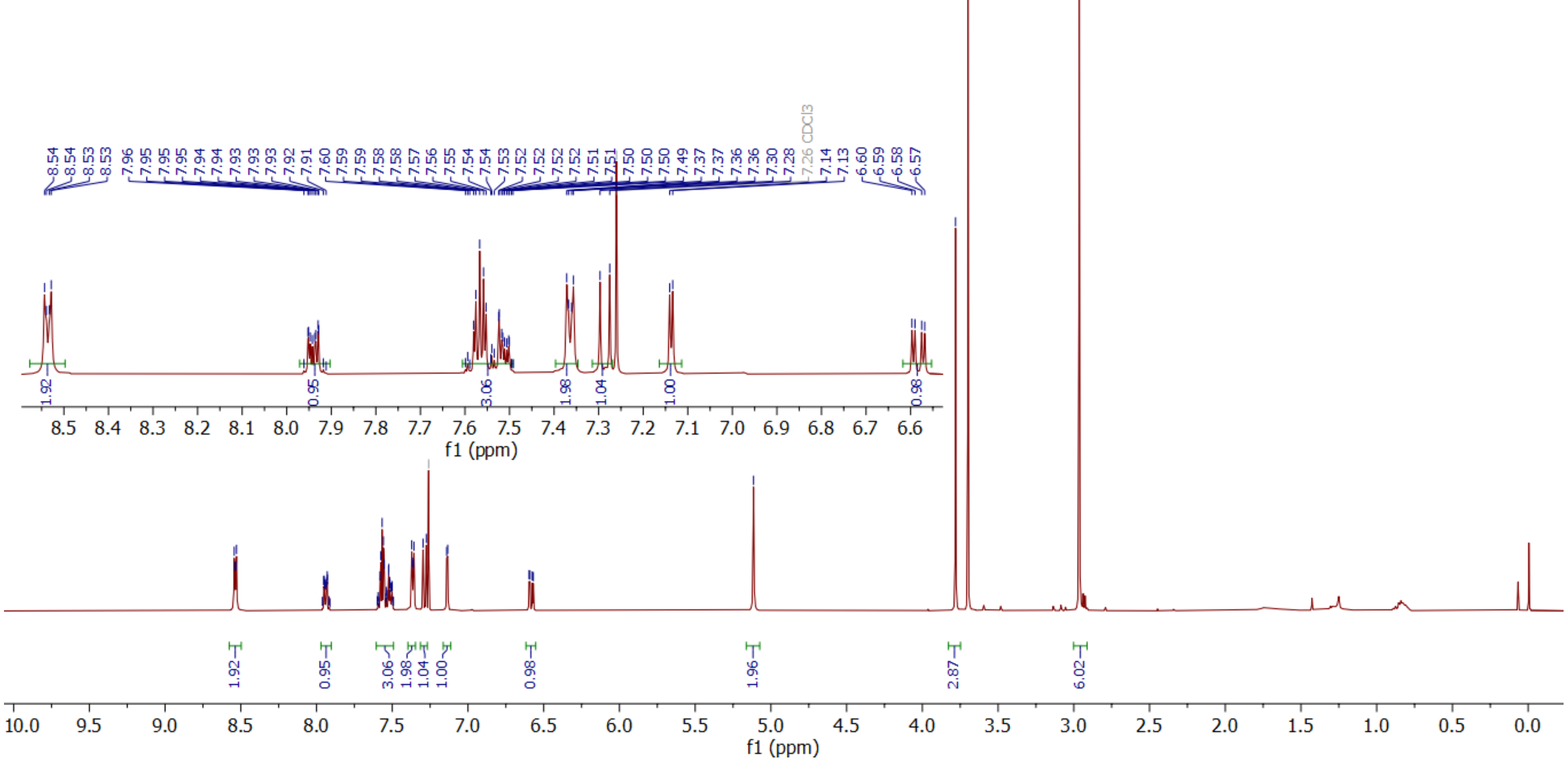

13C (100.63 MHz, CDCl3)

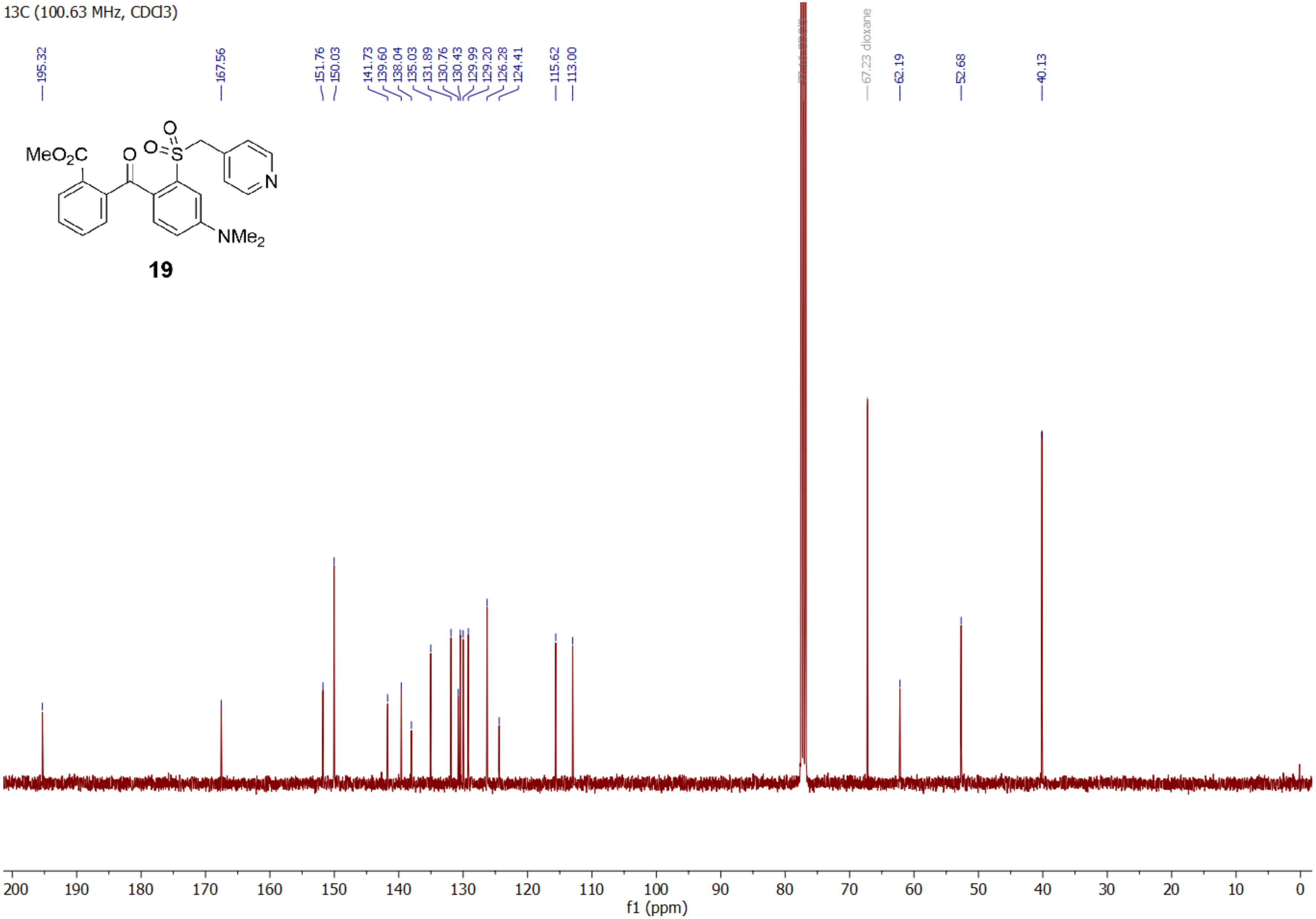

1H (400.15 MHz, DMSO)

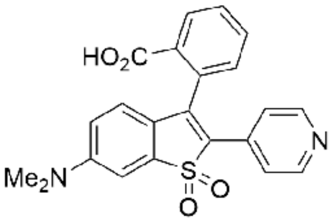

20

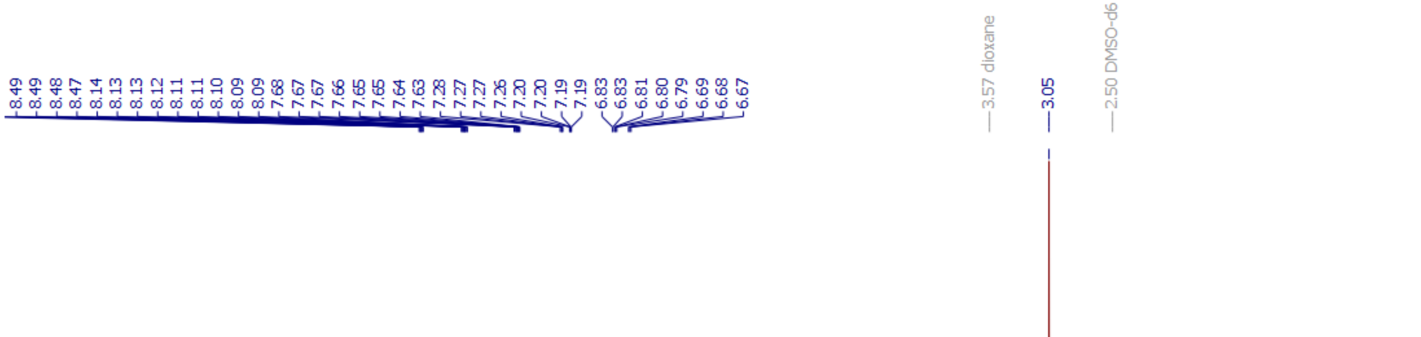

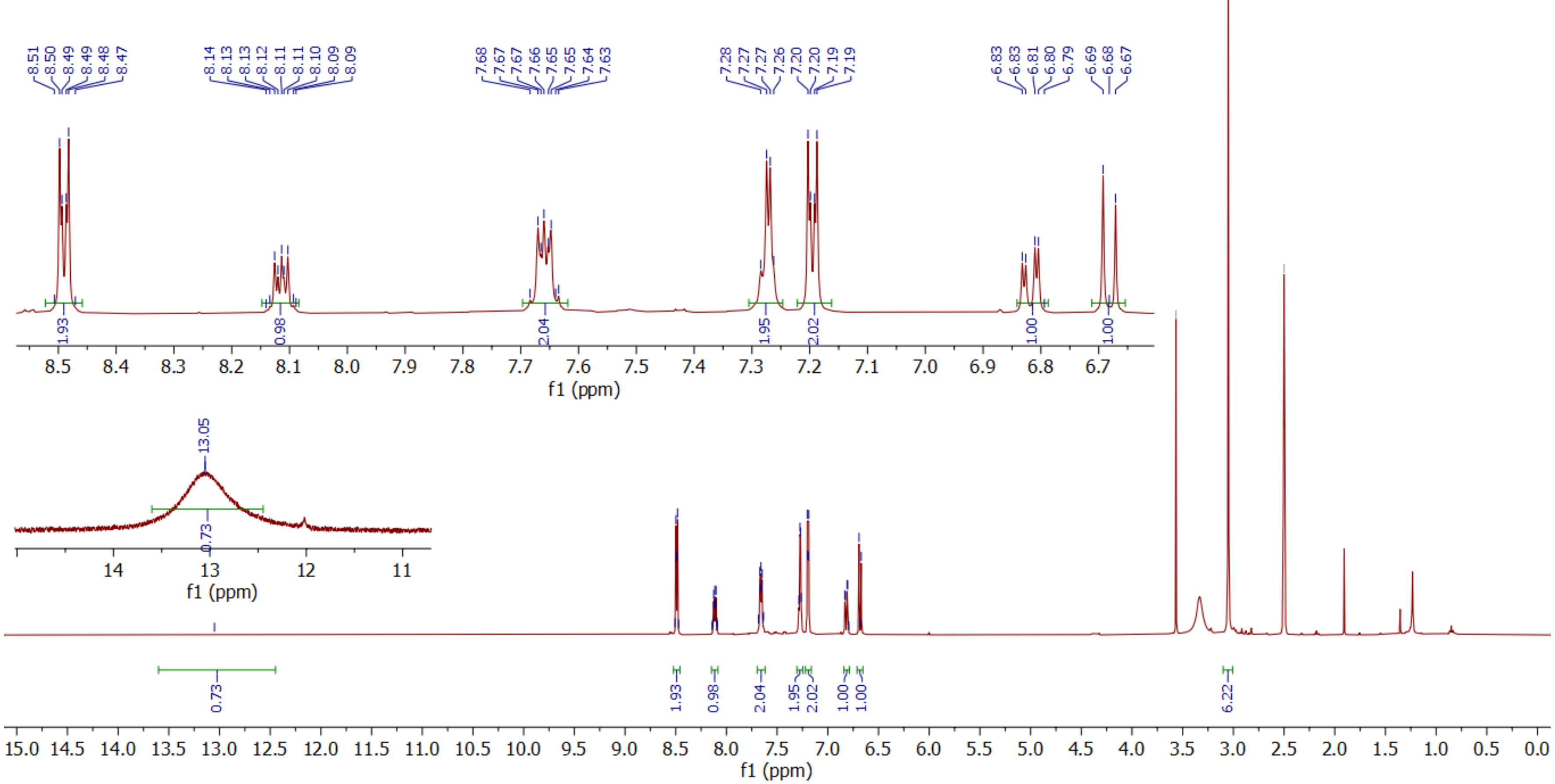

13C (100.63 MHz, DMSO)

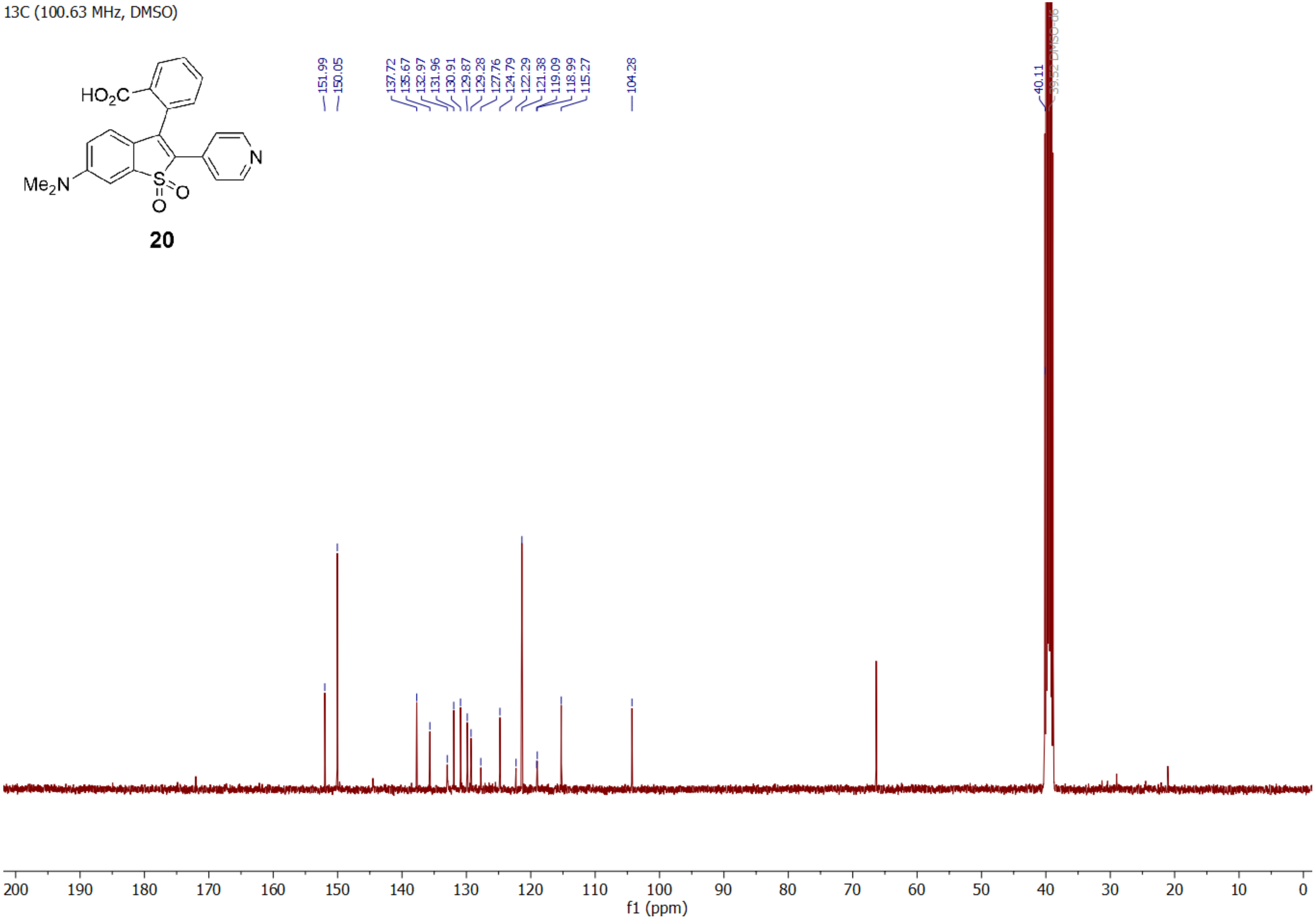

1H (400.15 MHz, CDCl3)

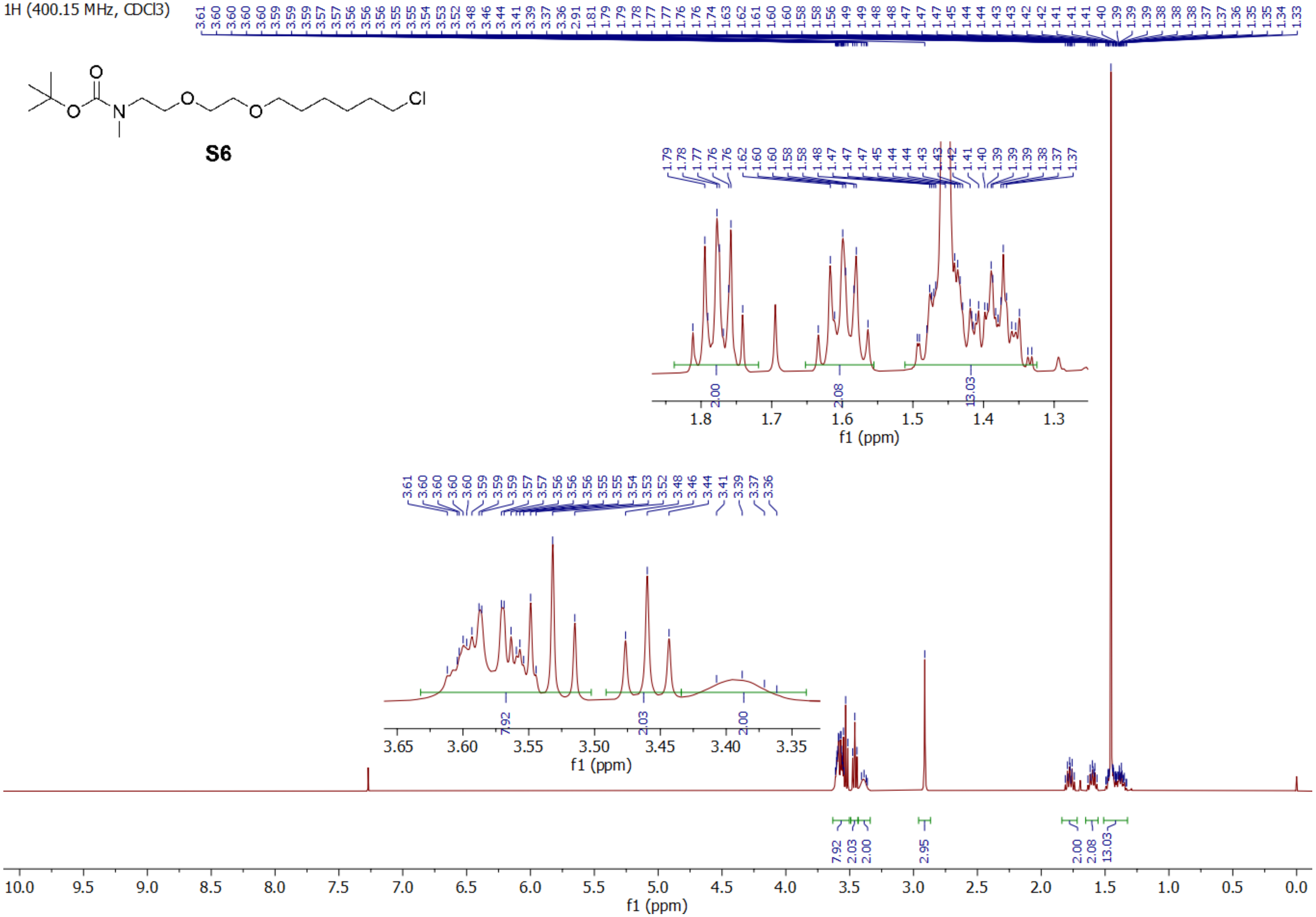

13C (100.63 MHz, CDCl3)

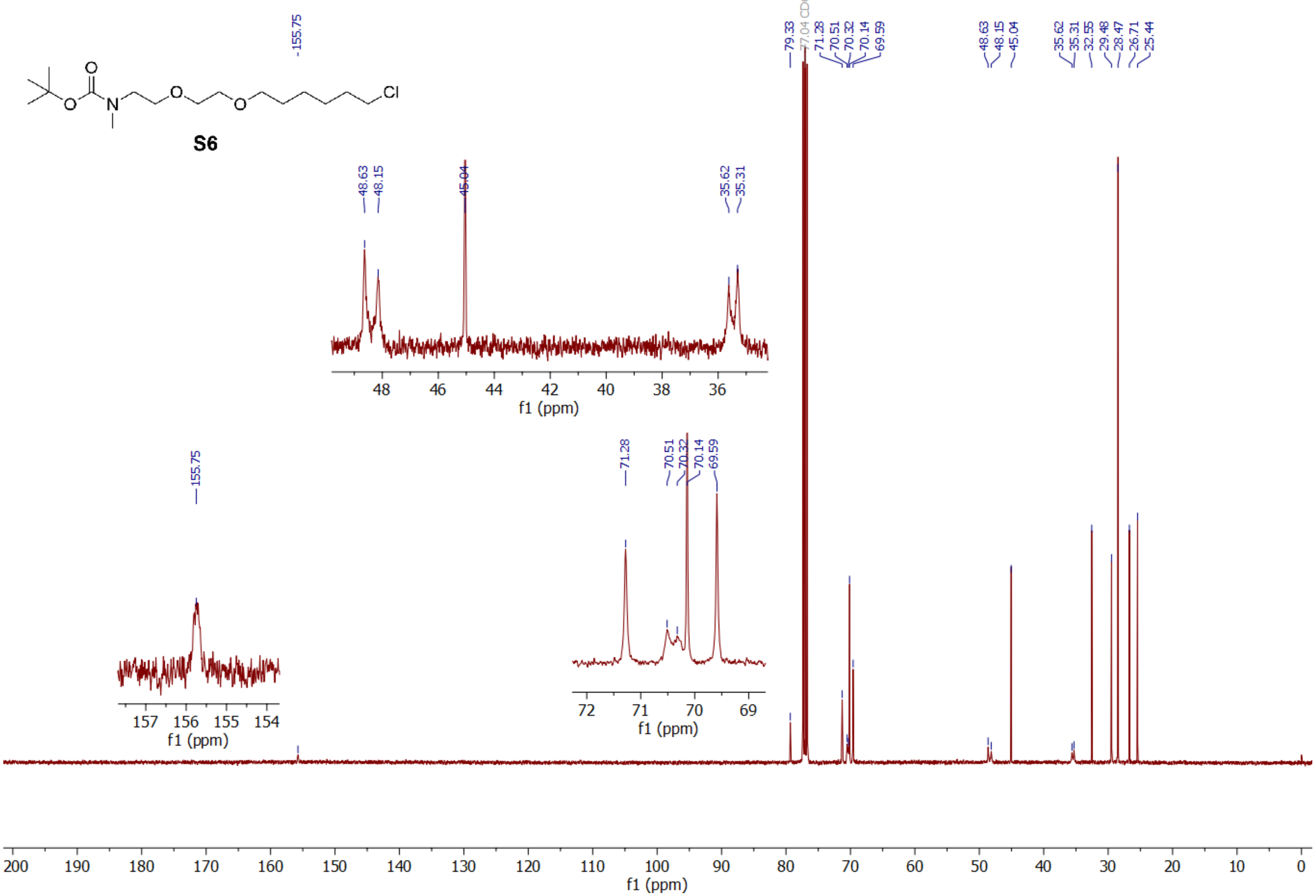

S6

1H (400.15 MHz, CDCl3)

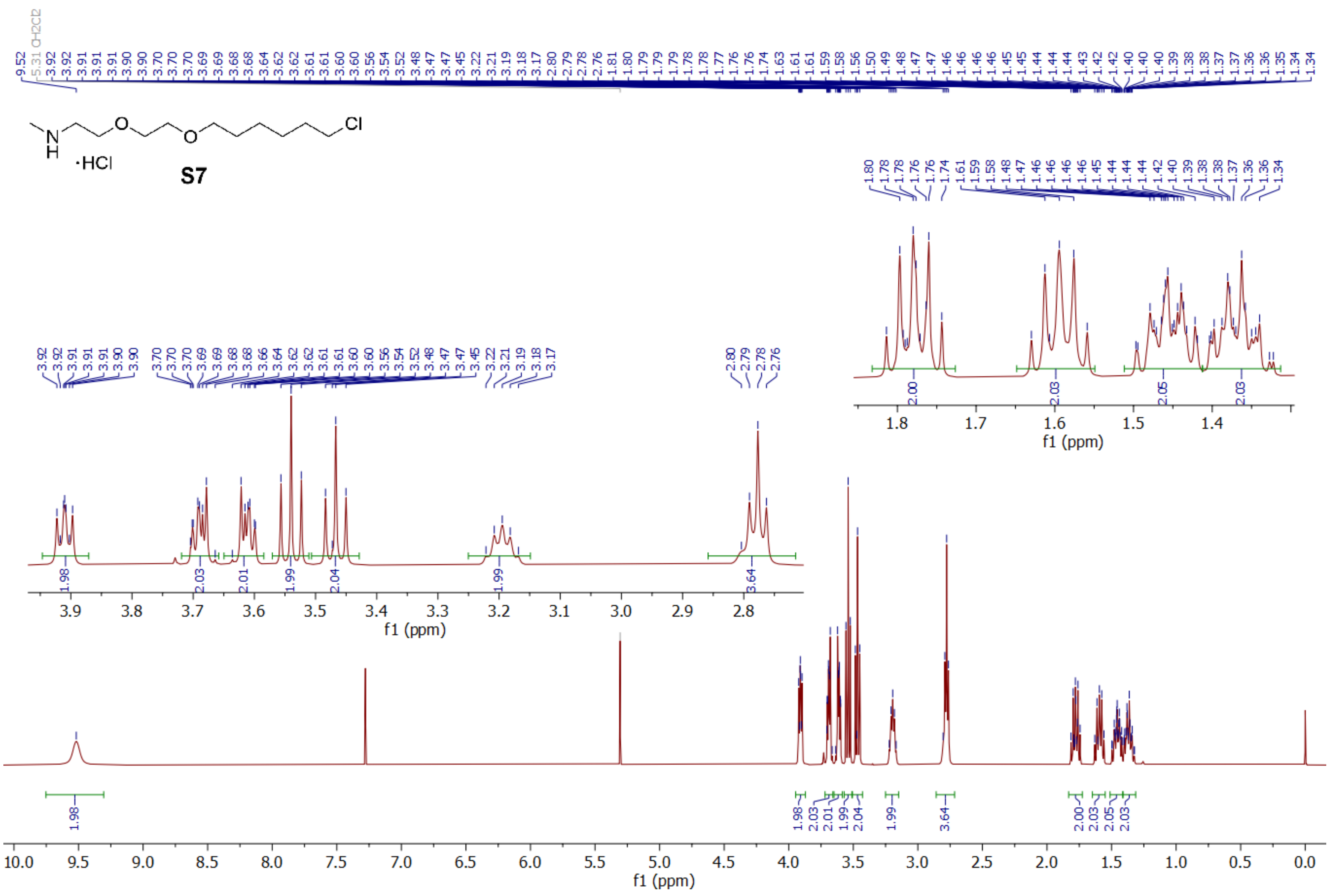

13C (100.63 MHz, CDCl3)

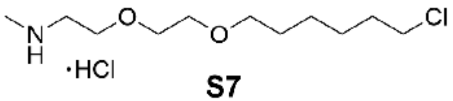

S7

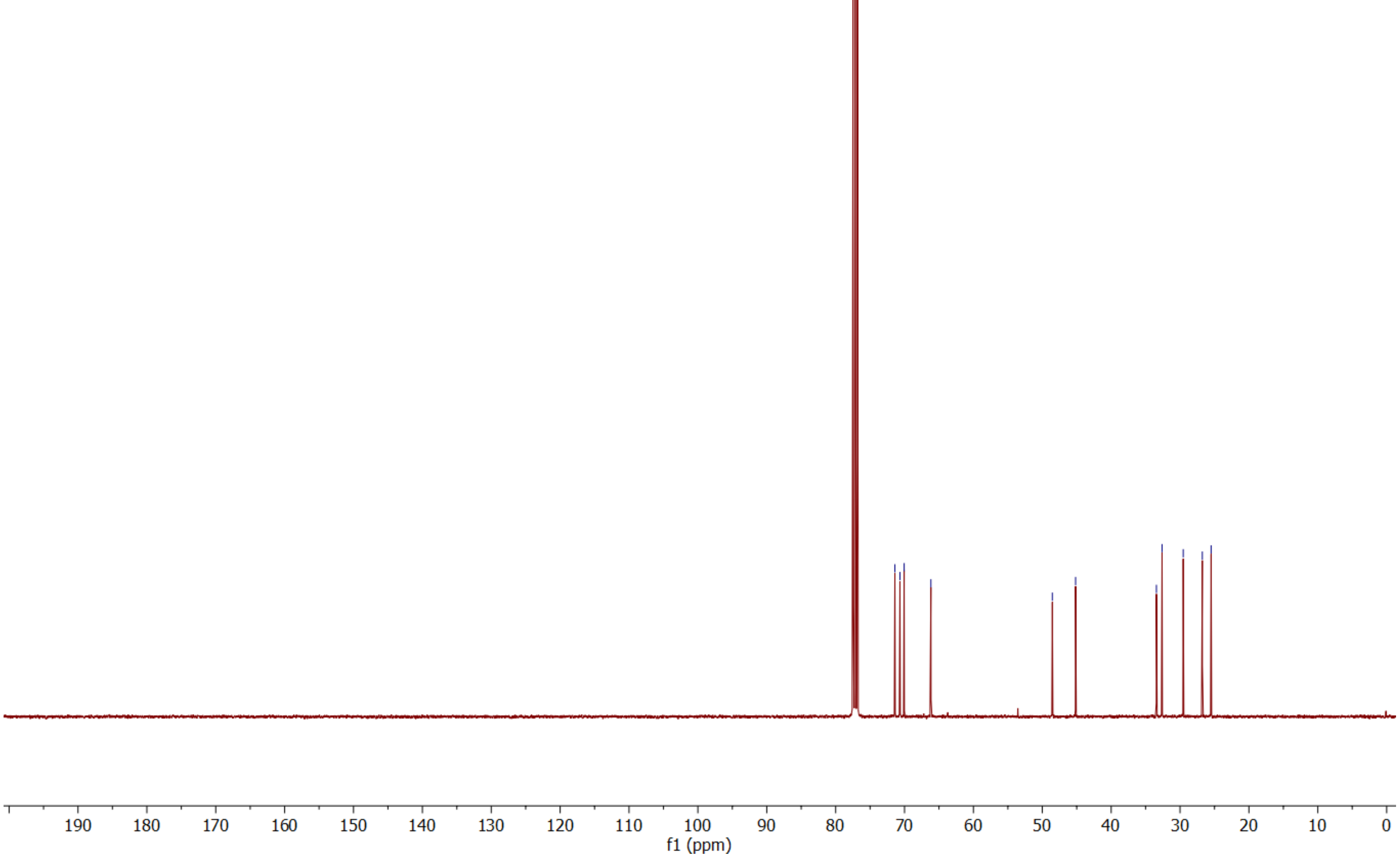

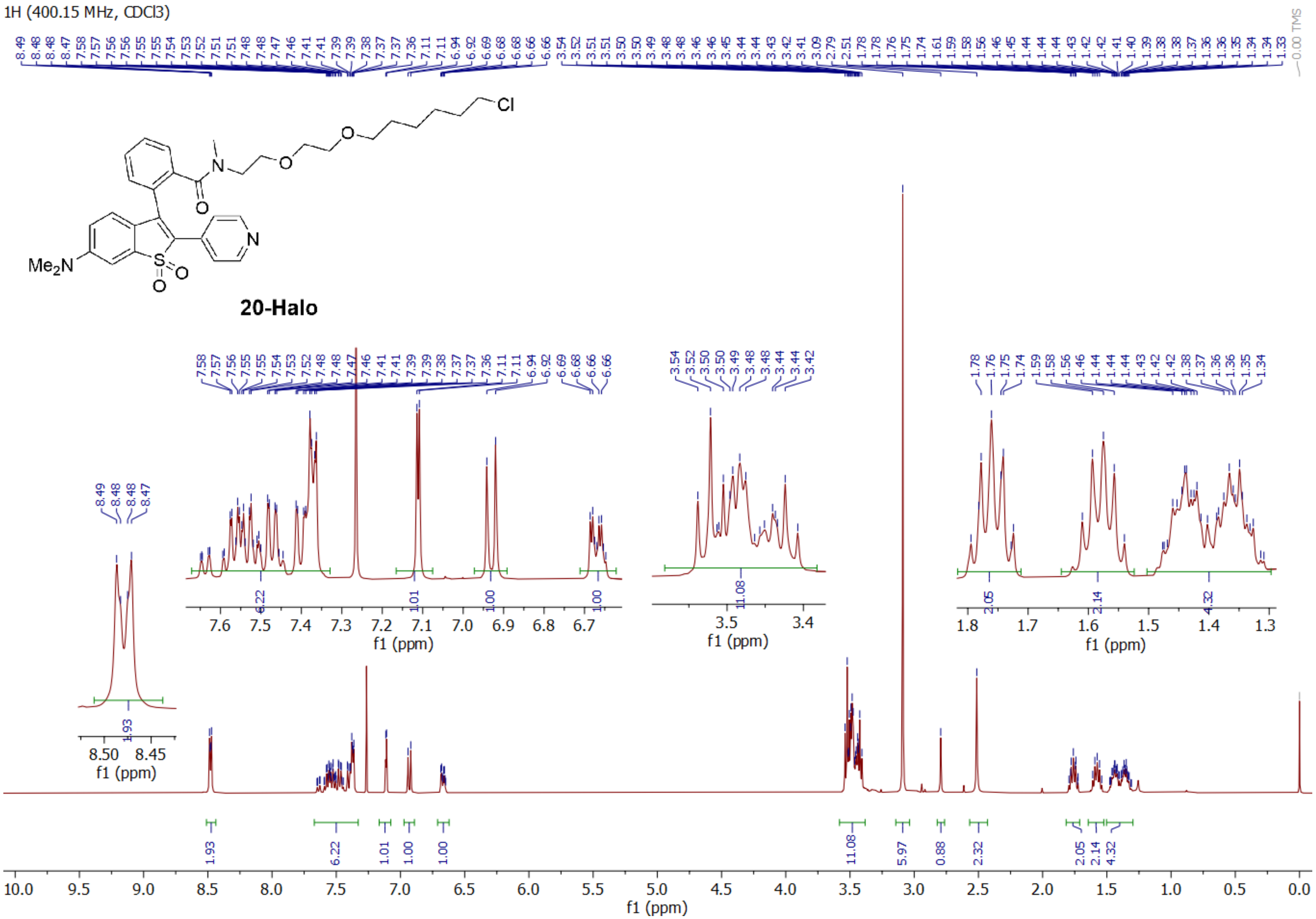
1H (400.15 MHz, CDCl3)
0.00 TMS
Me2N
20-Halo
f1 (ppm)

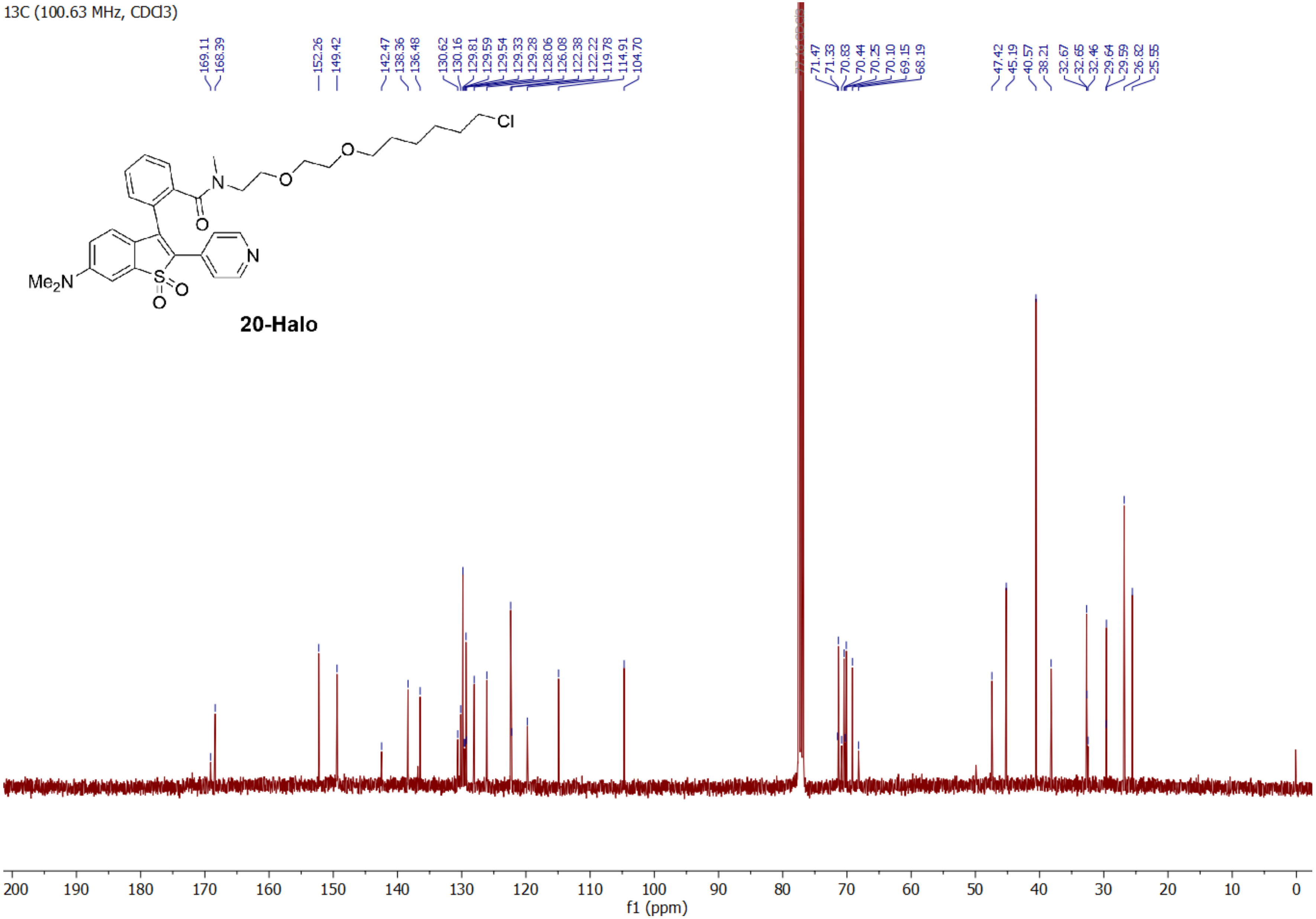
13C (100.63 MHz, CDCl3)
169.11
168.39
152.26
149.42
142.47
138.36
136.48
130.62
130.16
129.81
129.59
129.54
129.33
129.28
128.06
126.08
122.38
122.22
119.78
114.91
104.70
71.47
71.33
70.83
70.44
70.25
70.10
69.15
68.19
47.42
45.19
40.57
38.21
32.67
32.65
32.46
29.64
29.59
26.82
25.55
Cl
O
O
N
O
N
Me2N
S
O
O
20-Halo
200 190 180 170 160 150 140 130 120 110 100 90 80 70 60 50 40 30 20 10 0
f1 (ppm)

1H (400.15 MHz, CDCl3)

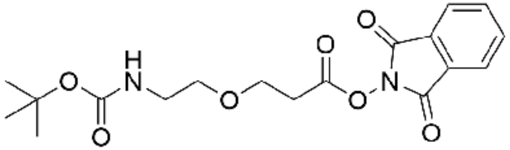

S8

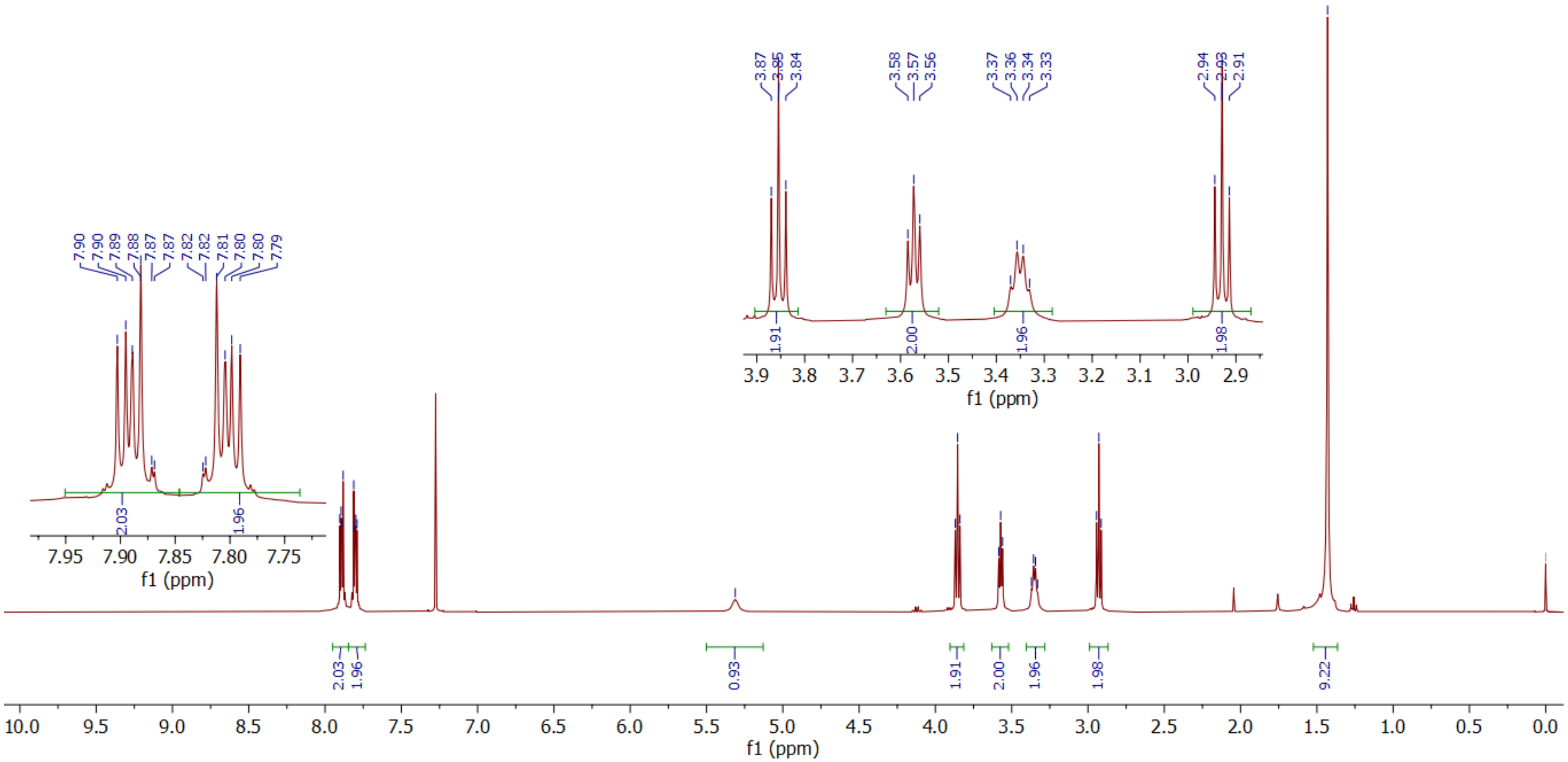

13C (100.63 MHz, CDCl3)

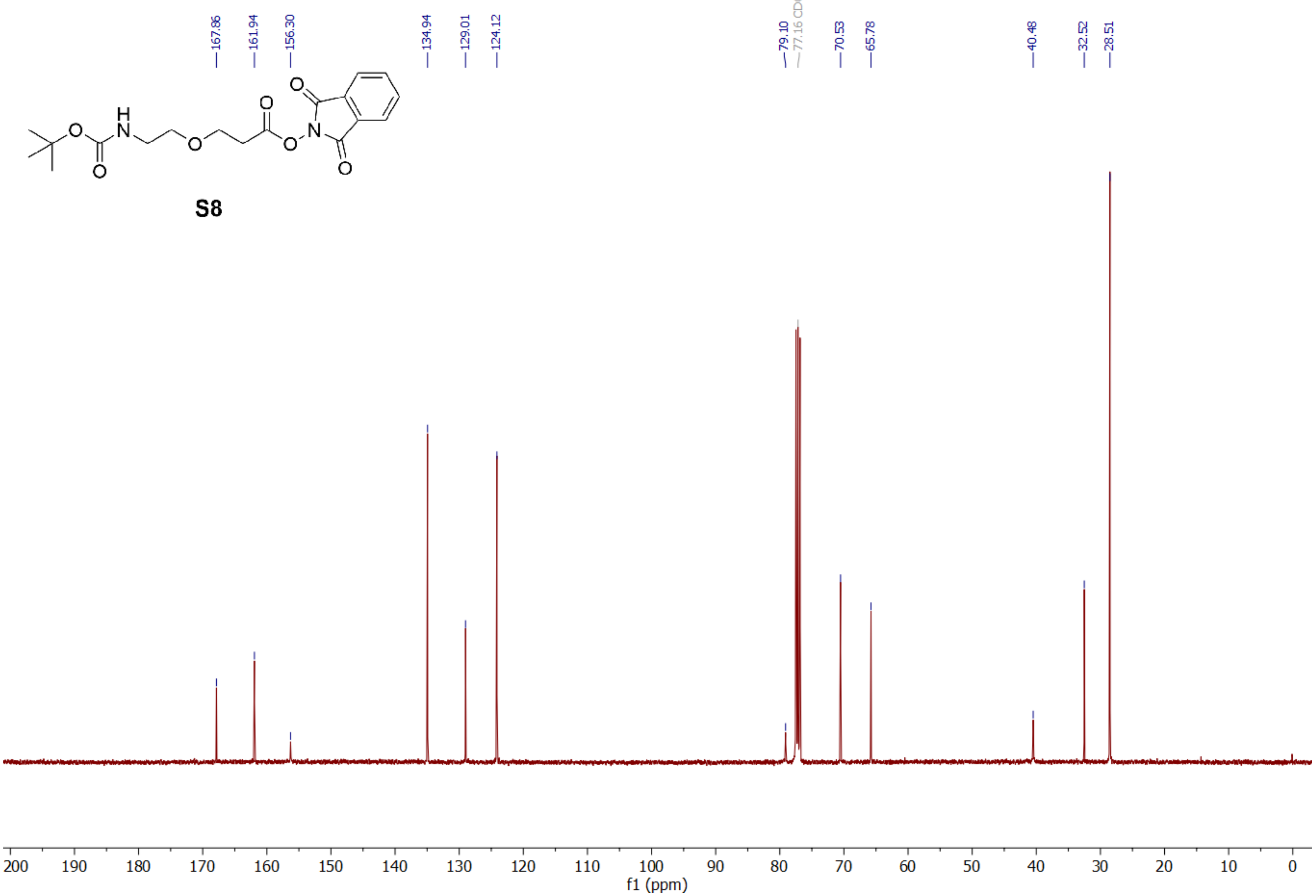

S8

1H (400.15 MHz, CDCl3)

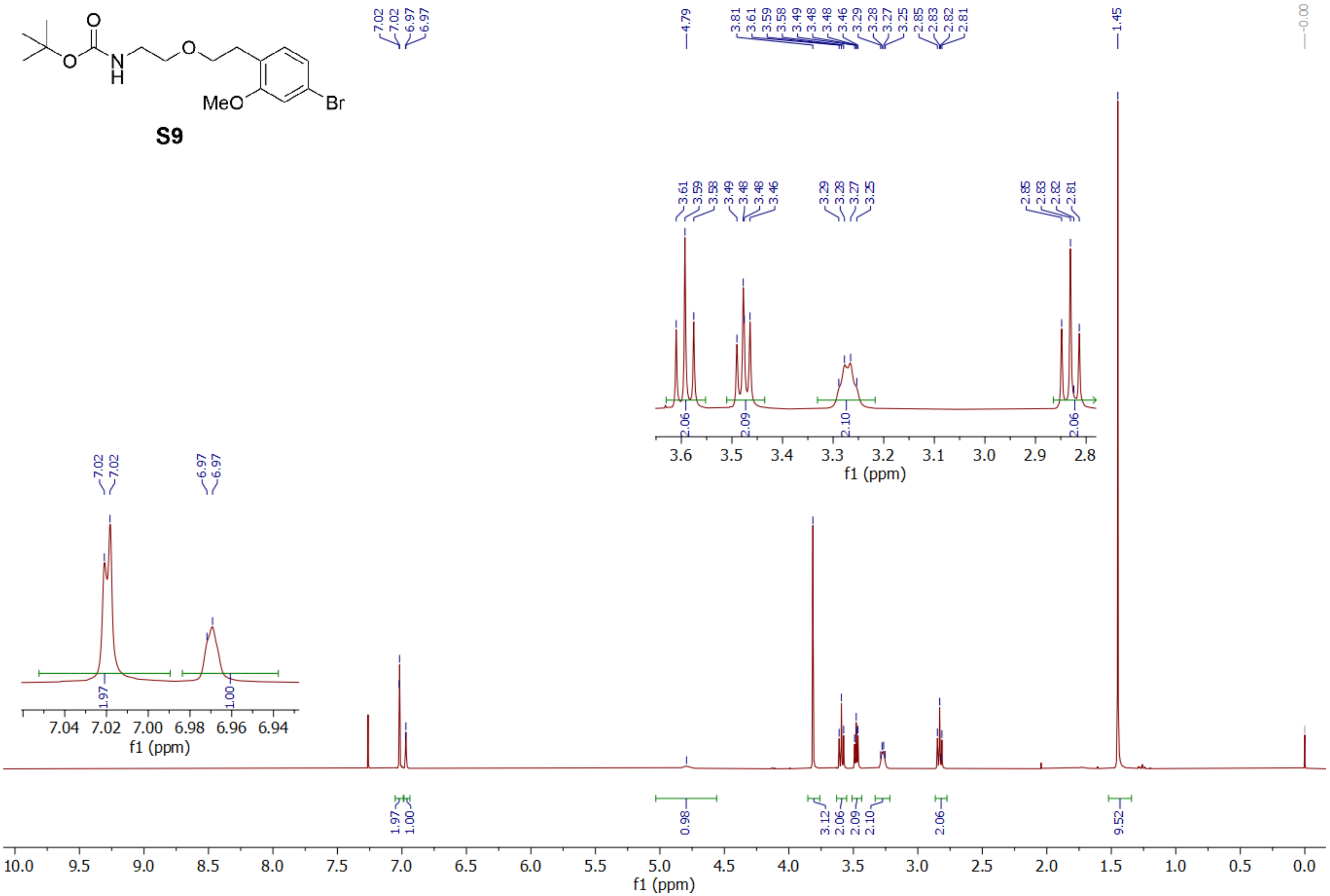

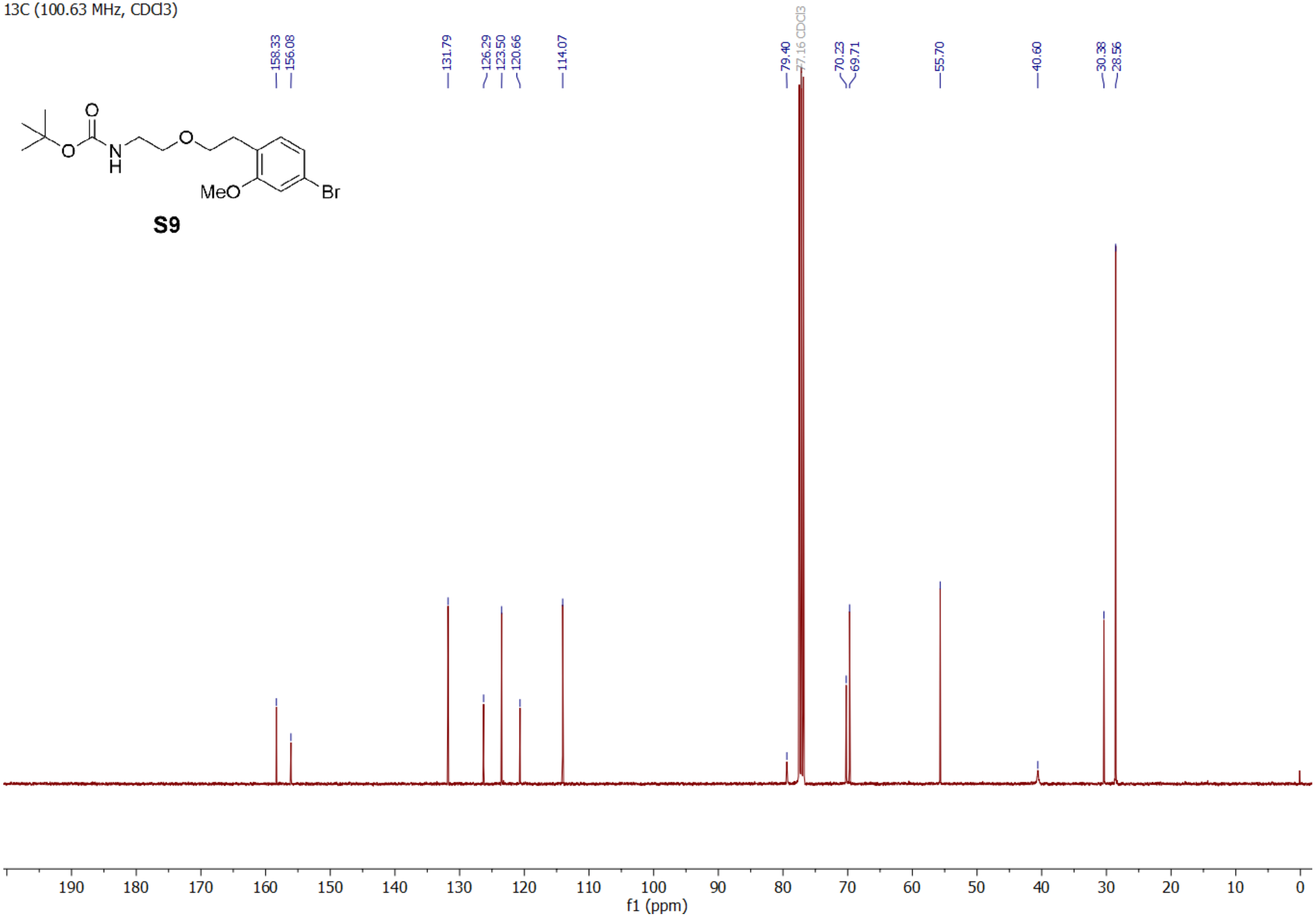
13C (100.63 MHz, CDCl3)
158.33
156.08
131.79
126.29
123.50
120.66
114.07
79.40
77.16 CDCl3
70.23
69.71
55.70
40.60
30.38
28.56
O
O
N
H
O
MeO
Br
S9
190
180
170
160
150
140
130
120
110
100
90
80
70
60
50
40
30
20
10
0
f1 (ppm)

1H (400.15 MHz, CDCl3)

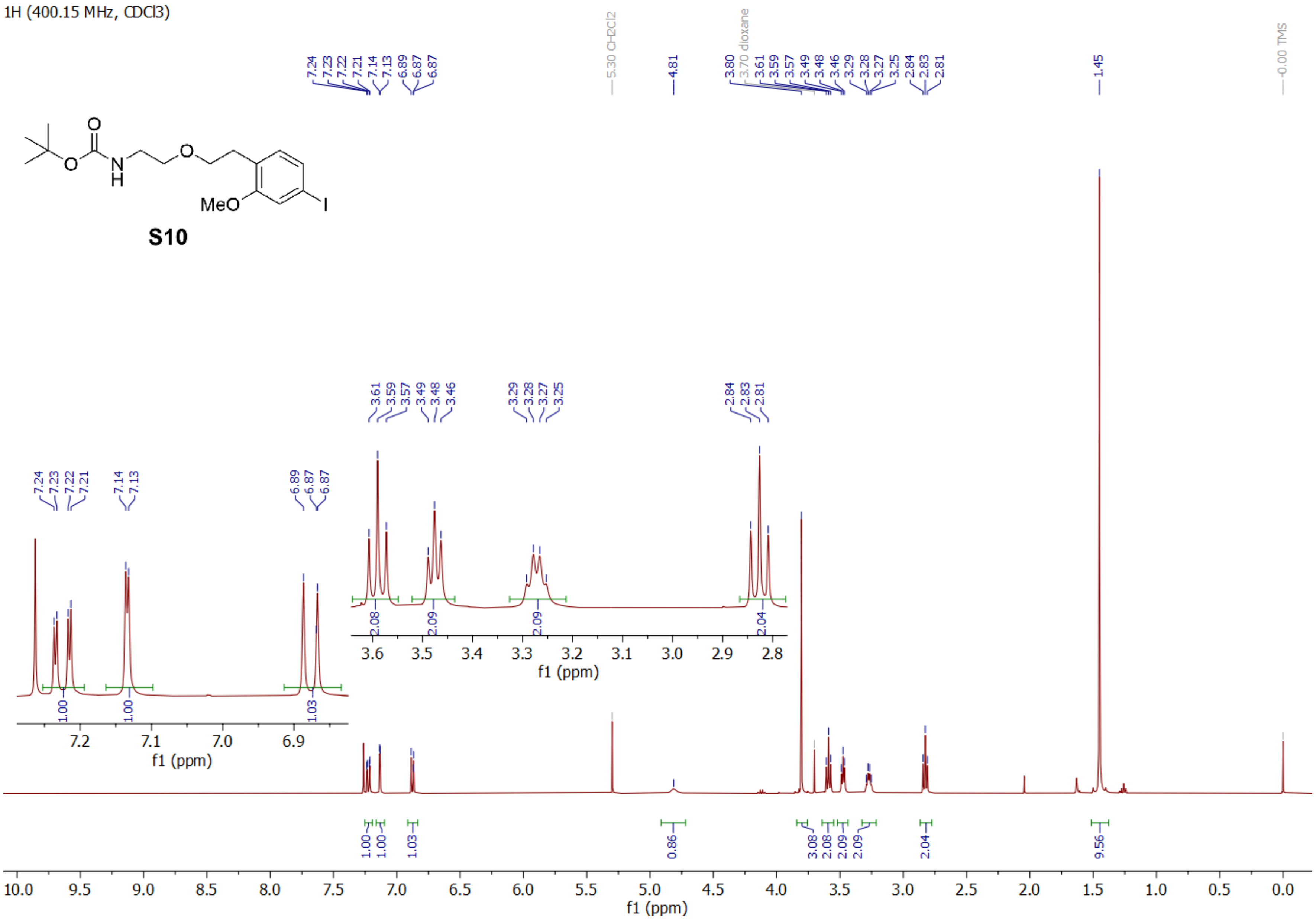

13C (100.63 MHz, CDCl3)

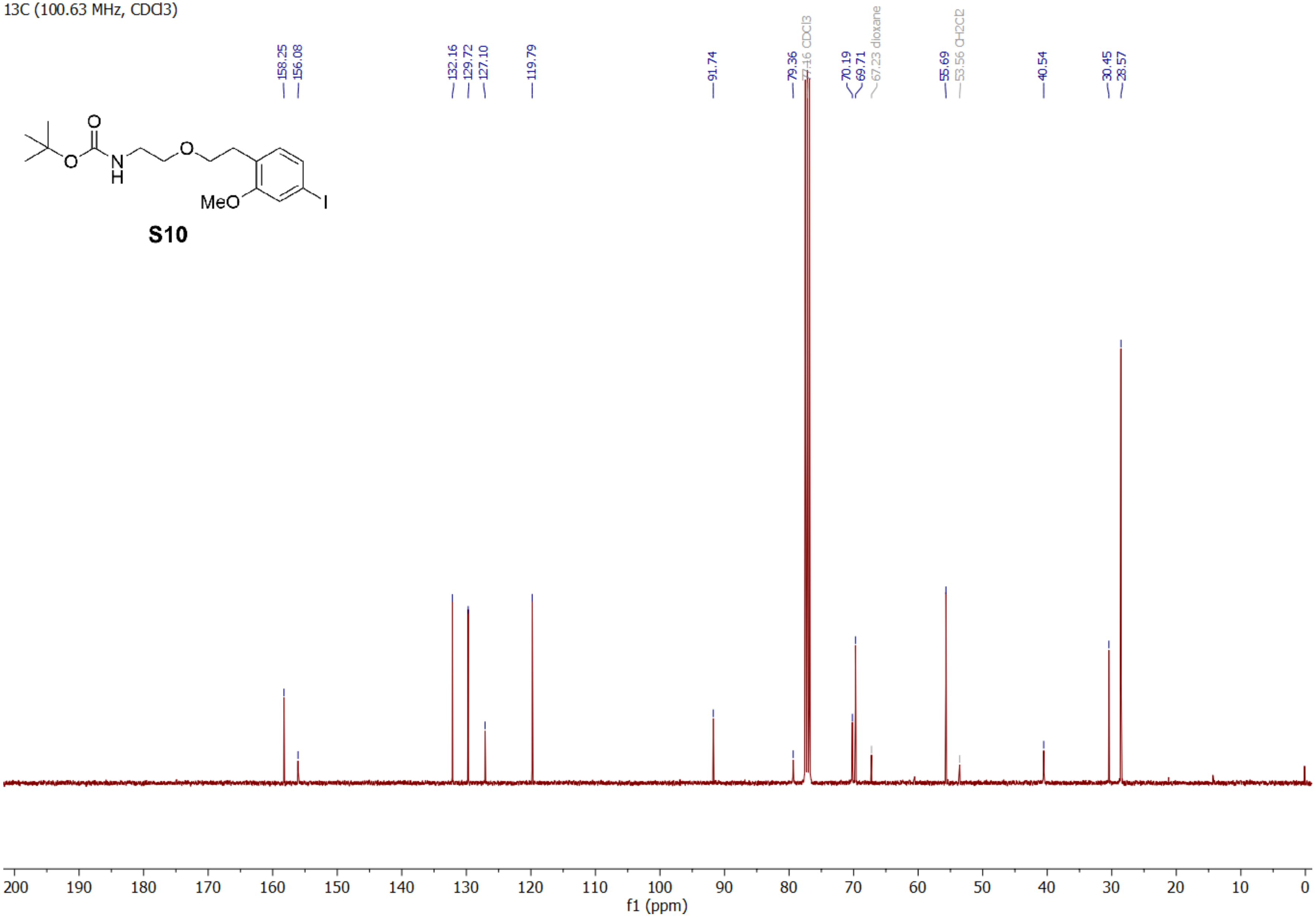

1H (400.15 MHz, CDCl3)

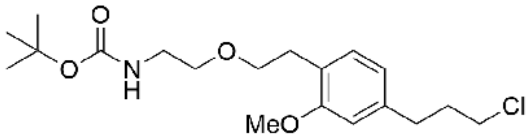

S11

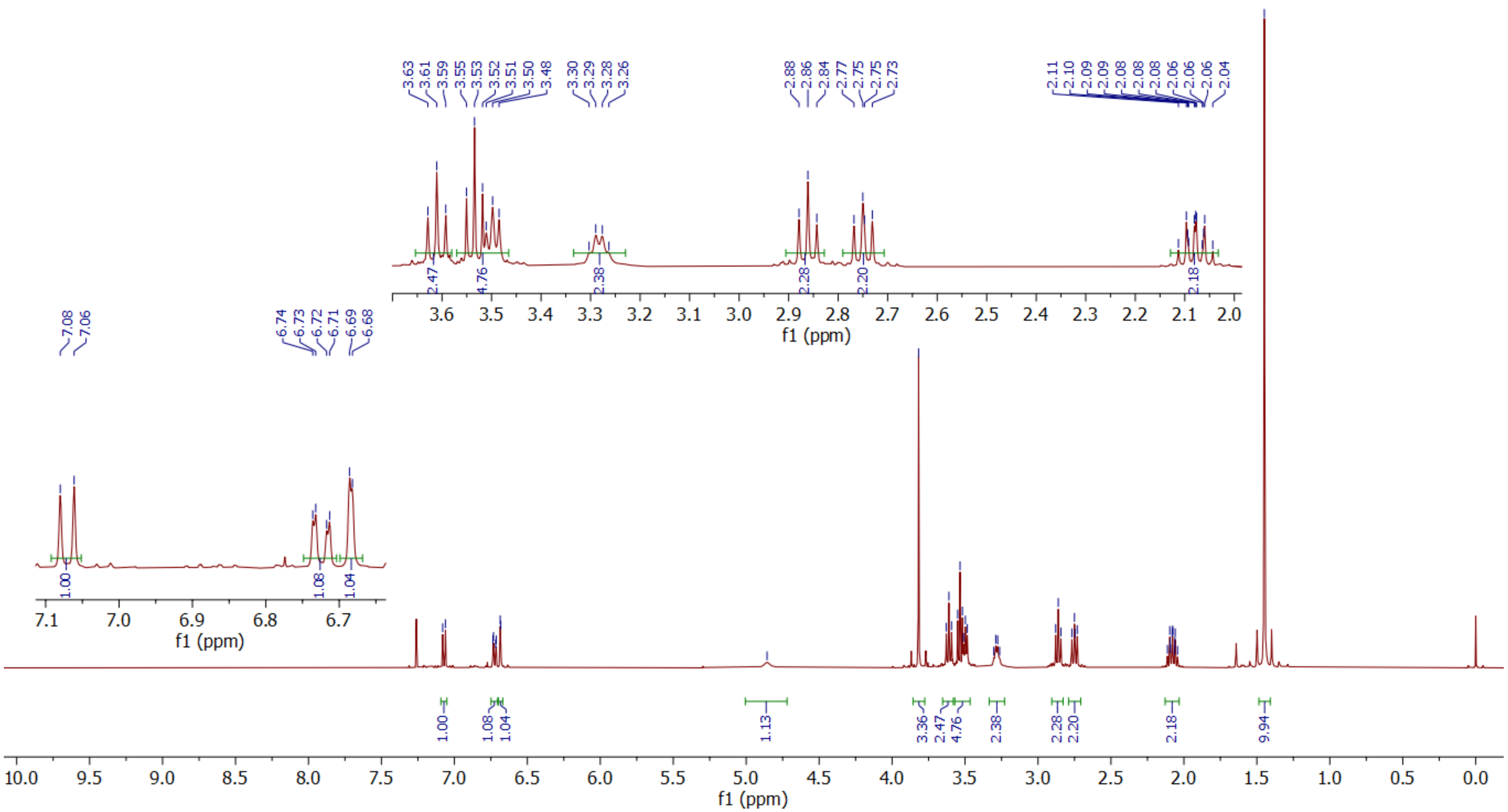

13C (100.63 MHz, CDCl3)

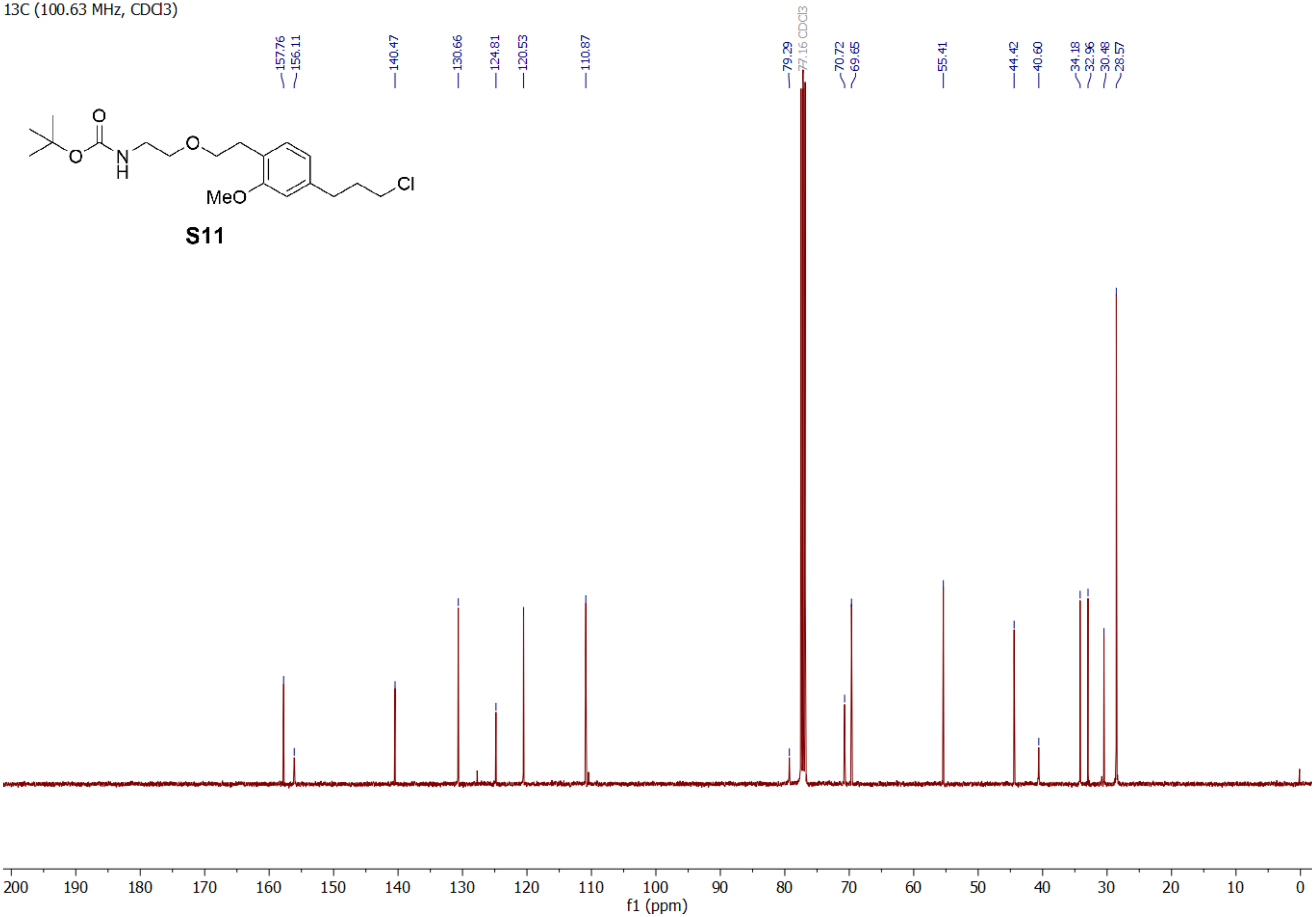

1H (400.15 MHz, CDCl3)

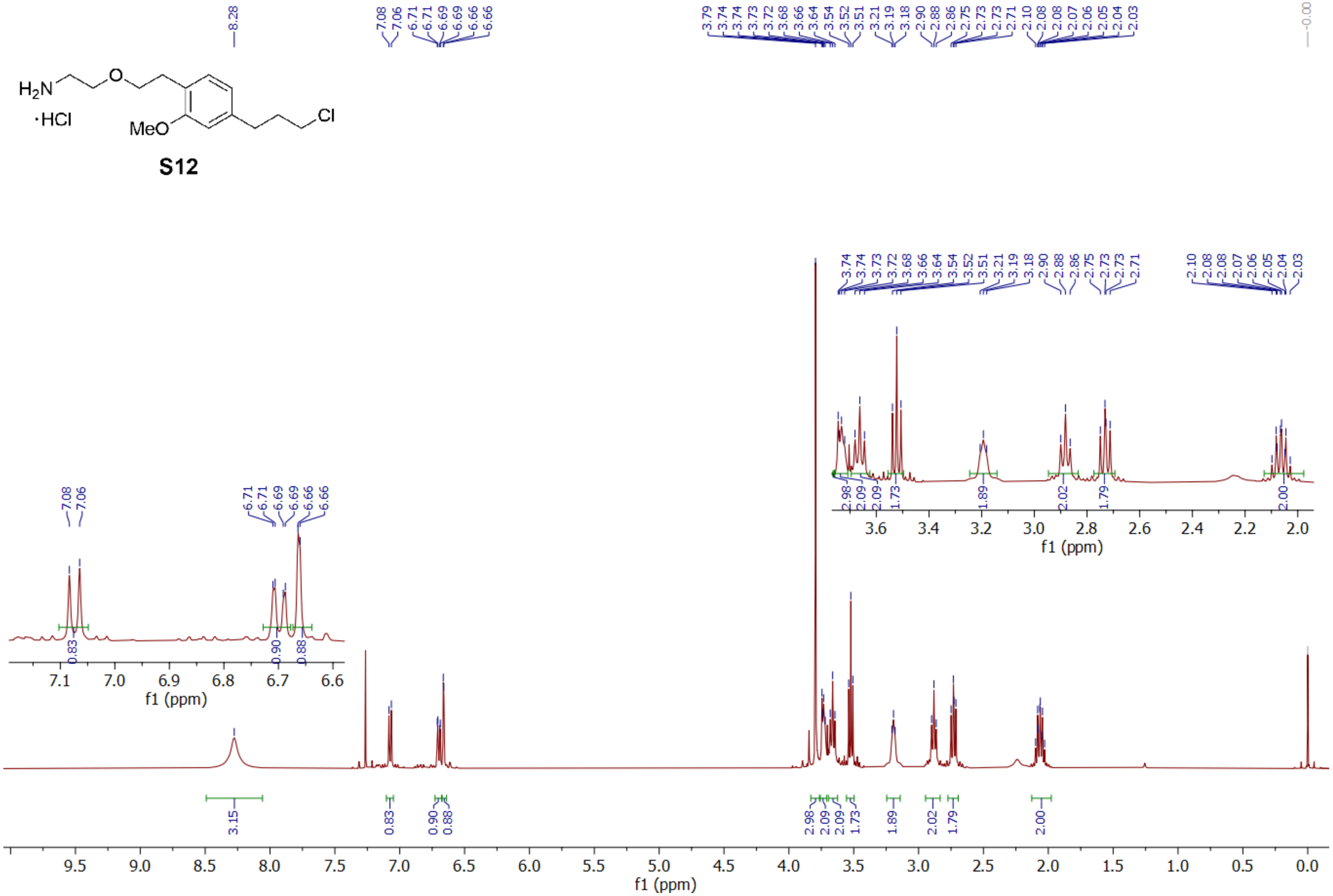

S12

13C (100.63 MHz, CDCl3)

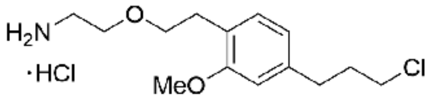

S12

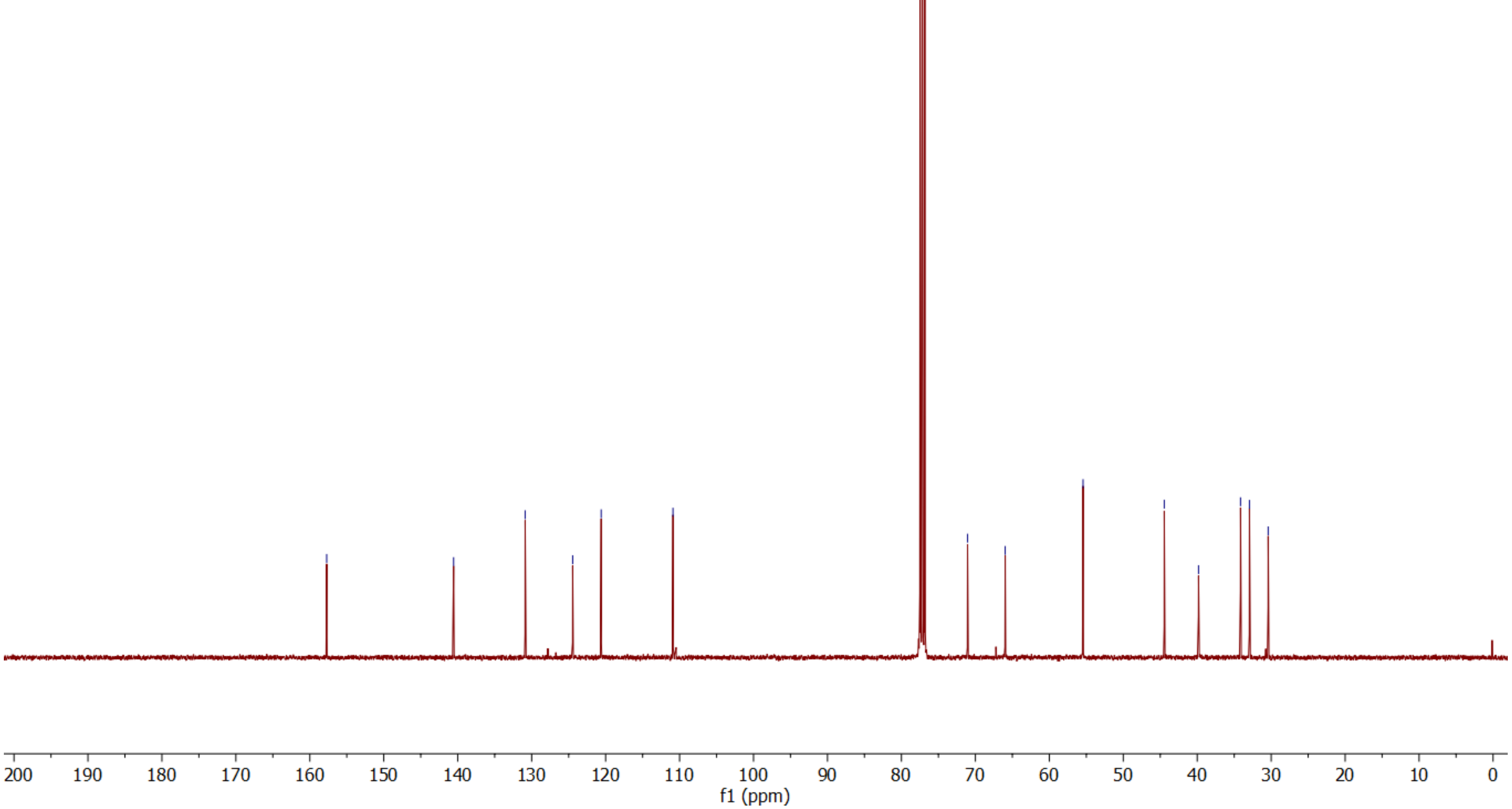

1H (400.15 MHz, CDCl3)

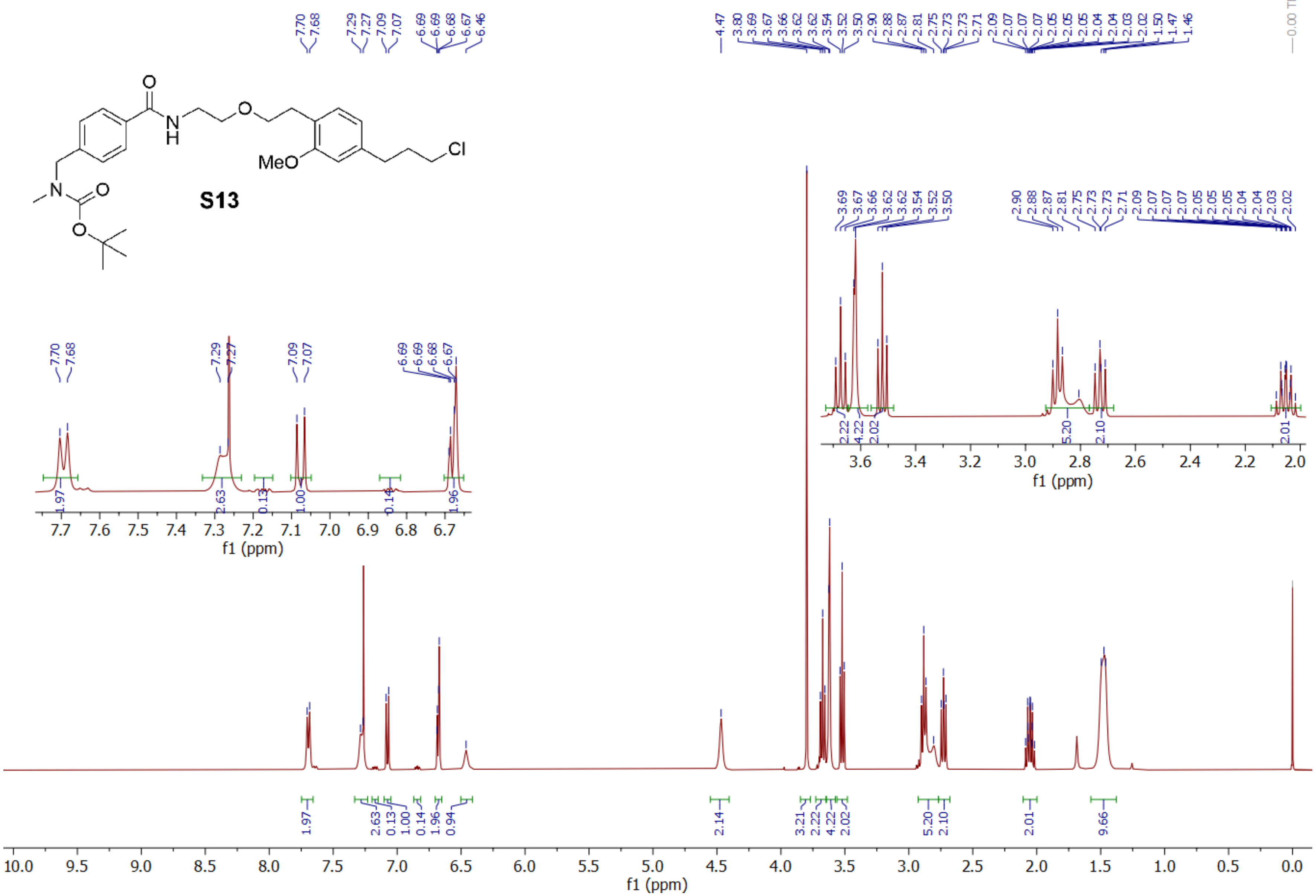

13C (100.63 MHz, CDCl3)

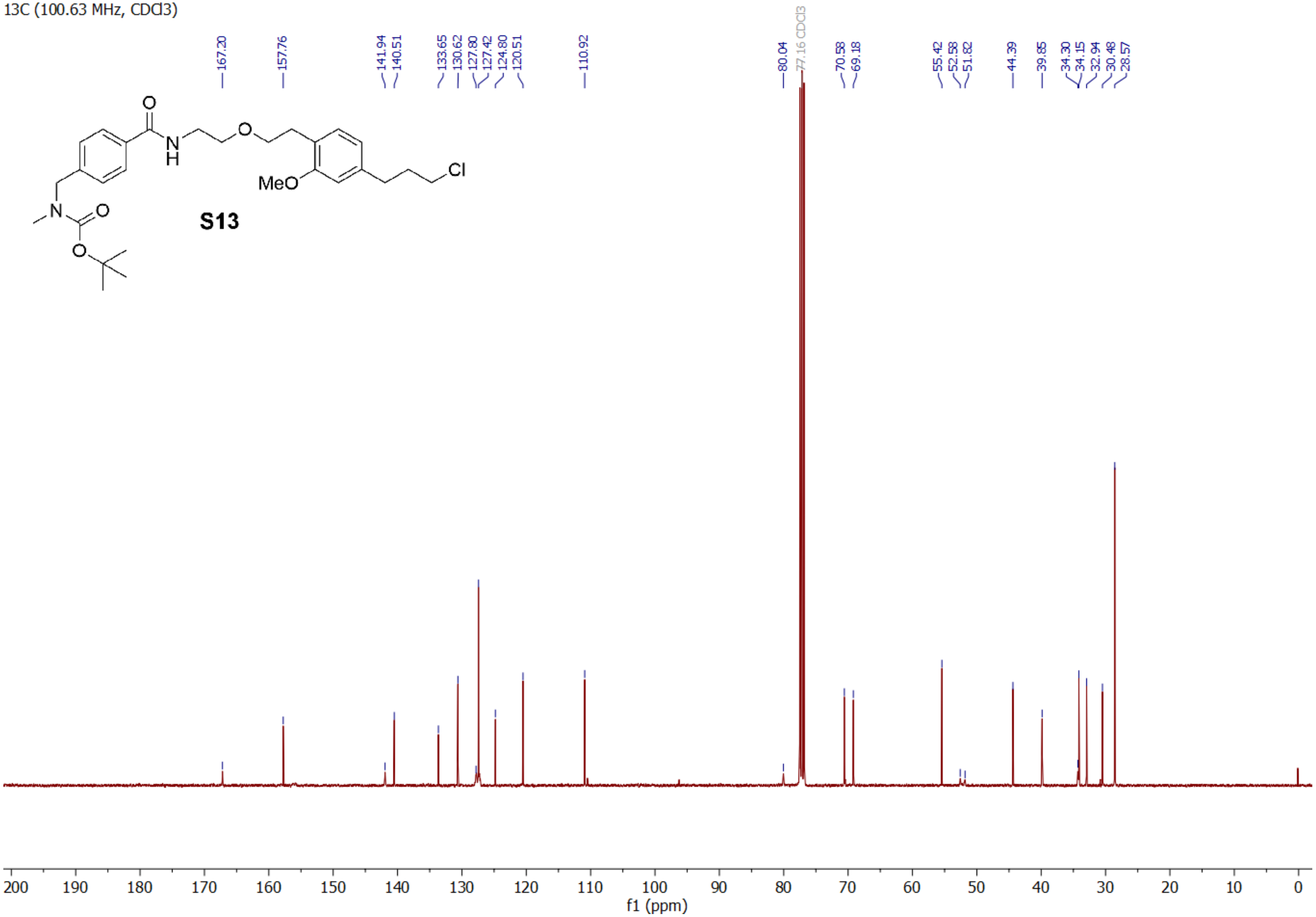

1H (400.15 MHz, CDCl3)

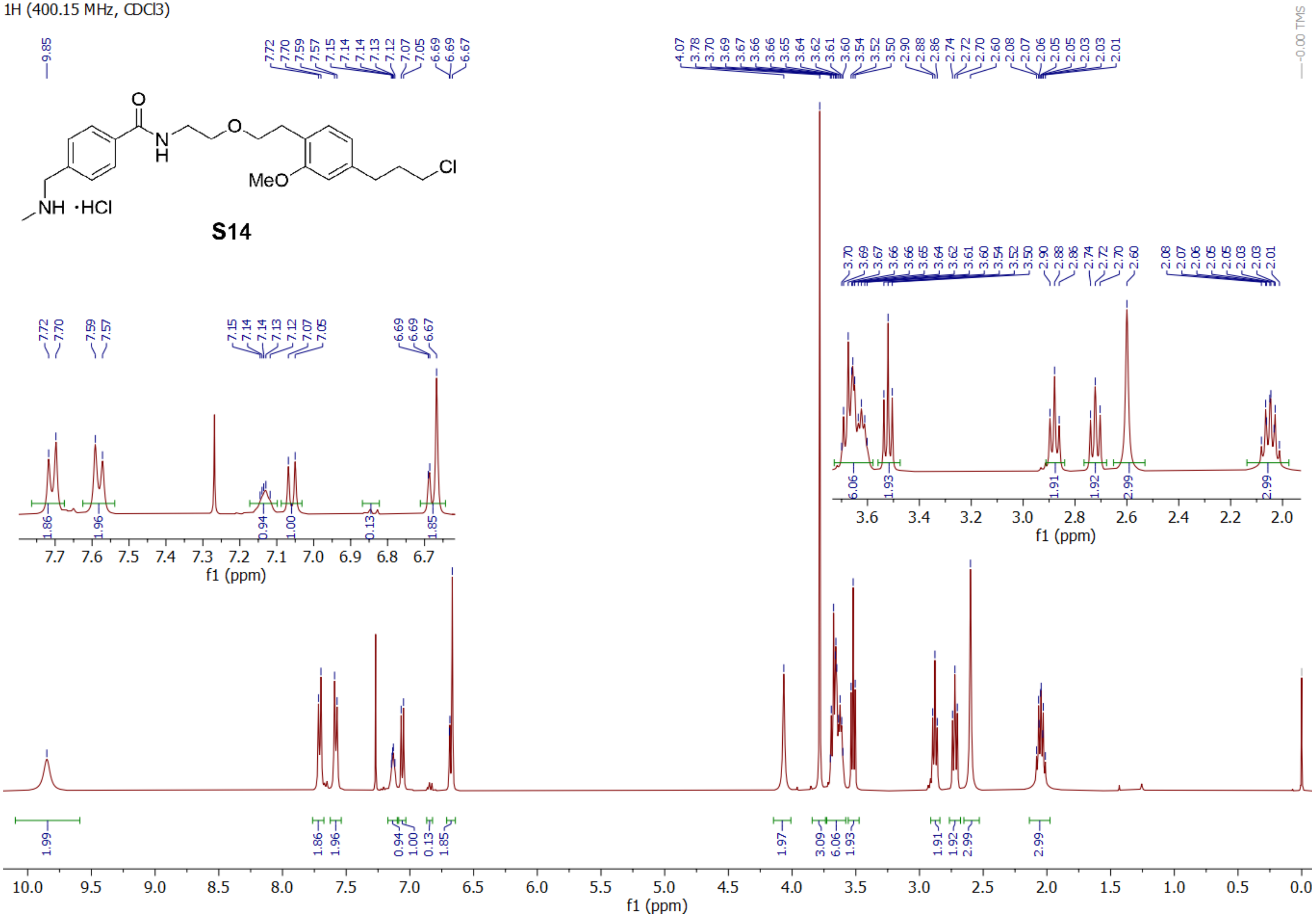

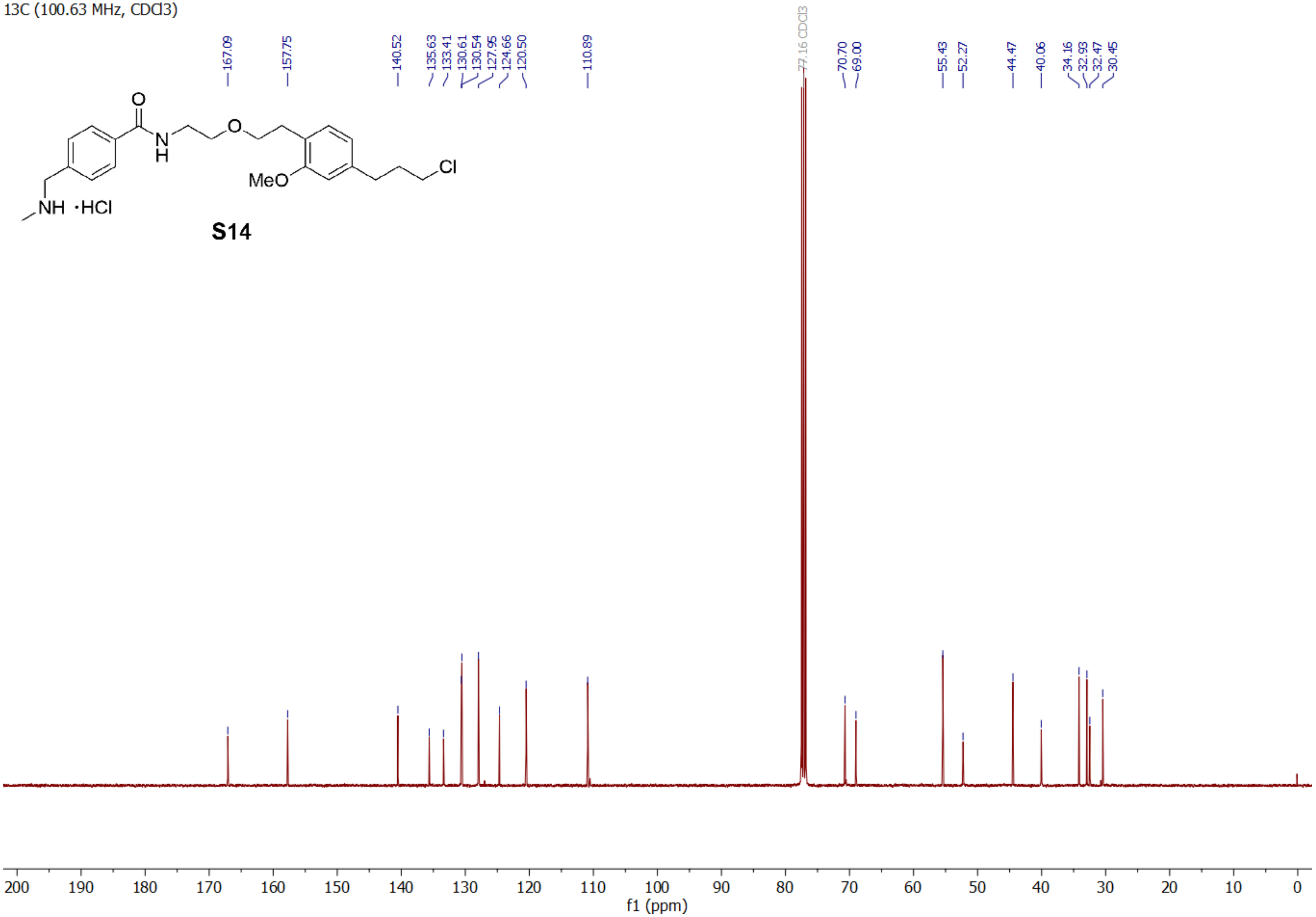
13C (100.63 MHz, CDCl3)
167.09
157.75
140.52
135.63
133.41
130.61
130.54
127.95
124.66
120.50
110.89
77.16 CDCl3
70.70
69.00
55.43
52.27
44.47
40.06
34.16
32.93
32.47
30.45
O
N
H
O
MeO
Cl
NH ·HCl
S14
f1 (ppm)

1H (400.15 MHz, CDCl3)

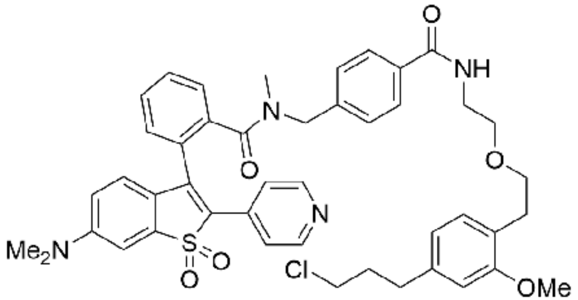

20-HTL2

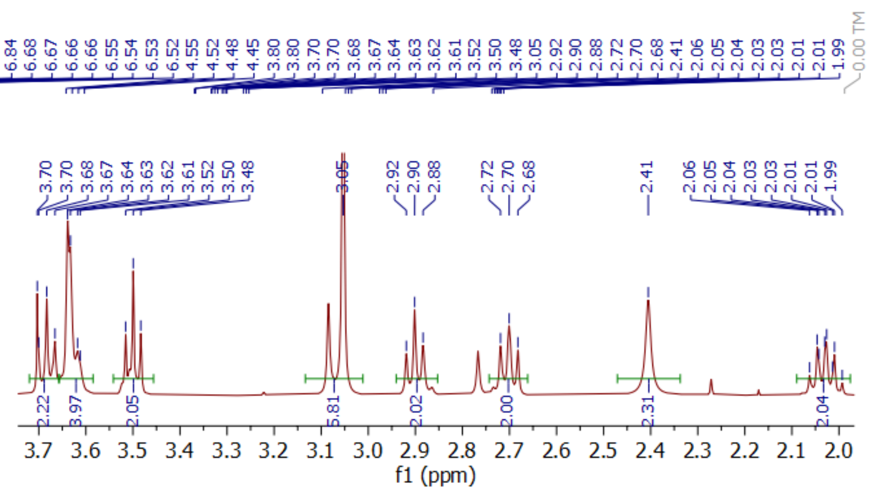

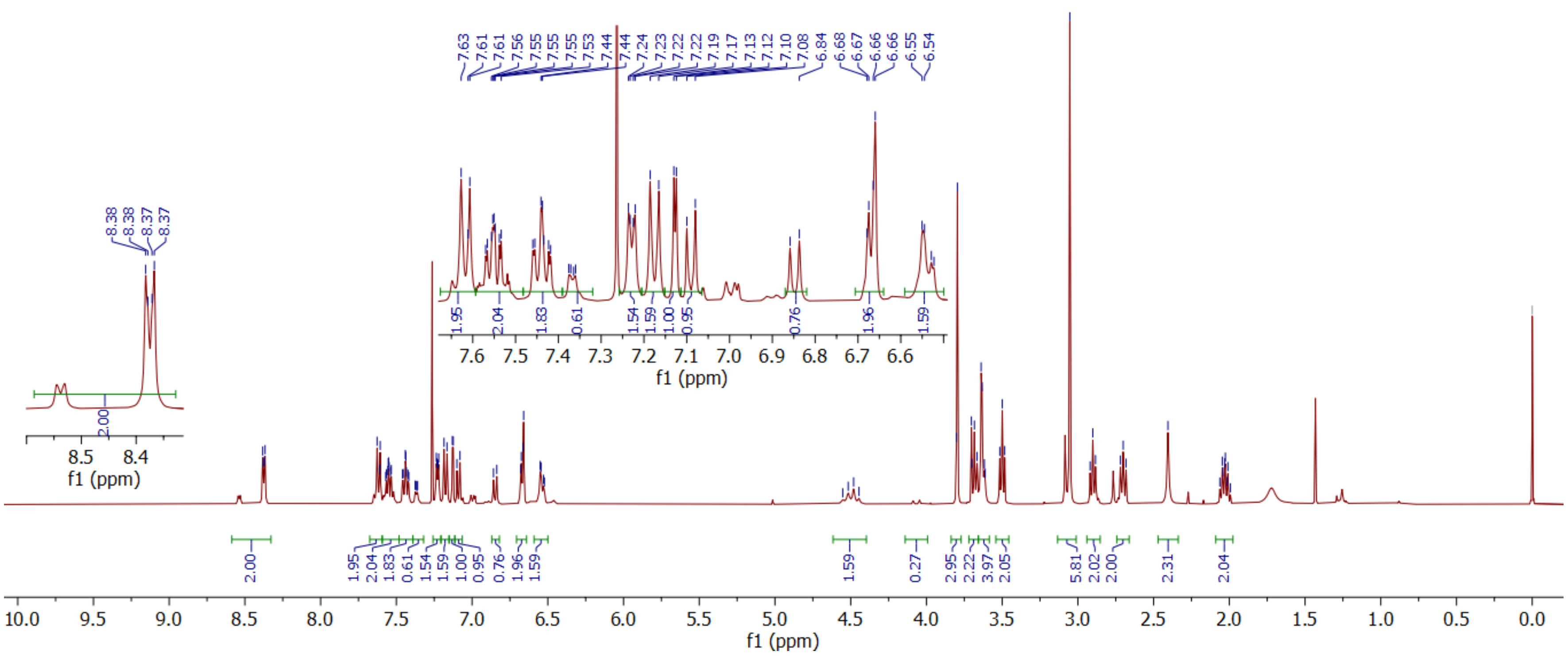

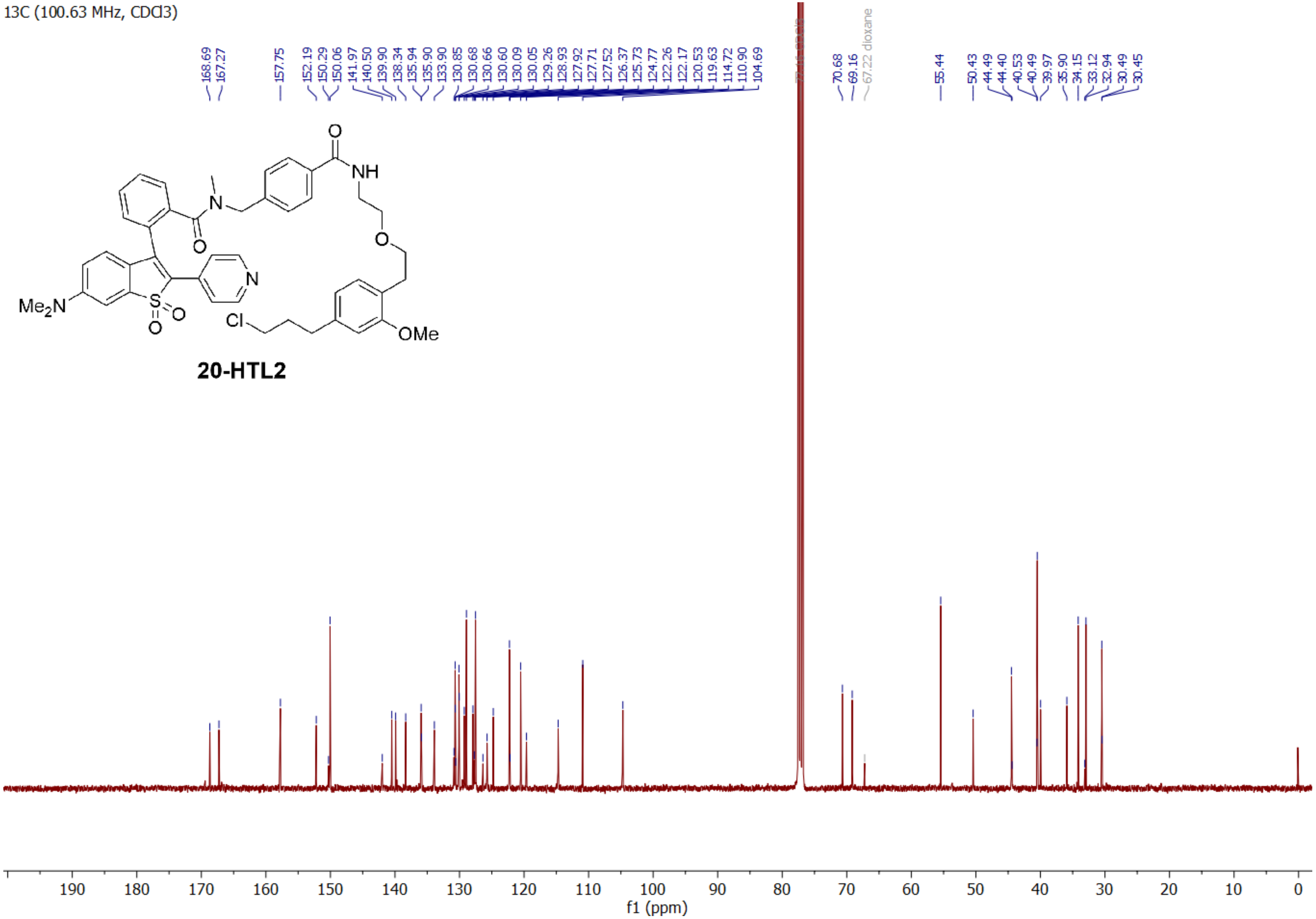
13C (100.63 MHz, CDCl3)
168.69
167.27
157.75
152.19
150.29
150.06
141.97
140.50
139.90
138.34
135.94
135.90
133.90
130.85
130.68
130.66
130.60
130.09
130.05
129.26
128.93
127.92
127.71
127.52
126.37
125.73
124.77
122.26
122.17
120.53
119.63
114.72
110.90
104.69
70.68
69.16
67.22 dioxane
55.44
50.43
44.49
44.40
40.53
40.49
39.97
35.90
34.15
33.12
32.94
30.49
30.45
Me2N
NH
OMe
Cl
f1 (ppm)

20-HTL2

1H (400.15 MHz, CDCl3)

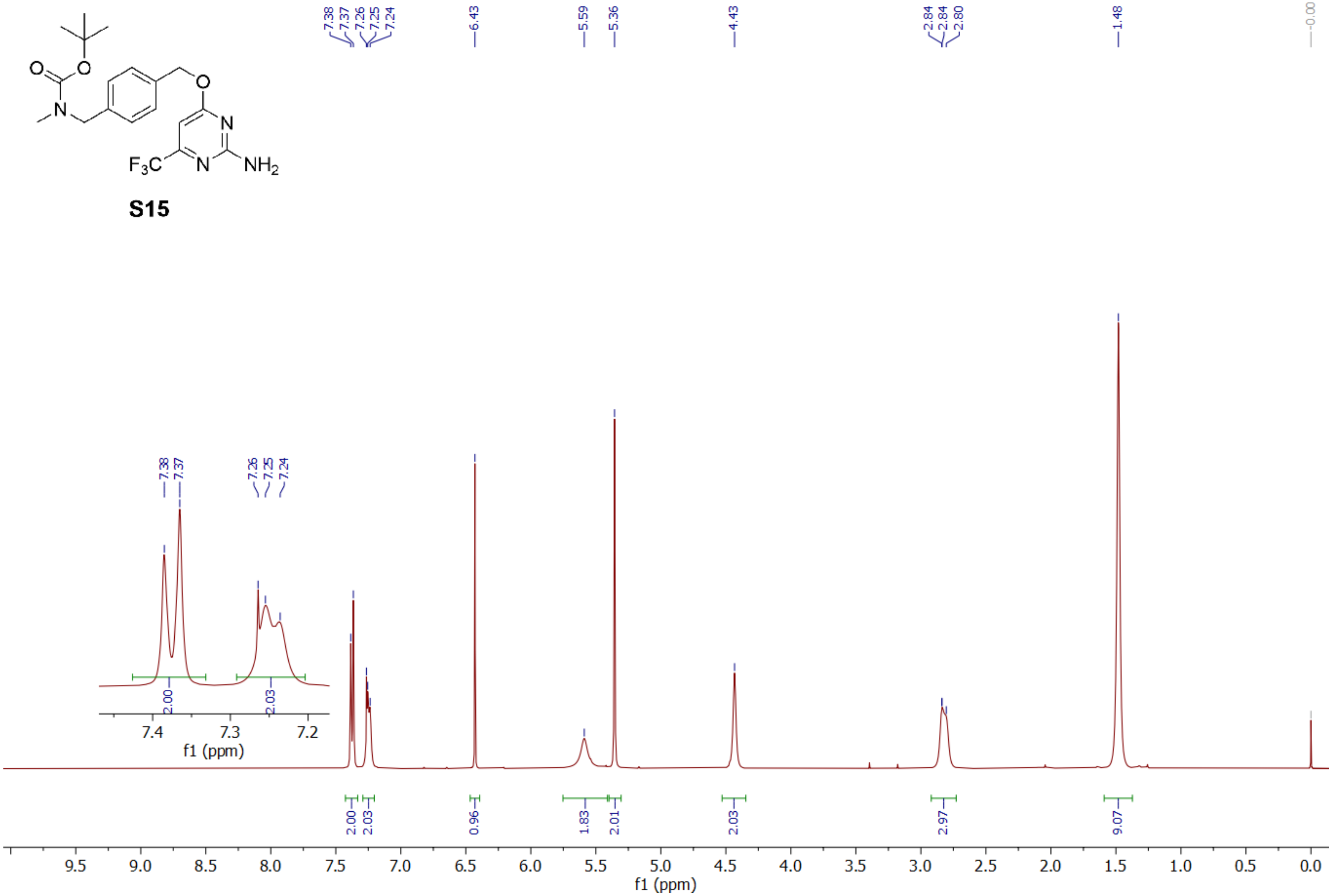

S15

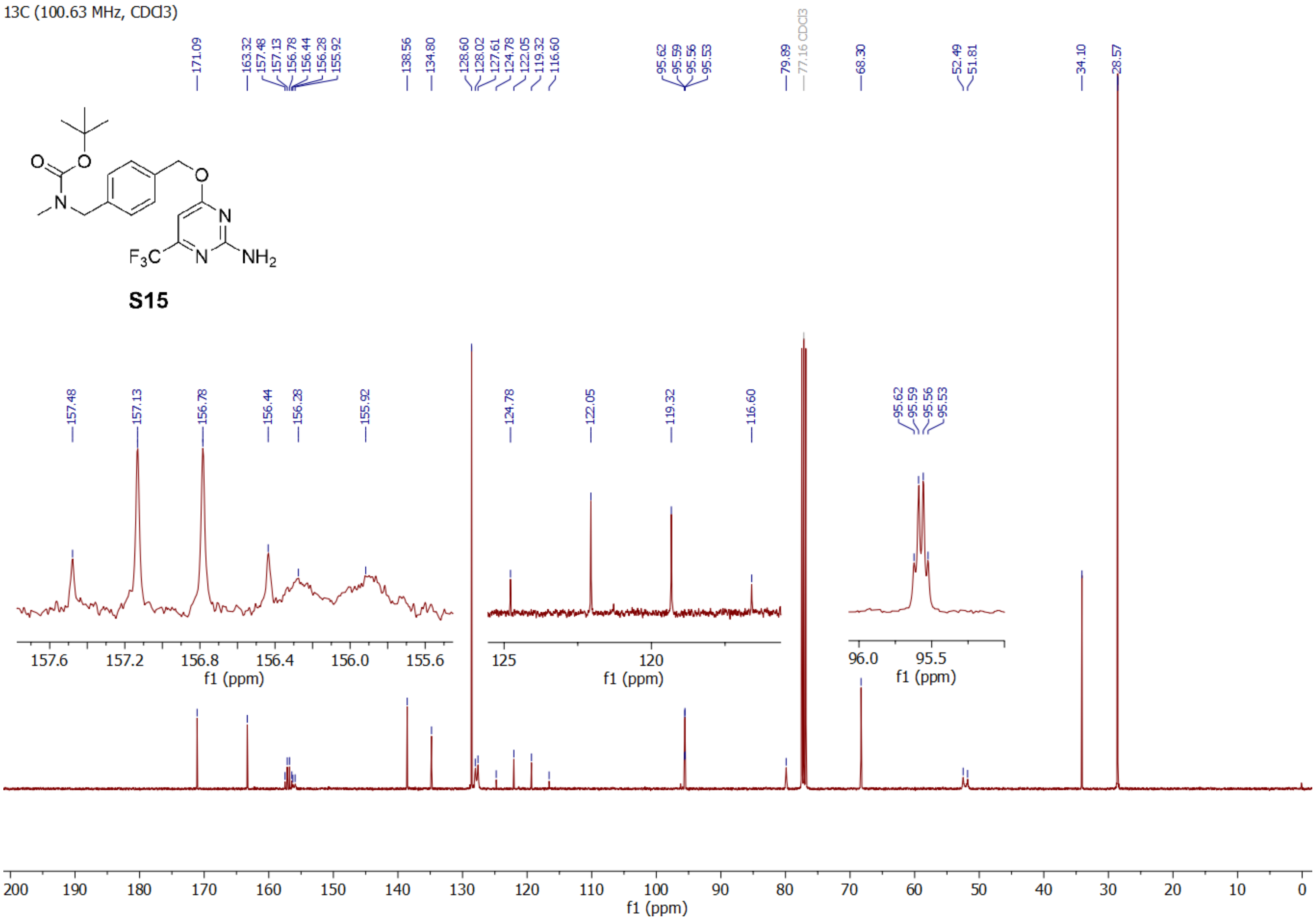
13C (100.63 MHz, CDCl3)
171.09
163.32
157.48
157.13
156.78
156.44
156.28
155.92
138.56
134.80
128.60
128.02
127.61
124.78
122.05
119.32
116.60
95.62
95.59
95.56
95.53
79.89
77.16 CDCl3
68.30
52.49
51.81
34.10
28.57
O
O
N
O
N
F3C
N
NH2
S15
f1 (ppm)

19F (376.48 MHz, CDCl3)

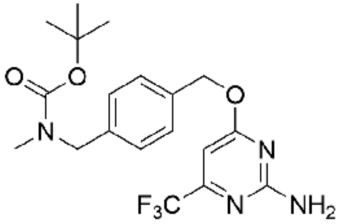

S15

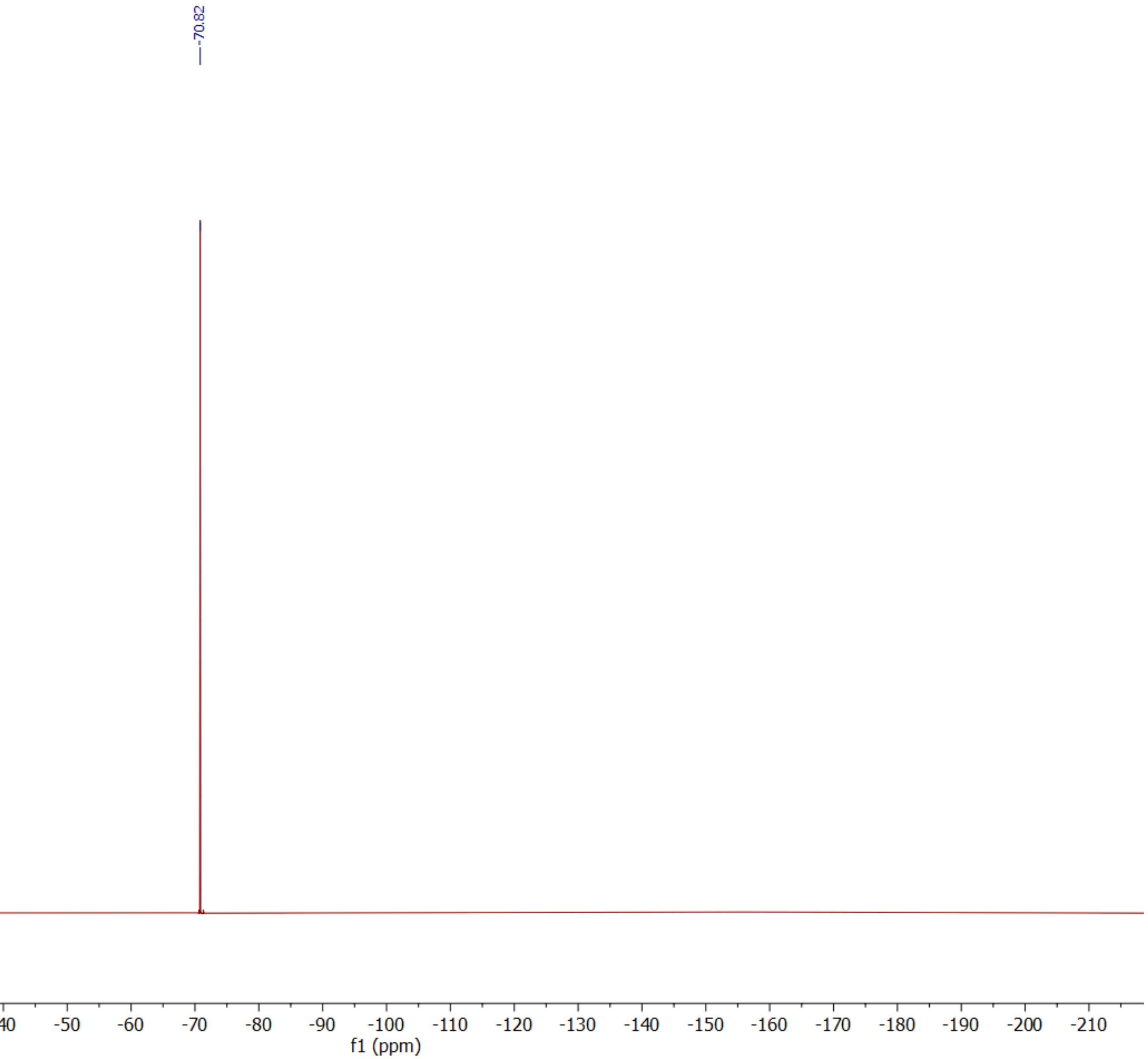

1H (400.15 MHz, DMSO)

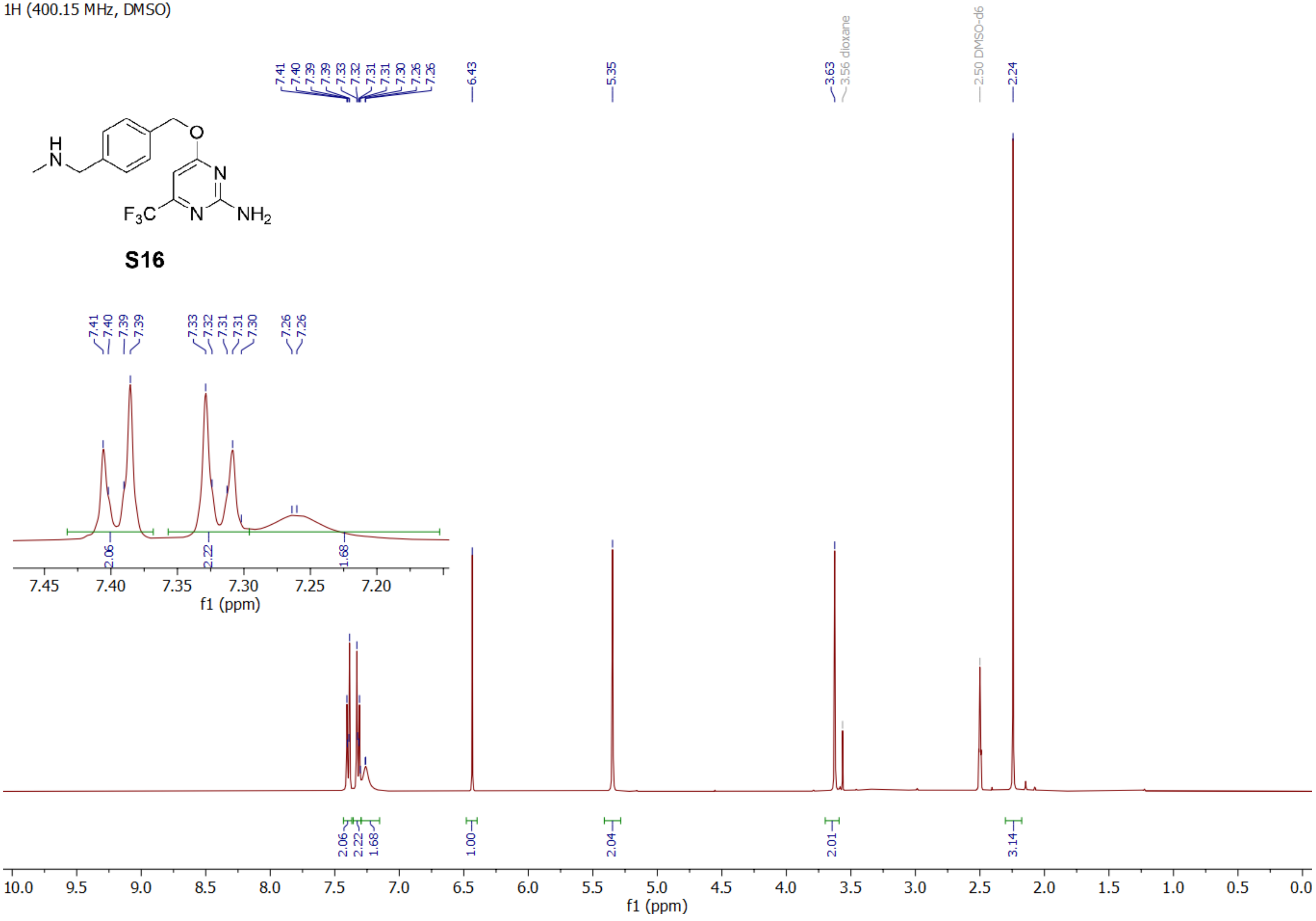

S16

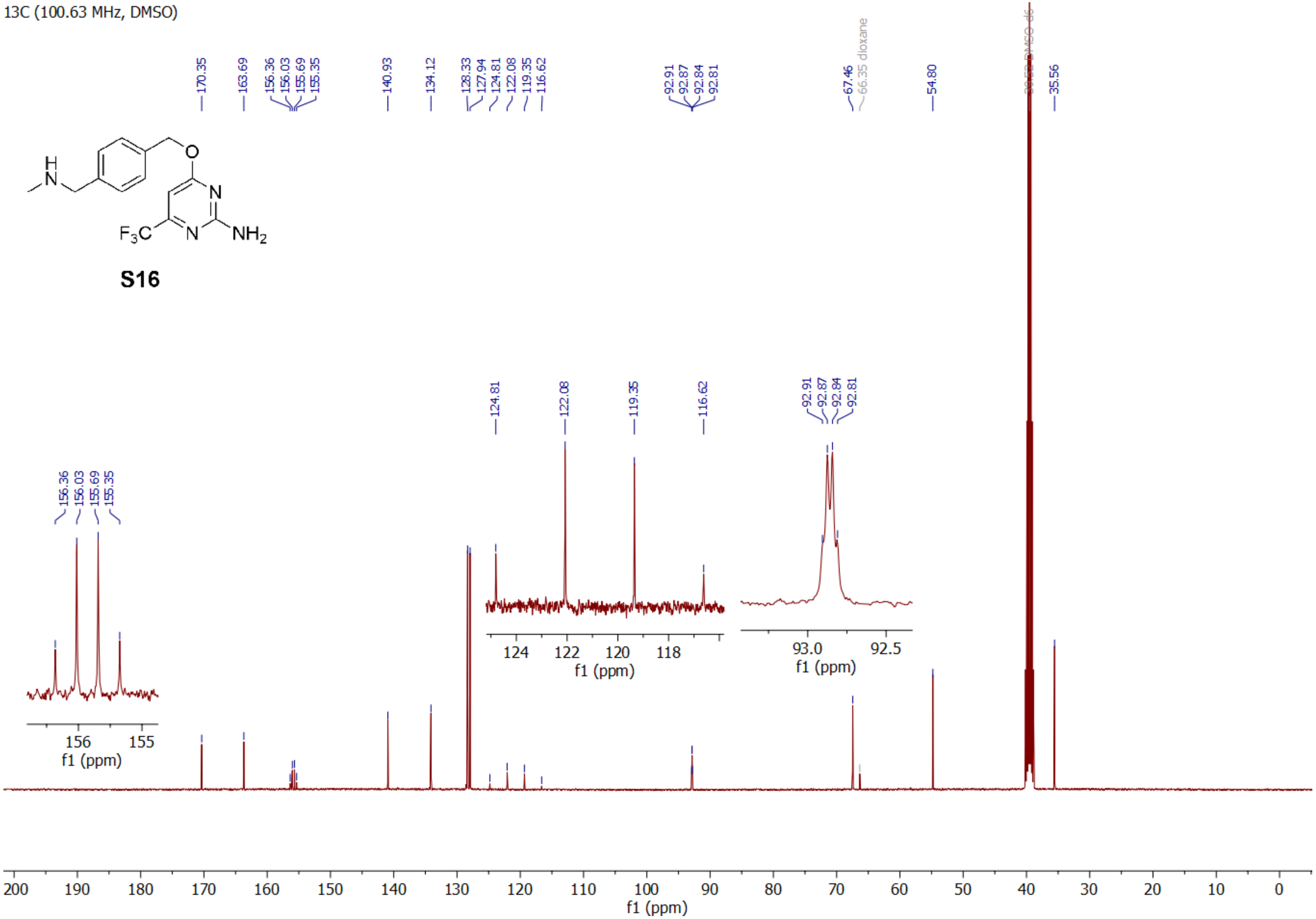

13C (100.63 MHz, DMSO)
170.35
163.69
156.36
156.03
155.69
155.35
140.93
134.12
128.33
127.94
124.81
122.08
119.35
116.62
92.91
92.87
92.84
92.81
67.46
66.35 dioxane
54.80
39.52 DMSO-d6
35.56
S16
f1 (ppm)

19F (376.48 MHz, DMSO)

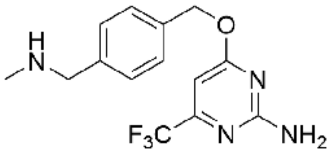

S16

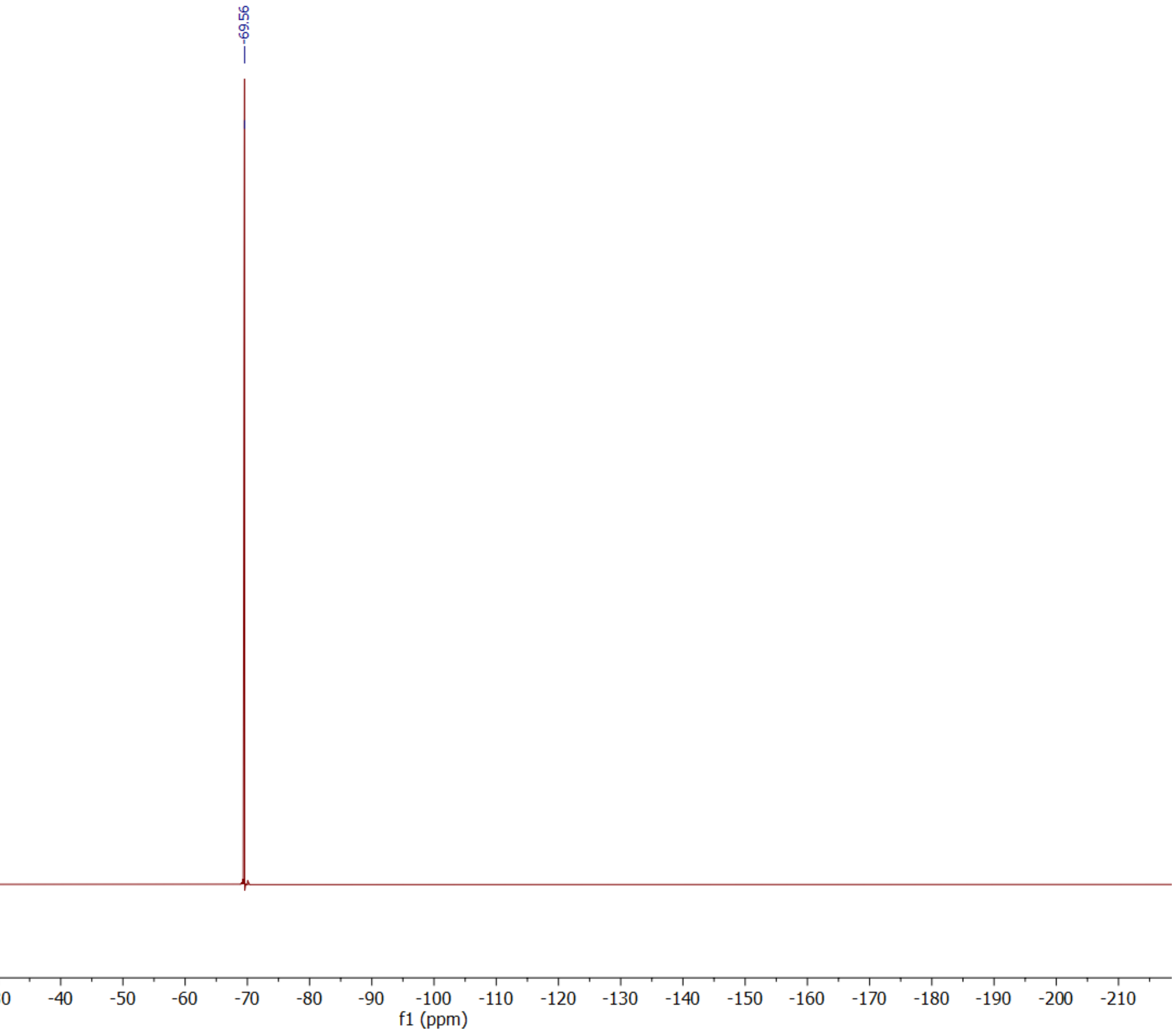

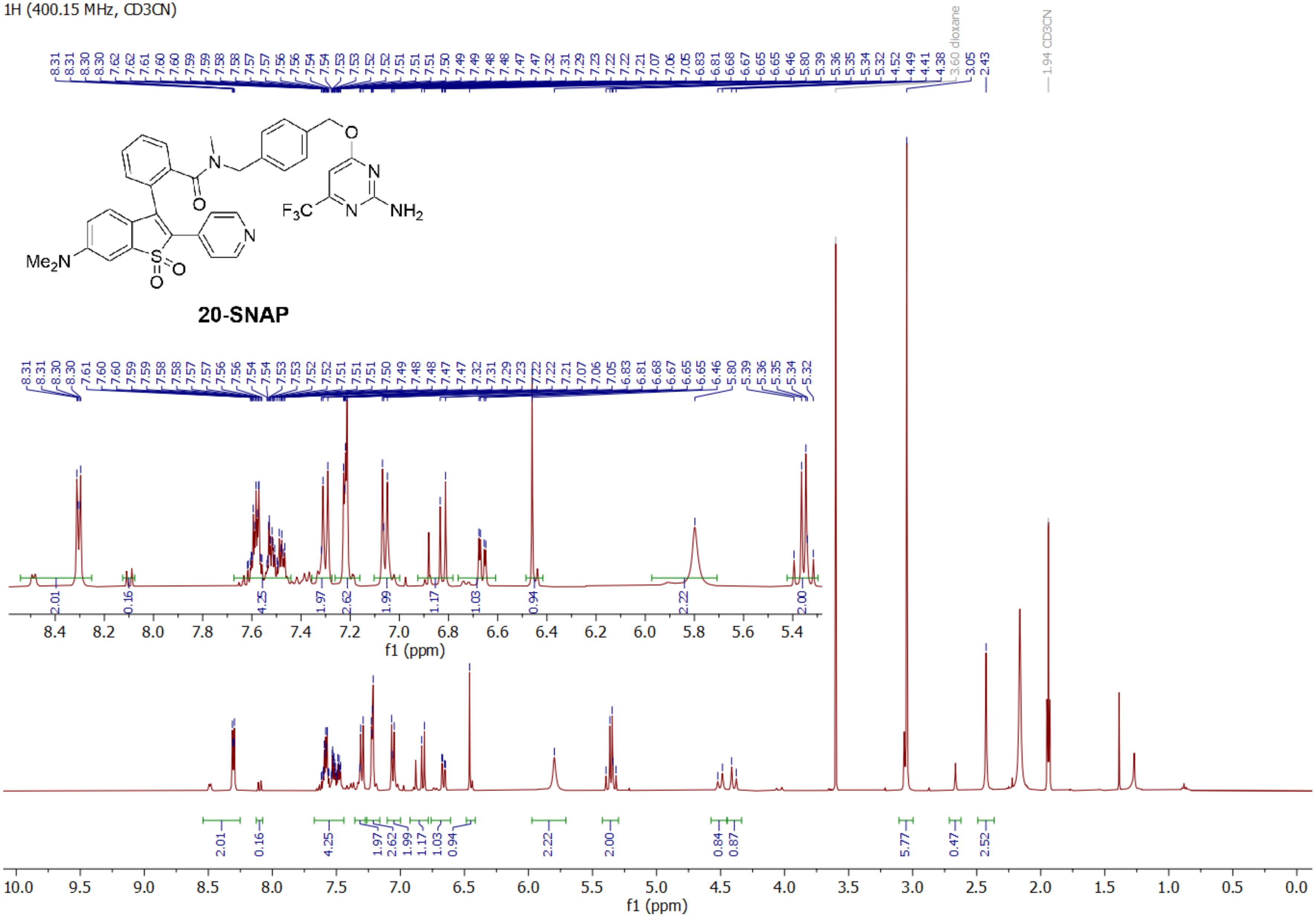
1H (400.15 MHz, CD3CN)
3.60 dioxane
1.94 CD3CN
8.31 8.31 8.30 8.30 7.62 7.61 7.60 7.60 7.59 7.59 7.58 7.58 7.57 7.57 7.56 7.56 7.54 7.54 7.53 7.53 7.52 7.52 7.51 7.51 7.51 7.50 7.49 7.49 7.48 7.48 7.47 7.47 7.47 7.32 7.31 7.29 7.23 7.22 7.22 7.21 7.07 7.06 7.05 6.83 6.81 6.68 6.67 6.65 6.65 6.46 5.80 5.39 5.36 5.35 5.34 5.32 4.52 4.49 4.41 4.38 3.05 2.43
2.01 0.16 4.25 1.97 2.62 1.99 1.17 1.03 0.94 2.22 2.00 0.84 0.87 5.77 0.47 2.52
f1 (ppm)

20-SNAP

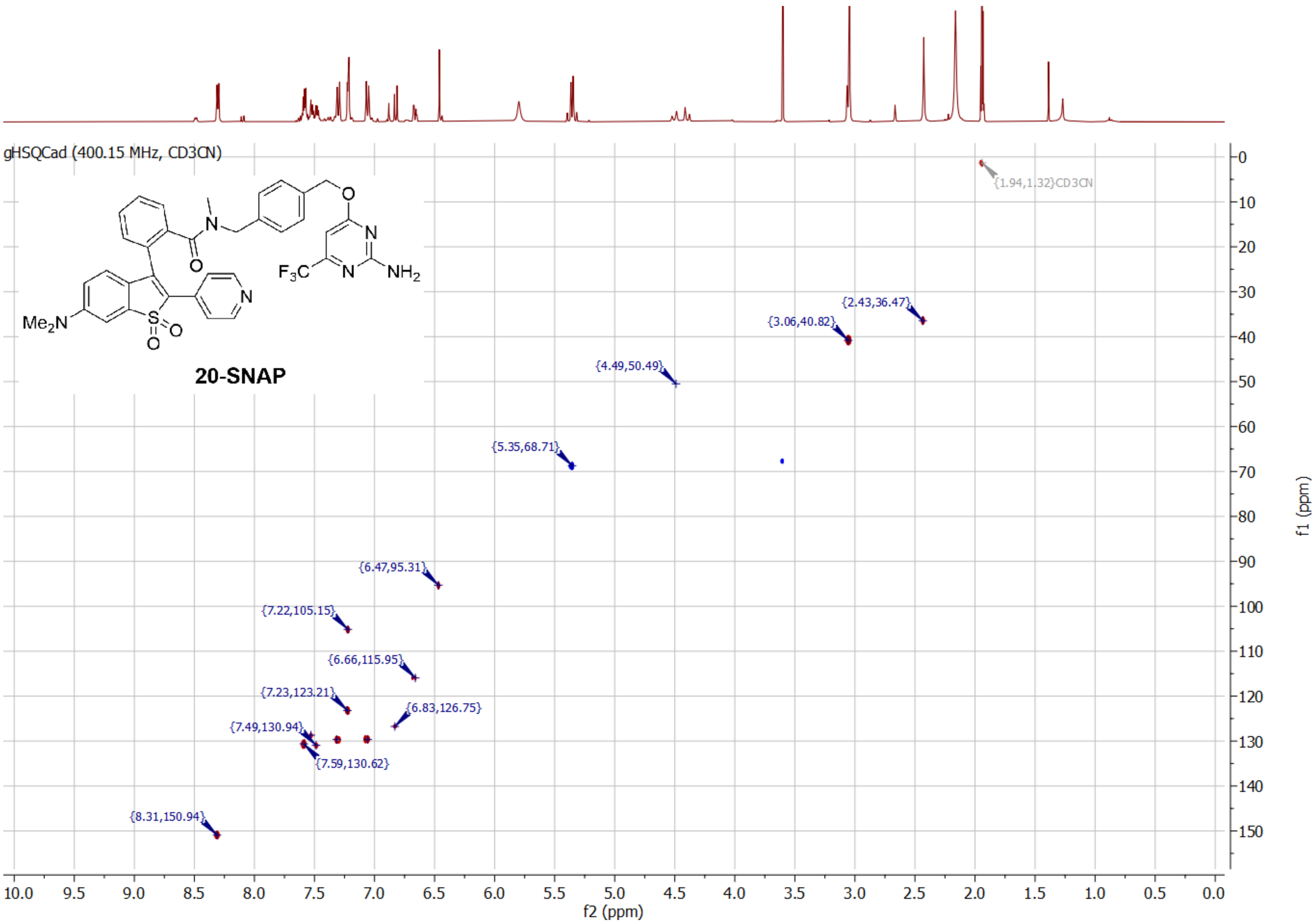
gHSQCad (400.15 MHz, CD3CN)
Me2N
S
O
O
N
O
N
O
N
N
F3C
NH2
20-SNAP
{1.94,1.32}CD3CN
{2.43,36.47}
{3.06,40.82}
{4.49,50.49}
{5.35,68.71}
{6.47,95.31}
{7.22,105.15}
{6.66,115.95}
{7.23,123.21}
{6.83,126.75}
{7.49,130.94}
{7.59,130.62}
{8.31,150.94}
f2 (ppm)
f1 (ppm)

19F (376.48 MHz, CD3CN)

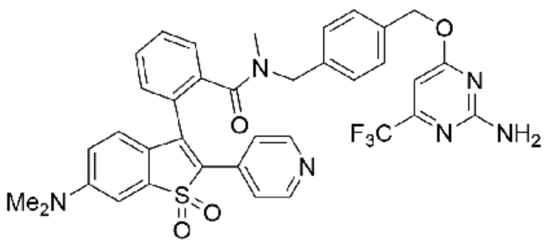

20-SNAP

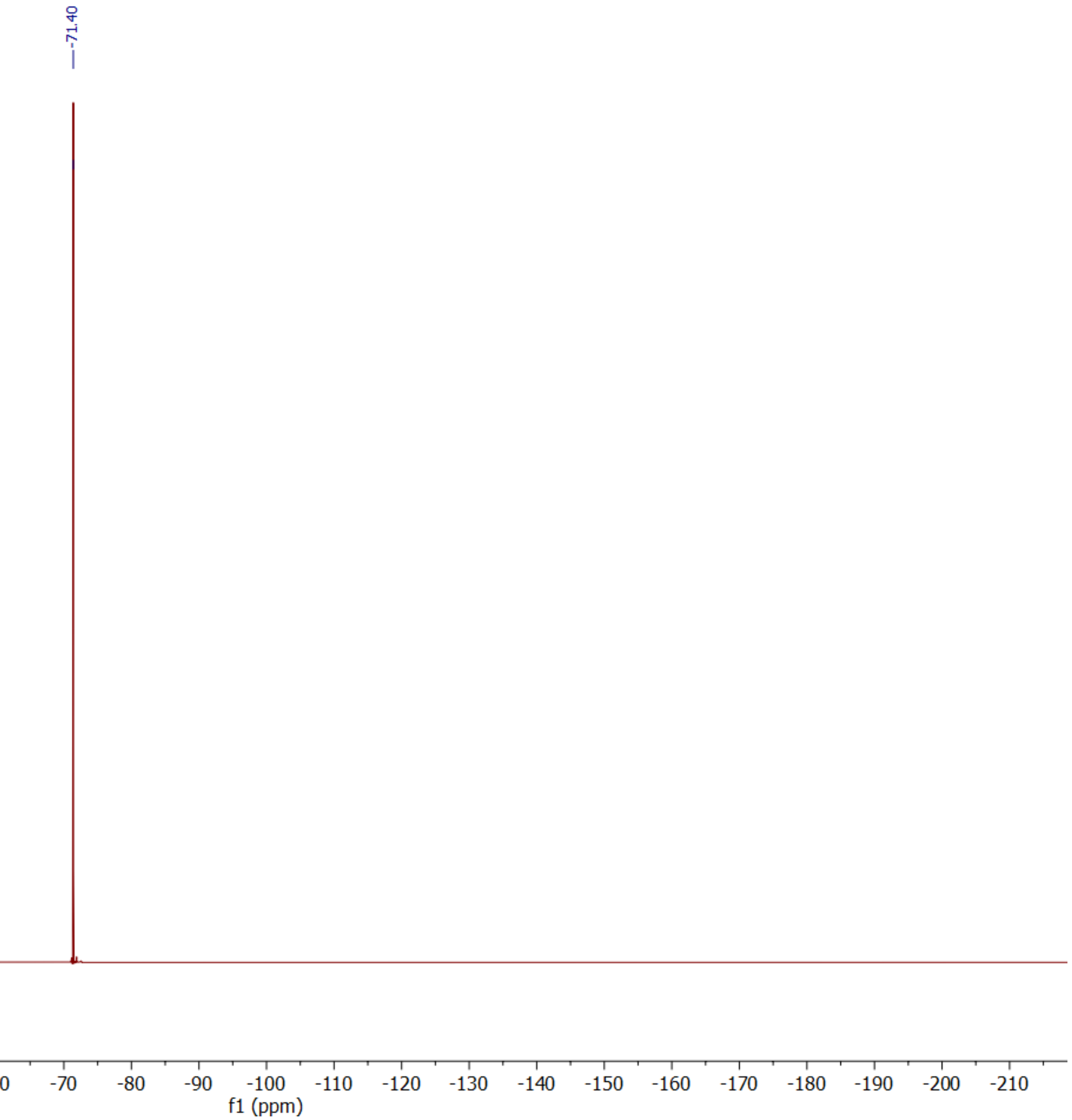

1H (400.15 MHz, CDCl3)

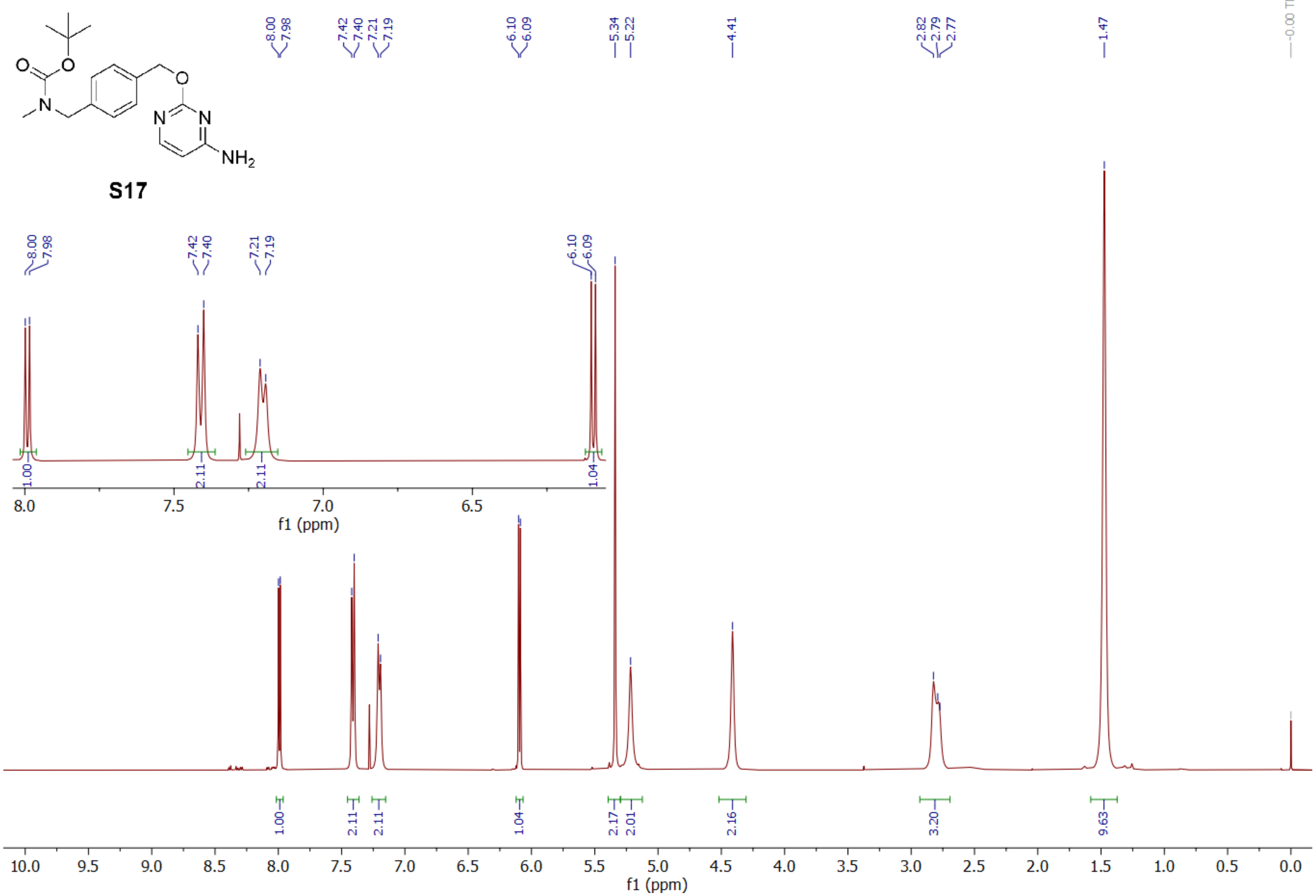

13C (100.63 MHz, CDCl3)

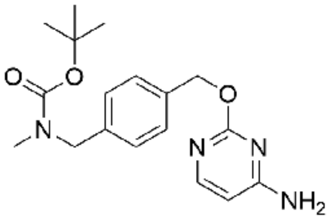

S17

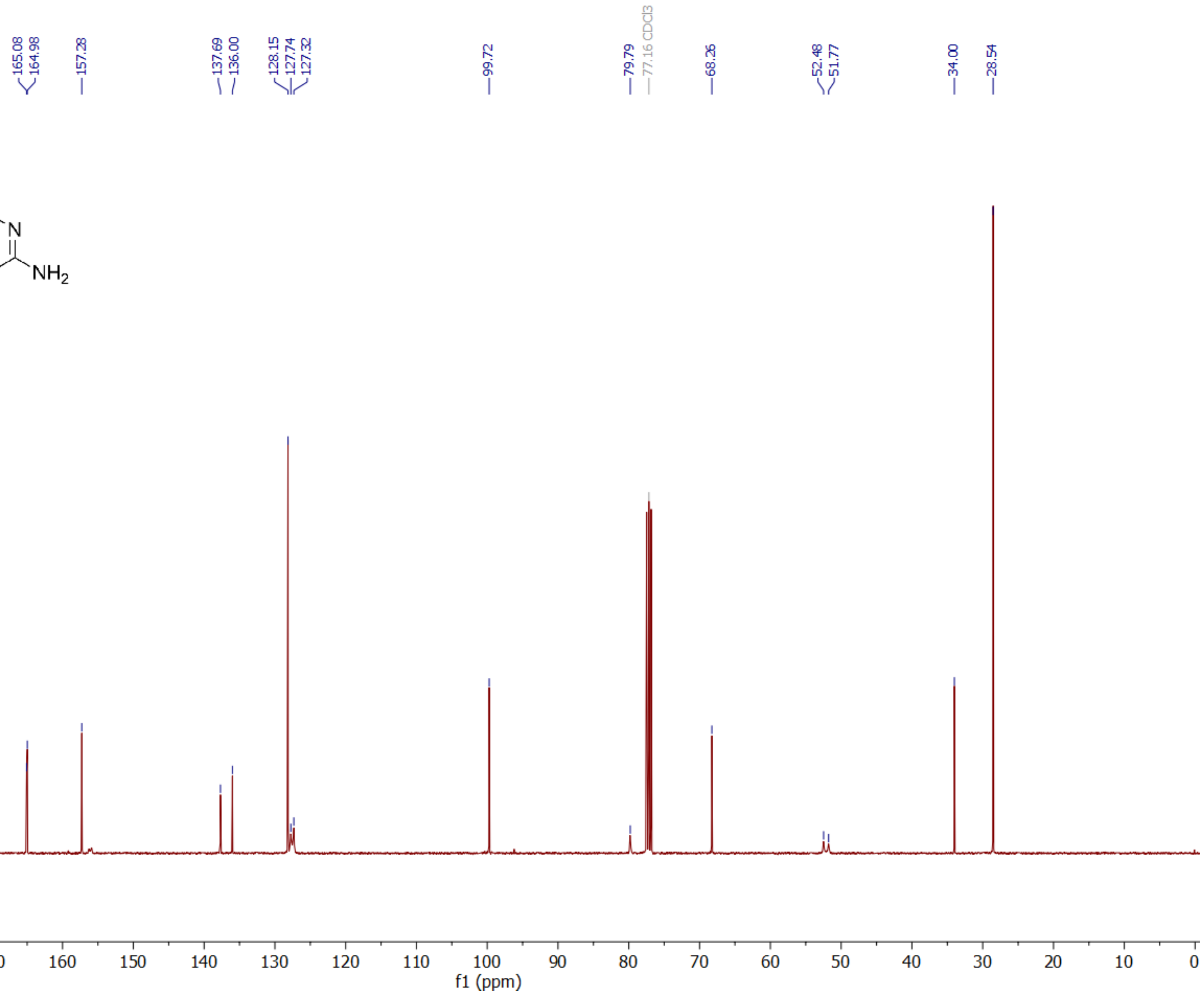

1H (400.15 MHz, DMSO)

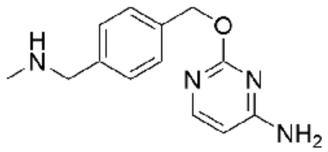

S18

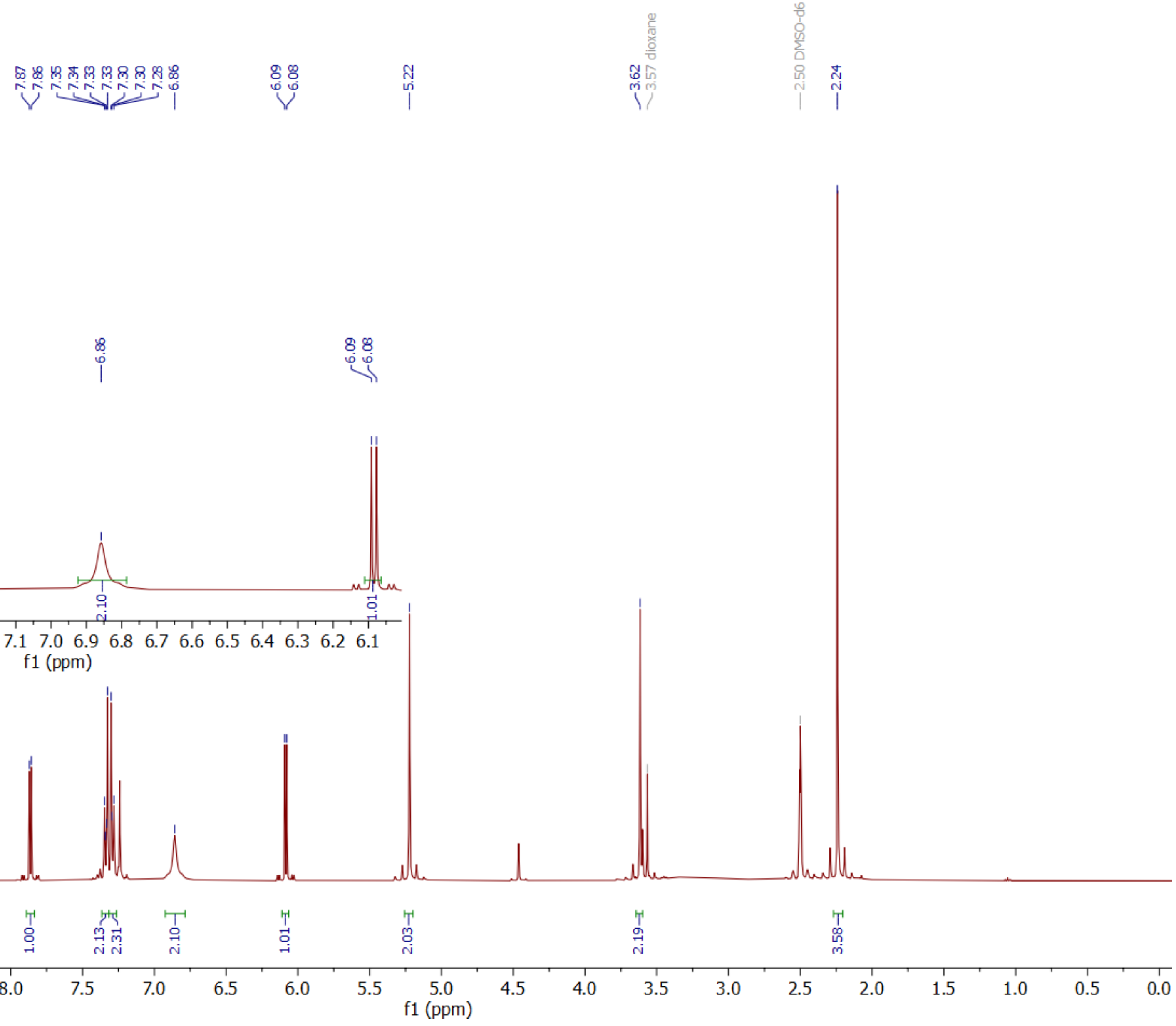

13C (100.63 MHz, DMSO)

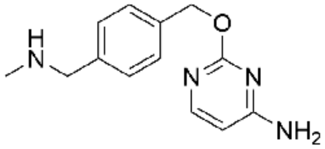

S18

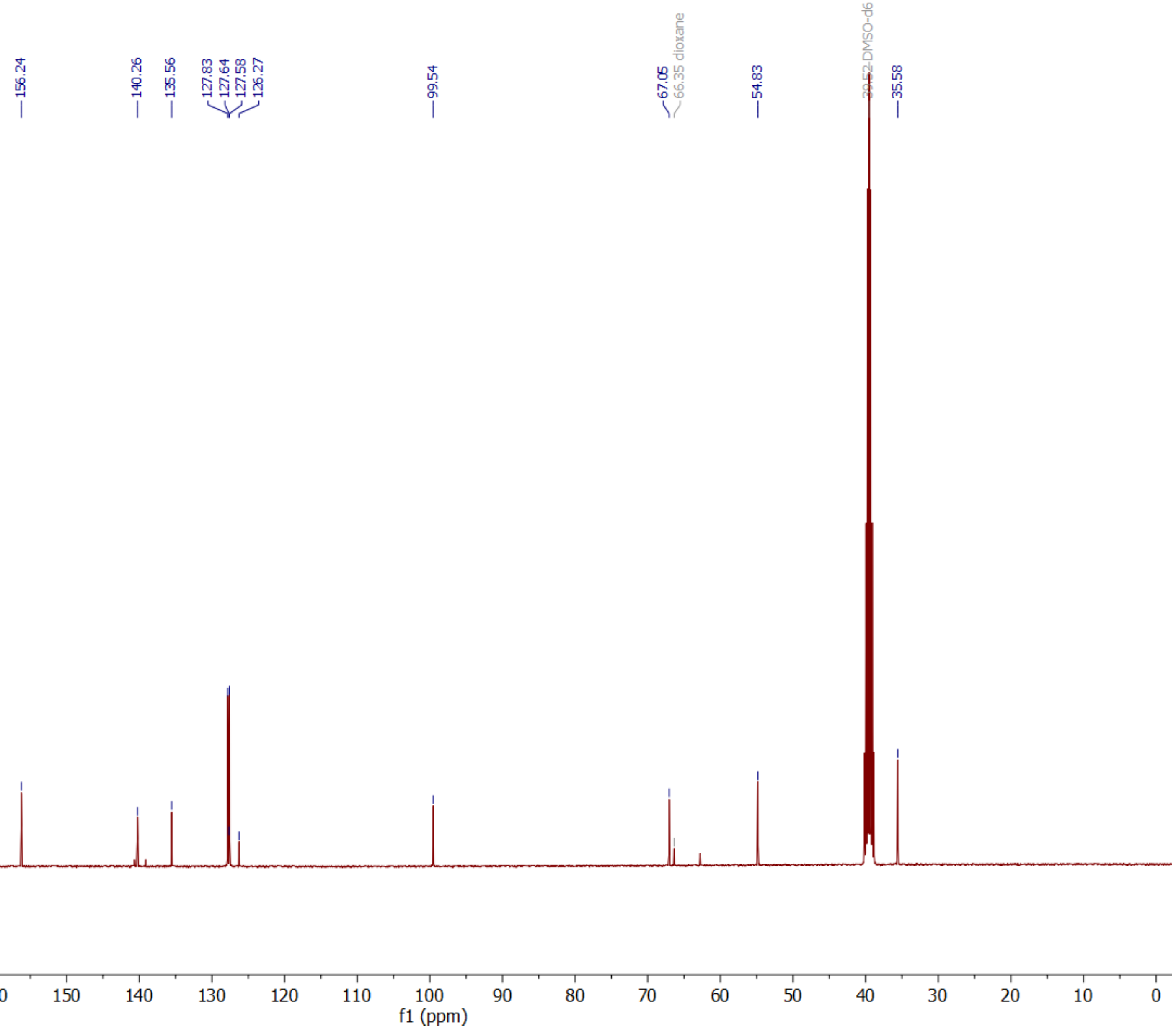

1H (400.15 MHz, DMSO)

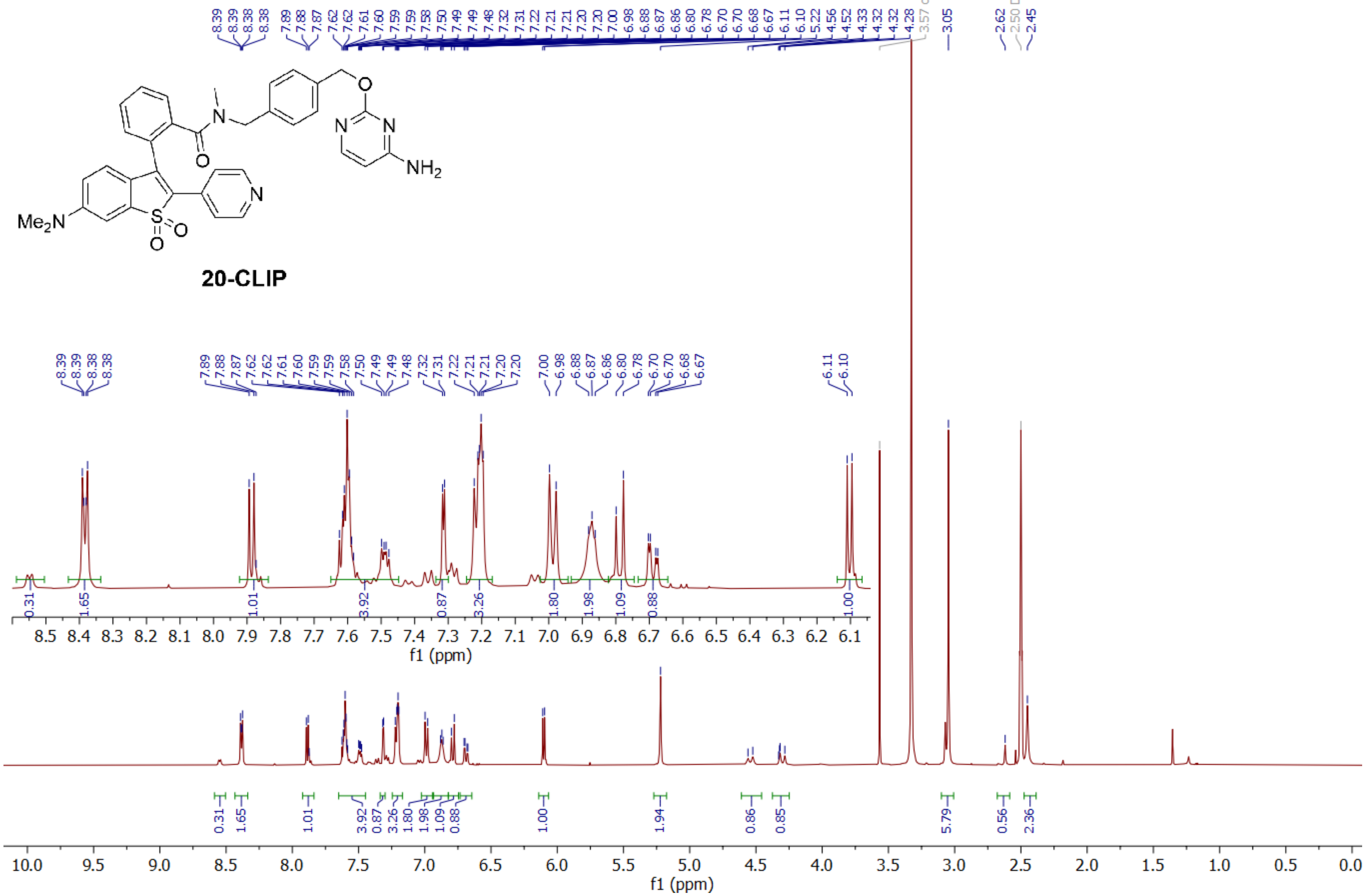

20-CLIP

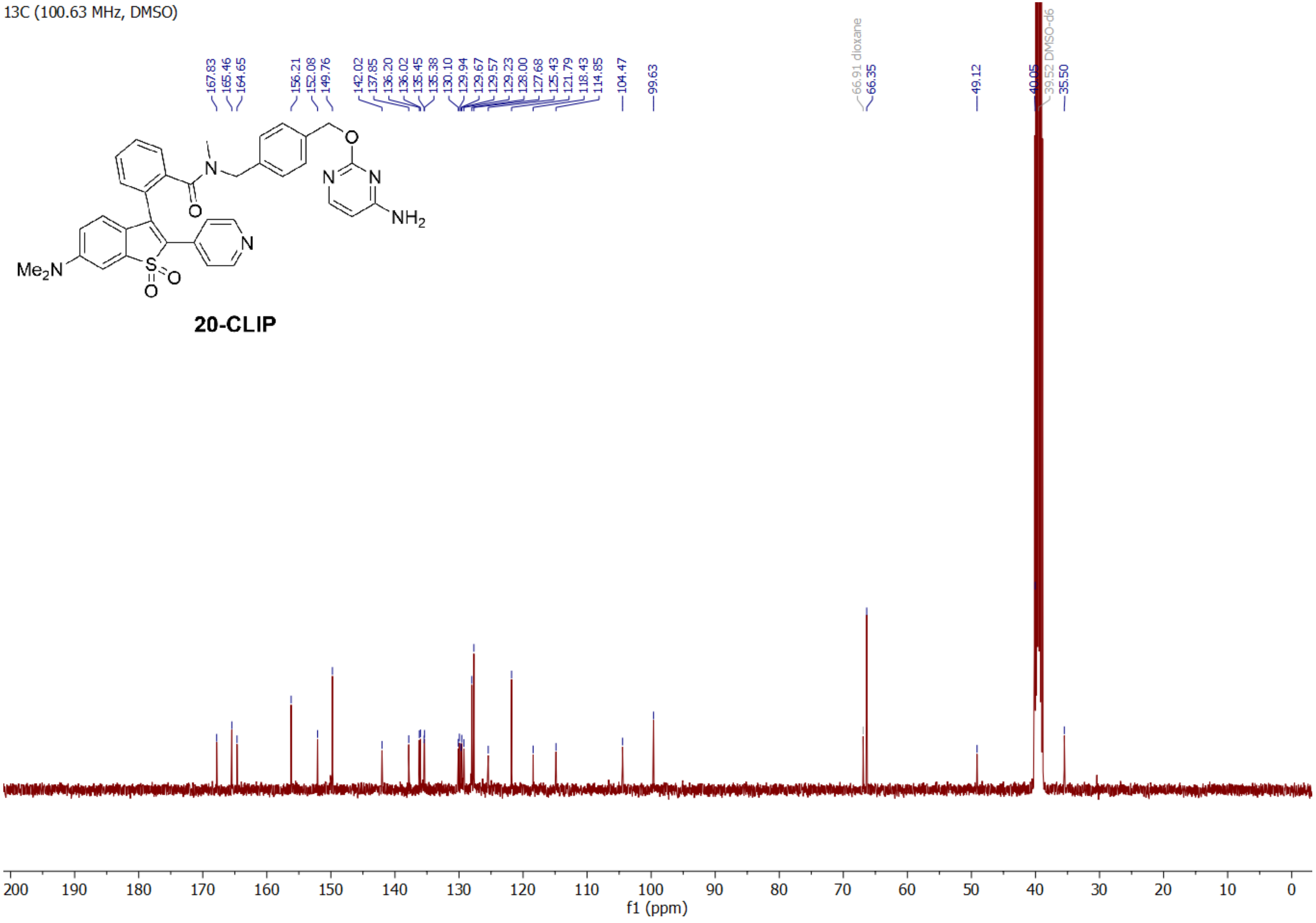
13C (100.63 MHz, DMSO)
167.83 165.46 164.65 156.21 152.08 149.76 142.02 137.85 136.20 136.02 135.45 135.38 130.10 129.94 129.67 129.57 129.23 128.00 127.68 125.43 121.79 118.43 114.85 104.47 99.63
66.91 dioxane 66.35 49.12 40.05 39.52 DMSO-d6 35.50
Me2N
O
N
NH2
20-CLIP
200 190 180 170 160 150 140 130 120 110 100 90 80 70 60 50 40 30 20 10 0
f1 (ppm)

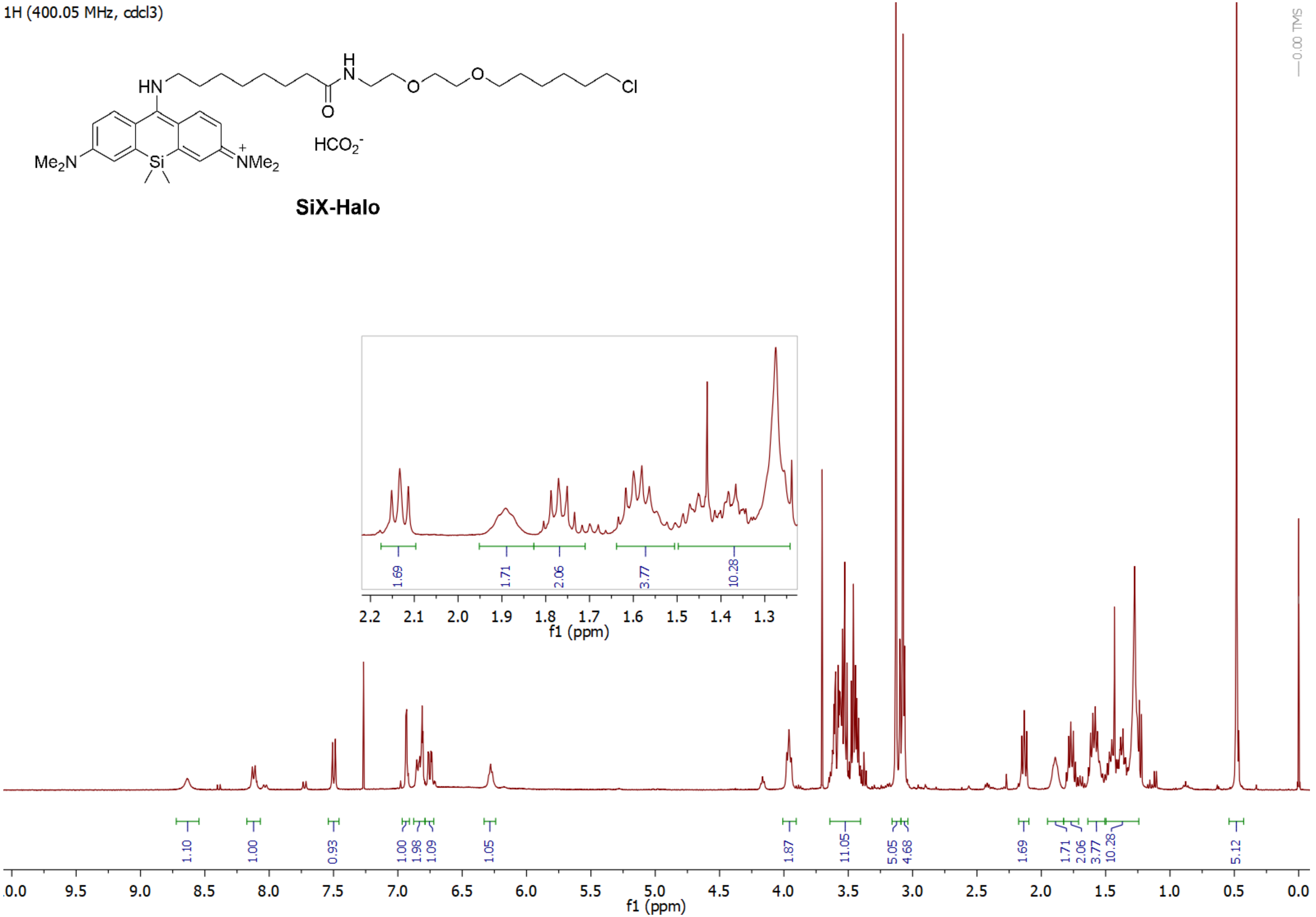
1H (400.05 MHz, cdcl3)
SiX-Halo
HCO2-
f1 (ppm)
—0.00 TMS

13C (100.60 MHz, cdcl3)

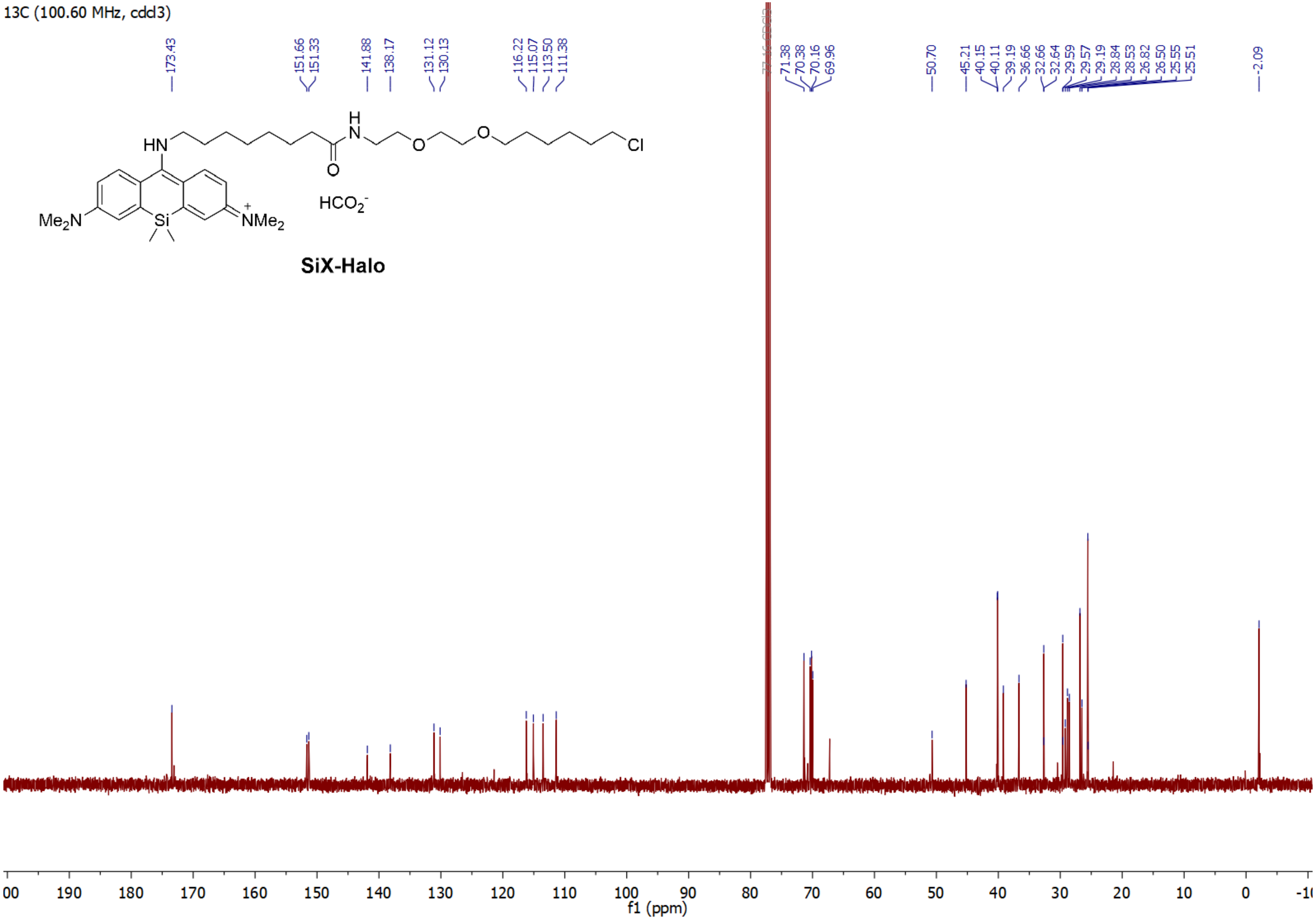

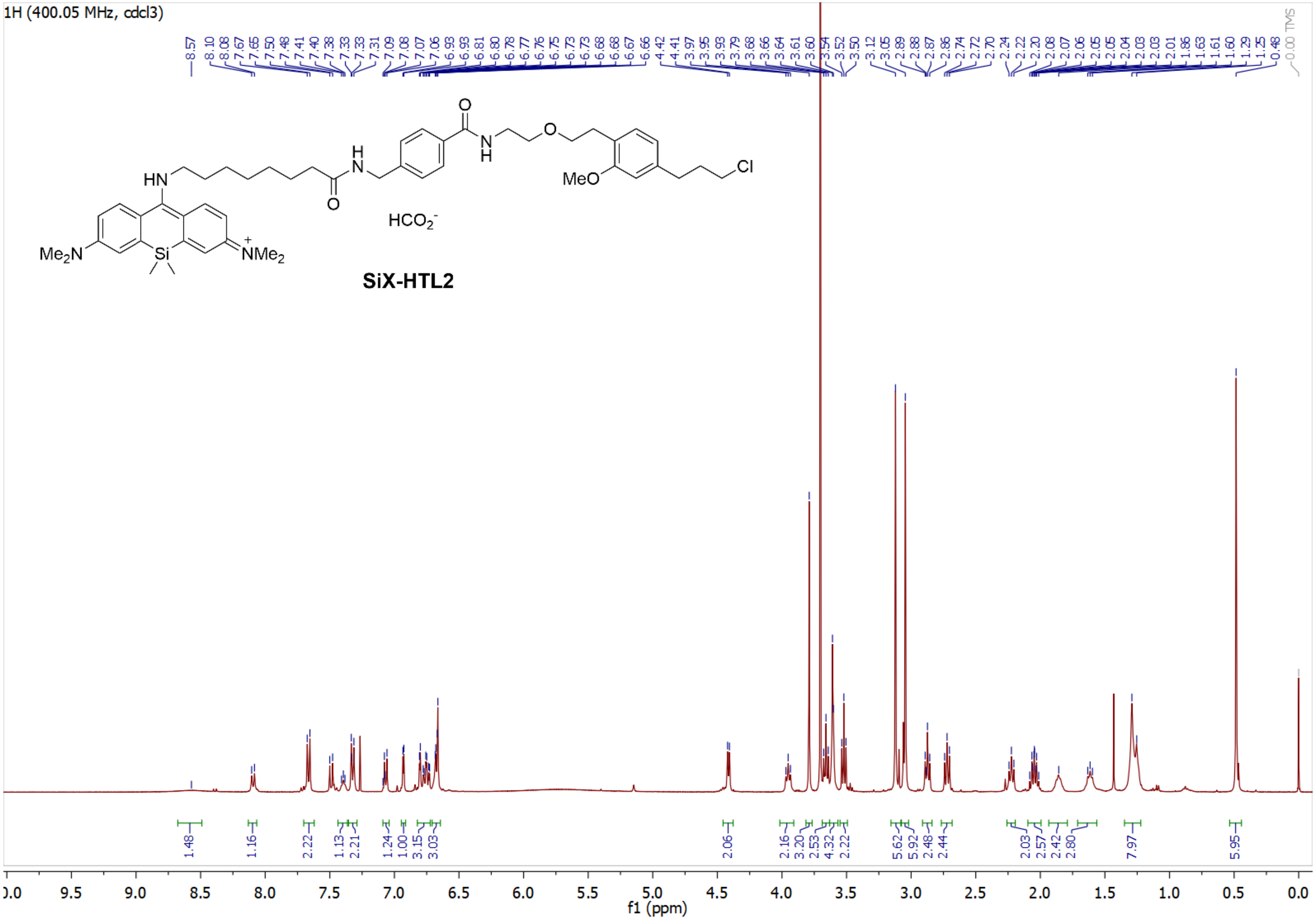

SiX-HTL2

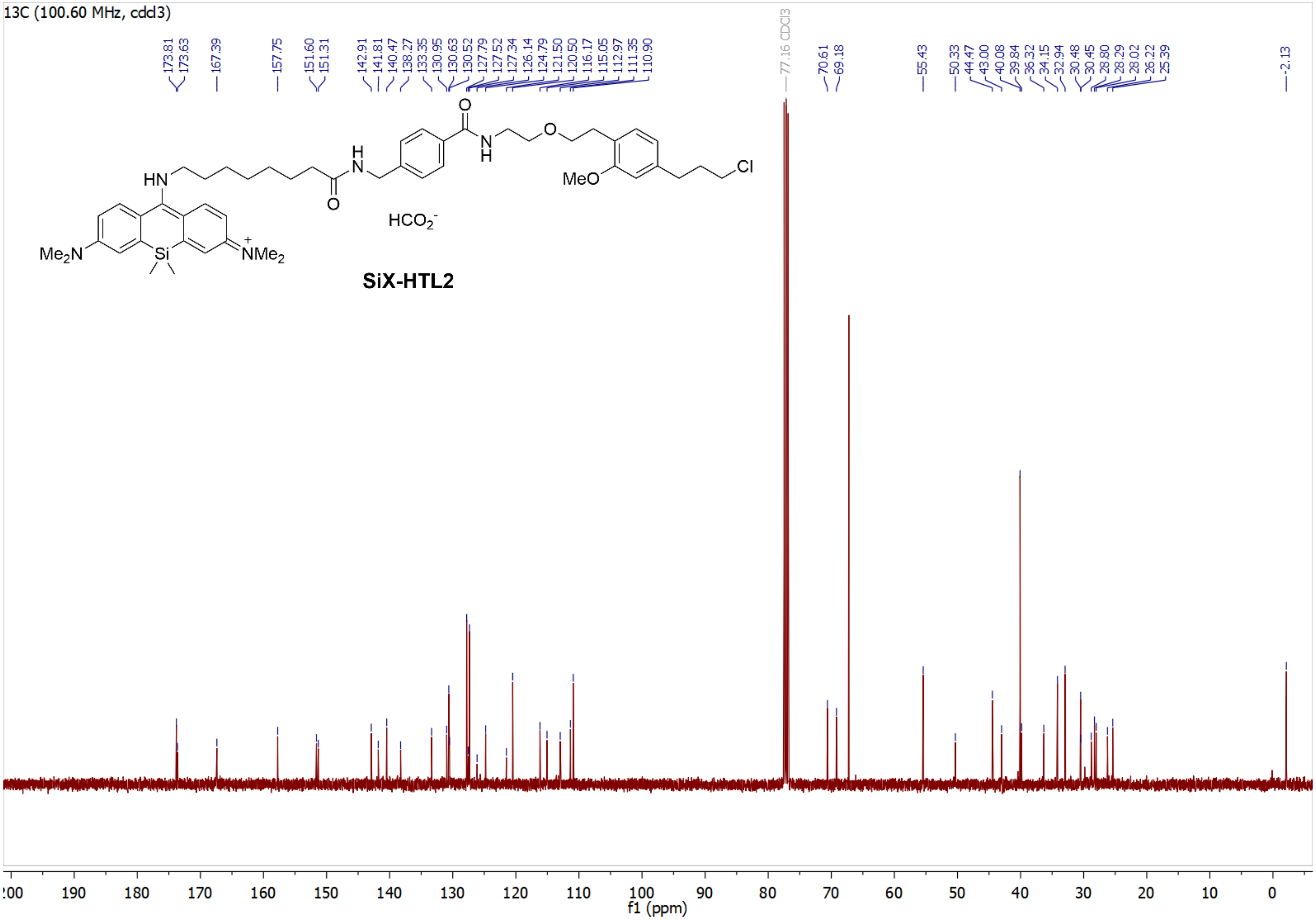

13C (100.60 MHz, cdcl3)
173.81
173.63
167.39
157.75
151.60
151.31
142.91
141.81
140.47
138.27
133.35
130.95
130.63
130.52
127.79
127.52
127.34
126.14
124.79
121.50
120.50
116.17
115.05
112.97
111.35
110.90
77.16 CDCl3
70.61
69.18
55.43
50.33
44.47
43.00
40.08
39.84
36.32
34.15
32.94
30.48
30.45
28.80
28.29
28.02
26.22
25.39
-2.13
HN
H
N
O
O
N
H
O
MeO
Cl
HCO_2^-
Me_2N
Si
$\overset{+}{N}Me_2$
SiX-HTL2
f1 (ppm)
